# Effects of ‘*Candidatus* Liberibacter solanacearum’ haplotypes A and B on tomato gene expression and geotropism

**DOI:** 10.1186/s12870-022-03505-z

**Published:** 2022-03-30

**Authors:** Kyle Harrison, Julien G. Levy, Cecilia Tamborindeguy

**Affiliations:** 1 Department of Horticultural Sciences, Texas A&M University, College station, TX 77843 USA; 2 Present address: USDA-ARS, Agroecosystem Management Research, Lincoln, NE 68503 USA; 3 Department of Entomology, Texas A&M University, College station, TX 77843 USA

**Keywords:** Psyllid, *Bactericera cockerelli*, Transcriptome, Lso haplotype, Gene expression, *Solanum lycopersicum* L., Transcriptomics, Plant-insect-microbe interactions, Potato, Psyllid, zebra chip, ‘*Candidatus* Liberibacter solanacearum’

## Abstract

**Background:**

The tomato psyllid, *Bactericera cockerelli* Šulc (Hemiptera: Triozidae), is a pest of solanaceous crops such as tomato (*Solanum lycopersicum* L.) in the U.S. and vectors the disease-causing pathogen ‘*Candidatus* Liberibacter solanacearum’ (or Lso). Disease symptom severity is dependent on Lso haplotype: tomato plants infected with Lso haplotype B experience more severe symptoms and higher mortality compared to plants infected with Lso haplotype A. By characterizing the molecular differences in the tomato plant’s responses to Lso haplotypes, the key components of LsoB virulence can be identified and, thus, targeted for disease mitigation strategies.

**Results:**

To characterize the tomato plant genes putatively involved in the differential immune responses to Lso haplotypes A and B, RNA was extracted from tomato ‘Moneymaker’ leaves 3 weeks after psyllid infestation. Gene expression levels were compared between uninfected tomato plants (i.e., controls and plants infested with Lso-free psyllids) and infected plants (i.e., plants infested with psyllids infected with either Lso haplotype A or Lso haplotype B). Furthermore, expression levels were compared between plants infected with Lso haplotype A and plants infected with Lso haplotype B. A whole transcriptome analysis identified 578 differentially expressed genes (DEGs) between uninfected and infected plants as well as 451 DEGs between LsoA- and LsoB-infected plants. These DEGs were primarily associated with plant defense against abiotic and biotic stressors, growth/development, plant primary metabolism, transport and signaling, and transcription/translation. These gene expression changes suggested that tomato plants traded off plant growth and homeostasis for improved defense against pathogens, especially when infected with LsoB. Consistent with these results, tomato plant growth experiments determined that LsoB-infected plants were significantly stunted and had impaired negative geotropism. However, it appeared that the defense responses mounted by tomatoes were insufficient for overcoming the disease symptoms and mortality caused by LsoB infection, while these defenses could compensate for LsoA infection.

**Conclusion:**

The transcriptomic analysis and growth experiments demonstrated that Lso-infected tomato plants underwent gene expression changes related to abiotic and biotic stressors, impaired growth/development, impaired plant primary metabolism, impaired transport and signaling transduction, and impaired transcription/translation. Furthermore, the transcriptomic analysis also showed that LsoB-infected plants, relative to LsoA-infected, experienced more severe stunting, had improved responses to some stressors and impaired responses to others, had poorer transport and signaling transduction, and had impaired carbohydrate synthesis and photosynthesis.

**Supplementary Information:**

The online version contains supplementary material available at 10.1186/s12870-022-03505-z.

## Background

‘*Candidatus* Liberibacter solanacearum’ (Lso) is a gram-negative bacterium responsible for several plant diseases in multiple plant families worldwide [1]. At least seven haplotypes have been identified [2–4]. LsoA and LsoB are two haplotypes transmitted by tomato psyllid *Bactericera cockerelli* Šulc (Hemiptera: Triozidae) that infect solanaceous plants in the Americas [5, 6]. Haplotypes LsoC, LsoD, and LsoE are transmitted by carrot psyllids *Trioza apicalis* Foerster and *B. trigonica* Foerster and infect apiaceous crops in Europe, North Africa, and the Middle East [7–11]. The diversity of Lso haplotypes, the diversity of insect vectors, and the economic significance of host plants make these systems valuable for studying plant-insect-microbe genetic interaction. In addition, the potato disease associated with Lso, called ‘zebra chip’, is responsible for millions of dollars in annual losses to the potato industry, meaning the relationship between Lso and its host plants should be studied to improve disease management practices [12].

Disease symptoms related to Lso infection are characterized by long latent periods in tomato and potato plants and typically develop after 3 weeks following infection [13–15]. In potato plants, disease symptoms associated with Lso haplotypes A and B are similar (i.e., wilting, chlorosis, leaf curling, tuber discoloration, and premature death), but LsoB-infected plants experience significantly higher rates of mortality and exhibit more severe tuber discoloration [16]. In tomatoes, however, symptoms associated with each Lso haplotype are significantly different: LsoA infection results in stunting, wilting, and chlorosis but the plants remain alive and still produce fruit, while LsoB infection invariably kills the plant prematurely, displaying severe stunting, wilting, and chlorosis, [17, 18]. Notably, Lso A and B distribution and titer within tomato plants are similar during early infection, but after 5 weeks, they differ significantly [18]. Furthermore, similar haplotype-specific differences in Lso disease symptom severity were recently identified in tobacco plants (Levy et al., unpublished). Since solanaceous crops are apparently more susceptible to LsoB than LsoA, tomato plants are likely responding to each haplotype differently. By characterizing the molecular differences in the tomato plant’s responses to Lso haplotypes, key components associated with susceptibility to Lso could be identified and, thus, targeted for disease mitigation strategies.

The current study evaluated and compared the transcriptomic and growth responses of tomato plants to infection by different Lso haplotypes, A and B. A whole transcriptome analysis compared gene expression levels between uninfested (control) and Lso-free psyllid-infested plants (LsoFree) (i.e., uninfected plants) to plants that had been infested with psyllids infected with either Lso haplotype A (LsoA) or haplotype B (LsoB) (i.e., infected plants). Furthermore, gene expression levels were likewise compared between plants infested with LsoA-infected psyllids and plants infested with LsoB-infected psyllids. This experimental design separated the gene expression levels associated with either normal homeostasis or psyllid infestation from the gene expression levels associated with Lso infection as well the unique gene expression levels associated with infection by each Lso haplotype. This study was the first to characterize a tomato plant’s molecular responses to different haplotypes of a Liberibacter pathogen. However, prior research suggested that genes involved in jasmonic acid-related pathways (particularly those involved in stress responses), carbohydrate metabolism, and auxin signaling underwent expression changes in the face of Lso challenge [19, 20]. These studies suggested that some of the observed symptoms associated with Lso infection (i.e., chlorosis and wilting) could be attributed to these expression changes. The presented research identified genes most likely involved in the tomato plant’s resistance to Liberibacter haplotypes. The hypotheses were: if tomato plants mount differential responses to Liberibacter haplotypes, then 1) a significant number of DEGs should be identified between uninfected and infected tomato plants (verifying prior research) and 2) a significant number of DEGs should be identified between tomato plants infected with different Lso haplotypes. In both cases, the expression changes observed after Lso infection were expected to be related to plant defense and stress response, carbohydrate metabolism, and auxin signaling. In addition, the role of auxin signaling in Lso symptomology was tested using growth experiments.

## Results

### 1-Transcriptomic analysis of Lso infected and uninfected plants

**Fig. 1:**
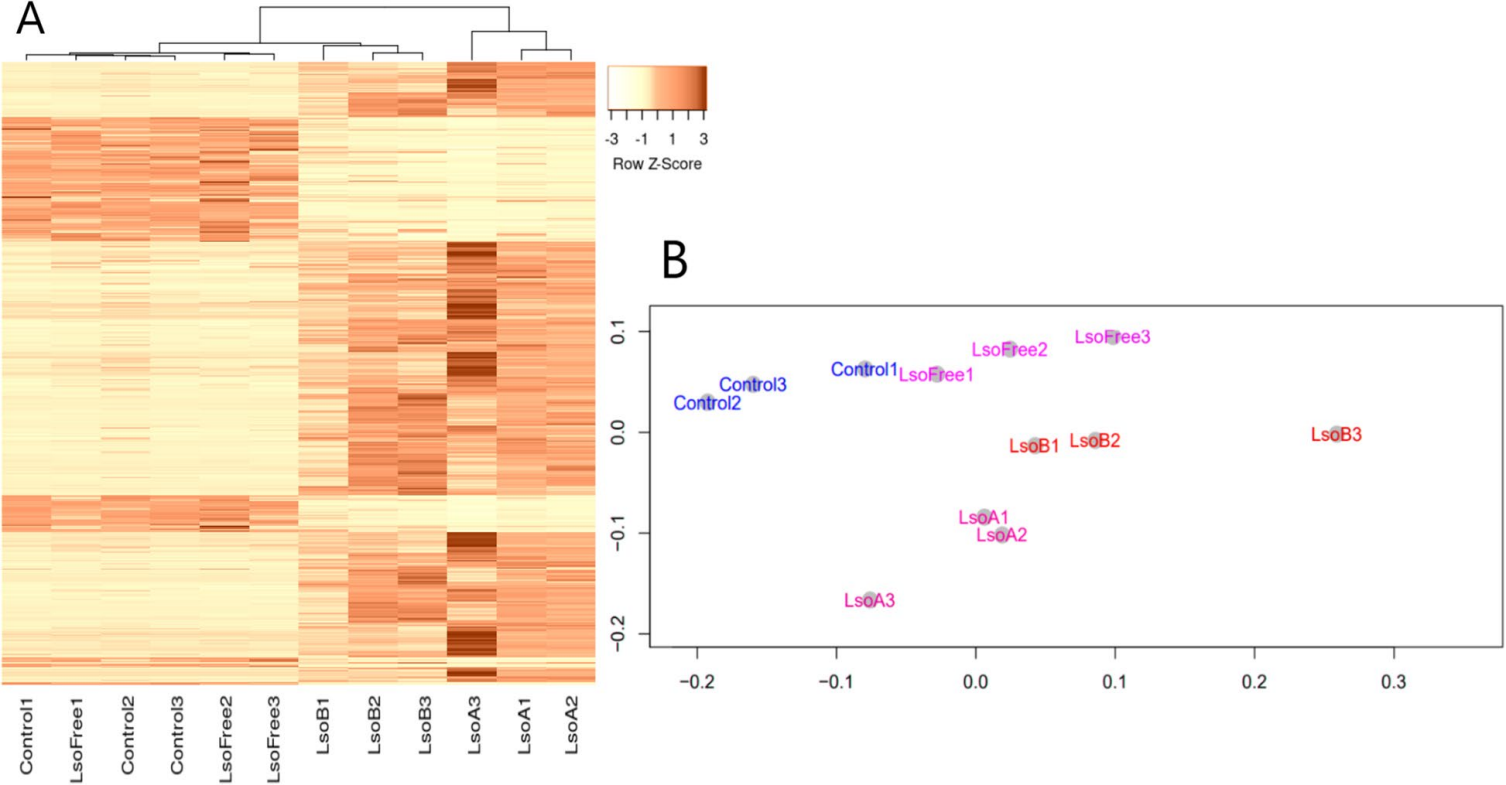
**A** Comparative heatmap of relative expression changes among uninfested (Control#), Lso-free psyllid infested (LsoFree#), LsoB-infected (LsoB#), and LsoA-infected (LsoA#) tomato plant DEGs. Dark colors denote down-regulation and light colors denote up-regulation. Lines above the heatmap depict the phylogenetic hiearchy among similar treatments and similar gene expression levels. **B** Multidimensional scaling (MDS) distance plot of fragments per kilobase per million reads (fpkm) among treatments: Uninfested (Control#), Lso-free psyllid infested (LsoFree#), LsoB-infected (LsoB#), and LsoA-infected (LsoA#)

Illumina sequencing of tomato cDNA libraries produced 297.7 million total reads that met FastQC criteria (i.e., Phred quality scores > 35). The average number of reads per library obtained from control plants (17,424,984 ± 998,723), Lso-free plants (18,095,563 ± 654,081), LsoA-infected plants (33,319,333 ± 340,077), and LsoB-infected plants (30,397,081 ± 4,549,117) were significantly different between uninfected and infected treatments, but not between control-vs-Lso-free plants or LsoA-infected-vs-LsoB-infected plants (f-ratio = 60.93; *p* < 0.001). HISAT2 alignment analysis also showed that, on average, 96 ± 0.24% of all reads from control plants, 96 ± 0.43% of reads from Lso-free plants, 94 ± 0.11% of reads from LsoA-infected plants, and 95 ± 0.70% of reads from LsoB-infected plants mapped to vSL3.0 of the *S. lycopersicum* genome (Supplementary Table 1); these alignment rates were significantly different between treatments, except for control-vs-Lso-free plants (f-ratio = 25.57; *p* < 0.01). The Ballgown analysis, which is an R package that provides functions to organize, visualize, and analyze the expression measurements of transcriptome assemblies [21], demonstrated that the distribution of fragments per kilobase per million read (or fpkm) values were uniformly distributed among the submitted libraries (Supplementary Fig. 1) and that transcript lengths were geometrically distributed (Supplementary Fig. 2). The Ballgown analysis identified 578 differentially expressed genes (DEGs) between uninfected (control and Lso-free combined) and Lso-infected plants (LsoA and LsoB combined) (*p*-value < 0.05; Supplementary Table 2). These DEGs represented the pattern of systemic tomato plant gene expression following Lso infection and visualized with a heatmap comparing the relative fold change (Z-score) for each gene between samples (Fig. 1). Z-scores were calculated based on significant deviations from the average fpkm value for each gene across all libraries. Also based on these fpkm values, a dendrogram and heatmap (Fig. 1A) and a multidimensional scaling (MDS) distance plot (Fig. 1B) were used to visualize relative similarities in gene expression levels across samples. Both the dendrogram and the MDS distance plot geometries suggested that the overall pattern of gene expression was consistent within each treatment, where per-gene fpkm values were most similar within treatment and most different between treatments (except for one Lso-free plant, whose expression was more like control plants), although differences between control and Lso-free plants were small. Furthermore, the MDS distance plot demonstrated a strong separation between the fpkm values derived from uninfected and Lso-infected plants (Supplementary Fig. 3). The MDS distance plot also demonstrated weak separations between control and Lso-free psyllid-infested plants as well as a separation between LsoA- and LsoB-infected plants. These results suggested that overall gene expression was most different between uninfected and infected plants but also suggested that psyllid infestation and Lso haplotype have significant consequences for tomato plant gene expression [22]. This interpretation is supported by the binomial distribution of differential expression (DE) values among all sample libraries (Supplementary Fig. 4).

**Fig. 2:**
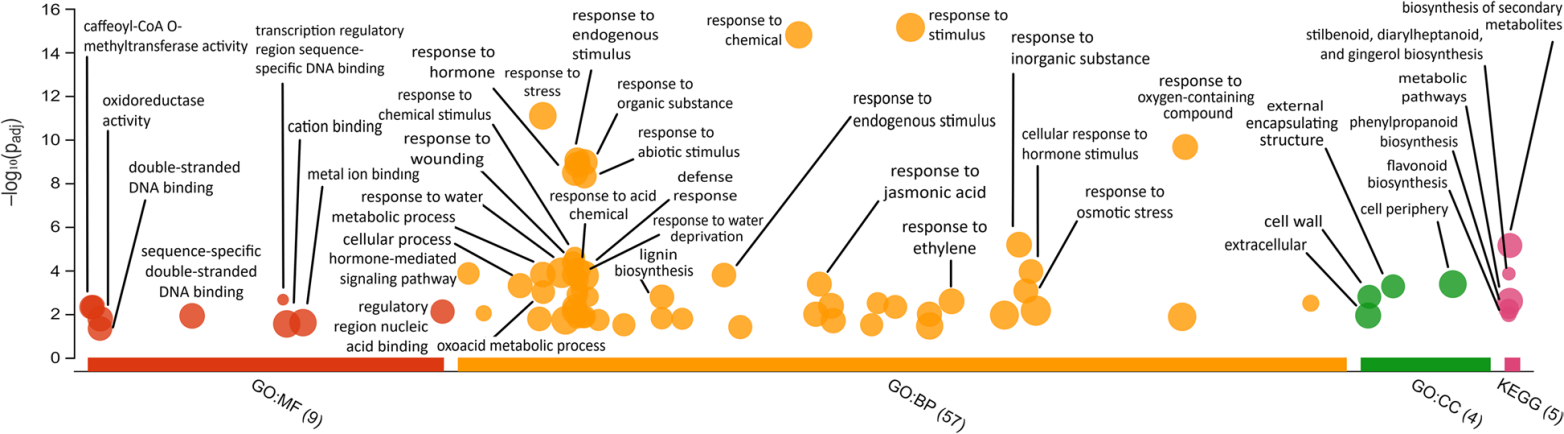
g:Profiler analysis of Lso-infected tomato plant DEG homologs depicting their relative overrepresentation among *Arabidopsis* molecular functions (MF), biological processes (BP), or cellular components (CC). The left axis represents the -log_10_(p_adj_) likelihood that a given MF, BP, or CC would be associated with a random selection of *Arabidopsis* genes. Circle sizes represent the relative number of times a given MF, BP, CC, or KEGG term appears among analyzed genes. In general, expression changes occurred throughout the cell and were most likely to be involved with response to stress or stimulus, biosynthesis of secondary metabolites, methyltransferases, and DNA binding (likely involved in expression regulation). Labels above, connected to arrows, or adjacent to circles describe specific the MF, BP, or CC associated with each circle; some labels have been omitted due to redundancy

Among the 578 DEGs between Lso-infected and uninfected plants, 416 (72%) were up-regulated in Lso-infected tomato plants relative to uninfected ones. On average, up-regulated genes were expressed at levels 3.2 ± 1.4 fold-change higher, while down-regulated genes were expressed 2.7 ± 0.8 fold-change lower. Among all DEGs, 344 (60%) involved a tomato gene or gene homolog that was sufficiently characterized for the purpose of assigning putative functions. In addition, 238 (41%) were involved in a KEGG pathway (Supplementary Table 3). Gene homologs came from different model organisms including *Arabidopsis thaliana,* corn, potato, rice, or tobacco. The g:Profiler analysis (https://biit.cs.ut.ee/gplink/l/3U_I4_TmSL) showed 245 DEGs (42%) could be assigned to two or more *A. thaliana* GO functional categories (Fig. 2; See Supplementary Table 4 for details). Based on the GO functional analysis, the KEGG analysis, and direct comparisons of tomato genes to homologs characterized in model systems, DEGs were assigned to one or more of the following broader categories for the purpose of correlating tomato plant gene expression with its (putative) physiological response to Lso infection: response to biotic and/or abiotic stress (139 DEGs, Supplementary Table 5), growth and development (116 DEGs, Supplementary Table 6), primary metabolism (98 DEGs, Supplementary Table 7), signaling and transport (84 DEGs, Supplementary Table 8), and transcription/ translation regulation (44 DEGs, Supplementary Table 9). Among these, several sub-categories appeared overrepresented among DEGs: defense response to pathogens (34 DEGs), cell wall structure and development (24 DEGs), response to drought stress and/or water transport (14 DEGs), metal ion transport and/or signaling (10 DEGs), lignin metabolism (9 DEGs), response to auxin signaling (8 DEGs), and defense response to herbivory (7 DEGs).

### 2-Transcriptomic comparison of LsoA- and LsoB-infected plants

**Fig. 3:**
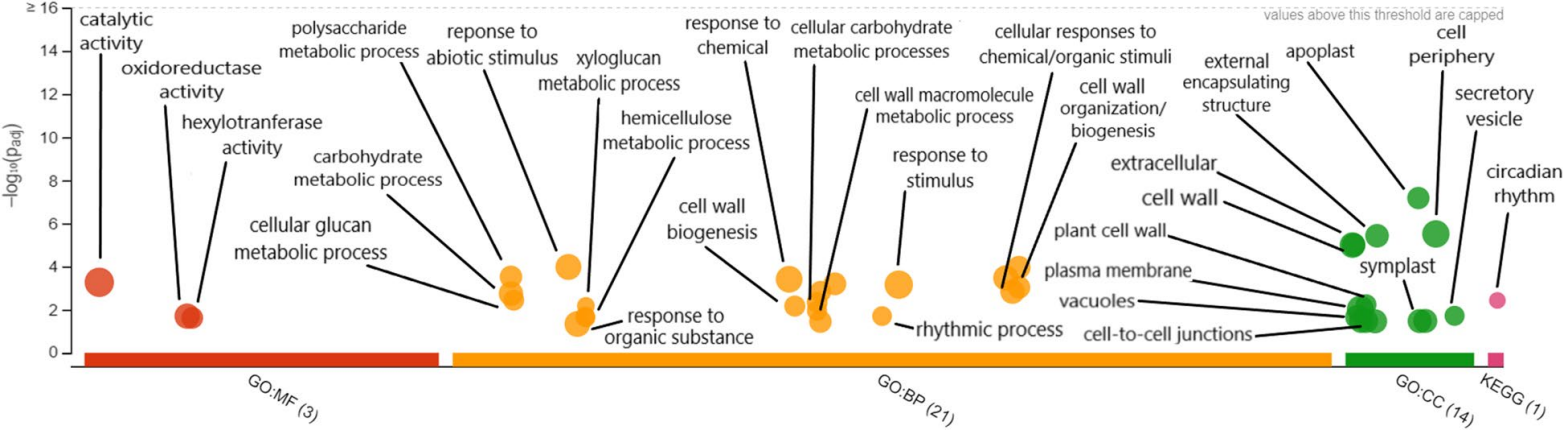
g:Profiler analysis of Lso haplotype-specific tomato plant DEG homologs depicting their relative overrepresentation among *Arabidopsis* molecular functions (MF), biological processes (BP), or cellular components (CC). The left axis represents the -log_10_(p_adj_) likelihood that a given MF, BP, or CC would be associated with a random selection of *Arabidopsis* genes. Circle sizes represent the relative number of times a given MF, BP, CC, or KEGG term appears among analyzed genes. In general, expression changes occurred throughout the cell and were most likely to be involved with response to stress or stimulus, biosynthesis of secondary metabolites, methyltransferases, and DNA binding (likely involved in expression regulation). Labels above, connected to arrows, or adjacent to circles describe specific the MF, BP, or CC associated with each circle; some labels have been omitted due to redundancy

In addition to the genes differentially expressed between infected and uninfected tomato plants, the Ballgown analysis revealed a set of 451 DEGs when comparing tomato plants infected with different Lso haplotypes (*p* < 0.05; Supplementary Table 10; Supplementary Fig. 5). Only 65 (14%) of these DEGs were simultaneously part of the 578 gene set differentially expressed between uninfected and infected plants. This suggested that the tomato plant response to Lso haplotype is largely independent of the plant’s overall response to Lso infection. Among the genes differentially expressed between plants infected with different Lso haplotypes, 261 (68%) were down-regulated in LsoB-infected plants compared to LsoA-infected plants. On average, up-regulated genes were expressed at levels 1.7 ± 0.7 fold-change higher in LsoB-infected plants, while down-regulated genes were expressed 1.6 ± 0.6 fold-change lower. The g:Profiler analysis (https://biit.cs.ut.ee/gplink/l/zkYmj7-eQ 9) showed 210 DEGs (68%) could be assigned to two or more *A. thaliana* GO functional categories (Fig. 3; See Supplementary Table 4 for details). In addition, (37%) were involved in a KEGG pathway (Supplementary Table 11). Based on these results and direct comparisons of tomato genes to their homologs, DEGs were assigned to one or more of the following broader categories for the purpose of correlating tomato plant gene expression with its (putative) physiological responses to different Lso haplotypes: growth and development (89 DEGs, including 9 specifically involved in circadian rhythm, Supplementary Table 12), response to abiotic/biotic stress (61 DEGs Supplementary Table 13), signaling and/or transport (48 DEGs, Supplementary Table 14), and carbohydrate metabolism (34 DEGs, including 16 specifically involved in photosynthesis, Supplementary Table 15). In addition, 24 of the DEGs related to growth and development were specifically involved in plant cell wall metabolism, and 17 of the DEGs related to signaling/transport involved either auxin (12 DEGs) or abscisic acid (5 DEGs).

### 3-validation of bioinformatic results by Rt-qPCR

**Fig. 4:**
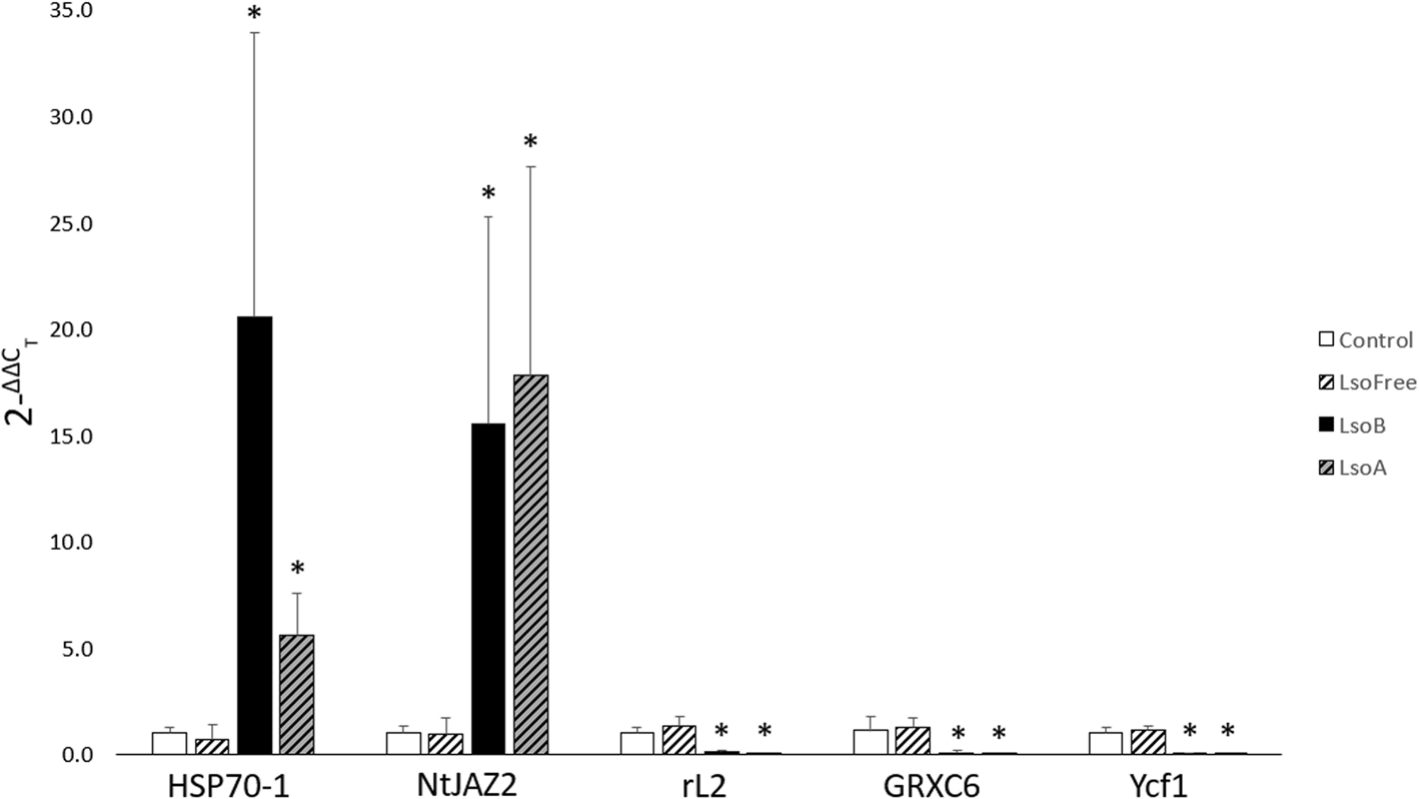
RT-qPCR results comparing 2^-ΔΔCT^ values between psyllid treatment groups: Control plants (solid white), Lso-free plants (white with black stripes), LsoB-infected plants (solid black), and LsoA-infected plants (grey with black stripes). Numbers listed above each column represent the average fpkm (fragments per kilobase of transcript per million reads) values calculated for the given treatment and target gene. Target DEGs were selected based on their expected relative expression levels between treatments: Heat shock cognate 70 kDa protein 1 (HSP70–1; Solyc06g076020.3) was expected to be up-regulated in LsoA-infected plants and expressed at even higher levels in LsoB-infected plants, jasmonate ZIM-domain protein 2 was expected to be up-regulated in Lso-infected plants (NtJAZ2; Solyc12g009220.2), ribosomal protein L2 was expected to be down-regulated in infected plants (rL2; Solyc10g047090.2), glutaredoxin-C6 was expected to be down-regulated in infected plants (GRXC6; Solyc11g069940.1), and Ycf1 was expected to be down-regulated in infected plants (Ycf1; Solyc12g035550.1). An asterisk denotes a significant difference with control plants

RT-qPCR analyses were performed for five DEGs (i.e., Solyc06g076020.3, Solyc12g009220.2, Solyc10g047090.2, Solyc11g069940.1, and Solyc12g035550.1) in plants grown independently of the plants submitted for transcriptomic analysis. Expression levels in these genes were congruent with the relative expression levels described in the transcriptomic analysis (Fig. 4). For example, Solyc06g076020.3 (or HSP70–1) was expressed at similar levels in control (1.0 ± 0.24) and Lso-free infested plants (0.7 ± 0.7), while being significantly over expressed in LsoB- (20.5 ± 13.4) and LsoA-infected plants (5.6 ± 1.9; f-ratio = 9.51, *P* < 0.01). Furthermore, this gene was expressed at significantly higher levels in LsoB-infected plants compared to LsoA-infected plants (t-value = 1.87, *P* = 0.05). Similarly, Solyc11g069940.1 (or GRXC6) was expressed at similar levels in control (1.1 ± 0.7) and Lso-free infested plants (1.3 ± 0.4), while being significantly under expressed in LsoB- (0.1 ± 0.1) and LsoA-infected plants (0.0 ± 1.1; f-ratio = 17.01, *P* < 0.01).

### 4- growth experiments

**Fig. 5:**
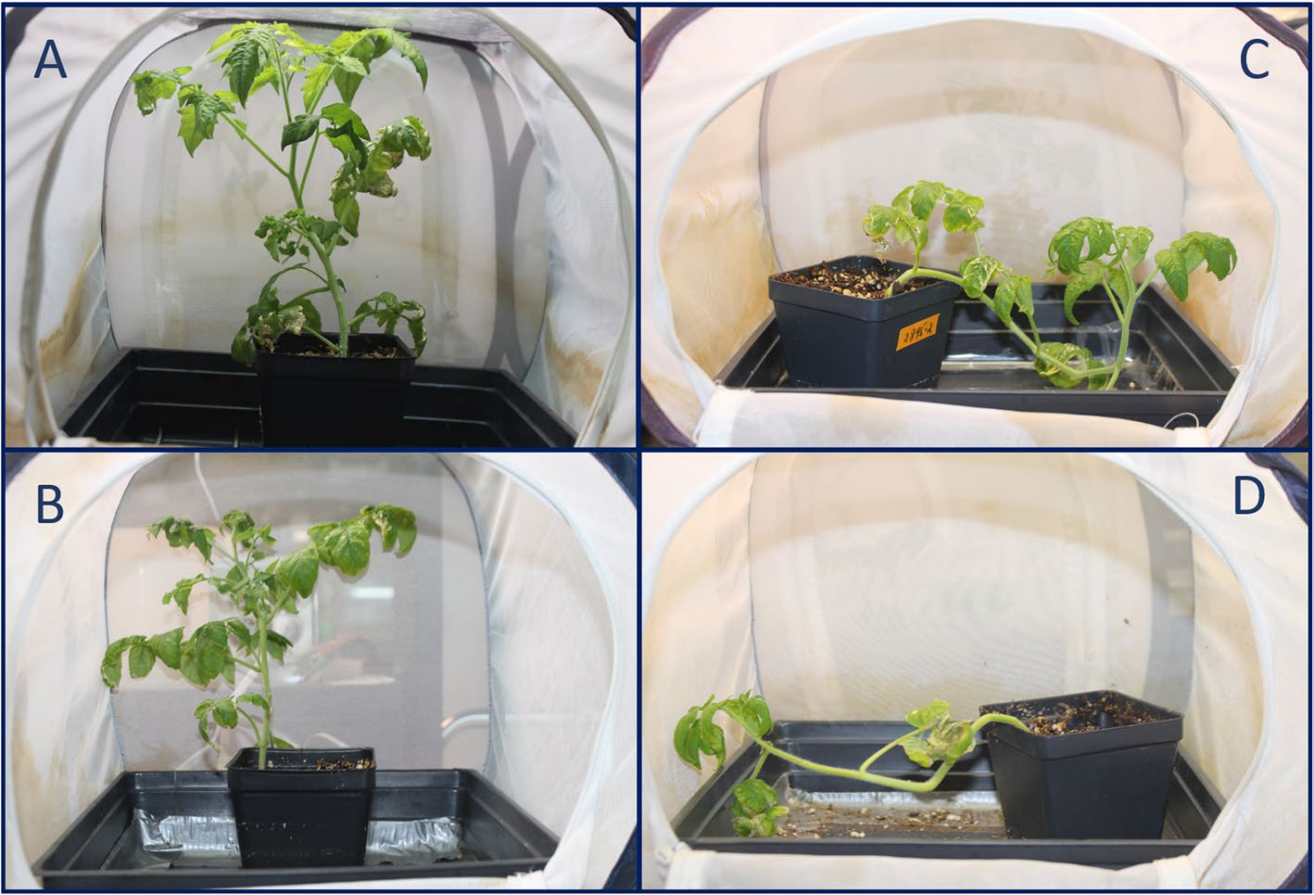
Photographs depicting typical growth of tomato plants among psyllid treatment groups: Control plants (**A**) grew normally and displayed normal negative geotropism, plants infested with Lso-free psyllids (**B**) were slightly stunted but displayed normal negative geotropism, LsoA-infected plants (**C**) were significantly stunted and failed to display negative geotropism, and LsoB-infected plants (**D**) were significantly stunted, failed to display negative geotropism, and would occasionally become limp

The experiments tracking tomato plant stem growth showed that, 3 weeks following psyllid infestation, control (8.5 ± 5.4 cm), Lso-free infested (8.3 ± 5.1 cm), and LsoA-infected plants (5.8 ± 3.4 cm) were all significantly longer than LsoB-infected plants (4.1 ± 2.8; f-ratio = 5.79, *p*-value< 0.001, *N* = 24). Although some LsoA-infected plants were stunted, their average lengths were not significantly lower than uninfected plants. In addition, the experiments tracking negative geotropism among Lso treatments showed that, within 24 h, control and Lso-free infested plants recovered from being placed on their side and grew vertically (100% and 96 ± 4%, respectively), while LsoB- and LsoA-infected plants seldom recovered (8 ± 8% and 13 ± 11%, respectively; f-ratio = 104.2, *p*-value< 0.001, *N* = 24). In fact, some LsoB-infected plants would become limp after being placed on their sides (Fig. 5).

## Discussion

Transcriptomic analysis of *S. lycopersicum* leaves showed that 578 genes were differentially expressed in tomato plants 3 weeks after Lso infection, suggesting that Lso infection has consequences for gene expression in tomato plants (Figs. 1 and 2; See Supplementary Table 2 for details). Based on the g:Profiler analysis of *Arabidopsis* homologs (Fig. 2), the genes differentially expressed between Lso-infected and uninfected plants were mostly associated with 1) defense against abiotic and biotic stress, 2) growth and development, 3) plant primary metabolism, 4) signaling and transport, and 5) transcription/translation regulation (Supplementary Tables 2 and 5, 6,7, 8, 9). The following interpretations for the observed expression changes are based on functional characterizations conducted in *A. thaliana*, *Nicotiana tabacum*, and other model organisms. Therefore, these interpretations are putative in nature and may function differently in tomato plants.

Among the genes differentially expressed between Lso-infected and uninfected plants, 139 were homologs of genes associated with plant response to biotic and/or abiotic stress (Supplementary Table 5). Based on previously published characterizations of gene homologs, the expression changes of 127 DEGs (91%) observed in Lso-infected plants were associated with defense against or responsiveness to stressors. For example, lysM domain receptor-like kinase 4 (LYK4; Solyc02g089900.1) is a Lysin motif receptor kinase that functions as a cell surface receptor in chitin elicitor signaling leading to innate immunity against certain fungal and bacterial pathogens [23]. Since expression of LYK4 was significantly up-regulated (DE = 2.42, *P* < 0.01) in Lso-infected tomato plants, both innate immunity and defense against certain pathogens were promoted. Based on the g:Profiler analysis, the expression changes observed in 127 of these genes (91% of stress-related DEGs) would have likely resulted in increased responsiveness to or promoted defense against stressors. These DEGs were predominantly related to defense against pathogens and response to drought, temperature, and salt stress (see Supplementary Table 5 for citations). A subset of these (34 DEGs) were specifically involved in the tomato plant’s defense against pathogen challenge, most of which would have increased the plant’s defense response. Overall, these results support the first hypothesis, which predicted a significant number of DEGs could be identified between uninfected and infected tomato plants and that many of the expression changes observed post-infection would be related to plant defense and stress response. Furthermore, the expression changes observed among these DEGs were consistent with a general broad-spectrum physiological response of a plant to an infection. Also, the results of the current research are consistent with previous research that characterized the global response of tomato plants to Lso, which also demonstrated that several defense-related genes were up-regulated post-infection [19].

Another set of 116 DEGs were homologs of genes involved in growth and/or development (Supplementary Table 6). Based on previously published characterizations of gene homologs, the expression changes in 75 of these genes (65%) observed in Lso-infected plants could be associated with promoted growth and development. For example, protein EXORDIUM (EXO; Solyc04g074420.1) is required for cell expansion in leaves and mediates brassinosteroid-induced leaf and root growth [24]. The expression of EXO was up-regulated in Lso-infected tomato plants (DE = 3.53, *P* < 0.01), meaning expression changes following Lso-infection would have led to leaf and root expansion. Similarly, up-regulated DEGs were related to cell expansion and elongation, reproduction, increased cell wall modification, and pigment production (see Supplementary Table 6 for citations). However, the growth experiments demonstrated that Lso-infected plants were stunted (4.9 ± 3.1 cm) compared to uninfected plants (8.4 ± 5.3 cm) (Fig. 5), suggesting the promotion of growth/development-related genes in Lso-infected. This result was seemingly contradictory, yet the simultaneous expression changes in several other genes would have resulted in impaired growth/development following Lso infection. Examples are the down-regulated Solyc08g036640.3 (TIFY5A-like), Solyc07g042170.3 (protein TIFY10b-like), and Solyc07g042170.3 (TIFY10b-like) which would have resulted in repressed jasmonate-related defense responses. Therefore, it is likely that 1) infected tomato plants attempted to compensate for losses to growth/development incurred by Lso infection [25, 26] and 2) Lso-infected plants undergo some expression trade-offs favoring optimization of stress response over growth/development [27].

Another set of 98 DEGs were homologs of genes involved in plant primary metabolism (Supplementary Table 7). The expression changes in 78 of these genes (80%) observed in Lso-infected plants were associated with primary metabolic processes. For example, arogenate dehydratase/prephenate dehydratase 6, chloroplastic-like (ADT6; Solyc06g074530.1) converts the prephenate produced from the shikimate-chorismate pathway into phenylalanine. Since the expression of ADT6 was up-regulated in Lso-infected tomato plants (DE = 3.07, *P* < 0.01), production of phenylalanine-derived metabolites would have been promoted. These DEGs were related to photosynthesis, protein turnover, fatty acid and sugar metabolism, and biosynthesis (see Supplementary Table 7 for citations). These results suggested tomato plants underwent primary metabolic changes to meet the energetic needs of their defense response to Lso infection [28, 29]. This conclusion is supported by the fact that Lso infection sites are sugar sinks, leading to over-accumulation of starch in plant stems (at least in potato plants) [30]. Furthermore, previous research determined that Lso-infected tomato plants experienced down-regulation in photosynthetic processes [19]. It is likely that photosynthesis is impaired in Lso-infected tomato plants while directing resources into defense processes [23, 24]. Furthermore, tomato plants may have adapted a strategy for prioritizing seed production in the face of terminal infection to mitigate the fitness costs of infection, [25] but were unsuccessful due to the rapid mortality induced by LsoB infection.

Another set of 84 DEGs were homologs of genes involved in plant signaling and transport (Supplementary Table 8). The expression changes in 45 of these genes related to signaling and transport (54%) would have resulted in impaired signaling transduction or transport. For example, ammonium transporter 1 member 3 (AMT1–3; Solyc03g045070.1) is a transporter involved in ammonium uptake from the soil, meaning its down-regulation in Lso-infected tomato plants (DE = − 2.44; *P* = 0.015) would have resulted in impaired ammonium uptake and transport [31]. These DEGs were related to chloroplastic import, metal ion signaling and transport, ABA signaling and transport, and PAMP-induced signaling (See Supplementary Table 8 for citations). Like the DEGs related to plant primary metabolism, these results suggested tomato plants changed their signaling and transport pathways to meet the energetic needs of defense. Since wilting is one of the major symptoms of Lso infection, it is likely that tomato plants are compensating for systemic water deficit. Furthermore, the impaired response to auxin predicted by certain DEGs (e.g., Solyc05g012030.1, Solyc08g079230.1, and Solyc07g014620.1) was supported by the growth experiments, which showed that Lso infection impaired tomato plant geotropism (Fig. 5).

Another set of 44 DEGs were homologs of genes involved in transcription or translation regulation (Supplementary Table 9). The expression changes in 17 of these genes (39%) would have modified regulation of transcription or translation. For example, multiple isoforms of histone H3.2 (HTR2; Solyc07g062700.3) are core components of the nucleosome, meaning their down-regulation in Lso-infected tomato plants (DE = 2.36, *P* < 0.01) would have resulted in increased transcription regulation and DNA repair [32]. These DEGs were related to housekeeping transcription regulation, response to auxin, and the homeostasis of certain compounds or ions (See Supplementary Table 9 for citations). Consistent with these results, previous research determined that several transcription factors similar to the ones identified in the current study (e.g., Ethylene responsive transcription factors such as and WRKY transcription factors such as) were likewise up-regulated in Lso-infected tomato plants [19]. These results suggested an overall pattern of change among regulatory genes. Considering the putative functions of the other DEGs identified in the transcriptomic research, it is likely that most of the differentially expressed regulatory genes are involved in stress response, growth/development, or primary metabolism.

In addition to the genes differentially expressed between infected and uninfected tomato plants, the transcriptomic analysis revealed 451 DEGs when comparing tomato plants infected with different Lso haplotypes (Supplementary Tables 10 and 12, 13, 14, 15). Based on the g:Profiler analysis of *Arabidopsis* homologs (Fig. 3), the genes differentially expressed in response to Lso haplotypes were mostly associated with 1) growth and/or development, 2) response to biotic/abiotic stress, 3) signaling and/or transport, 4) carbohydrate metabolism, and 5) photosynthesis.

Among the genes differentially expressed between LsoB- and LsoA-infected tomato plants, 89 were homologs of genes associated with growth and/or development (Supplementary Table 12). Based on previously published characterizations of gene homologs, the expression changes in 68 of these genes (76%) would have resulted in impaired growth and/or development in LsoB-infected plants relative to LsoA-infected plants. For example, expansin-A1 (EXPA1; Solyc05g007830.3) is involved in the loosening and extension of plant cell walls, meaning its down-regulation in LsoB-infected plants (DE = − 2.19; *P* < 0.05) would have resulted in impaired plant cell wall extension and, therefore, impaired growth (See Supplementary Table 12 for citations). These results suggested that LsoB infection had a greater negative impact on tomato plant growth/development compared to LsoA infection. These results were consistent with the growth experiments which demonstrated that LsoB-infected plants (4.1 ± 2.8 cm) were significantly shorter than LsoA-infected plants (5.8 ± 3.4 cm).

Another set of 61 DEGs were homologs of genes associated with plant response to biotic and/or abiotic stress (Supplementary Table 13). The expression changes in 32 of these genes (52%) would have resulted in impaired responsiveness to stressors in LsoB-infected plants relative to LsoA-infected plants. For example, aspartyl protease AED3 (AED3; Solyc01g096450.3) is involved in the regulation of systemic acquired resistance and programmed cell death, meaning its up-regulation in LsoB-infected plants (DE = 1.71; *P* < 0.05) would have resulted in impaired defense against pathogens (See Supplementary Table 13 for citations). These results suggested that LsoB is a more virulent pathogen compared to LsoA. These results are consistent with previous research indicating that LsoB infection is associated with relatively more severe disease symptoms and higher mortality when compared to haplotype A infection; this has been documented in both tomato [18] and potato plants [16, 17]. Overall, these results supported the second hypothesis, which predicted a significant number of DEGs could be identified between tomato plants infected with different Lso haplotypes and that many of the expression changes observed post-infection would be related to plant defense and stress response. The DEGs identified here should be further studied to better understand the molecular basis of Liberibacter pathogenicity. These genes are good candidates to reduce plant susceptibility to Lso.

Another set of 48 DEGs were homologs of genes associated with signaling and/or transport (Supplementary Table 14). The expression changes in 29 of these genes (60%) would have resulted in impaired signaling and/or transport in LsoB-infected plants relative to LsoA-infected plants. For example, protein GAST1 precursor (GAST1; Solyc02g089350.3) is involved in root-specific abscisic acid-signaling regulation, meaning its up-regulation in LsoB-infected plants (DE = 2.26; *P* < 0.05) would have resulted in impaired abscisic acid signal transduction throughout the roots (See Supplementary Table 14 for citations). In addition, 12 of these genes were related to auxin signaling. As with infected plants in general, these results suggested that LsoB-infected plants are less auxin-responsive than LsoA-infected plants. While an ANOVA showed that both LsoB- (8 ± 8%) and LsoA-infected plants (13 ± 11%) generally failed to recover after being tipped over for 24 h (f-ratio = 5.79, *p*-value< 0.01, *N* = 24), LsoB-infected plants would often go completely limp (Fig. 5). This is reflected in a host-hoc Tukey’s HSD (Honest Significant Difference) test, which showed that recovery rate was significantly different only when comparing LsoB-infected plants to either controls (Q = 5.00, *p*-value< 0.01) or Lso-free plants (Q = 4.79, *p*-value< 0.01).

Another set of 34 DEGs were homologs of genes associated with carbohydrate metabolism, along with a set of 16 DEGs which were homologs of genes associated with photosynthesis (Supplementary Table 15). The expression changes in 26 of the carbohydrate metabolism-related genes (76%) would have resulted in impaired carbohydrate metabolism in LsoB-infected plants relative to LsoA-infected plants, while every change among the photosynthesis-related genes would have resulted in impaired photosynthesis in LsoB-infected plants. For example, photosystem II protein D1 (psbA; Solyc12g039030.1) is a photosynthetic electron transporter in photosystem II, meaning its up-regulation in LsoB-infected plants (DE = 1.97; *P* < 0.05) would have resulted in impaired photosynthesis and, thus, impaired sugar synthesis (See Supplementary Table 15 for citations). These results suggested that, in addition to causing more severe disease symptoms, LsoB causes a more severe loss of plant productivity.

Although 344 genes differentially expressed between Lso-infected and uninfected plants were homologs of genes for which published characterizations were available, 234 DEGs (41%) lacked any supporting information. Therefore, a large portion of the transcriptomic response to Lso remained uncharacterized. Of these DEGs, 152 (65%) were up-regulated in Lso-infected plants, consistent with the overall pattern of gene expression observed among characterized genes. It would seem likely that the uncharacterized genes are also related to plant responses to abiotic/biotic stress, growth/development, plant primary metabolism, signaling and transport, or transcription and translation. In contrast, published characterizations existed for a larger proportion of the genes differentially expressed between tomato plants infected with different Lso-haplotypes, and *A. thaliana* GO functional categories were available for 210 of these DEGs (68%).

In conclusion, the results of this manuscript are the first to report the effects of infection by different Lso haplotypes on tomato plant gene expression. The transcriptomic analysis and growth experiments demonstrated that Lso-infected tomato plants underwent expression changes that likely impaired responsiveness to abiotic and biotic stressors, impaired growth/development, impaired plant primary metabolism, impaired transport and signaling transduction, and impaired transcription/translation. Furthermore, the transcriptomic analysis and growth experiments demonstrated that LsoB-infected plants, relative to LsoA-infected plants, experience more severe stunting, have a more impaired response to stressors, have poorer transport and signaling transduction, and have impaired carbohydrate synthesis and photosynthesis. The DEGs that likely resulted in improved defense against pathogen challenge may constitute the genes directly involved in the tomato’s long-term response to Lso challenge. This hypothesis is supported by the tomato plant geotropism experiments which showed that Lso-infected plants are likely unresponsive to auxin signaling, which is an outcome predicted by the transcriptomic results which suggested auxin-responsive genes are suppressed in Lso-infected tomatoes. The results of the current research parallel the transcriptomic analysis characterizing the genes differentially expressed in response to psyllid infestation [19, 22], which also demonstrated long-term consequences for plant expression following biotic challenge. Interestingly, some of the significant differences between uninfected and infected plants were the general promotion of jasmonic acid-related pathways and cell wall modification (Supplementary Tables 5 and 6), and these same processes were affected even more in LsoB-infected plants (Supplementary Tables 12 and 13). These results parallel previous research which suggested phloem regeneration contributed to ‘*Candidatus* Liberibacter asiaticus’ tolerance in *Citrus* L. [33]. Future research should investigate improvements to jasmonic-acid related pathways, cell wall modification, and phloem regeneration in tomato plants for the purpose of generating Lso-tolerant varieties. In addition, future research should validate the physiological responses to Lso infection predicted by the transcriptomic analysis. Specifically, several DEGs were shown to be involved in the tomato plant’s defense and growth responses to Lso infection, giving researchers several targets for promoting Lso resistance and/or survival under Lso challenge.

## Materials and methods

### Plant material

Tomato plants, *Solanum lycopersicum* L. ‘Moneymaker’ (Victory Seed Company; Molalla, OR), were grown from seed in Metro-Mix 900 (Sun Gro Horticulture, Agawam, MA) soil and individually transplanted to 10 × 10 cm square pots after 4 weeks. Plants were watered every other day and fertilized weekly according to the manufacturer’s recommendation (Miracle-Gro® Water Soluble Tomato Plant Food; 18–18-21 NPK). All experiments were conducted at the same photoperiod (16: 8) and temperature (22 ± 2 °C) used to rear psyllids.

### Insect colonies

Psyllids were maintained on tomato plants under a 16:8-h (Light: Dark) photoperiod at room temperature (22 ± 2 °C) inside insect terrarium cages (Bioquip Inc., Compton, CA). Lso-free and LsoA- or LsoB-infected colonies were maintained in separate rooms to reduce the risk of cross contamination [34]. The presence or absence of Lso, along with Lso haplotype identities, were verified among psyllid colonies each month using the diagnostic PCR method previously described [35]. Briefly, DNA from individual psyllids representing each colony was isolated using the 10% CTAB method. This DNA was subjected to PCR amplification for ‘*Candidatus* Liberibacter solanacearum’ 16S rDNA and Lso SSR using the Lso SSR-1 primers [36].

### Psyllid infestation, Lso infection, and sample collection

Psyllid infestations were initiated when plants were 6 weeks old. One leaflet below the apical meristem was caged using a small, white organza bag (amazon.com). Each bag contained an Eppendorf tube with no psyllids (control), with three psyllids from the Lso-free colony (Lso-free), three psyllids from the LsoB-infected psyllids (LsoB), or three psyllids from the LsoA-infected psyllids (LsoA). Only males were used to infest plants to avoid the potentially confounding effect of oviposition on tomato plant gene expression. Seven days after infestation, caged tomato leaves were removed using a bleach-sterilized razor blade. Three weeks later (i.e., when haplotype titers were similar and symptoms had just begun to develop), the top-most, fully developed leaf was similarly removed from each plant and immediately flash-frozen in liquid nitrogen for downstream transcriptome analysis. Samples were transferred to Eppendorf tubes and kept submerged under liquid nitrogen while ground with plastic, RNase-free pestles.

### RNA purification, sequencing, and bioinformatic analysis

Total RNA extraction was performed on leaf tissue harvested 3 weeks after psyllid infestation using the Plant RNeasy Mini Kit (Qiagen, Valencia, CA) following the manufacturer’s protocol. RNA samples were treated with RNase-Free DNase (Qiagen). Any remaining DNA was removed using the TURBO DNA-free Kit (Life Technologies, Carlsbad, CA). All remaining RNA was stored at − 80 °C for downstream quantitative reverse transcription PCR (RT-qPCR) validation. The isolated RNA was submitted to the Texas A&M Genomics and Bioinformatic Service for quality control analysis, library preparation, and sequencing. Three biological replicates were sequenced per treatment (i.e., control, Lso-free, LsoB-infected, and LsoA-infected, twelve samples total).

For transcriptomic sequencing, cDNA libraries were developed using the TruSeq RNA Library Prep Kit v2 (Illumina®; San Diego, CA) following the manufacturer’s protocol, generating 2 Х 150 bp read lengths. Libraries were multiplexed and sequenced on the Illumina PE HiSeq 2500 v4 platform. Sequence cluster identification, quality prefiltering, base calling, and uncertainty assessment were done in real time using Illumina’s HCS 2.2.38 and RTA 1.18.61 software with default parameter settings. Library preparation, sequencing, and read processing were performed by the Texas A&M Genomics and Bioinformatic Service. The processed sequences were uploaded to the CyVerse Discovery Environment computational infrastructure [37] where bioinformatic analysis was performed using the HISAT2-StringTie-Ballgown RNA-Seq workflow [38]. Libraries reads were mapped to the *S. lycopersicum* genome (vSL3.0) using HISAT2. StringTie assembled hits to known transcripts based on the vITAG3.2 annotation and made non-redundant with StringTie-Merge. All treatments were compared to each other and DEGs were identified using Ballgown, an R package which provides functions to organize, visualize, and analyze the expression measurements for transcriptome assemblies [21]. Genes were considered differentially expressed when comparative *p*-values were below 0.05. DEG gene names were searched against the tomato genome database [39] as well as the PhytoMine search engine in Phytozome [40]. DEGs were assigned putative functions based on their homology with other plant genes with known function published in Ensembl Plants (version SL2.50) and the UniProt Knowledgebase. *Arabidopsis thaliana* homologs of DEGs were uploaded to the NCBI Gene Expression Omnibus (GEO:GSE196951) functional genomics data repository to visualize overrepresentation among molecular pathways using the g:Profiler functional profiler. These same homologs were uploaded to KEGG (Kyoto Encyclopedia of Genes and Genomes) for search analysis among known molecular pathways.

### Transcriptome validation by RT-qPCR

To verify the results of the transcriptomic analysis, RT-qPCR analyses were performed on five genes differentially expressed between uninfected and infected tomato plants: two genes putatively up-regulated in LsoA- or LsoB-infected plants, relative to control and Lso-Free plants, (Solyc06g076020.3 and Solyc12g009220.2) and three down-regulated genes (Solyc10g047090.2, Solyc11g069940.1, and Solyc12g035550.1). These genes were selected because they were expected to be expressed in all treatments at detectable levels while displaying distinct expression patterns between treatments. RT-qPCR experiments were conducted using RNA from twelve independently grown tomato plants (three per treatment), which were obtained by repeating the infestation methods described above (three plants per treatment). An aliquot of 500 ng RNA was taken from each sample to develop cDNA libraries using the Verso cDNA Kit (Thermo Fisher Scientific, Waltham, MA), following the manufacturer’s manual. The cDNA libraries were diluted to 1:5 prior to RT-qPCR. Each reaction consisted of 1.0 μL cDNA, 5.0 μL SensiFAST SYBR Hi-ROX mix (Bioline, Memphis, TN), 0.4 μL of each primer (400 nM), and 3.6 μL of molecular grade water. Primers were designed using Primer3 [41], which targeted exons within a DEG, had an optimal annealing temperature of 60.0–62.0 °C, and generated 150 bp amplicons (Supplementary Table 1). RT-qPCR was performed in an Applied Biosystem QuantStudio 6 Flex system using the following parameters: 2 min at 95 °C, followed by 40 cycles of 5 s at 95 °C and 30 s at 60 °C. The melting curve for each reaction was generated to assure amplicon specificity. All RT-qPCR reactions were performed in triplicate. Relative expression levels for each gene were analyzed using the 2^-ΔΔCT^ method [42] with glyceraldehyde 3-phosphate dehydrogenase (GADPH) as a reference gene [19]. Since expression levels did not assume normality, they were analyzed using the Mann-Whitney U ranked test in JMP® Version 13 (SAS Institute Inc., Cary, NC, 1989–2018).

### Plant growth experiments

To test the potential role of auxin signaling in Lso symptomology, tomato plants were grown and treated using the same methods described above and 24 individual plants were assigned to each psyllid treatment group. To minimize handling stress, plant growth was tracked using pictures taken 3 weeks after infestation to compare the total stem length of psyllid-infested plants to uninfested plants. Each picture included a 52 cm-long tray that served as a size standard. The total length (in pixels) of a tomato plant main stem was measured from the soil to the tip of the apical meristem using ImageJ1.X [43] and converted to centimeters using the length standard. This no-contact method of measurement was chosen to minimize plant wounding. Stem lengths were analyzed using a one-way ANOVA and a post-hoc Tukey’s HSD (Honest Significant Difference) test in JMP. After stem lengths were measured, plants were tipped onto their sides and left to grow for another 24 h. Plants were then set back upright to test their ability to establish negative geotropism in this timeframe. Plants whose apical meristem pointed upwards were considered to have ‘recovered’. Recovery rates were compared between treatments using a one-way ANOVA and a post-hoc Tukey’s HSD test.

## Supplementary Information

**Additional file 1: Table S1.** HISAT2 alignment summary of uninfected and Lso-infected tomato plant transcriptomes to the *S. lycopersicum* vSL3.0 genome.

**Additional file 2: Table S2.** Complete table of all 578 DEGs between uninfected and Lso-infected tomato plants. Columns depict (in order) the tomato transcript ID, p- and q-values comparing the fragments per kilobase of transcript per million reads (fpkm) between uninfected to infected samples, the differential expression (DE) value comparing uninfected to infected samples, the fpkm for each sample, the NCBI protein name for the identified transcript, its gene ID, the *A. thaliana* homolog used in the g:Profiler analysis, the UniProt description, the predicted consequences for differential expression after infection (based on homologs of published genes), and supporting literature.

**Additional file 3: Table S3.** KEGG Pathway search analysis of tomato plant DEGs associated with Lso infection. Among the 238-infection associated DEGs also involved in a KEGG Pathway, 54 were involved in metabolic pathways, 44 were involved in the biosynthesis of (various) secondary metabolites, 9 were involved in phenylpropanoid biosynthesis, 9 were involved in the biosynthesis of amino acids, 7 were involved in plant hormone signal transduction, 5 were involved in the MAPK signaling pathway, 5 were involved in carbon metabolism, 4 were involved in flavonoid biosynthesis, 4 were involved in alpha-Linolenic acid metabolism, 4 were involved in stilbenoid, diarylheptanoid and gingerol biosynthesis, and 4 were involved in Arginine and proline metabolism. Several more DEGs were involved in other pathways (e.g., circadian rhythm photosynthesis), but with less representation.

**Additional file 4: Table S4.** Results from the g:Profiler analysis. The first column describes the GO information source (MF for molecular function, BP for ‘biological process’, and CC for ‘cellular component’ and KEGG for ‘Kyoto Encyclopedia of Genes and Genomes’) for each circle. The second column describes the term name associated with each circle. The third column describes the associated GO ID for the term. The fourth column shows the adjusted *p*-value for each term. Columns five through eight describe the term, query, intersection, and effective domain sizes.

**Additional file 5: Table S5.** The 139 tomato plant DEGs associated with plant defense response to abiotic and biotic stress under Lso infection. DEGs were sorted by differential expression (DE) values comparing uninfected (control and Lso-free) to infected (LsoA- and LsoB-infected) samples (p-value < 0.05). NCBI Blast searches were used to identify Gene IDs and protein products in tomatoes as well as their homologs in other species. Specifically, the expression changes in 127 of these genes (91%, *in italics*) would have resulted in increased responsiveness to or promoted defense against stressors in infected plants. These DEGs were predominantly related to defense against pathogens and response to drought, temperature, and salt stress.

**Additional file 6: Table S6.** The 116 tomato plant DEGs associated with plant growth and development under Lso infection. DEGs were sorted by differential expression (DE) values comparing uninfected to infected samples (*p*-value < 0.05). NCBI Blast searches were used to identify Gene IDs and protein products in tomatoes as well as their homologs in other species. Specifically, the expression changes in 75 of these genes (65%, *in italics*) would have modified cell growth and development in infected plants. These DEGs were predominantly related to cell expansion and elongation, reproduction, increased cell wall modification, and pigment production.

**Additional file 7: Table S7.** The 98 tomato plant DEGs associated with plant primary metabolism under Lso infection. DEGs were sorted by differential expression (DE) values comparing uninfected to infected samples (p-value < 0.05). NCBI Blast searches were used to identify Gene IDs and protein products in tomatoes as well as their homologs in other species. Specifically, the expression changes in 78 of these genes (80%, *in italics*) would have promoted primary metabolic processes in infected plants. These DEGs were related to photosynthesis, protein turnover, fatty acid and sugar metabolism, and biosynthesis.

**Additional file 8: Table S8.** The 84 tomato plant DEGs associated with plant signaling and transport under Lso infection. DEGs were sorted by differential expression (DE) values comparing uninfected to infected samples (*p*-value < 0.05). NCBI Blast searches were used to identify Gene IDs and protein products in tomatoes as well as their homologs in other species. Specifically, the expression changes in 45 of these genes (54%, *in italics*) would have resulted in impaired signaling transduction or transport in infected plants. These DEGs were related to chloroplastic import, metal ion signaling and transport, abscisic acid signaling and transport, auxin-responsive signaling, and PAMP-induced signaling.

**Additional file 9: Table S9.** The 44 tomato plant DEGs associated with transcription or translation regulation under Lso infection. DEGs were sorted by differential expression (DE) values comparing uninfected to infected samples (p-value< 0.05). NCBI Blast searches were used to identify Gene IDs and protein products in tomatoes as well as their homologs in other species. Specifically, the expression changes in 17 of these genes (39%, *in italics*) would have modified regulation of transcription or translation in infected plants. These DEGs were related to transcription regulation, response to auxin, and the homeostasis of certain compounds and ion.

**Additional file 10: Table S10.** Complete table of all 451 DEGs between LsoA-infected and LsoB-infected tomato plants. Columns depict (in order) the tomato transcript ID, p- and q-values comparing the fragments per kilobase of transcript per million reads (fpkm) between uninfected to infected samples, the differential expression (DE) comparing uninfected to infected samples, the fpkm for each sample, the NCBI protein name for the identified transcript, its gene ID, the *A. thaliana* homolog used in the g:Profiler analysis, the UniProt description, the predicted consequences for differential expression after infection (based on homologs of published genes), and supporting literature.

**Additional file 11: Table S11.** KEGG Pathway search analysis of tomato plant DEGs associated with infection by different Lso haplotypes. Among the 166-haplotype associated DEGs also involved in a KEGG Pathway, 39 were involved in metabolic pathways, 22 were involved in the biosynthesis of secondary metabolites, 5 were involved in plant hormone signal transduction, 5 were involved in photosynthesis, 5 were involved in circadian rhythm, and 5 were involved in phenylpropanoid biosynthesis. Several more DEGs were involved in other pathways (e.g., photosynthesis), but with less representation.

**Additional file 12: Table S12.** The 89 tomato plant DEGs associated with plant growth and development under infection by different Lso haplotypes. DEGs were sorted by differential expression (DE) values comparing uninfected to infected samples (*p*-value < 0.05). NCBI Blast searches were used to identify Gene IDs and protein products in tomatoes as well as their homologs in other species. Specifically, the expression changes in 68 of these genes (76%, *in italics*) would have resulted in impaired cell growth and development in LsoB-infected plants relative to LsoA-infected plants. These DEGs were predominantly related to cell expansion and elongation and increased cell wall modification.

**Additional file 13: Table S13.** The 61 tomato plant DEGs associated with plant defense response to abiotic and biotic stress under infection by different Lso haplotypes. DEGs were sorted by differential expression (DE) values comparing uninfected to infected samples (*p*-value < 0.05). NCBI Blast searches were used to identify Gene IDs and protein products in tomatoes as well as their homologs in other species. Specifically, the expression changes in 32 of these genes (52%, *in italics*) would have resulted in impaired responsiveness to or promoted defense against stressors in LsoB-infected plants relative to LsoA-infected plants. These DEGs were predominantly related to defense against pathogens and response to drought, temperature, and salt stress.

**Additional file 14: Table S14.** The 48 tomato plant DEGs associated with plant signaling and transport under infection by different Lso haplotypes. DEGs were sorted by differential expression (DE) values comparing uninfected to infected samples (*p*-value < 0.05). NCBI Blast searches were used to identify Gene IDs and protein products in tomatoes as well as their homologs in other species. Specifically, the expression changes in 29 of these genes (60%, *in italics*) would have resulted in impaired signaling transduction or transport in LsoB-infected plants relative to LsoA-infected plants. These DEGs were predominately related to ion transport, abscisic signaling and transport, and auxin-responsive signaling.

**Additional file 15: Table S15.** The 50 tomato plant DEGs associated with either carbohydrate metabolism or photosynthesis under infection by different Lso haplotypes. DEGs were sorted by differential expression (DE) values comparing uninfected to infected samples (p-value < 0.05). NCBI Blast searches were used to identify Gene IDs and protein products in tomatoes as well as their homologs in other species. Specifically, the expression changes in 26 of the 34-carbohydrate related genes (76%, *in italics*) would have resulted in impaired carbohydrate metabolism in LsoB-infected plants relative to LsoA-infected plants, while every change among the photosynthesis-related genes (100%, *in italics*) would have resulted in impaired photosynthesis in LsoB-infected plants. These DEGs were predominantly related to cell wall-related carbohydrates, sugar biosynthesis, and essential components of photosynthesis.

**Additional file 16: Figure S1.** Distribution of log2 fragments per kilobase of transcript per million reads (fpkm) among among uninfested (Control#), Lso-free psyllid infested (LsoFree#), LsoB-infected (LsoB#), and LsoA-infected (LsoA#) tomato plant sample libraries. These values were not signficantly different between treatments.

**Additional file 17: Figure S2.** Distribution of transcript lengths across all samples and treatments. The X-axis depicts transcript lengths (bp), while the Y-axis depicts the relative frequency of those transcripts.

**Additional file 18: Figure S3.** Per-gene comparison of log-transformed fragments per kilobase of transcript per million read (fpkm) values between uninfected (X-axis) and Lso-infected (Y-axis) tomato plants. Pink dots depict genes with significantly different fpkm values between uninfected and infected treatments. Black dots depict genes that do not have significantly different fpkm values between uninfected and infected plants.

**Additional file 19: Figure S4.** Distribution (in frequency) of differentially expressed genes (DEGs) across all libraries. The X-axis depicts the log-transformed fold change value of sequenced DEGs (relative to controls), while the Y-axis depicts the relative frequency of those DEGs.

**Additional file 20: Figure S5.** Per-gene comparison of log-transformed fragments per kilobase of transcript per million read (fpkm) values between LsoB- and LsoA-infected tomato plants. Pink dots depict genes with significantly different fpkm values between treatments infected with Lso haplotype B (X-axis) and treatments infected with Lso haplotype A (Y-axis). Black dots depict genes that do not have significantly different fpkm values.

## Data Availability

Raw sequence data, processed data, and metadata were made available on the Gene Expression Omnibus (GEO: GSE196951) functional genomics repository under the ‘kharrison18’ directory (to be published when manuscript is accepted; NCBI tracking system #X). Other data, including plant pictures and RT-qPCR results, can be obtained from the corresponding author, Dr. Cecilia Tamborindeguy, upon request.

## Abbreviations

DEG(s): Differentially expressed gene(s)
fpkm: Fragments per kilobase per million reads
GEO: Gene Expression Omnibus
Lso: ‘*Candidatus* Liberibacter solanacearum’
NIFA: National Institute of Food and Agriculture
PCA: Principal component analysis
RT-qPCR: Quantitative reverse transcription PCR
