# Supplementary material for: Effects of ‘Candidatus Liberibacter solanacearum’ haplotypes A and B on tomato gene expression and geotropism
Source: BMC Plant Biol. 2022 Mar 30;22:156. doi: 10.1186/s12870-022-03505-z (PMC8966271; doi:10.1186/s12870-022-03505-z)
Supplement: Supplementary file 1 — Additional file 1: Table S1. HISAT2 alignment summary of uninfected and Lso-infected tomato plant transcriptomes to the S. lycopersicum vSL3.0 genome. [file 12870_2022_3505_MOESM1_ESM.pdf]

Sample	Total Reads	Overall Alignment Rate %
Control1	17358465	96.31
Control2	16461183	96.45
Control3	18455303	95.99
LsoFree1	18557929	95.79
LsoFree2	17347190	96.64
LsoFree3	18381570	96.10
LsoB1	27747327	96.19
LsoB2	27794036	95.74
LsoB3	35649880	94.81
LsoA1	33426003	94.18
LsoA2	32938709	94.39
LsoA3	33593288	94.28
