# Supplementary material for: Effects of ‘Candidatus Liberibacter solanacearum’ haplotypes A and B on tomato gene expression and geotropism
Source: BMC Plant Biol. 2022 Mar 30;22:156. doi: 10.1186/s12870-022-03505-z (PMC8966271; doi:10.1186/s12870-022-03505-z)
Supplement: Supplementary file 2 — Additional file 2: Table S2. Complete table of all 578 DEGs between uninfected and Lso-infected tomato plants. Columns depict (in order) the tomato transcript ID, p- and q-values comparing the fragments per kilobase of transcript per million reads (fpkm) between uninfected to infected samples, the differential expression (DE) value comparing uninfected to infected samples, the fpkm for each sample, the NCBI protein name for the identified transcript, its gene ID, the A. thaliana homolog used in the g:Profiler analysis, the UniProt description, the predicted consequences for differential expression after infection (based on homologs of published genes), and supporting literature. [file 12870_2022_3505_MOESM2_ESM.pdf]

TomatoID	p-value	q-value	DE in Infected	Control1 FPKM	Control2 FPKM	Control3 FPKM	LsoFree1 FPKM	LsoFree2 FPKM	LsoFree3 FPKM	LsoB1 FPKM	LsoB2 FPKM	LsoB3 FPKM	LsoA1 FPKM	LsoA2 FPKM	LsoA3 FPKM
Solyc03g098790.3	0.001	0.016	11.71	0	0.09	0	0.09	0	0.47	459.85	225.86	16.23	5458.74	6056.46	8923.11
Solyc00g068980.2	<0.001	<0.001	9.43	0	0	0	0	0	0	197.45	347.84	520.03	1201.28	1236.01	811.74
Solyc12g010020.2	<0.001	0.005	9.14	0	0.09	0	0.65	0	1.19	55.29	84.19	67.56	450.61	426.8	1927.93
Solyc00g187050.3	<0.001	0.005	8.79	0	0.29	0	0	0	0	37.32	46.94	40.74	276.48	270.57	1350.07
Solyc12g010025.1	<0.001	0.006	8.79	0	0.01	0	0.63	0	1.01	41.67	55.04	46.89	313.69	315.27	1540.32
Solyc12g010030.2	<0.001	0.007	8.64	0	0.35	0	0	0	0	33.96	25.74	27.32	214.93	233.33	1277.21
Solyc09g089530.3	0.005	0.041	8.43	0	0	0	0	0	0	13.07	8.56	1.91	265.21	713.65	1149.07
Solyc09g084470.3	0.002	0.026	8.38	2.63	27.84	1.67	27.97	6.96	18.4	1802.24	515.29	56.9	4462.38	4631.07	6792.8
Solyc09g084480.3	0.002	0.026	8.38	0	0	0	2.01	0	1.59	105.95	73.74	26.13	1198.31	2306.86	3633.38
Solyc09g084490.3	0.002	0.026	8.38	0	2.62	0	0.44	0	0.56	338.38	76.4	13.55	549.22	494.58	1115.14
Solyc08g074630.2	0.003	0.031	8.19	0	1.56	0.22	1.17	1.63	0.95	129.77	51.12	8.63	953.78	1262.58	1553.81
Solyc01g095140.3	<0.001	0.013	7.9	0.1	0	0.3	0	0	0.32	10.15	409.04	378.43	152.11	174.51	604.78

TomatoID	p-value	q-value	DE in Infected	Control1 FPKM	Control2 FPKM	Control3 FPKM	LsoFree1 FPKM	LsoFree2 FPKM	LsoFree3 FPKM	LsoB1 FPKM	LsoB2 FPKM	LsoB3 FPKM	LsoA1 FPKM	LsoA2 FPKM	LsoA3 FPKM
Solyc03g098780.2	0.007	0.046	7.43	1.37	72.34	6.14	39.23	5.35	14.65	780.4	350.79	44.99	3007.29	3292.29	4952.38
Solyc09g083440.3	0.003	0.032	7.35	0.98	16.06	2.92	68.97	1.78	28.3	446.15	351.37	99.93	1878.59	1481.5	2841.52
Solyc06g062370.3	0.003	0.03	7.18	0.07	1.79	0.1	0	0.38	0	58.1	22.45	2.09	230.5	208.25	416.74
Solyc09g084450.3	0.002	0.026	6.67	0.77	2.09	0.6	0	0.52	0.25	23.32	6.8	3.58	108.89	147.88	347.91
Solyc09g089520.3	0.006	0.043	6.6	0	0	0.2	0	0	0	3.27	4.5	0.63	53.19	83.04	365.43
Solyc09g008670.3	0.003	0.029	6.58	0.2	1.46	0.32	0	0.09	0.48	10.63	10.96	1.74	97.06	95.73	320.81
Solyc12g096780.2	0.008	0.05	6.48	0	0.19	0	0	0	0	7.4	7.77	0.49	147	111.53	242.11
Solyc07g007250.3	0.001	0.015	6.31	32.55	49.05	22.87	62.74	40.18	62.86	1790.18	835.11	305.16	4705.42	4958.53	6135.3
Solyc09g089505.1	0.007	0.048	6.29	11.86	165.45	31.76	209.23	17	141.08	1077.31	595.73	143.58	3622.76	4980.13	7736.01
Solyc09g089510.3	0.007	0.048	6.29	0.22	3.44	0.6	8.39	0.64	3.41	7.59	3.16	4.15	26.24	79.19	52.08
Solyc11g027770.1	<0.001	0.001	6.16	0.12	0.03	0.02	0	0	0.04	22.83	38.71	52.67	115.03	120.02	83.33
Solyc07g008570.3	0.001	0.019	6.14	0.43	0.28	0.09	0.45	0.36	0.59	12.94	10.92	4.37	65.28	73.5	253.19

TomatoID	p-value	q-value	DE in Infected	Control1 FPKM	Control2 FPKM	Control3 FPKM	LsoFree1 FPKM	LsoFree2 FPKM	LsoFree3 FPKM	LsoB1 FPKM	LsoB2 FPKM	LsoB3 FPKM	LsoA1 FPKM	LsoA2 FPKM	LsoA3 FPKM
Solyc11g021060.2	0.006	0.044	6.13	0	3.38	0.58	1.8	1.77	0.22	41.43	16.01	4.2	216	143.16	478
Solyc01g091170.3	0.001	0.019	6.1	4.91	4.79	2.77	11.3	2.71	7.54	60.6	74.68	22.42	249.59	337.43	1023.2
Solyc01g017490.1	<0.001	0.004	6.06	0	0	0	0	0.11	0.1	18.07	27.24	48.13	107.91	133.13	83.65
Solyc06g083900.3	0.002	0.022	6.03	0	1.96	0	0	0.24	0.08	17.15	20.46	3.9	125.86	63.53	180.79
Solyc07g064600.3	0.001	0.015	5.98	2	2.77	1.94	8.03	2.13	6.39	41.77	51.87	34.61	222.07	221.93	645.66
Solyc12g010980.2	0.003	0.032	5.65	1.97	14.85	1.96	1.28	2.45	0.31	19.68	57.66	20.82	176.47	120.89	471.31
Solyc11g051200.1	<0.001	0.003	5.6	0	0	0	0	0	0	14.49	22.49	34.84	72.73	78.73	56.16
Solyc11g071480.1	0.008	0.05	5.55	0	0	0	0	0	0	3.25	1.93	0.41	35.23	46.69	128.14
Solyc01g006400.3	0.005	0.039	4.94	55.08	96.46	59.12	75.92	27.95	45.96	354.34	175.51	64.57	1652.69	1468.23	2601.8
Solyc12g009220.2	0.001	0.016	4.88	4.88	1.78	2.92	3.41	3.1	5.37	27.84	337.98	325.22	255.76	195.09	137.06
Solyc01g017050.1	<0.001	0.008	4.87	11.83	22.11	23.27	19.78	8.42	31.33	104.78	159.91	262.44	543.79	644.38	457.2
Solyc11g022590.1	0.004	0.036	4.84	99.62	232.33	235.27	544.02	45.34	624.43	1745.38	1019.87	325.79	4651.74	3942.3	4536.27

TomatoID	p-value	q-value	DE in Infected	Control1 FPKM	Control2 FPKM	Control3 FPKM	LsoFree1 FPKM	LsoFree2 FPKM	LsoFree3 FPKM	LsoB1 FPKM	LsoB2 FPKM	LsoB3 FPKM	LsoA1 FPKM	LsoA2 FPKM	LsoA3 FPKM
Solyc06g076020.3	<0.001	0.01	4.79	6.51	5.45	4.66	2.69	4.97	2.08	44.4	270.44	358.95	114.02	132.63	191.78
Solyc02g081980.3	0.021	0.089	4.78	2.03	18.53	3.19	4.81	3.02	0.38	20.74	30.62	4.38	166.75	133.79	353.04
Solyc01g060020.3	<0.001	0.014	4.77	0	0	0	0	0	0	55.72	40.21	74.79	111.91	7.86	20.58
Solyc10g005320.3	0.008	0.052	4.7	0.06	0.47	0.08	0.72	0.48	2.84	1.11	8.93	20.56	3.79	9.58	172.7
Solyc12g096770.1	0.003	0.031	4.69	2.88	18.76	4.54	4.48	4.21	1.95	47.55	48.4	14.78	160.15	116.52	294.85
Solyc02g093180.3	<0.001	0.008	4.66	0.13	0.29	0	0.07	0.06	0.05	5.07	4.9	5.73	14.29	13.3	59.11
Solyc04g077980.1	0.001	0.015	4.66	9.52	4.83	4.45	5.69	4.38	7.95	135.7	544.66	955.56	205.99	643.75	93.28
Solyc07g040960.1	0.002	0.024	4.66	1.08	1.13	2.17	1.84	1.9	1.94	71.65	363.21	589.85	115.55	592.52	30.52
Solyc11g071740.2	0.004	0.037	4.64	0.72	0.41	0.17	1.06	0.33	0.93	10	299.23	246.25	98.02	187.57	30.21

TomatoID	p-value	q-value	DE in Infected	Control1 FPKM	Control2 FPKM	Control3 FPKM	LsoFree1 FPKM	LsoFree2 FPKM	LsoFree3 FPKM	LsoB1 FPKM	LsoB2 FPKM	LsoB3 FPKM	LsoA1 FPKM	LsoA2 FPKM	LsoA3 FPKM
Solyc03g116890.3	0.001	0.015	4.56	1.15	0.66	0.78	0.42	1.57	1.15	11.88	135.1	124.23	76.34	41.78	63.03
Solyc04g074770.2	0.001	0.022	4.55	4.02	5.04	2.17	4.26	2.65	0.69	13.41	24.18	25.6	38.15	59.07	258.85
Solyc11g011030.2	<0.001	0.01	4.51	6.71	17.45	9.57	14.75	13.39	15.34	78.57	221.14	122.07	254.46	229.37	665.13
Solyc09g010980.1	0.002	0.027	4.49	1.52	3.82	2.05	0.54	0.47	1.16	12.16	161.04	165.44	94.53	67.95	34.51
Solyc04g079730.1	0.006	0.043	4.4	0.94	0.63	1.19	0.56	0.88	0.85	1.78	49.94	19.84	69.07	32.17	76.4
Solyc03g098760.2	0.006	0.043	4.38	0	0	0.25	0	0	0.09	0.76	0.93	0.6	4.88	1.72	91.08
Solyc11g018777.1	0.012	0.066	4.31	0.39	1.75	0.8	0	0.39	0.42	4.05	210.27	151.57	112.34	44.89	26.86
Solyc00g012430.1	<0.001	0.008	4.3	11.86	21.01	13.82	12.65	11.38	15.4	87.75	137.57	205.75	439.23	471.66	303.3
Solyc02g090970.1	<0.001	0.013	4.29	0.24	0.19	0.17	0.4	0.24	0.96	6	39.35	49.64	44.58	48.45	26.6
Solyc03g098730.1	0.001	0.022	4.26	7.52	3.19	4.51	2.72	6.93	4.88	32.8	326.27	454.8	232.45	136.55	125.59
Solyc06g066800.2	0.01	0.058	4.25	1.49	8.91	3.62	2.08	2.81	0.65	6.8	169.52	157.14	77.03	61	130.34
Solyc07g056000.2	0.006	0.042	4.25	0.38	1.52	0.33	0.57	0.35	0.1	9.65	52.98	248.06	59.86	168.37	18.02

TomatoID	p-value	q-value	DE in Infected	Control1 FPKM	Control2 FPKM	Control3 FPKM	LsoFree1 FPKM	LsoFree2 FPKM	LsoFree3 FPKM	LsoB1 FPKM	LsoB2 FPKM	LsoB3 FPKM	LsoA1 FPKM	LsoA2 FPKM	LsoA3 FPKM
Solyc02g083880.3	<0.001	0.007	4.24	1.43	0.33	0	0	0.17	0	8.75	35.39	29.05	13.43	12.59	32.75
Solyc07g042170.3	<0.001	0.012	4.24	9.52	11.51	9.22	12.47	11.53	7.2	46.49	266.42	189.34	234.86	210.16	411.81
Solyc04g074430.1	0.005	0.039	4.13	0.74	4.47	2.62	1.4	0.98	0.81	21.26	130.95	320.21	77.4	176.51	23.45
Solyc08g036640.3	0.019	0.082	4.11	0	0	0	0	0	0.91	0	43.83	14.03	30.48	16.84	37.56
Solyc04g007580.1	0.006	0.044	4.1	2.12	1.58	1.71	0.78	2.02	0.75	34.23	370.54	536.07	142.52	303.87	19.67
Solyc12g049400.2	0.003	0.029	4.1	1.24	1.61	0.57	6.15	2.12	4.59	11.92	22.97	18.98	29.4	45.73	158
Solyc07g006560.3	0.008	0.052	4.09	0.29	1.6	0.53	4.55	3.32	4.32	34.87	20.89	4.91	43.66	51.63	143.25
Solyc05g015800.3	0.003	0.033	4.08	0.41	0.45	0.28	0	0.61	0.15	2.35	4.58	1.39	9.5	10.48	64.28
Solyc10g086180.2	0.007	0.047	4.08	6.36	27.85	7.76	12.23	15.24	4.59	54.48	76.09	33.4	208.97	169.98	615.77
Solyc08g076970.3	0.001	0.02	4.06	19.66	89.31	40.09	108.68	68.35	102	942.52	537.55	353.55	1329.68	1093.23	1799.57

TomatoID	p-value	q-value	DE in Infected	Control1 FPKM	Control2 FPKM	Control3 FPKM	LsoFree1 FPKM	LsoFree2 FPKM	LsoFree3 FPKM	LsoB1 FPKM	LsoB2 FPKM	LsoB3 FPKM	LsoA1 FPKM	LsoA2 FPKM	LsoA3 FPKM
Solyc11g071760.3	0.014	0.071	4.06	0	0	0	0	0	0	1.19	100.76	75.83	39.74	31.03	19.57
Solyc06g051940.3	0.001	0.021	4.04	0.4	1.52	0.43	0.88	0.19	0.48	3.64	4.18	4.48	22.4	12.65	50.51
Solyc01g007030.3	0.004	0.035	3.99	0.1	0	0.14	0	0.1	0.19	5.87	103.62	79.34	34.72	80.11	11.27
Solyc03g013160.3	0.001	0.017	3.99	3.83	2.87	2.12	1.42	2.93	1.89	8.93	15.55	16.92	37.79	20.27	125.32
Solyc12g019320.2	0.001	0.016	3.99	1.86	7.5	2.58	8.09	4.4	3.72	29.14	45.27	35.78	76.07	55.31	203.35
Solyc11g018805.1	0.009	0.054	3.98	0.27	1.3	0.39	0.47	0	0	5.65	157.15	118.89	85.55	33.26	15.99
Solyc07g041920.3	0.014	0.072	3.88	3.91	15.13	3.62	2.4	9.15	0.76	87	25.72	9.3	67.02	114.14	162.21
Solyc00g071180.3	0.013	0.068	3.87	0	0	0	0	0	0	0.05	0.08	0.13	1.06	3.96	59.38
Solyc12g017460.1	<0.001	0.007	3.85	11.16	7.96	7.43	6.9	6.63	6.36	32.23	104.01	111.78	83.41	58.61	196.61
Solyc03g026280.3	0.011	0.061	3.8	0.55	0.21	0.42	0.61	0.27	0.65	21.53	113.56	180.79	23.85	210.4	7.37
Solyc02g087210.3	0.011	0.062	3.79	3.03	2.43	1.69	2.2	4.7	1.88	6.43	205.27	155.72	92.92	99.35	95.73
Solyc10g086500.1	0.004	0.034	3.78	7.67	20.99	14.63	34.51	9.24	12.34	51.56	102.96	41.08	236.26	200.69	388.19
Solyc00g145170.2	0.012	0.063	3.77	0	0	0	0	0	0	0.71	0.39	0	5.55	9.46	34.77

TomatoID	p-value	q-value	DE in Infected	Control1 FPKM	Control2 FPKM	Control3 FPKM	LsoFree1 FPKM	LsoFree2 FPKM	LsoFree3 FPKM	LsoB1 FPKM	LsoB2 FPKM	LsoB3 FPKM	LsoA1 FPKM	LsoA2 FPKM	LsoA3 FPKM
Solyc11g018800.2	0.01	0.06	3.77	0.3	1.02	0.19	0.12	0	0.12	2.22	75.16	43.17	65.33	20.76	16.84
Solyc08g036660.3	0.006	0.044	3.75	0	0	0	0	0	0	0.43	16.14	7.39	16.18	6.1	29.57
Solyc12g057060.2	0.002	0.027	3.74	6.24	6.08	8.54	19.98	4.27	14.85	19.87	31.75	29.45	78.92	98.64	205.19
Solyc02g087540.2	0.003	0.031	3.72	0.38	0.59	0.2	0.31	0.42	0.33	11.03	92.82	163.58	45.12	51.02	11.53
Solyc08g007830.1	0.018	0.081	3.72	0	0	0	0	0	0.09	0.59	57.71	47.73	28.85	16.16	17.64
Solyc06g073830.1	0.003	0.034	3.69	0.91	0.45	0.67	1.15	0.46	0.68	3	40.93	48.76	13.62	20.92	32.07
Solyc08g068600.3	0.002	0.028	3.68	1.8	2.12	1	1.49	1.32	0.38	26.6	123.4	269.61	68.21	118.51	17.64
Solyc11g071470.1	<0.001	0.003	3.65	0.05	0	0.05	0	0.05	0	4.93	12.64	9.76	16.36	6.87	16.73
Solyc01g096670.3	0.002	0.025	3.64	3.98	9.99	4	6.71	6.16	2.46	22.97	28.83	23.62	46.6	47.53	204.85
Solyc05g005150.1	<0.001	0.006	3.64	6.88	10.94	4.86	4.04	6.08	4.38	39.33	98.51	117.88	94.19	141.18	91.96

TomatoID	p-value	q-value	DE in Infected	Control1 FPKM	Control2 FPKM	Control3 FPKM	LsoFree1 FPKM	LsoFree2 FPKM	LsoFree3 FPKM	LsoB1 FPKM	LsoB2 FPKM	LsoB3 FPKM	LsoA1 FPKM	LsoA2 FPKM	LsoA3 FPKM
Solyc06g035700.1	0.024	0.096	3.64	0	0	0	0	0	0	0.56	51.36	32.09	55.1	41.63	12.43
Solyc01g007040.3	0.006	0.045	3.63	0	0	0.06	0.2	0	0	4.31	84.86	58.2	27.28	60.53	8.72
Solyc04g072375.1	0.001	0.015	3.63	1.18	0.41	1.89	0.06	0.78	0.53	5.53	13.59	23.93	12.08	4.84	27.91
Solyc02g079590.3	<0.001	0.004	3.62	0.93	1.25	0.68	1.4	1.51	1.74	16.01	31.29	57.86	21.86	20.18	36.83
Solyc01g068065.1	0.002	0.025	3.61	0.17	5.77	5.31	0	0	4.74	14.59	11.57	11.06	13.28	11.01	12.39
Solyc11g010250.1	0.001	0.019	3.61	5.11	10.34	8.95	7.05	6.06	2.74	54.62	272.95	267.94	112.79	267.61	71.77
Solyc02g086300.3	<0.001	0.002	3.6	0	0.47	0.08	0.21	0	0.16	5.8	14.48	17.95	11.06	16.95	14.02
Solyc12g057160.1	0.012	0.065	3.6	0.91	3.06	1.09	1.28	0.06	0.31	6.69	97.51	104.55	42.18	79.51	12.99
Solyc11g010170.2	0.006	0.044	3.58	2.92	3.67	2.85	4.54	4.28	2.85	7.8	16.93	9.88	45.75	43.34	146.4
Solyc11g044620.1	<0.001	0.013	3.55	1.32	2.93	3	2.25	1.19	2.67	9.28	11.27	16.65	40.79	45.25	35.03
Solyc07g064650.2	<0.001	0.001	3.54	0	0	0.36	0.61	0	0.44	7.98	10.14	12.1	9.91	6.57	15.89
Solyc10g076660.2	0.001	0.02	3.54	0.04	0	0	0.05	0.07	0.1	1.09	3.13	3.72	2.34	5.41	28.52
Solyc01g095150.3	0.003	0.029	3.53	37.98	27.95	36.46	28.68	28.18	30.24	66.16	677.63	570.84	403.32	443.63	469.65

TomatoID	p-value	q-value	DE in Infected	Control1 FPKM	Control2 FPKM	Control3 FPKM	LsoFree1 FPKM	LsoFree2 FPKM	LsoFree3 FPKM	LsoB1 FPKM	LsoB2 FPKM	LsoB3 FPKM	LsoA1 FPKM	LsoA2 FPKM	LsoA3 FPKM
Solyc04g074420.1	0.002	0.024	3.53	0.24	0.67	0.11	0.59	0.14	0.12	5.53	31.87	49.13	24.09	53.95	11.49
Solyc06g082530.2	<0.001	0.013	3.53	3.28	5.96	3.06	3.9	3.71	2.89	14.96	24.91	18.86	37.77	31.21	110.71
Solyc04g082140.3	0.015	0.072	3.52	3.7	30.09	8.06	8.86	7.73	0.67	25.19	109.42	87.55	117.74	112.39	177.41
Solyc00g095860.1	0.005	0.039	3.51	1.62	1.39	1.33	2.07	1.81	1.98	6.16	99.83	89.65	46.93	56.85	0
Solyc10g009270.3	0.001	0.016	3.5	0.21	0.37	0.24	0.24	0	0.09	2.86	3.72	1.66	12.14	7.53	20.81
Solyc01g108240.3	0.012	0.065	3.44	0	0	0	0	0	0.05	0.48	31.47	13	23.77	13.99	15.3
Solyc06g083130.3	0.01	0.058	3.44	0.49	0.55	0.27	0	0	0.14	0.61	0.76	0.33	7.71	9.03	24.94
Solyc09g011860.3	0.009	0.056	3.44	0.75	0.82	0.45	0.2	0.92	0.26	4.14	91.05	94.95	36.97	37.22	16.58
Solyc09g061840.3	0.001	0.019	3.4	13.3	11.67	14.01	10.04	24.04	11.17	55.72	124.65	156.04	75.53	76.04	360.06

TomatoID	p-value	q-value	DE in Infected	Control1 FPKM	Control2 FPKM	Control3 FPKM	LsoFree1 FPKM	LsoFree2 FPKM	LsoFree3 FPKM	LsoB1 FPKM	LsoB2 FPKM	LsoB3 FPKM	LsoA1 FPKM	LsoA2 FPKM	LsoA3 FPKM
Solyc10g005480.3	0.001	0.021	3.38	2.06	1.87	1.3	1.38	1.66	2.33	14.05	82.66	114.63	65.75	46.91	22.25
Solyc04g005040.1	0.008	0.052	3.34	0.88	1.54	1.23	1.36	0.65	0.81	7.56	99.22	89.72	53.21	82.96	12.73
Solyc10g084320.2	0.042	0.135	3.34	4.02	8.89	1.84	7.17	5.46	5.28	9.23	21.4	3	50.51	59.4	217.6
Solyc07g052203.1	0.001	0.017	3.33	1.16	4.16	1.19	2.12	1.24	1.76	7.89	34.92	28.28	41.2	20.89	38.03
Solyc03g096050.3	0.007	0.047	3.3	1.37	3.05	1.19	1.05	2.64	0.37	5.46	7.88	4.84	19.8	20.75	61.98
Solyc06g061200.1	0.009	0.054	3.3	3.39	39.17	11.06	23.37	17.75	9.98	111.42	99.91	60.06	281.67	232.85	265.89
Solyc09g072750.2	0.038	0.128	3.3	0.49	15.09	2.19	6.51	5.74	0.84	18.03	45.07	7.96	52.81	28.71	176.22
Solyc12g014430.2	0.001	0.017	3.3	0.68	1.5	0.72	0.9	0.94	0.24	4.4	6.95	4.81	11.29	10.34	36.52
Solyc02g077330.3	0.012	0.064	3.29	0	5.77	0.09	0.09	0.11	0	2.68	1.55	3.3	9.85	3.56	26.97
Solyc03g122340.3	0.004	0.037	3.29	7.2	8.35	8.73	9.14	7.01	7.74	13.12	117.8	110.58	119.96	113.19	121.72

TomatoID	p-value	q-value	DE in Infected	Control1 FPKM	Control2 FPKM	Control3 FPKM	LsoFree1 FPKM	LsoFree2 FPKM	LsoFree3 FPKM	LsoB1 FPKM	LsoB2 FPKM	LsoB3 FPKM	LsoA1 FPKM	LsoA2 FPKM	LsoA3 FPKM
Solyc08g068610.3	0.005	0.038	3.29	0.94	1.1	0.75	1.18	0.97	0.48	19.4	89.75	189.84	41.66	78.04	9.92
Solyc10g075110.2	0.001	0.015	3.29	0	0.4	0.4	0.62	0.24	1.27	3.45	2.14	2.5	6.18	7.61	22.52
Solyc12g087790.1	0.01	0.058	3.29	2.47	3.76	0.71	1.22	0.93	1.21	12.31	164.28	104.73	60.94	93.22	15.24
Solyc07g045350.3	<0.001	0.004	3.27	0.98	1.26	0.99	2.68	0.7	2.49	15.66	15.38	28.97	14.22	14.31	24.16
Solyc06g074030.1	0.008	0.049	3.26	8.28	5.47	6.31	6.29	6.31	5.7	107.88	313.65	544.68	114.89	488.71	30.06
Solyc10g050970.1	0.027	0.104	3.26	0	0	0	0	0	0	1.02	38.95	55.14	19.94	48.64	7.01
Solyc12g009000.1	0.009	0.055	3.22	0.45	0.39	0.5	0.64	0.31	0.64	7.53	60.57	72.23	24.78	82.42	6.8
Solyc12g010420.1	0.009	0.053	3.21	9.44	4.17	7.89	14.88	3.35	16.79	18.63	154.36	129	66.29	131.1	66.67
Solyc05g006220.3	0.005	0.041	3.18	5.16	5.69	4.8	3.16	8.65	4.02	11.31	40.49	15.11	36.51	38.1	144.47
Solyc10g076250.2	0.016	0.076	3.17	1.75	1.37	1.74	4.8	3.03	9.24	4.98	8.3	5.17	10.75	13.19	113.23

TomatoID	p-value	q-value	DE in Infected	Control1 FPKM	Control2 FPKM	Control3 FPKM	LsoFree1 FPKM	LsoFree2 FPKM	LsoFree3 FPKM	LsoB1 FPKM	LsoB2 FPKM	LsoB3 FPKM	LsoA1 FPKM	LsoA2 FPKM	LsoA3 FPKM
Solyc08g079090.3	0.026	0.102	3.16	0.83	11.95	3.14	1.7	1.14	0.07	4	43.07	25.87	46.96	26.3	32.67
Solyc08g074682.1	<0.001	0.002	3.15	0	0	0	0	0	0	4.65	9.49	10.08	6.36	3.99	10.51
Solyc03g124110.2	0.034	0.118	3.14	0.04	0.02	0	0.02	0.02	0.05	3.78	63.99	90.77	17.25	87.99	3.83
Solyc04g063210.3	0.004	0.036	3.14	2.71	2.08	1.52	1.91	1.34	0.61	5.17	9.64	4.12	25.09	21.27	38.02
Solyc08g081550.3	0.008	0.049	3.14	1.28	1.33	0	1.8	1.56	0	5.13	71.92	63.66	33.87	42.2	27.84
Solyc08g068710.1	0.007	0.048	3.12	7.11	5.29	6.42	1.15	2.31	2.34	49.76	115.48	114.84	75.2	40.85	14.78
Solyc01g005865.1	0.009	0.054	3.11	0.3	0	0	0	0	1.51	0	0.94	0	0.58	1.25	3.76
Solyc01g005870.2	0.009	0.054	3.11	1.73	3.34	3.4	7.92	2.61	11.24	6.08	15.27	10.59	25.43	26.11	90.12
Solyc01g105650.3	0.012	0.065	3.1	0	0	0	0	0	0	0.05	6	3.62	1.91	1.45	23.98
Solyc12g009240.1	0.016	0.075	3.09	0	0.07	0.08	0.13	0.05	0.27	13.36	45.7	61.91	12.17	88.2	2.86
Solyc01g058500.3	0.002	0.025	3.08	77.69	110.33	62.64	66.28	58.03	84.34	181.61	276.58	525.02	1022.17	968.8	648.58
Solyc06g075690.3	0.031	0.113	3.08	6.76	2.22	2.82	1.08	2.68	1.34	6.87	134.77	173.95	82.63	57.39	22.15

TomatoID	p-value	q-value	DE in Infected	Control1 FPKM	Control2 FPKM	Control3 FPKM	LsoFree1 FPKM	LsoFree2 FPKM	LsoFree3 FPKM	LsoB1 FPKM	LsoB2 FPKM	LsoB3 FPKM	LsoA1 FPKM	LsoA2 FPKM	LsoA3 FPKM
Solyc06g083040.3	0.015	0.074	3.08	54.89	78.07	50.55	52.88	32.6	25.35	135.81	92.83	31.07	359.77	296.27	633.23
Solyc07g006890.1	0.001	0.018	3.08	5.1	8.62	5.6	8.24	5.53	4.17	31.16	162.86	148.93	123.02	137.28	56.21
Solyc06g074530.1	0.001	0.021	3.07	5.46	9.53	4.83	3.94	5.74	2.1	38.87	121.69	181.67	62.39	119.45	42.68
Solyc12g005450.1	0.003	0.03	3.07	0	0	0.01	0	0	0	2.62	24.8	24.24	11.55	23.03	6.36
Solyc04g074950.3	0.002	0.025	3.06	6.63	4.66	6.12	7.04	4.75	6.83	21.62	133.38	122.84	121.3	52.62	56.3
Solyc07g048060.2	0.027	0.103	3.06	1.26	2.31	0.2	0	0.43	0.37	1.99	57.93	49.13	14.23	11.72	15.37
Solyc08g077020.1	0.01	0.058	3.04	2.73	5.18	2.43	2.9	2.17	1.74	13.97	127.96	153.71	83.86	105.96	20.37
Solyc01g097520.3	0.001	0.014	3.03	22.92	20.87	20.53	30.08	27.81	18.03	80.07	189.97	143.5	113.69	125.42	417.03
Solyc03g111290.1	0.016	0.076	3.03	0	0.12	0.03	0.19	0.39	0.08	0.96	17.54	4.4	10.37	1.9	24.2
Solyc03g119980.3	0.005	0.039	3.03	4.46	11.55	5.32	11.61	6.51	5.77	18.8	25.8	16.76	50.6	56.22	143.67

TomatoID	p-value	q-value	DE in Infected	Control1 FPKM	Control2 FPKM	Control3 FPKM	LsoFree1 FPKM	LsoFree2 FPKM	LsoFree3 FPKM	LsoB1 FPKM	LsoB2 FPKM	LsoB3 FPKM	LsoA1 FPKM	LsoA2 FPKM	LsoA3 FPKM
Solyc01g090180.3	0.034	0.118	3.02	5.95	4.83	4.43	3.45	2.18	0.69	5.26	3.84	1.61	31.43	23.75	60.72
Solyc01g059990.3	0.015	0.072	3	0	0	0	0	0	0.01	46.59	26	53.49	16.29	4.29	3.05
Solyc03g007240.3	0.004	0.034	3	11.61	13.33	8.57	13.82	15.72	14.33	34.1	37.55	29.84	98.53	71.23	254.29
Solyc03g093080.3	0.032	0.116	2.99	0.34	0.59	0	0.1	0.2	0	2.43	57.56	66.89	43.89	54.92	5.37
Solyc03g096290.3	0.007	0.048	2.99	73.34	39.12	64.31	22.49	30.4	7.67	97.77	108.5	115.81	111.02	87.76	398.95
Solyc07g006570.3	0.02	0.087	2.99	2.72	14.6	7	17.74	12.38	9.77	67.88	40.47	11.71	77.28	91.98	187.46
Solyc07g007760.3	0.012	0.064	2.99	14.39	20.94	25.61	4.47	20.73	3.01	55.16	164.38	70.27	224.34	102.2	122.47
Solyc01g087590.3	0.031	0.112	2.98	0	2	0.19	0.81	0.75	0.39	2.12	80.09	22.76	56.62	13.93	19.19
Solyc02g085730.3	<0.001	0.014	2.98	40.46	51.51	45.83	52.86	35.1	38.9	85.86	186.66	189.87	215.91	177.04	614.27

TomatoID	p-value	q-value	DE in Infected	Control1 FPKM	Control2 FPKM	Control3 FPKM	LsoFree1 FPKM	LsoFree2 FPKM	LsoFree3 FPKM	LsoB1 FPKM	LsoB2 FPKM	LsoB3 FPKM	LsoA1 FPKM	LsoA2 FPKM	LsoA3 FPKM
Solyc08g006740.3	<0.001	0.01	2.98	0.39	0.86	0.33	1.07	0.45	0.77	3.6	5.18	8.48	8.32	12.09	17.96
Solyc09g056385.1	0.001	0.018	2.98	23.28	36.68	53.84	42.15	11.1	16.4	75.77	153.06	170.59	179.71	171.26	164.61
Solyc10g078495.1	<0.001	0.014	2.97	0	0.64	0.39	0.41	0	0	1.63	4.15	2.83	3.3	4.13	16.09
Solyc01g007955.1	0.005	0.038	2.96	1.29	1.25	1.12	2.17	1	1.05	2.46	5.35	3.13	8.78	11.2	38.4
Solyc10g080690.2	0.04	0.132	2.96	0.32	10.65	1.86	2.4	1.52	0.07	2.34	18.22	11.32	26.58	13.44	51.36
Solyc02g090360.3	0.014	0.072	2.95	1.85	4.1	1.1	1.38	1.26	0.61	4.84	62.99	43.6	70.4	39.21	18.91
Solyc02g080200.3	0.039	0.128	2.93	2.38	31.03	1.89	3.18	0	0.75	46.71	16.04	0	60.28	28.09	97.19
Solyc02g080210.3	0.039	0.128	2.93	0.02	2.42	0	0.11	1.35	0	1.7	0.07	1.59	2.65	1.08	3.33
Solyc00g272810.1	0.007	0.046	2.92	5.58	12.14	13.46	40.29	12.99	30.41	49.82	144.2	161.14	91.38	99.29	210.63
Solyc01g007020.3	0.006	0.043	2.92	1.15	1.29	1.22	0.53	0.83	0.36	4.94	46.56	36.72	16.72	38.65	10.74
Solyc04g074480.3	<0.001	0.004	2.91	31.45	45.67	30.48	50.7	34.95	34.83	182.97	249.13	337.76	220.11	208.24	410.98
Solyc11g071550.2	0.005	0.039	2.91	1.93	1.88	1.52	2.82	1.27	2.19	5.18	10.08	1.98	9.88	10.3	44.42
Solyc01g091165.1	0.028	0.106	2.9	0.26	0	0	0.04	0	0.12	0.21	1.19	0.24	15.04	3.66	14.67

TomatoID	p-value	q-value	DE in Infected	Control1 FPKM	Control2 FPKM	Control3 FPKM	LsoFree1 FPKM	LsoFree2 FPKM	LsoFree3 FPKM	LsoB1 FPKM	LsoB2 FPKM	LsoB3 FPKM	LsoA1 FPKM	LsoA2 FPKM	LsoA3 FPKM
Solyc02g077050.3	0.001	0.017	2.9	5.89	11.21	5.47	4.3	5.68	1.94	38.04	89.45	106.55	52.8	46.16	49.18
Solyc06g082535.1	0.001	0.02	2.89	15.29	26.68	12.55	18.17	17.64	14.28	42.83	76.78	59.94	101.58	89.48	268.23
Solyc04g082200.2	0.006	0.045	2.88	68.92	67.63	65.4	98.47	85.7	123.99	153.62	735.97	855.28	278.05	342.58	1245.46
Solyc07g063410.3	0.014	0.07	2.88	4.5	2.67	5.02	9.41	5.61	15.88	8.21	13.39	13.06	36.14	26.08	114.2
Solyc02g078890.1	0.03	0.11	2.87	3.73	3.86	2.55	10.27	8.05	4.46	10.03	60.46	14.77	109.12	53.72	134.57
Solyc08g081555.1	0.011	0.061	2.87	1.93	1.22	0.76	2.19	2.12	1.6	4.52	71.07	58.3	30.4	39.96	26.43
Solyc10g076240.2	0.028	0.106	2.86	0	0	0.1	0	0	0	14.61	7.77	70.96	2.54	3.96	4.19
Solyc07g056480.3	<0.001	0.01	2.85	33.22	28.59	29.62	46.34	26.07	37.13	91.44	94.32	96.98	128.74	119.31	413.89
Solyc10g084880.3	0.007	0.046	2.85	2.24	1.15	1.32	1.46	1.68	1.85	15.57	85.56	82.72	21.1	77.09	11.31
Solyc03g118810.1	0.002	0.026	2.84	49.37	26.47	45.97	49.03	29.65	56.34	128.71	573.67	543.75	354.26	550.79	228.98
Solyc06g034370.1	0.004	0.035	2.83	0.23	0.95	0	0.75	0.31	0.21	6.52	18	25.44	11.89	2.74	13.02
Solyc02g080120.2	0.002	0.027	2.82	0.52	0.65	1.52	0.6	0.59	1.47	1.4	5.05	4.22	1.83	4.25	23.42

TomatoID	p-value	q-value	DE in Infected	Control1 FPKM	Control2 FPKM	Control3 FPKM	LsoFree1 FPKM	LsoFree2 FPKM	LsoFree3 FPKM	LsoB1 FPKM	LsoB2 FPKM	LsoB3 FPKM	LsoA1 FPKM	LsoA2 FPKM	LsoA3 FPKM
Solyc10g085190.2	0.012	0.065	2.82	0	0	0	0.05	0	0	0.39	1.8	1.44	0.24	0.47	21.09
Solyc03g093360.3	0.033	0.116	2.81	0.43	15.45	2.14	8.78	4.95	1.12	30.97	66.38	15.32	95.87	74.43	61.44
Solyc03g117600.3	0.001	0.018	2.81	4.01	7.13	4.52	7.17	5.66	5.38	18.88	20.04	14.79	30.11	26.99	89.51
Solyc09g083360.3	0.006	0.042	2.81	0.55	0.25	0.38	0.48	0.72	0.71	1.96	22.59	12.11	20.08	7.58	15.29
Solyc10g080370.1	0.003	0.032	2.81	26.18	48.42	47.64	39.57	23.81	16.74	117.18	412.73	479.79	464.22	412.84	174.24
Solyc01g057770.3	0.016	0.077	2.8	1.1	2.5	1.67	1.11	1.56	0.54	5.25	76.36	66	60.05	32.09	14.19
Solyc09g008060.3	<0.001	0.011	2.8	1.45	3.99	2.11	1.97	1.17	1.14	5.63	8.36	10.13	10.26	11.64	27.24
Solyc09g066360.1	<0.001	0.013	2.8	0	0	0	0.62	0.1	0.37	6.34	4.74	13.88	3.73	3.3	9.31
Solyc04g071770.3	0.01	0.058	2.79	0.05	0.07	0.13	0.4	0	0.62	0.61	3.24	6.59	0.66	1.56	17.19
Solyc06g072460.1	0.006	0.044	2.78	2.33	1.31	1.93	2.01	1.62	1.48	10.16	64.49	85.27	19.89	18.3	17.26
Solyc10g054440.2	0.001	0.019	2.78	30.1	43.68	21.55	32.4	41.59	43.96	109.93	442.16	449.42	268.18	262.05	367.54

TomatoID	p-value	q-value	DE in Infected	Control1 FPKM	Control2 FPKM	Control3 FPKM	LsoFree1 FPKM	LsoFree2 FPKM	LsoFree3 FPKM	LsoB1 FPKM	LsoB2 FPKM	LsoB3 FPKM	LsoA1 FPKM	LsoA2 FPKM	LsoA3 FPKM
Solyc01g079200.3	0.043	0.137	2.77	0.43	0.51	0.25	0.51	0.14	0.29	1.2	59.22	40.15	35.82	14.91	7.78
Solyc03g093610.1	<0.001	0.011	2.77	3.13	5.11	4.02	4.32	3.79	2.5	34.18	65.24	94.5	27.72	44.05	28.55
Solyc04g079360.1	0.001	0.02	2.77	0.26	0.36	0.75	0.79	1.06	0.48	5.79	29.59	31.51	16.38	30.14	9.79
Solyc09g091600.3	0.003	0.029	2.77	1.7	3.59	2.47	4.42	4.15	0.26	28.48	37.15	78.53	28.98	18.73	36.19
Solyc02g089685.1	<0.001	<0.001	2.76	0	0	0	0	0	0	4.93	8.36	8.55	6.86	7.68	5.37
Solyc00g011160.2	0.003	0.032	2.74	63.64	91.32	58.44	55.8	45.36	55.65	116.69	177.94	306.97	620.66	605.21	408.49
Solyc12g005640.2	0.002	0.026	2.74	0.25	0	0.21	0	0.34	0.66	0.96	2.76	3.26	6.02	2.58	14.12
Solyc12g042073.1	<0.001	<0.001	2.74	0	0	0	0.05	0	0	6.52	5.17	5.25	5.63	8.48	5.18
Solyc06g083440.3	0.002	0.026	2.73	3	9.91	4.51	5.27	3.87	1.62	15.32	18.51	17.69	30.48	20.24	57.48
Solyc08g008280.3	0.037	0.126	2.73	5.35	3.65	3.64	2.05	2.73	2.13	18.64	170.04	276.32	130.43	71.1	12.16
Solyc10g075100.2	0.042	0.135	2.73	81.6	378.41	48.51	36.45	222.82	33.92	865.57	340.43	842.77	812.35	293.8	1316.9

TomatoID	p-value	q-value	DE in Infected	Control1 FPKM	Control2 FPKM	Control3 FPKM	LsoFree1 FPKM	LsoFree2 FPKM	LsoFree3 FPKM	LsoB1 FPKM	LsoB2 FPKM	LsoB3 FPKM	LsoA1 FPKM	LsoA2 FPKM	LsoA3 FPKM
Solyc07g008560.3	0.001	0.02	2.72	10.68	4.86	6.84	18.15	5.05	21.38	57.11	49.38	42.3	66.48	48.45	61.02
Solyc01g098910.3	0.003	0.032	2.71	1.57	2.34	1.48	1.98	2.72	2.63	6.97	48.16	46.57	33.76	19.64	27.12
Solyc04g054990.3	0.024	0.097	2.71	3.84	9.37	4.87	18.19	8.24	5.76	22.42	18.79	12.79	77.53	63.42	130.46
Solyc09g089500.3	0.024	0.097	2.71	0	0	0	0	0.14	0	0	0	0	0.15	2.51	19.79
Solyc03g080190.3	0.005	0.04	2.7	17.76	14.62	14.26	6.43	16.95	5.33	92.16	137.01	235.39	79.18	40.6	91.56
Solyc05g050800.3	<0.001	0.007	2.7	24.55	25.13	23.08	20.41	30.81	17.73	157.22	307.16	427.89	214.1	140.16	175.86
Solyc02g091180.1	0.021	0.089	2.69	0.26	0.14	0	0.58	0.16	0.47	6.76	35.66	41.84	10.57	68.12	3.52
Solyc07g042230.1	0.026	0.101	2.69	0	0	0	0	0	0	0.16	21.52	10	9.78	5.08	8.85
Solyc12g011030.2	0.015	0.074	2.69	0.04	1.52	0.24	0.08	0.17	0	1.63	26.4	8.68	22.17	15.11	7.52
Solyc03g117270.1	0.005	0.039	2.68	6.81	9.32	5.59	14.3	6.79	13.9	24.18	35.53	14.93	63.55	49.15	105.83
Solyc04g009440.3	0.003	0.028	2.68	6.14	6.69	4.24	9.33	7.69	5.11	27.45	143.52	138.5	105.3	41.48	68.38

TomatoID	p-value	q-value	DE in Infected	Control1 FPKM	Control2 FPKM	Control3 FPKM	LsoFree1 FPKM	LsoFree2 FPKM	LsoFree3 FPKM	LsoB1 FPKM	LsoB2 FPKM	LsoB3 FPKM	LsoA1 FPKM	LsoA2 FPKM	LsoA3 FPKM
Solyc02g064680.3	0.009	0.056	2.67	7.73	7.85	6.82	9.51	9.15	8	25.09	251.64	219.26	203.96	91.73	61.42
Solyc05g009470.3	0.025	0.099	2.67	4.86	27.96	6.85	16.78	4.51	11.56	28.08	15.47	9.16	58.26	63.64	88.62
Solyc06g074420.1	0.042	0.135	2.67	0.09	1.45	0.13	1.14	8.96	0.52	6.26	25.6	30.87	14.02	11.52	73.79
Solyc11g027760.1	0.004	0.037	2.67	36.06	61.62	35.94	36.11	29.99	45.69	81.05	105.82	210.76	437.15	380.93	245.19
Solyc01g109710.3	<0.001	0.014	2.66	13.23	9.98	11.94	20.71	17.54	18.28	52.25	133.93	149.67	95.31	81.36	157.82
Solyc03g122190.3	0.004	0.037	2.66	12.96	12.39	11.99	17.08	15.12	16.9	22.88	122.65	74.9	128.4	97.76	163.9
Solyc01g087020.2	0.006	0.042	2.65	8.66	4.72	8.49	4.6	4.43	6.93	13.26	85.48	77.56	58.95	61.43	32.29
Solyc03g123620.4	0.026	0.101	2.65	3.33	2.28	1.42	1.6	0.87	1.16	2.58	36.72	37.73	36.41	35.45	13.28
Solyc10g081570.2	0.043	0.138	2.65	20.82	14.35	18.69	27.55	27.08	25.41	19.35	326.66	357.54	142.75	103.18	319.51
Solyc08g007840.2	0.014	0.069	2.64	0	0.09	0	0	0	0	0.06	9.09	5.8	3.3	3.42	10.97
Solyc02g080190.3	0.03	0.11	2.63	0.44	7.41	0.47	1.19	0.93	0.29	9.74	4.03	1.72	16.61	7.04	21.3

TomatoID	p-value	q-value	DE in Infected	Control1 FPKM	Control2 FPKM	Control3 FPKM	LsoFree1 FPKM	LsoFree2 FPKM	LsoFree3 FPKM	LsoB1 FPKM	LsoB2 FPKM	LsoB3 FPKM	LsoA1 FPKM	LsoA2 FPKM	LsoA3 FPKM
Solyc09g018250.2	<0.001	<0.001	2.63	0	0	0	0	0	0	4.26	5.34	5.87	4.93	5.78	5.33
Solyc03g026270.3	0.046	0.142	2.62	0	0	0	0	0	0	1.64	23.68	55.1	21.84	32.38	2.68
Solyc12g099000.2	0.003	0.03	2.62	42.41	44.96	36.45	72.86	52.75	40.61	101.5	247.83	262.32	201.04	180.79	666.12
Solyc06g007120.3	0.001	0.019	2.61	2.56	4.73	2.52	4.05	3.43	2.93	11.58	13.38	15.35	10.68	10.36	52.92
Solyc02g071610.3	0.005	0.039	2.6	10.5	35.23	15.26	31.93	9.7	15.43	34.55	79.55	49.51	90.78	74.03	168.29
Solyc02g071720.2	0.01	0.06	2.6	0.36	0.3	0.47	0.68	0.33	0.21	0.88	1.48	0.47	4.61	4.2	17.18
Solyc04g082030.1	0.022	0.092	2.6	17.67	25.59	21.92	7.89	15.61	10.2	13.58	13.24	10.7	39.73	48.81	205.12
Solyc02g067610.1	0.014	0.072	2.59	2.85	1.07	0.97	0.9	1.49	1	6.52	69.12	62.17	39.4	29.34	10.19
Solyc07g005330.3	0.01	0.058	2.59	17.75	30.74	15.56	21.84	26.23	16.84	33.48	244.47	177.36	164.3	115.76	268.48
Solyc09g062970.1	0.02	0.086	2.59	0	3.1	0.1	0	0.18	0	2.06	1.87	0.82	2.78	0.57	17.65
Solyc02g085495.1	<0.001	<0.001	2.58	0	0	0	0	0	0	4.4	4.12	4.08	4.76	5.8	5.06

TomatoID	p-value	q-value	DE in Infected	Control1 FPKM	Control2 FPKM	Control3 FPKM	LsoFree1 FPKM	LsoFree2 FPKM	LsoFree3 FPKM	LsoB1 FPKM	LsoB2 FPKM	LsoB3 FPKM	LsoA1 FPKM	LsoA2 FPKM	LsoA3 FPKM
Solyc01g080010.2	0.001	0.015	2.57	1.15	2.58	1.21	2.05	1.16	0.78	4.96	12.66	8.96	12.03	10.89	21.43
Solyc04g011790.1	<0.001	0.004	2.57	0.2	0	0	0.35	0	0.66	3.36	3.91	4.47	6.42	3.07	7.31
Solyc09g006010.2	0.026	0.101	2.57	0.17	3.88	0.35	1.77	2.41	0.19	5.28	11.19	3.42	13.94	12.04	36.93
Solyc10g083970.1	0.001	0.016	2.57	33.98	70.01	48.67	35.97	35.2	25.82	133.01	399.54	324.96	320.41	318.42	227.98
Solyc12g008350.2	0.003	0.031	2.57	0.44	3.69	2.38	1.14	2.19	0.82	6.83	32.18	19.56	21.54	22.01	17.38
Solyc03g007230.3	0.008	0.051	2.56	1.83	2.54	2.38	4.37	2.89	12.3	6.56	8.01	9.66	16.6	14.92	43.51
Solyc01g091360.3	0.001	0.019	2.55	1.26	1.54	1.18	0.11	0.22	0.2	1.45	4.17	2.3	4.58	3.82	11.81
Solyc12g057150.1	0.03	0.111	2.55	1.17	4.62	2.53	1.31	4.17	0.32	8.32	96.59	102.89	43.78	79.29	17.27
Solyc02g083850.3	0.003	0.032	2.54	1.57	0.72	0.71	0.78	1.32	0.53	4.03	16.6	15.9	8.44	4.51	18.33
Solyc07g008380.2	0.038	0.127	2.54	0	0.57	0	0.03	0.05	0	0.66	0.3	0.05	11.99	3.01	10.2

TomatoID	p-value	q-value	DE in Infected	Control1 FPKM	Control2 FPKM	Control3 FPKM	LsoFree1 FPKM	LsoFree2 FPKM	LsoFree3 FPKM	LsoB1 FPKM	LsoB2 FPKM	LsoB3 FPKM	LsoA1 FPKM	LsoA2 FPKM	LsoA3 FPKM
Solyc00g009760.2	0.004	0.037	2.53	9.18	11.2	8.18	8.79	6.89	8.42	15.74	25.34	39.66	85.56	87.51	58.84
Solyc02g064980.1	0.01	0.057	2.53	0.18	0.31	0.12	0.35	0.54	0.28	1.51	24.05	12.41	17.64	13.54	8.56
Solyc06g075010.3	0.003	0.031	2.53	5.56	7.22	5.94	11.29	5.77	7.29	18.62	15.62	13.74	43.99	42.51	69.62
Solyc10g086690.2	0.019	0.084	2.53	0	0.17	0.14	0.38	0.95	0.13	0.96	14.45	11.27	4.16	4.01	17.21
Solyc05g008370.1	0.002	0.025	2.52	37.85	40.6	45.19	24.59	29.13	30.03	49.63	122.28	77.23	214.14	143.14	252.06
Solyc06g024210.2	0.005	0.041	2.52	23.27	30.5	21.56	21.63	18.15	28.6	39.8	54.34	107.77	208.3	210.79	138.7
Solyc04g016190.1	0.01	0.059	2.51	0.14	0.53	0	0.71	0.14	0.81	0.78	1.06	0.82	2.1	1.39	18.62
Solyc07g008550.3	0.001	0.022	2.51	1.93	0.34	1.35	2.54	0.15	4.25	5.59	5.31	4.43	9.57	6.99	10.52
Solyc03g031620.3	0.007	0.047	2.5	0.46	2.72	2.1	2.89	2.18	0	6.26	8.68	15.01	15.82	11.43	24.91
Solyc06g068650.3	0.007	0.047	2.5	13.34	23.73	14.04	22.09	23.28	15.9	37.76	62.57	37.97	97.21	80.41	257.41
Solyc09g075890.3	0.01	0.059	2.5	4.18	4.09	3.65	11.75	6.61	11.38	12.29	37.28	31.24	68.11	47.12	71.54

TomatoID	p-value	q-value	DE in Infected	Control1 FPKM	Control2 FPKM	Control3 FPKM	LsoFree1 FPKM	LsoFree2 FPKM	LsoFree3 FPKM	LsoB1 FPKM	LsoB2 FPKM	LsoB3 FPKM	LsoA1 FPKM	LsoA2 FPKM	LsoA3 FPKM
Solyc10g079350.2	0.05	0.15	2.5	0.44	4.57	1.09	0.7	0.49	0.43	1.21	0.83	0.51	12.23	7.72	17.05
Solyc04g074470.1	0.001	0.017	2.49	0.38	2.12	1.03	0.36	1.24	0.42	4.94	16.61	15.02	12.39	9.42	11.51
Solyc08g062330.3	0.001	0.016	2.49	3.26	3.07	1.75	1.32	3.61	1.36	20.97	32.73	65.08	27.51	20.08	17.22
Solyc12g099400.2	0.016	0.076	2.49	20.42	12.67	15.44	14.87	19.33	16.92	15.61	41.54	27.19	36.17	35	277.21
Solyc01g105890.3	0.029	0.107	2.48	0.09	2.94	0.41	0.75	1.42	0	3.6	10.71	1.4	6.96	5.67	25.06
Solyc06g068960.1	0.01	0.057	2.48	42.95	43.26	41.27	25.25	42.44	23.99	63.74	357.4	258.72	105.52	118.02	348.09
Solyc08g068680.3	0.022	0.091	2.48	1.61	1.88	0.72	0.8	1.05	0.33	20.41	66.92	131.53	35.7	28.16	4.67
Solyc02g032860.3	0.005	0.038	2.47	3.19	6.75	4.74	7.46	6.35	1.52	30.36	42.62	92.06	29.85	21.08	47.72
Solyc02g070040.1	0.002	0.026	2.47	0.85	2.28	0.9	1.49	1.42	0.76	7.16	21.63	19.58	26.95	40.77	10.57
Solyc03g083680.1	0.023	0.093	2.47	0.18	0.18	1.27	0.39	0.1	0	5.18	35.79	37.58	20.35	17.25	2.61
Solyc05g010420.2	0.001	0.017	2.47	459.02	504.02	349.54	321.05	568.72	332.47	1494.47	1341.7	1168.55	2990.31	2233.17	3503.66
Solyc12g097000.2	0.003	0.032	2.46	2.45	1.19	0.9	0.68	2.46	0.38	16.63	28.15	59.13	17.34	11.75	11.6

TomatoID	p-value	q-value	DE in Infected	Control1 FPKM	Control2 FPKM	Control3 FPKM	LsoFree1 FPKM	LsoFree2 FPKM	LsoFree3 FPKM	LsoB1 FPKM	LsoB2 FPKM	LsoB3 FPKM	LsoA1 FPKM	LsoA2 FPKM	LsoA3 FPKM
Solyc00g028960.1	0.04	0.131	2.45	0.3	0.22	0.2	5.17	0	6.63	2.8	4.54	4.28	9.5	10.1	12.83
Solyc02g077110.3	0.005	0.039	2.45	2.85	2.98	3.7	2.29	1.85	1.9	3.85	13.18	17.09	4.35	7.31	29.47
Solyc03g111100.1	0.008	0.05	2.45	0.07	0.69	0.07	1.27	1.21	2.43	7.06	29.21	36.65	14.32	34.39	8.51
Solyc04g008330.1	0.006	0.042	2.45	0.05	0.41	0.38	0.37	0.09	0.09	1.11	3	0.36	1.62	4.05	11.34
Solyc06g082440.1	0.005	0.039	2.45	0.86	1.5	0.72	1.28	1.23	0.7	3.38	23.11	27.03	14.68	19.16	12.83
Solyc08g074683.1	0.012	0.064	2.45	0	0.26	0	0.03	0	0	0.73	1.59	0.34	6.57	1.2	10.12
Solyc03g098720.3	0.019	0.083	2.43	0	0	0	0	0	0	0.15	0.37	0	0	0.35	15.11
Solyc03g120660.3	0.003	0.034	2.43	0.66	1.84	0.81	0.55	0.85	0.56	6.48	1.43	1.97	8.38	5.01	12.21
Solyc07g018010.3	<0.001	0.011	2.43	16.7	13.21	16.83	9.97	10.56	9.63	24.28	39.81	65.12	43.58	37.63	88.75
Solyc07g062700.3	0.006	0.043	2.43	56.12	49.24	50.22	73.44	46.77	89.57	80.32	398.18	359.67	218.87	222.39	482.93
Solyc08g068770.2	0.032	0.115	2.43	23.99	19.51	24.25	8.01	23.43	14.01	76.66	453.89	695.99	247.52	290.84	55.77
Solyc08g080585.1	0.048	0.147	2.43	0	1.83	0.5	0.92	2.41	0	45.65	74.28	50.81	13.84	5.24	14.52

TomatoID	p-value	q-value	DE in Infected	Control1 FPKM	Control2 FPKM	Control3 FPKM	LsoFree1 FPKM	LsoFree2 FPKM	LsoFree3 FPKM	LsoB1 FPKM	LsoB2 FPKM	LsoB3 FPKM	LsoA1 FPKM	LsoA2 FPKM	LsoA3 FPKM
Solyc08g080590.3	0.048	0.147	2.43	7.32	0	0	0	0	0	0	0	0	0	0	0
Solyc12g099260.2	0.016	0.075	2.43	2.22	13.55	3.99	7.45	4.28	2.42	13.5	16.98	10.83	34.98	35.85	51.5
Solyc02g089900.1	0.008	0.051	2.42	8.56	5.26	5.58	4.5	6.02	3.38	22.36	123.09	130.59	96.1	75.11	25.14
Solyc02g093250.3	0.013	0.068	2.42	2.25	2.83	1.64	0.69	1.43	0.89	9.23	53.34	55.45	32.6	28.19	7.51
Solyc04g076307.1	<0.001	0.014	2.42	3.77	1.88	3.71	4.04	0	4.03	5.41	3.44	2.28	5.54	4.01	14
Solyc06g062920.3	0.027	0.103	2.42	0.56	0.34	0.3	0.21	0.22	0.05	5.42	30.84	59.06	12.59	24.42	2.7
Solyc01g096430.3	<0.001	0.01	2.41	8.68	5.14	5.76	9.06	5.12	6.25	17.27	24.24	23.26	40.76	31.17	50.49
Solyc04g011840.1	0.002	0.027	2.41	0.36	0.24	0.1	2.58	0.64	2.27	6.17	7.3	9.31	13.75	8.31	11.69
Solyc10g075160.1	0.028	0.106	2.41	196.8	392.64	255.43	161.72	173.96	89.1	601.88	364.36	122.13	1004.21	587.41	1535.54
Solyc03g007960.3	0.003	0.029	2.4	1.08	1.12	0.85	1.56	1.97	2.06	3.81	15.36	14.82	11.64	8.26	21.71

TomatoID	p-value	q-value	DE in Infected	Control1 FPKM	Control2 FPKM	Control3 FPKM	LsoFree1 FPKM	LsoFree2 FPKM	LsoFree3 FPKM	LsoB1 FPKM	LsoB2 FPKM	LsoB3 FPKM	LsoA1 FPKM	LsoA2 FPKM	LsoA3 FPKM
Solyc06g075000.3	0.002	0.026	2.39	1.36	1.5	1.9	2.33	1.71	1.89	5.06	4.3	3.67	9.91	13.58	20.81
Solyc10g006700.1	0.029	0.109	2.39	14.55	8.14	13.08	11.91	17.58	9.96	139.82	298.07	694.63	124	510.18	33.21
Solyc12g088190.2	<0.001	0.009	2.39	1.14	2.64	1.32	1.3	0.93	1.43	9.12	5.3	8.51	4.51	4.15	14.62
Solyc05g056170.3	<0.001	0.013	2.38	5.36	5.34	2.07	4.05	3.55	3.01	14.5	12.47	20.8	21.48	15.59	35.26
Solyc09g007260.3	0.007	0.047	2.37	4.22	4.62	4.54	8.75	2.71	4.81	7.69	10.01	5.44	16.59	24.79	44.59
Solyc01g079940.3	0.031	0.113	2.36	3.06	5	3.74	10.32	2.91	5.43	5.25	60.66	41.25	53.13	41.02	37.5
Solyc01g086800.3	0.001	0.021	2.36	1.88	3.55	1.9	2.41	1.32	1.18	13.41	9.49	25.96	29.81	12.94	11.24
Solyc01g087570.2	0.005	0.039	2.36	6.36	13.48	7.54	3.68	5.45	3.05	13.32	33.41	29.09	49.19	45.47	35.06
Solyc03g117870.3	0.004	0.037	2.36	49.23	66.31	60.46	48.2	69.32	40.7	87.95	155.13	120.14	239.21	194.63	566.38
Solyc08g076980.3	0.037	0.124	2.36	0	0	0	0	0	0	0	2.39	0	0	4.64	10.57
Solyc02g071620.3	0.009	0.055	2.35	9.85	7.62	6.97	8.29	7.92	8.35	11.59	84.19	52.84	40.22	29.38	76.04
Solyc03g005470.3	0.001	0.015	2.35	17.15	33.27	22.57	29.29	19.95	22.39	134.17	131.4	292.43	108.89	99.43	118.02
Solyc12g009925.1	0.008	0.05	2.35	2.52	7.99	3.99	7.08	4.69	1.45	20.88	28.12	10.34	23.83	38.21	41.66

TomatoID	p-value	q-value	DE in Infected	Control1 FPKM	Control2 FPKM	Control3 FPKM	LsoFree1 FPKM	LsoFree2 FPKM	LsoFree3 FPKM	LsoB1 FPKM	LsoB2 FPKM	LsoB3 FPKM	LsoA1 FPKM	LsoA2 FPKM	LsoA3 FPKM
Solyc03g112880.1	0.008	0.052	2.33	1.06	2.76	0.73	1.61	1.41	1.13	3.42	3.49	2.8	9.23	9.62	21.41
Solyc07g006480.3	0.018	0.081	2.33	4.67	2.67	2.3	0.98	3.72	0.33	17.21	65.46	94	34.21	25.02	12.09
Solyc07g043500.1	0.048	0.146	2.33	8	41.1	39.93	28.39	14.7	6.02	43.4	60.55	19.39	82.57	121.39	131.43
Solyc01g066760.2	0.003	0.029	2.32	0.46	2.65	0.31	0.84	0.18	0.12	3.4	2.95	5.34	8.9	5.26	8.42
Solyc01g091465.1	0.001	0.016	2.32	0.29	0.18	0.73	0.6	0.74	0.16	7.86	8.04	23.71	9.32	6.79	5.39
Solyc09g014280.1	<0.001	0.004	2.32	24.34	18.91	16.23	28.51	21.76	24.98	116.47	144.29	179.46	112.67	89.12	132.81
Solyc02g092790.3	0.043	0.137	2.31	1.38	12.61	4.38	3.41	3.06	0.54	6.07	31.56	16.78	41.9	28.97	27.29
Solyc03g120990.3	0.015	0.073	2.31	0.07	0.36	0.1	0.07	0.43	0.06	0.64	3.48	2.66	12.36	7.63	7.67
Solyc04g071890.3	0.014	0.07	2.3	11.25	7.91	5.2	27.56	17.94	25.3	246.92	146.04	276.07	90.09	72.86	80.54
Solyc01g096320.3	0.003	0.032	2.29	0.67	0.21	0.27	2.15	1.53	1.88	6.49	4.09	7.28	10.85	7.62	15.1
Solyc02g083120.2	0.002	0.023	2.29	0.36	1.96	0.74	1.54	2.18	0.87	5.86	9.12	6.69	8.45	11.1	19.51

TomatoID	p-value	q-value	DE in Infected	Control1 FPKM	Control2 FPKM	Control3 FPKM	LsoFree1 FPKM	LsoFree2 FPKM	LsoFree3 FPKM	LsoB1 FPKM	LsoB2 FPKM	LsoB3 FPKM	LsoA1 FPKM	LsoA2 FPKM	LsoA3 FPKM
Solyc04g011830.1	0.004	0.037	2.29	1.75	0.65	0.37	5.06	0.64	2.32	7.63	14.14	11.99	8.74	11.25	14.1
Solyc08g067505.1	0.048	0.147	2.29	0.7	0	0	3.61	0	1.49	0.52	2.23	2.2	3.43	10.16	13.45
Solyc11g005150.2	0.037	0.124	2.29	0.26	3.81	0.83	0.64	0.47	0.04	1.38	9.43	3.79	18.21	12.8	9.48
Solyc00g010525.1	0.006	0.044	2.28	4.69	6.12	4.58	4.51	3.13	5.48	7.17	9.11	16.48	32.12	39.03	0
Solyc02g093270.3	0.004	0.034	2.28	12.59	5.19	3.67	7.09	9.14	5.33	31.86	48.67	78.58	26.01	25.78	58.58
Solyc03g115380.2	0.002	0.024	2.28	0	0	0.3	0.44	0	0.03	0.88	1.53	1.2	4.08	3.09	7.19
Solyc03g116510.1	0.01	0.058	2.28	3.33	2.95	2.07	2.33	1.21	3.84	18.18	25.33	58.4	13.09	10.04	9.86
Solyc11g006910.2	0.02	0.085	2.28	0	0.89	0.28	1.91	3.02	0	53.92	20.84	63.41	14.84	8.23	10.05
Solyc02g088630.3	0.013	0.067	2.27	5.21	6.23	6.45	5.38	4.91	3.18	7.72	63.36	46.05	46.44	29.43	36.46
Solyc03g097750.3	<0.001	0.008	2.27	0.77	0.22	0.51	0.97	0.49	0.18	3.91	3.58	4.02	2.94	3.75	9.74
Solyc01g009160.2	0.027	0.105	2.26	0.43	0.77	0.59	1.13	1.03	1.02	5.11	40.62	62.1	20.97	37.4	5.26
Solyc07g049370.2	0.016	0.077	2.26	0.87	3.24	0.9	1.2	1.25	0.11	2.42	4.03	2.77	7.7	4.5	21.03

TomatoID	p-value	q-value	DE in Infected	Control1 FPKM	Control2 FPKM	Control3 FPKM	LsoFree1 FPKM	LsoFree2 FPKM	LsoFree3 FPKM	LsoB1 FPKM	LsoB2 FPKM	LsoB3 FPKM	LsoA1 FPKM	LsoA2 FPKM	LsoA3 FPKM
Solyc09g064820.1	0.013	0.068	2.26	0.5	0.96	0.75	3.38	0.93	3.49	5.33	7.17	4.86	24.4	9.84	13.49
Solyc02g069110.3	0.024	0.096	2.24	2.08	4.53	2.21	4.86	2.71	1	5.88	13.12	3.75	18.86	15.67	35.33
Solyc10g078360.2	0.02	0.085	2.23	0	0	0	0	0	0	0.03	0	0.05	0.29	0.14	11.96
Solyc06g060690.2	0.009	0.054	2.22	3.16	3.46	2.16	3.21	2.92	2.24	19.25	61.04	108.11	32.43	54.59	11.1
Solyc07g053230.3	0.007	0.048	2.22	0.13	0.44	0	0.45	0.21	0	8.94	22.56	37.97	12.09	10.54	2.6
Solyc01g087850.2	<0.001	0.005	2.21	1.36	3.03	1.49	2.36	2.05	2.45	16.75	18.5	27.88	14.11	14.91	12.45
Solyc01g006320.3	0.005	0.038	2.2	5.22	6.44	4.49	4.43	6.93	4.16	54.75	31.93	96.03	26.57	18.15	27.37
Solyc06g051010.1	<0.001	0.004	2.2	10.5	10.31	9.07	11.22	9.44	7.76	36.95	63.62	80.67	45.83	53.27	52.28
Solyc09g061890.3	0.013	0.069	2.2	0	1.45	0.04	0.06	0.05	0.02	0.23	1.52	1.46	2.8	1.52	9.77

TomatoID	p-value	q-value	DE in Infected	Control1 FPKM	Control2 FPKM	Control3 FPKM	LsoFree1 FPKM	LsoFree2 FPKM	LsoFree3 FPKM	LsoB1 FPKM	LsoB2 FPKM	LsoB3 FPKM	LsoA1 FPKM	LsoA2 FPKM	LsoA3 FPKM
Solyc03g083010.3	0.016	0.075	2.19	9.29	7.88	8.35	8.82	9.75	8.67	10.11	31.08	20.17	63.24	21.06	86.41
Solyc11g072600.2	0.04	0.13	2.19	13.01	11.54	12.77	36.98	16.67	24.17	18.77	30.42	22.04	31.96	63.16	224.66
Solyc03g083720.1	0.001	0.015	2.18	0.3	0.75	0.49	0.47	0.1	0.39	0.94	2.18	3.11	2.73	2.91	8
Solyc06g069380.3	0.036	0.123	2.18	0.97	6.12	2.66	2.58	3.44	0.44	4.19	9.46	4.74	9.54	11.75	35.49
Solyc04g025650.3	0.01	0.058	2.17	13.89	10.12	13.51	13.8	11.98	18.78	31.41	172.22	162.34	136.23	79.82	54.73
Solyc02g088090.1	0.026	0.101	2.16	9.95	4.98	5.9	5.34	10.59	4.2	96.16	123.34	368.29	75.54	137.18	16.97
Solyc04g074440.1	0.039	0.13	2.16	0.47	2.14	0.46	2.1	1.47	2.05	13.31	43.23	74.91	14.93	74.82	5.6
Solyc04g078750.3	0.011	0.063	2.15	5.25	22.02	10.67	9.49	6.1	5.83	13.31	46.03	41.97	44.06	39.97	49.26
Solyc12g094520.2	0.007	0.048	2.15	36.26	30.12	29.47	38.39	41.27	42.05	49.41	187	125.03	142.68	102.87	314.63
Solyc02g071030.2	0.011	0.062	2.14	583.82	765.4	864.03	605.21	249.24	398.96	1989.46	789.04	296.67	1189.46	1107.74	1961.55
Solyc02g082930.3	0.013	0.066	2.14	1.31	6.56	1.28	4.5	10.53	2.15	23.54	26.43	44.75	28.01	21.87	51.47
Solyc03g082660.3	0.006	0.044	2.14	14.75	29.19	23.19	18.72	18.91	11.37	28.93	107.57	77.41	102.91	90.46	119.3

TomatoID	p-value	q-value	DE in Infected	Control1 FPKM	Control2 FPKM	Control3 FPKM	LsoFree1 FPKM	LsoFree2 FPKM	LsoFree3 FPKM	LsoB1 FPKM	LsoB2 FPKM	LsoB3 FPKM	LsoA1 FPKM	LsoA2 FPKM	LsoA3 FPKM
Solyc04g081530.1	0.017	0.077	2.14	22.17	9.71	13.92	16.03	23.63	23.54	25.39	120.54	69.89	106.79	49.31	149.74
Solyc07g052510.4	0.006	0.045	2.13	0	0	0	0.09	0	0.09	0.62	1.23	0.07	2.05	2.77	6.54
Solyc07g064820.1	<0.001	0.004	2.13	0.36	0.51	0.25	0.57	0.4	0.49	3.66	8.96	6.1	8.4	7.63	5.41
Solyc12g037930.2	0.008	0.05	2.13	2.49	8.5	5.19	5.24	4.37	3.18	9.9	9.62	6.86	18.51	19.75	37.61
Solyc03g116070.1	<0.001	0.004	2.12	0	0	0	0.25	0	0	3.19	2.89	1.57	2	2.63	4.72
Solyc04g071400.3	0.02	0.086	2.12	0.16	0.13	0.08	0.11	0.06	0	0.27	3.82	0.19	3.13	3.19	7.51
Solyc06g051620.3	0.003	0.029	2.12	1.14	0.38	0.62	1.28	1.33	0.79	5.2	8.12	13.07	4.08	3.85	12.09
Solyc06g083650.3	0.004	0.034	2.12	0	1.16	0.3	0.04	0.05	0	1.38	0.78	1	2.99	4.29	5.41
Solyc09g065620.3	0.028	0.105	2.12	1.76	8.46	4.79	2.97	1.89	3.27	2.04	4.12	1.86	4.05	8.61	34.18
Solyc12g014420.2	0.022	0.092	2.12	0.88	2.34	1.23	1.86	1.95	0.66	1.9	9.12	6.28	5.45	4.51	25.99

TomatoID	p-value	q-value	DE in Infected	Control1 FPKM	Control2 FPKM	Control3 FPKM	LsoFree1 FPKM	LsoFree2 FPKM	LsoFree3 FPKM	LsoB1 FPKM	LsoB2 FPKM	LsoB3 FPKM	LsoA1 FPKM	LsoA2 FPKM	LsoA3 FPKM
Solyc03g118450.1	0.025	0.1	2.11	0.38	0.94	1.03	2.62	1.01	1.77	3.23	23	29.72	10.21	23.89	8.52
Solyc05g007490.3	0.001	0.02	2.11	6.05	8.21	6.42	5.16	4.84	3.83	9.47	24.42	18.86	24.51	25.17	35.46
Solyc09g083445.1	0.039	0.129	2.11	0	0.76	0	0.6	0.43	0	1.29	0.65	0	4.43	2.24	11.83
Solyc11g010390.1	<0.001	0.008	2.11	18.85	29.18	27.73	23.12	20.89	14.35	94.01	154.43	137.43	126.34	148.78	83.46
Solyc01g099370.3	0.025	0.099	2.1	18.07	13.68	14.5	18.04	20.57	16.86	55.99	403.05	421.01	158.1	308.26	58.43
Solyc05g015840.3	0.016	0.075	2.1	3.41	5.18	2.3	2.17	5.64	1.25	11.85	59.8	67.75	22.47	19.22	24.36
Solyc07g054220.1	<0.001	0.012	2.1	1.97	2.2	1.55	2.45	2.09	2.86	6.04	10.52	12.3	12.23	19.07	14.89
Solyc10g055780.1	0.02	0.086	2.1	0.31	6.82	2.45	0.64	3.64	1.39	23.44	9.65	13.05	19.34	9.65	11.94
Solyc11g039860.2	0.02	0.086	2.1	302.48	572.81	417.43	360.64	191.78	573.25	332.23	497.11	787.86	1691.68	1961.7	1339.53
Solyc01g079600.3	0.038	0.127	2.09	2.34	2.03	2.48	3.51	3.39	4.15	4.08	36.45	60.26	21.28	34.93	18.19
Solyc04g051360.3	0.047	0.144	2.09	0	0	0	0	0	0	0.22	4.05	8.06	0.37	0.57	6.98

TomatoID	p-value	q-value	DE in Infected	Control1 FPKM	Control2 FPKM	Control3 FPKM	LsoFree1 FPKM	LsoFree2 FPKM	LsoFree3 FPKM	LsoB1 FPKM	LsoB2 FPKM	LsoB3 FPKM	LsoA1 FPKM	LsoA2 FPKM	LsoA3 FPKM
Solyc08g082820.3	0.004	0.036	2.09	9.09	14.79	12.19	13.64	9.28	7.16	85.22	47.75	134.6	46.25	38.97	39.27
Solyc03g020040.3	0.022	0.092	2.08	0	0.35	0.08	0	0.35	0	0.42	8.84	2.87	6.02	2.05	7.69
Solyc04g076570.3	0.003	0.032	2.08	4.45	4.94	5.35	4.74	3.49	3.42	6.43	6.37	5.1	12.19	11.76	35.31
Solyc06g053640.1	0.003	0.031	2.08	0.42	0.85	0.19	0.07	0.41	0.21	2.47	10.4	8.08	11.18	10.82	4.04
Solyc06g069070.1	0.039	0.128	2.08	1.79	6.83	2.65	3.05	4.8	0.18	10.36	37.9	8.28	30.93	21.39	28.36
Solyc09g031920.1	0.001	0.016	2.08	7.83	10.81	10.23	11.36	10.8	9.18	20.4	43.32	40.16	29.27	40.59	69.99
Solyc03g116590.3	0.003	0.032	2.07	28.83	34.04	28.63	27.73	34.52	18.83	50.56	153.77	100.91	96.58	112.99	208.98
Solyc05g005160.3	<0.001	0.009	2.07	5.88	13.14	7	9.6	6.55	5.5	26.48	25.31	31.06	29.45	28.63	43.82
Solyc10g008000.1	0.003	0.031	2.07	0	0.26	0.31	0.23	1.97	0	7.11	3.44	11.92	4.86	7.62	7.25
Solyc01g080600.3	0.015	0.074	2.06	1.3	8.06	2.5	1.63	2.57	0.64	15.13	7.01	15.97	23.51	12.81	12.13
Solyc02g069910.2	0.045	0.141	2.06	10.98	22.85	16.34	9.41	4.59	4.01	18.09	92.35	53.56	64.45	80.32	25.76

TomatoID	p-value	q-value	DE in Infected	Control1 FPKM	Control2 FPKM	Control3 FPKM	LsoFree1 FPKM	LsoFree2 FPKM	LsoFree3 FPKM	LsoB1 FPKM	LsoB2 FPKM	LsoB3 FPKM	LsoA1 FPKM	LsoA2 FPKM	LsoA3 FPKM
Solyc06g066600.3	0.003	0.032	2.06	0.25	1.97	0.34	0.31	0.37	0	2.48	2.79	3.34	7.59	5.31	6.13
Solyc07g007870.3	0.004	0.036	2.06	21.09	20.19	17.91	29.49	24.74	23.55	35.85	73.31	63.28	64.48	63.17	195
Solyc08g083370.3	0.043	0.136	2.06	0.7	5.74	4.23	1.84	0.53	0.17	3.61	3.5	0.98	7.32	8.65	10.03
Solyc10g006660.3	0.04	0.13	2.06	8.99	4.61	5.37	4.9	8.76	6.17	49.93	125.54	259.72	67.87	188	14.4
Solyc04g054950.3	0.008	0.05	2.05	1.72	0.71	1.42	0.22	0.47	0.68	6.58	10.74	18.48	6.26	4.51	3.67
Solyc06g049020.1	0.013	0.066	2.05	20.88	24.63	12.71	10.55	29.34	32.84	74.24	170.16	319.39	155.57	214.02	71.69
Solyc07g016215.1	0.018	0.081	2.05	0.06	0.44	0	0.15	0.19	0	1.86	6.81	1.09	13.58	5.47	4.62
Solyc08g075870.3	0.016	0.076	2.05	0.68	2.66	0.65	1.16	1.49	0.34	2.42	7.85	14.51	5.22	5.51	15.09
Solyc12g040570.1	0.004	0.034	2.05	0	1.12	0	0	1.04	0.65	4.07	13.71	16.38	10.31	6.37	5.6
Solyc01g080910.2	0.034	0.118	2.04	16.59	15.13	18.46	20.48	18.2	14.98	30.11	22.29	9.68	61.21	61.6	135.1
Solyc02g087770.3	0.001	0.019	2.04	8.2	7.41	7.15	7.81	7.53	5.88	17.31	18.81	15.72	40.63	25.5	47.12

TomatoID	p-value	q-value	DE in Infected	Control1 FPKM	Control2 FPKM	Control3 FPKM	LsoFree1 FPKM	LsoFree2 FPKM	LsoFree3 FPKM	LsoB1 FPKM	LsoB2 FPKM	LsoB3 FPKM	LsoA1 FPKM	LsoA2 FPKM	LsoA3 FPKM
Solyc05g050560.1	0.005	0.039	2.04	6.97	5.61	6.15	6.42	6.9	8.61	8.4	29.22	18.86	28.6	21.08	50.38
Solyc07g017600.3	0.003	0.032	2.04	5.53	13.78	8.12	11.7	6.37	8.52	14.35	31.1	19.51	27.84	32.82	55.57
Solyc09g008280.2	0.014	0.072	2.04	54.04	139.23	84.33	73.4	62.2	29.57	105.06	273.94	190.1	257.69	278.49	408.37
Solyc09g089930.2	0.012	0.066	2.04	2	4.16	1.8	3.49	5.64	6.15	46.69	51.4	61.78	21.44	12.48	20.5
Solyc01g102390.3	0.016	0.077	2.03	8.34	10.64	4.02	3.56	3.89	2.62	32.34	30.53	63.93	20.4	13.57	19.08
Solyc02g093230.3	0.042	0.135	2.03	1.78	2.05	1.91	0.78	1.03	1	5.65	41.38	39.37	28.29	22.37	4.26
Solyc07g008410.3	0.036	0.122	2.03	0	0.69	0.09	0.22	0.07	0	0.61	0.72	0.08	6.84	3.05	6.33
Solyc01g107390.3	0.006	0.043	2.02	0.07	0.11	0.07	0.07	0.1	0.07	1.33	6.6	11.15	2.92	3.43	3.86
Solyc02g082430.3	0.001	0.015	2.02	4.07	4.32	4.21	6.16	4.2	8.03	10.73	21.14	15.43	19.28	15.82	31.7
Solyc03g112960.1	<0.001	0.003	2.02	2.73	4	2.9	2.44	2.07	2.35	8.44	7.78	13.14	11.21	12.23	13.98

TomatoID	p-value	q-value	DE in Infected	Control1 FPKM	Control2 FPKM	Control3 FPKM	LsoFree1 FPKM	LsoFree2 FPKM	LsoFree3 FPKM	LsoB1 FPKM	LsoB2 FPKM	LsoB3 FPKM	LsoA1 FPKM	LsoA2 FPKM	LsoA3 FPKM
Solyc10g050160.2	0.016	0.077	2.02	2.66	10.29	1.6	3.83	4.02	2.6	9.66	17.5	28.45	13.64	11.56	33.31
Solyc04g072000.3	0.004	0.036	2.01	3.9	4.82	3.75	2.93	3.15	3.13	20.56	46.03	37.57	31.97	11.12	14.21
Solyc06g083050.3	0.013	0.066	2.01	0.4	0.44	0.94	0.27	0	0	1.12	0.9	0	3.73	4.04	4.89
Solyc07g007590.1	0.001	0.016	2.01	6.89	7.2	6.82	5.71	6.03	4.73	21.59	49.17	51.95	47.95	53.19	22.2
Solyc11g010500.1	0.021	0.087	2.01	18.01	22.98	11.85	18.83	22.05	16.62	61.34	392.78	366.48	315.76	301.29	60.92
Solyc12g036470.2	0.001	0.017	2.01	0	0	0.09	0	0.04	0	1.77	2.03	3	0.55	1.37	4.74
Solyc02g082260.3	0.037	0.125	2	0.51	3.75	0.74	1.65	1.05	0.76	2.24	3.9	1.73	9.47	11.6	13.56
Solyc01g102640.2	0.01	0.058	-2	5.78	8.82	2.43	2.14	12.16	2.76	0.72	1.66	1.96	0.4	1.93	1.24
Solyc02g077770.3	0.001	0.017	-2.01	21.17	10.51	16.8	22.26	22.87	33.89	6.94	4.83	3.72	5.21	4.41	4.02

TomatoID	p-value	q-value	DE in Infected	Control1 FPKM	Control2 FPKM	Control3 FPKM	LsoFree1 FPKM	LsoFree2 FPKM	LsoFree3 FPKM	LsoB1 FPKM	LsoB2 FPKM	LsoB3 FPKM	LsoA1 FPKM	LsoA2 FPKM	LsoA3 FPKM
Solyc10g018580.1	0.001	0.017	-2.01	3.85	3.56	4.27	8.59	1.51	4.12	0	0	0	0	0	0
Solyc01g094210.2	0.001	0.015	-2.02	54.31	55.79	56.77	44.62	74.76	39.85	36.54	27.6	21.14	21.71	15.15	10.01
Solyc03g006880.3	0.023	0.094	-2.02	5.49	20.5	8.7	14.77	15.75	6.01	5.36	4.21	0.5	5.04	7.14	2.43
Solyc04g076010.3	0.003	0.029	-2.02	58.91	48.04	50.98	56.49	86.35	62.41	45.51	26.89	38.34	17.2	13.06	12.43
Solyc08g066350.2	0.001	0.017	-2.02	8.46	9.03	11.61	6.8	12.75	16.94	1.57	4.24	2.33	3.21	3.24	1.35
Solyc11g012680.2	0.001	0.016	-2.02	70.26	64.8	54.55	65.63	113.52	75.67	63.86	42	45.93	33.43	25.75	13.87
Solyc03g097930.3	0.001	0.019	-2.03	48.8	26	40.38	24.3	61.88	24.67	13.71	20.13	11.93	15.07	8.94	8.28
Solyc10g051020.2	0.046	0.144	-2.04	65.66	62.73	38.4	24.61	112.78	26.01	46.88	60.42	133.3	14.04	11.3	6.47
Solyc10g051030.2	0.046	0.144	-2.04	5.01	5.28	3.47	3.56	11.79	1.49	3.81	3.8	5.09	6.48	4.53	3.02
Solyc10g084070.1	0.026	0.101	-2.04	12.23	8.92	6.25	1.4	9.7	2.43	1.44	1.34	2.47	2.07	0.41	0.35
Solyc06g076490.3	<0.001	0.003	-2.05	32.45	20.82	27.88	35.21	42.47	38.66	10.85	9.05	12.23	9.22	9.23	7.01
Solyc11g010270.2	<0.001	0.009	-2.05	477.12	373.22	424.87	526.75	614.31	662.42	263.97	202.76	191.73	135.56	196.91	99.07

TomatoID	p-value	q-value	DE in Infected	Control1 FPKM	Control2 FPKM	Control3 FPKM	LsoFree1 FPKM	LsoFree2 FPKM	LsoFree3 FPKM	LsoB1 FPKM	LsoB2 FPKM	LsoB3 FPKM	LsoA1 FPKM	LsoA2 FPKM	LsoA3 FPKM
Solyc12g096490.1	0.007	0.046	-2.05	24.16	11.72	17.66	33.7	14.74	47.31	7.22	4.03	2.29	4.53	4.18	3.31
Solyc05g054850.1	0.004	0.035	-2.06	3.52	4.62	2.1	2.79	6.38	0.51	1.19	0.43	0.15	1.15	0.37	0.26
Solyc06g008760.1	0.003	0.029	-2.06	294.14	170.98	291.13	180.14	142.77	209.83	52.15	51.42	33.59	78.15	68.13	26.39
Solyc10g051120.2	0.006	0.044	-2.06	8.69	3.39	8.88	5.89	4.14	10.28	1.93	0	0	0	0.94	0
Solyc12g056950.2	<0.001	0.012	-2.07	54.3	47.62	62.3	40.39	59.36	50.07	24.79	17.49	10.32	14.75	12.73	8.62
Solyc03g025350.3	0.016	0.076	-2.08	3.17	0.54	0.78	0.51	10.12	0.19	0.47	0.37	0.98	0.35	0.27	0.03
Solyc00g203660.2	0.001	0.015	-2.09	2.51	5.45	9.21	5.5	2.59	8.74	0	0	0	0	0	0
Solyc06g069060.1	0.001	0.019	-2.09	11.16	7.13	11.91	4.82	6.5	9.43	1.94	0.74	0.68	0.1	1.06	0.34
Solyc08g068490.3	0.007	0.048	-2.09	8.26	38.34	19.01	14.86	14.49	22.08	4.27	4.76	1.52	4.5	5.42	2.34
Solyc04g081460.2	<0.001	0.009	-2.11	33.17	40.76	47.81	42.75	33.54	53.75	15.27	12.06	9.07	8.88	10.35	5.63
Solyc07g014620.1	0.006	0.042	-2.11	10.22	4.66	6.99	12.91	21.5	11.66	11.35	5.38	3.75	3.95	2.08	1.93

TomatoID	p-value	q-value	DE in Infected	Control1 FPKM	Control2 FPKM	Control3 FPKM	LsoFree1 FPKM	LsoFree2 FPKM	LsoFree3 FPKM	LsoB1 FPKM	LsoB2 FPKM	LsoB3 FPKM	LsoA1 FPKM	LsoA2 FPKM	LsoA3 FPKM
Solyc07g056280.3	0.011	0.063	-2.11	22.57	10.18	13.09	4.93	28.27	6.06	4.17	4.3	6.65	3.65	2.16	2.26
Solyc09g082980.3	0.005	0.039	-2.11	139.97	115.33	116.21	128.4	169.16	128.91	55.58	32.05	10.53	38.71	43.63	32.06
Solyc10g080670.2	0.024	0.095	-2.11	190.84	71.28	133.45	246.54	215.83	483.9	119.8	73.63	106.54	46.39	47.32	32.81
Solyc02g084930.3	<0.001	0.009	-2.12	4.97	3.91	5.2	4.91	10.5	5.84	2.21	1.49	2.19	0.81	0.71	0.68
Solyc12g036550.2	<0.001	0.001	-2.12	2.58	4.58	2.55	3.98	2.81	3.06	0	0	0.07	0	0	0
Solyc04g007210.3	0.001	0.015	-2.13	57.74	45.65	60.07	56.6	48	75.53	20.09	14.47	7.97	9.7	17.8	8.33
Solyc04g082480.3	0.001	0.018	-2.13	21.03	17.29	18.4	8.87	25.47	10.94	6.51	7.3	6.4	5.04	4.33	2.2
Solyc12g006630.2	<0.001	0.011	-2.13	11.11	17.22	15.31	14.95	24.45	21.13	5.55	4.07	3.31	2.54	6.56	3.19
Solyc06g074800.1	<0.001	0.009	-2.14	11.92	13.13	12	7.18	14.03	10.99	3.94	2.58	1.23	1.88	1.9	1.46
Solyc11g069700.2	0.018	0.08	-2.14	106.61	56.04	45.67	47.19	345.7	22.77	61.93	126.68	141.11	43.22	52.45	24.17
Solyc00g013170.1	<0.001	0.008	-2.15	8.43	7.83	9.6	12.69	9.15	6.24	1.32	2.13	3.89	1.37	2.66	1.08
Solyc01g100490.3	0.006	0.044	-2.15	36.26	41.14	32.66	27	37.17	34.89	31.17	23.36	11.67	15.35	10.68	3.49
Solyc05g010427.1	0.018	0.08	-2.15	2.65	2.15	0.16	2.81	4.94	2.95	0.32	6.95	0.25	0.48	0.29	0.25

TomatoID	p-value	q-value	DE in Infected	Control1 FPKM	Control2 FPKM	Control3 FPKM	LsoFree1 FPKM	LsoFree2 FPKM	LsoFree3 FPKM	LsoB1 FPKM	LsoB2 FPKM	LsoB3 FPKM	LsoA1 FPKM	LsoA2 FPKM	LsoA3 FPKM
Solyc06g061240.3	0.002	0.025	-2.15	105.45	72.75	87.39	117.52	147.94	179.97	69.58	47.01	46.38	26.29	27.71	21.49
Solyc10g080910.1	<0.001	0.006	-2.15	4.33	4.76	5.59	3.99	3.83	3.52	0	0	0	0.93	0.43	0
Solyc08g005305.1	<0.001	0.002	-2.16	7.42	5.4	6.29	5.31	6.65	5.72	0.35	0.56	0.66	0	0.66	0.73
Solyc01g098130.2	<0.001	0.009	-2.17	15.32	14.34	16.19	26.03	13.73	24.63	4.12	3.1	1.83	1.92	1.88	3.3
Solyc06g060830.3	<0.001	0.01	-2.17	8.75	16.48	11.1	19.1	19.16	11.38	6.04	4.65	2.11	3.13	4.56	2.85
Solyc10g054820.2	0.003	0.033	-2.17	44.19	49.6	44.58	25.29	42.26	26.27	17.14	8.22	4.87	14.62	11.38	4.93
Solyc07g014680.3	0.002	0.027	-2.18	7.42	15.97	16.53	22.97	21.58	35.26	5.2	4.12	4.27	2.6	3.22	3.65
Solyc10g080920.2	<0.001	0.007	-2.19	13.12	15.93	16.67	12.52	10.64	17.1	2.16	2.86	2.15	4.49	2.62	1.27
Solyc03g119320.1	<0.001	0.001	-2.2	14.08	10.07	11.62	11.14	13.75	11.68	3.56	2.35	1.81	2.75	2.56	1.46
Solyc07g066330.3	0.042	0.135	-2.2	5.42	2.19	6.07	17.03	7.6	34.44	2.43	1.53	2.1	0.18	1.23	0.89
Solyc00g126000.1	0.001	0.015	-2.22	4.83	5.59	8.66	12.86	10.99	10.27	1.12	1.25	2.4	0.78	0.74	1.7
Solyc03g070430.1	<0.001	0.008	-2.22	3.74	3.02	4.59	4.28	5.8	1.8	0.73	0.73	1.16	0.84	0.02	0.06
Solyc04g080040.3	0.005	0.039	-2.22	42.7	48.24	42.95	19.44	45.32	17.05	18.93	12.67	10.63	10.97	6.65	4.39
Solyc08g074893.1	<0.001	0.006	-2.22	3.57	3.48	2.74	5.19	5.85	8.11	0.7	0.72	0.55	0.77	0.97	0.2

TomatoID	p-value	q-value	DE in Infected	Control1 FPKM	Control2 FPKM	Control3 FPKM	LsoFree1 FPKM	LsoFree2 FPKM	LsoFree3 FPKM	LsoB1 FPKM	LsoB2 FPKM	LsoB3 FPKM	LsoA1 FPKM	LsoA2 FPKM	LsoA3 FPKM
Solyc09g090360.3	0.005	0.04	-2.22	3.83	4.83	5.77	6.02	11.87	1	1.95	2.89	1.34	6.48	1.53	0.47
Solyc03g058390.2	<0.001	<0.001	-2.23	34.02	35.4	35.69	45.23	37.18	42.36	6.42	6.94	8.21	8.33	8.18	7.66
Solyc03g119910.3	0.027	0.104	-2.23	17.19	9	10.66	15.79	17.02	4.31	6.64	10.15	0.85	15.11	4.98	1.43
Solyc02g011830.1	<0.001	0.001	-2.24	4.6	5.35	6.16	4.94	3.14	3.12	0	0	0	0	0	0
Solyc08g081700.1	0.011	0.06	-2.25	27.44	15.98	21.52	13.92	34.96	12.55	19.79	11.25	14.25	6.52	3.53	2.27
Solyc00g126010.1	<0.001	0.009	-2.27	3.93	8.32	7.99	10.46	5.88	10.3	1.27	0.72	1.14	0.95	1.16	0.37
Solyc10g005080.3	0.01	0.058	-2.27	17.08	20.96	15.12	9.5	31.16	9.86	10.77	12.3	12.39	3.81	2.16	2.45
Solyc11g021310.1	<0.001	<0.001	-2.27	4.38	5.97	5.4	5.11	4.03	3.35	0.06	0	0.03	0	0.13	0.18
Solyc05g023730.1	<0.001	0.004	-2.28	63.6	77.45	62.78	62.45	29.46	79.8	5.6	6.72	6.12	7.98	8.35	8.64
Solyc08g075740.1	0.001	0.018	-2.28	16.75	18.45	20.93	23	18.39	26.43	9.02	4.45	6.18	3.88	2	2.31
Solyc05g049990.3	<0.001	0.014	-2.29	46.29	54.79	62.94	30.17	65.37	34.11	18.83	18.05	15.46	16.33	10.29	6.49
Solyc11g066510.2	0.001	0.019	-2.29	12.97	12.44	22.13	12.45	12.1	17.05	5.8	1.71	1.68	2	2.05	0.84
Solyc12g005750.1	<0.001	0.007	-2.29	5.23	5.02	2.16	2.09	4.8	2.42	0.24	0.37	0.05	0.05	0	0

TomatoID	p-value	q-value	DE in Infected	Control1 FPKM	Control2 FPKM	Control3 FPKM	LsoFree1 FPKM	LsoFree2 FPKM	LsoFree3 FPKM	LsoB1 FPKM	LsoB2 FPKM	LsoB3 FPKM	LsoA1 FPKM	LsoA2 FPKM	LsoA3 FPKM
Solyc01g110360.3	0.001	0.021	-2.3	2150.17	1563.89	1983.19	1170.96	1293.94	882.98	542.15	488.38	407.35	360.65	254.2	193.25
Solyc04g058150.3	0.003	0.029	-2.31	781.51	698.98	725.49	444.11	775.09	329.55	206.32	123.67	65.72	218.91	138.05	111.17
Solyc11g007860.1	<0.001	0.001	-2.31	57.25	49.61	52.08	60.61	70.32	63.34	19.31	15.25	14.98	12.03	11.61	10.93
Solyc04g006950.3	0.034	0.119	-2.32	25.64	7.35	23.45	4.23	9.69	2.73	0.29	1.19	0	1.97	2.28	0.42
Solyc08g075880.3	0.001	0.022	-2.32	15.84	13.45	9.39	25.94	21.32	17.5	2.42	2.35	0.98	4.08	4.37	4.48
Solyc07g005210.3	0.001	0.02	-2.33	208.69	149.62	142.87	84.98	217.78	88.26	71.66	55.77	45.13	34.33	38.69	22.02
Solyc11g006290.2	0.002	0.023	-2.33	5.11	6.87	7.65	1.97	8.52	2.81	1.12	0.3	0.47	0.36	0.17	0.05
Solyc12g036793.1	0.043	0.137	-2.34	18.75	3.83	10.75	4.43	31.2	4.58	6.94	0.55	7.35	3.28	2.77	0.76
Solyc12g096130.2	0.001	0.018	-2.34	12.9	16.03	14.46	13.5	18.85	20.06	6.29	4.06	5.7	1.37	2.04	1.87
Solyc01g007810.1	0.006	0.042	-2.37	10.57	28.84	16.9	27.06	14.81	28.92	8.18	2.38	0.85	3.43	2.73	2.32
Solyc02g080510.1	0.007	0.047	-2.39	38.87	37.55	56.63	17.64	75.4	17.23	19.54	12.89	6.01	8.43	9.13	5.29
Solyc05g053850.3	0.045	0.141	-2.39	48.39	58.63	43.38	6.73	46.32	14.46	14.07	10.83	6.44	19.36	4.94	2.22
Solyc11g005290.1	0.007	0.046	-2.4	6.89	6.97	4.28	19.88	16.37	15.2	3.17	5.06	0.46	4.96	2.31	2.42
Solyc01g112190.3	0.001	0.017	-2.41	13.46	7.48	15.53	5.91	11.75	4.88	1.56	2.21	1.5	1.68	1.93	0.4

TomatoID	p-value	q-value	DE in Infected	Control1 FPKM	Control2 FPKM	Control3 FPKM	LsoFree1 FPKM	LsoFree2 FPKM	LsoFree3 FPKM	LsoB1 FPKM	LsoB2 FPKM	LsoB3 FPKM	LsoA1 FPKM	LsoA2 FPKM	LsoA3 FPKM
Solyc07g006600.1	0.019	0.084	-2.42	16.21	18.35	15.29	29.24	20.18	14.86	8.7	11.7	0.47	13.35	4.65	2.47
Solyc07g045530.2	0.045	0.142	-2.42	21.03	6.56	11.39	5.24	15.48	7.69	6.31	2.7	14.84	1.43	0.4	0.3
Solyc10g079810.1	0.011	0.062	-2.43	8.72	11.42	13.78	10.77	16.93	5.97	7.52	1.99	11.79	3.16	1.41	0.57
Solyc03g045070.1	0.015	0.073	-2.44	65.51	66.48	70.99	20.6	65.31	17.7	16.12	7.32	3.46	12.81	11.61	5.4
Solyc03g065050.1	<0.001	0.005	-2.46	7.31	4.33	4.3	9.12	4.63	6.41	0	0.71	0.63	0.85	0.46	0.22
Solyc05g007880.3	0.001	0.016	-2.46	8.68	9.84	5.43	6.49	22.89	7.18	3.73	6.04	4.46	1.8	1.13	1.6
Solyc09g065390.1	<0.001	0.012	-2.47	16.83	10.56	17.78	18.48	23.16	27.18	5.55	3.08	2.12	1.56	2.82	2.39
Solyc12g088670.2	<0.001	0.004	-2.47	1341.04	772.98	1090.05	987.84	1550.13	1658.19	354.87	277.71	269.35	265.9	276.28	185.35
Solyc12g099200.2	0.001	0.015	-2.47	107.29	138.87	116.44	191.21	145.49	240.23	73.3	40.88	38.63	44.3	34.61	18.63
Solyc11g008530.2	0.005	0.039	-2.48	24.89	27.78	15.82	14.8	43.91	8.7	15.8	17.62	23.39	5.9	7.31	2.34
Solyc12g036415.1	0.017	0.079	-2.49	6.46	10.68	8.05	5.83	5.97	11.82	0	0	0	0	6.36	0
Solyc01g049685.1	<0.001	<0.001	-2.51	0	0	0	0	0	0	37.03	35.09	39.4	30.4	33.71	28
Solyc01g049690.3	<0.001	<0.001	-2.51	43.05	32.63	32.05	45.37	42.95	52.57	0.33	0.24	0.2	0.34	0.23	0.19

TomatoID	p-value	q-value	DE in Infected	Control1 FPKM	Control2 FPKM	Control3 FPKM	LsoFree1 FPKM	LsoFree2 FPKM	LsoFree3 FPKM	LsoB1 FPKM	LsoB2 FPKM	LsoB3 FPKM	LsoA1 FPKM	LsoA2 FPKM	LsoA3 FPKM
Solyc01g080870.3	0.004	0.036	-2.51	8.73	18.31	8.85	5.71	14.34	3.67	3.55	5.04	1.55	6.02	1.82	0.45
Solyc10g053880.1	<0.001	0.004	-2.52	9.65	8.05	8.52	5.87	6.21	6.14	0	0.46	1.03	0	0	0.39
Solyc04g011495.1	0.033	0.116	-2.53	13.6	14.28	12.91	13.86	13.72	14.88	0	8.83	0	0.34	0	4.41
Solyc07g063420.3	<0.001	0.014	-2.53	12.86	7.48	12.37	8.42	9.29	20.05	1.64	1.37	2.33	0.6	1.27	0.32
Solyc01g081060.3	<0.001	0.014	-2.54	300.86	250.69	348.98	305.91	367.2	398.98	152.47	98.65	114.22	73.45	57.56	37.48
Solyc11g021130.1	<0.001	0.001	-2.54	72.9	71.04	111.18	94.14	53.84	113.05	8.97	11.58	8.8	10.27	11.01	10.45
Solyc01g110580.2	0.001	0.017	-2.55	26.96	41.13	24.09	32.71	22.28	18.39	10.17	5.12	2.08	5.37	3.4	3.02
Solyc01g109800.2	<0.001	0.006	-2.56	17.41	16.44	7.75	22.95	34.54	13.49	6.21	6.27	2.83	4.82	6.26	3.74
Solyc02g065220.3	0.002	0.027	-2.56	64.79	63.49	67.53	25.85	68.32	35.12	24.52	10.88	10.58	11.44	9.76	4.6
Solyc11g012690.2	0.002	0.026	-2.56	40.36	30.17	48.01	25.38	24.35	15.27	7.88	4.86	3.29	9.29	4.9	1.96
Solyc04g051470.1	0.002	0.026	-2.57	15.45	17.25	17.94	27.51	29.93	28.13	18.93	11.78	8.2	14.69	10.44	1.19
Solyc08g079430.3	<0.001	0.005	-2.59	29.84	11.91	22.13	17.1	27.97	17.11	4.67	4.82	3.42	3.9	4.37	2.04
Solyc04g071780.3	0.005	0.042	-2.6	95.75	22.71	52.63	41.73	29.66	42.22	6.4	7.8	16.32	3.95	9.35	2.94

TomatoID	p-value	q-value	DE in Infected	Control1 FPKM	Control2 FPKM	Control3 FPKM	LsoFree1 FPKM	LsoFree2 FPKM	LsoFree3 FPKM	LsoB1 FPKM	LsoB2 FPKM	LsoB3 FPKM	LsoA1 FPKM	LsoA2 FPKM	LsoA3 FPKM
Solyc01g080460.3	0.001	0.018	-2.61	228.6	160.9	216.76	74.37	242.7	106.64	43.64	39.76	26.99	40.92	25.79	20.36
Solyc07g065410.1	0.019	0.083	-2.61	26.44	24.27	16.87	8.3	115.41	3.76	11.82	9.66	12.61	10.06	6.8	4.69
Solyc12g087870.2	0.002	0.027	-2.62	50.04	18.54	41.8	32.14	59.58	51.47	17.61	21.11	21.25	15.5	9.33	3.18
Solyc05g012030.1	0.002	0.022	-2.63	6.02	12.75	5.27	9.57	20.01	9.34	4.31	2.3	0.4	3.02	0.92	1.53
Solyc01g104910.3	<0.001	0.003	-2.64	50.48	41.54	47.44	35.91	82.04	36.17	14.87	9.48	8.7	9.99	9.98	6.93
Solyc01g107460.2	0.026	0.101	-2.64	270.17	228.54	325.74	70.38	198.61	66.43	119.89	48.02	31.23	32.39	27.23	11.59
Solyc04g011670.3	<0.001	0.004	-2.64	47.04	24.42	41.64	36.49	48.17	40.94	8.79	12.78	7.79	9.56	9.38	4.2
Solyc05g013530.3	<0.001	0.001	-2.65	166.72	139.1	149.14	113.63	157.26	148.17	35.03	25.12	21.67	27.09	31.67	16.86
Solyc05g041360.2	<0.001	0.004	-2.65	11.51	12.47	9.11	7.6	4.47	6.19	0.31	0.07	0.25	0.2	0.39	0
Solyc08g074490.3	<0.001	0.005	-2.65	45.69	28.89	35.61	27.25	55.82	43.8	11.73	11.27	11.78	10.73	8.88	3.76
Solyc09g061390.1	<0.001	0.008	-2.66	13.2	16.47	16.02	12.77	3.94	7.79	0.05	0.05	0.24	0.29	0.35	0.21
Solyc11g073120.2	<0.001	0.013	-2.66	149.28	113.53	145.41	150.26	167.34	154.07	77.92	61.4	43.27	45.08	29.98	14.09
Solyc10g078570.1	<0.001	0.001	-2.67	29.25	27.12	32.7	35.94	35.96	41.98	8.01	6.06	6.71	4.2	5.35	3.64

TomatoID	p-value	q-value	DE in Infected	Control1 FPKM	Control2 FPKM	Control3 FPKM	LsoFree1 FPKM	LsoFree2 FPKM	LsoFree3 FPKM	LsoB1 FPKM	LsoB2 FPKM	LsoB3 FPKM	LsoA1 FPKM	LsoA2 FPKM	LsoA3 FPKM
Solyc01g066260.2	<0.001	0.012	-2.71	14.44	8.83	13.12	6.69	16.89	11.74	2.93	0.6	1.76	1.4	1.76	0.38
Solyc06g005330.3	0.024	0.096	-2.71	67.03	46.94	103.55	80.75	25.08	33.66	31.65	10.58	3.19	12.24	7.32	2.07
Solyc08g075570.3	0.028	0.106	-2.72	26.75	3.51	17.58	1.64	13.34	1.62	0.19	0.21	0.15	0.12	0.07	0.05
Solyc05g047440.1	<0.001	<0.001	-2.75	5.98	9.02	6.37	7.49	5.74	6.28	0.15	0	0	0	0.39	0.1
Solyc03g115920.3	0.011	0.062	-2.77	48.47	46.05	24.74	17.71	101.13	11.03	21.16	12.22	42.67	11.24	7.16	4.23
Solyc06g068500.3	0.012	0.066	-2.77	52.23	32.41	38.72	153.87	99.11	240.9	17.58	10.36	5.21	12.96	16.56	15.27
Solyc01g014250.2	<0.001	0.001	-2.78	8.86	8.09	11.76	13.23	10.87	13.52	1.73	1.49	1.68	0.97	1.01	0.4
Solyc05g054750.3	0.002	0.026	-2.78	2.79	9.35	5.02	9.63	9.09	17.25	2.42	1.9	1.36	0.33	0.47	0.18
Solyc08g068850.3	0.018	0.081	-2.78	4.12	4.31	2.43	2.08	30.18	0.75	2.45	5.46	13.63	1.49	0.43	0.32
Solyc11g069450.1	0.001	0.018	-2.8	57.71	54.74	51.83	109.77	120.78	135.04	33.62	18.77	11.03	10.63	11.7	13.74
Solyc11g032190.1	<0.001	0.001	-2.84	5.42	7.34	6.77	9.88	10.27	6.24	0.99	0.74	1.05	2.28	1.16	0.12
Solyc12g008380.1	<0.001	0.007	-2.86	14.07	14.01	12.81	8.62	25.77	13.59	4.67	2.18	1.84	2.28	1.11	1.04
Solyc10g047090.2	<0.001	0.002	-2.87	48.67	56.46	38.68	34.68	16.73	52.49	1.56	1.99	2.65	1.58	1.95	3.13
Solyc11g069940.1	0.006	0.043	-2.87	62.35	36.76	69.05	18.3	111.04	16.83	13.98	12.4	17.15	9.16	5.44	4.72

TomatoID	p-value	q-value	DE in Infected	Control1 FPKM	Control2 FPKM	Control3 FPKM	LsoFree1 FPKM	LsoFree2 FPKM	LsoFree3 FPKM	LsoB1 FPKM	LsoB2 FPKM	LsoB3 FPKM	LsoA1 FPKM	LsoA2 FPKM	LsoA3 FPKM
Solyc05g005870.3	0.001	0.015	-2.88	52.76	50.93	62.25	38.49	48.53	44.42	17.19	14.59	9.64	14.33	10.64	2.44
Solyc10g047390.1	<0.001	<0.001	-2.92	8	9.67	7.25	10.69	4.49	7.66	0	0	0	0	0	0
Solyc09g008200.3	<0.001	0.006	-2.95	207.91	112.49	192.47	152.68	137.55	158.8	38.58	21.24	23.04	15.4	22.57	12.19
Solyc11g021240.2	<0.001	<0.001	-2.95	7.34	7.39	8.85	8.99	6.02	6.07	0	0	0	0.25	0	0
Solyc05g026460.1	<0.001	0.005	-2.96	7.66	8.36	6.12	9.16	14.34	17.3	1.86	0.97	1.65	0.86	0.41	0.45
Solyc06g005320.1	0.015	0.073	-3.02	33.67	15.71	47.23	25.02	11.81	13.48	7.27	8.5	0.74	4.34	2.1	0.07
Solyc06g066530.1	<0.001	0.004	-3.14	31.65	36.73	22.83	19.54	23.61	41.01	4.22	3.35	2.08	2.22	1.58	1.48
Solyc11g056500.2	<0.001	<0.001	-3.21	7.06	10.67	6.96	10.63	10.63	10.05	0	0.13	0.55	0.4	0.64	0.25
Solyc12g035550.1	<0.001	0.001	-3.21	5.54	10.53	11.46	10.66	7.59	5.54	0	0	0	0	0	0
Solyc08g079230.1	0.036	0.124	-3.32	311.12	18	139.8	17.46	58.1	17.48	3.2	11.13	6.3	8.62	2.21	1.92
Solyc09g009130.3	0.001	0.017	-3.32	396.93	270.5	443.73	237.28	313.96	296.44	96.62	37.68	63.02	29.51	21.55	17.47
Solyc08g016000.1	<0.001	<0.001	-3.35	11.93	12.8	12.94	13.4	7.24	9.64	0	0.06	0.09	0	0	0

TomatoID	p-value	q-value	DE in Infected	Control1 FPKM	Control2 FPKM	Control3 FPKM	LsoFree1 FPKM	LsoFree2 FPKM	LsoFree3 FPKM	LsoB1 FPKM	LsoB2 FPKM	LsoB3 FPKM	LsoA1 FPKM	LsoA2 FPKM	LsoA3 FPKM
Solyc07g006310.1	0.002	0.023	-3.43	4.58	23.54	9.78	24.45	20.59	4.37	2.14	0.18	0.27	1.52	0.96	0.9
Solyc09g010530.3	0.002	0.025	-3.57	34.28	31.15	31.49	33.03	39.7	7.66	10.78	7.66	2.8	14.16	3.14	0.69
Solyc11g021300.1	<0.001	<0.001	-3.62	12.35	14.68	13.18	13.11	10	11.87	0	0.01	0.06	0	0	0
Solyc11g021260.1	<0.001	<0.001	-3.66	12.91	13.87	13.24	16.27	9.47	10.61	0	0	0	0	0	0
Solyc06g005310.3	0.004	0.034	-3.7	62.16	27.64	78.02	44.4	21.8	26.6	9.43	6.58	1.32	4.61	2.22	0.13
Solyc01g086660.2	0.003	0.029	-3.72	49.61	54.34	17.05	16.95	62.95	7.07	6.29	12.99	20.62	8.59	2.92	1.06
Solyc11g021290.2	<0.001	<0.001	-3.91	14.81	17.45	18.5	21.92	11.29	12.92	0	0	0.05	0	0.09	0
Solyc08g065610.3	<0.001	0.008	-4.03	882.3	279.33	589.81	299.44	745.37	1105.08	48.57	46.75	48.75	42.42	43.16	22.91
Solyc10g049470.1	<0.001	<0.001	-4.08	18.78	19.52	21.34	24.45	12.53	15.32	0	0	0	0	0	0
Solyc01g109090.2	0.001	0.016	-4.12	167.44	74.65	145.01	358.66	313.83	493.43	40.43	24.95	42.31	18.38	12.44	10.64
Solyc11g021280.1	<0.001	<0.001	-4.23	22.2	19.6	28.99	31.15	14.04	17.95	0	0	0.2	0	0	0

TomatoID	p-value	q-value	DE in Infected	Control1 FPKM	Control2 FPKM	Control3 FPKM	LsoFree1 FPKM	LsoFree2 FPKM	LsoFree3 FPKM	LsoB1 FPKM	LsoB2 FPKM	LsoB3 FPKM	LsoA1 FPKM	LsoA2 FPKM	LsoA3 FPKM
Solyc11g021270.1	<0.001	<0.001	-4.71	32.56	29.46	41.88	53.3	17.16	23.2	0	0	0	0	0	0
Solyc04g054745.1	<0.001	0.001	-4.97	43.73	49.55	61.38	27.1	32.09	13.42	0	0	0	0	0	0
Solyc09g065790.1	<0.001	<0.001	-5.14	113.64	99.57	153.11	235.36	84.75	184.47	1.01	1.45	2.79	2.36	2.34	2.67
Solyc09g064580.2	<0.001	<0.001	-5.99	46.87	67.59	82.83	84.15	56.09	105.86	0	0	0	0	0	0
Solyc01g017220.1	<0.001	<0.001	-6.05	1691.72	2005.67	2520.6	2479.06	865.06	1715.53	13.46	11.51	9.5	17.54	16.97	21.32
Solyc04g039850.1	<0.001	<0.001	-6.4	581.67	761.08	842.23	686.51	284.76	577.12	2.29	2.59	1.72	3.75	3.02	4.86
Solyc06g007515.1	0.012	0.066	-6.81	63.2	0	0.08	171.68	139.06	0	0	0	0	0	0	0

TomatoID	NCBI Protein Name	Gene ID	*A. Thaliana* Homolog	Uniprot Description	Putative Consequences for Infection	Citation
Solyc03g098790.3	cathepsin D inhibitor protein precursor	cathDInh	N/A	Endopeptidase inhibitor activity	N/A	N/A
Solyc00g068980.2	N/A	N/A	N/A	N/A	N/A	N/A
Solyc12g010020.2	leucine aminopeptidase 1, chloroplastic	LAPA1	AT2G24200	Involved in the processing and regular turnover of intracellular proteins	Increased intracellular protein turnover	Pautot, Veronique, et al. "Leucine aminopeptidase: an inducible component of the defense response in Lycopersicon esculentum (tomato)." Proceedings of the National Academy of Sciences 90.21 (1993): 9906-9910.
Solyc00g187050.3	leucine aminopeptidase 1, chloroplastic	LAPA1	AT2G24200	Involved in the processing and regular turnover of intracellular proteins	Increased intracellular protein turnover	Pautot, Veronique, et al. "Leucine aminopeptidase: an inducible component of the defense response in Lycopersicon esculentum (tomato)." Proceedings of the National Academy of Sciences 90.21 (1993): 9906-9910.
Solyc12g010025.1	N/A	N/A	N/A	N/A	N/A	N/A
Solyc12g010030.2	leucine aminopeptidase 2, chloroplastic isoform X1	LAP2	AT4G30920	Involved in the processing and regular turnover of intracellular proteins; Heat shock protein	Increased turnover of intracellular proteins; Increased protection from heat-induced damaged	Waditee-Sirisattha, Rungaroon, et al. "The Arabidopsis aminopeptidase LAP2 regulates plant growth, leaf longevity and stress response." New Phytologist 191.4 (2011): 958-969.
Solyc09g089530.3	proteinase inhibitor 1-like	N/A	N/A	N/A	N/A	N/A
Solyc09g084470.3	wound-induced proteinase inhibitor 1 precursor	N/A	N/A	Inhibits both chymotrypsin and trypsin, supressing protein digestion by herbivores	Increased defense against herbivory	Cleveland, Thomas E., Robert W. Thornburg, and Clarence A. Ryan. "Molecular characterization of a wound-inducible inhibitor I gene from potato and the processing of its mRNA and protein." Plant molecular biology 8.3 (1987): 199-207.
Solyc09g084480.3	wound-induced proteinase inhibitor 1-like	N/A	N/A	Inhibits both chymotrypsin and trypsin, supressing protein digestion by herbivores	Increased defense against herbivory	Cleveland, Thomas E., Robert W. Thornburg, and Clarence A. Ryan. "Molecular characterization of a wound-inducible inhibitor I gene from potato and the processing of its mRNA and protein." Plant molecular biology 8.3 (1987): 199-207.
Solyc09g084490.3	wound-induced proteinase inhibitor 1-like	N/A	N/A	Inhibits both chymotrypsin and trypsin, supressing protein digestion by herbivores	Increased defense against herbivory	Cleveland, Thomas E., Robert W. Thornburg, and Clarence A. Ryan. "Molecular characterization of a wound-inducible inhibitor I gene from potato and the processing of its mRNA and protein." Plant molecular biology 8.3 (1987): 199-207.
Solyc08g074630.2	polyphenol oxidase F, chloroplastic	N/A	N/A	Catalyzes the oxidation of mono- and o-diphenols to o-diquinones	N/A	Newman, Sally M., et al. "Organisation of the tomato polyphenol oxidase gene family." Plant molecular biology 21.6 (1993): 1035-1051.
Solyc01g095140.3	ethylene-responsive late embryogenesis-like protein	ER5	N/A	Involved in response to desiccation	Increased response to drought	Zegzouti, Hicham, et al. "ER5, a tomato cDNA encoding an ethylene-responsive LEA-like protein: characterization and expression in response to drought, ABA and wounding." Plant molecular biology 35.6 (1997): 847-854.

TomatoID	NCBI Protein Name	Gene ID	*A. Thaliana* Homolog	Uniprot Description	Putative Consequences for Infection	Citation
Solyc03g098780.2	aspartic protease inhibitor 1	N/A	N/A	Inhibitor of cathepsin D; Protects the plant by inhibiting proteases of invading organisms	Increased defense against herbivory	Ishikawa, Atsushi, et al. "A family of potato genes that encode Kunitz-type proteinase inhibitors: structural comparisons and differential expression." Plant and cell physiology 35.2 (1994): 303-312.
Solyc09g083440.3	wound-induced proteinase inhibitor 1	N/A	N/A	Inhibits both chymotrypsin and trypsin, supressing protein digestion by herbivores	Increased defense against herbivory	Cleveland, Thomas E., Robert W. Thornburg, and Clarence A. Ryan. "Molecular characterization of a wound-inducible inhibitor I gene from potato and the processing of its mRNA and protein." Plant molecular biology 8.3 (1987): 199-207.
Solyc06g062370.3	Glucose-1-phosphate adenylyltransferase small subunit, chloroplastic	APS1	AT5G48300	Plays a role in starch synthesis; Catalyzes the synthesis of the activated glycosyl donor, ADP-glucose from Glc-1-P, and ATP	Increased starch synthesis	Tanaka, Hiroshi, et al. "Isolation and some properties of acid phosphatase-11 from tomato leaves." Agricultural and biological chemistry 54.8 (1990): 1947-1952.
Solyc09g084450.3	transcription factor bHLH92 isoform X1	BHLH92	AT5G43650	Involved in regulation of transcription	N/A	Jiang, Yuanqing, Bo Yang, and Michael K. Deyholos. "Functional characterization of the Arabidopsis bHLH92 transcription factor in abiotic stress." Molecular Genetics and Genomics 282.5 (2009): 503-516.
Solyc09g089520.3	proteinase inhibitor I-B-like	LOC107794480	N/A	Involved in response to wounding	Promoted response to wounding	Sierro, Nicolas, et al. "The tobacco genome sequence and its comparison with those of tomato and potato." Nature communications 5.1 (2014): 1-9.
Solyc09g008670.3	threonine deaminase	N/A	N/A	Involved in cellular amino acid biosynthetic process	N/A	Tieman, Denise M., and Avtar K. Handa. "Molecular cloning and characterization of genes expressed during early tomato (Lycopersicon esculentum Mill.) fruit development by mRNA differential display." Journal of the American Society for Horticultural Science 121.1 (1996): 52-56.
Solyc12g096780.2	enoyl-[acyl-carrier-protein] reductase, mitochondrial-like	AT3G45770	AT3G45770	Catalyzes the NADPH-dependent reduction of trans-2-enoyl thioesters in mitochondrial fatty acid synthesis	Increased fatty acid synthesis and metabolism	Li-Beisson, Yonghua, et al. "Acyl-lipid metabolism." The Arabidopsis book/American Society of Plant Biologists 11 (2013).
Solyc07g007250.3	metallocarboxypeptidase inhibitor IIa precursor	N/A	N/A	Carboxypeptidase and endopeptidase inhibitor activity	N/A	N/A
Solyc09g089505.1	N/A	N/A	N/A	N/A	N/A	N/A
Solyc09g089510.3	proteinase inhibitor I-B	LOC107794480	N/A	Involved in response to wounding	Promoted response to wounding	Sierro, Nicolas, et al. "The tobacco genome sequence and its comparison with those of tomato and potato." Nature communications 5.1 (2014): 1-9.
Solyc11g027770.1	N/A	N/A	N/A	N/A	N/A	N/A
Solyc07g008570.3	probable inactive purple acid phosphatase 27	PAP27	AT5G50400	Acid phosphatase activity; Metal ion binding	N/A	Cheng, Chia-Yi, et al. "Araport11: a complete reannotation of the Arabidopsis thaliana reference genome." The Plant Journal 89.4 (2017): 789-804.

TomatoID	NCBI Protein Name	Gene ID	*A. Thaliana* Homolog	Uniprot Description	Putative Consequences for Infection	Citation
Solyc11g021060.2	proteinase inhibitor type-2 TR8 precursor	ARPI	N/A	Serine-type endopeptidase inhibitor activity	N/A	Taylor, Brian H., Roger J. Young, and Chantel F. Scheuring. "Induction of a proteinase inhibitor II-class gene by auxin in tomato roots." Plant molecular biology 23.5 (1993): 1005-1014.
Solyc01g091170.3	Arginase 2, chloroplastic/mitochondrial	ARGAH2	AT4G08870	Utilized in the urea cycle; Precursor for the synthesis of both polyamines and proline; Catalyzes the formation of putrescine from agmatine	Promoted jasmonate-related functions; Increased urea waste management; Increased freezing tolerance	Dombrecht, Bruno, et al. "MYC2 differentially modulates diverse jasmonate-dependent functions in Arabidopsis." The Plant Cell 19.7 (2007): 2225-2245.
Solyc01g017490.1	N/A	N/A	N/A	N/A	N/A	N/A
Solyc06g083900.3	transcription factor MYB13-like	MYB13	AT1G06180	Component of a regulatory network controlling the establishment and/or development of the shoot system by the regulation of apical meristem function	Increased establishment and/or development of the shoot system by the regulation of apical meristem function	Kirik, Victor, et al. "Ectopic expression of a novel MYB gene modifies the architecture of the Arabidopsis inflorescence." The Plant Journal 13.6 (1998): 729-742.
Solyc07g064600.3	inducible plastid-lipid associated protein	CHRD	N/A	Involved in organonitrogen compound catabolic process	N/A	N/A
Solyc12g010980.2	acetyl-CoA-benzylalcohol acetyltransferase	BEAT	N/A	Involved in the biosynthesis of benzyl acetate, a major constituent of the floral scent	Increased synthesis of benzyl acetate	Dudareva, Natalia, et al. "Acetyl-CoA: benzylalcohol acetyltransferase–an enzyme involved in floral scent production in Clarkia breweri." The Plant Journal 14.3 (1998): 297-304.
Solyc11g051200.1	N/A	N/A	N/A	N/A	N/A	N/A
Solyc11g071480.1	agmatine hydroxycinnamoyltransferase 1-like	AHT1	AT3G61600	Promotes the synthesis of hydroxycinnamic acid amides, which play a role in defense against pathogens	Increased defense against pathogens	Chen, Wei, et al. "Genome-wide association analyses provide genetic and biochemical insights into natural variation in rice metabolism." Nature genetics 46.7 (2014): 714-721.
Solyc01g006400.3	Hop-interacting protein THI101 precursor	N/A	N/A	Transcriptional activator	N/A	N/A
Solyc12g009220.2	jasmonate ZIM-domain protein 2	NtJAZ2	N/A	Involved in regulation of defense response; Involved in regulation of JA-mediated signaling pathway; Involved in response to wounding	Increased regulation of defense response; Increased regulation of JA-mediated signaling; Increased response to wounding	Shoji, Tsubasa, Takayuki Ogawa, and Takashi Hashimoto. "Jasmonate-induced nicotine formation in tobacco is mediated by tobacco COI1 and JAZ genes." Plant and cell physiology 49.7 (2008): 1003-1012.
Solyc01g017050.1	N/A	N/A	N/A	N/A	N/A	N/A
Solyc11g022590.1	uncharacterized protein LOC544001 precursor	N/A	N/A	N/A	N/A	N/A

TomatoID	NCBI Protein Name	Gene ID	*A. Thaliana* Homolog	Uniprot Description	Putative Consequences for Infection	Citation
Solyc06g076020.3	heat shock cognate 70 kDa protein 1	HSP70-1	AT5G02500	Facilitates folding of de novo proteins, assists translocation of precursor proteins into organelles; Degrades damaged protein under stress conditions; Involved in defense response; Involved in protein transport to chloroplasts; Modulates stomatal aperture in response to environmental conditions	Increased protein folding; Increased translocation of presursor proteins into organelles; Increased degradation of damaged proteins; Promoted defense response; Increased response to abiotic stress and ABA signaling	Sung, Dong Yul, and Charles L. Guy. "Physiological and molecular assessment of altered expression of Hsc70-1 in Arabidopsis. Evidence for pleiotropic consequences." Plant Physiology 132.2 (2003): 979-987.
Solyc02g081980.3	apyrase isoform X1	RROP1	N/A	Catalyzes the hydrolysis of phosphoanhydride bonds of nucleoside tri- and di-phosphates	N/A	Handa, Masahisa, and Guido Guidotti. "Purification and cloning of a soluble ATP-diphosphohydrolase (apyrase) from potato tubers (Solanum tuberosum)." Biochemical and biophysical research communications 218.3 (1996): 916-923.
Solyc01g060020.3	glucan endo-1,3-beta-glucosidase B precursor	N/A	N/A	Involved in plant defense against pathogens	Increased defense against pathogens	van Kan, Jan AL, et al. "Differential accumulation of mRNAs encoding extracellular and intracellular PR proteins in tomato induced by virulent and avirulent races of Cladosporium fulvum." Plant molecular biology 20.3 (1992): 513-527.
Solyc10g005320.3	tryptophan synthase beta chain 1-like	TSB1	AT5G54810	The beta subunit is responsible for the synthesis of L-tryptophan from indole and L-serine	Increased synthesis of L-tryptophan from indole and L-serine	Ouyang, Jian, Xiang Shao, and Jiayang Li. "Indole-3-glycerol phosphate, a branchpoint of indole-3-acetic acid biosynthesis from the tryptophan biosynthetic pathway in Arabidopsis thaliana." The Plant Journal 24.3 (2000): 327-334.
Solyc12g096770.1	acetyl-CoA-benzylalcohol acetyltransferase-like	BEAT	N/A	Involved in the biosynthesis of benzyl acetate, a major constituent of the floral scent	Increased synthesis of benzyl acetate	Dudareva, Natalia, et al. "Acetyl-CoA: benzylalcohol acetyltransferase–an enzyme involved in floral scent production in Clarkia breweri." The Plant Journal 14.3 (1998): 297-304.
Solyc02g093180.3	uncharacterized acetyltransferase At3g50280	AT3G50280	AT3G50280	N/A	N/A	N/A
Solyc04g077980.1	C2H2-type zinc finger protein	At4g35610	At4g35610	Transcription factor activity	Promoted transcription regulation	Cheng, Chia-Yi, et al. "Araport11: a complete reannotation of the Arabidopsis thaliana reference genome." The Plant Journal 89.4 (2017): 789-804.
Solyc07g040960.1	Solanum lycopersicum salt responsive protein 2 (SRG2), mRNA	SRG2	N/A	N/A	N/A	N/A
Solyc11g071740.2	putative calcium-binding protein CML19	CML19	N/A	Calcium sensor	Promoted calcium sensing	Boonburapong, Bongkoj, and Teerapong Buaboocha. "Genome-wide identification and analyses of the rice calmodulin and related potential calcium sensor proteins." BMC plant biology 7.1 (2007): 4.

TomatoID	NCBI Protein Name	Gene ID	*A. Thaliana* Homolog	Uniprot Description	Putative Consequences for Infection	Citation
Solyc03g116890.3	probable WRKY transcription factor 40 isoform X1	WRKY40	AT1G80840	Transcription factor	N/A	Eulgem, Thomas, et al. "The WRKY superfamily of plant transcription factors." Trends in plant science 5.5 (2000): 199-206.
Solyc04g074770.2	uncharacterized protein LOC101267111	N/A	N/A	N/A	N/A	N/A
Solyc11g011030.2	Pto-responsive gene 1 protein	PTI1	N/A	A serine-threonine kinase involved in the hypersensitive response-mediated signaling cascade	Promoted hypersensitive response-mediated signaling	Zhou, Jianmin, et al. "The tomato gene Pti1 encodes a serine/threonine kinase that is phosphorylated by Pto and is involved in the hypersensitive response." Cell 83.6 (1995): 925-935.
Solyc09g010980.1	laccase-17	LAC17	AT5G60020	Involved in lignin degradation and detoxification of lignin-derived products	Increased lignin degradation and detoxification of lignin-derived products	Sibout, Richard, et al. "CINNAMYL ALCOHOL DEHYDROGENASE-C and-D are the primary genes involved in lignin biosynthesis in the floral stem of Arabidopsis." The Plant Cell 17.7 (2005): 2059-2076.
Solyc04g079730.1	allene oxide synthase	CYP74A	AT5G42650	Involved in the synthesis of JA	Increased production of JA	Laudert, Dietmar, et al. "Cloning, molecular and functional characterization of Arabidopsis thaliana allene oxide synthase (CYP 74), the first enzyme of the octadecanoid pathway to jasmonates." Plant molecular biology 31.2 (1996): 323-335.
Solyc03g098760.2	serine protease inhibitor 5-like	SCPL5	AT1G73290	Carboxypeptidase	N/A	N/A
Solyc11g018777.1	N/A	N/A	N/A	N/A	N/A	N/A
Solyc00g012430.1	N/A	N/A	N/A	N/A	N/A	N/A
Solyc02g090970.1	mitogen-activated protein kinase kinase kinase 17-like	MAPKKK17	AT2G32510	Act as ABA signal transducer under abiotic stress	Increased response to stress	Danquah, Agyemang, et al. "Identification and characterization of an ABA-activated MAP kinase cascade in Arabidopsis thaliana." The Plant Journal 82.2 (2015): 232-244.
Solyc03g098730.1	kunitz trypsin inhibitor 2	KTI2	AT1G72290	Involved in the control of cell death in the transmitting tract and septum epidermis during flower development; Plays a role in herbivore resistance during seedling greening	Increased programmed cell death; Increased defense against herbivores	Shoji, Tsubasa, Takayuki Ogawa, and Takashi Hashimoto. "Jasmonate-induced nicotine formation in tobacco is mediated by tobacco COI1 and JAZ genes." Plant and cell physiology 49.7 (2008): 1003-1012.
Solyc06g066800.2	protein CDI	AT1G64980	AT1G64980	Nucleotide-diphospho-sugar transferase required for pollen germination and tube growth	Promoted pollen germination and tube growth	Li, Hong-Mei, et al. "Cdi gene is required for pollen germination and tube growth in Arabidopsis." FEBS letters 586.7 (2012): 1027-1031.
Solyc07g056000.2	xyloglycan endo-transglycosylase precursor	XET1	N/A	Involved in carbohydrate metabolic process	Increased carbohydrate metabolism	Tucker, Mark L., et al. "Gene expression profiles for cell wall-modifying proteins associated with soybean cyst nematode infection, petiole abscission, root tips, flowers, apical buds, and leaves." Journal of Experimental Botany 58.12 (2007): 3395-3406.

TomatoID	NCBI Protein Name	Gene ID	*A. Thaliana* Homolog	Uniprot Description	Putative Consequences for Infection	Citation
Solyc02g083880.3	gibberellin-regulated protein 11	GASA11	AT2G18420	Gibberellin-regulated protein that functions in hormonal controlled steps of reproductive development	Promoted plant development	Cheng, Chia-Yi, et al. "Araport11: a complete reannotation of the Arabidopsis thaliana reference genome." The Plant Journal 89.4 (2017): 789-804.
Solyc07g042170.3	protein TIFY 10b-like	AT1G74950	AT1G74950	Repressor of jasmonate responses; Negatively regulates root hair development	Repressed jasmonate responses; Impaired root hair development	Fernández-Calvo, Patricia, et al. "The Arabidopsis bHLH transcription factors MYC3 and MYC4 are targets of JAZ repressors and act additively with MYC2 in the activation of jasmonate responses." The Plant Cell 23.2 (2011): 701-715.
Solyc04g074430.1	protein EXORDIUM-like	EXO	AT4G08950	Required for cell expansion in leaves; Mediate brassinosteroid-induced leaf and root growth/development	Promoted cell expansion in the leaves; Promoted leaf and root growth/development	Coll-Garcia, Danahe, et al. "EXORDIUM regulates brassinosteroid-responsive genes." FEBS letters 563.1-3 (2004): 82-86.
Solyc08g036640.3	protein TIFY 5A-like	TIFY5A	AT1G30135	Repressor of jasmonate responses; Interacts with and suppresses RHD6 and RSL1 transcription factor activities to negatively regulate root hair development	Repressed jasmonate responses; Impaired root hair development	Chung, Hoo Sun, and Gregg A. Howe. "A critical role for the TIFY motif in repression of jasmonate signaling by a stabilized splice variant of the JASMONATE ZIM-domain protein JAZ10 in Arabidopsis." The Plant Cell 21.1 (2009): 131-145.
Solyc04g007580.1	uncharacterized protein LOC101267223	N/A	N/A	N/A	N/A	N/A
Solyc12g049400.2	protein TIFY 10b-like	AT1G74950	AT1G74950	Repressor of jasmonate responses; Negatively regulates root hair development	Repressed jasmonate responses; Impaired root hair development	Fernández-Calvo, Patricia, et al. "The Arabidopsis bHLH transcription factors MYC3 and MYC4 are targets of JAZ repressors and act additively with MYC2 in the activation of jasmonate responses." The Plant Cell 23.2 (2011): 701-715.
Solyc07g006560.3	ribonuclease MC-like	LOC107896791	N/A	Involved in RNA catabolic process	N/A	Li, Fuguang, et al. "Genome sequence of cultivated Upland cotton (Gossypium hirsutum TM-1) provides insights into genome evolution." Nature biotechnology 33.5 (2015): 524-530.
Solyc05g015800.3	methanol O-anthraniloyltransferase	AMAT	N/A	Acyltransferase that catalyzes the formation of methyl anthranilate	Increased production of methyl anthranilate	Wang, Jihong, and Vincenzo De Luca. "The biosynthesis and regulation of biosynthesis of Concord grape fruit esters, including 'foxy'methylanthranilate." The Plant Journal 44.4 (2005): 606-619.
Solyc10g086180.2	phenylalanine ammonia-lyase	PAL	N/A	Enzyme of plant metabolism catalyzing the first reaction in the biosynthesis from L-phenylalanine of a wide variety of natural products based on the phenylpropane skeleton	Increased biosynthesis of L-phenylalanine derivatives	N/A
Solyc08g076970.3	N2-acetylornithine deacetylase	N/A	N/A	N/A	N/A	N/A

TomatoID	NCBI Protein Name	Gene ID	*A. Thaliana* Homolog	Uniprot Description	Putative Consequences for Infection	Citation
Solyc11g071760.3	regulator of gene silencing	N/A	N/A	N/A	N/A	N/A
Solyc06g051940.3	protein phosphatase 2C	PP2CA	AT3G11410	Major negative regulator of ABA responses during seed germination and cold acclimation; Prevents stomata closure	Impaired ABA responses; Promoted stomatal opening	Chérel, Isabelle, et al. "Physical and functional interaction of the Arabidopsis K+ channel AKT2 and phosphatase AtPP2CA." The Plant Cell 14.5 (2002): 1133-1146.
Solyc01g007030.3	E3 ubiquitin-protein ligase PUB22-like	PUB22	AT3G52450	Negatively regulates water stress response; Mediates drought signaling pathway; Negative regulator of immune response triggered by PAMPs	Impaired response to drought; Impaired response to PAMPs; Impaired attenuation to PAMP-induced signaling	Trujillo, Marco, et al. "Negative regulation of PAMP-triggered immunity by an E3 ubiquitin ligase triplet in Arabidopsis." Current Biology 18.18 (2008): 1396-1401.
Solyc03g013160.3	amino acid transporter AVT1I	AVT1I	AT3G28960	Amino acid transporter	Increased amino acid transport	Fujiki, Yuki, et al. "Functional identification of AtAVT3, a family of vacuolar amino acid transporters, in Arabidopsis." FEBS letters 591.1 (2017): 5-15.
Solyc12g019320.2	protein DETOXIFICATION 30-like	LOC107764941	N/A	Xenobiotic transmembrane transporter activity	Increased detoxification	Sierro, Nicolas, et al. "The tobacco genome sequence and its comparison with those of tomato and potato." Nature communications 5.1 (2014): 1-9.
Solyc11g018805.1	N/A	N/A	N/A	N/A	N/A	N/A
Solyc07g041920.3	cysteine proteinase 3-like	CYS3	AT2G40880	Involved in the regulation of endogenous processes; Involved in the regulation of defense against pests and pathogens	Promoted regulation of endogenous processes; Increased regulation of defense	Belenghi, Beatrice, et al. "AtCYS1, a cystatin from Arabidopsis thaliana, suppresses hypersensitive cell death." European Journal of Biochemistry 270.12 (2003): 2593-2604.
Solyc00g071180.3	multicystatin	N/A	N/A	N/A	N/A	Jacinto, Tânia, et al. "Leaves of transgenic tomato plants overexpressing prosystemin accumulate high levels of cystatin." Plant Science 138.1 (1998): 35-42.
Solyc12g017460.1	GDSL esterase/lipase At5g03980	AT5G03980	AT5G03980	Hydralose/esterase activity	N/A	Lai, Chia-Ping, et al. "Genome-wide analysis of GDSL-type esterases/lipases in Arabidopsis." Plant molecular biology 95.1-2 (2017): 181-197.
Solyc03g026280.3	C-repeat-binding factor-1	CBF1	N/A	Transcription factor	N/A	Zhen, Ying, and Mark C. Ungerer. "Relaxed selection on the CBF/DREB1 regulatory genes and reduced freezing tolerance in the southern range of Arabidopsis thaliana." Molecular Biology and Evolution 25.12 (2008): 2547-2555.
Solyc02g087210.3	stress-associated protein 11	SAP11	N/A	Involved in environmental stress response	Increased response to environmental stress	Vij, Shubha, and Akhilesh K. Tyagi. "Genome-wide analysis of the stress associated protein (SAP) gene family containing A20/AN1 zinc-finger (s) in rice and their phylogenetic relationship with Arabidopsis." Molecular Genetics and Genomics 276.6 (2006): 565-575.
Solyc10g086500.1	steroid 5-alpha-reductase DET2	DET2	AT2G38050	Involved in a reduction step in the biosynthesis of the plant steroid, brassinolide	Increased brassinolide biosynthesis	Fujioka, Shozo, et al. "The Arabidopsis deetiolated2 mutant is blocked early in brassinosteroid biosynthesis." The Plant Cell 9.11 (1997): 1951-1962.
Solyc00g145170.2	proteinase inhibitor type-2 precursor	N/A	N/A	N/A	N/A	Balandin, Teresa, et al. "Structure and induction pattern of a novel proteinase inhibitor class II gene of tobacco." Plant molecular biology 27.6 (1995): 1197-1204.

TomatoID	NCBI Protein Name	Gene ID	*A. Thaliana* Homolog	Uniprot Description	Putative Consequences for Infection	Citation
Solyc11g018800.2	lignin-forming anionic peroxidase	N/A	N/A	Involved in removal of H2O2, oxidation of toxic reductants, biosynthesis and degradation of lignin, suberization, auxin catabolism, and response to biotic stressors	Increased response to oxidative stress; Increased auxin catabolism; Increased defense against wounding and pathogen attack	Lagrimini, L. Mark, et al. "Molecular cloning of complementary DNA encoding the lignin-forming peroxidase from tobacco: molecular analysis and tissue-specific expression." Proceedings of the National Academy of Sciences 84.21 (1987): 7542-7546.
Solyc08g036660.3	protein TIFY 5A-like	TIFY5A	AT1G30135	Repressor of jasmonate responses; Interacts with and suppresses RHD6 and RSL1 transcription factor activities to negatively regulate root hair development	Repressed jasmonate responses; Impaired root hair development	Chung, Hoo Sun, and Gregg A. Howe. "A critical role for the TIFY motif in repression of jasmonate signaling by a stabilized splice variant of the JASMONATE ZIM-domain protein JAZ10 in Arabidopsis." The Plant Cell 21.1 (2009): 131-145.
Solyc12g057060.2	7-deoxyloganetin glucosyltransferase-like	N/A	N/A	Iridoid glucosyltransferase acting exclusively on 7-deoxyloganetin; Involved in the synthesis of secologanin	Increassed synthesis of secologanin	Asada, Keisuke, et al. "A 7-deoxyloganetic acid glucosyltransferase contributes a key step in secologanin biosynthesis in Madagascar periwinkle." The Plant Cell 25.10 (2013): 4123-4134.
Solyc02g087540.2	AAA-ATPase At3g28510-like	AT3G28510	AT3G28510	Involved in inter-tissue RNA transport	Increased inter-tissue RNA transport	Thieme, Christoph J., et al. "Endogenous Arabidopsis messenger RNAs transported to distant tissues." Nature Plants 1.4 (2015): 1-9.
Solyc08g007830.1	dehydration-responsive element-binding protein 1F-like	DREB1F	AT1G12610	Transcriptional activator that binds specifically to the C-repeat/DRE element mediating cold-inducible transcription	Increased cold acclimation and freezing tolerance	Sakuma, Yoh, et al. "DNA-binding specificity of the ERF/AP2 domain of Arabidopsis DREBs, transcription factors involved in dehydration-and cold-inducible gene expression." Biochemical and biophysical research communications 290.3 (2002): 998-1009.
Solyc06g073830.1	putative calcium-binding protein CML19	CML19	N/A	Calcium sensor	Promoted calcium sensing	Boonburapong, Bongkoj, and Teerapong Buaboocha. "Genome-wide identification and analyses of the rice calmodulin and related potential calcium sensor proteins." BMC plant biology 7.1 (2007): 4.
Solyc08g068600.3	aromatic amino acid decarboxylase 1B	AADC1B	N/A	Involved in carboxylic acid metabolic process	Increased carboxylic acid metabolism	Tieman, Denise, et al. "Tomato aromatic amino acid decarboxylases participate in synthesis of the flavor volatiles 2-phenylethanol and 2-phenylacetaldehyde." Proceedings of the National Academy of Sciences 103.21 (2006): 8287-8292.
Solyc11g071470.1	agmatine hydroxycinnamoyltransferase 1-like	AHT1	AT3G61600	Promotes the synthesis of hydroxycinnamic acid amides, which play a role in defense against pathogens	Increased defense against pathogens	Chen, Wei, et al. "Genome-wide association analyses provide genetic and biochemical insights into natural variation in rice metabolism." Nature genetics 46.7 (2014): 714-721.
Solyc01g096670.3	cytochrome P450 98A2	CYP98A2	N/A	Heme binding; Iron ion binding; Oxidoreductase activity	N/A	N/A
Solyc05g005150.1	F-box/kelch-repeat protein At1g23390	AT1G23390	AT1G23390	Involved in proteasome-mediated, ubiquitin-dependent protein catabolic process	N/A	Pischke, Melissa S., et al. "A transcriptome-based characterization of habituation in plant tissue culture." Plant Physiology 140.4 (2006): 1255-1278.

TomatoID	NCBI Protein Name	Gene ID	*A. Thaliana* Homolog	Uniprot Description	Putative Consequences for Infection	Citation
Solyc06g035700.1	ethylene-responsive transcription factor ERF025-like	ERF025	AT5G52020	Binds to the GCC-box pathogenesis-related promoter element; Regulates gene expression under stress	Increased response to stress and pathogenesis	Riechmann, José Luis, et al. "Arabidopsis transcription factors: genome-wide comparative analysis among eukaryotes." Science 290.5499 (2000): 2105-2110.
Solyc01g007040.3	E3 ubiquitin-protein ligase PUB22-like	PUB22	AT3G52450	Negatively regulates water stress response; Mediates drought signaling pathway; Negative regulator of immune response triggered by PAMPs	Impaired response to drought; Impaired response to PAMPs; Impaired attenuation to PAMP-induced signaling	Trujillo, Marco, et al. "Negative regulation of PAMP-triggered immunity by an E3 ubiquitin ligase triplet in Arabidopsis." Current Biology 18.18 (2008): 1396-1401.
Solyc04g072375.1	N/A	N/A	N/A	N/A	N/A	N/A
Solyc02g079590.3	receptor-like serine/threonine-protein kinase SD1-8	SD18	AT4G21380	Involved in the regulation of cellular expansion and differentiation	Increased regulation of cellular expansion and differentiation	Ascencio-Ibáñez, José Trinidad, et al. "Global analysis of Arabidopsis gene expression uncovers a complex array of changes impacting pathogen response and cell cycle during geminivirus infection." Plant physiology 148.1 (2008): 436-454.
Solyc01g068065.1	N/A	N/A	N/A	N/A	N/A	N/A
Solyc11g010250.1	uncharacterized protein LOC101262884	N/A	N/A	N/A	N/A	N/A
Solyc02g086300.3	putative glutamine amidotransferase GAT1_2.1	GAT1_2.1	AT1G15040	Flutamine amidotransferase that represses shoot branching; Links nitrogen stress response and branching control	Impaired shoot branching; Stunting	Zhu, Huifen, and Robert G. Kranz. "A nitrogen-regulated glutamine amidotransferase (GAT1_2. 1) represses shoot branching in Arabidopsis." Plant physiology 160.4 (2012): 1770-1780.
Solyc12g057160.1	classical arabinogalactan protein 5	AGP5	AT1G35230	Proteoglycan involved in differentiation, cell-cell recognition, embryogenesis, and programmed cell death	Promoted plant growth/development and cell differentiation; Promoted programmed cell death	Thieme, Christoph J., et al. "Endogenous Arabidopsis messenger RNAs transported to distant tissues." Nature Plants 1.4 (2015): 1-9.
Solyc11g010170.2	lanC-like protein GCL1	GCL1	AT5G65280	Plays a role in signaling	N/A	Gao, Yajun, et al. "Genetic characterization reveals no role for the reported ABA receptor, GCR2, in ABA control of seed germination and early seedling development in Arabidopsis." The Plant Journal 52.6 (2007): 1001-1013.
Solyc11g044620.1	N/A	N/A	N/A	N/A	N/A	N/A
Solyc07g064650.2	N/A	N/A	N/A	N/A	N/A	N/A
Solyc10g076660.2	probable 2-oxoglutarate-dependent dioxygenase At5g05600 isoform X1	AT5G05600	AT5G05600	Involved in anthocyanin and protoanthocyanidin biosynthesis by catalyzing the oxidation of leucoanthocyanidins into anthocyanidins	Increased anthocyanin and protoanthocyanidin biosynthesis	Wang, Yi, et al. "Transcriptome analyses show changes in gene expression to accompany pollen germination and tube growth in Arabidopsis." Plant physiology 148.3 (2008): 1201-1211.
Solyc01g095150.3	late embryogenesis (Lea)-like protein	N/A	At4g27400	N/A	N/A	N/A

TomatoID	NCBI Protein Name	Gene ID	*A. Thaliana* Homolog	Uniprot Description	Putative Consequences for Infection	Citation
Solyc04g074420.1	protein EXORDIUM	EXO	AT4G08950	Required for cell expansion in leaves; Mediate brassinosteroid-induced leaf and root growth/development	Promoted cell expansion in the leaves; Promoted leaf and root growth/development	Coll-Garcia, Danahe, et al. "EXORDIUM regulates brassinosteroid-responsive genes." FEBS letters 563.1-3 (2004): 82-86.
Solyc06g082530.2	scarecrow-like protein 9	SCL9	AT2G37650	Transcription factor involved in plant development	Promoted plant development	Menges, Margit, et al. "Cell cycle-regulated gene expression inarabidopsis." Journal of Biological Chemistry 277.44 (2002): 41987-42002.
Solyc04g082140.3	multicopper oxidase-like protein precursor	LPR1	AT1G23010	Multicopper oxidase involved in Cu homeostasis and oxidative stress response; Necessary for root growth inhibition	Promoted Cu homeostasis; Increased response to oxidative stress; Root growth inhibition	Svistoonoff, Sergio, et al. "Root tip contact with low-phosphate media reprograms plant root architecture." Nature genetics 39.6 (2007): 792-796.
Solyc00g095860.1	1-aminocyclopropane-1-carboxylate synthase-like	ACS1	AT3G61510	Involved in ethylene biosynthesis	Increased ethylene biosynthesis; Increased ethylene-related signaling	Tsuchisaka, Atsunari, et al. "A combinatorial interplay among the 1-aminocyclopropane-1-carboxylate isoforms regulates ethylene biosynthesis in Arabidopsis thaliana." Genetics 183.3 (2009): 979-1003.
Solyc10g009270.3	transcription factor MYC2-like	MYC2	AT1G32640	Involved in the regulation of ABA-inducible genes under drought stress; Negative regulator of light-regulated gene expression and growth; Positive regulator of lateral root formation; Regulates sesquiterpene biosynthesis	Promoted response to drought; Impaired response to light-mediated expression; Promoted lateral root formation; Increased regulation of sesquiterpene biosynthesis	Abe, Hiroshi, et al. "Role of Arabidopsis MYC and MYB homologs in drought-and abscisic acid-regulated gene expression." The Plant Cell 9.10 (1997): 1859-1868.
Solyc01g108240.3	ethylene-responsive transcription factor ERF109	ERF109	AT4G34410	Binds to the GCC-box pathogenesis-related promoter element; Regulates gene expression under stress	Increased response to stress and pathogenesis	Riechmann, José Luis, et al. "Arabidopsis transcription factors: genome-wide comparative analysis among eukaryotes." Science 290.5499 (2000): 2105-2110.
Solyc06g083130.3	dCTP pyrophosphatase 1	DCTPP1	AT3G25400	Hydrolyzes dNTPs to the corresponding nucleoside monophosphates; Protects DNA/RNA against the incorporation of these genotoxic nucleotide analogs through their catabolism	Increased DNA/RNA protection against genotoxic nucleotide analogs	Cheng, Chia-Yi, et al. "Araport11: a complete reannotation of the Arabidopsis thaliana reference genome." The Plant Journal 89.4 (2017): 789-804.
Solyc09g011860.3	O-fucosyltransferase 19-like	OFUT19	AT2G37980	Involved in Glycan metabolism	Increased Glycan metabolism	Culligan, Kevin M., et al. "ATR and ATM play both distinct and additive roles in response to ionizing radiation." The Plant Journal 48.6 (2006): 947-961.
Solyc09g061840.3	acetyl-CoA C-acetyltranferase 5	N/A	N/A	Involved in plant growth and development	Increased regulation of plant growth/development	Jin, Huanan, Zhihong Song, and Basil J. Nikolau. "Reverse genetic characterization of two paralogous acetoacetyl CoA thiolase genes in Arabidopsis reveals their importance in plant growth and development." The Plant Journal 70.6 (2012): 1015-1032.

TomatoID	NCBI Protein Name	Gene ID	*A. Thaliana* Homolog	Uniprot Description	Putative Consequences for Infection	Citation
Solyc10g005480.3	F-box protein SKIP27	SKIP27	AT4G21510	Component of SCF(ASK-cullin-F-box) E3 ubiquitin ligase complexes, which mediate the ubiquitination and subsequent proteasomal degradation of target proteins	Increased proteasomal degradation	Risseeuw, Eddy P., et al. "Protein interaction analysis of SCF ubiquitin E3 ligase subunits from Arabidopsis." The Plant Journal 34.6 (2003): 753-767.
Solyc04g005040.1	matrix metalloproteinase 2 precursor	2MMP	AT1G70170	Play a role in the degradation and remodeling of the extracellular matrix during development or in response to stressors; Required for plant growth, morphogenesis, and development	Increased degradation and remodeling of the extracellular matrix; Promoted plant growth, morphogenesis, and development; Promoted flowering and senescence	Golldack, Dortje, Olga V. Popova, and Karl-Josef Dietz. "Mutation of the matrix metalloproteinase At2-MMP inhibits growth and causes late flowering and early senescence in Arabidopsis." Journal of Biological Chemistry 277.7 (2002): 5541-5547.
Solyc10g084320.2	subtilisin-like protease SBT1.9	SBT1.9	AT5G67090	Involved in proteolysis	Increased proteolysis	Barrero, José María, et al. "INCURVATA2 encodes the catalytic subunit of DNA polymerase α and interacts with genes involved in chromatin-mediated cellular memory in Arabidopsis thaliana." The Plant Cell 19.9 (2007): 2822-2838.
Solyc07g052203.1	N/A	N/A	N/A	N/A	N/A	N/A
Solyc03g096050.3	probable 2-oxoglutarate-dependent dioxygenase At5g05600	AT5G05600	AT5G05600	Involved in anthocyanin and protoanthocyanidin biosynthesis	Increased anthocyanin and protoanthocyanidin biosynthesis	Wang, Yi, et al. "Transcriptome analyses show changes in gene expression to accompany pollen germination and tube growth in Arabidopsis." Plant physiology 148.3 (2008): 1201-1211.
Solyc06g061200.1	glycine-rich protein TomR2	TomR2	N/A	N/A	N/A	Lin, Wan-Chi, et al. "A glycine-rich protein gene family predominantly expressed in tomato roots, but not in leaves and ripe fruit." Plant science 168.2 (2005): 283-295.
Solyc09g072750.2	uncharacterized protein LOC104649361	N/A	N/A	N/A	N/A	N/A
Solyc12g014430.2	cellulose synthase-like protein G3	CSLG3	AT4G23990	Golgi-localized beta-glycan synthase that polymerize the backbones of noncellulosic polysaccharides of plant cell wall	Promoted plant cell wall growth/development	Lao, Jeemeng, et al. "The plant glycosyltransferase clone collection for functional genomics." The Plant Journal 79.3 (2014): 517-529.
Solyc02g077330.3	GDSL-like lipase/acylhydrolase superfamily protein precursor	AT1G20132	AT1G20132	Lipid metabolic process	Increased lipid catabolism	Cheng, Chia-Yi, et al. "Araport11: a complete reannotation of the Arabidopsis thaliana reference genome." The Plant Journal 89.4 (2017): 789-804.
Solyc03g122340.3	lipoxygenase	N/A	N/A	Involved in growth and development, pest resistance, and senescence in response to wounding	Promoted plant growth/development, pest resistance, and senescence	Chechetkin, I. R., et al. "Specificity of oxidation of linoleic acid homologs by plant lipoxygenases." Biochemistry (Moscow) 74.8 (2009): 855-861.

TomatoID	NCBI Protein Name	Gene ID	*A. Thaliana* Homolog	Uniprot Description	Putative Consequences for Infection	Citation
Solyc08g068610.3	aromatic amino acid decarboxylase 1B	AADC1B	N/A	Involved in carboxylic acid metabolic process	Increased carboxylic acid metabolism	Tieman, Denise, et al. "Tomato aromatic amino acid decarboxylases participate in synthesis of the flavor volatiles 2-phenylethanol and 2-phenylacetaldehyde." Proceedings of the National Academy of Sciences 103.21 (2006): 8287-8292.
Solyc10g075110.2	non-specific lipid-transfer protein 1 precursor	LTP1	N/A	Play a role in wax or cutin deposition in the cell walls of expanding epidermal cells and certain secretory tissues	Promoted cell wall expansion	Skriver, Karen, et al. "Structure and expression of the barley lipid transfer protein gene Ltp1." Plant molecular biology 18.3 (1992): 585-589.
Solyc12g087790.1	N/A	N/A	N/A	N/A	N/A	N/A
Solyc07g045350.3	Acetyl-CoA acetyltransferase, cytosolic 1	AAT1	AT5G48230	Involved in plant growth and development	Increased regulation of plant growth/development	Jin, Huanan, Zhihong Song, and Basil J. Nikolau. "Reverse genetic characterization of two paralogous acetoacetyl CoA thiolase genes in Arabidopsis reveals their importance in plant growth and development." The Plant Journal 70.6 (2012): 1015-1032.
Solyc06g074030.1	probable CCR4-associated factor 1 homolog 9	CAF1-9	AT3G44260	Ubiquitous transcription factor required for a diverse set of processes; Component of the CCR4 complex involved in the control of gene expression	Promoted gene expression	Chang, Shuang, and Craig S. Pikaard. "Transcript profiling in Arabidopsis reveals complex responses to global inhibition of DNA methylation and histone deacetylation." Journal of Biological Chemistry 280.1 (2005): 796-804.
Solyc10g050970.1	ethylene-responsive transcription factor ERF109-like	ERF109	AT4G34410	Regulates gene expression under stress	Increased response to stress and pathogenesis	Riechmann, José Luis, et al. "Arabidopsis transcription factors: genome-wide comparative analysis among eukaryotes." Science 290.5499 (2000): 2105-2110.
Solyc12g009000.1	protein ALP1-like	AT3G55350	AT3G55350	Transposase-derived protein that has nuclease activity	N/A	Joly-Lopez, Zoé, et al. "Abiotic stress phenotypes are associated with conserved genes derived from transposable elements." Frontiers in plant science 8 (2017): 2027.
Solyc12g010420.1	uncharacterized protein LOC104644843	N/A	N/A	N/A	N/A	N/A
Solyc05g006220.3	IAA-amino acid hydrolase ILR1-like 2	ILL2	AT5G56660	Hydrolyzes certain amino acid conjugates of the plant growth regulator indole-3-acetic acid; Acts with ILR1 to provide free IAA to germinating seedlings	Promoted regulation of plant growth; Increased IAA supply to seedlings; Increased response to auxin	LeClere, Sherry, et al. "Characterization of a family of IAA-amino acid conjugate hydrolases from Arabidopsis." Journal of Biological Chemistry 277.23 (2002): 20446-20452.
Solyc10g076250.2	alanine--glyoxylate aminotransferase 2 homolog 2, mitochondrial	AGT3	AT2G38400	Transminase activity	N/A	Carrie, Chris, et al. "Identification of cleavage sites and substrate proteins for two mitochondrial intermediate peptidases in Arabidopsis thaliana." Journal of experimental botany 66.9 (2015): 2691-2708.

TomatoID	NCBI Protein Name	Gene ID	*A. Thaliana* Homolog	Uniprot Description	Putative Consequences for Infection	Citation
Solyc08g079090.3	monocopper oxidase-like protein SKU5	SKU5	AT4G12420	Involved in directional growth processes, possibly by participating in cell wall expansion	Promoted cell wall expansion; Promoted directional growth processes	Shahollari, Bationa, et al. "A leucine-rich repeat protein is required for growth promotion and enhanced seed production mediated by the endophytic fungus Piriformospora indica in Arabidopsis thaliana." The Plant Journal 50.1 (2007): 1-13.
Solyc08g074682.1	N/A	N/A	N/A	N/A	N/A	N/A
Solyc03g124110.2	dehydration-responsive element-binding protein 1A	DREB1A	AT4G25480	Transcriptional activator that binds specifically to the C-repeat/DRE element mediating cold-inducible transcription	Increased cold acclimation and freezing tolerance	Alonso-Blanco, Carlos, et al. "Genetic and molecular analyses of natural variation indicate CBF2 as a candidate gene for underlying a freezing tolerance quantitative trait locus in Arabidopsis." Plant Physiology 139.3 (2005): 1304-1312.
Solyc04g063210.3	probable caffeoyl-CoA O-methyltransferase At4g26220 isoform X2	AT4G26220	AT4G26220	Plays a role in the synthesis of feruloylated polysaccharides; Involved in the reinforcement of the plant cell wall; Involved in response to wounding and/or pathogen challenge	Increased reinforcement of plant cell walls; Promoted response to wounding and/or pathogen challenge	Meyermans, Hugo, et al. "Modifications in lignin and accumulation of phenolic glucosides in poplar xylem upon down-regulation of caffeoyl-coenzyme A O-methyltransferase, an enzyme involved in lignin biosynthesis." Journal of Biological Chemistry 275.47 (2000): 36899-36909.
Solyc08g081550.3	1-aminocyclopropane-1-carboxylate synthase	ACS1	AT3G61510	Involved in ethylene biosynthesis	Increased ethylene biosynthesis	Tsuchisaka, Atsunari, et al. "A combinatorial interplay among the 1-aminocyclopropane-1-carboxylate isoforms regulates ethylene biosynthesis in Arabidopsis thaliana." Genetics 183.3 (2009): 979-1003.
Solyc08g068710.1	tyramine N-feruloyltransferase 4/11-like	THT4	N/A	Synthesizes amides which are involved in stress response in the cell wall	Promoted stress response in the plant cell wall	Farmer, Mary Jo, et al. "Identification and characterization of cDNA clones encoding hydroxycinnamoyl-CoA: tyramine N-hydroxycinnamoyltransferase from tobacco." European journal of biochemistry 263.3 (1999): 686-694.
Solyc01g005865.1	N/A	N/A	N/A	N/A	N/A	N/A
Solyc01g005870.2	receptor-like protein Cf-9	CF-9	N/A	Involved in plant defense; Confers resistance to the fungal pathogen	Promoted defense against certain fungal pathogens	van der Hoorn, Renier AL, et al. "Structure–function analysis of cf-9, a receptor-like protein with extracytoplasmic leucine-rich repeats." The Plant Cell 17.3 (2005): 1000-1015.
Solyc01g105650.3	protein DMR6-LIKE OXYGENASE 2-like	DLO2	AT4G10490	Component of a negative feedback regulation system of SA levels; Negative regulator of defense against certain pathogens	Negative system regulation of SA; Down regulaton of defense against certain pathogens	Zeilmaker, Tieme, et al. "DOWNY MILDEW RESISTANT 6 and DMR 6-LIKE OXYGENASE 1 are partially redundant but distinct suppressors of immunity in Arabidopsis." The Plant Journal 81.2 (2015): 210-222.
Solyc12g009240.1	ethylene-responsive transcription factor ERF017	ERF017	AT1G19210	Regulates gene expression under stress	Increased response to stress and pathogenesis	Riechmann, José Luis, et al. "Arabidopsis transcription factors: genome-wide comparative analysis among eukaryotes." Science 290.5499 (2000): 2105-2110.
Solyc01g058500.3	uncharacterized protein LOC101254183	N/A	N/A	N/A	N/A	N/A
Solyc06g075690.3	auxin-regulated protein isoform X1	N/A	N/A	N/A	N/A	N/A

TomatoID	NCBI Protein Name	Gene ID	*A. Thaliana* Homolog	Uniprot Description	Putative Consequences for Infection	Citation
Solyc06g083040.3	wound-inducible carboxypeptidase	N/A	N/A	N/A	N/A	N/A
Solyc07g006890.1	cytochrome P450 94A1-like	CYP94A1	N/A	Catalyzes the omega-hydroxylation of various fatty acids; Plays a minor role in cutin synthesis and plant defense	Increased cutin synthesis and plant defense	TIJET, Nathalie, et al. "Functional expression in yeast and characterization of a clofibrate-inducible plant cytochrome P-450 (CYP94A1) involved in cutin monomers synthesis." Biochemical Journal 332.2 (1998): 583-589.
Solyc06g074530.1	arogenate dehydratase/prephenate dehydratase 6, chloroplastic-like	ADT6	AT1G08250	Converts the prephenate produced from the shikimate-chorismate pathway into phenylalanine	Increased phenylalanine production; Increased production of primary and secondary metabolites	Cho, Man-Ho, et al. "Phenylalanine biosynthesis in Arabidopsis thaliana identification and characterization of Arogenate dehydratases." Journal of Biological Chemistry 282.42 (2007): 30827-30835.
Solyc12g005450.1	probable receptor-like protein kinase At4g10390	AT4G10390	AT4G10390	Involved in protein phosphorylation; Involved in response to wounding	Increased protein phosphorylation; Promoted response to wounding	Peng, Mingsheng, et al. "Genome-wide analysis of Arabidopsis responsive transcriptome to nitrogen limitation and its regulation by the ubiquitin ligase gene NLA." Plant molecular biology 65.6 (2007): 775-797.
Solyc04g074950.3	esterase CG5412 isoform X2	CG5412	N/A	Hydrolase activity	N/A	N/A
Solyc07g048060.2	cytochrome b561 and DOMON domain-containing protein At5g48750	AT5G48750	AT5G48750	Involved in oxidation-reduction process	Improved oxidation-reduction	Kotani, Hirokazu, et al. "Structural analysis of Arabidopsis thaliana chromosome 5. VI. Sequence features of the regions of 1,367,185 bp covered by 19 physically assigned P1 and TAC clones." DNA Research 5.3 (1998): 203-216.
Solyc08g077020.1	auxin-responsive protein SAUR76	SAUR76	AT5G20820	Involved in the regulation of ethylene receptor signaling; Promotes cell expansion and plant growth; Involved in the regulation of cell elongation	Increased ethylene receptor signaling; Promoted cell expansion and plant growth/development; Leaf curl	Markakis, Marios Nektarios, et al. "Characterization of a small auxin-up RNA (SAUR)-like gene involved in Arabidopsis thaliana development." PloS one 8.11 (2013): e82596.
Solyc01g097520.3	annexin D4-like	ANN4	AT2G38750	Involved in osmotic stress response and ABA signaling	Increased osmostic stress and ABA signaling	Lee, Sumin, et al. "Proteomic identification of annexins, calcium-dependent membrane binding proteins that mediate osmotic stress and abscisic acid signal transduction in Arabidopsis." The Plant Cell 16.6 (2004): 1378-1391.
Solyc03g111290.1	cytochrome P450 94B3-like	CYP94B3	AT3G48520	Hydroxylase involved in the oxidation of the plant hormone JA-Ile; Exerts negative feedback control on JA-Ile levels and plays a key role in attenuation of jasmonate responses	Promoted attenuation to JA-Ile and response to jasmonate	Kitaoka, Naoki, et al. "Arabidopsis CYP94B3 encodes jasmonyl-L-isoleucine 12-hydroxylase, a key enzyme in the oxidative catabolism of jasmonate." Plant and Cell Physiology 52.10 (2011): 1757-1765.
Solyc03g119980.3	caffeoylshikimate esterase	CSE	AT1G52760	Involved in the biosynthesis of lignin; Promotes the degradation of lysophosphatidylcholine and detoxifies the peroxidized membrane in response to cadmium-induced oxidative stress	Increased biosynthesis of lignin; Increased detoxification of peroxidized membrane; Increased response to oxidative stress	Gao, Wei, et al. "Acyl-CoA-binding protein 2 binds lysophospholipase 2 and lysoPC to promote tolerance to cadmium-induced oxidative stress in transgenic Arabidopsis." The Plant Journal 62.6 (2010): 989-1003.

TomatoID	NCBI Protein Name	Gene ID	*A. Thaliana* Homolog	Uniprot Description	Putative Consequences for Infection	Citation
Solyc01g090180.3	4,5-DOPA dioxygenase extradiol	DODA	N/A	Opens the cyclic ring of dihydroxy-phenylalanine, producing betalamic acid	Increased betalamic acid production; Increased pigment production	Christinet, Laurent, et al. "Characterization and functional identification of a novel plant 4, 5-extradiol dioxygenase involved in betalain pigment biosynthesis in Portulaca grandiflora." Plant Physiology 134.1 (2004): 265-274.
Solyc01g059990.3	uncharacterized protein LOC101244610	N/A	N/A	N/A	N/A	N/A
Solyc03g007240.3	SPM1 protein	spm1	N/A	Structural constituent of nuclear pore	N/A	Knight, Pamela, et al. "The Theileria annulata sporozoite and macroschizont polypeptide encoded by the spm1 gene shares phenylalanine-glycine motifs with nuclear pore proteins." Molecular & Biochemical Parasitology 1.100 (1999): 135-140.
Solyc03g093080.3	probable xyloglucan endotransglucosylase/hydrolase protein 23	XTH23	AT4G25810	Cleaves and religates xyloglucan polymers, an essential constituent of the primary cell wall; Participates in cell wall construction of growing tissues	Increased cell wall plant expansion; Increased response to auxin	Ascencio-Ibáñez, José Trinidad, et al. "Global analysis of Arabidopsis gene expression uncovers a complex array of changes impacting pathogen response and cell cycle during geminivirus infection." Plant physiology 148.1 (2008): 436-454.
Solyc03g096290.3	aquaporin PIP1-7	PIP1-1	AT3G61430	Water channel required to facilitate the transport of water across cell membrane	Increased water transport	Marmagne, Anne, et al. "Identification of new intrinsic proteins in Arabidopsis plasma membrane proteome." Molecular & Cellular Proteomics 3.7 (2004): 675-691.
Solyc07g006570.3	ribonuclease 3-like	RTL1	AT1G80650	Involved in production of siRNA involved in RNA interference	Increased gene silencing	Charbonnel, Cyril, et al. "The siRNA suppressor RTL1 is redox-regulated through glutathionylation of a conserved cysteine in the double-stranded-RNA-binding domain." Nucleic acids research 45.20 (2017): 11891-11907.
Solyc07g007760.3	defensin-like protein precursor	FST	N/A	Involved in floral organogenesis; Protects reproductive organs from potential pathogen attack	Promoted floral organogenesis; Increased defense against pathogens in reproductive organs	Gu, Qing, et al. "A flower-specific cDNA encoding a novel thionin in tobacco." Molecular and general genetics MGG 234.1 (1992): 89-96.
Solyc01g087590.3	polyamine oxidase 1	PAO1	AT5G13700	Plays an important role in the regulation of polyamine intracellular concentration; Involved in the production of hydrogen peroxide in response to salt and cold stresses	Increased polyamine intracellular concentration; Involved in the production of hydrogen peroxide in response to salt and cold stress	Tavladoraki, Paraskevi, et al. "Heterologous expression and biochemical characterization of a polyamine oxidase from Arabidopsis involved in polyamine back conversion." Plant Physiology 141.4 (2006): 1519-1532.
Solyc02g085730.3	allene oxide cyclase isoform 1, chloroplastic	AOC1	AT3G25760	Involved in the production of 12-oxo-phytodienoic acid, a precursor of JA	Increased production of JA precursor	Zhai, Qingzhe, et al. "Phytochrome chromophore deficiency leads to overproduction of jasmonic acid and elevated expression of jasmonate-responsive genes in Arabidopsis." Plant and Cell Physiology 48.7 (2007): 1061-1071.

TomatoID	NCBI Protein Name	Gene ID	*A. Thaliana* Homolog	Uniprot Description	Putative Consequences for Infection	Citation
Solyc08g006740.3	aromatic amino acid decarboxylase 2	AADC2	N/A	Involved in carboxylic acid metabolic process	Increased carboxylic acid metabolism	Tieman, Denise, et al. "Tomato aromatic amino acid decarboxylases participate in synthesis of the flavor volatiles 2-phenylethanol and 2-phenylacetaldehyde." Proceedings of the National Academy of Sciences 103.21 (2006): 8287-8292.
Solyc09g056385.1	N/A	N/A	N/A	N/A	N/A	N/A
Solyc10g078495.1	N/A	N/A	N/A	N/A	N/A	N/A
Solyc01g007955.1	N/A	N/A	N/A	N/A	N/A	N/A
Solyc10g080690.2	patatin-like protein 3	PLP3	AT4G37050	Possesses non-specific lipolytic acyl hydrolase activity; Plays a role in root development	Promoted root development	Rietz, Steffen, et al. "Roles of Arabidopsis patatin-related phospholipases a in root development are related to auxin responses and phosphate deficiency." Molecular Plant 3.3 (2010): 524-538.
Solyc02g090360.3	L-ascorbate oxidase homolog	N/A	N/A	Oxidoreductase involved in pollen tube growth	Promoted pollen tube growth	Weterings, Koen, et al. "Characterization of a pollen-specific cDNA clone from Nicotiana tabacum expressed during microgametogenesis and germination." Plant molecular biology 18.6 (1992): 1101-1111.
Solyc02g080200.3	pectinesterase	N/A	N/A	Acts in the modification of cell walls via demethylesterification of cell wall pectin	Increased modification of plant cell walls	N/A
Solyc02g080210.3	pectinesterase-like	N/A	N/A	Acts in the modification of cell walls via demethylesterification of cell wall pectin	Increased modification of plant cell walls	N/A
Solyc00g272810.1	probable acetyltransferase NATA1-like	AT2G39020	AT2G39020	N-acetyltransferase activity	N/A	Hanada, Kousuke, et al. "Functional compensation of primary and secondary metabolites by duplicate genes in Arabidopsis thaliana." Molecular biology and evolution 28.1 (2011): 377-382.
Solyc01g007020.3	E3 ubiquitin-protein ligase PUB22-like	PUB22	AT3G52450	Negatively regulates water stress response; Mediates drought signaling pathway; Negative regulator of immune response triggered by PAMPs	Impaired response to drought; Impaired response to PAMPs; Impaired attenuation to PAMP-induced signaling	Trujillo, Marco, et al. "Negative regulation of PAMP-triggered immunity by an E3 ubiquitin ligase triplet in Arabidopsis." Current Biology 18.18 (2008): 1396-1401.
Solyc04g074480.3	phospho-2-dehydro-3-deoxyheptonate aldolase 2, chloroplastic	N/A	N/A	Involved in the synthesis of secondary metabolites derived from intermediates of the pre-chorismate pathway	Increased secondary metabolites derived from intermediates of the pre-chorismate pathway	Görlach, Jörn, et al. "Differential expression of tomato (Lycopersicon esculentum L.) genes encoding shikimate pathway isoenzymes. I. 3-deoxy-D-arabino-heptulosonate 7-phosphate synthase." Plant molecular biology 23.4 (1993): 697-706.
Solyc11g071550.2	probable amidase At4g34880	AT4G34880	AT4G34880	Amidase activity	N/A	Aronsson, Henrik, et al. "Toc64/OEP64 is not essential for the efficient import of proteins into chloroplasts in Arabidopsis thaliana." The Plant Journal 52.1 (2007): 53-68.
Solyc01g091165.1	N/A	N/A	N/A	N/A	N/A	N/A

TomatoID	NCBI Protein Name	Gene ID	*A. Thaliana* Homolog	Uniprot Description	Putative Consequences for Infection	Citation
Solyc02g077050.3	zingipain-2	LOC107763929	N/A	Cysteine-type peptidase activity	N/A	Sierro, Nicolas, et al. "The tobacco genome sequence and its comparison with those of tomato and potato." Nature communications 5.1 (2014): 1-9.
Solyc06g082535.1	N/A	N/A	N/A	N/A	N/A	N/A
Solyc04g082200.2	dehydrin	dhn	N/A	Involved in drought response	Increased drought response	Sander, I., et al. "Multiple wheat flour allergens and cross-reactive carbohydrate determinants bind IgE in baker's asthma." Allergy 66.9 (2011): 1208-1215.
Solyc07g063410.3	NAC domain-containing protein JA2L	JA2L	N/A	Transcription factor that acts in the jasmonate-mediated response to infection; Involved in jasmonate- and coronatine-mediated stomatal reopening in response to infection	Promoted jasmonate- and coronatine-mediated response to infection	Du, Minmin, et al. "MYC2 orchestrates a hierarchical transcriptional cascade that regulates jasmonate-mediated plant immunity in tomato." The Plant Cell 29.8 (2017): 1883-1906.
Solyc02g078890.1	N/A	N/A	N/A	N/A	N/A	N/A
Solyc08g081555.1	N/A	N/A	N/A	N/A	N/A	N/A
Solyc10g076240.2	cationic peroxidase 1	PNC1	N/A	Involved in removal of H2O2, oxidation of toxic reductants, biosynthesis/degradation of lignin, suberization, auxin catabolism, and response to environmental stressesors	Promoted response to oxidative stress and other environmental stressors; Increased lignin and auxin metabolism	Buffard, Dominique, et al. "Molecular cloning of complementary DNAs encoding two cationic peroxidases from cultivated peanut cells." Proceedings of the National Academy of Sciences 87.22 (1990): 8874-8878.
Solyc07g056480.3	glutathione S-transferase/peroxidase	BI-GST/GPX	N/A	Has peroxidase and transferase activity	N/A	Kampranis, Sotirios C., et al. "A novel plant glutathione S-transferase/peroxidase suppresses Bax lethality in yeast." Journal of Biological Chemistry 275.38 (2000): 29207-29216.
Solyc10g084880.3	uncharacterized protein LOC100134881	N/A	N/A	N/A	N/A	N/A
Solyc03g118810.1	calcium-binding allergen Ole e 8	N/A	N/A	N/A	N/A	Ledesma, Amalia, Mayte Villalba, and Rosalía Rodríguez. "Cloning, expression and characterization of a novel four EF-hand Ca2+-binding protein from olive pollen with allergenic activity." FEBS letters 466.1 (2000): 192-196.
Solyc06g034370.1	21 kDa protein-like	N/A	N/A	N/A	N/A	N/A
Solyc02g080120.2	gibberellin 2-beta-dioxygenase 8	GA2OX8	AT4G21200	Catalyzes the 2-beta-hydroxylation of gibberellins precursors, rendering them unable to be converted to their active forms	Increased gibberellin regulation; Impaired growth/development	Schomburg, Fritz M., et al. "Overexpression of a novel class of gibberellin 2-oxidases decreases gibberellin levels and creates dwarf plants." The Plant Cell 15.1 (2003): 151-163.

TomatoID	NCBI Protein Name	Gene ID	*A. Thaliana* Homolog	Uniprot Description	Putative Consequences for Infection	Citation
Solyc10g085190.2	probable 2-oxoglutarate-dependent dioxygenase At3g11180	AT3G11180	AT3G11180	Involved in anthocyanin and protoanthocyanidin biosynthesis by catalyzing the oxidation of leucoanthocyanidins into anthocyanidins	Increased anthocyanin and protoanthocyanidin biosynthesis	Wang, Yi, et al. "Transcriptome analyses show changes in gene expression to accompany pollen germination and tube growth in Arabidopsis." Plant physiology 148.3 (2008): 1201-1211.
Solyc03g093360.3	PLAT domain-containing protein 3	PLAT3	AT5G65158	Involved in response to abiotic stress	Promoted response to abiotic stress	Moskal, William A., et al. "Experimental validation of novel genes predicted in the un-annotated regions of the Arabidopsis genome." BMC genomics 8.1 (2007): 18.
Solyc03g117600.3	shikimate O-hydroxycinnamoyltransferase	HST	AT5G48930	Acyltransferase involved in the biosynthesis of lignin	Increased biosynthesis of lignin	Hoffmann, Laurent, et al. "Silencing of hydroxycinnamoyl-coenzyme A shikimate/quinate hydroxycinnamoyltransferase affects phenylpropanoid biosynthesis." The Plant Cell 16.6 (2004): 1446-1465.
Solyc09g083360.3	uncharacterized protein LOC101255829	N/A	N/A	N/A	N/A	N/A
Solyc10g080370.1	N/A	N/A	N/A	N/A	N/A	N/A
Solyc01g057770.3	boron transporter 1 isoform X1	BOR1	AT2G47160	Efflux-type boron transporter for xylem loading Boron is essential for maintaining the integrity of plants cell walls	Promoted boron homeostasis; Promoted plant cell wall development and integrity	Noguchi, Kyotaro, et al. "bor1-1, an Arabidopsis thaliana mutant that requires a high level of boron." Plant Physiology 115.3 (1997): 901-906.
Solyc09g008060.3	UDP-glycosyltransferase 86A1-like	UGT86A1	AT2G36970	UDP-glycosyltransferase activity	N/A	Pischke, Melissa S., et al. "A transcriptome-based characterization of habituation in plant tissue culture." Plant Physiology 140.4 (2006): 1255-1278.
Solyc09g066360.1	ethylene-response factor C3	ERF.C.3	N/A	Acts downstream of MYC2 in the jasmonate-mediated response to infection	Increased ethylene-responsive signaling; Promoted jasmonate-mediated defense against pathogens	Du, Minmin, et al. "MYC2 orchestrates a hierarchical transcriptional cascade that regulates jasmonate-mediated plant immunity in tomato." The Plant Cell 29.8 (2017): 1883-1906.
Solyc04g071770.3	ethylene-responsive transcription factor ABR1-like	ABR1	AT5G64750	Negative regulator of the ABA signaling pathway involved in seed germination and in response to stressors	Increased ABA signaling; Increased response to stress and pathogenesis	Pandey, Girdhar K., et al. "ABR1, an APETALA2-domain transcription factor that functions as a repressor of ABA response in Arabidopsis." Plant Physiology 139.3 (2005): 1185-1193.
Solyc06g072460.1	uncharacterized protein LOC101268692	N/A	N/A	N/A	N/A	N/A
Solyc10g054440.2	arginine decarboxylase 1	ADC1	AT2G16500	Catalyzes the first step of polyamine biosynthesis to produce putrescine from arginine; Controls polyamine homeostasis which is crucial for normal plant growth and development	Increased freezing tolerance; Increased seed production; Promoted growth/development	Hanfrey, Colin, et al. "Arabidopsis polyamine biosynthesis: absence of ornithine decarboxylase and the mechanism of arginine decarboxylase activity." The Plant Journal 27.6 (2001): 551-560.

TomatoID	NCBI Protein Name	Gene ID	*A. Thaliana* Homolog	Uniprot Description	Putative Consequences for Infection	Citation
Solyc01g079200.3	gibberellin 2-oxidase	GA2OX	N/A	Oxidoreductase activity	N/A	Blackman, Benjamin K., et al. "Sunflower domestication alleles support single domestication center in eastern North America." Proceedings of the National Academy of Sciences 108.34 (2011): 14360-14365.
Solyc03g093610.1	ethylene response factor A.2	ERF2	N/A	Involved in defense response; Involved in intracellular signal transduction; Involved in lipid metabolism	Increased defense response; Increased intracellular signal transduction; Increased lipid metabolism	Zhang, Zhijin, et al. "Transcriptional regulation of the ethylene response factor LeERF2 in the expression of ethylene biosynthesis genes controls ethylene production in tomato and tobacco." Plant Physiology 150.1 (2009): 365-377.
Solyc04g079360.1	transcription factor MYB44-like	MYB44	AT5G67300	Represses the expression of protein phosphatases 2C in response to ABA; Auxin-responsive; Promotes SA-mediated defense, but represses JA-mediated defense	Increases response to ABA; Increased response to auxin signaling; Increases SA-mediated defense; Decreased JA-mediated defense	Riechmann, José Luis, et al. "Arabidopsis transcription factors: genome-wide comparative analysis among eukaryotes." Science 290.5499 (2000): 2105-2110.
Solyc09g091600.3	protein SULFUR DEFICIENCY-INDUCED 2	AT1G04770	AT1G04770	Involved in the utilization of stored sulfate under sulfur-deficient conditions	Increased storage of sulfate; Increased response to sulfur deficiency	Hanada, Kousuke, et al. "Functional compensation of primary and secondary metabolites by duplicate genes in Arabidopsis thaliana." Molecular biology and evolution 28.1 (2011): 377-382.
Solyc02g089685.1	N/A	N/A	N/A	N/A	N/A	N/A
Solyc00g011160.2	N/A	N/A	N/A	N/A	N/A	N/A
Solyc12g005640.2	transcription factor MYB14-like	MYB14	AT2G31180	Transcription activator that regulates freezing tolerance by affecting expression of CBF genes	Promoted regulation of freezing tolerance	Chen, Yan, et al. "AtMYB14 regulates cold tolerance in Arabidopsis." Plant molecular biology reporter 31.1 (2013): 87-97.
Solyc12g042073.1	N/A	N/A	N/A	N/A	N/A	N/A
Solyc06g083440.3	cytochrome b5-like	CB5LP	AT1G60660	Electron transfer activity; Heme binding; Metal ion binding	Impaired methionine catabolism	Rébeillé, Fabrice, et al. "Methionine catabolism in Arabidopsis cells is initiated by a γ-cleavage process and leads to S-methylcysteine and isoleucine syntheses." Proceedings of the National Academy of Sciences 103.42 (2006): 15687-15692.
Solyc08g008280.3	probable WRKY transcription factor 53	WRKY53	AT4G23810	Transcription factor regulating the early events of leaf senescence; Negatively regulates the expression of ESR/ESP; Promotes resistance to certain pathogens by enhancing SA- dependent genes; Contributes to the suppression of PDF1.2	Down regulation of ESR/ESP; Promoted resistance to certain pathogens by enhancing SA- dependent genes; Suppression of PDF1.2	Miao, Ying, and Ulrike Zentgraf. "The antagonist function of Arabidopsis WRKY53 and ESR/ESP in leaf senescence is modulated by the jasmonic and salicylic acid equilibrium." The Plant Cell 19.3 (2007): 819-830.
Solyc10g075100.2	non-specific lipid transfer protein precursor	N/A	N/A	Plays a role in wax/cutin deposition in the cell walls of expanding epidermal cells and certain secretory tissues	Promoted cell wall expansion	Xie, Wan-Qin, et al. "The effects of calmodulin on the lipid-binding activity of CaM-binding protein-10 and maize non-specific lipid transfer protein." Zhi wu Sheng li yu fen zi Sheng wu xue xue bao= Journal of Plant Physiology and Molecular Biology 32.6 (2006): 679-684.

TomatoID	NCBI Protein Name	Gene ID	*A. Thaliana* Homolog	Uniprot Description	Putative Consequences for Infection	Citation
Solyc07g008560.3	probable inactive purple acid phosphatase 27	PAP27	AT5G50400	Acid phosphatase activity; Metal ion binding	N/A	Cheng, Chia-Yi, et al. "Araport11: a complete reannotation of the Arabidopsis thaliana reference genome." The Plant Journal 89.4 (2017): 789-804.
Solyc01g098910.3	peroxisomal adenine nucleotide carrier 1	PNC1	N/A	Required for the conversion of seed-reserved triacylglycerols into sucrose; Necessary for growth before the onset of photosynthesis	Increased conversion of seed-reserved triacylglycerols; Promoted growth over storage	Arai, Yuko, Makoto Hayashi, and Mikio Nishimura. "Proteomic identification and characterization of a novel peroxisomal adenine nucleotide transporter supplying ATP for fatty acid β-oxidation in soybean and Arabidopsis." The Plant Cell 20.12 (2008): 3227-3240.
Solyc04g054990.3	PLAT domain-containing protein 2	PLAT2	AT2G22170	Involved in response to abiotic stress	Promoted response to abiotic stress	Giacomelli, Lisa, Andrea Rudella, and Klaas Jan van Wijk. "High light response of the thylakoid proteome in Arabidopsis wild type and the ascorbate-deficient mutant vtc2-2. A comparative proteomics study." Plant Physiology 141.2 (2006): 685-701.
Solyc09g089500.3	proteinase inhibitor I-B-like isoform X2	LOC107794480	N/A	Involved in response to wounding	Promoted response to wounding	Sierro, Nicolas, et al. "The tobacco genome sequence and its comparison with those of tomato and potato." Nature communications 5.1 (2014): 1-9.
Solyc03g080190.3	protein DMR6-like oxygenase	DLO1	AT4G10500	Component of negative feedback regulation of SA during senescence; Negative regulator of defense against certain pathogens	Negative system regulation of SA; Promoted leaf senescence; Down regulaton of defense against certain pathogens	Zeilmaker, Tieme, et al. "DOWNY MILDEW RESISTANT 6 and DMR 6-LIKE OXYGENASE 1 are partially redundant but distinct suppressors of immunity in Arabidopsis." The Plant Journal 81.2 (2015): 210-222.
Solyc05g050800.3	phosphoglycerate mutase family protein	N/A	N/A	N/A	N/A	N/A
Solyc02g091180.1	uncharacterized protein LOC104645686	N/A	N/A	N/A	N/A	N/A
Solyc07g042230.1	ethylene-responsive transcription factor ERF018-like	ERF018	AT1G74930	Binds to the GCC-box pathogenesis-related promoter element; Regulates gene expression under stress	Increased response to stress and pathogenesis	Riechmann, José Luis, et al. "Arabidopsis transcription factors: genome-wide comparative analysis among eukaryotes." Science 290.5499 (2000): 2105-2110.
Solyc12g011030.2	xyloglucan endotransglucosylase-hydrolase 9 precursor	XTH9	AT4G03210	Essential constituent of the primary cell wall; Participates in cell wall construction of growing tissues	Promoted plant cell wall elongation	Hyodo, Hideki, et al. "Active gene expression of a xyloglucan endotransglucosylase/hydrolase gene, XTH9, in inflorescence apices is related to cell elongation in Arabidopsis thaliana." Plant molecular biology 52.2 (2003): 473-482.
Solyc03g117270.1	F-box protein At5g07610	AT5G07610	AT5G07610	N/A	N/A	Cheng, Chia-Yi, et al. "Araport11: a complete reannotation of the Arabidopsis thaliana reference genome." The Plant Journal 89.4 (2017): 789-804.
Solyc04g009440.3	NAC domain protein	NAC002	AT1G01720	Involved in cellular response to hypoxia; Involved in regulation of ABA-activated signaling pathway; Involved in response to wounding	Increased cellular response to hypoxia; Increased in regulation of ABA-activated signaling pathway; Increased in response to wounding	"Molecular characterization of Brassica napus NAC domain transcriptional activators induced in response to biotic and abiotic stress."

TomatoID	NCBI Protein Name	Gene ID	*A. Thaliana* Homolog	Uniprot Description	Putative Consequences for Infection	Citation
Solyc02g064680.3	calcium-transporting ATPase 2, plasma membrane-type-like	ACA2	AT4G37640	Catalyzes the hydrolysis of ATP coupled with the translocation of calcium from the cytosol into the endoplasmic reticulum	Increased ATP-dependent translocation of calcium into the endoplasmic reticulum	Harper, Jeffrey F., et al. "A novel calmodulin-regulated Ca2+-ATPase (ACA2) from Arabidopsis with an N-terminal autoinhibitory domain." Journal of Biological Chemistry 273.2 (1998): 1099-1106.
Solyc05g009470.3	alpha-xylosidase 1	XYL1	AT1G68560	Glycoside hydrolase releasing xylosyl residues from xyloglucan oligosaccharides; Essential for growth/development	Promoted growth/development	Sampedro, Javier, et al. "Cloning and expression pattern of a gene encoding an α-xylosidase active against xyloglucan oligosaccharides from Arabidopsis." Plant Physiology 126.2 (2001): 910-920.
Solyc06g074420.1	cytochrome P450 94C1	CYP94C1	AT2G27690	Hydroxylase involved in the oxidation of JA-Ile; Exerts negative feedback control on JA-Ile levels and plays a key role in attenuation to jasmonate	Promoted attenuation to JA-Ile and response to jasmonate	Kandel, Sylvie, et al. "Characterization of a methyl jasmonate and wounding-responsive cytochrome P450 of Arabidopsis thaliana catalyzing dicarboxylic fatty acid formation in vitro." The FEBS journal 274.19 (2007): 5116-5127.
Solyc11g027760.1	uncharacterized protein LOC104645327	N/A	N/A	N/A	N/A	N/A
Solyc01g109710.3	protein response to dessication 2	Rd2	N/A	N/A	N/A	Tomato Genome Consortium. "The tomato genome sequence provides insights into fleshy fruit evolution." Nature 485.7400 (2012): 635.
Solyc03g122190.3	salt responsive protein 1	SISRG1	N/A	Involved in regulation of defense; Involved in regulation of JA-mediated signaling pathway	Promoted regulation of defense; Promoted regulation of JA-mediated signaling pathway	Ouyang, Bo, et al. "Identification of early salt stress response genes in tomato root by suppression subtractive hybridization and microarray analysis." Journal of experimental botany 58.3 (2007): 507-520.
Solyc01g087020.2	N/A	N/A	N/A	N/A	N/A	N/A
Solyc03g123620.4	pectinesterase 3	PME3	AT3G14310	Acts in the modification of cell walls via demethylesterification of cell wall pectin	Increased modification of plant cell walls	N/A
Solyc10g081570.2	ABC transporter F family member 4	ABCF4	AT3G54540	Involved in drought and pathogen resistance	Increased resistance to drought and pathogens	Kaundal, Amita, et al. "GENERAL CONTROL NONREPRESSIBLE4 degrades 14-3-3 and the RIN4 complex to regulate stomatal aperture with implications on nonhost disease resistance and drought tolerance." The Plant Cell 29.9 (2017): 2233-2248.
Solyc08g007840.2	ethylene-responsive transcription factor ERF027-like	ERF027	AT1G12630	Binds to the GCC-box pathogenesis-related promoter element; Regulates gene expression under stress	Increased response to stress and pathogenesis	Riechmann, José Luis, et al. "Arabidopsis transcription factors: genome-wide comparative analysis among eukaryotes." Science 290.5499 (2000): 2105-2110.
Solyc02g080190.3	nuclear transport factor 2B	NTF2B	AT1G27970	Part of a multicomponent system of cytosolic factors that assemble at the pore complex during nuclear import	Increased protein transport into the nucleus	Zhao, Qiao, et al. "Identification and characterization of the Arabidopsis orthologs of nuclear transport factor 2, the nuclear import factor of ran." Plant physiology 140.3 (2006): 869-878.

TomatoID	NCBI Protein Name	Gene ID	*A. Thaliana* Homolog	Uniprot Description	Putative Consequences for Infection	Citation
Solyc09g018250.2	GBF-interacting protein 1-like isoform X1	GIP1	AT3G13222	Contributes to bZIP-mediated gene regulation; Acts as negative co-regulator in red and blue light-mediated hypocotyl elongation	Promoted gene regulation; Increased seed germination	Sehnke, Paul C., et al. "Identification and characterization of GIP1, an Arabidopsis thaliana protein that enhances the DNA binding affinity and reduces the oligomeric state of G-box binding factors." Cell research 15.8 (2005): 567-575.
Solyc03g026270.3	dehydration-responsive element-binding protein 1A	DREB1A	AT4G25480	Transcriptional activator that binds specifically to the C-repeat/DRE element mediating cold-inducible transcription	Increased cold acclimation and freezing tolerance	Sakuma, Yoh, et al. "DNA-binding specificity of the ERF/AP2 domain of Arabidopsis DREBs, transcription factors involved in dehydration-and cold-inducible gene expression." Biochemical and biophysical research communications 290.3 (2002): 998-1009.
Solyc12g099000.2	S-adenosylmethionine synthase 2	SAM2	AT4G01850	Involved in SA-mediated defense	Increased S-adenosylmethionine production; Promoted early defense response	Rajjou, Loïc, et al. "Proteomic investigation of the effect of salicylic acid on Arabidopsis seed germination and establishment of early defense mechanisms." Plant physiology 141.3 (2006): 910-923.
Solyc06g007120.3	PI-phospholipase C PLC4	PLC4	AT5G58700	Involved in the production of the second messenger molecules diacylglycerol and inositol 1,4,5-trisphosphate	Impaired production of secondary messenger molecules	Hunt, L., et al. "Gene-specific expression and calcium activation of Arabidopsis thaliana phospholipase C isoforms." New Phytologist 162.3 (2004): 643-654.
Solyc02g071610.3	GDSL esterase/lipase At5g45670	AT5G45670	AT5G45670	Involved in lipid catabolism	Increased lipid catabolism	Rouhier, Nicolas, et al. "Identification of plant glutaredoxin targets." Antioxidants & redox signaling 7.7-8 (2005): 919-929.
Solyc02g071720.2	GDSL esterase/lipase At1g29670-like	AT1G29670	AT1G29670	Hydralose/esterase activity	N/A	Sierro, Nicolas, et al. "The tobacco genome sequence and its comparison with those of tomato and potato." Nature communications 5.1 (2014): 1-9.
Solyc04g082030.1	ornithine decarboxylase	ODC	N/A	Catalyzes the first and rate-limiting step of polyamine biosynthesis that converts ornithine into putrescine, which is the precursor for the polyamines, spermidine, and spermine	Increased production of polyamines, spermidine, and spermine	Alabadı, David, and Juan Carbonell. "Expression of ornithine decarboxylase is transiently increased by pollination, 2, 4-dichlorophenoxyacetic acid, and gibberellic acid in tomato ovaries." Plant physiology 118.1 (1998): 323-328.
Solyc02g067610.1	N/A	N/A	N/A	N/A	N/A	N/A
Solyc07g005330.3	beta-glucosidase 08 precursor	BGLU8	AT3G62750	Catalyzes hydrolysis of terminal, non-reducing beta-D-glucosyl residues with release of beta-D-glucose	Increased cellulose production; Promoted plant growth/development	Thieme, Christoph J., et al. "Endogenous Arabidopsis messenger RNAs transported to distant tissues." Nature Plants 1.4 (2015): 1-9.
Solyc09g062970.1	glycine-rich protein 5-like	GRP5	AT3G20470	Involved in organ growth by promoting cell elongation processes	Promoted cell elongation	Mangeon, Amanda, et al. "AtGRP5, a vacuole-located glycine-rich protein involved in cell elongation." Planta 230.2 (2009): 253-265.
Solyc02g085495.1	N/A	N/A	N/A	N/A	N/A	N/A

TomatoID	NCBI Protein Name	Gene ID	*A. Thaliana* Homolog	Uniprot Description	Putative Consequences for Infection	Citation
Solyc01g080010.2	xyloglucan-specific fungal endoglucanase inhibitor protein precursor	N/A	N/A	N/A	N/A	N/A
Solyc04g011790.1	monothiol glutaredoxin-S1-like	GRXS1	AT1G03020	Reduces GSH-thiol disulfides	Increased reduction of GSH-thiol disulfides	Patterson, Kurt, et al. "Nitrate-regulated glutaredoxins control Arabidopsis primary root growth." Plant Physiology 170.2 (2016): 989-999.
Solyc09g006010.2	pathogenesis-related leaf protein 4	N/A	N/A	Involved in defense response against pathogens	Promoted defense against pathogens	van Kan, Jan AL, et al. "Differential accumulation of mRNAs encoding extracellular and intracellular PR proteins in tomato induced by virulent and avirulent races of Cladosporium fulvum." Plant molecular biology 20.3 (1992): 513-527.
Solyc10g083970.1	S-adenosylmethionine synthase 3-like	METK3	AT2G36880	Catalyzes the formation of S-adenosylmethionine from methionine; Involved in SA-mediated defense; Involved in the biosynthesis of lignin	Increased S-adenosylmethionine production; Promoted early defense response; Increased lignin biosynthesis	Goto, Derek B., et al. "A single-nucleotide mutation in a gene encoding S-adenosylmethionine synthetase is associated with methionine over-accumulation phenotype in Arabidopsis thaliana." Genes & genetic systems 77.2 (2002): 89-95.
Solyc12g008350.2	dehydration responsive element binding protein	DREB	N/A	Transcriptional activator that binds specifically to the C-repeat/DRE element mediating cold-inducible transcription	Increased cold acclimation and freezing tolerance	Alonso-Blanco, Carlos, et al. "Genetic and molecular analyses of natural variation indicate CBF2 as a candidate gene for underlying a freezing tolerance quantitative trait locus in Arabidopsis." Plant Physiology 139.3 (2005): 1304-1312.
Solyc03g007230.3	protein phosphatase 2C 51-like	AT3G63340	AT3G63340	Metal ion binding; Protein serine/threonine phosphatase activity	N/A	Cheng, Chia-Yi, et al. "Araport11: a complete reannotation of the Arabidopsis thaliana reference genome." The Plant Journal 89.4 (2017): 789-804.
Solyc01g091360.3	uncharacterized protein LOC101253424	N/A	N/A	N/A	N/A	N/A
Solyc12g057150.1	classical arabinogalactan protein 5	AGP5	AT1G35230	Proteoglycan involved in differentiation, cell-cell recognition, embryogenesis, and programmed cell death	Promoted plant growth/development and cell differentiation; Promoted programmed cell death	Thieme, Christoph J., et al. "Endogenous Arabidopsis messenger RNAs transported to distant tissues." Nature Plants 1.4 (2015): 1-9.
Solyc02g083850.3	calcium-dependent protein kinase 18-like	CPK18	AT4G36070	Plays a role in signal transduction pathways that involve calcium as a second messenger	Promoted calcium-dependent signaling	Cheng, Shu-Hua, et al. "Calcium signaling through protein kinases. The Arabidopsis calcium-dependent protein kinase gene family." Plant physiology 129.2 (2002): 469-485.
Solyc07g008380.2	salutaridinol 7-O-acetyltransferase-like	SALAT	N/A	Involved in biosynthesis of morphinan-type benzylisoquinoline alkaloids	Increased in biosynthesis of morphinan-type benzylisoquinoline alkaloids	Lenz, Rainer, and Meinhart H. Zenk. "Acetyl coenzyme A: salutaridinol-7-O-acetyltransferase from Papaver somniferum plant cell cultures: The enzyme catalyzing the formation of thebaine in morphine biosynthesis." Journal of Biological Chemistry 270.52 (1995): 31091-31096.

TomatoID	NCBI Protein Name	Gene ID	*A. Thaliana* Homolog	Uniprot Description	Putative Consequences for Infection	Citation
Solyc00g009760.2	uncharacterized protein LOC101246232	N/A	N/A	N/A	N/A	N/A
Solyc02g064980.1	mitogen-activated protein kinase kinase kinase 18-like	MAPKKK18	AT1G05100	Act as ABA signal transducer under abiotic stress; Promotes stomatal growth/development; Inhibits germination and root growth; Promotes leaf senescence	Increased response to stress; Increased reproductive development; Decreased growth/development; Increased leaf senescence	Mitula, Filip, et al. "Arabidopsis ABA-activated kinase MAPKKK18 is regulated by protein phosphatase 2C ABI1 and the ubiquitin–proteasome pathway." Plant and Cell Physiology 56.12 (2015): 2351-2367.
Solyc06g075010.3	ruBisCO large subunit-binding protein subunit alpha, chloroplastic	N/A	N/A	Involved in protein refolding	N/A	N/A
Solyc10g086690.2	phosphatidylinositol:ceramide inositolphosphotransferase 2	IPCS2	AT2G37940	Essential for sphingolipid biosynthesis; Plays an important role in modulating plant programmed cell death	Increased sphingolipid biosynthesis; Promoted programmed cell death	Wang, Wenming, et al. "An inositolphosphorylceramide synthase is involved in regulation of plant programmed cell death associated with defense in Arabidopsis." The Plant Cell 20.11 (2008): 3163-3179.
Solyc05g008370.1	probable ribose-5-phosphate isomerase 2	RPI2	AT2G01290	Involved in programmed cell death; Involved in vegetative-to-reproductive phase transition in meristems	Increased primary metabolism; Promoted programmed cell death; Promoted transition from vegetative-to-reproductive phase	Xiong, Yuqing, et al. "Deficiency in a cytosolic ribose-5-phosphate isomerase causes chloroplast dysfunction, late flowering and premature cell death in Arabidopsis." Physiologia plantarum 137.3 (2009): 249-263.
Solyc06g024210.2	uncharacterized protein LOC101254183	N/A	N/A	N/A	N/A	N/A
Solyc04g016190.1	zeatin O-glucosyltransferase-like	ZOG1	AT2G36750	Regulates cytokinin activity and storage; Impacts seed growth	Increased cellular division and plant growth in roots and shoots; Increased reproductive investment	Hou, Bingkai, et al. "N-glucosylation of cytokinins by glycosyltransferases of Arabidopsis thaliana." Journal of Biological Chemistry 279.46 (2004): 47822-47832.
Solyc07g008550.3	probable inactive purple acid phosphatase 27	PAP27	AT5G50400	Acid phosphatase activity; Metal ion binding	N/A	Cheng, Chia-Yi, et al. "Araport11: a complete reannotation of the Arabidopsis thaliana reference genome." The Plant Journal 89.4 (2017): 789-804.
Solyc03g031620.3	5'-adenylylsulfate reductase 3, chloroplastic-like	APR3	AT4G21990	Reduces sulfate for Cys biosynthesis	Increased Cys biosynthesis	Tsakraklides, George, et al. "Sulfate reduction is increased in transgenic Arabidopsis thaliana expressing 5′-adenylylsulfate reductase from Pseudomonas aeruginosa." The Plant Journal 32.6 (2002): 879-889.
Solyc06g068650.3	4-coumarate--CoA ligase	4CL1	AT1G51680	Produces CoA thioesters of a variety of hydroxy- and methoxy-substituted cinnamic acids, which are used to synthesize several phenylpropanoid-derived compounds	Increased production of phenylproanoid-derived compounds	Ehlting, Jürgen, Jane JK Shin, and Carl J. Douglas. "Identification of 4-coumarate: coenzyme A ligase (4CL) substrate recognition domains." The Plant Journal 27.5 (2001): 455-465.
Solyc09g075890.3	uncharacterized protein LOC101255829	N/A	N/A	N/A	N/A	N/A

TomatoID	NCBI Protein Name	Gene ID	*A. Thaliana* Homolog	Uniprot Description	Putative Consequences for Infection	Citation
Solyc10g079350.2	zeatin O-glucosyltransferase-like	ZOG1	AT2G36750	Regulates cytokinin activity and storage; Impacts seed growth	Increased cellular division and plant growth in roots and shoots; Increased reproductive investment	Hou, Bingkai, et al. "N-glucosylation of cytokinins by glycosyltransferases of Arabidopsis thaliana." Journal of Biological Chemistry 279.46 (2004): 47822-47832.
Solyc04g074470.1	protein EXORDIUM-like 2	EXL2	AT5G64260	Plays a role in a brassinosteroid-dependent regulation of growth/development	Increased regulation of leaf and root growth/development	Schröder, Florian, et al. "The extracellular EXO protein mediates cell expansion in Arabidopsis leaves." BMC plant biology 9.1 (2009): 20.
Solyc08g062330.3	ankyrin repeat-containing protein ITN1-like	ITN1	AT3G12360	Involved in salt stress tolerance; Acts through ABA signaling pathways and promotes ROS production	Increased salt stress tolerance; Increased ROS production	Sakamoto, Hikaru, Osamu Matsuda, and Koh Iba. "ITN1, a novel gene encoding an ankyrin-repeat protein that affects the ABA-mediated production of reactive oxygen species and is involved in salt-stress tolerance in Arabidopsis thaliana." The Plant Journal 56.3 (2008): 411-422.
Solyc12g099400.2	uncharacterized protein LOC101264805	N/A	N/A	N/A	N/A	N/A
Solyc01g105890.3	linalool synthase	N/A	N/A	N/A	N/A	N/A
Solyc06g068960.1	calcium-binding allergen Ole e 8	N/A	N/A	N/A	N/A	Ledesma, Amalia, Mayte Villalba, and Rosalía Rodríguez. "Cloning, expression and characterization of a novel four EF-hand Ca2+-binding protein from olive pollen with allergenic activity." FEBS letters 466.1 (2000): 192-196.
Solyc08g068680.3	aromatic amino acid decarboxylase 1A	AADC1A	N/A	Involved in carboxylic acid metabolic process	Increased carboxylic acid metabolism	Tieman, Denise, et al. "Tomato aromatic amino acid decarboxylases participate in synthesis of the flavor volatiles 2-phenylethanol and 2-phenylacetaldehyde." Proceedings of the National Academy of Sciences 103.21 (2006): 8287-8292.
Solyc02g032860.3	5'-adenylylsulfate reductase 3, chloroplastic	APR3	AT4G21990	Reduces sulfate for Cys biosynthesis	Increased Cys biosynthesis	Tsakraklides, George, et al. "Sulfate reduction is increased in transgenic Arabidopsis thaliana expressing 5′-adenylylsulfate reductase from Pseudomonas aeruginosa." The Plant Journal 32.6 (2002): 879-889.
Solyc02g070040.1	uncharacterized protein LOC101257038	N/A	N/A	N/A	N/A	N/A
Solyc03g083680.1	N/A	N/A	N/A	N/A	N/A	N/A
Solyc05g010420.2	S-adenosylmethionine decarboxylase	N/A	N/A	Adenosylmethionine decarboxylase activity	N/A	N/A
Solyc12g097000.2	TMV resistance protein N-like	N	N/A	Disease resistance protein; Triggers plant defense systems, including the hypersensitive response	Increased hypersensitive response	Dinesh-Kumar, S. P., and Barbara J. Baker. "Alternatively spliced N resistance gene transcripts: their possible role in tobacco mosaic virus resistance." Proceedings of the National Academy of Sciences 97.4 (2000): 1908-1913.

TomatoID	NCBI Protein Name	Gene ID	*A. Thaliana* Homolog	Uniprot Description	Putative Consequences for Infection	Citation
Solyc00g028960.1	monothiol glutaredoxin-S1-like	GRXS1	AT1G03020	Reduces GSH-thiol disulfides	Increased reduction of GSH-thiol disulfides	Patterson, Kurt, et al. "Nitrate-regulated glutaredoxins control Arabidopsis primary root growth." Plant Physiology 170.2 (2016): 989-999.
Solyc02g077110.3	phospholipase A1-II 1-like	OsI_03083	N/A	Acylhydrolase that catalyzes the hydrolysis of phospholipids at the sn-1 position; Involved in lipid catabolism	Increased lipid catabolism	Yu, Jun, et al. "The genomes of Oryza sativa: a history of duplications." PLoS Biol 3.2 (2005): e38.
Solyc03g111100.1	uncharacterized protein LOC101267981	N/A	N/A	N/A	N/A	N/A
Solyc04g008330.1	zeatin O-glucosyltransferase-like	ZOG1	AT2G36750	Regulates cytokinin activity and storage; Impacts seed growth	Increased cellular division and plant growth in roots and shoots; Increased reproductive investment	Hou, Bingkai, et al. "N-glucosylation of cytokinins by glycosyltransferases of Arabidopsis thaliana." Journal of Biological Chemistry 279.46 (2004): 47822-47832.
Solyc06g082440.1	CBL-interacting protein kinase 11	CIPK11	AT2G30360	Acts as a negative regulator of the plasma membrane proton pump AHA2 by preventing its interaction with 14-3-3 protein	Increased activity of plasma membrane proton pump	Fuglsang, Anja T., et al. "Arabidopsis protein kinase PKS5 inhibits the plasma membrane H+-ATPase by preventing interaction with 14-3-3 protein." The Plant Cell 19.5 (2007): 1617-1634.
Solyc08g074683.1	N/A	N/A	N/A	N/A	N/A	N/A
Solyc03g098720.3	kunitz-type serine protease inhibitor DrTI-like	N/A	N/A	Serine protease inhibitor that inhibits chymotripsin	Increased protein degradation	Chang, Long-sen, et al. "Purification and characterization of a chymotrypsin inhibitor from the venom of Ophiophagus hannah (King Cobra)." Biochemical and biophysical research communications 283.4 (2001): 862-867.
Solyc03g120660.3	uncharacterized protein LOC101263391	N/A	N/A	N/A	N/A	N/A
Solyc07g018010.3	transcription factor bHLH35	BHLH35	AT5G57150	Involved in regulation of transcription	N/A	Heim, Marc A., et al. "The basic helix–loop–helix transcription factor family in plants: a genome-wide study of protein structure and functional diversity." Molecular biology and evolution 20.5 (2003): 735-747.
Solyc07g062700.3	sodium/calcium exchanger NCL	NCL	AT1G53210	Participates in the maintenance of calcium homeostasis; Plays a role in auxin response, diurnal rhythm, and flowering time; Involved in salt stress response	Promoted calcium homeostasis; Increased response to auxin; Promoted circadian rhythm; Promoted salt stress response	Wang, Peng, et al. "A Na+/Ca2+ exchanger-like protein (AtNCL) involved in salt stress in Arabidopsis." Journal of Biological Chemistry 287.53 (2012): 44062-44070.
Solyc08g068770.2	N-hydroxycinnamoyl-CoA:tyramine N-hydroxycinnamoyl transferase THT1-3	THT1-3	N/A	Involved in the production of p-Coumaroylnoradrenaline	Increased defense against pathogens	von Roepenack-Lahaye, Edda, et al. "p-Coumaroylnoradrenaline, a novel plant metabolite implicated in tomato defense against pathogens." Journal of Biological Chemistry 278.44 (2003): 43373-43383.
Solyc08g080585.1	N/A	N/A	N/A	N/A	N/A	N/A

TomatoID	NCBI Protein Name	Gene ID	*A. Thaliana* Homolog	Uniprot Description	Putative Consequences for Infection	Citation
Solyc08g080590.3	thaumatin-like protein	TLP1	AT4G24180	Involved in local responses of roots to colonization by non-pathogenic plant growth-promoting rhizobacteria	Increased colonization of growth-promoting rhizobacteria	Leon-Kloosterziel, Karen M., et al. "Colonization of the Arabidopsis rhizosphere by fluorescent Pseudomonas spp. activates a root-specific, ethylene-responsive PR-5 gene in the vascular bundle." Plant molecular biology 57.5 (2005): 731-748.
Solyc12g099260.2	ATP-citrate synthase beta chain protein 2-like	ACLA-2	AT1G60810	Used for the elongation of fatty acids and biosynthesis of isoprenoids, flavonoids, and malonated derivatives; Required for normal growth and development in seeds	Promoted seed development; Increased production of primary metabolites	Fatland, Beth L., Basil J. Nikolau, and Eve Syrkin Wurtele. "Reverse genetic characterization of cytosolic acetyl-CoA generation by ATP-citrate lyase in Arabidopsis." The Plant Cell 17.1 (2005): 182-203.
Solyc02g089900.1	lysM domain receptor-like kinase 4	LYK4	AT2G23770	Lysin motif receptor kinase that functions as a cell surface receptor in chitin elicitor signaling leading to innate immunity aganst certain fungal and bacterial pathogens	Promoted innate immunity; Increased resistance to certain fungal and bacterial pathogens	Wan, Jinrong, et al. "LYK4, a lysin motif receptor-like kinase, is important for chitin signaling and plant innate immunity in Arabidopsis." Plant physiology 160.1 (2012): 396-406.
Solyc02g093250.3	caffeoyl-CoA O-methyltransferase-like	CCOAOMT1	AT4G34050	Involved in the reinforcement of the plant cell wall; Involved in response to wounding or pathogen challenge by promoting the formation of cell wall-bound ferulic acid polymers	Increased production of polysaccharides; Reinforced cell wall; Increased response to wounding and pathogens	Do, Cao-Trung, et al. "Both caffeoyl Coenzyme A 3-O-methyltransferase 1 and caffeic acid O-methyltransferase 1 are involved in redundant functions for lignin, flavonoids and sinapoyl malate biosynthesis in Arabidopsis." Planta 226.5 (2007): 1117-1129.
Solyc04g076307.1	N/A	N/A	N/A	N/A	N/A	N/A
Solyc06g062920.3	auxin-regulated dual specificity cytosolic kinase	N/A	N/A	N/A	N/A	N/A
Solyc01g096430.3	NAD(P)H:quinone oxidoreductase	NQR	AT3G27890	Serves as a quinone reductase in connection with conjugation reactions of hydroquinones involved in detoxification	Increased detoxification	Sparla, Francesca, et al. "Cloning and heterologous expression of NAD(P)H: quinone reductase of Arabidopsis thaliana, a functional homologue of animal DT-diaphorase." FEBS letters 463.3 (1999): 382-386.
Solyc04g011840.1	monothiol glutaredoxin-S1-like	GRXS1	AT1G03020	Reduces GSH-thiol disulfides	Increased reduction of GSH-thiol disulfides	Patterson, Kurt, et al. "Nitrate-regulated glutaredoxins control Arabidopsis primary root growth." Plant Physiology 170.2 (2016): 989-999.
Solyc10g075160.1	ferredoxin	AT1G32550	AT1G32550	Ferredoxins are iron-sulfur proteins that transfer electrons in a wide variety of metabolic reactions	Promoted metabolism	Zapata, Luis, et al. "Chromosome-level assembly of Arabidopsis thaliana Ler reveals the extent of translocation and inversion polymorphisms." Proceedings of the National Academy of Sciences 113.28 (2016): E4052-E4060.
Solyc03g007960.3	Beta-carotene 3-hydroxylase 1, chloroplastic	BETA-OHASE 1	AT4G25700	Nonheme diiron monooxygenase involved in the biosynthesis of xanthophylls	Increased biosynthesis of xanthophylls; Increased pigment production	Sun, Zairen, Elisabeth Gantt, and Francis X. Cunningham. "Cloning and functional analysis of the β-carotene hydroxylase of Arabidopsis thaliana." Journal of Biological Chemistry 271.40 (1996): 24349-24352.

TomatoID	NCBI Protein Name	Gene ID	*A. Thaliana* Homolog	Uniprot Description	Putative Consequences for Infection	Citation
Solyc06g075000.3	uncharacterized protein LOC101265549	N/A	N/A	N/A	N/A	N/A
Solyc10g006700.1	calcium-binding protein PBP1	PBP1	AT5G54490	Potential calcium sensor	Promoted calcium sensing; Promoted regulation of trichome morphogenesis	Reddy, Vaka S., et al. "KIC, a novel Ca2+ binding protein with one EF-hand motif, interacts with a microtubule motor protein and regulates trichome morphogenesis." The Plant Cell 16.1 (2004): 185-200.
Solyc12g088190.2	amino acid permease 6	AAP6	AT5G49630	Involved in the uptake of amino acids diffusing out of the xylem tracheids into the xylem parenchyma	Increased amino acid uptake and mobilization	Okumoto, Sakiko, et al. "High affinity amino acid transporters specifically expressed in xylem parenchyma and developing seeds of Arabidopsis." Journal of Biological Chemistry 277.47 (2002): 45338-45346.
Solyc05g056170.3	phenylalanine ammonia-lyase 2	PAL2	AT3G53260	Enzyme of plant metabolism catalyzing the first reaction in the biosynthesis of a wide variety of natural products	Increased biosynthesis of L-phenylalanine derivatives	Cochrane, Fiona C., Laurence B. Davin, and Norman G. Lewis. "The Arabidopsis phenylalanine ammonia lyase gene family: kinetic characterization of the four PAL isoforms." Phytochemistry 65.11 (2004): 1557-1564.
Solyc09g007260.3	ethylene-responsive transcription factor RAP2-7-like	RAP2-7	AT2G28550	Regulates gene expression under stress; Negatively regulates flowering	Increased response to stress and pathogenesis; Flowering delay	Riechmann, José Luis, et al. "Arabidopsis transcription factors: genome-wide comparative analysis among eukaryotes." Science 290.5499 (2000): 2105-2110.
Solyc01g079940.3	basic 7S globulin	BG	N/A	Seed storage protein; Protein kinase activity; Binds leginsulin	Promoted seed development	Kagawa, Hiroyuki, and Hisashi Hirano. "Sequence of a cDNA encoding soybean basic 7S globulin." Nucleic acids research 17.21 (1989): 8868.
Solyc01g086800.3	histone H3.2	HTR2	AT3G27360	Core component of the nucleosome; Plays central roles in transcription regulation, DNA repair, DNA replication and chromosomal stability	Increased transcription regulation and DNA repair	Seki, Motoaki, et al. "Functional annotation of a full-length Arabidopsis cDNA collection." Science 296.5565 (2002): 141-145.
Solyc01g087570.2	N/A	N/A	N/A	N/A	N/A	N/A
Solyc03g117870.3	4-coumarate--CoA ligase	4CL1	AT1G51680	Produces CoA thioesters of a variety of hydroxy- and methoxy-substituted cinnamic acids	Increased production of phenylproanoid-derived compounds	Ehlting, Jürgen, Jane JK Shin, and Carl J. Douglas. "Identification of 4-coumarate: coenzyme A ligase (4CL) substrate recognition domains." The Plant Journal 27.5 (2001): 455-465.
Solyc08g076980.3	acetylornithine deacetylase-like	AT4G17830	AT4G17830	Involved in flowering and fruit development	Promoted flowering and fruit development	Molesini, Barbara, et al. "Involvement of the putative N-acetylornithine deacetylase from Arabidopsis thaliana in flowering and fruit development." Plant and Cell Physiology 56.6 (2015): 1084-1096.
Solyc02g071620.3	GDSL esterase/lipase At1g29670	AT1G29670	AT1G29670	Hydralose/esterase activity	N/A	Sierro, Nicolas, et al. "The tobacco genome sequence and its comparison with those of tomato and potato." Nature communications 5.1 (2014): 1-9.
Solyc03g005470.3	uncharacterized protein LOC101261585	N/A	N/A	N/A	N/A	N/A
Solyc12g009925.1	N/A	N/A	N/A	N/A	N/A	N/A

TomatoID	NCBI Protein Name	Gene ID	*A. Thaliana* Homolog	Uniprot Description	Putative Consequences for Infection	Citation
Solyc03g112880.1	fasciclin-like arabinogalactan protein 4	FLA4	AT3G46550	Cell surface adhesion protein required for normal cell expansion	Increased cell expansion	Xu, Shou-Ling, et al. "Two leucine-rich repeat receptor kinases mediate signaling, linking cell wall biosynthesis and ACC synthase in Arabidopsis." The Plant Cell 20.11 (2008): 3065-3079.
Solyc07g006480.3	probably inactive leucine-rich repeat receptor-like protein kinase At5g48380	AT5G48380	AT5G48380	Negative regulator of defense response to bacteria; Negative regulator of programmed cell death	Impaired defense response to bacteria; Impaired programmed cell death	Gao, Minghui, et al. "Regulation of cell death and innate immunity by two receptor-like kinases in Arabidopsis." Cell host & microbe 6.1 (2009): 34-44.
Solyc07g043500.1	beta-D-glucosyl crocetin beta-1,6-glucosyltransferase	UGT94E5	N/A	Glucosyltransferase catalyzing crocetin gentiobiosyl esters biosynthesis	Promoted production of crocetin and crocin; Increased pigment production	Nagatoshi, Mai, et al. "UGT75L6 and UGT94E5 mediate sequential glucosylation of crocetin to crocin in Gardenia jasminoides." FEBS letters 586.7 (2012): 1055-1061.
Solyc01g066760.2	uncharacterized protein LOC101255730	N/A	N/A	N/A	N/A	N/A
Solyc01g091465.1	N/A	N/A	N/A	N/A	N/A	N/A
Solyc09g014280.1	uncharacterized acetyltransferase At3g50280	AT3G50280	AT3G50280	N/A	N/A	N/A
Solyc02g092790.3	Classical arabinogalactan protein 1	AGP1	AT5G64310	Proteoglycan implicated in diverse developmental roles such as differentiation, cell-cell recognition, embryogenesis, and programmed cell death	Promoted growth/development	Vergnolle, Chantal, et al. "The cold-induced early activation of phospholipase C and D pathways determines the response of two distinct clusters of genes in Arabidopsis cell suspensions." Plant physiology 139.3 (2005): 1217-1233.
Solyc03g120990.3	NADP-dependent malic enzyme, chloroplastic	ME6	N/A	Cecarboxylates malate shuttled from neighboring mesophyll cells;<br>CO_2 released is refixed by RuBisCo;<br>Eliminates the photorespiratory loss of CO_2 that occurs in most plants	Promoted photorespiratory efficiency of CO_2 loss	Cheng, Yuxiang, et al. "Expression, purification, and characterization of two NADP-malic enzymes of rice (Oryza sativa L.) in Escherichia coli." Protein expression and purification 45.1 (2006): 200-205.
Solyc04g071890.3	peroxidase 12 precursor	PER12	AT1G71695	Involved in removal of H_2O_2, oxidation of toxic reductants, biosynthesis and degradation of lignin, suberization, auxin catabolism, and response to environmental stressors	Increased removal of H_2O_2, oxidation of toxic reductants, biosynthesis and degradation of lignin, suberization, auxin catabolism, and response to environmental stressors	Paynel, Florence, et al. "Temporal regulation of cell-wall pectin methylesterase and peroxidase isoforms in cadmium-treated flax hypocotyl." Annals of botany 104.7 (2009): 1363-1372.
Solyc01g096320.3	homeobox-leucine zipper protein ATHB-12	ATHB-12	AT3G61890	Transcription activator that acts as a growth regulator in response to water deficit	Increased regulation of growth/development; Increased response to drought	Olsson, Anna, Peter Engström, and Eva Söderman. "The homeobox genes ATHB12 and ATHB7encode potential regulators of growth in response to water deficit in Arabidopsis." Plant molecular biology 55.5 (2004): 663-677.
Solyc02g083120.2	uncharacterized protein LOC101250469	N/A	N/A	N/A	N/A	N/A

TomatoID	NCBI Protein Name	Gene ID	*A. Thaliana* Homolog	Uniprot Description	Putative Consequences for Infection	Citation
Solyc04g011830.1	monothiol glutaredoxin-S1-like	GRXS1	AT1G03020	Reduces GSH-thiol disulfides	Increased reduction of GSH-thiol disulfides	Patterson, Kurt, et al. "Nitrate-regulated glutaredoxins control Arabidopsis primary root growth." Plant Physiology 170.2 (2016): 989-999.
Solyc08g067505.1	N/A	N/A	N/A	N/A	N/A	N/A
Solyc11g005150.2	leucine-rich repeat extensin-like protein 6	LRX6	AT3G22800	Modulates cell morphogenesis by regulating cell wall formation and assembly, and/or growth polarization	Promoted cell morphogenesis/regulation of cell wall formation and assembly, and/or growth polarization	Cheng, Hui, et al. "Gibberellin acts through jasmonate to control the expression of MYB21, MYB24, and MYB57 to promote stamen filament growth in Arabidopsis." PLoS Genet 5.3 (2009): e1000440.
Solyc00g010525.1	N/A	N/A	N/A	N/A	N/A	N/A
Solyc02g093270.3	caffeoyl-CoA O-methyltransferase-like	CCOAOMT1	AT4G34050	Involved in the reinforcement of the plant cell wall; Involved in response to wounding or pathogen challenge by promoting the formation of cell wall-bound ferulic acid polymers	Increased production of polysaccharides; Reinforced cell wall; Increased response to wounding and pathogens	Do, Cao-Trung, et al. "Both caffeoyl Coenzyme A 3-O-methyltransferase 1 and caffeic acid O-methyltransferase 1 are involved in redundant functions for lignin, flavonoids and sinapoyl malate biosynthesis in Arabidopsis." Planta 226.5 (2007): 1117-1129.
Solyc03g115380.2	UDP-glucose 6-dehydrogenase 1-like	UGD1	AT1G26570	Involved in the biosynthesis of UDP-glucuronic acid, providing nucleotide sugars for cell-wall polymers	Increased nucleotide transport for cell-wall polymers	Oka, Takuji, and Yoshifumi Jigami. "Reconstruction of de novo pathway for synthesis of UDP-glucuronic acid and UDP-xylose from intrinsic UDP-glucose in Saccharomyces cerevisiae." The FEBS journal 273.12 (2006): 2645-2657.
Solyc03g116510.1	uncharacterized protein LOC104646577	N/A	N/A	N/A	N/A	N/A
Solyc11g006910.2	ferredoxin, root R-B2	N/A	N/A	Ferredoxins are iron-sulfur proteins that transfer electrons in a wide variety of metabolic reactions	Promoted metabolism	Wada, Keishiro, Masaaki Onda, and Hiroshi Matsubara. "Amino acid sequences of ferredoxin isoproteins from radish roots." The Journal of Biochemistry 105.4 (1989): 619-625.
Solyc02g088630.3	probable galacturonosyltransferase 14	GAUT14	AT5G15470	Involved in pectin and/or xylans biosynthesis in cell walls	Increased pectin and xylan biosynthesis; Increased cell wall expansion	Wang, Li, et al. "Arabidopsis galacturonosyltransferase (GAUT) 13 and GAUT14 have redundant functions in pollen tube growth." Molecular plant 6.4 (2013): 1131-1148.
Solyc03g097750.3	translocon-associated protein subunit beta-like	LOC109001308	N/A	N/A	N/A	Martínez-García, Pedro J., et al. "The walnut (Juglans regia) genome sequence reveals diversity in genes coding for the biosynthesis of non-structural polyphenols." The Plant Journal 87.5 (2016): 507-532.
Solyc01g009160.2	harpin-induced1-like	OS12G06210	N/A	N/A	N/A	Sakai, Hiroaki, et al. "Rice Annotation Project Database (RAP-DB): an integrative and interactive database for rice genomics." Plant and Cell Physiology 54.2 (2013): e6-e6.
Solyc07g049370.2	glucan endo-1,3-beta-glucosidase 12	AT4G29360	AT4G29360	Involved in carbohydrate metabolic process, cell wall organization, and plant defense response	Increased carbohydrate metabolism, cell wall organization, and plant defense response	Wu, Qiong, et al. "Long-term balancing selection contributes to adaptation in Arabidopsis and its relatives." Genome biology 18.1 (2017): 1-15.

TomatoID	NCBI Protein Name	Gene ID	*A. Thaliana* Homolog	Uniprot Description	Putative Consequences for Infection	Citation
Solyc09g064820.1	EID1-like F-box protein 3	EDL3	AT3G63060	Involved in the following processes: ABA-activated signaling pathway, regulation of seed germination, response to osmotic stress, response to salt stress, response to water deprivation, and meristem phase transition	Increased ABA-activated signaling; Increased regulation of seed germination; Promoted response to osmotic, salt, and water stress; Promoted transition from vegetative to reproductive phase in the meristem	Friso, Giulia, et al. "In-depth analysis of the thylakoid membrane proteome of Arabidopsis thaliana chloroplasts: new proteins, new functions, and a plastid proteome database." The Plant Cell 16.2 (2004): 478-499.
Solyc02g069110.3	cathepsin B-like protease 2	CATHB2	AT1G02305	Thiol protease that plays a central role in plant programmed cell death; Required for full levels of PCD during resistance gene-mediated hypersensitive response	Increased initiation of programmed cell death; Promoted hypersensitive response	Theologis, Athanasios, et al. "Sequence and analysis of chromosome 1 of the plant Arabidopsis thaliana." Nature 408.6814 (2000): 816-820.
Solyc10g078360.2	uncharacterized protein LOC101265973	N/A	N/A	N/A	N/A	N/A
Solyc06g060690.2	non-functional pseudokinase ZED1 isoform X1	ZED1	AT3G57750	Involved in the regulation of the ambient temperature-sensitive intersection of growth and immune response	Repressed regulation of temperature-sensitive intersection of growth and immune response	Lewis, Jennifer D., et al. "The Arabidopsis ZED1 pseudokinase is required for ZAR1-mediated immunity induced by the Pseudomonas syringae type III effector HopZ1a." Proceedings of the National Academy of Sciences 110.46 (2013): 18722-18727.
Solyc07g053230.3	transcription factor MYB15-like	MYB15	AT3G23250	Transcription factor involved in cold-regulation of CBF genes and in freezing tolerance; Involved in drought and salt tolerance; Enhances ABA biosynthesis and signaling	Promoted freezing, drought, and salt tolerance; Enchanced ABA biosynthesis and signlaing	Agarwal, Manu, et al. "A R2R3 type MYB transcription factor is involved in the cold regulation of CBF genes and in acquired freezing tolerance." Journal of Biological Chemistry 281.49 (2006): 37636-37645.
Solyc01g087850.2	subtilisin-like protease precursor	N/A	N/A	N/A	N/A	N/A
Solyc01g006320.3	protein NDR1-like	NRD1	AT3G20600	Required for disease resistance conferred by R genes recognizing bacterial and oomycete pathogens; Required for the establishment of hypersensitive response and SAR	Promoted diseased resistance against bacterial and fungal pathogens; Promoted hypersensitive response; Promoted SAR	Century, Karen S., Eric B. Holub, and Brian J. Staskawicz. "NDR1, a locus of Arabidopsis thaliana that is required for disease resistance to both a bacterial and a fungal pathogen." Proceedings of the National Academy of Sciences 92.14 (1995): 6597-6601.
Solyc06g051010.1	peptide-N4-(N-acetyl-beta-glucosaminyl)asparagine amidase A-like	N/A	N/A	Peptide-N4-(N-acetyl-beta-glucosaminyl)asparagine amidase	N/A	Altmann, Friedrich, et al. "Characterisation of peptide-N4-(N-acetyl-β-glucosaminyl) asparagine amidase A and its N-glycans." European journal of biochemistry 252.1 (1998): 118-123.
Solyc09g061890.3	probable pectate lyase 8	AT3G07010	AT3G07010	Involved in pectin catabolic process	Increased pectin catabolism	Weber, Hans, et al. "Selective and powerful stress gene expression in Arabidopsis in response to malondialdehyde." The Plant Journal 37.6 (2004): 877-888.

TomatoID	NCBI Protein Name	Gene ID	*A. Thaliana* Homolog	Uniprot Description	Putative Consequences for Infection	Citation
Solyc03g083010.3	uncharacterized protein LOC101261000 isoform X3	N/A	N/A	N/A	N/A	N/A
Solyc11g072600.2	AP2 transcription factor SlAP2d isoform X1	N/A	N/A	DNA-binding transcription factor; Involved in tomato fruit ripening	Increased tomato ripening	Karlova, Rumyana, et al. "Transcriptome and metabolite profiling show that APETALA2a is a major regulator of tomato fruit ripening." The Plant Cell 23.3 (2011): 923-941.
Solyc03g083720.1	21 kDa protein-like	N/A	N/A	N/A	N/A	N/A
Solyc06g069380.3	O-fucosyltransferase 34	N/A	N/A	N/A	N/A	N/A
Solyc04g025650.3	monooxygenase 1	MO1	AT4G15760	FAD binding; Monooxygenase activity	N/A	Cheng, Chia-Yi, et al. "Araport11: a complete reannotation of the Arabidopsis thaliana reference genome." The Plant Journal 89.4 (2017): 789-804.
Solyc02g088090.1	probable calcium-binding protein CML30	CML30	N/A	Calcium sensor	Promoted calcium sensing	Boonburapong, Bongkoj, and Teerapong Buaboocha. "Genome-wide identification and analyses of the rice calmodulin and related potential calcium sensor proteins." BMC plant biology 7.1 (2007): 4.
Solyc04g074440.1	protein EXORDIUM-like	EXO	AT4G08950	Required for cell expansion in leaves; Mediate brassinosteroid-induced leaf and root growth/development	Promoted cell expansion in the leaves; Promoted leaf and root growth/development	Coll-Garcia, Danahe, et al. "EXORDIUM regulates brassinosteroid-responsive genes." FEBS letters 563.1-3 (2004): 82-86.
Solyc04g078750.3	uncharacterized protein LOC101250111 isoform X1	N/A	N/A	N/A	N/A	N/A
Solyc12g094520.2	4-coumarate--CoA ligase-like 5	4CLL5	AT1G20510	Contributes to JA biosynthesis by initiating the beta-oxidative chain shortening of its precursors	Increased production of phenylproanoid-derived compounds; Increased JA biosynthesis	Koo, Abraham JK, et al. "Identification of a peroxisomal acyl-activating enzyme involved in the biosynthesis of jasmonic acid in Arabidopsis." Journal of Biological Chemistry 281.44 (2006): 33511-33520.
Solyc02g071030.2	chlorophyll a-b binding protein 1B, chloroplastic	LHCB1.3	AT1G29930	Light-harvesting complex functions as a light receptor; Captures and delivers excitation energy to photosystems	Promoted light capture and photosynthesis	Yadav, Vandana, et al. "Light regulated modulation of Z-box containing promoters by photoreceptors and downstream regulatory components, COP1 and HY5, in Arabidopsis." The Plant Journal 31.6 (2002): 741-753.
Solyc02g082930.3	acidic 27 kDa endochitinase precursor	CHI17	N/A	Involved in defense against chitin-containing fungal pathogens	Increased defense against fungal pathogens	Danhash, Nadia, et al. "Molecular characterization of four chitinase cDNAs obtained fromCladosporium fulvum-infected tomato." Plant molecular biology 22.6 (1993): 1017-1029.
Solyc03g082660.3	molybdate-anion transporter	MFSD5	N/A	Mediates high-affinity intracellular uptake of the rare oligo-element molybdenum	Increased intracellular uptake of molybdenum	Tejada-Jiménez, Manuel, Aurora Galván, and Emilio Fernández. "Algae and humans share a molybdate transporter." Proceedings of the National Academy of Sciences 108.16 (2011): 6420-6425.

TomatoID	NCBI Protein Name	Gene ID	*A. Thaliana* Homolog	Uniprot Description	Putative Consequences for Infection	Citation
Solyc04g081530.1	DNAJ-like protein	T5J17.130	AT4G39960	ATP binding, heat shock protein binding, metal ion binding, unfolding protein binding	N/A	Mayer, Klauss, et al. "Sequence and analysis of chromosome 4 of the plant Arabidopsis thaliana." Nature 402.6763 (1999): 769-777.
Solyc07g052510.4	peroxidase 3 precursor	PER3	AT1G05260	Involved in removal of H2O2, oxidation of toxic reductants, biosynthesis and degradation of lignin, suberization, auxin catabolism, response to environmental stressors	Increased removal of H2O2, oxidation of toxic reductants, biosynthesis and degradation of lignin, suberization, auxin catabolism, and response to environmental stressors	Paynel, Florence, et al. "Temporal regulation of cell-wall pectin methylesterase and peroxidase isoforms in cadmium-treated flax hypocotyl." Annals of botany 104.7 (2009): 1363-1372.
Solyc07g064820.1	mitogen-activated protein kinase kinase kinase 18-like	MAPKKK18	AT1G05100	Act as ABA signal transducer under abiotic stress; Promotes stomatal growth/development; Inhibits germination and root growth; Promotes leaf senescence	Increased response to stress; Increased reproductive development; Decreased growth/development; Increased leaf senescence	Mitula, Filip, et al. "Arabidopsis ABA-activated kinase MAPKKK18 is regulated by protein phosphatase 2C ABI1 and the ubiquitin–proteasome pathway." Plant and Cell Physiology 56.12 (2015): 2351-2367.
Solyc12g037930.2	N/A	N/A	N/A	N/A	N/A	N/A
Solyc03g116070.1	mini zinc finger protein 3	MIF3	AT1G18835	Involved in integrating signals from multiple hormones by regulating the expression of specific genes; Promotes the formation of ectopic shoot meristems	Increased hormonal signaling; Promoted formation of ectopic shoot meristems	Hu, Wei, Baomin Feng, and Hong Ma. "Ectopic expression of the Arabidopsis MINI ZINC FINGER1 and MIF3 genes induces shoot meristems on leaf margins." Plant molecular biology 76.1-2 (2011): 57-68.
Solyc04g071400.3	WAT1-related protein At1g43650-like isoform X2	AT1G43650	AT1G43650	Transmembrane transporter activity	Increased transmembrane transporter	Busov, Victor B., et al. "An auxin-inducible gene from loblolly pine (Pinus taeda L.) is differentially expressed in mature and juvenile-phase shoots and encodes a putative transmembrane protein." Planta 218.6 (2004): 916-927.
Solyc06g051620.3	putative phospholipase C	plcD	N/A	Hydrolase activity, acting on ester bonds	N/A	Zheng, Huajun, et al. "Genetic basis of virulence attenuation revealed by comparative genomic analysis of Mycobacterium tuberculosis strain H37Ra versus H37Rv." PloS one 3.6 (2008): e2375.
Solyc06g083650.3	GDSL esterase/lipase At5g33370	AT5G33370	AT5G33370	Involved in cuticle development; Involved in lipid catabolism	Increased cuticle development; Increased lipid catabolism	Li-Beisson, Yonghua, et al. "Acyl-lipid metabolism." The Arabidopsis book/American Society of Plant Biologists 11 (2013).
Solyc09g065620.3	chlorophyllase-2, chloroplastic isoform X4	CLH2	AT5G43860	Catalyzes the hydrolysis of ester bond in chlorophyll to yield chlorophyllide and phytol	Increased chlorophyllide and phytol photosynthesis; Promoted photosynthesis	Tsuchiya, Tohru, et al. "Cloning of chlorophyllase, the key enzyme in chlorophyll degradation: finding of a lipase motif and the induction by methyl jasmonate." Proceedings of the National Academy of Sciences 96.26 (1999): 15362-15367.
Solyc12g014420.2	glucan endo-1,3-beta-glucosidase 13-like	AT5G56590	AT5G56590	Involved in carbohydrate metabolic process, cell wall organization, and plant defense response	Increased carbohydrate metabolism, cell wall organization, and plant defense response	Wu, Qiong, et al. "Long-term balancing selection contributes to adaptation in Arabidopsis and its relatives." Genome biology 18.1 (2017): 1-15.

TomatoID	NCBI Protein Name	Gene ID	*A. Thaliana* Homolog	Uniprot Description	Putative Consequences for Infection	Citation
Solyc03g118450.1	uncharacterized protein LOC104646728	N/A	N/A	N/A	N/A	N/A
Solyc05g007490.3	probable methyltransferase PMT2	AT1G26850	AT1G26850	Involved in methylation	N/A	Ascencio-Ibáñez, José Trinidad, et al. "Global analysis of Arabidopsis gene expression uncovers a complex array of changes impacting pathogen response and cell cycle during geminivirus infection." Plant physiology 148.1 (2008): 436-454.
Solyc09g083445.1	N/A	N/A	N/A	N/A	N/A	N/A
Solyc11g010390.1	classical arabinogalactan protein 10-like	AGP10	AT4G09030	Proteoglycan implicated in diverse developmental roles such as differentiation, cell-cell recognition, embryogenesis, and programmed cell death	Promoted plant growth/development and cell differentiation; Promoted programmed cell death	Thieme, Christoph J., et al. "Endogenous Arabidopsis messenger RNAs transported to distant tissues." Nature Plants 1.4 (2015): 1-9.
Solyc01g099370.3	protein SRC2 homolog	SRC2	AT1G09070	Acts as an activator of RBOHF, which mediates reactive oxygene species production; Plays a role in cold responses	Increased response to reactive oxygen species; Promoted response to cold	Kawarazaki, Tomoko, et al. "A low temperature-inducible protein AtSRC2 enhances the ROS-producing activity of NADPH oxidase AtRbohF." Biochimica et Biophysica Acta (BBA)-Molecular Cell Research 1833.12 (2013): 2775-2780.
Solyc05g015840.3	teosinte glume architecture 1	TGA1	N/A	Acts as a transcriptional repressor of growth of lateral branches in length and numbers	Repressed lateral branching and growth	Wang, Huai, et al. "Evidence that the origin of naked kernels during maize domestication was caused by a single amino acid substitution in tga1." Genetics 200.3 (2015): 965-974.
Solyc07g054220.1	ethylene-responsive transcription factor ERF054	ERF054	AT4G28140	Binds to the GCC-box pathogenesis-related promoter element; Regulates gene expression under stress	Increased response to stress and pathogenesis	Riechmann, José Luis, et al. "Arabidopsis transcription factors: genome-wide comparative analysis among eukaryotes." Science 290.5499 (2000): 2105-2110.
Solyc10g055780.1	endochitinase 4	N/A	N/A	Involved in defense against chitin-containing fungal and bacterial pathogens	Increased defense against chitin-containing pathogens	Herget, Thomas, Jeff Schell, and Peter H. Schreier. "Elicitor-specific induction of one member of the chitinase gene family in Arachis hypogaea." Molecular and General Genetics MGG 224.3 (1990): 469-476.
Solyc11g039860.2	N/A	N/A	N/A	N/A	N/A	N/A
Solyc01g079600.3	phospholipase A1 PLIP2, chloroplastic isoform X1	PLIP2	AT1G02660	Catalyzes the initial step of oxylipins and jasmonate biosynthesis; Links ABA-mediated abiotic stress responses and oxylipin and jasmonate signaling pathways	Increased jasmonate biosynthesis; Increased jasmonate signaling crosstalk	Wang, Kun, et al. "Two abscisic acid-responsive plastid lipase genes involved in jasmonic acid biosynthesis in Arabidopsis thaliana." The Plant Cell 30.5 (2018): 1006-1022.
Solyc04g051360.3	ethylene-responsive transcription factor ABR1-like	ABR1	AT5G64750	Negative regulator of the ABA signaling pathway involved in seed germination and in responses to stress	Increased ABA signaling; Increased response to stress and pathogenesis	Pandey, Girdhar K., et al. "ABR1, an APETALA2-domain transcription factor that functions as a repressor of ABA response in Arabidopsis." Plant Physiology 139.3 (2005): 1185-1193.

TomatoID	NCBI Protein Name	Gene ID	*A. Thaliana* Homolog	Uniprot Description	Putative Consequences for Infection	Citation
Solyc08g082820.3	mediator of RNA polymerase II transcription subunit 37a	LOC107780639	N/A	Plays a role in facilitating the assembly of multimeric protein complexes inside the ER	Increased assembly of multimeric protein complexes inside the ER	N/A
Solyc03g020040.3	proteinase inhibitor type-2-like	N/A	N/A	N/A	N/A	Balandin, Teresa, et al. "Structure and induction pattern of a novel proteinase inhibitor class II gene of tobacco." Plant molecular biology 27.6 (1995): 1197-1204.
Solyc04g076570.3	protein CLT2, chloroplastic isoform X3	CLT2	AT4G24460	Involved in thiol transport from the plastid to the cytosol; Transports glutathione and its precursor	Increased thiol transport; Increased transport of glutathione and its presursor	Maughan, Spencer C., et al. "Plant homologs of the Plasmodium falciparum chloroquine-resistance transporter, PfCRT, are required for glutathione homeostasis and stress responses." Proceedings of the National Academy of Sciences 107.5 (2010): 2331-2336.
Solyc06g053640.1	RING-H2 finger protein ATL16-like	ATL16	AT5G43420	Involved in protein ubiquitination	Promoted protein ubiquitination	Thieme, Christoph J., et al. "Endogenous Arabidopsis messenger RNAs transported to distant tissues." Nature Plants 1.4 (2015): 1-9.
Solyc06g069070.1	non-specific lipid-transfer protein 2	LTP2	AT2G38530	Plays a role in wax/cutin deposition in the cell walls of expanding epidermal cells and certain secretory tissues	Promoted cell wall expansion	Peragine, Angela, et al. "SGS3 and SGS2/SDE1/RDR6 are required for juvenile development and the production of trans-acting siRNAs in Arabidopsis." Genes & development 18.19 (2004): 2368-2379.
Solyc09g031920.1	uncharacterized protein LOC101268505	N/A	N/A	N/A	N/A	N/A
Solyc03g116590.3	embryo-specific protein ATS3B	ATS3B	AT5G62200	Plays a role during embryo development	Promoted embryo development	Sato, Shusei, et al. "Structural analysis of Arabidopsis thaliana chromosome 5. X. Sequence features of the regions of 3,076,755 bp covered by sixty P1 and TAC clones." DNA research 7.1 (2000): 31-63.
Solyc05g005160.3	ATP-citrate synthase alpha chain protein 2	ACLA-2	AT1G60810	ATP citrate-lyase is the primary enzyme responsible for the synthesis of cytosolic acetyl-CoA; Required for normal growth and development in seeds	Promoted seed development; Increased production of primary metabolites	Fatland, Beth L., Basil J. Nikolau, and Eve Syrkin Wurtele. "Reverse genetic characterization of cytosolic acetyl-CoA generation by ATP-citrate lyase in Arabidopsis." The Plant Cell 17.1 (2005): 182-203.
Solyc10g008000.1	protein LIGHT-DEPENDENT SHORT HYPOCOTYLS 10-like	LSH10	AT2G42610	Transcription regulator that acts as a developmental regulator by promoting cell growth in response to light	Promoted cell growth in response to light	Iyer, Lakshminarayan M., and L. Aravind. "ALOG domains: provenance of plant homeotic and developmental regulators from the DNA-binding domain of a novel class of DIRS1-type retroposons." Biology direct 7.1 (2012): 1-8.
Solyc01g080600.3	histone H3.2	HTR2	AT1G09200	Core component of the nucleosome; Plays central roles in transcription regulation, DNA repair/replication, and chromosomal stability	Increased transcription regulation and DNA repair	Seki, Motoaki, et al. "Functional annotation of a full-length Arabidopsis cDNA collection." Science 296.5565 (2002): 141-145.
Solyc02g069910.2	N/A	N/A	N/A	N/A	N/A	N/A

TomatoID	NCBI Protein Name	Gene ID	*A. Thaliana* Homolog	Uniprot Description	Putative Consequences for Infection	Citation
Solyc06g066600.3	probable plastidic glucose transporter 3 isoform X2	N/A	N/A	N/A	N/A	N/A
Solyc07g007870.3	12-oxophytodienoate reductase 3	OPR3	AT2G06050	Involved in the biosynthesis of JA and other oxylipin signaling moleclules; Required for the spatial and temporal regulation of JA levels during dehiscence of anthers	Increased biosynthesis of JA and other oxylipin signaling molecules	Sanders, Paul M., et al. "The Arabidopsis DELAYED DEHISCENCE1 gene encodes an enzyme in the jasmonic acid synthesis pathway." The Plant Cell 12.7 (2000): 1041-1061.
Solyc08g083370.3	soluble inorganic pyrophosphatase PPA1-like isoform X1	PPA1	AT1G01050	Controls the equilibrium of gluconeogenic reactions in the heterotrophic growth phase of early seedling establishment	Promoted equilibriation gluconeogenic reactions	Navarro-De la Sancha, Ernesto, et al. "Characterization of two soluble inorganic pyrophosphatases from Arabidopsis thaliana." Plant science 172.4 (2007): 796-807.
Solyc10g006660.3	calcium-binding protein KRP1	KRP1	AT4G27280	Potential calcium sensor	Promoted calcium sensing; Promoted regulation of trichome morphogenesis	Reddy, Vaka S., et al. "KIC, a novel Ca2+ binding protein with one EF-hand motif, interacts with a microtubule motor protein and regulates trichome morphogenesis." The Plant Cell 16.1 (2004): 185-200.
Solyc04g054950.3	tropinone reductase homolog	AT1G07440	AT1G07440	Oxidoreductase activity	N/A	Cheng, Chia-Yi, et al. "Araport11: a complete reannotation of the Arabidopsis thaliana reference genome." The Plant Journal 89.4 (2017): 789-804.
Solyc06g049020.1	uncharacterized protein LOC101254906	N/A	N/A	N/A	N/A	N/A
Solyc07g016215.1	N/A	N/A	N/A	N/A	N/A	N/A
Solyc08g075870.3	probable methyltransferase PMT21	ERD3	AT4G19120	Involved in methylation	Increased methylation	Ascencio-Ibáñez, José Trinidad, et al. "Global analysis of Arabidopsis gene expression uncovers a complex array of changes impacting pathogen response and cell cycle during geminivirus infection." Plant physiology 148.1 (2008): 436-454.
Solyc12g040570.1	6-phosphogluconate dehydrogenase, decarboxylating 3	PGD3	AT5G41670	Essential component in cell redox homeostasis	Increased cell redox homeostasis	Fernández-Fernández, Álvaro D., and Francisco J. Corpas. "In silico analysis of Arabidopsis thaliana peroxisomal 6-phosphogluconate dehydrogenase." Scientifica 2016 (2016).
Solyc01g080910.2	uncharacterized protein LOC101252815	N/A	N/A	N/A	N/A	N/A
Solyc02g087770.3	aldose 1-epimerase superfamily protein precursor	N/A	N/A	Mutarotase which converts alpha-aldose to the beta-anomer	N/A	Sriranganadane, Dev, et al. "Identification of novel secreted proteases during extracellular proteolysis by dermatophytes at acidic pH." Proteomics 11.22 (2011): 4422-4433.

TomatoID	NCBI Protein Name	Gene ID	*A. Thaliana* Homolog	Uniprot Description	Putative Consequences for Infection	Citation
Solyc05g050560.1	transcription factor bHLH13-like	BHLH13	AT1G01260	Involved in negative regulation of gene expression	N/A	Borsics, Tamás, et al. "The cyclic nucleotide-gated calmodulin-binding channel AtCNGC10 localizes to the plasma membrane and influences numerous growth responses and starch accumulation in Arabidopsis thaliana." Planta 225.3 (2007): 563-573.
Solyc07g017600.3	pectinesterase/pectinesterase inhibitor PPE8B	N/A	N/A	Plays roles in the deposition of pectin in developing tissues and in the wall loosening and cell separation that occurs in cell expansion, fruit ripening and abscission.	Increased pectin deposition and plant cell wall loosening	Glover, H., et al. "Multiple pectin esterase genes are expressed in ripening peach fruit: nucleotide sequence of a cDNA encoding peach pectin esterase." Plant Physiology 112 (1996): 864-864.
Solyc09g008280.2	S-adenosylmethionine synthase 3	METK3	AT2G36880	Catalyzes the formation of S-adenosylmethionine from methionine; Involved in SA-mediated defense; Involved in the biosynthesis of lignin	Increased S-adenosylmethionine production; Promoted early defense response; Increased lignin biosynthesis	Goto, Derek B., et al. "A single-nucleotide mutation in a gene encoding S-adenosylmethionine synthetase is associated with methionine over-accumulation phenotype in Arabidopsis thaliana." Genes & genetic systems 77.2 (2002): 89-95.
Solyc09g089930.2	ethylene responsive element binding protein	EREBP1	N/A	Involved in defense response; Involved in ethylene-activated signaling pathway	Increased defense response; Increased ethylene-responsive signaling	Horvath, Diana M., Dorothy J. Huang, and Nam-Hai Chua. "Four classes of salicylate-induced tobacco genes." Molecular plant-microbe interactions 11.9 (1998): 895-905.
Solyc01g102390.3	germin-like protein 5-1	OS05G0197200	N/A	Plays a role in plant defense	Increased plant defense	Kawahara, Yoshihiro, et al. "Improvement of the Oryza sativa Nipponbare reference genome using next generation sequence and optical map data." Rice 6.1 (2013): 4.
Solyc02g093230.3	caffeoyl-CoA O-methyltransferase 1	CCOAOMT1	At1g67980	Involved in the reinforcement of the plant cell wall; Involved in response to wounding or pathogen challenge by promoting the formation of cell wall-bound ferulic acid polymers	Increased production of feruloylated polysaccharides; Promoted plant cell wall development and integrity	Meyermans, Hugo, et al. "Modifications in lignin and accumulation of phenolic glucosides in poplar xylem upon down-regulation of caffeoyl-coenzyme A O-methyltransferase, an enzyme involved in lignin biosynthesis." Journal of Biological Chemistry 275.47 (2000): 36899-36909.
Solyc07g008410.3	protein DETOXIFICATION 29-like	DTX29	AT3G26590	Xenobiotic transmembrane transporter activity	Increased detoxification	Hanada, Kousuke, et al. "Functional compensation of primary and secondary metabolites by duplicate genes in Arabidopsis thaliana." Molecular biology and evolution 28.1 (2011): 377-382.
Solyc01g107390.3	indole-3-acetic acid-amido synthetase GH3.2	GH3.2	AT4G37390	Catalyzes the synthesis of indole-3-acetic acid-amino acid conjugates, providing a mechanism for the plant to cope with the presence of excess auxin	Promoted auxin homeostasis	Hilson, Pierre, et al. "Versatile gene-specific sequence tags for Arabidopsis functional genomics: transcript profiling and reverse genetics applications." Genome research 14.10b (2004): 2176-2189.
Solyc02g082430.3	MLO-like protein 8 isoform X2	MLO8	AT2G17480	Involved in modulation of pathogen defense and leaf cell death	Promoted programmed cell death; Increased defense against pathogens	Menges, Margit, et al. "Cell cycle-regulated gene expression inarabidopsis." Journal of Biological Chemistry 277.44 (2002): 41987-42002.
Solyc03g112960.1	pectinesterase 1	PME1	AT1G53840	Acts in the modification of cell walls; Acts as negative regulator of genes involved in salt stress response	Increased modification of plant cell walls; Impaired salt stress response	Creighton, Maria T., et al. "Methylation of protein phosphatase 2A—Influence of regulators and environmental stress factors." Plant, cell & environment 40.10 (2017): 2347-2358.

TomatoID	NCBI Protein Name	Gene ID	*A. Thaliana* Homolog	Uniprot Description	Putative Consequences for Infection	Citation
Solyc10g050160.2	caffeoyl-CoA O-methyltransferase 5	CCOAOMT5	N/A	Plays a role in the synthesis of feruloylated polysaccharides; Involved in the reinforcement of the plant cell wall; Involved in response to wounding or pathogen challenge	Increased production of feruloylated polysaccharides; Promoted plant cell wall development and integrity	Meyermans, Hugo, et al. "Modifications in lignin and accumulation of phenolic glucosides in poplar xylem upon down-regulation of caffeoyl-coenzyme A O-methyltransferase, an enzyme involved in lignin biosynthesis." Journal of Biological Chemistry 275.47 (2000): 36899-36909.
Solyc04g072000.3	endochitinase EP3	EP3	AT3G54420	Involved in hypersensitive reaction against specific pathogens	Increased hypersensitive reaction against some pathogens	Liliane, B. de A., et al. "Arabidopsis thaliana class IV chitinase is early induced during the interaction with Xanthomonas campestris." Febs Letters 419.1 (1997): 69-75.
Solyc06g083050.3	serine carboxypeptidase-like 20	SCPL20	AT4G12910	Carboxypeptidase	N/A	N/A
Solyc07g007590.1	arogenate dehydrogenase 2, chloroplastic-like	TYRAAT2	AT1G15710	Involved in the biosynthesis of tyrosine	Increased production of tyrosine biosynthesis	Rippert, Pascal, and Michel Matringe. "Purification and kinetic analysis of the two recombinant arogenate dehydrogenase isoforms of Arabidopsis thaliana." European Journal of Biochemistry 269.19 (2002): 4753-4761.
Solyc11g010500.1	mitochondrial uncoupling protein 5	PUMP5	AT2G22500	Mitochondrial transporters that create proton leaks across the inner mitochondrial membrane; Involved in protecting plant cells against oxidative stress damage	Increased defense against oxidative stress	Borecký, Jiří, et al. "The plant energy-dissipating mitochondrial systems: depicting the genomic structure and the expression profiles of the gene families of uncoupling protein and alternative oxidase in monocots and dicots." Journal of Experimental Botany 57.4 (2006): 849-864.
Solyc12g036470.2	transcription factor BEE 1-like	BEE1	AT1G18400	Positive regulator of brassinosteroid signaling	Promoted brassinosteroid signaling	Friedrichsen, Danielle M., et al. "Three redundant brassinosteroid early response genes encode putative bHLH transcription factors required for normal growth." Genetics 162.3 (2002): 1445-1456.
Solyc02g082260.3	3-hydroxy-3-methylglutaryl-coenzyme A reductase 1	HMG1	AT1G76490	Catalyzes the synthesis of mevalonate, the specific precursor of all isoprenoid compounds present in plants	Increased synthesis of isoprenoids	Dale, Susan, et al. "Bacterial expression of the catalytic domain of 3–hydroxy-3–methylglutaryl-coa reductase (isoform HMGR1) from Arabidopsis thaliana, and Its inactivation by phosphorylation at Ser577 by Brassica oleracea 3-hydroxy-3-methylglutaryl-CoA reductase kinase." European Journal of Biochemistry 233.2 (1995): 506-513.
Solyc01g102640.2	nuclear pore complex protein NUP98A-like	NUP98A	AT1G10390	Structural constituent of nuclear pore; Necessary for mRNA transport from the nucleus and protein import into the nucleus; Required for photoperiodism; Required for constitutive defense	Impaired mRNA transport; Impaired photoperiodism; Impaired constitutive defense	"A putative nucleoporin 96 is required for both basal defense and constitutive resistance responses mediated by suppressor of npr1-1,constitutive 1."
Solyc02g077770.3	N/A	N/A	N/A	N/A	N/A	N/A

TomatoID	NCBI Protein Name	Gene ID	*A. Thaliana* Homolog	Uniprot Description	Putative Consequences for Infection	Citation
Solyc10g018580.1	hypothetical chloroplast RF1	ycf1a	N/A	Kinase activity	N/A	Lemieux, Claude, et al. "Chloroplast phylogenomic analysis of chlorophyte green algae identifies a novel lineage sister to the Sphaeropleales (Chlorophyceae)." BMC Evolutionary Biology 15.1 (2015): 264.
Solyc01g094210.2	(+)-neomenthol dehydrogenase	SDR1	AT3G61220	Involved in basal resistance against pathogens	Impaired basal resistance against pathogens	Choi, Hyong Woo, et al. "A role for a menthone reductase in resistance against microbial pathogens in plants." Plant physiology 148.1 (2008): 383-401.
Solyc03g006880.3	gibberellin 20-oxidase	GA20OX1	AT4G25420	Promotes transition from vegetative to floral state, plant fertility, and silique elongation	Impaired plant reproduction	Rieu, Ivo, et al. "The gibberellin biosynthetic genes AtGA20ox1 and AtGA20ox2 act, partially redundantly, to promote growth and development throughout the Arabidopsis life cycle." The Plant Journal 53.3 (2008): 488-504.
Solyc04g076010.3	uncharacterized protein LOC101247909	N/A	N/A	N/A	N/A	N/A
Solyc08g066350.2	histidine-containing phosphotransfer protein 4-like	AHP4	AT3G16360	Plays an important role in propagating cytokinin signal transduction through the multistep His-to-Asp phosphorelay	Impaired stem and bud growth; Impaired response to auxin	Hwang, Ildoo, Huei-Chi Chen, and Jen Sheen. "Two-component signal transduction pathways in Arabidopsis." Plant Physiology 129.2 (2002): 500-515.
Solyc11g012680.2	pollen-specific leucine-rich repeat extensin-like protein 1	PEX1	AT3G19020	Modulates cell morphogenesis by regulating cell wall formation and assembly, and/or growth polarization	Impaired cell morphogenesis by regulating cell wall formation and assembly, and/or growth polarization	Mecchia, Martin A., et al. "RALF4/19 peptides interact with LRX proteins to control pollen tube growth in Arabidopsis." Science 358.6370 (2017): 1600-1603.
Solyc03g097930.3	potassium channel SKOR-like	SKOR	AT3G02850	Involved in potassium release into the xylem sap toward the shoots; Interacts with the cytoskeleton or with regulatory proteins	Impaired potassium transport	Gaymard, Frédéric, et al. "Identification and disruption of a plant shaker-like outward channel involved in K+ release into the xylem sap." Cell 94.5 (1998): 647-655.
Solyc10g051020.2	cytochrome P450 CYP72A219	LOC104242999	N/A	Heme binding; Iron ion binding; Monooxygenase activity	N/A	Sierro, Nicolas, et al. "Reference genomes and transcriptomes of Nicotiana sylvestris and Nicotiana tomentosiformis." Genome biology 14.6 (2013): R60.
Solyc10g051030.2	uncharacterized protein LOC101245596 isoform X3	N/A	N/A	N/A	N/A	N/A
Solyc10g084070.1	N/A	N/A	N/A	N/A	N/A	N/A
Solyc06g076490.3	uncharacterized protein LOC101266832	N/A	N/A	N/A	N/A	N/A
Solyc11g010270.2	homeobox-leucine zipper protein ATHB-6	ATHB-6	AT2G22430	Acts as a growth regulator in response to water deficit; Involved in the negative regulation of the ABA signaling pathway	Impaired response to drought; Impaired regulation of ABA signaling	Himmelbach, Axel, et al. "Homeodomain protein ATHB6 is a target of the protein phosphatase ABI1 and regulates hormone responses in Arabidopsis." The EMBO journal 21.12 (2002): 3029-3038.

TomatoID	NCBI Protein Name	Gene ID	*A. Thaliana* Homolog	Uniprot Description	Putative Consequences for Infection	Citation
Solyc12g096490.1	protein GLUTAMINE DUMPER 5-like	GDU5	AT5G24920	Involved in the regulation of the amino acid metabolism	Impaired amino acid tranport; Impaired regulation of amino acid metabolism	Pratelli, Réjane, et al. "Stimulation of nonselective amino acid export by glutamine dumper proteins." Plant Physiology 152.2 (2010): 762-773.
Solyc05g054850.1	probable membrane-associated kinase regulator 3	MAKR3	AT2G37380	Kinase inhibitor activity	N/A	Cheng, Chia-Yi, et al. "Araport11: a complete reannotation of the Arabidopsis thaliana reference genome." The Plant Journal 89.4 (2017): 789-804.
Solyc06g008760.1	glutaredoxin-C13	GRXC13	AT2G47880	Involved in regulation of plant defense responses	Decreased regulation of plant defense	Brodersen, Peter, et al. "Arabidopsis MAP kinase 4 regulates salicylic acid-and jasmonic acid/ethylene-dependent responses via EDS1 and PAD4." The Plant Journal 47.4 (2006): 532-546.
Solyc10g051120.2	mitochondrial pyruvate carrier 1-like	MPC1	AT5G20090	Mediates the uptake of pyruvate into mitochondria	Impaired drought response	Li, Chun-Long, et al. "NRGA1, a putative mitochondrial pyruvate carrier, mediates ABA regulation of guard cell ion channels and drought stress responses in Arabidopsis." Molecular plant 7.10 (2014): 1508-1521.
Solyc12g056950.2	protein MARD1-like	MARD1	AT3G63210	Facilitates the interaction of SnRK1 complex with effector proteins; Involved in seed dormancy control	Impaired seed dormancy	He, Yuehui, and Susheng Gan. "A novel zinc-finger protein with a proline-rich domain mediates ABA-regulated seed dormancy in Arabidopsis." Plant molecular biology 54.1 (2004): 1-9.
Solyc03g025350.3	silicon efflux transporter LSI2-like	LSI2	N/A	Silicon efflux transporter involved in silicon transport from the root cells to the apoplast	Impaired cell wall response to abiotic and biotic stressors	Ma, Jian Feng, et al. "An efflux transporter of silicon in rice." Nature 448.7150 (2007): 209-212.
Solyc00g203660.2	ribulose-1,5-bisphosphate carboxylase/oxygenase large subunit	rbcL	ATCG00490	RuBisCO catalyzes two reactions: Participates in the the carboxylation of D-ribulose 1,5-bisphosphate of RuBisCO	Decreased RuBisCo catalysis	Chun, Louis, Alana Kawakami, and David A. Christopher. "Phytochrome A mediates blue light and UV-A-dependent chloroplast gene transcription in green leaves." Plant Physiology 125.4 (2001): 1957-1966.
Solyc06g069060.1	N/A	N/A	N/A	N/A	N/A	N/A
Solyc08g068490.3	indole-3-acetic acid-amido synthetase GH3.10-like	GH3.10	AT4G03400	Catalyzes the synthesis of indole-3-acetic acid-amino acid conjugates, providing a mechanism for the plant to cope with the presence of excess auxin	Impaired auxin homeostasis	Takase, Tomoyuki, et al. "DFL2, a new member of the Arabidopsis GH3 gene family, is involved in red light-specific hypocotyl elongation." Plant and Cell Physiology 44.10 (2003): 1071-1080.
Solyc04g081460.2	uncharacterized protein LOC101250258	N/A	N/A	N/A	N/A	N/A
Solyc07g014620.1	auxin-responsive protein SAUR50	SAUR50	AT4G34760	Effector of hormonal and environmental signals in plant growth	Impaired response to hormonal and environmental signaling; Impaired growth/development	Ren, Hong, and William M. Gray. "SAUR proteins as effectors of hormonal and environmental signals in plant growth." Molecular plant 8.8 (2015): 1153-1164.

TomatoID	NCBI Protein Name	Gene ID	*A. Thaliana* Homolog	Uniprot Description	Putative Consequences for Infection	Citation
Solyc07g056280.3	WRKY transcription factor 30	WRKY30	AT5G24110	Transcription factor involved in leaf senescence, response to hydrogen peroxide, response to ozone, and SA signaling pathway	Impaired leaf senescence; Impaired response to hydrogen peroxide; Impaired reponse to ozone; Impaired resonse to SA signaling	El-Esawi, Mohamed A., et al. "Overexpression of AtWRKY30 transcription factor enhances heat and drought stress tolerance in wheat (Triticum aestivum L.)." Genes 10.2 (2019): 163.
Solyc09g082980.3	uncharacterized protein LOC101261455	N/A	N/A	N/A	N/A	N/A
Solyc10g080670.2	uncharacterized protein LOC101267365	N/A	N/A	N/A	N/A	N/A
Solyc02g084930.3	abscisic acid 8'-hydroxylase 3-like	CYP707A3	AT5G45340	Involved in the oxidative degradation of ABA; Involved in the control of postgermination growth	Impaired degradation of ABA; Impaired postgermination growth	Umezawa, Taishi, et al. "CYP707A3, a major ABA 8′-hydroxylase involved in dehydration and rehydration response in Arabidopsis thaliana." The Plant Journal 46.2 (2006): 171-182.
Solyc12g036550.2	Ycf1	Ycf1	N/A	Involved in protein precursor import into chloroplasts; Protein-conducting channel at the inner envelope	Decreased protein precursor import into chloroplasts	Kikuchi, Shingo, et al. "Uncovering the protein translocon at the chloroplast inner envelope membrane." Science 339.6119 (2013): 571-574.
Solyc04g007210.3	zinc finger protein CONSTANS-LIKE 16	COL16	AT1G25440	Involved in regulation of transcription	N/A	Khanna, Rajnish, et al. "The Arabidopsis B-box zinc finger family." The Plant Cell 21.11 (2009): 3416-3420.
Solyc04g082480.3	protein NLP4 isoform X1	LOC101512737	N/A	N/A	N/A	Parween, Sabiha, et al. "An advanced draft genome assembly of a desi type chickpea (Cicer arietinum L.)." Scientific reports 5 (2015): 12806.
Solyc12g006630.2	uncharacterized protein LOC101264095	N/A	N/A	N/A	N/A	N/A
Solyc06g074800.1	zinc finger protein ZAT5-like	ZAT5	AT2G28200	Transcription factor involved in stress responses	Impaired response to stressors	Mittler, Ron, et al. "Gain-and loss-of-function mutations in Zat10 enhance the tolerance of plants to abiotic stress." FEBS letters 580.28-29 (2006): 6537-6542.
Solyc11g069700.2	elongation factor 1-alpha	A1	AT1G07940	Promotes the GTP-dependent binding of aminoacyl-tRNA to the A-site of ribosomes during protein biosynthesis	Impaired binding of aminoacyl-tRNA to the A-site of ribosomes during protein biosynthesis	Lahmy, Sylvie, et al. "DOMINO1, a member of a small plant-specific gene family, encodes a protein essential for nuclear and nucleolar functions." The Plant Journal 39.6 (2004): 809-820.
Solyc00g013170.1	N/A	N/A	N/A	N/A	N/A	N/A
Solyc01g100490.3	nicotianamine synthase	NAS1	AT5G04950	Synthesizes nicotianamine, a sensor for the physiological iron status within the plant; Involved in the transport of iron	Impaired iron transport	Weber, Michael, et al. "Comparative microarray analysis of Arabidopsis thaliana and Arabidopsis halleri roots identifies nicotianamine synthase, a ZIP transporter and other genes as potential metal hyperaccumulation factors." The Plant Journal 37.2 (2004): 269-281.
Solyc05g010427.1	N/A	N/A	N/A	N/A	N/A	N/A

TomatoID	NCBI Protein Name	Gene ID	*A. Thaliana* Homolog	Uniprot Description	Putative Consequences for Infection	Citation
Solyc06g061240.3	uncharacterized protein LOC101268004	N/A	N/A	N/A	N/A	N/A
Solyc10g080910.1	N/A	N/A	N/A	N/A	N/A	N/A
Solyc08g005305.1	N/A	N/A	N/A	N/A	N/A	N/A
Solyc01g098130.2	N/A	N/A	N/A	N/A	N/A	N/A
Solyc06g060830.3	homeobox-leucine zipper protein HAT4	HAT4	AT4G16780	Involved in the negative regulation of cell elongation and specific cell proliferation processes; Mediator of the red/far-red light effects on leaf cell expansion in the shading response	Impaired regulation of cell elongation; Impaired regulation of lateral root formation and vascular system; Impaired shading response	Schena, Mark, Alan M. Lloyd, and Ronald W. Davis. "The HAT4 gene of Arabidopsis encodes a developmental regulator." Genes & development 7.3 (1993): 367-379.
Solyc10g054820.2	aquaporin-5 isoform X2	PIP2-5	AT3G54820	Water channel required to facilitate the transport of water across cell membrane	Decreased water transport	Marmagne, Anne, et al. "Identification of new intrinsic proteins in Arabidopsis plasma membrane proteome." Molecular & Cellular Proteomics 3.7 (2004): 675-691.
Solyc07g014680.3	Na+ transporter HKT1,1	HKT1	AT4G10310	Plays a central role in plant tolerance to salt; Involved in Na+ recirculation from shoots to roots by mediating Na+ loading into the phloem sap in shoots and unloading in roots	Impaired salt tolerance	Uozumi, Nobuyuki, et al. "The Arabidopsis HKT1 gene homolog mediates inward Na+ currents in Xenopus laevis oocytes and Na+ uptake in Saccharomyces cerevisiae." Plant physiology 122.4 (2000): 1249-1260.
Solyc10g080920.2	protein RADIALIS-like 1	RL1	AT4G39250	Transcription factor	N/A	Cheng, Chia-Yi, et al. "Araport11: a complete reannotation of the Arabidopsis thaliana reference genome." The Plant Journal 89.4 (2017): 789-804.
Solyc03g119320.1	uncharacterized protein LOC101259728	N/A	N/A	N/A	N/A	N/A
Solyc07g066330.3	NAC domain-containing protein 21/22 isoform X2	LOC107784013	N/A	Involved in regulation of transcription	Impaired regulation of transcription	Sierro, Nicolas, et al. "The tobacco genome sequence and its comparison with those of tomato and potato." Nature communications 5.1 (2014): 1-9.
Solyc00g126000.1	N/A	N/A	N/A	N/A	N/A	N/A
Solyc03g070430.1	N/A	N/A	N/A	N/A	N/A	N/A
Solyc04g080040.3	J domain-containing protein required for chloroplast accumulation response 1	JAC1	AT1G75100	Required for chloroplast photorelocation movement	Impaired photoperiodism	Suetsugu, Noriyuki, Takatoshi Kagawa, and Masamitsu Wada. "An auxilin-like J-domain protein, JAC1, regulates phototropin-mediated chloroplast movement in Arabidopsis." Plant Physiology 139.1 (2005): 151-162.
Solyc08g074893.1	N/A	N/A	N/A	N/A	N/A	N/A

TomatoID	NCBI Protein Name	Gene ID	*A. Thaliana* Homolog	Uniprot Description	Putative Consequences for Infection	Citation
Solyc09g090360.3	phosphate transporter PHO1	PHO1	AT3G23430	Regulates Pi starvation responses; Involved in the transfer of Pi from roots to shoots; Involved in ABA induction of stomatal closure	Impaired Pi starvation response; Impaired transfer of Pi from roots to shoots; Impaired ABA induction of stomatal closure	Stefanovic, Aleksandra, et al. "Members of the PHO1 gene family show limited functional redundancy in phosphate transfer to the shoot, and are regulated by phosphate deficiency via distinct pathways." The Plant Journal 50.6 (2007): 982-994.
Solyc03g058390.2	eukaryotic translation initiation factor 5B-like	AT1G76720	AT1G76720	GTPase activity; Translation initiation factor	N/A	Cheng, Chia-Yi, et al. "Araport11: a complete reannotation of the Arabidopsis thaliana reference genome." The Plant Journal 89.4 (2017): 789-804.
Solyc03g119910.3	3b-hydroxylase	Le3OH-1	N/A	N/A	N/A	Yang, Y. Y., et al. "Cloning of two cDNAs encoding gibberellin 3beta-hydroxylase (Accession No. AB010991, AB010992) of tomato (Solanum lycopersicum L.) seedlings (PGR98-200)." Plant Physiol 118.1534 (1998): 1534.
Solyc02g011830.1	Ycf1	Ycf1	N/A	Involved in protein precursor import into chloroplasts; Part of an intermediate translocation complex	Decreased protein precursor import into chloroplasts	Kikuchi, Shingo, et al. "Uncovering the protein translocon at the chloroplast inner envelope membrane." Science 339.6119 (2013): 571-574.
Solyc08g081700.1	N/A	N/A	N/A	N/A	N/A	N/A
Solyc00g126010.1	N/A	N/A	N/A	N/A	N/A	N/A
Solyc10g005080.3	protein LHY isoform X2	LHY	AT1G01060	Transcription factor involved in the circadian clock; Represses CCA1	Impaired circadian rhythm; Impaired regulation of CCA1	Mizoguchi, Tsuyoshi, et al. "LHY and CCA1 are partially redundant genes required to maintain circadian rhythms in Arabidopsis." Developmental cell 2.5 (2002): 629-641.
Solyc11g021310.1	Ycf1	Ycf1	N/A	Involved in protein precursor import into chloroplasts; Translocation complex acting as a protein-conducting channel	Decreased protein precursor import into chloroplasts	Kikuchi, Shingo, et al. "Uncovering the protein translocon at the chloroplast inner envelope membrane." Science 339.6119 (2013): 571-574.
Solyc05g023730.1	ribosomal protein L2	N/A	N/A	N/A	N/A	N/A
Solyc08g075740.1	N/A	N/A	N/A	N/A	N/A	N/A
Solyc05g049990.3	heavy metal-associated isoprenylated plant protein 39-like	HIPP39	AT1G01490	Heavy metal-binding protein involved in metal ion transport	Impaired metal ion transport	Hanada, Kousuke, et al. "Functional compensation of primary and secondary metabolites by duplicate genes in Arabidopsis thaliana." Molecular biology and evolution 28.1 (2011): 377-382.
Solyc11g066510.2	RING-H2 finger protein ATL57	ATL57	AT2G27940	Involved in the pathway protein ubiquitination, which is part of protein modification	Impaired protein ubiquitination	Thieme, Christoph J., et al. "Endogenous Arabidopsis messenger RNAs transported to distant tissues." Nature Plants 1.4 (2015): 1-9.
Solyc12g005750.1	B-box domain protein 30	MIP1A	AT4G15248	Developmental regulator; Involved in the CO-mediated long-day flowering-promotion pathway	Decreased sequestration of CO and CO-like proteins; Impaired flowering	Graeff, Moritz, et al. "MicroProtein-mediated recruitment of CONSTANS into a TOPLESS trimeric complex represses flowering in Arabidopsis." PLoS genetics 12.3 (2016): e1005959.

TomatoID	NCBI Protein Name	Gene ID	*A. Thaliana* Homolog	Uniprot Description	Putative Consequences for Infection	Citation
Solyc01g110360.3	fructose-bisphosphate aldolase 1	FBA1	AT2G21330	Plays a key role in glycolysis and gluconeogenesis	Impaired glycolysis and glucogenesis	Searle, Iain R., et al. "Long-distance signaling in nodulation directed by a CLAVATA1-like receptor kinase." Science 299.5603 (2003): 109-112.
Solyc04g058150.3	metallothionein-like protein type 2 A	MT2A	AT3G09390	Functions as metal chelator of copper and Zn; Plays a role in Cu homeostasis	Impaired Cu homeostasis; Impaired metal ion transport	Guo, Woei-Jiun, Metha Meetam, and Peter B. Goldsbrough. "Examining the specific contributions of individual Arabidopsis metallothioneins to copper distribution and metal tolerance." Plant physiology 146.4 (2008): 1697-1706.
Solyc11g007860.1	self-pruning interacting protein	N/A	N/A	N/A	N/A	N/A
Solyc04g006950.3	N/A	N/A	N/A	N/A	N/A	N/A
Solyc08g075880.3	heavy metal-associated isoprenylated plant protein 30-like	HIPP30	AT2G18196	Involved in metal ion transport; Involved in response to drought stress	Impaired metal ion transport; Impaired response to drought stress	"Stress induced and nuclear localized HIPP26 from Arabidopsis thaliana interacts via its heavy metal associated domain with the drought stress related zinc finger transcription factor ATHB29."
Solyc07g005210.3	temperature-induced lipocalin-1	TIL	AT5G58070	Involved in basal and acquired thermotolerance; Lipocalin that confers protection against oxidative stress caused by heat, hypersalinity, freezing, paraquat, and light	Impaired thermotolerance; Impaired tolerance to salt stress, freezing, paraquat, and light	Abo-Ogiala, Atef, et al. "Temperature-induced lipocalin (TIL) is translocated under salt stress and protects chloroplasts from ion toxicity." Journal of plant physiology 171.3-4 (2014): 250-259.
Solyc11g006290.2	3-oxo-5-alpha-steroid 4-dehydrogenase 1	SRD5A1	AT5G16010	Involved in phytohormone signaling	Impaired phytohormone signaling	Thieme, Christoph J., et al. "Endogenous Arabidopsis messenger RNAs transported to distant tissues." Nature Plants 1.4 (2015): 1-9.
Solyc12g036793.1	N/A	N/A	N/A	N/A	N/A	N/A
Solyc12g096130.2	N/A	N/A	N/A	N/A	N/A	N/A
Solyc01g007810.1	transcription repressor OFP15	OFP15	AT2G36050	Transcriptional repressor that regulates multiple aspects of plant growth and development	Promoted plant growth and development; Impaired regulation of plant growth and development	Wang, Shucai, et al. "Arabidopsis ovate family proteins, a novel transcriptional repressor family, control multiple aspects of plant growth and development." PLoS One 6.8 (2011): e23896.
Solyc02g080510.1	uncharacterized protein LOC101253959	N/A	N/A	N/A	N/A	N/A
Solyc05g053850.3	protein SELF PRUNING 5G	SP	N/A	N/A	N/A	Thouet, Johanna, et al. "Revisiting the involvement of SELF-PRUNING in the sympodial growth of tomato." Plant Physiology 148.1 (2008): 61-64.
Solyc11g005290.1	RING-H2 finger protein ATL78-like	ATL78	AT2G27940	Involved in the pathway protein ubiquitination, which is part of protein modification	Impaired protein ubiquitination	Thieme, Christoph J., et al. "Endogenous Arabidopsis messenger RNAs transported to distant tissues." Nature Plants 1.4 (2015): 1-9.
Solyc01g112190.3	protein NLP2	NLP2	AT4G35270	Transcription factor	N/A	Konishi, Mineko, and Shuichi Yanagisawa. "Arabidopsis NIN-like transcription factors have a central role in nitrate signalling." Nature communications 4.1 (2013): 1-9.

TomatoID	NCBI Protein Name	Gene ID	*A. Thaliana* Homolog	Uniprot Description	Putative Consequences for Infection	Citation
Solyc07g006600.1	protein GLUTAMINE DUMPER 2	GDU2	AT4G25760	Subunit of an amino acid transporter involved in the regulation of the amino acid metabolism	Impaired regulation of amino acid metabolism	Pratelli, Réjane, et al. "Stimulation of nonselective amino acid export by glutamine dumper proteins." Plant Physiology 152.2 (2010): 762-773.
Solyc07g045530.2	N/A	N/A	N/A	N/A	N/A	N/A
Solyc10g079810.1	N/A	N/A	N/A	N/A	N/A	N/A
Solyc03g045070.1	ammonium transporter 1 member 3	AMT1-3	AT3G24300	Transporter involved in ammonium uptake from the soil; Contributes with AMT1-1 to the overall ammonium uptake capacity in roots under nitrogen-deficiency conditions	Impaired ammonium uptake and transport; Impaired response to nitrogen-deficiency	Loqué, Dominique, et al. "Additive contribution of AMT1; 1 and AMT1; 3 to high-affinity ammonium uptake across the plasma membrane of nitrogen-deficient Arabidopsis roots." The Plant Journal 48.4 (2006): 522-534.
Solyc03g065050.1	ribosomal protein S12	N/A	N/A	N/A	N/A	N/A
Solyc05g007880.3	cyclic dof factor 1 isoform X1	CDF1	AT5G62430	Regulates a photoperiodic flowering response; Transcriptional repressor of 'CONSTANS' expression	Impaired photoperiodic flowering response; Decreased repression of CONSTANS expression	Imaizumi, Takato, et al. "FKF1 F-box protein mediates cyclic degradation of a repressor of CONSTANS in Arabidopsis." Science 309.5732 (2005): 293-297.
Solyc09g065390.1	protein MARD1	MARD1	AT3G63210	Acts as an adapter to facilitate the interaction of SnRK1 complex with effector proteins; Involved in seed dormancy control	Impaired seed dormancy	He, Yuehui, and Susheng Gan. "A novel zinc-finger protein with a proline-rich domain mediates ABA-regulated seed dormancy in Arabidopsis." Plant molecular biology 54.1 (2004): 1-9.
Solyc12g088670.2	low-temperature-induced cysteine proteinase precursor	N/A	N/A	Involved in cellular protein catabolic process	N/A	Spiegelman, Ziv, Amit Shahar, and Shmuel Wolf. "Down-regulation of SlCyp1 in the phloem reduces auxin response and photosynthetic rate in tomato (Solanum lycopersicum) plants." Plant signaling & behavior 12.6 (2017): e1338224.
Solyc12g099200.2	cell wall invertase inhibitor precursor	LOC107808322	N/A	Enzyme inhibitor activity	Decreased enzyme inhibition	Hothorn, Michael, and Klaus Scheffzek. "Multiple crystal forms of the cell-wall invertase inhibitor from tobacco support high conformational rigidity over a broad pH range." Acta Crystallographica Section D: Biological Crystallography 62.6 (2006): 665-670.
Solyc11g008530.2	endoribonuclease Dicer 2d isoform X1	AT3G03300	AT3G03300	Plays an essential role in transitive silencing of transgenes by processing secondary siRNAs; Plays a role in antiviral RNA silencing	Impaired post-transcriptional gene silencing; Impaired defense against viral pathogens	"DICER-LIKE2 plays a primary role in transitive silencing of transgenes in Arabidopsis."
Solyc12g036415.1	N/A	N/A	N/A	N/A	N/A	N/A
Solyc01g049685.1	N/A	N/A	N/A	N/A	N/A	N/A
Solyc01g049690.3	transcription factor Pur-alpha 1 isoform X2	PURA1	AT2G32080	Transcription factor that specifically binds the purine-rich double-stranded telomeric repeated sequence	Impaired gene expression	Tremousaygue, Dominique, et al. "Plant interstitial telomere motifs participate in the control of gene expression in root meristems." The Plant Journal 20.5 (1999): 553-561.

TomatoID	NCBI Protein Name	Gene ID	*A. Thaliana* Homolog	Uniprot Description	Putative Consequences for Infection	Citation
Solyc01g080870.3	protein NRT1/ PTR FAMILY 7.3	NPF7.3	AT1G32450	Low-affinity proton-dependent bidirectional nitrate transporter; Involved in nitrate loading into xylem	Impaired nitrate transport	Lin, Shan-Hua, et al. "Mutation of the Arabidopsis NRT1. 5 nitrate transporter causes defective root-to-shoot nitrate transport." The Plant Cell 20.9 (2008): 2514-2528.
Solyc10g053880.1	N/A	N/A	N/A	N/A	N/A	N/A
Solyc04g011495.1	N/A	N/A	N/A	N/A	N/A	N/A
Solyc07g063420.3	NAC domain transcription factor	N/A	N/A	N/A	N/A	N/A
Solyc01g081060.3	xyloglucan endotransglucosylase-hydrolase XTH5	XTH5	AT5G13870	Catalyzes xyloglucan endohydrolysis and endotransglycosylation; Essential constituent of the primary cell wall; Participates in cell wall construction	Impaired plant cell wall elongation; Impaired cell wall integrity	Hyodo, Hideki, et al. "Active gene expression of a xyloglucan endotransglucosylase/hydrolase gene, XTH9, in inflorescence apices is related to cell elongation in Arabidopsis thaliana." Plant molecular biology 52.2 (2003): 473-482.
Solyc11g021130.1	ribosomal protein S7	N/A	N/A	N/A	N/A	N/A
Solyc01g110580.2	auxin-responsive protein SAUR50	SAUR50	AT4G34760	Effector of hormonal and environmental signals in plant growth	Impaired response to hormonal and environmental signaling; Impaired growth/development	Ren, Hong, and William M. Gray. "SAUR proteins as effectors of hormonal and environmental signals in plant growth." Molecular plant 8.8 (2015): 1153-1164.
Solyc01g109800.2	uncharacterized protein LOC104645424	N/A	N/A	N/A	N/A	N/A
Solyc02g065220.3	geraniol 8-hydroxylase-like	CYP76B6	N/A	Hydroxylase involved in the biosynthesis of hydroxygeraniol, a precursor of the terpenoid indole alkaloids	Decreased biosynthesis of hydroxygeraniol	Collu, Graziella, et al. "Activity of the cytochrome P450 enzyme geraniol 10-hydroxylase and alkaloid production in plant cell cultures." Plant Science 162.1 (2002): 165-172.
Solyc11g012690.2	heavy metal-associated isoprenylated plant protein 7-like isoform X2	HIPP07	AT5G63530	Heavy metal-binding protein involved in metal ion transport	Impaired metal ion transport	Dykema, Philip E., et al. "A new class of proteins capable of binding transition metals." Plant molecular biology 41.1 (1999): 139-150.
Solyc04g051470.1	uncharacterized protein LOC101261064	N/A	N/A	N/A	N/A	N/A
Solyc08g079430.3	copper amine oxidase precursor	N/A	N/A	N/A	N/A	N/A
Solyc04g071780.3	cytochrome P450 71A1	CYP71A1	N/A	Involved in the metabolism of compounds associated with the development of flavor in the ripening fruit process	Impaired fruit ripening	O'Keefe, Daniel P., and Kenneth J. Leto. "Cytochrome P-450 from the mesocarp of avocado (Persea americana)." Plant physiology 89.4 (1989): 1141-1149.

TomatoID	NCBI Protein Name	Gene ID	*A. Thaliana* Homolog	Uniprot Description	Putative Consequences for Infection	Citation
Solyc01g080460.3	pyruvate, phosphate dikinase, chloroplastic	PPDK	N/A	Formation of phosphoenolpyruvate, which is the primary acceptor of CO2 in C4 and Crassulacean plants	Impaired primary metabolism	Rosche, Elke, and Peter Westhoff. "Primary structure of pyruvate, orthophosphate dikinase in the dicotyledonous C4 plant Flaveria trinervia." FEBS letters 273.1-2 (1990): 116-121.
Solyc07g065410.1	uncharacterized protein LOC101261249	N/A	N/A	N/A	N/A	N/A
Solyc12g087870.2	purine permease 3-like	PUP3	AT1G28220	Involved in transport of purine derivatives during pollen germination and tube elongation	Impaired transport of purine derivatives during pollen germination and tube elongation	Bürkle, Lukas, et al. "Transport of cytokinins mediated by purine transporters of the PUP family expressed in phloem, hydathodes, and pollen of Arabidopsis." The Plant Journal 34.1 (2003): 13-26.
Solyc05g012030.1	protein BIG GRAIN 1-like E	AT1G69160	AT1G69160	Involved in auxin transport; Regulator of the auxin signaling pathway	Impaired auxin transport; Impaired regulation of auxin signaling	Culligan, Kevin M., et al. "ATR and ATM play both distinct and additive roles in response to ionizing radiation." The Plant Journal 48.6 (2006): 947-961.
Solyc01g104910.3	uncharacterized protein LOC101246968	N/A	N/A	N/A	N/A	N/A
Solyc01g107460.2	uncharacterized protein LOC101248432	N/A	N/A	N/A	N/A	N/A
Solyc04g011670.3	TGACG-sequence-specific DNA-binding protein TGA-1A isoform X2	TGA1A	N/A	Promotes auxin- and SA-inducible transcription	Impaired auxin- and SA-inducible transcription	Pascuzzi, Pete, et al. "Auxin-induced stress potentiates trans-activation by a conserved plant basic/leucine-zipper factor." Journal of Biological Chemistry 273.41 (1998): 26631-26637.
Solyc05g013530.3	uncharacterized protein LOC101267605 isoform X1	N/A	N/A	N/A	N/A	N/A
Solyc05g041360.2	maturase K	matK	ATCG00040	Assists in splicing its own and other chloroplast group II introns	Impaired chloroplastic gene splicing	Sato, Shusei, et al. "Complete structure of the chloroplast genome of Arabidopsis thaliana." DNA research 6.5 (1999): 283-290.
Solyc08g074490.3	uncharacterized protein At4g15970	AT4G15970	AT4G15970	N/A	N/A	N/A
Solyc09g061390.1	maturase K	matK	ATCG00040	Assists in splicing its own and other chloroplast group II introns	Impaired chloroplastic gene splicing	Sato, Shusei, et al. "Complete structure of the chloroplast genome of Arabidopsis thaliana." DNA research 6.5 (1999): 283-290.
Solyc11g073120.2	transcription factor MYB48	MYB48	AT3G46130	Transcriptional regulator	N/A	Riechmann, José Luis, et al. "Arabidopsis transcription factors: genome-wide comparative analysis among eukaryotes." Science 290.5499 (2000): 2105-2110.
Solyc10g078570.1	uncharacterized protein LOC101265504	N/A	N/A	N/A	N/A	N/A

TomatoID	NCBI Protein Name	Gene ID	*A. Thaliana* Homolog	Uniprot Description	Putative Consequences for Infection	Citation
Solyc01g066260.2	N/A	N/A	N/A	N/A	N/A	N/A
Solyc06g005330.3	transcription factor MYB48-like	MYB48	AT3G46130	Transcriptional regulator	N/A	Riechmann, José Luis, et al. "Arabidopsis transcription factors: genome-wide comparative analysis among eukaryotes." Science 290.5499 (2000): 2105-2110.
Solyc08g075570.3	urea-proton symporter DUR3	DUR3	AT5G45380	High-affinity urea-proton symporter involved in the active transport of urea across the plasma membrane into root cells	Impaired urea uptake and transport	Liu, Lai-Hua, et al. "AtDUR3 encodes a new type of high-affinity urea/H+ symporter in Arabidopsis." The Plant Cell 15.3 (2003): 790-800.
Solyc05g047440.1	Ycf1	Ycf1	N/A	Part of an intermediate translocation complex acting as a protein-conducting channel at the inner chloroplastic envelope	Decreased protein precursor import into chloroplasts	Kikuchi, Shingo, et al. "Uncovering the protein translocon at the chloroplast inner envelope membrane." Science 339.6119 (2013): 571-574.
Solyc03g115920.3	probable E3 ubiquitin-protein ligase RNF217	RNF217	N/A	E3 ubiquitin-protein ligase which accepts ubiquitin from E2 ubiquitin-conjugating enzymes in the form of a thioester	Impaired primary metabolism	Ohmura-Hoshino, Mari, et al. "A novel family of membrane-bound E3 ubiquitin ligases." Journal of biochemistry 140.2 (2006): 147-154.
Solyc06g068500.3	chaperone protein dnaJ 8, chloroplastic-like	ATJ8	AT1G80920	Plays a continuous role in plant development and the structural organization of compartments	Impaired plant development and structural organization	Nanjo, Tokihiko, et al. "Toxicity of free proline revealed in an Arabidopsis T-DNA-tagged mutant deficient in proline dehydrogenase." Plant and Cell Physiology 44.5 (2003): 541-548.
Solyc01g014250.2	N/A	N/A	N/A	N/A	N/A	N/A
Solyc05g054750.3	uncharacterized protein LOC101266225 isoform X1	N/A	N/A	N/A	N/A	N/A
Solyc08g068850.3	uncharacterized protein LOC101268894 isoform 1	N/A	N/A	N/A	N/A	N/A
Solyc11g069450.1	uncharacterized protein LOC101259309	N/A	N/A	N/A	N/A	N/A
Solyc11g032190.1	N/A	N/A	N/A	N/A	N/A	N/A
Solyc12g008380.1	uncharacterized protein LOC101257239	N/A	N/A	N/A	N/A	N/A
Solyc10g047090.2	ribosomal protein L2	N/A	N/A	N/A	N/A	N/A
Solyc11g069940.1	glutaredoxin-C6	GRXC6	AT4G33040	Involved in regulation of plant defense responses	Impaired regulation of plant defense	Brodersen, Peter, et al. "Arabidopsis MAP kinase 4 regulates salicylic acid-and jasmonic acid/ethylene-dependent responses via EDS1 and PAD4." The Plant Journal 47.4 (2006): 532-546.

TomatoID	NCBI Protein Name	Gene ID	*A. Thaliana* Homolog	Uniprot Description	Putative Consequences for Infection	Citation
Solyc05g005870.3	nodulin-related MtN21 family protein	UMAMIT16-psi	AT4G24980	Transmembrane transporter activity	N/A	Cheng, Chia-Yi, et al. "Araport11: a complete reannotation of the Arabidopsis thaliana reference genome." The Plant Journal 89.4 (2017): 789-804.
Solyc10g047390.1	Ycf1	Ycf1	N/A	Involved in protein precursor import into chloroplasts; Translocation complex acting as a protein-conducting channel	Decreased protein precursor import into chloroplasts	Kikuchi, Shingo, et al. "Uncovering the protein translocon at the chloroplast inner envelope membrane." Science 339.6119 (2013): 571-574.
Solyc09g008200.3	heavy metal-associated isoprenylated plant protein 5	HIPP05	AT2G36950	Heavy-metal-binding protein involved in metal ion tranpsport; Involved in disease resistance	Impaired metal ion transport; Impaired disease resistance	Cooper, Bret, et al. "A network of rice genes associated with stress response and seed development." Proceedings of the National Academy of Sciences 100.8 (2003): 4945-4950.
Solyc11g021240.2	hypothetical chloroplast RF1	ycf1a	N/A	Kinase activity	N/A	Lemieux, Claude, et al. "Chloroplast phylogenomic analysis of chlorophyte green algae identifies a novel lineage sister to the Sphaeropleales (Chlorophyceae)." BMC Evolutionary Biology 15.1 (2015): 264.
Solyc05g026460.1	N/A	N/A	N/A	N/A	N/A	N/A
Solyc06g005320.1	transcription factor MYB48-like	MYB48	AT3G46130	Transcriptional regulator	N/A	Riechmann, José Luis, et al. "Arabidopsis transcription factors: genome-wide comparative analysis among eukaryotes." Science 290.5499 (2000): 2105-2110.
Solyc06g066530.1	uncharacterized protein LOC101252498	N/A	N/A	N/A	N/A	N/A
Solyc11g056500.2	uncharacterized protein LOC104644731	N/A	N/A	N/A	N/A	N/A
Solyc12g035550.1	Ycf1	Ycf1	N/A	Involved in protein precursor import into chloroplasts; Protein-conducting channel at the inner envelope	Decreased protein precursor import into chloroplasts	Kikuchi, Shingo, et al. "Uncovering the protein translocon at the chloroplast inner envelope membrane." Science 339.6119 (2013): 571-574.
Solyc08g079230.1	14 kDa proline-rich protein DC2.15-like	N/A	N/A	Involved with the initiation of embryogenesis or with the metabolic changes produced by the removal of auxins	Impaired embryogenesis; Impaired response to auxin signaling	Aleith, F., and G. Richter. "Gene expression during induction of somatic embryogenesis in carrot cell suspensions." Planta 183.1 (1991): 17-24.
Solyc09g009130.3	putative phytosulfokine peptide precursor	N/A	N/A	N/A	N/A	N/A
Solyc08g016000.1	Ycf1	Ycf1	N/A	Involved in protein precursor import into chloroplasts; Translocation complex acting as a protein-conducting channel	Decreased protein precursor import into chloroplasts	Kikuchi, Shingo, et al. "Uncovering the protein translocon at the chloroplast inner envelope membrane." Science 339.6119 (2013): 571-574.

TomatoID	NCBI Protein Name	Gene ID	*A. Thaliana* Homolog	Uniprot Description	Putative Consequences for Infection	Citation
Solyc07g006310.1	transcription factor IBH1-like 1	IBL1	AT4G30410	Acts as transcriptional repressor that negatively regulates cell and organ elongation in response to gibberellin and brassinosteroid signaling	Impaired regulation of cell and organ elongation in response to gibberellin and brassinosteroid signaling	Zhiponova, Miroslava K., et al. "Helix–loop–helix/basic helix–loop–helix transcription factor network represses cell elongation in Arabidopsis through an apparent incoherent feed-forward loop." Proceedings of the National Academy of Sciences 111.7 (2014): 2824-2829.
Solyc09g010530.3	cation/H(+) antiporter 20	CHX20	AT3G53720	Operates as a K+/H+ antiporter that maintains K+ homeostasis in guard cells; Helps regulate pH; Plays a critical role in osmoregulation through via stomatal opening	Impaired K+ homeostasis and pH homeostasis; Impaired osmoregulation	Padmanaban, Senthilkumar, et al. "Participation of endomembrane cation/H+ exchanger AtCHX20 in osmoregulation of guard cells." Plant Physiology 144.1 (2007): 82-93.
Solyc11g021300.1	Ycf1	Ycf1	N/A	Involved in protein precursor import into chloroplasts; Translocation complex acting as a protein-conducting channel	Decreased protein precursor import into chloroplasts	Kikuchi, Shingo, et al. "Uncovering the protein translocon at the chloroplast inner envelope membrane." Science 339.6119 (2013): 571-574.
Solyc11g021260.1	Ycf1	Ycf1	N/A	Involved in protein precursor import into chloroplasts; Translocation complex acting as a protein-conducting channel	Decreased protein precursor import into chloroplasts	Kikuchi, Shingo, et al. "Uncovering the protein translocon at the chloroplast inner envelope membrane." Science 339.6119 (2013): 571-574.
Solyc06g005310.3	transcription factor MYB48-like	MYB48	AT3G46130	Transcriptional regulator	N/A	Riechmann, José Luis, et al. "Arabidopsis transcription factors: genome-wide comparative analysis among eukaryotes." Science 290.5499 (2000): 2105-2110.
Solyc01g086660.2	N/A	N/A	N/A	N/A	N/A	N/A
Solyc11g021290.2	Ycf1	Ycf1	N/A	Involved in protein precursor import into chloroplasts; Translocation complex acting as a protein-conducting channel	Decreased protein precursor import into chloroplasts	Kikuchi, Shingo, et al. "Uncovering the protein translocon at the chloroplast inner envelope membrane." Science 339.6119 (2013): 571-574.
Solyc08g065610.3	vacuolar processing enzyme VPE3 precursor	VPE3	N/A	Proteolysis involved in cellular protein catabolic process	N/A	Radchuk, Volodymyr, et al. "Development of maternal seed tissue in barley is mediated by regulated cell expansion and cell disintegration and coordinated with endosperm growth." Journal of experimental botany 62.3 (2011): 1217-1227.
Solyc10g049470.1	Ycf1	Ycf1	N/A	Involved in protein precursor import into chloroplasts; Translocation complex acting as a protein-conducting channel	Decreased protein precursor import into chloroplasts	Kikuchi, Shingo, et al. "Uncovering the protein translocon at the chloroplast inner envelope membrane." Science 339.6119 (2013): 571-574.
Solyc01g109090.2	N/A	N/A	N/A	N/A	N/A	N/A
Solyc11g021280.1	Ycf1	Ycf1	N/A	Involved in protein precursor import into chloroplasts; Translocation complex acting as a protein-conducting channel	Decreased protein precursor import into chloroplasts	Kikuchi, Shingo, et al. "Uncovering the protein translocon at the chloroplast inner envelope membrane." Science 339.6119 (2013): 571-574.

TomatoID	NCBI Protein Name	Gene ID	*A. Thaliana* Homolog	Uniprot Description	Putative Consequences for Infection	Citation
Solyc11g021270.1	Ycf1	Ycf1	N/A	Involved in protein precursor import into chloroplasts; Translocation complex acting as a protein-conducting channel	Decreased protein precursor import into chloroplasts	Kikuchi, Shingo, et al. "Uncovering the protein translocon at the chloroplast inner envelope membrane." Science 339.6119 (2013): 571-574.
Solyc04g054745.1	N/A	N/A	N/A	N/A	N/A	N/A
Solyc09g065790.1	clp protease proteolytic subunit	CLPR2	AT1G12410	Required for chloroplast development and integrity; Involved in the regulation of plastoglobules formation	Impaired chloroplast development and integrity; Impaired regulation of plastoglobule formation	Rudella, Andrea, et al. "Downregulation of ClpR2 leads to reduced accumulation of the ClpPRS protease complex and defects in chloroplast biogenesis in Arabidopsis." The Plant Cell 18.7 (2006): 1704-1721.
Solyc09g064580.2	photosystem II protein M	psbM	N/A	Component of the core complex of photosystem II; Light-driven water:plastoquinone oxidoreductase that uses light energy to abstract electrons from H2O	Impaired light capture and photosynthesis	Kawakami, Keisuke, et al. "Location of chloride and its possible functions in oxygen-evolving photosystem II revealed by X-ray crystallography." Proceedings of the National Academy of Sciences 106.21 (2009): 8567-8572.
Solyc01g017220.1	ATP synthase CF1 epsilon subunit	atpE	ATCG00470	Produces ATP from ADP in the presence of a proton gradient across the membrane	Impaired ATP production	Goulas, Estelle, et al. "The chloroplast lumen and stromal proteomes of Arabidopsis thaliana show differential sensitivity to short-and long-term exposure to low temperature." The Plant Journal 47.5 (2006): 720-734.
Solyc04g039850.1	ATP synthase beta subunit	atpB	ATCG00480	Produces ATP from ADP in the presence of a proton gradient across the membrane	Impaired ATP production	Goulas, Estelle, et al. "The chloroplast lumen and stromal proteomes of Arabidopsis thaliana show differential sensitivity to short-and long-term exposure to low temperature." The Plant Journal 47.5 (2006): 720-734.
Solyc06g007515.1	N/A	N/A	N/A	N/A	N/A	N/A
