# Supplementary material for: Effects of ‘Candidatus Liberibacter solanacearum’ haplotypes A and B on tomato gene expression and geotropism
Source: BMC Plant Biol. 2022 Mar 30;22:156. doi: 10.1186/s12870-022-03505-z (PMC8966271; doi:10.1186/s12870-022-03505-z)
Supplement: Supplementary file 3 — Additional file 3: Table S3. KEGG Pathway search analysis of tomato plant DEGs associated with Lso infection. Among the 238-infection associated DEGs also involved in a KEGG Pathway, 54 were involved in metabolic pathways, 44 were involved in the biosynthesis of (various) secondary metabolites, 9 were involved in phenylpropanoid biosynthesis, 9 were involved in the biosynthesis of amino acids, 7 were involved in plant hormone signal transduction, 5 were involved in the MAPK signaling pathway, 5 were involved in carbon metabolism, 4 were involved in flavonoid biosynthesis, 4 were involved in alpha-Linolenic acid metabolism, 4 were involved in stilbenoid, diarylheptanoid and gingerol biosynthesis, and 4 were involved in Arginine and proline metabolism. Several more DEGs were involved in other pathways (e.g., circadian rhythm photosynthesis), but with less representation. [file 12870_2022_3505_MOESM3_ESM.pdf]

KEGG Pathway	Pathway Name	KEGG Gene ID	Gene Name
ath01100	Metabolic pathways	ath:AT1G05260	RCI3; Peroxidase superfamily protein
ath01100	Metabolic pathways	ath:AT1G07440	NAD(P)-binding Rossmann-fold superfamily protein
ath01100	Metabolic pathways	ath:AT1G08250	ADT6; arogenate dehydratase 6
ath01100	Metabolic pathways	ath:AT1G15710	prephenate dehydrogenase family protein
ath01100	Metabolic pathways	ath:AT1G20510	OPCL1; OPC-8:0 CoA ligase1
ath01100	Metabolic pathways	ath:AT1G26570	UGD1; UDP-glucose dehydrogenase 1
ath01100	Metabolic pathways	ath:AT1G29930	CAB1; chlorophyll A/B binding protein 1
ath01100	Metabolic pathways	ath:AT1G32550	FdC2; 2Fe-2S ferredoxin-like superfamily protein
ath01100	Metabolic pathways	ath:AT1G51680	4CL1; 4-coumarate:CoA ligase 1
ath01100	Metabolic pathways	ath:AT1G52760	LysoPL2; lysophospholipase 2
ath01100	Metabolic pathways	ath:AT1G60810	ACLA-2; ATP-citrate lyase A-2
ath01100	Metabolic pathways	ath:AT1G67980	CCOAMT; caffeoyl-CoA 3-O-methyltransferase
ath01100	Metabolic pathways	ath:AT1G71695	Peroxidase superfamily protein
ath01100	Metabolic pathways	ath:AT1G76490	HMG1; hydroxy methylglutaryl CoA reductase 1
ath01100	Metabolic pathways	ath:AT2G01290	RPI2; ribose-5-phosphate isomerase 2
ath01100	Metabolic pathways	ath:AT2G06050	OPR3; oxophytodienoate-reductase 3
ath01100	Metabolic pathways	ath:AT2G16500	ADC1; arginine decarboxylase 1
ath01100	Metabolic pathways	ath:AT2G21330	FBA1; fructose-bisphosphate aldolase 1
ath01100	Metabolic pathways	ath:AT2G24200	LAP1; Cytosol aminopeptidase family protein
ath01100	Metabolic pathways	ath:AT2G36880	MAT3; methionine adenosyltransferase 3
ath01100	Metabolic pathways	ath:AT2G38050	DET2; 3-oxo-5-alpha-steroid 4-dehydrogenase family protein
ath01100	Metabolic pathways	ath:AT2G38400	AGT3; alanine:glyoxylate aminotransferase 3
ath01100	Metabolic pathways	ath:AT3G07010	Pectin lyase-like superfamily protein
ath01100	Metabolic pathways	ath:AT3G14310	PME3; pectin methylesterase 3
ath01100	Metabolic pathways	ath:AT3G25400	dCTP pyrophosphatase-like protein
ath01100	Metabolic pathways	ath:AT3G25760	AOC1; allene oxide cyclase 1
ath01100	Metabolic pathways	ath:AT3G45770	Polyketide synthase, enoylreductase family protein
ath01100	Metabolic pathways	ath:AT3G53260	PAL2; phenylalanine ammonia-lyase 2
ath01100	Metabolic pathways	ath:AT3G54420	EP3; homolog of carrot EP3-3 chitinase
ath01100	Metabolic pathways	ath:AT3G61510	ACS1; ACC synthase 1
ath01100	Metabolic pathways	ath:AT3G62750	BGLU8; beta glucosidase 8
ath01100	Metabolic pathways	ath:AT4G01850	SAM-2; S-adenosylmethionine synthetase 2
ath01100	Metabolic pathways	ath:AT4G08870	ARGAH2; Arginase/deacetylase superfamily protein
ath01100	Metabolic pathways	ath:AT4G17830	Peptidase M20/M25/M40 family protein
ath01100	Metabolic pathways	ath:AT4G21990	APR3; APS reductase 3
ath01100	Metabolic pathways	ath:AT4G25420	GA20OX1; 2-oxoglutarate (2OG) and Fe(II)-dependent oxygenase superfamily protein
ath01100	Metabolic pathways	ath:AT4G25700	BETA-OHASE_1; beta-hydroxylase 1
ath01100	Metabolic pathways	ath:AT4G26220	S-adenosyl-L-methionine-dependent methyltransferases superfamily protein
ath01100	Metabolic pathways	ath:AT4G30920	LAP2; Cytosol aminopeptidase family protein
ath01100	Metabolic pathways	ath:AT4G34050	CCoAOMT1; S-adenosyl-L-methionine-dependent methyltransferases superfamily protein
ath01100	Metabolic pathways	ath:AT4G34880	Amidase family protein
ath01100	Metabolic pathways	ath:AT5G04950	NAS1; nicotianamine synthase 1
ath01100	Metabolic pathways	ath:AT5G13700	PAO1; polyamine oxidase 1
ath01100	Metabolic pathways	ath:AT5G41670	6-phosphogluconate dehydrogenase family protein

KEGG Pathway	Pathway Name	KEGG Gene ID	Gene Name
ath01100	Metabolic pathways	ath:AT5G42650	AOS; allene oxide synthase
ath01100	Metabolic pathways	ath:AT5G43860	CLH2; chlorophyllase 2
ath01100	Metabolic pathways	ath:AT5G48230	ACAT2; acetoacetyl-CoA thiolase 2
ath01100	Metabolic pathways	ath:AT5G48300	ADG1; ADP glucose pyrophosphorylase 1
ath01100	Metabolic pathways	ath:AT5G48930	HCT; hydroxycinnamoyl-CoA shikimate/quinate hydroxycinnamoyl transferase
ath01100	Metabolic pathways	ath:AT5G54810	TSB1; tryptophan synthase beta-subunit 1
ath01100	Metabolic pathways	ath:AT5G58700	PLC4; phosphatidylinositol-speciwc phospholipase C4
ath01100	Metabolic pathways	ath:ArthCp028	atpE; ATP synthase CF1 epsilon subunit
ath01100	Metabolic pathways	ath:ArthCp029	atpB; ATP synthase CF1 beta subunit
ath01100	Metabolic pathways	ath:ArthCp030	rbcL; ribulose-1,5-bisphosphate carboxylase/oxygenase large subunit
ath01110	Biosynthesis of secondary metabolites	ath:AT1G05260	RCI3; Peroxidase superfamily protein
ath01110	Biosynthesis of secondary metabolites	ath:AT1G07440	NAD(P)-binding Rossmann-fold superfamily protein
ath01110	Biosynthesis of secondary metabolites	ath:AT1G08250	ADT6; arogenate dehydratase 6
ath01110	Biosynthesis of secondary metabolites	ath:AT1G15710	prephenate dehydrogenase family protein
ath01110	Biosynthesis of secondary metabolites	ath:AT1G20510	OPCL1; OPC-8:0 CoA ligase1
ath01110	Biosynthesis of secondary metabolites	ath:AT1G51680	4CL1; 4-coumarate:CoA ligase 1
ath01110	Biosynthesis of secondary metabolites	ath:AT1G52760	LysoPL2; lysophospholipase 2
ath01110	Biosynthesis of secondary metabolites	ath:AT1G60810	ACLA-2; ATP-citrate lyase A-2
ath01110	Biosynthesis of secondary metabolites	ath:AT1G67980	CCOAMT; caffeoyl-CoA 3-O-methyltransferase
ath01110	Biosynthesis of secondary metabolites	ath:AT1G71695	Peroxidase superfamily protein
ath01110	Biosynthesis of secondary metabolites	ath:AT1G76490	HMG1; hydroxy methylglutaryl CoA reductase 1
ath01110	Biosynthesis of secondary metabolites	ath:AT2G01290	RPI2; ribose-5-phosphate isomerase 2
ath01110	Biosynthesis of secondary metabolites	ath:AT2G06050	OPR3; oxophytodienoate-reductase 3
ath01110	Biosynthesis of secondary metabolites	ath:AT2G21330	FBA1; fructose-bisphosphate aldolase 1
ath01110	Biosynthesis of secondary metabolites	ath:AT2G36750	UGT73C1; UDP-glucosyl transferase 73C1
ath01110	Biosynthesis of secondary metabolites	ath:AT2G36880	MAT3; methionine adenosyltransferase 3
ath01110	Biosynthesis of secondary metabolites	ath:AT2G38050	DET2; 3-oxo-5-alpha-steroid 4-dehydrogenase family protein
ath01110	Biosynthesis of secondary metabolites	ath:AT2G38400	AGT3; alanine:glyoxylate aminotransferase 3
ath01110	Biosynthesis of secondary metabolites	ath:AT3G25760	AOC1; allene oxide cyclase 1
ath01110	Biosynthesis of secondary metabolites	ath:AT3G53260	PAL2; phenylalanine ammonia-lyase 2
ath01110	Biosynthesis of secondary metabolites	ath:AT3G61220	SDR1; NAD(P)-binding Rossmann-fold superfamily protein
ath01110	Biosynthesis of secondary metabolites	ath:AT3G61510	ACS1; ACC synthase 1
ath01110	Biosynthesis of secondary metabolites	ath:AT3G62750	BGLU8; beta glucosidase 8
ath01110	Biosynthesis of secondary metabolites	ath:AT4G01850	SAM-2; S-adenosylmethionine synthetase 2
ath01110	Biosynthesis of secondary metabolites	ath:AT4G08870	ARGAH2; Arginase/deacetylase superfamily protein
ath01110	Biosynthesis of secondary metabolites	ath:AT4G17830	Peptidase M20/M25/M40 family protein
ath01110	Biosynthesis of secondary metabolites	ath:AT4G25420	GA20OX1; 2-oxoglutarate (2OG) and Fe(II)-dependent oxygenase superfamily protein
ath01110	Biosynthesis of secondary metabolites	ath:AT4G25700	BETA-OHASE_1; beta-hydroxylase 1
ath01110	Biosynthesis of secondary metabolites	ath:AT4G26220	S-adenosyl-L-methionine-dependent methyltransferases superfamily protein
ath01110	Biosynthesis of secondary metabolites	ath:AT4G34050	CCoAOMT1; S-adenosyl-L-methionine-dependent methyltransferases superfamily protein
ath01110	Biosynthesis of secondary metabolites	ath:AT5G04950	NAS1; nicotianamine synthase 1
ath01110	Biosynthesis of secondary metabolites	ath:AT5G41670	6-phosphogluconate dehydrogenase family protein
ath01110	Biosynthesis of secondary metabolites	ath:AT5G42650	AOS; allene oxide synthase

KEGG Pathway	Pathway Name	KEGG Gene ID	Gene Name
ath01110	Biosynthesis of secondary metabolites	ath:AT5G43860	CLH2; chlorophyllase 2
ath01110	Biosynthesis of secondary metabolites	ath:AT5G45340	CYP707A3; cytochrome P450, family 707, subfamily A, polypeptide 3
ath01110	Biosynthesis of secondary metabolites	ath:AT5G48230	ACAT2; acetoacetyl-CoA thiolase 2
ath01110	Biosynthesis of secondary metabolites	ath:AT5G48300	ADG1; ADP glucose pyrophosphorylase 1
ath01110	Biosynthesis of secondary metabolites	ath:AT5G48930	HCT; hydroxycinnamoyl-CoA shikimate/quinate hydroxycinnamoyl transferase
ath01110	Biosynthesis of secondary metabolites	ath:AT5G54810	TSB1; tryptophan synthase beta-subunit 1
ath01110	Biosynthesis of secondary metabolites	ath:ArthCp030	rbcL; ribulose-1,5-bisphosphate carboxylase/oxygenase large subunit
ath00940	Phenylpropanoid biosynthesis	ath:AT1G05260	RCI3; Peroxidase superfamily protein
ath00940	Phenylpropanoid biosynthesis	ath:AT1G51680	4CL1; 4-coumarate:CoA ligase 1
ath00940	Phenylpropanoid biosynthesis	ath:AT1G52760	LysoPL2; lysophospholipase 2
ath00940	Phenylpropanoid biosynthesis	ath:AT1G67980	CCOAMT; caffeoyl-CoA 3-O-methyltransferase
ath00940	Phenylpropanoid biosynthesis	ath:AT1G71695	Peroxidase superfamily protein
ath00940	Phenylpropanoid biosynthesis	ath:AT3G53260	PAL2; phenylalanine ammonia-lyase 2
ath00940	Phenylpropanoid biosynthesis	ath:AT4G26220	S-adenosyl-L-methionine-dependent methyltransferases superfamily protein
ath00940	Phenylpropanoid biosynthesis	ath:AT4G34050	CCoAOMT1; S-adenosyl-L-methionine-dependent methyltransferases superfamily protein
ath00940	Phenylpropanoid biosynthesis	ath:AT5G48930	HCT; hydroxycinnamoyl-CoA shikimate/quinate hydroxycinnamoyl transferase
ath01230	Biosynthesis of amino acids	ath:AT1G08250	ADT6; arogenate dehydratase 6
ath01230	Biosynthesis of amino acids	ath:AT1G15710	prephenate dehydrogenase family protein
ath01230	Biosynthesis of amino acids	ath:AT2G01290	RPI2; ribose-5-phosphate isomerase 2
ath01230	Biosynthesis of amino acids	ath:AT2G21330	FBA1; fructose-bisphosphate aldolase 1
ath01230	Biosynthesis of amino acids	ath:AT2G36880	MAT3; methionine adenosyltransferase 3
ath01230	Biosynthesis of amino acids	ath:AT4G01850	SAM-2; S-adenosylmethionine synthetase 2
ath01230	Biosynthesis of amino acids	ath:AT4G08870	ARGAH2; Arginase/deacetylase superfamily protein
ath01230	Biosynthesis of amino acids	ath:AT4G17830	Peptidase M20/M25/M40 family protein
ath01230	Biosynthesis of amino acids	ath:AT5G54810	TSB1; tryptophan synthase beta-subunit 1
ath04075	Plant hormone signal transduction	ath:AT1G32640	MYC2; Basic helix-loop-helix (bHLH) DNA-binding family protein
ath04075	Plant hormone signal transduction	ath:AT1G74950	TIFY10B; TIFY domain/Divergent CCT motif family protein
ath04075	Plant hormone signal transduction	ath:AT3G11410	PP2CA; protein phosphatase 2CA
ath04075	Plant hormone signal transduction	ath:AT3G16360	AHP4; HPT phosphotransmitter 4
ath04075	Plant hormone signal transduction	ath:AT4G03400	DFL2; Auxin-responsive GH3 family protein
ath04075	Plant hormone signal transduction	ath:AT4G34760	SAUR-like auxin-responsive protein family
ath04075	Plant hormone signal transduction	ath:AT4G37390	BRU6; Auxin-responsive GH3 family protein
ath04016	MAPK signaling pathway	ath:AT1G05100	MAPKKK18; mitogen-activated protein kinase kinase kinase 18
ath04016	MAPK signaling pathway	ath:AT1G32640	MYC2; Basic helix-loop-helix (bHLH) DNA-binding family protein
ath04016	MAPK signaling pathway	ath:AT2G32510	MAPKKK17; mitogen-activated protein kinase kinase kinase 17
ath04016	MAPK signaling pathway	ath:AT3G11410	PP2CA; protein phosphatase 2CA
ath04016	MAPK signaling pathway	ath:AT3G61510	ACS1; ACC synthase 1
ath00270	Cysteine and methionine metabolism	ath:AT2G36880	MAT3; methionine adenosyltransferase 3
ath00270	Cysteine and methionine metabolism	ath:AT2G38400	AGT3; alanine:glyoxylate aminotransferase 3
ath00270	Cysteine and methionine metabolism	ath:AT3G61510	ACS1; ACC synthase 1
ath00270	Cysteine and methionine metabolism	ath:AT4G01850	SAM-2; S-adenosylmethionine synthetase 2

KEGG Pathway	Pathway Name	KEGG Gene ID	Gene Name
ath00270	Cysteine and methionine metabolism	ath:AT5G04950	NAS1; nicotianamine synthase 1
ath01200	Carbon metabolism	ath:AT2G01290	RPI2; ribose-5-phosphate isomerase 2
ath01200	Carbon metabolism	ath:AT2G21330	FBA1; fructose-bisphosphate aldolase 1
ath01200	Carbon metabolism	ath:AT5G41670	6-phosphogluconate dehydrogenase family protein
ath01200	Carbon metabolism	ath:AT5G48230	ACAT2; acetoacetyl-CoA thiolase 2
ath01200	Carbon metabolism	ath:ArthCp030	rbcL; ribulose-1,5-bisphosphate carboxylase/oxygenase large subunit
ath00941	Flavonoid biosynthesis	ath:AT1G67980	CCOAMT; caffeoyl-CoA 3-O-methyltransferase
ath00941	Flavonoid biosynthesis	ath:AT4G26220	S-adenosyl-L-methionine-dependent methyltransferases superfamily protein
ath00941	Flavonoid biosynthesis	ath:AT4G34050	CCoAOMT1; S-adenosyl-L-methionine-dependent methyltransferases superfamily protein
ath00941	Flavonoid biosynthesis	ath:AT5G48930	HCT; hydroxycinnamoyl-CoA shikimate/quinate hydroxycinnamoyl transferase
ath00592	alpha-Linolenic acid metabolism	ath:AT1G20510	OPCL1; OPC-8:0 CoA ligase1
ath00592	alpha-Linolenic acid metabolism	ath:AT2G06050	OPR3; oxophytodienoate-reductase 3
ath00592	alpha-Linolenic acid metabolism	ath:AT3G25760	AOC1; allene oxide cyclase 1
ath00592	alpha-Linolenic acid metabolism	ath:AT5G42650	AOS; allene oxide synthase
ath00945	Stilbenoid, diarylheptanoid and gingerol biosynthesis	ath:AT1G67980	CCOAMT; caffeoyl-CoA 3-O-methyltransferase
ath00945	Stilbenoid, diarylheptanoid and gingerol biosynthesis	ath:AT4G26220	S-adenosyl-L-methionine-dependent methyltransferases superfamily protein
ath00945	Stilbenoid, diarylheptanoid and gingerol biosynthesis	ath:AT4G34050	CCoAOMT1; S-adenosyl-L-methionine-dependent methyltransferases superfamily protein
ath00945	Stilbenoid, diarylheptanoid and gingerol biosynthesis	ath:AT5G48930	HCT; hydroxycinnamoyl-CoA shikimate/quinate hydroxycinnamoyl transferase
ath00330	Arginine and proline metabolism	ath:AT2G16500	ADC1; arginine decarboxylase 1
ath00330	Arginine and proline metabolism	ath:AT4G08870	ARGAH2; Arginase/deacetylase superfamily protein
ath00330	Arginine and proline metabolism	ath:AT4G34880	Amidase family protein
ath00330	Arginine and proline metabolism	ath:AT5G13700	PAO1; polyamine oxidase 1
ath00999	Biosynthesis of various plant secondary metabolites	ath:AT2G36880	MAT3; methionine adenosyltransferase 3
ath00999	Biosynthesis of various plant secondary metabolites	ath:AT3G62750	BGLU8; beta glucosidase 8
ath00999	Biosynthesis of various plant secondary metabolites	ath:AT4G01850	SAM-2; S-adenosylmethionine synthetase 2
ath00999	Biosynthesis of various plant secondary metabolites	ath:AT5G04950	NAS1; nicotianamine synthase 1
ath00190	Oxidative phosphorylation	ath:AT1G01050	PPa1; pyrophosphorylase 1
ath00190	Oxidative phosphorylation	ath:ArthCp028	atpE; ATP synthase CF1 epsilon subunit
ath00190	Oxidative phosphorylation	ath:ArthCp029	atpB; ATP synthase CF1 beta subunit
ath00400	Phenylalanine, tyrosine and tryptophan biosynthesis	ath:AT1G08250	ADT6; arogenate dehydratase 6
ath00400	Phenylalanine, tyrosine and tryptophan biosynthesis	ath:AT1G15710	prephenate dehydrogenase family protein
ath00400	Phenylalanine, tyrosine and tryptophan biosynthesis	ath:AT5G54810	TSB1; tryptophan synthase beta-subunit 1
ath00710	Carbon fixation in photosynthetic organisms	ath:AT2G01290	RPI2; ribose-5-phosphate isomerase 2
ath00710	Carbon fixation in photosynthetic organisms	ath:AT2G21330	FBA1; fructose-bisphosphate aldolase 1
ath00710	Carbon fixation in photosynthetic organisms	ath:ArthCp030	rbcL; ribulose-1,5-bisphosphate carboxylase/oxygenase large subunit
ath00520	Amino sugar and nucleotide sugar metabolism	ath:AT1G26570	UGD1; UDP-glucose dehydrogenase 1
ath00520	Amino sugar and nucleotide sugar metabolism	ath:AT3G54420	EP3; homolog of carrot EP3-3 chitinase

KEGG Pathway	Pathway Name	KEGG Gene ID	Gene Name
ath00520	Amino sugar and nucleotide sugar metabolism	ath:AT5G48300	ADG1; ADP glucose pyrophosphorylase 1
ath00040	Pentose and glucuronate interconversions	ath:AT1G26570	UGD1; UDP-glucose dehydrogenase 1
ath00040	Pentose and glucuronate interconversions	ath:AT3G07010	Pectin lyase-like superfamily protein
ath00040	Pentose and glucuronate interconversions	ath:AT3G14310	PME3; pectin methylesterase 3
ath00480	Glutathione metabolism	ath:AT2G24200	LAP1; Cytosol aminopeptidase family protein
ath00480	Glutathione metabolism	ath:AT4G30920	LAP2; Cytosol aminopeptidase family protein
ath00480	Glutathione metabolism	ath:AT5G41670	6-phosphogluconate dehydrogenase family protein
ath00195	Photosynthesis	ath:AT1G32550	FdC2; 2Fe-2S ferredoxin-like superfamily protein
ath00195	Photosynthesis	ath:ArthCp028	atpE; ATP synthase CF1 epsilon subunit
ath00195	Photosynthesis	ath:ArthCp029	atpB; ATP synthase CF1 beta subunit
ath01240	Biosynthesis of cofactors	ath:AT1G26570	UGD1; UDP-glucose dehydrogenase 1
ath01240	Biosynthesis of cofactors	ath:AT2G36880	MAT3; methionine adenosyltransferase 3
ath01240	Biosynthesis of cofactors	ath:AT4G01850	SAM-2; S-adenosylmethionine synthetase 2
ath00030	Pentose phosphate pathway	ath:AT2G01290	RPI2; ribose-5-phosphate isomerase 2
ath00030	Pentose phosphate pathway	ath:AT2G21330	FBA1; fructose-bisphosphate aldolase 1
ath00030	Pentose phosphate pathway	ath:AT5G41670	6-phosphogluconate dehydrogenase family protein
ath00220	Arginine biosynthesis	ath:AT4G08870	ARGAH2; Arginase/deacetylase superfamily protein
ath00220	Arginine biosynthesis	ath:AT4G17830	Peptidase M20/M25/M40 family protein
ath00260	Glycine, serine and threonine metabolism	ath:AT2G38400	AGT3; alanine:glyoxylate aminotransferase 3
ath00260	Glycine, serine and threonine metabolism	ath:AT5G54810	TSB1; tryptophan synthase beta-subunit 1
ath04712	Circadian rhythm	ath:AT1G01060	LHY; Homeodomain-like superfamily protein
ath04712	Circadian rhythm	ath:AT5G62430	CDF1; cycling DOF factor 1
ath00906	Carotenoid biosynthesis	ath:AT4G25700	BETA-OHASE_1; beta-hydroxylase 1
ath00906	Carotenoid biosynthesis	ath:AT5G45340	CYP707A3; cytochrome P450, family 707, subfamily A, polypeptide 3
ath00900	Terpenoid backbone biosynthesis	ath:AT1G76490	HMG1; hydroxy methylglutaryl CoA reductase 1
ath00900	Terpenoid backbone biosynthesis	ath:AT5G48230	ACAT2; acetoacetyl-CoA thiolase 2
ath00380	Tryptophan metabolism	ath:AT4G34880	Amidase family protein
ath00380	Tryptophan metabolism	ath:AT5G48230	ACAT2; acetoacetyl-CoA thiolase 2
ath00280	Valine, leucine and isoleucine degradation	ath:AT2G38400	AGT3; alanine:glyoxylate aminotransferase 3
ath00280	Valine, leucine and isoleucine degradation	ath:AT5G48230	ACAT2; acetoacetyl-CoA thiolase 2
ath03013	Nucleocytoplasmic transport	ath:AT1G10390	Nucleoporin autopeptidase
ath03013	Nucleocytoplasmic transport	ath:AT5G60390	GTP binding Elongation factor Tu family protein
ath00630	Glyoxylate and dicarboxylate metabolism	ath:AT5G48230	ACAT2; acetoacetyl-CoA thiolase 2
ath00630	Glyoxylate and dicarboxylate metabolism	ath:ArthCp030	rbcL; ribulose-1,5-bisphosphate carboxylase/oxygenase large subunit
ath00500	Starch and sucrose metabolism	ath:AT3G62750	BGLU8; beta glucosidase 8
ath00500	Starch and sucrose metabolism	ath:AT5G48300	ADG1; ADP glucose pyrophosphorylase 1
ath00360	Phenylalanine metabolism	ath:AT3G53260	PAL2; phenylalanine ammonia-lyase 2
ath00360	Phenylalanine metabolism	ath:AT4G34880	Amidase family protein
ath01212	Fatty acid metabolism	ath:AT3G45770	Polyketide synthase, enoylreductase family protein
ath01212	Fatty acid metabolism	ath:AT5G48230	ACAT2; acetoacetyl-CoA thiolase 2
ath01250	Biosynthesis of nucleotide sugars	ath:AT1G26570	UGD1; UDP-glucose dehydrogenase 1
ath01250	Biosynthesis of nucleotide sugars	ath:AT5G48300	ADG1; ADP glucose pyrophosphorylase 1
ath04144	Endocytosis	ath:AT5G02500	HSC70-1; heat shock cognate protein 70-1
ath04626	Plant-pathogen interaction	ath:AT4G36070	CPK18; calcium-dependent protein kinase 18

KEGG Pathway	Pathway Name	KEGG Gene ID	Gene Name
ath00908	Zeatin biosynthesis	ath:AT2G36750	UGT73C1; UDP-glucosyl transferase 73C1
ath00902	Monoterpenoid biosynthesis	ath:AT3G61220	SDR1; NAD(P)-binding Rossmann-fold superfamily protein
ath00053	Ascorbate and aldarate metabolism	ath:AT1G26570	UGD1; UDP-glucose dehydrogenase 1
ath00620	Pyruvate metabolism	ath:AT5G48230	ACAT2; acetoacetyl-CoA thiolase 2
ath00920	Sulfur metabolism	ath:AT4G21990	APR3; APS reductase 3
ath00051	Fructose and mannose metabolism	ath:AT2G21330	FBA1; fructose-bisphosphate aldolase 1
ath00650	Butanoate metabolism	ath:AT5G48230	ACAT2; acetoacetyl-CoA thiolase 2
ath04070	Phosphatidylinositol signaling system	ath:AT5G58700	PLC4; phosphatidylinositol-speciwc phospholipase C4
ath00904	Diterpenoid biosynthesis	ath:AT4G25420	GA20OX1; 2-oxoglutarate (2OG) and Fe(II)-dependent oxygenase superfamily protein
ath00010	Glycolysis / Gluconeogenesis	ath:AT2G21330	FBA1; fructose-bisphosphate aldolase 1
ath00960	Tropane, piperidine and pyridine alkaloid biosynthesis	ath:AT1G07440	NAD(P)-binding Rossmann-fold superfamily protein
ath00905	Brassinosteroid biosynthesis	ath:AT2G38050	DET2; 3-oxo-5-alpha-steroid 4-dehydrogenase family protein
ath00460	Cyanoamino acid metabolism	ath:AT3G62750	BGLU8; beta glucosidase 8
ath00310	Lysine degradation	ath:AT5G48230	ACAT2; acetoacetyl-CoA thiolase 2
ath00062	Fatty acid elongation	ath:AT3G45770	Polyketide synthase, enoylreductase family protein
ath00130	Ubiquinone and other terpenoid-quinone biosynthesis	ath:AT1G51680	4CL1; 4-coumarate:CoA ligase 1
ath00240	Pyrimidine metabolism	ath:AT3G25400	dCTP pyrophosphatase-like protein
ath00562	Inositol phosphate metabolism	ath:AT5G58700	PLC4; phosphatidylinositol-speciwc phospholipase C4
ath03040	Spliceosome	ath:AT5G02500	HSC70-1; heat shock cognate protein 70-1
ath04141	Protein processing in endoplasmic reticulum	ath:AT5G02500	HSC70-1; heat shock cognate protein 70-1
ath03018	RNA degradation	ath:AT3G44260	CAF1a; Polynucleotidyl transferase, ribonuclease H-like superfamily protein
ath00250	Alanine, aspartate and glutamate metabolism	ath:AT2G38400	AGT3; alanine:glyoxylate aminotransferase 3
ath00071	Fatty acid degradation	ath:AT5G48230	ACAT2; acetoacetyl-CoA thiolase 2
ath00196	Photosynthesis - antenna proteins	ath:AT1G29930	CAB1; chlorophyll A/B binding protein 1
ath00020	Citrate cycle (TCA cycle)	ath:AT1G60810	ACLA-2; ATP-citrate lyase A-2
ath00410	beta-Alanine metabolism	ath:AT5G13700	PAO1; polyamine oxidase 1
ath00061	Fatty acid biosynthesis	ath:AT3G45770	Polyketide synthase, enoylreductase family protein
ath00860	Porphyrin metabolism	ath:AT5G43860	CLH2; chlorophyllase 2
ath01210	2-Oxocarboxylic acid metabolism	ath:AT4G17830	Peptidase M20/M25/M40 family protein
