# Supplementary material for: Effects of ‘Candidatus Liberibacter solanacearum’ haplotypes A and B on tomato gene expression and geotropism
Source: BMC Plant Biol. 2022 Mar 30;22:156. doi: 10.1186/s12870-022-03505-z (PMC8966271; doi:10.1186/s12870-022-03505-z)
Supplement: Supplementary file 4 — Additional file 4: Table S4. Results from the g:Profiler analysis. The first column describes the GO information source (MF for molecular function, BP for ‘biological process’, and CC for ‘cellular component’ and KEGG for ‘Kyoto Encyclopedia of Genes and Genomes’) for each circle. The second column describes the term name associated with each circle. The third column describes the associated GO ID for the term. The fourth column shows the adjusted p-value for each term. Columns five through eight describe the term, query, intersection, and effective domain sizes. [file 12870_2022_3505_MOESM4_ESM.pdf]

Source	Term Name	Term ID	Adjusted p-value	Term Size	Query Size	Intersection Size	Effective Domain Size
GO:MF	caffeoyl-CoA O-methyltransferase activity	GO:0042409	<0.01	4	239	3	21188
GO:MF	transcription regulatory region sequence-specific DNA binding	GO:0000976	<0.01	960	239	27	21188
GO:MF	regulatory region nucleic acid binding	GO:0001067	<0.01	960	239	27	21188
GO:MF	sequence-specific double-stranded DNA binding	GO:1990837	<0.01	986	239	27	21188
GO:MF	oxidoreductase activity	GO:0016491	0.01	1685	239	38	21188
GO:MF	DNA-binding transcription factor activity	GO:0003700	0.02	1644	239	37	21188
GO:MF	metal ion binding	GO:0046872	0.03	3708	239	66	21188
GO:MF	cation binding	GO:0043169	0.03	3724	239	66	21188
GO:MF	double-stranded DNA binding	GO:0003690	0.05	1095	239	27	21188
GO:BP	response to stimulus	GO:0050896	<0.01	6807	242	136	23416
GO:BP	response to chemical	GO:0042221	<0.01	3407	242	90	23416
GO:BP	response to stress	GO:0006950	<0.01	3969	242	91	23416
GO:BP	response to oxygen-containing compound	GO:1901700	<0.01	1812	242	55	23416
GO:BP	response to endogenous stimulus	GO:0009719	<0.01	1877	242	55	23416
GO:BP	response to organic substance	GO:0010033	<0.01	2313	242	62	23416
GO:BP	response to hormone	GO:0009725	<0.01	1839	242	54	23416
GO:BP	response to abiotic stimulus	GO:0009628	<0.01	2372	242	62	23416
GO:BP	response to inorganic substance	GO:0010035	<0.01	1015	242	38	23416
GO:BP	cellular response to chemical stimulus	GO:0070887	<0.01	1793	242	46	23416
GO:BP	response to wounding	GO:0009611	<0.01	204	242	14	23416
GO:BP	response to water	GO:0009415	<0.01	406	242	19	23416
GO:BP	hormone-mediated signaling pathway	GO:0009755	<0.01	981	242	30	23416
GO:BP	cellular response to endogenous stimulus	GO:0071495	<0.01	1101	242	32	23416
GO:BP	metabolic process	GO:0008152	<0.01	13480	242	178	23416
GO:BP	response to acid chemical	GO:0001101	<0.01	437	242	19	23416
GO:BP	defense response	GO:0006952	<0.01	1603	242	40	23416
GO:BP	response to water deprivation	GO:0009414	<0.01	397	242	18	23416
GO:BP	cellular response to hormone stimulus	GO:0032870	<0.01	1062	242	31	23416
GO:BP	cellular process	GO:0009987	<0.01	16066	242	201	23416
GO:BP	response to jasmonic acid	GO:0009753	<0.01	247	242	14	23416
GO:BP	oxoacid metabolic process	GO:0043436	<0.01	1290	242	34	23416
GO:BP	organic acid metabolic process	GO:0006082	<0.01	1299	242	34	23416
GO:BP	lignin biosynthetic process	GO:0009809	<0.01	51	242	7	23416
GO:BP	cellular response to organic substance	GO:0071310	<0.01	1270	242	33	23416
GO:BP	response to osmotic stress	GO:0006970	<0.01	701	242	23	23416
GO:BP	response to ethylene	GO:0009723	<0.01	323	242	15	23416
GO:BP	response to chitin	GO:0010200	<0.01	139	242	10	23416
GO:BP	carboxylic acid metabolic process	GO:0019752	<0.01	1123	242	30	23416
GO:BP	oxidation-reduction process	GO:0055114	<0.01	1860	242	41	23416
GO:BP	response to cadmium ion	GO:0046686	<0.01	348	242	15	23416
GO:BP	regulation of jasmonic acid mediated signaling pathway	GO:2000022	<0.01	41	242	6	23416
GO:BP	small molecule metabolic process	GO:0044281	<0.01	1897	242	41	23416
GO:BP	regulation of response to stimulus	GO:0048583	<0.01	770	242	23	23416
GO:BP	response to biotic stimulus	GO:0009607	<0.01	1439	242	34	23416
GO:BP	organic substance metabolic process	GO:0071704	0.01	11945	242	157	23416

Source	Term Name	Term ID	Adjusted p-value	Term Size	Query Size	Intersection Size	Effective Domain Size
GO:BP	response to external stimulus	GO:0009605	0.01	1873	242	40	23416
GO:BP	phenylpropanoid biosynthetic process	GO:0009699	0.01	101	242	8	23416
GO:BP	defense response to insect	GO:0002213	0.01	29	242	5	23416
GO:BP	response to external biotic stimulus	GO:0043207	0.01	1426	242	33	23416
GO:BP	response to other organism	GO:0051707	0.01	1426	242	33	23416
GO:BP	lignin metabolic process	GO:0009808	0.01	75	242	7	23416
GO:BP	biological regulation	GO:0065007	0.01	7043	242	104	23416
GO:BP	response to metal ion	GO:0010038	0.01	492	242	17	23416
GO:BP	organic substance biosynthetic process	GO:1901576	0.01	5682	242	88	23416
GO:BP	regulation of signal transduction	GO:0009966	0.01	347	242	14	23416
GO:BP	secondary metabolic process	GO:0019748	0.02	398	242	15	23416
GO:BP	phenylpropanoid metabolic process	GO:0009698	0.02	144	242	9	23416
GO:BP	regulation of signaling	GO:0023051	0.02	352	242	14	23416
GO:BP	ion transport	GO:0006811	0.02	1075	242	27	23416
GO:BP	regulation of cell communication	GO:0010646	0.02	356	242	14	23416
GO:BP	biosynthetic process	GO:0009058	0.02	5820	242	89	23416
GO:BP	interspecies interaction between organisms	GO:0044419	0.02	1475	242	33	23416
GO:BP	carboxylic acid biosynthetic process	GO:0046394	0.03	580	242	18	23416
GO:BP	organic acid biosynthetic process	GO:0016053	0.03	580	242	18	23416
GO:BP	cellular response to stimulus	GO:0051716	0.04	3511	242	60	23416
GO:BP	response to lipid	GO:0033993	0.04	881	242	23	23416
GO:CC	cell periphery	GO:0071944	<0.01	4222	232	76	21213
GO:CC	external encapsulating structure	GO:0030312	<0.01	809	232	25	21213
GO:CC	cell wall	GO:0005618	<0.01	807	232	24	21213
GO:CC	extracellular region	GO:0005576	0.01	2008	232	41	21213
KEGG	Biosynthesis of secondary metabolites	KEGG:01110	<0.01	1230	72	38	4976
KEGG	Stilbenoid, diarylheptanoid and gingerol biosynthesis	KEGG:00945	<0.01	9	72	4	4976
KEGG	Metabolic pathways	KEGG:01100	<0.01	2266	72	49	4976
KEGG	Phenylpropanoid biosynthesis	KEGG:00940	0.01	177	72	10	4976
KEGG	Flavonoid biosynthesis	KEGG:00941	0.01	24	72	4	4976
