# Supplementary material for: Effects of ‘Candidatus Liberibacter solanacearum’ haplotypes A and B on tomato gene expression and geotropism
Source: BMC Plant Biol. 2022 Mar 30;22:156. doi: 10.1186/s12870-022-03505-z (PMC8966271; doi:10.1186/s12870-022-03505-z)
Supplement: Supplementary file 5 — Additional file 5: Table S5. The 139 tomato plant DEGs associated with plant defense response to abiotic and biotic stress under Lso infection. DEGs were sorted by differential expression (DE) values comparing uninfected (control and Lso-free) to infected (LsoA- and LsoB-infected) samples (p-value < 0.05). NCBI Blast searches were used to identify Gene IDs and protein products in tomatoes as well as their homologs in other species. Specifically, the expression changes in 127 of these genes (91%, in italics) would have resulted in increased responsiveness to or promoted defense against stressors in infected plants. These DEGs were predominantly related to defense against pathogens and response to drought, temperature, and salt stress. [file 12870_2022_3505_MOESM5_ESM.pdf]

TomatoID	DE in Infected	NCBI Protein Name	Gene ID	Uniprot Description	Putative Consequences for Infection	Citation
Solyc12g010030.2	8.64	leucine aminopeptidase 2, chloroplastic isoform X1	LAP2	Involved in the processing and regular turnover of intracellular proteins; Heat shock protein	*Increased turnover of intracellular proteins; Increased protection from heat-induced damaged*	Waditee-Sirisattha, Rungaroon, et al. "The Arabidopsis aminopeptidase LAP2 regulates plant growth, leaf longevity and stress response." New Phytologist 191.4 (2011): 958-969.
Solyc09g084470.3	8.38	wound-induced proteinase inhibitor 1 precursor	N/A	Inhibits both chymotrypsin and trypsin, supressing protein digestion by herbivores	*Increased defense against herbivory*	Cleveland, Thomas E., Robert W. Thornburg, and Clarence A. Ryan. "Molecular characterization of a wound-inducible inhibitor I gene from potato and the processing of its mRNA and protein." Plant molecular biology 8.3 (1987): 199-207.
Solyc09g084480.3	8.38	wound-induced proteinase inhibitor 1-like	N/A	Inhibits both chymotrypsin and trypsin, supressing protein digestion by herbivores	*Increased defense against herbivory*	Cleveland, Thomas E., Robert W. Thornburg, and Clarence A. Ryan. "Molecular characterization of a wound-inducible inhibitor I gene from potato and the processing of its mRNA and protein." Plant molecular biology 8.3 (1987): 199-207.
Solyc09g084490.3	8.38	wound-induced proteinase inhibitor 1-like	N/A	Inhibits both chymotrypsin and trypsin, supressing protein digestion by herbivores	*Increased defense against herbivory*	Cleveland, Thomas E., Robert W. Thornburg, and Clarence A. Ryan. "Molecular characterization of a wound-inducible inhibitor I gene from potato and the processing of its mRNA and protein." Plant molecular biology 8.3 (1987): 199-207.
Solyc01g095140.3	7.90	ethylene-responsive late embryogenesis-like protein	ER5	Involved in response to desiccation	*Increased response to drought*	Zegzouti, Hicham, et al. "ER5, a tomato cDNA encoding an ethylene-responsive LEA-like protein: characterization and expression in response to drought, ABA and wounding." Plant molecular biology 35.6 (1997): 847-854.
Solyc09g089540.3	7.71	wound-induced proteinase inhibitor 1-like	N/A	Inhibits both chymotrypsin and trypsin, supressing protein digestion by herbivores	*Increased defense against herbivory*	Cleveland, Thomas E., Robert W. Thornburg, and Clarence A. Ryan. "Molecular characterization of a wound-inducible inhibitor I gene from potato and the processing of its mRNA and protein." Plant molecular biology 8.3 (1987): 199-207.
Solyc03g098780.2	7.43	aspartic protease inhibitor 1	N/A	Inhibitor of cathepsin D; Protects the plant by inhibiting proteases of invading organisms	*Increased defense against herbivory*	Ishikawa, Atsushi, et al. "A family of potato genes that encode Kunitz-type proteinase inhibitors: structural comparisons and differential expression." Plant and cell physiology 35.2 (1994): 303-312.
Solyc09g083440.3	7.35	wound-induced proteinase inhibitor 1	N/A	Inhibits both chymotrypsin and trypsin, supressing protein digestion by herbivores	*Increased defense against herbivory*	Cleveland, Thomas E., Robert W. Thornburg, and Clarence A. Ryan. "Molecular characterization of a wound-inducible inhibitor I gene from potato and the processing of its mRNA and protein." Plant molecular biology 8.3 (1987): 199-207.
Solyc09g089520.3	6.60	proteinase inhibitor I-B-like	LOC107794480	Involved in response to wounding	*Promoted response to wounding*	Sierro, Nicolas, et al. "The tobacco genome sequence and its comparison with those of tomato and potato." Nature communications 5.1 (2014): 1-9.
Solyc09g089510.3	6.29	proteinase inhibitor I-B	LOC107794480	Involved in response to wounding	*Promoted response to wounding*	Sierro, Nicolas, et al. "The tobacco genome sequence and its comparison with those of tomato and potato." Nature communications 5.1 (2014): 1-9.
Solyc01g091170.3	6.10	Arginase 2, chloroplastic/mitochondrial	ARGAH2	Utilized in the urea cycle; Precursor for the synthesis of both polyamines and proline; Catalyzes the formation of putrescine from agmatine	*Promoted jasmonate-related functions; Increased urea waste management; Increased freezing tolerance*	Dombrecht, Bruno, et al. "MYC2 differentially modulates diverse jasmonate-dependent functions in Arabidopsis." The Plant Cell 19.7 (2007): 2225-2245.

TomatoID	DE in Infected	NCBI Protein Name	Gene ID	Uniprot Description	Putative Consequences for Infection	Citation
Solyc11g071480.1	5.55	agmatine hydroxycinnamoyltransferase 1-like	AHT1	Promotes the synthesis of hydroxycinnamic acid amides, which play a role in defense against pathogens	*Increased defense against pathogens*	Chen, Wei, et al. "Genome-wide association analyses provide genetic and biochemical insights into natural variation in rice metabolism." Nature genetics 46.7 (2014): 714-721.
Solyc12g009220.2	4.88	jasmonate ZIM-domain protein 2	NtJAZ2	Involved in regulation of defense response; Involved in regulation of JA-mediated signaling pathway; Involved in response to wounding	*Increased regulation of defense response; Increased regulation of JA-mediated signaling; Increased response to wounding*	Shoji, Tsubasa, Takayuki Ogawa, and Takashi Hashimoto. "Jasmonate-induced nicotine formation in tobacco is mediated by tobacco COI1 and JAZ genes." Plant and cell physiology 49.7 (2008): 1003-1012.
Solyc06g076020.3	4.79	heat shock cognate 70 kDa protein 1	HSP70-1	Facilitates folding of de novo proteins, assists translocation of precursor proteins into organelles; Degrades damaged protein under stress conditions; Involved in defense response; Involved in protein transport to chloroplasts; Modulates stomatal aperture in response to environmental conditions	*Increased protein folding; Increased translocation of presursor proteins into organelles; Increased degradation of damaged proteins; Promoted defense response; Increased response to abiotic stress and ABA signaling*	Sung, Dong Yul, and Charles L. Guy. "Physiological and molecular assessment of altered expression of Hsc70-1 in Arabidopsis. Evidence for pleiotropic consequences." Plant Physiology 132.2 (2003): 979-987.
Solyc01g060020.3	4.77	glucan endo-1,3-beta-glucosidase B precursor	N/A	Involved in plant defense against pathogens	*Increased defense against pathogens*	van Kan, Jan AL, et al. "Differential accumulation of mRNAs encoding extracellular and intracellular PR proteins in tomato induced by virulent and avirulent races of Cladosporium fulvum." Plant molecular biology 20.3 (1992): 513-527.
Solyc11g011030.2	4.51	Pto-responsive gene 1 protein	PTI1	A serine-threonine kinase involved in the hypersensitive response-mediated signaling cascade	*Promoted hypersensitive response-mediated signaling*	Zhou, Jianmin, et al. "The tomato gene Pti1 encodes a serine/threonine kinase that is phosphorylated by Pto and is involved in the hypersensitive response." Cell 83.6 (1995): 925-935.
Solyc04g079730.1	4.40	allene oxide synthase	CYP74A	Involved in the synthesis of JA	*Increased production of JA*	Laudert, Dietmar, et al. "Cloning, molecular and functional characterization of Arabidopsis thaliana allene oxide synthase (CYP 74), the first enzyme of the octadecanoid pathway to jasmonates." Plant molecular biology 31.2 (1996): 323-335.
Solyc02g090970.1	4.29	mitogen-activated protein kinase kinase kinase 17-like	MAPKKK17	Act as ABA signal transducer under abiotic stress	*Increased response to stress*	Danquah, Agyemang, et al. "Identification and characterization of an ABA-activated MAP kinase cascade in Arabidopsis thaliana." The Plant Journal 82.2 (2015): 232-244.
Solyc03g098730.1	4.26	kunitz trypsin inhibitor 2	KTI2	Involved in the control of cell death in the transmitting tract and septum epidermis during flower development; Plays a role in herbivore resistance during seedling greening	*Increased programmed cell death; Increased defense against herbivores*	Shoji, Tsubasa, Takayuki Ogawa, and Takashi Hashimoto. "Jasmonate-induced nicotine formation in tobacco is mediated by tobacco COI1 and JAZ genes." Plant and cell physiology 49.7 (2008): 1003-1012.
Solyc07g042170.3	4.24	protein TIFY 10b-like	AT1G74950	Repressor of jasmonate responses; Negatively regulates root hair development	Repressed jasmonate responses; Impaired root hair development	Fernández-Calvo, Patricia, et al. "The Arabidopsis bHLH transcription factors MYC3 and MYC4 are targets of JAZ repressors and act additively with MYC2 in the activation of jasmonate responses." The Plant Cell 23.2 (2011): 701-715.

TomatoID	DE in Infected	NCBI Protein Name	Gene ID	Uniprot Description	Putative Consequences for Infection	Citation
Solyc08g036640.3	4.11	protein TIFY 5A-like	TIFY5A	Repressor of jasmonate responses; Interacts with and suppresses RHD6 and RSL1 transcription factor activities to negatively regulate root hair development	Repressed jasmonate responses; Impaired root hair development	Chung, Hoo Sun, and Gregg A. Howe. "A critical role for the TIFY motif in repression of jasmonate signaling by a stabilized splice variant of the JASMONATE ZIM-domain protein JAZ10 in Arabidopsis." The Plant Cell 21.1 (2009): 131-145.
Solyc12g049400.2	4.10	protein TIFY 10b-like	AT1G74950	Repressor of jasmonate responses; Negatively regulates root hair development	Repressed jasmonate responses; Impaired root hair development	Fernández-Calvo, Patricia, et al. "The Arabidopsis bHLH transcription factors MYC3 and MYC4 are targets of JAZ repressors and act additively with MYC2 in the activation of jasmonate responses." The Plant Cell 23.2 (2011): 701-715.
Solyc01g007030.3	3.99	E3 ubiquitin-protein ligase PUB22-like	PUB22	Negatively regulates water stress response; Mediates drought signaling pathway; Negative regulator of immune response triggered by PAMPs	Impaired response to drought; Impaired response to PAMPs; Impaired attenuation to PAMP-induced signaling	Trujillo, Marco, et al. "Negative regulation of PAMP-triggered immunity by an E3 ubiquitin ligase triplet in Arabidopsis." Current Biology 18.18 (2008): 1396-1401.
Solyc12g019320.2	3.99	protein DETOXIFICATION 30-like	LOC107764941	Xenobiotic transmembrane transporter activity	*Increased detoxification*	Sierro, Nicolas, et al. "The tobacco genome sequence and its comparison with those of tomato and potato." Nature communications 5.1 (2014): 1-9.
Solyc07g041920.3	3.88	cysteine proteinase 3-like	CYS3	Involved in the regulation of endogenous processes; Involved in the regulation of defense against pests and pathogens	*Promoted regulation of endogenous processes; Increased regulation of defense*	Belenghi, Beatrice, et al. "AtCYS1, a cystatin from Arabidopsis thaliana, suppresses hypersensitive cell death." European Journal of Biochemistry 270.12 (2003): 2593-2604.
Solyc02g087210.3	3.79	stress-associated protein 11	SAP11	Involved in environmental stress response	*Increased response to environmental stress*	Vij, Shubha, and Akhilesh K. Tyagi. "Genome-wide analysis of the stress associated protein (SAP) gene family containing A20/AN1 zinc-finger (s) in rice and their phylogenetic relationship with Arabidopsis." Molecular Genetics and Genomics 276.6 (2006): 565-575.
Solyc11g018800.2	3.77	lignin-forming anionic peroxidase	N/A	Involved in removal of H2O2, oxidation of toxic reductants, biosynthesis and degradation of lignin, suberization, auxin catabolism, and response to biotic stressors	*Increased response to oxidative stress; Increased auxin catabolism; Increased defense against wounding and pathogen attack*	Lagrimini, L. Mark, et al. "Molecular cloning of complementary DNA encoding the lignin-forming peroxidase from tobacco: molecular analysis and tissue-specific expression." Proceedings of the National Academy of Sciences 84.21 (1987): 7542-7546.
Solyc08g036660.3	3.75	protein TIFY 5A-like	TIFY5A	Repressor of jasmonate responses; Interacts with and suppresses RHD6 and RSL1 transcription factor activities to negatively regulate root hair development	Repressed jasmonate responses; Impaired root hair development	Chung, Hoo Sun, and Gregg A. Howe. "A critical role for the TIFY motif in repression of jasmonate signaling by a stabilized splice variant of the JASMONATE ZIM-domain protein JAZ10 in Arabidopsis." The Plant Cell 21.1 (2009): 131-145.
Solyc08g007830.1	3.72	dehydration-responsive element-binding protein 1F-like	DREB1F	Transcriptional activator that binds specifically to the C-repeat/DRE element mediating cold-inducible transcription	*Increased cold acclimation and freezing tolerance*	Sakuma, Yoh, et al. "DNA-binding specificity of the ERF/AP2 domain of Arabidopsis DREBs, transcription factors involved in dehydration-and cold-inducible gene expression." Biochemical and biophysical research communications 290.3 (2002): 998-1009.

TomatoID	DE in Infected	NCBI Protein Name	Gene ID	Uniprot Description	Putative Consequences for Infection	Citation
Solyc11g071470.1	3.65	agmatine hydroxycinnamoyltransferase 1-like	AHT1	Promotes the synthesis of hydroxycinnamic acid amides, which play a role in defense against pathogens	*Increased defense against pathogens*	Chen, Wei, et al. "Genome-wide association analyses provide genetic and biochemical insights into natural variation in rice metabolism." Nature genetics 46.7 (2014): 714-721.
Solyc06g035700.1	3.64	ethylene-responsive transcription factor ERF025-like	ERF025	Binds to the GCC-box pathogenesis-related promoter element; Regulates gene expression under stress	*Increased response to stress and pathogenesis*	Riechmann, José Luis, et al. "Arabidopsis transcription factors: genome-wide comparative analysis among eukaryotes." Science 290.5499 (2000): 2105-2110.
Solyc01g007040.3	3.63	E3 ubiquitin-protein ligase PUB22-like	PUB22	Negatively regulates water stress response; Mediates drought signaling pathway; Negative regulator of immune response triggered by PAMPs	Impaired response to drought; Impaired response to PAMPs; Impaired attenuation to PAMP-induced signaling	Trujillo, Marco, et al. "Negative regulation of PAMP-triggered immunity by an E3 ubiquitin ligase triplet in Arabidopsis." Current Biology 18.18 (2008): 1396-1401.
Solyc12g057160.1	3.60	classical arabinogalactan protein 5	AGP5	Proteoglycan involved in differentiation, cell-cell recognition, embryogenesis, and programmed cell death	*Promoted plant growth/development and cell differentiation; Promoted programmed cell death*	Thieme, Christoph J., et al. "Endogenous Arabidopsis messenger RNAs transported to distant tissues." Nature Plants 1.4 (2015): 1-9.
Solyc04g082140.3	3.52	multicopper oxidase-like protein precursor	LPR1	Multicopper oxidase involved in Cu homeostasis and oxidative stress response; Necessary for root growth inhibition	*Promoted Cu homeostasis; Increased response to oxidative stress; Root growth inhibition*	Svistoonoff, Sergio, et al. "Root tip contact with low-phosphate media reprograms plant root architecture." Nature genetics 39.6 (2007): 792-796.
Solyc10g009270.3	3.50	transcription factor MYC2-like	MYC2	Involved in the regulation of ABA-inducible genes under drought stress; Negative regulator of light-regulated gene expression and growth; Positive regulator of lateral root formation; Regulates sesquiterpene biosynthesis	*Promoted response to drought; Impaired response to light-mediated expression; Promoted lateral root formation; Increased regulation of sesquiterpene biosynthesis*	Abe, Hiroshi, et al. "Role of Arabidopsis MYC and MYB homologs in drought-and abscisic acid-regulated gene expression." The Plant Cell 9.10 (1997): 1859-1868.
Solyc06g083130.3	3.44	dCTP pyrophosphatase 1	DCTPP1	Hydrolyzes dNTPs to the corresponding nucleoside monophosphates; Protects DNA/RNA against the incorporation of these genotoxic nucleotide analogs through their catabolism	*Increased DNA/RNA protection against genotoxic nucleotide analogs*	Cheng, Chia-Yi, et al. "Araport11: a complete reannotation of the Arabidopsis thaliana reference genome." The Plant Journal 89.4 (2017): 789-804.
Solyc01g108240.3	3.44	ethylene-responsive transcription factor ERF109	ERF109	Binds to the GCC-box pathogenesis-related promoter element; Regulates gene expression under stress	*Increased response to stress and pathogenesis*	Riechmann, José Luis, et al. "Arabidopsis transcription factors: genome-wide comparative analysis among eukaryotes." Science 290.5499 (2000): 2105-2110.
Solyc03g122340.3	3.29	lipoxygenase	N/A	Involved in growth and development, pest resistance, and senescence in response to wounding	*Promoted plant growth/development, pest resistance, and senescence*	Chechetkin, I. R., et al. "Specificity of oxidation of linoleic acid homologs by plant lipoxygenases." Biochemistry (Moscow) 74.8 (2009): 855-861.

TomatoID	DE in Infected	NCBI Protein Name	Gene ID	Uniprot Description	Putative Consequences for Infection	Citation
Solyc10g050970.1	3.26	ethylene-responsive transcription factor ERF109-like	ERF109	Regulates gene expression under stress	*Increased response to stress and pathogenesis*	Riechmann, José Luis, et al. "Arabidopsis transcription factors: genome-wide comparative analysis among eukaryotes." Science 290.5499 (2000): 2105-2110.
Solyc03g124110.2	3.14	dehydration-responsive element-binding protein 1A	DREB1A	Transcriptional activator that binds specifically to the C-repeat/DRE element mediating cold-inducible transcription	*Increased cold acclimation and freezing tolerance*	Alonso-Blanco, Carlos, et al. "Genetic and molecular analyses of natural variation indicate CBF2 as a candidate gene for underlying a freezing tolerance quantitative trait locus in Arabidopsis." Plant Physiology 139.3 (2005): 1304-1312.
Solyc04g063210.3	3.14	probable caffeoyl-CoA O-methyltransferase At4g26220 isoform X2	AT4G26220	Plays a role in the synthesis of feruloylated polysaccharides; Involved in the reinforcement of the plant cell wall; Involved in response to wounding and/or pathogen challenge	*Increased reinforcement of plant cell walls; Promoted response to wounding and/or pathogen challenge*	Meyermans, Hugo, et al. "Modifications in lignin and accumulation of phenolic glucosides in poplar xylem upon down-regulation of caffeoyl-coenzyme A O-methyltransferase, an enzyme involved in lignin biosynthesis." Journal of Biological Chemistry 275.47 (2000): 36899-36909.
Solyc08g068710.1	3.12	tyramine N-feruloyltransferase 4/11-like	THT4	Synthesizes amides which are involved in stress response in the cell wall	*Promoted stress response in the plant cell wall*	Farmer, Mary Jo, et al. "Identification and characterization of cDNA clones encoding hydroxycinnamoyl-CoA: tyramine N-hydroxycinnamoyltransferase from tobacco." European journal of biochemistry 263.3 (1999): 686-694.
Solyc01g005870.2	3.11	receptor-like protein Cf-9	CF-9	Involved in plant defense; Confers resistance to the fungal pathogen	*Promoted defense against certain fungal pathogens*	van der Hoorn, Renier AL, et al. "Structure–function analysis of cf-9, a receptor-like protein with extracytoplasmic leucine-rich repeats." The Plant Cell 17.3 (2005): 1000-1015.
Solyc01g105650.3	3.10	protein DMR6-LIKE OXYGENASE 2-like	DLO2	Component of a negative feedback regulation system of SA levels; Negative regulator of defense against certain pathogens	Negative system regulation of SA; Down regulaton of defense against certain pathogens	Zeilmaker, Tieme, et al. "DOWNY MILDEW RESISTANT 6 and DMR 6-LIKE OXYGENASE 1 are partially redundant but distinct suppressors of immunity in Arabidopsis." The Plant Journal 81.2 (2015): 210-222.
Solyc12g009240.1	3.09	ethylene-responsive transcription factor ERF017	ERF017	Regulates gene expression under stress	*Increased response to stress and pathogenesis*	Riechmann, José Luis, et al. "Arabidopsis transcription factors: genome-wide comparative analysis among eukaryotes." Science 290.5499 (2000): 2105-2110.
Solyc07g006890.1	3.08	cytochrome P450 94A1-like	CYP94A1	Catalyzes the omega-hydroxylation of various fatty acids; Plays a minor role in cutin synthesis and plant defense	*Increased cutin synthesis and plant defense*	TIJET, Nathalie, et al. "Functional expression in yeast and characterization of a clofibrate-inducible plant cytochrome P-450 (CYP94A1) involved in cutin monomers synthesis." Biochemical Journal 332.2 (1998): 583-589.
Solyc12g005450.1	3.07	probable receptor-like protein kinase At4g10390	AT4G10390	Involved in protein phosphorylation; Involved in response to wounding	*Increased protein phosphorylation; Promoted response to wounding*	Peng, Mingsheng, et al. "Genome-wide analysis of Arabidopsis responsive transcriptome to nitrogen limitation and its regulation by the ubiquitin ligase gene NLA." Plant molecular biology 65.6 (2007): 775-797.
Solyc07g048060.2	3.06	cytochrome b561 and DOMON domain-containing protein At5g48750	AT5G48750	Involved in oxidation-reduction process	*Improved oxidation-reduction*	Kotani, Hirokazu, et al. "Structural analysis of Arabidopsis thaliana chromosome 5. VI. Sequence features of the regions of 1,367,185 bp covered by 19 physically assigned P1 and TAC clones." DNA Research 5.3 (1998): 203-216.

TomatoID	DE in Infected	NCBI Protein Name	Gene ID	Uniprot Description	Putative Consequences for Infection	Citation
Solyc01g097520.3	3.03	annexin D4-like	ANN4	Involved in osmotic stress response and ABA signaling	*Increased osmostic stress and ABA signaling*	Lee, Sumin, et al. "Proteomic identification of annexins, calcium-dependent membrane binding proteins that mediate osmotic stress and abscisic acid signal transduction in Arabidopsis." The Plant Cell 16.6 (2004): 1378-1391.
Solyc03g111290.1	3.03	cytochrome P450 94B3-like	CYP94B3	Hydroxylase involved in the oxidation of the plant hormone JA-Ile; Exerts negative feedback control on JA-Ile levels and plays a key role in attenuation of jasmonate responses	*Promoted attenuation to JA-Ile and response to jasmonate*	Kitaoka, Naoki, et al. "Arabidopsis CYP94B3 encodes jasmonyl-L-isoleucine 12-hydroxylase, a key enzyme in the oxidative catabolism of jasmonate." Plant and Cell Physiology 52.10 (2011): 1757-1765.
Solyc03g119980.3	3.03	caffeoylshikimate esterase	CSE	Involved in the biosynthesis of lignin; Promotes the degradation of lysophosphatidylcholine and detoxifies the peroxidized membrane in response to cadmium-induced oxidative stress	*Increased biosynthesis of lignin; Increased detoxification of peroxidized membrane; Increased response to oxidative stress*	Gao, Wei, et al. "Acyl-CoA-binding protein 2 binds lysophospholipase 2 and lysoPC to promote tolerance to cadmium-induced oxidative stress in transgenic Arabidopsis." The Plant Journal 62.6 (2010): 989-1003.
Solyc07g007760.3	2.99	defensin-like protein precursor	FST	Involved in floral organogenesis; Protects reproductive organs from potential pathogen attack	*Promoted floral organogenesis; Increased defense against pathogens in reproductive organs*	Gu, Qing, et al. "A flower-specific cDNA encoding a novel thionin in tobacco." Molecular and general genetics MGG 234.1 (1992): 89-96.
Solyc01g087590.3	2.98	polyamine oxidase 1	PAO1	Plays an important role in the regulation of polyamine intracellular concentration; Involved in the production of hydrogen peroxide in response to salt and cold stresses	*Increased polyamine intracellular concentration; Involved in the production of hydrogen peroxide in response to salt and cold stress*	Tavladoraki, Paraskevi, et al. "Heterologous expression and biochemical characterization of a polyamine oxidase from Arabidopsis involved in polyamine back conversion." Plant Physiology 141.4 (2006): 1519-1532.
Solyc02g085730.3	2.98	allene oxide cyclase isoform 1, chloroplastic	AOC1	Involved in the production of 12-oxo-phytodienoic acid, a precursor of JA	*Increased production of JA precursor*	Zhai, Qingzhe, et al. "Phytochrome chromophore deficiency leads to overproduction of jasmonic acid and elevated expression of jasmonate-responsive genes in Arabidopsis." Plant and Cell Physiology 48.7 (2007): 1061-1071.
Solyc01g007020.3	2.92	E3 ubiquitin-protein ligase PUB22-like	PUB22	Negatively regulates water stress response; Mediates drought signaling pathway; Negative regulator of immune response triggered by PAMPs	Impaired response to drought; Impaired response to PAMPs; Impaired attenuation to PAMP-induced signaling	Trujillo, Marco, et al. "Negative regulation of PAMP-triggered immunity by an E3 ubiquitin ligase triplet in Arabidopsis." Current Biology 18.18 (2008): 1396-1401.
Solyc04g082200.2	2.88	dehydrin	dhn	Involved in drought response	*Promoted drought response*	Sander, I., et al. "Multiple wheat flour allergens and cross-reactive carbohydrate determinants bind IgE in baker's asthma." Allergy 66.9 (2011): 1208-1215.
Solyc07g063410.3	2.88	NAC domain-containing protein JA2L	JA2L	Transcription factor that acts in the jasmonate-mediated response to infection; Involved in jasmonate- and coronatine-mediated stomatal reopening in response to infection	*Promoted jasmonate- and coronatine-mediated response to infection*	Du, Minmin, et al. "MYC2 orchestrates a hierarchical transcriptional cascade that regulates jasmonate-mediated plant immunity in tomato." The Plant Cell 29.8 (2017): 1883-1906.

TomatoID	DE in Infected	NCBI Protein Name	Gene ID	Uniprot Description	Putative Consequences for Infection	Citation
Solyc10g076240.2	2.86	cationic peroxidase 1	PNC1	Involved in removal of H2O2, oxidation of toxic reductants, biosynthesis/degradation of lignin, suberization, auxin catabolism, and response to environmental stressesors	*Promoted response to oxidative stress and other environmental stressors; Increased lignin and auxin metabolism*	Buffard, Dominique, et al. "Molecular cloning of complementary DNAs encoding two cationic peroxidases from cultivated peanut cells." Proceedings of the National Academy of Sciences 87.22 (1990): 8874-8878.
Solyc03g093360.3	2.81	PLAT domain-containing protein 3	PLAT3	Involved in response to abiotic stress	*Promoted response to abiotic stress*	Moskal, William A., et al. "Experimental validation of novel genes predicted in the un-annotated regions of the Arabidopsis genome." BMC genomics 8.1 (2007): 18.
Solyc09g066360.1	2.80	ethylene-response factor C3	ERF.C.3	Acts downstream of MYC2 in the jasmonate-mediated response to infection	*Increased ethylene-responsive signaling; Promoted jasmonate-mediated defense against pathogens*	Du, Minmin, et al. "MYC2 orchestrates a hierarchical transcriptional cascade that regulates jasmonate-mediated plant immunity in tomato." The Plant Cell 29.8 (2017): 1883-1906.
Solyc04g071770.3	2.79	ethylene-responsive transcription factor ABR1-like	ABR1	Negative regulator of the ABA signaling pathway involved in seed germination and in response to stressors	*Increased ABA signaling; Increased response to stress and pathogenesis*	Pandey, Girdhar K., et al. "ABR1, an APETALA2-domain transcription factor that functions as a repressor of ABA response in Arabidopsis." Plant Physiology 139.3 (2005): 1185-1193.
Solyc10g054440.2	2.78	arginine decarboxylase 1	ADC1	Catalyzes the first step of polyamine biosynthesis to produce putrescine from arginine; Controls polyamine homeostasis which is crucial for normal plant growth and development	*Increased freezing tolerance; Increased seed production; Promoted growth/development*	Hanfrey, Colin, et al. "Arabidopsis polyamine biosynthesis: absence of ornithine decarboxylase and the mechanism of arginine decarboxylase activity." The Plant Journal 27.6 (2001): 551-560.
Solyc03g093610.1	2.77	ethylene response factor A.2	ERF2	Involved in defense response; Involved in intracellular signal transduction; Involved in lipid metabolism	*Increased defense response; Increased intracellular signal transduction; Increased lipid metabolism*	Zhang, Zhijin, et al. "Transcriptional regulation of the ethylene response factor LeERF2 in the expression of ethylene biosynthesis genes controls ethylene production in tomato and tobacco." Plant Physiology 150.1 (2009): 365-377.
Solyc04g079360.1	2.77	transcription factor MYB44-like	MYB44	Represses the expression of protein phosphatases 2C in response to ABA; Auxin-responsive; Promotes SA-mediated defense, but represses JA-mediated defense	*Increases response to ABA; Increased response to auxin signaling; Increases SA-mediated defense; Decreased JA-mediated defense*	Riechmann, José Luis, et al. "Arabidopsis transcription factors: genome-wide comparative analysis among eukaryotes." Science 290.5499 (2000): 2105-2110.
Solyc12g005640.2	2.74	transcription factor MYB14-like	MYB14	Transcription activator that regulates freezing tolerance by affecting expression of CBF genes	*Promoted regulation of freezing tolerance*	Chen, Yan, et al. "AtMYB14 regulates cold tolerance in Arabidopsis." Plant molecular biology reporter 31.1 (2013): 87-97.
Solyc08g008280.3	2.73	probable WRKY transcription factor 53	WRKY53	Transcription factor regulating the early events of leaf senescence; Negatively regulates the expression of ESR/ESP; Promotes resistance to certain pathogens by enhancing SA- dependent genes; Contributes to the suppression of PDF1.2	*Down regulation of ESR/ESP; Promoted resistance to certain pathogens by enhancing SA- dependent genes; Suppression of PDF1.2*	Miao, Ying, and Ulrike Zentgraf. "The antagonist function of Arabidopsis WRKY53 and ESR/ESP in leaf senescence is modulated by the jasmonic and salicylic acid equilibrium." The Plant Cell 19.3 (2007): 819-830.

TomatoID	DE in Infected	NCBI Protein Name	Gene ID	Uniprot Description	Putative Consequences for Infection	Citation
Solyc04g054990.3	2.71	PLAT domain-containing protein 2	PLAT2	Involved in response to abiotic stress	*Promoted response to abiotic stress*	Giacomelli, Lisa, Andrea Rudella, and Klaas Jan van Wijk. "High light response of the thylakoid proteome in Arabidopsis wild type and the ascorbate-deficient mutant vtc2-2. A comparative proteomics study." Plant Physiology 141.2 (2006): 685-701.
Solyc09g089500.3	2.71	proteinase inhibitor I-B-like isoform X2	LOC107794480	Involved in response to wounding	*Promoted response to wounding*	Sierro, Nicolas, et al. "The tobacco genome sequence and its comparison with those of tomato and potato." Nature communications 5.1 (2014): 1-9.
Solyc03g080190.3	2.70	protein DMR6-like oxygenase	DLO1	Component of negative feedback regulation of SA during senescence; Negative regulator of defense against certain pathogens	*Negative system regulation of SA; Promoted leaf senescence; Down regulaton of defense against certain pathogens*	Zeilmaker, Tieme, et al. "DOWNY MILDEW RESISTANT 6 and DMR 6-LIKE OXYGENASE 1 are partially redundant but distinct suppressors of immunity in Arabidopsis." The Plant Journal 81.2 (2015): 210-222.
Solyc07g042230.1	2.69	ethylene-responsive transcription factor ERF018-like	ERF018	Binds to the GCC-box pathogenesis-related promoter element; Regulates gene expression under stress	*Increased response to stress and pathogenesis*	Riechmann, José Luis, et al. "Arabidopsis transcription factors: genome-wide comparative analysis among eukaryotes." Science 290.5499 (2000): 2105-2110.
Solyc04g009440.3	2.68	NAC domain protein	NAC002	Involved in cellular response to hypoxia; Involved in regulation of ABA-activated signaling pathway; Involved in response to wounding	*Increased cellular response to hypoxia; Increased in regulation of ABA-activated signaling pathway; Increased in response to wounding*	"Molecular characterization of Brassica napus NAC domain transcriptional activators induced in response to biotic and abiotic stress."
Solyc06g074420.1	2.67	cytochrome P450 94C1	CYP94C1	Hydroxylase involved in the oxidation of JA-Ile; Exerts negative feedback control on JA-Ile levels and plays a key role in attenuation to jasmonate	*Promoted attenuation to JA-Ile and response to jasmonate*	Kandel, Sylvie, et al. "Characterization of a methyl jasmonate and wounding-responsive cytochrome P450 of Arabidopsis thaliana catalyzing dicarboxylic fatty acid formation in vitro." The FEBS journal 274.19 (2007): 5116-5127.
Solyc03g122190.3	2.66	salt responsive protein 1	SlSRG1	Involved in regulation of defense; Involved in regulation of JA-mediated signaling pathway	*Promoted regulation of defense; Promoted regulation of JA-mediated signaling pathway*	Ouyang, Bo, et al. "Identification of early salt stress response genes in tomato root by suppression subtractive hybridization and microarray analysis." Journal of experimental botany 58.3 (2007): 507-520.
Solyc10g081570.2	2.65	ABC transporter F family member 4	ABCF4	Involved in drought and pathogen resistance	*Increased resistance to drought and pathogens*	Kaundal, Amita, et al. "GENERAL CONTROL NONREPRESSIBLE4 degrades 14-3-3 and the RIN4 complex to regulate stomatal aperture with implications on nonhost disease resistance and drought tolerance." The Plant Cell 29.9 (2017): 2233-2248.
Solyc08g007840.2	2.64	ethylene-responsive transcription factor ERF027-like	ERF027	Binds to the GCC-box pathogenesis-related promoter element; Regulates gene expression under stress	*Increased response to stress and pathogenesis*	Riechmann, José Luis, et al. "Arabidopsis transcription factors: genome-wide comparative analysis among eukaryotes." Science 290.5499 (2000): 2105-2110.
Solyc03g026270.3	2.62	dehydration-responsive element-binding protein 1A	DREB1A	Transcriptional activator that binds specifically to the C-repeat/DRE element mediating cold-inducible transcription	*Increased cold acclimation and freezing tolerance*	Sakuma, Yoh, et al. "DNA-binding specificity of the ERF/AP2 domain of Arabidopsis DREBs, transcription factors involved in dehydration-and cold-inducible gene expression." Biochemical and biophysical research communications 290.3 (2002): 998-1009.

TomatoID	DE in Infected	NCBI Protein Name	Gene ID	Uniprot Description	Putative Consequences for Infection	Citation
Solyc12g099000.2	2.62	S-adenosylmethionine synthase 2	SAM2	Involved in SA-mediated defense	*Increased S-adenosylmethionine production; Promoted early defense response*	Rajjou, Loïc, et al. "Proteomic investigation of the effect of salicylic acid on Arabidopsis seed germination and establishment of early defense mechanisms." Plant physiology 141.3 (2006): 910-923.
Solyc09g006010.2	2.57	pathogenesis-related leaf protein 4	N/A	Involved in defense response against pathogens	*Promoted defense against pathogens*	van Kan, Jan AL, et al. "Differential accumulation of mRNAs encoding extracellular and intracellular PR proteins in tomato induced by virulent and avirulent races of Cladosporium fulvum." Plant molecular biology 20.3 (1992): 513-527.
Solyc10g083970.1	2.57	S-adenosylmethionine synthase 3-like	METK3	Catalyzes the formation of S-adenosylmethionine from methionine; Involved in SA-mediated defense; Involved in the biosynthesis of lignin	*Increased S-adenosylmethionine production; Promoted early defense response; Increased lignin biosynthesis*	Goto, Derek B., et al. "A single-nucleotide mutation in a gene encoding S-adenosylmethionine synthetase is associated with methionine over-accumulation phenotype in Arabidopsis thaliana." Genes & genetic systems 77.2 (2002): 89-95.
Solyc12g008350.2	2.57	dehydration responsive element binding protein	DREB	Transcriptional activator that binds specifically to the C-repeat/DRE element mediating cold-inducible transcription	*Increased cold acclimation and freezing tolerance*	Alonso-Blanco, Carlos, et al. "Genetic and molecular analyses of natural variation indicate CBF2 as a candidate gene for underlying a freezing tolerance quantitative trait locus in Arabidopsis." Plant Physiology 139.3 (2005): 1304-1312.
Solyc12g057150.1	2.55	classical arabinogalactan protein 5	AGP5	Proteoglycan involved in differentiation, cell-cell recognition, embryogenesis, and programmed cell death	*Promoted plant growth/development and cell differentiation; Promoted programmed cell death*	Thieme, Christoph J., et al. "Endogenous Arabidopsis messenger RNAs transported to distant tissues." Nature Plants 1.4 (2015): 1-9.
Solyc02g064980.1	2.53	mitogen-activated protein kinase kinase kinase 18-like	MAPKKK18	Act as ABA signal transducer under abiotic stress; Promotes stomatal growth/development; Inhibits germination and root growth; Promotes leaf senescence	*Increased response to stress; Increased reproductive development; Decreased growth/development; Increased leaf senescence*	Mitula, Filip, et al. "Arabidopsis ABA-activated kinase MAPKKK18 is regulated by protein phosphatase 2C ABI1 and the ubiquitin–proteasome pathway." Plant and Cell Physiology 56.12 (2015): 2351-2367.
Solyc10g086690.2	2.53	phosphatidylinositol:ceramide inositolphosphotransferase 2	IPCS2	Essential for sphingolipid biosynthesis; Plays an important role in modulating plant programmed cell death	*Increased sphingolipid biosynthesis; Promoted programmed cell death*	Wang, Wenming, et al. "An inositolphosphorylceramide synthase is involved in regulation of plant programmed cell death associated with defense in Arabidopsis." The Plant Cell 20.11 (2008): 3163-3179.
Solyc05g008370.1	2.52	probable ribose-5-phosphate isomerase 2	RPI2	Involved in programmed cell death; Involved in vegetative-to-reproductive phase transition in meristems	*Increased primary metabolism; Promoted programmed cell death; Promoted transition from vegetative-to-reproductive phase*	Xiong, Yuqing, et al. "Deficiency in a cytosolic ribose-5-phosphate isomerase causes chloroplast dysfunction, late flowering and premature cell death in Arabidopsis." Physiologia plantarum 137.3 (2009): 249-263.
Solyc06g068650.3	2.50	4-coumarate--CoA ligase	4CL1	Produces CoA thioesters of a variety of hydroxy- and methoxy-substituted cinnamic acids	*Increased production of phenylproanoid-derived compounds; Increased JA biosynthesis*	Ehlting, Jürgen, Jane JK Shin, and Carl J. Douglas. "Identification of 4-coumarate: coenzyme A ligase (4CL) substrate recognition domains." The Plant Journal 27.5 (2001): 455-465.
Solyc08g062330.3	2.49	ankyrin repeat-containing protein ITN1-like	ITN1	Involved in salt stress tolerance; Acts through ABA signaling pathways and promotes ROS production	*Increased salt stress tolerance; Increased ROS production*	Sakamoto, Hikaru, Osamu Matsuda, and Koh Iba. "ITN1, a novel gene encoding an ankyrin-repeat protein that affects the ABA-mediated production of reactive oxygen species and is involved in salt-stress tolerance in Arabidopsis thaliana." The Plant Journal 56.3 (2008): 411-422.

TomatoID	DE in Infected	NCBI Protein Name	Gene ID	Uniprot Description	Putative Consequences for Infection	Citation
Solyc12g097000.2	2.46	TMV resistance protein N-like	N	Disease resistance protein; Triggers plant defense systems, including the hypersensitive response	*Increased hypersensitive response*	Dinesh-Kumar, S. P., and Barbara J. Baker. "Alternatively spliced N resistance gene transcripts: their possible role in tobacco mosaic virus resistance." Proceedings of the National Academy of Sciences 97.4 (2000): 1908-1913.
Solyc07g062700.3	2.43	sodium/calcium exchanger NCL	NCL	Participates in the maintenance of calcium homeostasis; Plays a role in auxin response, diurnal rhythm, and flowering time; Involved in salt stress response	*Promoted calcium homeostasis; Increased response to auxin; Promoted circadian rhythm; Promoted salt stress response*	Wang, Peng, et al. "A Na+/Ca2+ exchanger-like protein (AtNCL) involved in salt stress in Arabidopsis." Journal of Biological Chemistry 287.53 (2012): 44062-44070.
Solyc08g068770.2	2.43	N-hydroxycinnamoyl-CoA:tyramine N-hydroxycinnamoyl transferase THT1-3	THT1-3	Involved in the production of p-Coumaroylnoradrenaline	*Increased defense against pathogens*	von Roepenack-Lahaye, Edda, et al. "p-Coumaroylnoradrenaline, a novel plant metabolite implicated in tomato defense against pathogens." Journal of Biological Chemistry 278.44 (2003): 43373-43383.
Solyc02g089900.1	2.42	lysM domain receptor-like kinase 4	LYK4	Lysin motif receptor kinase that functions as a cell surface receptor in chitin elicitor signaling leading to innate immunity against certain fungal and bacterial pathogens	*Promoted innate immunity; Increased resistance to certain fungal and bacterial pathogens*	Wan, Jinrong, et al. "LYK4, a lysin motif receptor-like kinase, is important for chitin signaling and plant innate immunity in Arabidopsis." Plant physiology 160.1 (2012): 396-406.
Solyc02g093250.3	2.42	caffeoyl-CoA O-methyltransferase-like	CCOAOMT1	Involved in the reinforcement of the plant cell wall; Involved in response to wounding or pathogen challenge by promoting the formation of cell wall-bound ferulic acid polymers	*Increased production of polysaccharides; Reinforced cell wall; Increased response to wounding and pathogens*	Do, Cao-Trung, et al. "Both caffeoyl Coenzyme A 3-O-methyltransferase 1 and caffeic acid O-methyltransferase 1 are involved in redundant functions for lignin, flavonoids and sinapoyl malate biosynthesis in Arabidopsis." Planta 226.5 (2007): 1117-1129.
Solyc01g096430.3	2.41	NAD(P)H:quinone oxidoreductase	NQR	Serves as a quinone reductase in connection with conjugation reactions of hydroquinones involved in detoxification	*Increased detoxification*	Sparla, Francesca, et al. "Cloning and heterologous expression of NAD(P)H: quinone reductase of Arabidopsis thaliana, a functional homologue of animal DT-diaphorase." FEBS letters 463.3 (1999): 382-386.
Solyc09g007260.3	2.37	ethylene-responsive transcription factor RAP2-7-like	RAP2-7	Regulates gene expression under stress; Negatively regulates flowering	*Increased response to stress and pathogenesis; Flowering delay*	Riechmann, José Luis, et al. "Arabidopsis transcription factors: genome-wide comparative analysis among eukaryotes." Science 290.5499 (2000): 2105-2110.
Solyc03g117870.3	2.36	4-coumarate--CoA ligase	4CL1	Produces CoA thioesters of a variety of hydroxy- and methoxy-substituted cinnamic acids	*Increased production of phenylproanoid-derived compounds; Increased JA biosynthesis*	Ehlting, Jürgen, Jane JK Shin, and Carl J. Douglas. "Identification of 4-coumarate: coenzyme A ligase (4CL) substrate recognition domains." The Plant Journal 27.5 (2001): 455-465.
Solyc07g006480.3	2.33	probably inactive leucine-rich repeat receptor-like protein kinase At5g48380	AT5G48380	Negative regulator of defense response to bacteria; Negative regulator of programmed cell death	Impaired defense response to bacteria; Impaired programmed cell death	Gao, Minghui, et al. "Regulation of cell death and innate immunity by two receptor-like kinases in Arabidopsis." Cell host & microbe 6.1 (2009): 34-44.

TomatoID	DE in Infected	NCBI Protein Name	Gene ID	Uniprot Description	Putative Consequences for Infection	Citation
Solyc04g071890.3	2.30	peroxidase 12 precursor	PER12	Involved in removal of H2O2, oxidation of toxic reductants, biosynthesis and degradation of lignin, suberization, auxin catabolism, and response to environmental stressors	*Increased removal of H2O2, oxidation of toxic reductants, biosynthesis and degradation of lignin, suberization, auxin catabolism, and response to environmental stressors*	Paynel, Florence, et al. "Temporal regulation of cell-wall pectin methylesterase and peroxidase isoforms in cadmium-treated flax hypocotyl." Annals of botany 104.7 (2009): 1363-1372.
Solyc01g096320.3	2.29	homeobox-leucine zipper protein ATHB-12	ATHB-12	Transcription activator that acts as a growth regulator in response to water deficit	*Increased regulation of growth/development; Increased response to drought*	Olsson, Anna, Peter Engström, and Eva Söderman. "The homeobox genes ATHB12 and ATHB7encode potential regulators of growth in response to water deficit in Arabidopsis." Plant molecular biology 55.5 (2004): 663-677.
Solyc02g093270.3	2.28	caffeoyl-CoA O-methyltransferase-like	CCOAOMT1	Involved in the reinforcement of the plant cell wall; Involved in response to wounding or pathogen challenge by promoting the formation of cell wall-bound ferulic acid polymers	*Increased production of polysaccharides; Reinforced cell wall; Increased response to wounding and pathogens*	Do, Cao-Trung, et al. "Both caffeoyl Coenzyme A 3-O-methyltransferase 1 and caffeic acid O-methyltransferase 1 are involved in redundant functions for lignin, flavonoids and sinapoyl malate biosynthesis in Arabidopsis." Planta 226.5 (2007): 1117-1129.
Solyc07g049370.2	2.26	glucan endo-1,3-beta-glucosidase 12	AT4G29360	Involved in carbohydrate metabolic process, cell wall organization, and plant defense response	*Increased carbohydrate metabolism, cell wall organization, and plant defense response*	Wu, Qiong, et al. "Long-term balancing selection contributes to adaptation in Arabidopsis and its relatives." Genome biology 18.1 (2017): 1-15.
Solyc09g064820.1	2.26	EID1-like F-box protein 3	EDL3	Involved in the following processes: ABA-activated signaling pathway, regulation of seed germination, response to osmotic stress, response to salt stress, response to water deprivation, and meristem phase transition	*Increased ABA-activated signaling; Increased regulation of seed germination; Promoted response to osmotic, salt, and water stress; Promoted transition from vegetative to reproductive phase in the meristem*	Friso, Giulia, et al. "In-depth analysis of the thylakoid membrane proteome of Arabidopsis thaliana chloroplasts: new proteins, new functions, and a plastid proteome database." The Plant Cell 16.2 (2004): 478-499.
Solyc02g069110.3	2.24	cathepsin B-like protease 2	CATHB2	Thiol protease that plays a central role in plant programmed cell death; Required for full levels of PCD during resistance gene-mediated hypersensitive response	*Increased initiation of programmed cell death; Promoted hypersensitive response*	Theologis, Athanasios, et al. "Sequence and analysis of chromosome 1 of the plant Arabidopsis thaliana." Nature 408.6814 (2000): 816-820.
Solyc06g060690.2	2.22	non-functional pseudokinase ZED1 isoform X1	ZED1	Involved in the regulation of the ambient temperature-sensitive intersection of growth and immune response	Repressed regulation of temperature-sensitive intersection of growth and immune response	Lewis, Jennifer D., et al. "The Arabidopsis ZED1 pseudokinase is required for ZAR1-mediated immunity induced by the Pseudomonas syringae type III effector HopZ1a." Proceedings of the National Academy of Sciences 110.46 (2013): 18722-18727.
Solyc01g006320.3	2.20	protein NDR1-like	NRD1	Required for disease resistance conferred by R genes recognizing bacterial and oomycete pathogens; Required for the establishment of hypersensitive response and SAR	*Promoted diseased resistance against bacterial and fungal pathogens; Promoted hypersensitive response; Promoted SAR*	Century, Karen S., Eric B. Holub, and Brian J. Staskawicz. "NDR1, a locus of Arabidopsis thaliana that is required for disease resistance to both a bacterial and a fungal pathogen." Proceedings of the National Academy of Sciences 92.14 (1995): 6597-6601.
Solyc12g094520.2	2.15	4-coumarate--CoA ligase-like 5	4CLL5	Contributes to JA biosynthesis by initiating the beta-oxidative chain shortening of its precursors	*Increased production of phenylproanoid-derived compounds; Increased JA biosynthesis*	Koo, Abraham JK, et al. "Identification of a peroxisomal acyl-activating enzyme involved in the biosynthesis of jasmonic acid in Arabidopsis." Journal of Biological Chemistry 281.44 (2006): 33511-33520.

TomatoID	DE in Infected	NCBI Protein Name	Gene ID	Uniprot Description	Putative Consequences for Infection	Citation
Solyc02g082930.3	2.14	acidic 27 kDa endochitinase precursor	CHI17	Involved in defense against chitin-containing fungal pathogens	*Increased defense against fungal pathogens*	Danhash, Nadia, et al. "Molecular characterization of four chitinase cDNAs obtained fromCladosporium fulvum-infected tomato." Plant molecular biology 22.6 (1993): 1017-1029.
Solyc07g052510.4	2.13	peroxidase 3 precursor	PER3	Involved in removal of H2O2, oxidation of toxic reductants, biosynthesis and degradation of lignin, suberization, auxin catabolism, response to environmental stressors	*Increased removal of H2O2, oxidation of toxic reductants, biosynthesis and degradation of lignin, suberization, auxin catabolism, and response to environmental stressors*	Paynel, Florence, et al. "Temporal regulation of cell-wall pectin methylesterase and peroxidase isoforms in cadmium-treated flax hypocotyl." Annals of botany 104.7 (2009): 1363-1372.
Solyc07g064820.1	2.13	mitogen-activated protein kinase kinase kinase 18-like	MAPKKK18	Act as ABA signal transducer under abiotic stress; Promotes stomatal growth/development; Inhibits germination and root growth; Promotes leaf senescence	*Increased response to stress; Increased reproductive development; Decreased growth/development; Increased leaf senescence*	Mitula, Filip, et al. "Arabidopsis ABA-activated kinase MAPKKK18 is regulated by protein phosphatase 2C ABI1 and the ubiquitin–proteasome pathway." Plant and Cell Physiology 56.12 (2015): 2351-2367.
Solyc12g014420.2	2.12	glucan endo-1,3-beta-glucosidase 13-like	AT5G56590	Involved in carbohydrate metabolic process, cell wall organization, and plant defense response	*Increased carbohydrate metabolism, cell wall organization, and plant defense response*	Wu, Qiong, et al. "Long-term balancing selection contributes to adaptation in Arabidopsis and its relatives." Genome biology 18.1 (2017): 1-15.
Solyc11g010390.1	2.11	classical arabinogalactan protein 10-like	AGP10	Proteoglycan involved in differentiation, cell-cell recognition, embryogenesis, and programmed cell death	*Promoted plant growth/development and cell differentiation; Promoted programmed cell death*	Thieme, Christoph J., et al. "Endogenous Arabidopsis messenger RNAs transported to distant tissues." Nature Plants 1.4 (2015): 1-9.
Solyc01g099370.3	2.10	protein SRC2 homolog	SRC2	Acts as an activator of RBOHF, which mediates reactive oxygene species production; Plays a role in cold responses	*Increased response to reactive oxygen species; Promoted response to cold*	Kawarazaki, Tomoko, et al. "A low temperature-inducible protein AtSRC2 enhances the ROS-producing activity of NADPH oxidase AtRbohF." Biochimica et Biophysica Acta (BBA)-Molecular Cell Research 1833.12 (2013): 2775-2780.
Solyc07g054220.1	2.10	ethylene-responsive transcription factor ERF054	ERF054	Binds to the GCC-box pathogenesis-related promoter element; Regulates gene expression under stress	*Increased response to stress and pathogenesis*	Riechmann, José Luis, et al. "Arabidopsis transcription factors: genome-wide comparative analysis among eukaryotes." Science 290.5499 (2000): 2105-2110.
Solyc10g055780.1	2.10	endochitinase 4	N/A	Involved in defense against chitin-containing fungal and bacterial pathogens	*Increased defense against chitin-containing pathogens*	Herget, Thomas, Jeff Schell, and Peter H. Schreier. "Elicitor-specific induction of one member of the chitinase gene family in Arachis hypogaea." Molecular and General Genetics MGG 224.3 (1990): 469-476.
Solyc01g079600.3	2.09	phospholipase A1 PLIP2, chloroplastic isoform X1	PLIP2	Catalyzes the initial step of oxylipins and jasmonate biosynthesis; Links ABA-mediated abiotic stress responses and oxylipin and jasmonate signaling pathways	*Increased jasmonate biosynthesis; Increased jasmonate signaling crosstalk*	Wang, Kun, et al. "Two abscisic acid-responsive plastid lipase genes involved in jasmonic acid biosynthesis in Arabidopsis thaliana." The Plant Cell 30.5 (2018): 1006-1022.
Solyc04g051360.3	2.09	ethylene-responsive transcription factor ABR1-like	ABR1	Negative regulator of the ABA signaling pathway involved in seed germination and in responses to stress	*Increased ABA signaling; Increased response to stress and pathogenesis*	Pandey, Girdhar K., et al. "ABR1, an APETALA2-domain transcription factor that functions as a repressor of ABA response in Arabidopsis." Plant Physiology 139.3 (2005): 1185-1193.

TomatoID	DE in Infected	NCBI Protein Name	Gene ID	Uniprot Description	Putative Consequences for Infection	Citation
Solyc07g007870.3	2.06	12-oxophytodienoate reductase 3	OPR3	Involved in the biosynthesis of JA and other oxylipin signaling moleclules; Required for the spatial and temporal regulation of JA levels during dehiscence of anthers	*Increased biosynthesis of JA and other oxylipin signaling molecules*	Sanders, Paul M., et al. "The Arabidopsis DELAYED DEHISCENCE1 gene encodes an enzyme in the jasmonic acid synthesis pathway." The Plant Cell 12.7 (2000): 1041-1061.
Solyc09g008280.2	2.04	S-adenosylmethionine synthase 3	METK3	Catalyzes the formation of S-adenosylmethionine from methionine; Involved in SA-mediated defense; Involved in the biosynthesis of lignin	*Increased S-adenosylmethionine production; Promoted early defense response; Increased lignin biosynthesis*	Goto, Derek B., et al. "A single-nucleotide mutation in a gene encoding S-adenosylmethionine synthetase is associated with methionine over-accumulation phenotype in Arabidopsis thaliana." Genes & genetic systems 77.2 (2002): 89-95.
Solyc09g089930.2	2.04	ethylene responsive element binding protein	EREBP1	Involved in defense response; Involved in ethylene-activated signaling pathway	*Increased defense response; Increased ethylene-responsive signaling*	Horvath, Diana M., Dorothy J. Huang, and Nam-Hai Chua. "Four classes of salicylate-induced tobacco genes." Molecular plant-microbe interactions 11.9 (1998): 895-905.
Solyc01g102390.3	2.03	germin-like protein 5-1	N/A	Plays a role in plant defense	*Increased plant defense*	Kawahara, Yoshihiro, et al. "Improvement of the Oryza sativa Nipponbare reference genome using next generation sequence and optical map data." Rice 6.1 (2013): 4.
Solyc07g008410.3	2.03	protein DETOXIFICATION 29-like	DTX29	Xenobiotic transmembrane transporter activity	*Increased detoxification*	Hanada, Kousuke, et al. "Functional compensation of primary and secondary metabolites by duplicate genes in Arabidopsis thaliana." Molecular biology and evolution 28.1 (2011): 377-382.
Solyc02g082430.3	2.02	MLO-like protein 8 isoform X2	MLO8	Involved in modulation of pathogen defense and leaf cell death	*Promoted programmed cell death; Increased defense against pathogens*	Menges, Margit, et al. "Cell cycle-regulated gene expression inarabidopsis." Journal of Biological Chemistry 277.44 (2002): 41987-42002.
Solyc03g112960.1	2.02	pectinesterase 1	PME1	Acts in the modification of cell walls; Acts as negative regulator of genes involved in salt stress response	*Increased modification of plant cell walls; Impaired salt stress response*	Creighton, Maria T., et al. "Methylation of protein phosphatase 2A—Influence of regulators and environmental stress factors." Plant, cell & environment 40.10 (2017): 2347-2358.
Solyc04g072000.3	2.01	endochitinase EP3	EP3	Involved in hypersensitive reaction against specific pathogens	*Increased hypersensitive reaction against some pathogens*	Liliane, B. de A., et al. "Arabidopsis thaliana class IV chitinase is early induced during the interaction with Xanthomonas campestris." Febs Letters 419.1 (1997): 69-75.
Solyc11g010500.1	2.01	mitochondrial uncoupling protein 5	PUMP5	Mitochondrial transporters that create proton leaks across the inner mitochondrial membrane; Involved in protecting plant cells against oxidative stress damage	*Increased defense against oxidative stress*	Borecký, Jiří, et al. "The plant energy-dissipating mitochondrial systems: depicting the genomic structure and the expression profiles of the gene families of uncoupling protein and alternative oxidase in monocots and dicots." Journal of Experimental Botany 57.4 (2006): 849-864.
Solyc01g102640.2	-2.00	nuclear pore complex protein NUP98A-like	NUP98A	Structural constituent of nuclear pore; Necessary for mRNA transport from the nucleus and protein import into the nucleus; Required for photoperiodism; Required for constitutive defense	*Impaired mRNA transport; Impaired photoperiodism; Impaired constitutive defense*	"A putative nucleoporin 96 is required for both basal defense and constitutive resistance responses mediated by suppressor of npr1-1,constitutive 1."
Solyc01g094210.2	-2.02	(+)-neomenthol dehydrogenase	SDR1	Involved in basal resistance against pathogens	*Impaired basal resistance against pathogens*	Choi, Hyong Woo, et al. "A role for a menthone reductase in resistance against microbial pathogens in plants." Plant physiology 148.1 (2008): 383-401.

TomatoID	DE in Infected	NCBI Protein Name	Gene ID	Uniprot Description	Putative Consequences for Infection	Citation
Solyc11g010270.2	-2.05	homeobox-leucine zipper protein ATHB-6	ATHB-6	Acts as a growth regulator in response to water deficit; Involved in the negative regulation of the ABA signaling pathway	*Impaired response to drought; Impaired regulation of ABA signaling*	Himmelbach, Axel, et al. "Homeodomain protein ATHB6 is a target of the protein phosphatase ABI1 and regulates hormone responses in Arabidopsis." The EMBO journal 21.12 (2002): 3029-3038.
Solyc06g008760.1	-2.06	glutaredoxin-C13	GRXC13	Involved in regulation of plant defense responses	Decreased regulation of plant defense	Brodersen, Peter, et al. "Arabidopsis MAP kinase 4 regulates salicylic acid-and jasmonic acid/ethylene-dependent responses via EDS1 and PAD4." The Plant Journal 47.4 (2006): 532-546.
Solyc10g051120.2	-2.06	mitochondrial pyruvate carrier 1-like	MPC1	Mediates the uptake of pyruvate into mitochondria	*Impaired drought response*	Li, Chun-Long, et al. "NRGA1, a putative mitochondrial pyruvate carrier, mediates ABA regulation of guard cell ion channels and drought stress responses in Arabidopsis." Molecular plant 7.10 (2014): 1508-1521.
Solyc03g025350.3	-2.08	silicon efflux transporter LSI2-like	LSI2	Silicon efflux transporter involved in silicon transport from the root cells to the apoplast	*Impaired cell wall response to abiotic and biotic stressors*	Ma, Jian Feng, et al. "An efflux transporter of silicon in rice." Nature 448.7150 (2007): 209-212.
Solyc07g014620.1	-2.11	auxin-responsive protein SAUR50	SAUR50	Effector of hormonal and environmental signals in plant growth	*Impaired response to hormonal and environmental signaling; Impaired growth/development*	Ren, Hong, and William M. Gray. "SAUR proteins as effectors of hormonal and environmental signals in plant growth." Molecular plant 8.8 (2015): 1153-1164.
Solyc07g056280.3	-2.11	WRKY transcription factor 30	WRKY30	Transcription factor involved in leaf senescence, response to hydrogen peroxide, response to ozone, and SA signaling pathway	*Impaired leaf senescence; Impaired response to hydrogen peroxide; Impaired reponse to ozone; Impaired resonse to SA signaling*	El-Esawi, Mohamed A., et al. "Overexpression of AtWRKY30 transcription factor enhances heat and drought stress tolerance in wheat (Triticum aestivum L.)." Genes 10.2 (2019): 163.
Solyc06g074800.1	-2.14	zinc finger protein ZAT5-like	ZAT5	Transcription factor involved in stress responses	*Impaired response to stressors*	Mittler, Ron, et al. "Gain-and loss-of-function mutations in Zat10 enhance the tolerance of plants to abiotic stress." FEBS letters 580.28-29 (2006): 6537-6542.
Solyc07g014680.3	-2.18	Na+ transporter HKT1,1	HKT1	Plays a central role in plant tolerance to salt; Involved in Na+ recirculation from shoots to roots by mediating Na+ loading into the phloem sap in shoots and unloading in roots	*Impaired salt tolerance*	Uozumi, Nobuyuki, et al. "The Arabidopsis HKT1 gene homolog mediates inward Na+ currents in Xenopus laevis oocytes and Na+ uptake in Saccharomyces cerevisiae." Plant physiology 122.4 (2000): 1249-1260.
Solyc08g075880.3	-2.32	heavy metal-associated isoprenylated plant protein 30-like	HIPP30	Involved in metal ion transport; Involved in response to drought stress	*Impaired metal ion transport; Impaired response to drought stress*	"Stress induced and nuclear localized HIPP26 from Arabidopsis thaliana interacts via its heavy metal associated domain with the drought stress related zinc finger transcription factor ATHB29."
Solyc07g005210.3	-2.33	temperature-induced lipocalin-1	TIL	Involved in basal and acquired thermotolerance; Lipocalin that confers protection against oxidative stress caused by heat, hypersalinity, freezing, paraquat, and light	*Impaired thermotolerance; Impaired tolerance to salt stress, freezing, paraquat, and light*	Abo-Ogiala, Atef, et al. "Temperature-induced lipocalin (TIL) is translocated under salt stress and protects chloroplasts from ion toxicity." Journal of plant physiology 171.3-4 (2014): 250-259.

TomatoID	DE in Infected	NCBI Protein Name	Gene ID	Uniprot Description	Putative Consequences for Infection	Citation
Solyc11g008530.2	-2.48	endoribonuclease Dicer 2d isoform X1	AT3G03300	Plays an essential role in transitive silencing of transgenes by processing secondary siRNAs; Plays a role in antiviral RNA silencing	*Impaired post-transcriptional gene silencing; Impaired defense against viral pathogens*	"DICER-LIKE2 plays a primary role in transitive silencing of transgenes in Arabidopsis."
Solyc04g011670.3	-2.64	TGACG-sequence-specific DNA-binding protein TGA-1A isoform X2	TGA1A	Promotes auxin- and SA-inducible transcription	*Impaired auxin- and SA-inducible transcription*	Pascuzzi, Pete, et al. "Auxin-induced stress potentiates trans-activation by a conserved plant basic/leucine-zipper factor." Journal of Biological Chemistry 273.41 (1998): 26631-26637.
Solyc11g069940.1	-2.87	glutaredoxin-C6	GRXC6	Involved in regulation of plant defense responses	*Impaired regulation of plant defense*	Brodersen, Peter, et al. "Arabidopsis MAP kinase 4 regulates salicylic acid-and jasmonic acid/ethylene-dependent responses via EDS1 and PAD4." The Plant Journal 47.4 (2006): 532-546.
Solyc09g008200.3	-2.95	heavy metal-associated isoprenylated plant protein 5	HIPP05	Heavy-metal-binding protein involved in metal ion tranpsport; Involved in disease resistance	*Impaired metal ion transport; Impaired disease resistance*	Cooper, Bret, et al. "A network of rice genes associated with stress response and seed development." Proceedings of the National Academy of Sciences 100.8 (2003): 4945-4950.
