# Supplementary material for: Effects of ‘Candidatus Liberibacter solanacearum’ haplotypes A and B on tomato gene expression and geotropism
Source: BMC Plant Biol. 2022 Mar 30;22:156. doi: 10.1186/s12870-022-03505-z (PMC8966271; doi:10.1186/s12870-022-03505-z)
Supplement: Supplementary file 6 — Additional file 6: Table S6. The 116 tomato plant DEGs associated with plant growth and development under Lso infection. DEGs were sorted by differential expression (DE) values comparing uninfected to infected samples (p-value < 0.05). NCBI Blast searches were used to identify Gene IDs and protein products in tomatoes as well as their homologs in other species. Specifically, the expression changes in 75 of these genes (65%, in italics) would have modified cell growth and development in infected plants. These DEGs were predominantly related to cell expansion and elongation, reproduction, increased cell wall modification, and pigment production. [file 12870_2022_3505_MOESM6_ESM.pdf]

TomatoID	DE in Infected	NCBI Protein Name	Gene ID	Uniprot Description	Putative Consequences for Infection	Citation
Solyc06g083900.3	6.03	transcription factor MYB13-like	MYB13	Component of a regulatory network controlling the establishment and/or development of the shoot system by the regulation of apical meristem function	*Increased establishment and/or development of the shoot system by the regulation of apical meristem function*	Kirik, Victor, et al. "Ectopic expression of a novel MYB gene modifies the architecture of the Arabidopsis inflorescence." The Plant Journal 13.6 (1998): 729-742.
Solyc06g066800.2	4.25	protein CDI	AT1G64980	Nucleotide-diphospho-sugar transferase required for pollen germination and tube growth	*Promoted pollen germination and tube growth*	Li, Hong-Mei, et al. "Cdi gene is required for pollen germination and tube growth in Arabidopsis." FEBS letters 586.7 (2012): 1027-1031.
Solyc02g083880.3	4.24	gibberellin-regulated protein 11	GASA11	Gibberellin-regulated protein that functions in hormonal controlled steps of reproductive development	*Promoted plant development*	Cheng, Chia-Yi, et al. "Araport11: a complete reannotation of the Arabidopsis thaliana reference genome." The Plant Journal 89.4 (2017): 789-804.
Solyc07g042170.3	4.24	protein TIFY 10b-like	AT1G74950	Repressor of jasmonate responses; Negatively regulates root hair development	Repressed jasmonate responses; Impaired root hair development	Fernández-Calvo, Patricia, et al. "The Arabidopsis bHLH transcription factors MYC3 and MYC4 are targets of JAZ repressors and act additively with MYC2 in the activation of jasmonate responses." The Plant Cell 23.2 (2011): 701-715.
Solyc04g074430.1	4.13	protein EXORDIUM-like	EXO	Required for cell expansion in leaves; Mediate brassinosteroid-induced leaf and root growth/development	*Promoted cell expansion in the leaves; Promoted leaf and root growth/development*	Coll-Garcia, Danahe, et al. "EXORDIUM regulates brassinosteroid-responsive genes." FEBS letters 563.1-3 (2004): 82-86.
Solyc08g036640.3	4.11	protein TIFY 5A-like	TIFY5A	Repressor of jasmonate responses; Interacts with and suppresses RHD6 and RSL1 transcription factor activities to negatively regulate root hair development	Repressed jasmonate responses; Impaired root hair development	Chung, Hoo Sun, and Gregg A. Howe. "A critical role for the TIFY motif in repression of jasmonate signaling by a stabilized splice variant of the JASMONATE ZIM-domain protein JAZ10 in Arabidopsis." The Plant Cell 21.1 (2009): 131-145.
Solyc12g049400.2	4.10	protein TIFY 10b-like	AT1G74950	Repressor of jasmonate responses; Negatively regulates root hair development	Repressed jasmonate responses; Impaired root hair development	Fernández-Calvo, Patricia, et al. "The Arabidopsis bHLH transcription factors MYC3 and MYC4 are targets of JAZ repressors and act additively with MYC2 in the activation of jasmonate responses." The Plant Cell 23.2 (2011): 701-715.
Solyc10g086500.1	3.78	steroid 5-alpha-reductase DET2	DET2	Involved in a reduction step in the biosynthesis of the plant steroid, brassinolide	*Increased brassinolide biosynthesis; Promoted cell elongation*	Fujioka, Shozo, et al. "The Arabidopsis deetiolated2 mutant is blocked early in brassinosteroid biosynthesis." The Plant Cell 9.11 (1997): 1951-1962.
Solyc11g018800.2	3.77	lignin-forming anionic peroxidase	N/A	Involved in removal of H2O2, oxidation of toxic reductants, biosynthesis and degradation of lignin, suberization, auxin catabolism, and response to biotic stressors	Increased response to oxidative stress; Increased auxin catabolism; Increased defense against wounding and pathogen attack	Lagrimini, L. Mark, et al. "Molecular cloning of complementary DNA encoding the lignin-forming peroxidase from tobacco: molecular analysis and tissue-specific expression." Proceedings of the National Academy of Sciences 84.21 (1987): 7542-7546.
Solyc08g036660.3	3.75	protein TIFY 5A-like	TIFY5A	Repressor of jasmonate responses; Interacts with and suppresses RHD6 and RSL1 transcription factor activities to negatively regulate root hair development	Repressed jasmonate responses; Impaired root hair development	Chung, Hoo Sun, and Gregg A. Howe. "A critical role for the TIFY motif in repression of jasmonate signaling by a stabilized splice variant of the JASMONATE ZIM-domain protein JAZ10 in Arabidopsis." The Plant Cell 21.1 (2009): 131-145.

TomatoID	DE in Infected	NCBI Protein Name	Gene ID	Uniprot Description	Putative Consequences for Infection	Citation
Solyc02g079590.3	3.62	receptor-like serine/threonine-protein kinase SD1-8	SD18	Involved in the regulation of cellular expansion and differentiation	*Increased regulation of cellular expansion and differentiation*	Ascencio-Ibáñez, José Trinidad, et al. "Global analysis of Arabidopsis gene expression uncovers a complex array of changes impacting pathogen response and cell cycle during geminivirus infection." Plant physiology 148.1 (2008): 436-454.
Solyc02g086300.3	3.60	putative glutamine amidotransferase GAT1_2.1	GAT1_2.1	Flutamine amidotransferase that represses shoot branching; Links nitrogen stress response and branching control	Impaired shoot branching; Stunting	Zhu, Huifen, and Robert G. Kranz. "A nitrogen-regulated glutamine amidotransferase (GAT1_2. 1) represses shoot branching in Arabidopsis." Plant physiology 160.4 (2012): 1770-1780.
Solyc12g057160.1	3.60	classical arabinogalactan protein 5	AGP5	Proteoglycan involved in differentiation, cell-cell recognition, embryogenesis, and programmed cell death	*Promoted plant growth/development and cell differentiation; Promoted programmed cell death*	Thieme, Christoph J., et al. "Endogenous Arabidopsis messenger RNAs transported to distant tissues." Nature Plants 1.4 (2015): 1-9.
Solyc04g074420.1	3.53	protein EXORDIUM	EXO	Required for cell expansion in leaves; Mediate brassinosteroid-induced leaf and root growth/development	*Promoted cell expansion in the leaves; Promoted leaf and root growth/development*	Coll-Garcia, Danahe, et al. "EXORDIUM regulates brassinosteroid-responsive genes." FEBS letters 563.1-3 (2004): 82-86.
Solyc06g082530.2	3.53	scarecrow-like protein 9	SCL9	Transcription factor involved in plant development	*Promoted plant development*	Menges, Margit, et al. "Cell cycle-regulated gene expression inarabidopsis." Journal of Biological Chemistry 277.44 (2002): 41987-42002.
Solyc04g082140.3	3.52	multicopper oxidase-like protein precursor	LPR1	Multicopper oxidase involved in Cu homeostasis and oxidative stress response; Necessary for root growth inhibition	Promoted Cu homeostasis; Increased response to oxidative stress; Root growth inhibition	Svistoonoff, Sergio, et al. "Root tip contact with low-phosphate media reprograms plant root architecture." Nature genetics 39.6 (2007): 792-796.
Solyc10g009270.3	3.50	transcription factor MYC2-like	MYC2	Involved in the regulation of ABA-inducible genes under drought stress; Negative regulator of light-regulated gene expression and growth; Positive regulator of lateral root formation; Regulates sesquiterpene biosynthesis	*Promoted response to drought; Impaired response to light-mediated expression; Promoted lateral root formation; Increased regulation of sesquiterpene biosynthesis*	Abe, Hiroshi, et al. "Role of Arabidopsis MYC and MYB homologs in drought-and abscisic acid-regulated gene expression." The Plant Cell 9.10 (1997): 1859-1868.
Solyc09g061840.3	3.40	acetyl-CoA C-acetyltranferase 5	N/A	Involved in plant growth and development	*Increased regulation of plant growth/development*	Jin, Huanan, Zhihong Song, and Basil J. Nikolau. "Reverse genetic characterization of two paralogous acetoacetyl CoA thiolase genes in Arabidopsis reveals their importance in plant growth and development." The Plant Journal 70.6 (2012): 1015-1032.
Solyc04g005040.1	3.34	matrix metalloproteinase 2 precursor	2MMP	Play a role in the degradation and remodeling of the extracellular matrix during development or in response to stressors; Required for plant growth, morphogenesis, and development	*Increased degradation and remodeling of the extracellular matrix; Promoted plant growth, morphogenesis, and development; Promoted flowering and senescence*	Golldack, Dortje, Olga V. Popova, and Karl-Josef Dietz. "Mutation of the matrix metalloproteinase At2-MMP inhibits growth and causes late flowering and early senescence in Arabidopsis." Journal of Biological Chemistry 277.7 (2002): 5541-5547.

TomatoID	DE in Infected	NCBI Protein Name	Gene ID	Uniprot Description	Putative Consequences for Infection	Citation
Solyc12g014430.2	3.30	cellulose synthase-like protein G3	CSLG3	Golgi-localized beta-glycan synthase that polymerize the backbones of noncellulosic polysaccharides of plant cell wall	*Promoted plant cell wall growth/development*	Lao, Jeemeng, et al. "The plant glycosyltransferase clone collection for functional genomics." The Plant Journal 79.3 (2014): 517-529.
Solyc03g122340.3	3.29	lipoxygenase	N/A	Involved in growth and development, pest resistance, and senescence in response to wounding	*Promoted plant growth/development, pest resistance, and senescence*	Chechetkin, I. R., et al. "Specificity of oxidation of linoleic acid homologs by plant lipoxygenases." Biochemistry (Moscow) 74.8 (2009): 855-861.
Solyc10g075110.2	3.29	non-specific lipid-transfer protein 1 precursor	LTP1	Play a role in wax or cutin deposition in the cell walls of expanding epidermal cells and certain secretory tissues	*Promoted cell wall expansion*	Skriver, Karen, et al. "Structure and expression of the barley lipid transfer protein gene Ltp1." Plant molecular biology 18.3 (1992): 585-589.
Solyc07g045350.3	3.27	Acetyl-CoA acetyltransferase, cytosolic 1	AAT1	Involved in plant growth and development	*Increased regulation of plant growth/development*	Jin, Huanan, Zhihong Song, and Basil J. Nikolau. "Reverse genetic characterization of two paralogous acetoacetyl CoA thiolase genes in Arabidopsis reveals their importance in plant growth and development." The Plant Journal 70.6 (2012): 1015-1032.
Solyc05g006220.3	3.18	IAA-amino acid hydrolase ILR1-like 2	ILL2	Hydrolyzes certain amino acid conjugates of the plant growth regulator indole-3-acetic acid; Acts with ILR1 to provide free IAA to germinating seedlings	*Promoted regulation of plant growth; Increased IAA supply to seedlings; Increased response to auxin*	LeClere, Sherry, et al. "Characterization of a family of IAA-amino acid conjugate hydrolases from Arabidopsis." Journal of Biological Chemistry 277.23 (2002): 20446-20452.
Solyc08g079090.3	3.16	monocopper oxidase-like protein SKU5	SKU5	Involved in directional growth processes, possibly by participating in cell wall expansion	*Promoted cell wall expansion; Promoted directional growth processes*	Shahollari, Bationa, et al. "A leucine-rich repeat protein is required for growth promotion and enhanced seed production mediated by the endophytic fungus Piriformospora indica in Arabidopsis thaliana." The Plant Journal 50.1 (2007): 1-13.
Solyc04g063210.3	3.14	probable caffeoyl-CoA O-methyltransferase At4g26220 isoform X2	AT4G26220	Plays a role in the synthesis of feruloylated polysaccharides; Involved in the reinforcement of the plant cell wall; Involved in response to wounding and/or pathogen challenge	*Increased reinforcement of plant cell walls; Promoted response to wounding and/or pathogen challenge*	Meyermans, Hugo, et al. "Modifications in lignin and accumulation of phenolic glucosides in poplar xylem upon down-regulation of caffeoyl-coenzyme A O-methyltransferase, an enzyme involved in lignin biosynthesis." Journal of Biological Chemistry 275.47 (2000): 36899-36909.
Solyc08g068710.1	3.12	tyramine N-feruloyltransferase 4/11-like	THT4	Synthesizes amides which are involved in stress response in the cell wall	*Promoted stress response in the plant cell wall*	Farmer, Mary Jo, et al. "Identification and characterization of cDNA clones encoding hydroxycinnamoyl-CoA: tyramine N-hydroxycinnamoyltransferase from tobacco." European journal of biochemistry 263.3 (1999): 686-694.
Solyc08g077020.1	3.04	auxin-responsive protein SAUR76	SAUR76	Involved in the regulation of ethylene receptor signaling; Promotes cell expansion and plant growth; Involved in the regulation of cell elongation	*Increased ethylene receptor signaling; Promoted cell expansion and plant growth/development; Leaf curl*	Markakis, Marios Nektarios, et al. "Characterization of a small auxin-up RNA (SAUR)-like gene involved in Arabidopsis thaliana development." PloS one 8.11 (2013): e82596.

TomatoID	DE in Infected	NCBI Protein Name	Gene ID	Uniprot Description	Putative Consequences for Infection	Citation
Solyc03g119980.3	3.03	caffeoylshikimate esterase	CSE	Involved in the biosynthesis of lignin; Promotes the degradation of lysophosphatidylcholine and detoxifies the peroxidized membrane in response to cadmium-induced oxidative stress	*Increased biosynthesis of lignin; Increased detoxification of peroxidized membrane; Increased response to oxidative stress*	Gao, Wei, et al. "Acyl-CoA-binding protein 2 binds lysophospholipase 2 and lysoPC to promote tolerance to cadmium-induced oxidative stress in transgenic Arabidopsis." The Plant Journal 62.6 (2010): 989-1003.
Solyc01g090180.3	3.02	4,5-DOPA dioxygenase extradiol	DODA	Opens the cyclic ring of dihydroxy-phenylalanine, producing betalamic acid	*Increased betalamic acid production; Increased pigment production*	Christinet, Laurent, et al. "Characterization and functional identification of a novel plant 4, 5-extradiol dioxygenase involved in betalain pigment biosynthesis in Portulaca grandiflora." Plant Physiology 134.1 (2004): 265-274.
Solyc03g093080.3	2.99	probable xyloglucan endotransglucosylase/hydrolase protein 23	XTH23	Cleaves and religates xyloglucan polymers, an essential constituent of the primary cell wall; Participates in cell wall construction of growing tissues	*Increased cell wall plant expansion; Increased response to auxin*	Ascencio-Ibáñez, José Trinidad, et al. "Global analysis of Arabidopsis gene expression uncovers a complex array of changes impacting pathogen response and cell cycle during geminivirus infection." Plant physiology 148.1 (2008): 436-454.
Solyc07g007760.3	2.99	defensin-like protein precursor	FST	Involved in floral organogenesis; Protects reproductive organs from potential pathogen attack	*Promoted floral organogenesis; Increased defense against pathogens in reproductive organs*	Gu, Qing, et al. "A flower-specific cDNA encoding a novel thionin in tobacco." Molecular and general genetics MGG 234.1 (1992): 89-96.
Solyc10g080690.2	2.96	patatin-like protein 3	PLP3	Possesses non-specific lipolytic acyl hydrolase activity; Plays a role in root development	*Promoted root development*	Rietz, Steffen, et al. "Roles of Arabidopsis patatin-related phospholipases a in root development are related to auxin responses and phosphate deficiency." Molecular Plant 3.3 (2010): 524-538.
Solyc02g090360.3	2.95	L-ascorbate oxidase homolog	N/A	Oxidoreductase involved in pollen tube growth	*Promoted pollen tube growth*	Weterings, Koen, et al. "Characterization of a pollen-specific cDNA clone from Nicotiana tabacum expressed during microgametogenesis and germination." Plant molecular biology 18.6 (1992): 1101-1111.
Solyc02g080200.3	2.93	pectinesterase	N/A	Acts in the modification of cell walls via demethylesterification of cell wall pectin	*Increased modification of plant cell walls*	N/A
Solyc02g080210.3	2.93	pectinesterase-like	N/A	Acts in the modification of cell walls via demethylesterification of cell wall pectin	*Increased modification of plant cell walls*	N/A
Solyc10g076240.2	2.86	cationic peroxidase 1	PNC1	Involved in removal of H2O2, oxidation of toxic reductants, biosynthesis/degradation of lignin, suberization, auxin catabolism, and response to environmental stressesors	*Promoted response to oxidative stress and other environmental stressors; Increased lignin and auxin metabolism*	Buffard, Dominique, et al. "Molecular cloning of complementary DNAs encoding two cationic peroxidases from cultivated peanut cells." Proceedings of the National Academy of Sciences 87.22 (1990): 8874-8878.
Solyc02g080120.2	2.82	gibberellin 2-beta-dioxygenase 8	GA2OX8	Catalyzes the 2-beta-hydroxylation of gibberellins precursors, rendering them unable to be converted to their active forms	Increased gibberellin regulation; Impaired growth/development	Schomburg, Fritz M., et al. "Overexpression of a novel class of gibberellin 2-oxidases decreases gibberellin levels and creates dwarf plants." The Plant Cell 15.1 (2003): 151-163.

TomatoID	DE in Infected	NCBI Protein Name	Gene ID	Uniprot Description	Putative Consequences for Infection	Citation
Solyc03g117600.3	2.81	shikimate O-hydroxycinnamoyltransferase	HST	Acyltransferase involved in the biosynthesis of lignin	*Increased biosynthesis of lignin*	Hoffmann, Laurent, et al. "Silencing of hydroxycinnamoyl-coenzyme A shikimate/quinate hydroxycinnamoyltransferase affects phenylpropanoid biosynthesis." The Plant Cell 16.6 (2004): 1446-1465.
Solyc01g057770.3	2.80	boron transporter 1 isoform X1	BOR1	Efflux-type boron transporter for xylem loading Boron is essential for maintaining the integrity of plants cell walls	*Promoted boron homeostasis; Promoted plant cell wall development and integrity*	Noguchi, Kyotaro, et al. "bor1-1, an Arabidopsis thaliana mutant that requires a high level of boron." Plant Physiology 115.3 (1997): 901-906.
Solyc10g054440.2	2.78	arginine decarboxylase 1	ADC1	Catalyzes the first step of polyamine biosynthesis to produce putrescine from arginine; Controls polyamine homeostasis which is crucial for normal plant growth and development	*Increased freezing tolerance; Increased seed production; Promoted growth/development*	Hanfrey, Colin, et al. "Arabidopsis polyamine biosynthesis: absence of ornithine decarboxylase and the mechanism of arginine decarboxylase activity." The Plant Journal 27.6 (2001): 551-560.
Solyc04g079360.1	2.77	transcription factor MYB44-like	MYB44	Represses the expression of protein phosphatases 2C in response to ABA; Auxin-responsive; Promotes SA-mediated defense, but represses JA-mediated defense	*Increases response to ABA; Increased response to auxin signaling; Increases SA-mediated defense; Decreased JA-mediated defense*	Riechmann, José Luis, et al. "Arabidopsis transcription factors: genome-wide comparative analysis among eukaryotes." Science 290.5499 (2000): 2105-2110.
Solyc10g075100.2	2.73	non-specific lipid transfer protein precursor	N/A	Plays a role in wax/cutin deposition in the cell walls of expanding epidermal cells and certain secretory tissues	*Promoted cell wall expansion*	Xie, Wan-Qin, et al. "The effects of calmodulin on the lipid-binding activity of CaM-binding protein-10 and maize non-specific lipid transfer protein." Zhi wu Sheng li yu fen zi Sheng wu xue xue bao= Journal of Plant Physiology and Molecular Biology 32.6 (2006): 679-684.
Solyc01g098910.3	2.71	peroxisomal adenine nucleotide carrier 1	PNC1	Required for the conversion of seed-reserved triacylglycerols into sucrose; Necessary for growth before the onset of photosynthesis	*Increased conversion of seed-reserved triacylglycerols; Promoted growth over storage*	Arai, Yuko, Makoto Hayashi, and Mikio Nishimura. "Proteomic identification and characterization of a novel peroxisomal adenine nucleotide transporter supplying ATP for fatty acid β-oxidation in soybean and Arabidopsis." The Plant Cell 20.12 (2008): 3227-3240.
Solyc12g011030.2	2.69	xyloglucan endotransglucosylase-hydrolase 9 precursor	XTH9	Essential constituent of the primary cell wall; Participates in cell wall construction of growing tissues	*Promoted plant cell wall elongation*	Hyodo, Hideki, et al. "Active gene expression of a xyloglucan endotransglucosylase/hydrolase gene, XTH9, in inflorescence apices is related to cell elongation in Arabidopsis thaliana." Plant molecular biology 52.2 (2003): 473-482.
Solyc05g009470.3	2.67	alpha-xylosidase 1	XYL1	Glycoside hydrolase releasing xylosyl residues from xyloglucan oligosaccharides; Essential for growth/development	*Promoted growth/development*	Sampedro, Javier, et al. "Cloning and expression pattern of a gene encoding an α-xylosidase active against xyloglucan oligosaccharides from Arabidopsis." Plant Physiology 126.2 (2001): 910-920.
Solyc09g018250.2	2.63	GBF-interacting protein 1-like isoform X1	GIP1	Contributes to bZIP-mediated gene regulation; Acts as negative co-regulator in red and blue light-mediated hypocotyl elongation	*Promoted gene regulation; Increased seed germination*	Sehnke, Paul C., et al. "Identification and characterization of GIP1, an Arabidopsis thaliana protein that enhances the DNA binding affinity and reduces the oligomeric state of G-box binding factors." Cell research 15.8 (2005): 567-575.

TomatoID	DE in Infected	NCBI Protein Name	Gene ID	Uniprot Description	Putative Consequences for Infection	Citation
Solyc07g005330.3	2.59	beta-glucosidase 08 precursor	BGLU8	Catalyzes hydrolysis of terminal, non-reducing beta-D-glucosyl residues with release of beta-D-glucose	*Increased cellulose production; Promoted plant growth/development*	Thieme, Christoph J., et al. "Endogenous Arabidopsis messenger RNAs transported to distant tissues." Nature Plants 1.4 (2015): 1-9.
Solyc09g062970.1	2.59	glycine-rich protein 5-like	GRP5	Involved in organ growth by promoting cell elongation processes	*Promoted cell elongation*	Mangeon, Amanda, et al. "AtGRP5, a vacuole-located glycine-rich protein involved in cell elongation." Planta 230.2 (2009): 253-265.
Solyc10g083970.1	2.57	S-adenosylmethionine synthase 3-like	METK3	Catalyzes the formation of S-adenosylmethionine from methionine; Involved in SA-mediated defense; Involved in the biosynthesis of lignin	*Increased S-adenosylmethionine production; Promoted early defense response; Increased lignin biosynthesis*	Goto, Derek B., et al. "A single-nucleotide mutation in a gene encoding S-adenosylmethionine synthetase is associated with methionine over-accumulation phenotype in Arabidopsis thaliana." Genes & genetic systems 77.2 (2002): 89-95.
Solyc12g057150.1	2.55	classical arabinogalactan protein 5	AGP5	Proteoglycan involved in differentiation, cell-cell recognition, embryogenesis, and programmed cell death	*Promoted plant growth/development and cell differentiation; Promoted programmed cell death*	Thieme, Christoph J., et al. "Endogenous Arabidopsis messenger RNAs transported to distant tissues." Nature Plants 1.4 (2015): 1-9.
Solyc02g064980.1	2.53	mitogen-activated protein kinase kinase kinase 18-like	MAPKKK18	Act as ABA signal transducer under abiotic stress; Promotes stomatal growth/development; Inhibits germination and root growth; Promotes leaf senescence	Increased response to stress; Increased reproductive development; Decreased growth/development; Increased leaf senescence	Mitula, Filip, et al. "Arabidopsis ABA-activated kinase MAPKKK18 is regulated by protein phosphatase 2C ABI1 and the ubiquitin–proteasome pathway." Plant and Cell Physiology 56.12 (2015): 2351-2367.
Solyc05g008370.1	2.52	probable ribose-5-phosphate isomerase 2	RPI2	Involved in programmed cell death; Involved in vegetative-to-reproductive phase transition in meristems	*Increased primary metabolism; Promoted programmed cell death; Promoted transition from vegetative-to-reproductive phase*	Xiong, Yuqing, et al. "Deficiency in a cytosolic ribose-5-phosphate isomerase causes chloroplast dysfunction, late flowering and premature cell death in Arabidopsis." Physiologia plantarum 137.3 (2009): 249-263.
Solyc04g016190.1	2.51	zeatin O-glucosyltransferase-like	ZOG1	Regulates cytokinin activity and storage; Impacts seed growth	*Increased cellular division and plant growth in roots and shoots; Increased reproductive investment*	Hou, Bingkai, et al. "N-glucosylation of cytokinins by glycosyltransferases of Arabidopsis thaliana." Journal of Biological Chemistry 279.46 (2004): 47822-47832.
Solyc10g079350.2	2.50	zeatin O-glucosyltransferase-like	ZOG1	Regulates cytokinin activity and storage; Impacts seed growth	*Increased cellular division and plant growth in roots and shoots; Increased reproductive investment*	Hou, Bingkai, et al. "N-glucosylation of cytokinins by glycosyltransferases of Arabidopsis thaliana." Journal of Biological Chemistry 279.46 (2004): 47822-47832.
Solyc04g074470.1	2.49	protein EXORDIUM-like 2	EXL2	Plays a role in a brassinosteroid-dependent regulation of growth/development	*Increased regulation of leaf and root growth/development*	Schröder, Florian, et al. "The extracellular EXO protein mediates cell expansion in Arabidopsis leaves." BMC plant biology 9.1 (2009): 20.
Solyc04g008330.1	2.45	zeatin O-glucosyltransferase-like	ZOG1	Regulates cytokinin activity and storage; Impacts seed growth	*Increased cellular division and plant growth in roots and shoots; Increased reproductive investment*	Hou, Bingkai, et al. "N-glucosylation of cytokinins by glycosyltransferases of Arabidopsis thaliana." Journal of Biological Chemistry 279.46 (2004): 47822-47832.
Solyc07g062700.3	2.43	sodium/calcium exchanger NCL	NCL	Participates in the maintenance of calcium homeostasis; Plays a role in auxin response, diurnal rhythm, and flowering time; Involved in salt stress response	*Promoted calcium homeostasis; Increased response to auxin; Promoted circadian rhythm; Promoted salt stress response*	Wang, Peng, et al. "A Na+/Ca2+ exchanger-like protein (AtNCL) involved in salt stress in Arabidopsis." Journal of Biological Chemistry 287.53 (2012): 44062-44070.

TomatoID	DE in Infected	NCBI Protein Name	Gene ID	Uniprot Description	Putative Consequences for Infection	Citation
Solyc08g080590.3	2.43	thaumatin-like protein	TLP1	Involved in local responses of roots to colonization by non-pathogenic plant growth-promoting rhizobacteria	*Increased colonization of growth-promoting rhizobacteria*	Leon-Kloosterziel, Karen M., et al. "Colonization of the Arabidopsis rhizosphere by fluorescent Pseudomonas spp. activates a root-specific, ethylene-responsive PR-5 gene in the vascular bundle." Plant molecular biology 57.5 (2005): 731-748.
Solyc12g099260.2	2.43	ATP-citrate synthase beta chain protein 2-like	ACLA-2	Used for the elongation of fatty acids and biosynthesis of isoprenoids, flavonoids, and malonated derivatives; Required for normal growth and development in seeds	*Promoted seed development; Increased production of primary metabolites*	Fatland, Beth L., Basil J. Nikolau, and Eve Syrkin Wurtele. "Reverse genetic characterization of cytosolic acetyl-CoA generation by ATP-citrate lyase in Arabidopsis." The Plant Cell 17.1 (2005): 182-203.
Solyc02g093250.3	2.42	caffeoyl-CoA O-methyltransferase-like	CCOAOMT1	Involved in the reinforcement of the plant cell wall; Involved in response to wounding or pathogen challenge by promoting the formation of cell wall-bound ferulic acid polymers	*Increased production of polysaccharides; Reinforced cell wall; Increased response to wounding and pathogens*	Do, Cao-Trung, et al. "Both caffeoyl Coenzyme A 3-O-methyltransferase 1 and caffeic acid O-methyltransferase 1 are involved in redundant functions for lignin, flavonoids and sinapoyl malate biosynthesis in Arabidopsis." Planta 226.5 (2007): 1117-1129.
Solyc03g007960.3	2.40	Beta-carotene 3-hydroxylase 1, chloroplastic	BETA-OHASE 1	Nonheme diiron monooxygenase involved in the biosynthesis of xanthophylls	*Increased biosynthesis of xanthophylls; Increased pigment production*	Sun, Zairen, Elisabeth Gantt, and Francis X. Cunningham. "Cloning and functional analysis of the β-carotene hydroxylase of Arabidopsis thaliana." Journal of Biological Chemistry 271.40 (1996): 24349-24352.
Solyc10g006700.1	2.39	calcium-binding protein PBP1	PBP1	Potential calcium sensor	Promoted calcium sensing; Promoted regulation of trichome morphogenesis	Reddy, Vaka S., et al. "KIC, a novel Ca2+ binding protein with one EF-hand motif, interacts with a microtubule motor protein and regulates trichome morphogenesis." The Plant Cell 16.1 (2004): 185-200.
Solyc09g007260.3	2.37	ethylene-responsive transcription factor RAP2-7-like	RAP2-7	Regulates gene expression under stress; Negatively regulates flowering	Increased response to stress and pathogenesis; Flowering delay	Riechmann, José Luis, et al. "Arabidopsis transcription factors: genome-wide comparative analysis among eukaryotes." Science 290.5499 (2000): 2105-2110.
Solyc01g079940.3	2.36	basic 7S globulin	BG	Seed storage protein; Protein kinase activity; Binds leginsulin	*Promoted seed development*	Kagawa, Hiroyuki, and Hisashi Hirano. "Sequence of a cDNA encoding soybean basic 7S globulin." Nucleic acids research 17.21 (1989): 8868.
Solyc08g076980.3	2.36	acetylornithine deacetylase-like	AT4G17830	Involved in flowering and fruit development	*Promoted flowering and fruit development*	Molesini, Barbara, et al. "Involvement of the putative N-acetylornithine deacetylase from Arabidopsis thaliana in flowering and fruit development." Plant and Cell Physiology 56.6 (2015): 1084-1096.
Solyc03g112880.1	2.33	fasciclin-like arabinogalactan protein 4	FLA4	Cell surface adhesion protein required for normal cell expansion	*Increased cell expansion*	Xu, Shou-Ling, et al. "Two leucine-rich repeat receptor kinases mediate signaling, linking cell wall biosynthesis and ACC synthase in Arabidopsis." The Plant Cell 20.11 (2008): 3065-3079.
Solyc07g043500.1	2.33	beta-D-glucosyl crocetin beta-1,6-glucosyltransferase	UGT94E5	Glucosyltransferase catalyzing crocetin gentiobiosyl esters biosynthesis	*Promoted production of crocetin and crocin; Increased pigment production*	Nagatoshi, Mai, et al. "UGT75L6 and UGT94E5 mediate sequential glucosylation of crocetin to crocin in Gardenia jasminoides." FEBS letters 586.7 (2012): 1055-1061.

TomatoID	DE in Infected	NCBI Protein Name	Gene ID	Uniprot Description	Putative Consequences for Infection	Citation
Solyc02g092790.3	2.31	Classical arabinogalactan protein 1	AGP1	Proteoglycan implicated in diverse developmental roles such as differentiation, cell-cell recognition, embryogenesis, and programmed cell death	*Promoted growth/development*	Vergnolle, Chantal, et al. "The cold-induced early activation of phospholipase C and D pathways determines the response of two distinct clusters of genes in Arabidopsis cell suspensions." Plant physiology 139.3 (2005): 1217-1233.
Solyc04g071890.3	2.30	peroxidase 12 precursor	PER12	Involved in removal of H2O2, oxidation of toxic reductants, biosynthesis and degradation of lignin, suberization, auxin catabolism, and response to environmental stressors	Increased removal of H2O2, oxidation of toxic reductants, biosynthesis and degradation of lignin, suberization, auxin catabolism, and response to environmental stressors	Paynel, Florence, et al. "Temporal regulation of cell-wall pectin methylesterase and peroxidase isoforms in cadmium-treated flax hypocotyl." Annals of botany 104.7 (2009): 1363-1372.
Solyc01g096320.3	2.29	homeobox-leucine zipper protein ATHB-12	ATHB-12	Transcription activator that acts as a growth regulator in response to water deficit	Increased regulation of growth/development; Increased response to drought	Olsson, Anna, Peter Engström, and Eva Söderman. "The homeobox genes ATHB12 and ATHB7encode potential regulators of growth in response to water deficit in Arabidopsis." Plant molecular biology 55.5 (2004): 663-677.
Solyc11g005150.2	2.29	leucine-rich repeat extensin-like protein 6	LRX6	Modulates cell morphogenesis by regulating cell wall formation and assembly, and/or growth polarization	*Promoted cell morphogenesis/regulation of cell wall formation and assembly, and/or growth polarization*	Cheng, Hui, et al. "Gibberellin acts through jasmonate to control the expression of MYB21, MYB24, and MYB57 to promote stamen filament growth in Arabidopsis." PLoS Genet 5.3 (2009): e1000440.
Solyc02g093270.3	2.28	caffeoyl-CoA O-methyltransferase-like	CCOAOMT1	Involved in the reinforcement of the plant cell wall; Involved in response to wounding or pathogen challenge by promoting the formation of cell wall-bound ferulic acid polymers	*Increased production of polysaccharides; Reinforced cell wall; Increased response to wounding and pathogens*	Do, Cao-Trung, et al. "Both caffeoyl Coenzyme A 3-O-methyltransferase 1 and caffeic acid O-methyltransferase 1 are involved in redundant functions for lignin, flavonoids and sinapoyl malate biosynthesis in Arabidopsis." Planta 226.5 (2007): 1117-1129.
Solyc02g088630.3	2.27	probable galacturonosyltransferase 14	GAUT14	Involved in pectin and/or xylans biosynthesis in cell walls	*Increased pectin and xylan biosynthesis; Increased cell wall expansion*	Wang, Li, et al. "Arabidopsis galacturonosyltransferase (GAUT) 13 and GAUT14 have redundant functions in pollen tube growth." Molecular plant 6.4 (2013): 1131-1148.
Solyc07g049370.2	2.26	glucan endo-1,3-beta-glucosidase 12	AT4G29360	Involved in carbohydrate metabolic process, cell wall organization, and plant defense response	*Increased carbohydrate metabolism, cell wall organization, and plant defense response*	Wu, Qiong, et al. "Long-term balancing selection contributes to adaptation in Arabidopsis and its relatives." Genome biology 18.1 (2017): 1-15.
Solyc09g064820.1	2.26	EID1-like F-box protein 3	EDL3	Involved in the following processes: ABA-activated signaling pathway, regulation of seed germination, response to osmotic stress, response to salt stress, response to water deprivation, and meristem phase transition	*Increased ABA-activated signaling; Increased regulation of seed germination; Promoted response to osmotic, salt, and water stress; Promoted transition from vegetative to reproductive phase in the meristem*	Friso, Giulia, et al. "In-depth analysis of the thylakoid membrane proteome of Arabidopsis thaliana chloroplasts: new proteins, new functions, and a plastid proteome database." The Plant Cell 16.2 (2004): 478-499.
Solyc06g060690.2	2.22	non-functional pseudokinase ZED1 isoform X1	ZED1	Involved in the regulation of the ambient temperature-sensitive intersection of growth and immune response	Repressed regulation of temperature-sensitive intersection of growth and immune response	Lewis, Jennifer D., et al. "The Arabidopsis ZED1 pseudokinase is required for ZAR1-mediated immunity induced by the Pseudomonas syringae type III effector HopZ1a." Proceedings of the National Academy of Sciences 110.46 (2013): 18722-18727.

TomatoID	DE in Infected	NCBI Protein Name	Gene ID	Uniprot Description	Putative Consequences for Infection	Citation
Solyc11g072600.2	2.19	AP2 transcription factor SlAP2d isoform X1	N/A	DNA-binding transcription factor; Involved in tomato fruit ripening	*Increased tomato ripening*	Karlova, Rumyana, et al. "Transcriptome and metabolite profiling show that APETALA2a is a major regulator of tomato fruit ripening." The Plant Cell 23.3 (2011): 923-941.
Solyc04g074440.1	2.16	protein EXORDIUM-like	EXO	Required for cell expansion in leaves; Mediate brassinosteroid-induced leaf and root growth/development	*Promoted cell expansion in the leaves; Promoted leaf and root growth/development*	Coll-Garcia, Danahe, et al. "EXORDIUM regulates brassinosteroid-responsive genes." FEBS letters 563.1-3 (2004): 82-86.
Solyc07g052510.4	2.13	peroxidase 3 precursor	PER3	Involved in removal of H2O2, oxidation of toxic reductants, biosynthesis and degradation of lignin, suberization, auxin catabolism, response to environmental stressors	Increased removal of H2O2, oxidation of toxic reductants, biosynthesis and degradation of lignin, suberization, auxin catabolism, and response to environmental stressors	Paynel, Florence, et al. "Temporal regulation of cell-wall pectin methylesterase and peroxidase isoforms in cadmium-treated flax hypocotyl." Annals of botany 104.7 (2009): 1363-1372.
Solyc07g064820.1	2.13	mitogen-activated protein kinase kinase kinase 18-like	MAPKKK18	Act as ABA signal transducer under abiotic stress; Promotes stomatal growth/development; Inhibits germination and root growth; Promotes leaf senescence	Increased response to stress; Increased reproductive development; Decreased growth/development; Increased leaf senescence	Mitula, Filip, et al. "Arabidopsis ABA-activated kinase MAPKKK18 is regulated by protein phosphatase 2C ABI1 and the ubiquitin–proteasome pathway." Plant and Cell Physiology 56.12 (2015): 2351-2367.
Solyc03g116070.1	2.12	mini zinc finger protein 3	MIF3	Involved in integrating signals from multiple hormones by regulating the expression of specific genes; Promotes the formation of ectopic shoot meristems	*Increased hormonal signaling; Promoted formation of ectopic shoot meristems*	Hu, Wei, Baomin Feng, and Hong Ma. "Ectopic expression of the Arabidopsis MINI ZINC FINGER1 and MIF3 genes induces shoot meristems on leaf margins." Plant molecular biology 76.1-2 (2011): 57-68.
Solyc12g014420.2	2.12	glucan endo-1,3-beta-glucosidase 13-like	AT5G56590	Involved in carbohydrate metabolic process, cell wall organization, and plant defense response	Increased carbohydrate metabolism, cell wall organization, and plant defense response	Wu, Qiong, et al. "Long-term balancing selection contributes to adaptation in Arabidopsis and its relatives." Genome biology 18.1 (2017): 1-15.
Solyc11g010390.1	2.11	classical arabinogalactan protein 10-like	AGP10	Proteoglycan involved in differentiation, cell-cell recognition, embryogenesis, and programmed cell death	*Promoted plant growth/development and cell differentiation; Promoted programmed cell death*	Thieme, Christoph J., et al. "Endogenous Arabidopsis messenger RNAs transported to distant tissues." Nature Plants 1.4 (2015): 1-9.
Solyc05g015840.3	2.10	teosinte glume architecture 1	TGA1	Acts as a transcriptional repressor of growth of lateral branches in length and numbers	Repressed lateral branching and growth	Wang, Huai, et al. "Evidence that the origin of naked kernels during maize domestication was caused by a single amino acid substitution in tga1." Genetics 200.3 (2015): 965-974.
Solyc06g069070.1	2.08	non-specific lipid-transfer protein 2	LTP2	Plays a role in wax/cutin deposition in the cell walls of expanding epidermal cells and certain secretory tissues	*Promoted cell wall expansion*	Peragine, Angela, et al. "SGS3 and SGS2/SDE1/RDR6 are required for juvenile development and the production of trans-acting siRNAs in Arabidopsis." Genes & development 18.19 (2004): 2368-2379.
Solyc03g116590.3	2.07	embryo-specific protein ATS3B	ATS3B	Plays a role during embryo development	Promoted embryo development	Sato, Shusei, et al. "Structural analysis of Arabidopsis thaliana chromosome 5. X. Sequence features of the regions of 3,076,755 bp covered by sixty P1 and TAC clones." DNA research 7.1 (2000): 31-63.

TomatoID	DE in Infected	NCBI Protein Name	Gene ID	Uniprot Description	Putative Consequences for Infection	Citation
Solyc05g005160.3	2.07	ATP-citrate synthase alpha chain protein 2	ACLA-2	ATP citrate-lyase is the primary enzyme responsible for the synthesis of cytosolic acetyl-CoA; Required for normal growth and development in seeds	Promoted seed development; Increased production of primary metabolites	Fatland, Beth L., Basil J. Nikolau, and Eve Syrkin Wurtele. "Reverse genetic characterization of cytosolic acetyl-CoA generation by ATP-citrate lyase in Arabidopsis." The Plant Cell 17.1 (2005): 182-203.
Solyc10g008000.1	2.07	protein LIGHT-DEPENDENT SHORT HYPOCOTYLS 10-like	LSH10	Transcription regulator that acts as a developmental regulator by promoting cell growth in response to light	*Promoted cell growth in response to light*	Iyer, Lakshminarayan M., and L. Aravind. "ALOG domains: provenance of plant homeotic and developmental regulators from the DNA-binding domain of a novel class of DIRS1-type retroposons." Biology direct 7.1 (2012): 1-8.
Solyc10g006660.3	2.06	calcium-binding protein KRP1	KRP1	Potential calcium sensor	*Promoted calcium sensing; Promoted regulation of trichome morphogenesis*	Reddy, Vaka S., et al. "KIC, a novel Ca2+ binding protein with one EF-hand motif, interacts with a microtubule motor protein and regulates trichome morphogenesis." The Plant Cell 16.1 (2004): 185-200.
Solyc09g008280.2	2.04	S-adenosylmethionine synthase 3	METK3	Catalyzes the formation of S-adenosylmethionine from methionine; Involved in SA-mediated defense; Involved in the biosynthesis of lignin	*Increased S-adenosylmethionine production; Promoted early defense response; Increased lignin biosynthesis*	Goto, Derek B., et al. "A single-nucleotide mutation in a gene encoding S-adenosylmethionine synthetase is associated with methionine over-accumulation phenotype in Arabidopsis thaliana." Genes & genetic systems 77.2 (2002): 89-95.
Solyc02g093230.3	2.03	caffeoyl-CoA O-methyltransferase 1	CCOAOMT1	Involved in the reinforcement of the plant cell wall; Involved in response to wounding or pathogen challenge by promoting the formation of cell wall-bound ferulic acid polymers	*Increased production of feruloylated polysaccharides; Promoted plant cell wall development and integrity*	Meyermans, Hugo, et al. "Modifications in lignin and accumulation of phenolic glucosides in poplar xylem upon down-regulation of caffeoyl-coenzyme A O-methyltransferase, an enzyme involved in lignin biosynthesis." Journal of Biological Chemistry 275.47 (2000): 36899-36909.
Solyc01g107390.3	2.02	indole-3-acetic acid-amido synthetase GH3.2	GH3.2	Catalyzes the synthesis of indole-3-acetic acid-amino acid conjugates, providing a mechanism for the plant to cope with the presence of excess auxin	*Promoted auxin homeostasis*	Hilson, Pierre, et al. "Versatile gene-specific sequence tags for Arabidopsis functional genomics: transcript profiling and reverse genetics applications." Genome research 14.10b (2004): 2176-2189.
Solyc03g112960.1	2.02	pectinesterase 1	PME1	Acts in the modification of cell walls; Acts as negative regulator of genes involved in salt stress response	*Increased modification of plant cell walls; Impaired salt stress response*	Creighton, Maria T., et al. "Methylation of protein phosphatase 2A—Influence of regulators and environmental stress factors." Plant, cell & environment 40.10 (2017): 2347-2358.
Solyc10g050160.2	2.02	caffeoyl-CoA O-methyltransferase 5	CCOAOMT5	Plays a role in the synthesis of feruloylated polysaccharides; Involved in the reinforcement of the plant cell wall; Involved in response to wounding or pathogen challenge	*Increased production of feruloylated polysaccharides; Promoted plant cell wall development and integrity*	Meyermans, Hugo, et al. "Modifications in lignin and accumulation of phenolic glucosides in poplar xylem upon down-regulation of caffeoyl-coenzyme A O-methyltransferase, an enzyme involved in lignin biosynthesis." Journal of Biological Chemistry 275.47 (2000): 36899-36909.
Solyc03g006880.3	-2.02	gibberellin 20-oxidase	GA20OX1	Promotes transition from vegetative to floral state, plant fertility, and silique elongation	Impaired plant reproduction	Rieu, Ivo, et al. "The gibberellin biosynthetic genes AtGA20ox1 and AtGA20ox2 act, partially redundantly, to promote growth and development throughout the Arabidopsis life cycle." The Plant Journal 53.3 (2008): 488-504.

TomatoID	DE in Infected	NCBI Protein Name	Gene ID	Uniprot Description	Putative Consequences for Infection	Citation
Solyc08g066350.2	-2.02	histidine-containing phosphotransfer protein 4-like	AHP4	Plays an important role in propagating cytokinin signal transduction through the multistep His-to-Asp phosphorelay	Impaired stem and bud growth; Impaired response to auxin	Hwang, Ildoo, Huei-Chi Chen, and Jen Sheen. "Two-component signal transduction pathways in Arabidopsis." Plant Physiology 129.2 (2002): 500-515.
Solyc11g012680.2	-2.02	pollen-specific leucine-rich repeat extensin-like protein 1	PEX1	Modulates cell morphogenesis by regulating cell wall formation and assembly, and/or growth polarization	Impaired cell morphogenesis by regulating cell wall formation and assembly, and/or growth polarization	Mecchia, Martin A., et al. "RALF4/19 peptides interact with LRX proteins to control pollen tube growth in Arabidopsis." Science 358.6370 (2017): 1600-1603.
Solyc12g056950.2	-2.07	protein MARD1-like	MARD1	Facilitates the interaction of SnRK1 complex with effector proteins; Involved in seed dormancy control	Impaired seed dormancy	He, Yuehui, and Susheng Gan. "A novel zinc-finger protein with a proline-rich domain mediates ABA-regulated seed dormancy in Arabidopsis." Plant molecular biology 54.1 (2004): 1-9.
Solyc03g025350.3	-2.08	silicon efflux transporter LSI2-like	LSI2	Silicon efflux transporter involved in silicon transport from the root cells to the apoplast	Impaired cell wall response to abiotic and biotic stressors	Ma, Jian Feng, et al. "An efflux transporter of silicon in rice." Nature 448.7150 (2007): 209-212.
Solyc08g068490.3	-2.09	indole-3-acetic acid-amido synthetase GH3.10-like	GH3.10	Catalyzes the synthesis of indole-3-acetic acid-amino acid conjugates, providing a mechanism for the plant to cope with the presence of excess auxin	Impaired auxin homeostasis	Takase, Tomoyuki, et al. "DFL2, a new member of the Arabidopsis GH3 gene family, is involved in red light-specific hypocotyl elongation." Plant and Cell Physiology 44.10 (2003): 1071-1080.
Solyc07g014620.1	-2.11	auxin-responsive protein SAUR50	SAUR50	Effector of hormonal and environmental signals in plant growth	Impaired response to hormonal and environmental signaling; Impaired growth/development	Ren, Hong, and William M. Gray. "SAUR proteins as effectors of hormonal and environmental signals in plant growth." Molecular plant 8.8 (2015): 1153-1164.
Solyc02g084930.3	-2.12	abscisic acid 8'-hydroxylase 3-like	CYP707A3	Involved in the oxidative degradation of ABA; Involved in the control of postgermination growth	Impaired degradation of ABA; Impaired postgermination growth	Umezawa, Taishi, et al. "CYP707A3, a major ABA 8′-hydroxylase involved in dehydration and rehydration response in Arabidopsis thaliana." The Plant Journal 46.2 (2006): 171-182.
Solyc06g060830.3	-2.17	homeobox-leucine zipper protein HAT4	HAT4	Involved in the negative regulation of cell elongation and specific cell proliferation processes; Mediator of the red/far-red light effects on leaf cell expansion in the shading response	Impaired regulation of cell elongation; Impaired regulation of lateral root formation and vascular system; Impaired shading response	Schena, Mark, Alan M. Lloyd, and Ronald W. Davis. "The HAT4 gene of Arabidopsis encodes a developmental regulator." Genes & development 7.3 (1993): 367-379.
Solyc12g005750.1	-2.29	B-box domain protein 30	MIP1A	Developmental regulator; Involved in the CO-mediated long-day flowering-promotion pathway	Decreased sequestration of CO and CO-like proteins; Impaired flowering	Graeff, Moritz, et al. "MicroProtein-mediated recruitment of CONSTANS into a TOPLESS trimeric complex represses flowering in Arabidopsis." PLoS genetics 12.3 (2016): e1005959.
Solyc01g007810.1	-2.37	transcription repressor OFP15	OFP15	Transcriptional repressor that regulates multiple aspects of plant growth and development	Promoted plant growth and development; Impaired regulation of plant growth and development	Wang, Shucai, et al. "Arabidopsis ovate family proteins, a novel transcriptional repressor family, control multiple aspects of plant growth and development." PLoS One 6.8 (2011): e23896.
Solyc05g007880.3	-2.46	cyclic dof factor 1 isoform X1	CDF1	Regulates a photoperiodic flowering response; Transcriptional repressor of 'CONSTANS' expression	Impaired photoperiodic flowering response; Decreased repression of CONSTANS expression	Imaizumi, Takato, et al. "FKF1 F-box protein mediates cyclic degradation of a repressor of CONSTANS in Arabidopsis." Science 309.5732 (2005): 293-297.

TomatoID	DE in Infected	NCBI Protein Name	Gene ID	Uniprot Description	Putative Consequences for Infection	Citation
Solyc09g065390.1	-2.47	protein MARD1	MARD1	Acts as an adapter to facilitate the interaction of SnRK1 complex with effector proteins; Involved in seed dormancy control	Impaired seed dormancy	He, Yuehui, and Susheng Gan. "A novel zinc-finger protein with a proline-rich domain mediates ABA-regulated seed dormancy in Arabidopsis." Plant molecular biology 54.1 (2004): 1-9.
Solyc01g081060.3	-2.54	xyloglucan endotransglucosylase-hydrolase XTH5	XTH5	Catalyzes xyloglucan endohydrolysis and endotransglycosylation; Essential constituent of the primary cell wall; Participates in cell wall construction	Impaired plant cell wall elongation; Impaired cell wall integrity	Hyodo, Hideki, et al. "Active gene expression of a xyloglucan endotransglucosylase/hydrolase gene, XTH9, in inflorescence apices is related to cell elongation in Arabidopsis thaliana." Plant molecular biology 52.2 (2003): 473-482.
Solyc01g110580.2	-2.55	auxin-responsive protein SAUR50	SAUR50	Effector of hormonal and environmental signals in plant growth	Impaired response to hormonal and environmental signaling; Impaired growth/development	Ren, Hong, and William M. Gray. "SAUR proteins as effectors of hormonal and environmental signals in plant growth." Molecular plant 8.8 (2015): 1153-1164.
Solyc04g071780.3	-2.60	cytochrome P450 71A1	CYP71A1	Involved in the metabolism of compounds associated with the development of flavor in the ripening fruit process	Impaired fruit ripening	O'Keefe, Daniel P., and Kenneth J. Leto. "Cytochrome P-450 from the mesocarp of avocado (Persea americana)." Plant physiology 89.4 (1989): 1141-1149.
Solyc12g087870.2	-2.62	purine permease 3-like	PUP3	Involved in transport of purine derivatives during pollen germination and tube elongation	Impaired transport of purine derivatives during pollen germination and tube elongation	Bürkle, Lukas, et al. "Transport of cytokinins mediated by purine transporters of the PUP family expressed in phloem, hydathodes, and pollen of Arabidopsis." The Plant Journal 34.1 (2003): 13-26.
Solyc05g012030.1	-2.63	protein BIG GRAIN 1-like E	AT1G69160	Involved in auxin transport; Regulator of the auxin signaling pathway	Impaired auxin transport; Impaired regulation of auxin signaling	Culligan, Kevin M., et al. "ATR and ATM play both distinct and additive roles in response to ionizing radiation." The Plant Journal 48.6 (2006): 947-961.
Solyc06g068500.3	-2.77	chaperone protein dnaJ 8, chloroplastic-like	ATJ8	Plays a continuous role in plant development and the structural organization of compartments	Impaired plant development and structural organization	Nanjo, Tokihiko, et al. "Toxicity of free proline revealed in an Arabidopsis T-DNA-tagged mutant deficient in proline dehydrogenase." Plant and Cell Physiology 44.5 (2003): 541-548.
Solyc08g079230.1	-3.32	14 kDa proline-rich protein DC2.15-like	N/A	Involved with the initiation of embryogenesis or with the metabolic changes produced by the removal of auxins	Impaired embryogenesis; Impaired response to auxin signaling	Aleith, F., and G. Richter. "Gene expression during induction of somatic embryogenesis in carrot cell suspensions." Planta 183.1 (1991): 17-24.
Solyc07g006310.1	-3.43	transcription factor IBH1-like 1	IBL1	Acts as transcriptional repressor that negatively regulates cell and organ elongation in response to gibberellin and brassinosteroid signaling	Impaired regulation of cell and organ elongation in response to gibberellin and brassinosteroid signaling	Zhiponova, Miroslava K., et al. "Helix–loop–helix/basic helix–loop–helix transcription factor network represses cell elongation in Arabidopsis through an apparent incoherent feed-forward loop." Proceedings of the National Academy of Sciences 111.7 (2014): 2824-2829.
