# Supplementary material for: Effects of ‘Candidatus Liberibacter solanacearum’ haplotypes A and B on tomato gene expression and geotropism
Source: BMC Plant Biol. 2022 Mar 30;22:156. doi: 10.1186/s12870-022-03505-z (PMC8966271; doi:10.1186/s12870-022-03505-z)
Supplement: Supplementary file 7 — Additional file 7: Table S7. The 98 tomato plant DEGs associated with plant primary metabolism under Lso infection. DEGs were sorted by differential expression (DE) values comparing uninfected to infected samples (p-value < 0.05). NCBI Blast searches were used to identify Gene IDs and protein products in tomatoes as well as their homologs in other species. Specifically, the expression changes in 78 of these genes (80%, in italics) would have promoted primary metabolic processes in infected plants. These DEGs were related to photosynthesis, protein turnover, fatty acid and sugar metabolism, and biosynthesis. [file 12870_2022_3505_MOESM7_ESM.pdf]

TomatoID	DE in Infected	NCBI Protein Name	Gene ID	Uniprot Description	Putative Consequences for Infection	Citation
Solyc12g010020.2	9.14	leucine aminopeptidase 1, chloroplastic	LAPA1	Involved in the processing and regular turnover of intracellular proteins	*Increased intracellular protein turnover*	Pautot, Veronique, et al. "Leucine aminopeptidase: an inducible component of the defense response in Lycopersicon esculentum (tomato)." Proceedings of the National Academy of Sciences 90.21 (1993): 9906-9910.
Solyc00g187050.3	8.79	leucine aminopeptidase 1, chloroplastic	LAPA1	Involved in the processing and regular turnover of intracellular proteins	*Increased intracellular protein turnover*	Pautot, Veronique, et al. "Leucine aminopeptidase: an inducible component of the defense response in Lycopersicon esculentum (tomato)." Proceedings of the National Academy of Sciences 90.21 (1993): 9906-9910.
Solyc12g010030.2	8.64	leucine aminopeptidase 2, chloroplastic isoform X1	LAP2	Involved in the processing and regular turnover of intracellular proteins; Heat shock protein	*Increased turnover of intracellular proteins; Increased protection from heat-induced damaged*	Waditee-Sirisattha, Rungaroon, et al. "The Arabidopsis aminopeptidase LAP2 regulates plant growth, leaf longevity and stress response." New Phytologist 191.4 (2011): 958-969.
Solyc06g062370.3	7.18	Glucose-1-phosphate adenylyltransferase small subunit, chloroplastic	APS1	Plays a role in starch synthesis; Catalyzes the synthesis of the activated glycosyl donor, ADP-glucose from Glc-1-P, and ATP	*Increased starch synthesis*	Tanaka, Hiroshi, et al. "Isolation and some properties of acid phosphatase-11 from tomato leaves." Agricultural and biological chemistry 54.8 (1990): 1947-1952.
Solyc12g096780.2	6.48	enoyl-[acyl-carrier-protein] reductase, mitochondrial-like	AT3G45770	Catalyzes the NADPH-dependent reduction of trans-2-enoyl thioesters in mitochondrial fatty acid synthesis	*Increased fatty acid synthesis and metabolism*	Li-Beisson, Yonghua, et al. "Acyl-lipid metabolism." The Arabidopsis book/American Society of Plant Biologists 11 (2013).
Solyc01g091170.3	6.10	Arginase 2, chloroplastic/mitochondrial	ARGAH2	Utilized in the urea cycle; Precursor for the synthesis of both polyamines and proline; Catalyzes the formation of putrescine from agmatine	*Promoted jasmonate-related functions; Increased urea waste management; Increased freezing tolerance*	Dombrecht, Bruno, et al. "MYC2 differentially modulates diverse jasmonate-dependent functions in Arabidopsis." The Plant Cell 19.7 (2007): 2225-2245.
Solyc12g010980.2	5.65	acetyl-CoA-benzylalcohol acetyltransferase	BEAT	Involved in the biosynthesis of benzyl acetate, a major constituent of the floral scent	*Increased synthesis of benzyl acetate*	Dudareva, Natalia, et al. "Acetyl-CoA: benzylalcohol acetyltransferase–an enzyme involved in floral scent production in Clarkia breweri." The Plant Journal 14.3 (1998): 297-304.
Solyc10g005320.3	4.70	tryptophan synthase beta chain 1-like	TSB1	The beta subunit is responsible for the synthesis of L-tryptophan from indole and L-serine	*Increased synthesis of L-tryptophan from indole and L-serine*	Ouyang, Jian, Xiang Shao, and Jiayang Li. "Indole-3-glycerol phosphate, a branchpoint of indole-3-acetic acid biosynthesis from the tryptophan biosynthetic pathway in Arabidopsis thaliana." The Plant Journal 24.3 (2000): 327-334.
Solyc12g096770.1	4.69	acetyl-CoA-benzylalcohol acetyltransferase-like	BEAT	Involved in the biosynthesis of benzyl acetate, a major constituent of the floral scent	*Increased synthesis of benzyl acetate*	Dudareva, Natalia, et al. "Acetyl-CoA: benzylalcohol acetyltransferase–an enzyme involved in floral scent production in Clarkia breweri." The Plant Journal 14.3 (1998): 297-304.
Solyc09g010980.1	4.49	laccase-17	LAC17	Involved in lignin degradation and detoxification of lignin-derived products	*Increased lignin degradation and detoxification of lignin-derived products*	Sibout, Richard, et al. "CINNAMYL ALCOHOL DEHYDROGENASE-C and-D are the primary genes involved in lignin biosynthesis in the floral stem of Arabidopsis." The Plant Cell 17.7 (2005): 2059-2076.

TomatoID	DE in Infected	NCBI Protein Name	Gene ID	Uniprot Description	Putative Consequences for Infection	Citation
Solyc07g056000.2	4.25	xyloglycan endo-transglycosylase precursor	XET1	Involved in carbohydrate metabolic process	*Increased carbohydrate metabolism*	Tucker, Mark L., et al. "Gene expression profiles for cell wall-modifying proteins associated with soybean cyst nematode infection, petiole abscission, root tips, flowers, apical buds, and leaves." Journal of Experimental Botany 58.12 (2007): 3395-3406.
Solyc10g086500.1	3.78	steroid 5-alpha-reductase DET2	DET2	Involved in a reduction step in the biosynthesis of the plant steroid, brassinolide	*Increased brassinolide biosynthesis; Promoted cell elongation*	Fujioka, Shozo, et al. "The Arabidopsis deetiolated2 mutant is blocked early in brassinosteroid biosynthesis." The Plant Cell 9.11 (1997): 1951-1962.
Solyc11g018800.2	3.77	lignin-forming anionic peroxidase	N/A	Involved in removal of H2O2, oxidation of toxic reductants, biosynthesis and degradation of lignin, suberization, auxin catabolism, and response to biotic stressors	*Increased response to oxidative stress; Increased auxin catabolism; Increased defense against wounding and pathogen attack*	Lagrimini, L. Mark, et al. "Molecular cloning of complementary DNA encoding the lignin-forming peroxidase from tobacco: molecular analysis and tissue-specific expression." Proceedings of the National Academy of Sciences 84.21 (1987): 7542-7546.
Solyc12g057060.2	3.74	7-deoxyloganetin glucosyltransferase-like	N/A	Iridoid glucosyltransferase acting exclusively on 7-deoxyloganetin; Involved in the synthesis of secologanin	*Increassed synthesis of secologanin*	Asada, Keisuke, et al. "A 7-deoxyloganetic acid glucosyltransferase contributes a key step in secologanin biosynthesis in Madagascar periwinkle." The Plant Cell 25.10 (2013): 4123-4134.
Solyc08g068600.3	3.68	aromatic amino acid decarboxylase 1B	AADC1B	Involved in carboxylic acid metabolic process	*Increased carboxylic acid metabolism*	Tieman, Denise, et al. "Tomato aromatic amino acid decarboxylases participate in synthesis of the flavor volatiles 2-phenylethanol and 2-phenylacetaldehyde." Proceedings of the National Academy of Sciences 103.21 (2006): 8287-8292.
Solyc10g076660.2	3.54	probable 2-oxoglutarate-dependent dioxygenase At5g05600 isoform X1	AT5G05600	Involved in anthocyanin and protoanthocyanidin biosynthesis	*Increased anthocyanin and protoanthocyanidin biosynthesis*	Wang, Yi, et al. "Transcriptome analyses show changes in gene expression to accompany pollen germination and tube growth in Arabidopsis." Plant physiology 148.3 (2008): 1201-1211.
Solyc10g009270.3	3.50	transcription factor MYC2-like	MYC2	Involved in the regulation of ABA-inducible genes under drought stress; Negative regulator of light-regulated gene expression and growth; Positive regulator of lateral root formation; Regulates sesquiterpene biosynthesis	*Promoted response to drought; Impaired response to light-mediated expression; Promoted lateral root formation; Increased regulation of sesquiterpene biosynthesis*	Abe, Hiroshi, et al. "Role of Arabidopsis MYC and MYB homologs in drought-and abscisic acid-regulated gene expression." The Plant Cell 9.10 (1997): 1859-1868.
Solyc09g011860.3	3.44	O-fucosyltransferase 19-like	OFUT19	Involved in Glycan metabolism	*Increased Glycan metabolism*	Culligan, Kevin M., et al. "ATR and ATM play both distinct and additive roles in response to ionizing radiation." The Plant Journal 48.6 (2006): 947-961.
Solyc10g005480.3	3.38	F-box protein SKIP27	SKIP27	Component of SCF(ASK-cullin-F-box) E3 ubiquitin ligase complexes, which mediate the ubiquitination and subsequent proteasomal degradation of target proteins	*Increased proteasomal degradation*	Risseeuw, Eddy P., et al. "Protein interaction analysis of SCF ubiquitin E3 ligase subunits from Arabidopsis." The Plant Journal 34.6 (2003): 753-767.

TomatoID	DE in Infected	NCBI Protein Name	Gene ID	Uniprot Description	Putative Consequences for Infection	Citation
Solyc10g084320.2	3.34	subtilisin-like protease SBT1.9	SBT1.9	Involved in proteolysis	*Increased proteolysis*	Barrero, José María, et al. "INCURVATA2 encodes the catalytic subunit of DNA polymerase α and interacts with genes involved in chromatin-mediated cellular memory in Arabidopsis thaliana." The Plant Cell 19.9 (2007): 2822-2838.
Solyc03g096050.3	3.30	probable 2-oxoglutarate-dependent dioxygenase At5g05600	AT5G05600	Involved in anthocyanin and protoanthocyanidin biosynthesis	*Increased anthocyanin and protoanthocyanidin biosynthesis*	Wang, Yi, et al. "Transcriptome analyses show changes in gene expression to accompany pollen germination and tube growth in Arabidopsis." Plant physiology 148.3 (2008): 1201-1211.
Solyc02g077330.3	3.29	GDSL-like lipase/acylhydrolase superfamily protein precursor	AT1G20132	Lipid metabolic process	*Increased lipid catabolism*	Cheng, Chia-Yi, et al. "Araport11: a complete reannotation of the Arabidopsis thaliana reference genome." The Plant Journal 89.4 (2017): 789-804.
Solyc08g068610.3	3.29	aromatic amino acid decarboxylase 1B	AADC1B	Involved in carboxylic acid metabolic process	*Increased carboxylic acid metabolism*	Tieman, Denise, et al. "Tomato aromatic amino acid decarboxylases participate in synthesis of the flavor volatiles 2-phenylethanol and 2-phenylacetaldehyde." Proceedings of the National Academy of Sciences 103.21 (2006): 8287-8292.
Solyc06g074530.1	3.07	arogenate dehydratase/prephenate dehydratase 6, chloroplastic-like	ADT6	Converts the prephenate produced from the shikimate-chorismate pathway into phenylalanine	*Increased phenylalanine production; Increased production of primary and secondary metabolites*	Cho, Man-Ho, et al. "Phenylalanine biosynthesis in Arabidopsis thaliana identification and characterization of Arogenate dehydratases." Journal of Biological Chemistry 282.42 (2007): 30827-30835.
Solyc12g005450.1	3.07	probable receptor-like protein kinase At4g10390	AT4G10390	Involved in protein phosphorylation; Involved in response to wounding	*Increased protein phosphorylation; Promoted response to wounding*	Peng, Mingsheng, et al. "Genome-wide analysis of Arabidopsis responsive transcriptome to nitrogen limitation and its regulation by the ubiquitin ligase gene NLA." Plant molecular biology 65.6 (2007): 775-797.
Solyc07g048060.2	3.06	cytochrome b561 and DOMON domain-containing protein At5g48750	AT5G48750	Involved in oxidation-reduction process	*Improved oxidation-reduction*	Kotani, Hirokazu, et al. "Structural analysis of Arabidopsis thaliana chromosome 5. VI. Sequence features of the regions of 1,367,185 bp covered by 19 physically assigned P1 and TAC clones." DNA Research 5.3 (1998): 203-216.
Solyc03g119980.3	3.03	caffeoylshikimate esterase	CSE	Involved in the biosynthesis of lignin; Promotes the degradation of lysophosphatidylcholine and detoxifies the peroxidized membrane in response to cadmium-induced oxidative stress	*Increased biosynthesis of lignin; Increased detoxification of peroxidized membrane; Increased response to oxidative stress*	Gao, Wei, et al. "Acyl-CoA-binding protein 2 binds lysophospholipase 2 and lysoPC to promote tolerance to cadmium-induced oxidative stress in transgenic Arabidopsis." The Plant Journal 62.6 (2010): 989-1003.
Solyc01g090180.3	3.02	4,5-DOPA dioxygenase extradiol	DODA	Opens the cyclic ring of dihydroxy-phenylalanine, producing betalamic acid	*Increased betalamic acid production; Increased pigment production*	Christinet, Laurent, et al. "Characterization and functional identification of a novel plant 4, 5-extradiol dioxygenase involved in betalain pigment biosynthesis in Portulaca grandiflora." Plant Physiology 134.1 (2004): 265-274.

TomatoID	DE in Infected	NCBI Protein Name	Gene ID	Uniprot Description	Putative Consequences for Infection	Citation
Solyc08g006740.3	2.98	aromatic amino acid decarboxylase 2	AADC2	Involved in carboxylic acid metabolic process	*Increased carboxylic acid metabolism*	Tieman, Denise, et al. "Tomato aromatic amino acid decarboxylases participate in synthesis of the flavor volatiles 2-phenylethanol and 2-phenylacetaldehyde." Proceedings of the National Academy of Sciences 103.21 (2006): 8287-8292.
Solyc10g076240.2	2.86	cationic peroxidase 1	PNC1	Involved in removal of H2O2, oxidation of toxic reductants, biosynthesis/degradation of lignin, suberization, auxin catabolism, and response to environmental stressesors	*Promoted response to oxidative stress and other environmental stressors; Increased lignin and auxin metabolism*	Buffard, Dominique, et al. "Molecular cloning of complementary DNAs encoding two cationic peroxidases from cultivated peanut cells." Proceedings of the National Academy of Sciences 87.22 (1990): 8874-8878.
Solyc10g085190.2	2.82	probable 2-oxoglutarate-dependent dioxygenase At3g11180	AT3G11180	Involved in anthocyanin and protoanthocyanidin biosynthesis by catalyzing the oxidation of leucoanthocyanidins into anthocyanidins	*Increased anthocyanin and protoanthocyanidin biosynthesis*	Wang, Yi, et al. "Transcriptome analyses show changes in gene expression to accompany pollen germination and tube growth in Arabidopsis." Plant physiology 148.3 (2008): 1201-1211.
Solyc03g117600.3	2.81	shikimate O-hydroxycinnamoyltransferase	HST	Acyltransferase involved in the biosynthesis of lignin	*Increased biosynthesis of lignin*	Hoffmann, Laurent, et al. "Silencing of hydroxycinnamoyl-coenzyme A shikimate/quinate hydroxycinnamoyltransferase affects phenylpropanoid biosynthesis." The Plant Cell 16.6 (2004): 1446-1465.
Solyc09g091600.3	2.77	protein SULFUR DEFICIENCY-INDUCED 2	AT1G04770	Involved in the utilization of stored sulfate under sulfur-deficient conditions	*Increased storage of sulfate; Increased response to sulfur deficiency*	Hanada, Kousuke, et al. "Functional compensation of primary and secondary metabolites by duplicate genes in Arabidopsis thaliana." Molecular biology and evolution 28.1 (2011): 377-382.
Solyc03g093610.1	2.77	ethylene response factor A.2	ERF2	Involved in defense response; Involved in intracellular signal transduction; Involved in lipid metabolism	*Increased defense response; Increased intracellular signal transduction; Increased lipid metabolism*	Zhang, Zhijin, et al. "Transcriptional regulation of the ethylene response factor LeERF2 in the expression of ethylene biosynthesis genes controls ethylene production in tomato and tobacco." Plant Physiology 150.1 (2009): 365-377.
Solyc06g083440.3	2.73	cytochrome b5-like	CB5LP	Electron transfer activity; Heme binding; Metal ion binding	*Impaired methionine catabolism*	Rébeillé, Fabrice, et al. "Methionine catabolism in Arabidopsis cells is initiated by a γ-cleavage process and leads to S-methylcysteine and isoleucine syntheses." Proceedings of the National Academy of Sciences 103.42 (2006): 15687-15692.
Solyc01g098910.3	2.71	peroxisomal adenine nucleotide carrier 1	PNC1	Required for the conversion of seed-reserved triacylglycerols into sucrose; Necessary for growth before the onset of photosynthesis	*Increased conversion of seed-reserved triacylglycerols; Promoted growth over storage*	Arai, Yuko, Makoto Hayashi, and Mikio Nishimura. "Proteomic identification and characterization of a novel peroxisomal adenine nucleotide transporter supplying ATP for fatty acid β-oxidation in soybean and Arabidopsis." The Plant Cell 20.12 (2008): 3227-3240.
Solyc12g099000.2	2.62	S-adenosylmethionine synthase 2	SAM2	Involved in SA-mediated defense	*Increased S-adenosylmethionine production; Promoted early defense response*	Rajjou, Loïc, et al. "Proteomic investigation of the effect of salicylic acid on Arabidopsis seed germination and establishment of early defense mechanisms." Plant physiology 141.3 (2006): 910-923.
Solyc02g071610.3	2.60	GDSL esterase/lipase At5g45670	AT5G45670	Involved in lipid catabolism	*Increased lipid catabolism*	Rouhier, Nicolas, et al. "Identification of plant glutaredoxin targets." Antioxidants & redox signaling 7.7-8 (2005): 919-929.

TomatoID	DE in Infected	NCBI Protein Name	Gene ID	Uniprot Description	Putative Consequences for Infection	Citation
Solyc04g082030.1	2.60	ornithine decarboxylase	ODC	Catalyzes the first and rate-limiting step of polyamine biosynthesis that converts ornithine into putrescine, which is the precursor for the polyamines, spermidine, and spermine	*Increased production of polyamines, spermidine, and spermine*	Alabadı, David, and Juan Carbonell. "Expression of ornithine decarboxylase is transiently increased by pollination, 2, 4-dichlorophenoxyacetic acid, and gibberellic acid in tomato ovaries." Plant physiology 118.1 (1998): 323-328.
Solyc04g011790.1	2.57	monothiol glutaredoxin-S1-like	GRXS1	Reduces GSH-thiol disulfides	*Increased reduction of GSH-thiol disulfides*	Patterson, Kurt, et al. "Nitrate-regulated glutaredoxins control Arabidopsis primary root growth." Plant Physiology 170.2 (2016): 989-999.
Solyc10g083970.1	2.57	S-adenosylmethionine synthase 3-like	METK3	Catalyzes the formation of S-adenosylmethionine from methionine; Involved in SA-mediated defense; Involved in the biosynthesis of lignin	*Increased S-adenosylmethionine production; Promoted early defense response; Increased lignin biosynthesis*	Goto, Derek B., et al. "A single-nucleotide mutation in a gene encoding S-adenosylmethionine synthetase is associated with methionine over-accumulation phenotype in Arabidopsis thaliana." Genes & genetic systems 77.2 (2002): 89-95.
Solyc07g008380.2	2.54	salutaridinol 7-O-acetyltransferase-like	SALAT	Involved in biosynthesis of morphinan-type benzylisoquinoline alkaloids	*Increased in biosynthesis of morphinan-type benzylisoquinoline alkaloids*	Lenz, Rainer, and Meinhart H. Zenk. "Acetyl coenzyme A: salutaridinol-7-O-acetyltransferase from Papaver somniferum plant cell cultures: The enzyme catalyzing the formation of thebaine in morphine biosynthesis." Journal of Biological Chemistry 270.52 (1995): 31091-31096.
Solyc10g086690.2	2.53	phosphatidylinositol:ceramide inositolphosphotransferase 2	IPCS2	Essential for sphingolipid biosynthesis; Plays an important role in modulating plant programmed cell death	*Increased sphingolipid biosynthesis; Promoted programmed cell death*	Wang, Wenming, et al. "An inositolphosphorylceramide synthase is involved in regulation of plant programmed cell death associated with defense in Arabidopsis." The Plant Cell 20.11 (2008): 3163-3179.
Solyc05g008370.1	2.52	probable ribose-5-phosphate isomerase 2	RPI2	Involved in programmed cell death; Involved in vegetative-to-reproductive phase transition in meristems	*Increased primary metabolism; Promoted programmed cell death; Promoted transition from vegetative-to-reproductive phase*	Xiong, Yuqing, et al. "Deficiency in a cytosolic ribose-5-phosphate isomerase causes chloroplast dysfunction, late flowering and premature cell death in Arabidopsis." Physiologia plantarum 137.3 (2009): 249-263.
Solyc03g031620.3	2.50	5'-adenylylsulfate reductase 3, chloroplastic-like	APR3	Reduces sulfate for Cys biosynthesis	*Increased Cys biosynthesis*	Tsakraklides, George, et al. "Sulfate reduction is increased in transgenic Arabidopsis thaliana expressing 5′-adenylylsulfate reductase from Pseudomonas aeruginosa." The Plant Journal 32.6 (2002): 879-889.
Solyc08g068680.3	2.48	aromatic amino acid decarboxylase 1A	AADC1A	Involved in carboxylic acid metabolic process	*Increased carboxylic acid metabolism*	Tieman, Denise, et al. "Tomato aromatic amino acid decarboxylases participate in synthesis of the flavor volatiles 2-phenylethanol and 2-phenylacetaldehyde." Proceedings of the National Academy of Sciences 103.21 (2006): 8287-8292.
Solyc02g032860.3	2.47	5'-adenylylsulfate reductase 3, chloroplastic	APR3	Reduces sulfate for Cys biosynthesis	*Increased Cys biosynthesis*	Tsakraklides, George, et al. "Sulfate reduction is increased in transgenic Arabidopsis thaliana expressing 5′-adenylylsulfate reductase from Pseudomonas aeruginosa." The Plant Journal 32.6 (2002): 879-889.
Solyc02g077110.3	2.45	phospholipase A1-II 1-like	OsI_03083	Acylhydrolase that catalyzes the hydrolysis of phospholipids at the sn-1 position; Involved in lipid catabolism	*Increased lipid catabolism*	Yu, Jun, et al. "The genomes of Oryza sativa: a history of duplications." PLoS Biol 3.2 (2005): e38.

TomatoID	DE in Infected	NCBI Protein Name	Gene ID	Uniprot Description	Putative Consequences for Infection	Citation
Solyc03g098720.3	2.43	kunitz-type serine protease inhibitor DrTI-like	N/A	Serine protease inhibitor that inhibits chymotripsin	*Increased protein degradation*	Chang, Long-sen, et al. "Purification and characterization of a chymotrypsin inhibitor from the venom of Ophiophagus hannah (King Cobra)." Biochemical and biophysical research communications 283.4 (2001): 862-867.
Solyc07g062700.3	2.43	sodium/calcium exchanger NCL	NCL	Participates in the maintenance of calcium homeostasis; Plays a role in auxin response, diurnal rhythm, and flowering time; Involved in salt stress response	*Promoted calcium homeostasis; Increased response to auxin; Promoted circadian rhythm; Promoted salt stress response*	Wang, Peng, et al. "A Na+/Ca2+ exchanger-like protein (AtNCL) involved in salt stress in Arabidopsis." Journal of Biological Chemistry 287.53 (2012): 44062-44070.
Solyc12g099260.2	2.43	ATP-citrate synthase beta chain protein 2-like	ACLA-2	Used for the elongation of fatty acids and biosynthesis of isoprenoids, flavonoids, and malonated derivatives; Required for normal growth and development in seeds	*Promoted seed development; Increased production of primary metabolites*	Fatland, Beth L., Basil J. Nikolau, and Eve Syrkin Wurtele. "Reverse genetic characterization of cytosolic acetyl-CoA generation by ATP-citrate lyase in Arabidopsis." The Plant Cell 17.1 (2005): 182-203.
Solyc10g075160.1	2.41	ferredoxin	AT1G32550	Ferredoxins are iron-sulfur proteins that transfer electrons in a wide variety of metabolic reactions	*Promoted metabolism*	Zapata, Luis, et al. "Chromosome-level assembly of Arabidopsis thaliana Ler reveals the extent of translocation and inversion polymorphisms." Proceedings of the National Academy of Sciences 113.28 (2016): E4052-E4060.
Solyc04g011840.1	2.41	monothiol glutaredoxin-S1-like	GRXS1	Reduces GSH-thiol disulfides	*Increased reduction of GSH-thiol disulfides*	Patterson, Kurt, et al. "Nitrate-regulated glutaredoxins control Arabidopsis primary root growth." Plant Physiology 170.2 (2016): 989-999.
Solyc05g056170.3	2.38	phenylalanine ammonia-lyase 2	PAL2	Enzyme of plant metabolism catalyzing the first reaction in the biosynthesis of a wide variety of natural products	*Increased biosynthesis of L-phenylalanine derivatives*	Cochrane, Fiona C., Laurence B. Davin, and Norman G. Lewis. "The Arabidopsis phenylalanine ammonia lyase gene family: kinetic characterization of the four PAL isoforms." Phytochemistry 65.11 (2004): 1557-1564.
Solyc07g043500.1	2.33	beta-D-glucosyl crocetin beta-1,6-glucosyltransferase	UGT94E5	Glucosyltransferase catalyzing crocetin gentiobiosyl esters biosynthesis	*Promoted production of crocetin and crocin; Increased pigment production*	Nagatoshi, Mai, et al. "UGT75L6 and UGT94E5 mediate sequential glucosylation of crocetin to crocin in Gardenia jasminoides." FEBS letters 586.7 (2012): 1055-1061.
Solyc03g120990.3	2.31	NADP-dependent malic enzyme, chloroplastic	ME6	Cecarboxylates malate shuttled from neighboring mesophyll cells; CO2 released is refixed by RuBisCo; Eliminates the photorespiratory loss of CO2 that occurs in most plants	*Promoted photorespiratory efficiency of CO2 loss*	Cheng, Yuxiang, et al. "Expression, purification, and characterization of two NADP-malic enzymes of rice (Oryza sativa L.) in Escherichia coli." Protein expression and purification 45.1 (2006): 200-205.
Solyc04g071890.3	2.30	peroxidase 12 precursor	PER12	Involved in removal of H2O2, oxidation of toxic reductants, biosynthesis and degradation of lignin, suberization, auxin catabolism, and response to environmental stressors	*Increased removal of H2O2, oxidation of toxic reductants, biosynthesis and degradation of lignin, suberization, auxin catabolism, and response to environmental stressors*	Paynel, Florence, et al. "Temporal regulation of cell-wall pectin methylesterase and peroxidase isoforms in cadmium-treated flax hypocotyl." Annals of botany 104.7 (2009): 1363-1372.

TomatoID	DE in Infected	NCBI Protein Name	Gene ID	Uniprot Description	Putative Consequences for Infection	Citation
Solyc04g011830.1	2.29	monothiol glutaredoxin-S1-like	GRXS1	Reduces GSH-thiol disulfides	*Increased reduction of GSH-thiol disulfides*	Patterson, Kurt, et al. "Nitrate-regulated glutaredoxins control Arabidopsis primary root growth." Plant Physiology 170.2 (2016): 989-999.
Solyc11g006910.2	2.28	ferredoxin, root R-B2	N/A	Ferredoxins are iron-sulfur proteins that transfer electrons in a wide variety of metabolic reactions	*Promoted metabolism*	Wada, Keishiro, Masaaki Onda, and Hiroshi Matsubara. "Amino acid sequences of ferredoxin isoproteins from radish roots." The Journal of Biochemistry 105.4 (1989): 619-625.
Solyc02g088630.3	2.27	probable galacturonosyltransferase 14	GAUT14	Involved in pectin and/or xylans biosynthesis in cell walls	*Increased pectin and xylan biosynthesis; Increased cell wall expansion*	Wang, Li, et al. "Arabidopsis galacturonosyltransferase (GAUT) 13 and GAUT14 have redundant functions in pollen tube growth." Molecular plant 6.4 (2013): 1131-1148.
Solyc07g049370.2	2.26	glucan endo-1,3-beta-glucosidase 12	AT4G29360	Involved in carbohydrate metabolic process, cell wall organization, and plant defense response	*Increased carbohydrate metabolism, cell wall organization, and plant defense response*	Wu, Qiong, et al. "Long-term balancing selection contributes to adaptation in Arabidopsis and its relatives." Genome biology 18.1 (2017): 1-15.
Solyc09g061890.3	2.20	probable pectate lyase 8	AT3G07010	Involved in pectin catabolic process	*Increased pectin catabolism*	Weber, Hans, et al. "Selective and powerful stress gene expression in Arabidopsis in response to malondialdehyde." The Plant Journal 37.6 (2004): 877-888.
Solyc02g071030.2	2.14	chlorophyll a-b binding protein 1B, chloroplastic	LHCB1.3	Light-harvesting complex functions as a light receptor; Captures and delivers excitation energy to photosystems	*Promoted light capture and photosynthesis*	Yadav, Vandana, et al. "Light regulated modulation of Z-box containing promoters by photoreceptors and downstream regulatory components, COP1 and HY5, in Arabidopsis." The Plant Journal 31.6 (2002): 741-753.
Solyc07g052510.4	2.13	peroxidase 3 precursor	PER3	Involved in removal of H2O2, oxidation of toxic reductants, biosynthesis and degradation of lignin, suberization, auxin catabolism, response to environmental stressors	*Increased removal of H2O2, oxidation of toxic reductants, biosynthesis and degradation of lignin, suberization, auxin catabolism, and response to environmental stressors*	Paynel, Florence, et al. "Temporal regulation of cell-wall pectin methylesterase and peroxidase isoforms in cadmium-treated flax hypocotyl." Annals of botany 104.7 (2009): 1363-1372.
Solyc06g083650.3	2.12	GDSL esterase/lipase At5g33370	AT5G33370	Involved in cuticle development; Involved in lipid catabolism	*Increased cuticle development; Increased lipid catabolism*	Li-Beisson, Yonghua, et al. "Acyl-lipid metabolism." The Arabidopsis book/American Society of Plant Biologists 11 (2013).
Solyc09g065620.3	2.12	chlorophyllase-2, chloroplastic isoform X4	CLH2	Catalyzes the hydrolysis of ester bond in chlorophyll to yield chlorophyllide and phytol	*Increased chlorophyllide and phytol photosynthesis; Promoted photosynthesis*	Tsuchiya, Tohru, et al. "Cloning of chlorophyllase, the key enzyme in chlorophyll degradation: finding of a lipase motif and the induction by methyl jasmonate." Proceedings of the National Academy of Sciences 96.26 (1999): 15362-15367.
Solyc12g014420.2	2.12	glucan endo-1,3-beta-glucosidase 13-like	AT5G56590	Involved in carbohydrate metabolic process, cell wall organization, and plant defense response	*Increased carbohydrate metabolism, cell wall organization, and plant defense response*	Wu, Qiong, et al. "Long-term balancing selection contributes to adaptation in Arabidopsis and its relatives." Genome biology 18.1 (2017): 1-15.
Solyc06g053640.1	2.08	RING-H2 finger protein ATL16-like	ATL16	Involved in protein ubiquitination	*Promoted protein ubiquitination*	Thieme, Christoph J., et al. "Endogenous Arabidopsis messenger RNAs transported to distant tissues." Nature Plants 1.4 (2015): 1-9.
Solyc05g005160.3	2.07	ATP-citrate synthase alpha chain protein 2	ACLA-2	ATP citrate-lyase is the primary enzyme responsible for the synthesis of cytosolic acetyl-CoA; Required for normal growth and development in seeds	*Promoted seed development; Increased production of primary metabolites*	Fatland, Beth L., Basil J. Nikolau, and Eve Syrkin Wurtele. "Reverse genetic characterization of cytosolic acetyl-CoA generation by ATP-citrate lyase in Arabidopsis." The Plant Cell 17.1 (2005): 182-203.

TomatoID	DE in Infected	NCBI Protein Name	Gene ID	Uniprot Description	Putative Consequences for Infection	Citation
Solyc08g083370.3	2.06	soluble inorganic pyrophosphatase PPA1-like isoform X1	PPA1	Controls the equilibrium of gluconeogenic reactions in the heterotrophic growth phase of early seedling establishment	*Promoted equilibriation gluconeogenic reactions*	Navarro-De la Sancha, Ernesto, et al. "Characterization of two soluble inorganic pyrophosphatases from Arabidopsis thaliana." Plant science 172.4 (2007): 796-807.
Solyc12g040570.1	2.05	6-phosphogluconate dehydrogenase, decarboxylating 3	PGD3	Essential component in cell redox homeostasis	*Increased cell redox homeostasis*	Fernández-Fernández, Álvaro D., and Francisco J. Corpas. "In silico analysis of Arabidopsis thaliana peroxisomal 6-phosphogluconate dehydrogenase." Scientifica 2016 (2016).
Solyc09g008280.2	2.04	S-adenosylmethionine synthase 3	METK3	Catalyzes the formation of S-adenosylmethionine from methionine; Involved in SA-mediated defense; Involved in the biosynthesis of lignin	*Increased S-adenosylmethionine production; Promoted early defense response; Increased lignin biosynthesis*	Goto, Derek B., et al. "A single-nucleotide mutation in a gene encoding S-adenosylmethionine synthetase is associated with methionine over-accumulation phenotype in Arabidopsis thaliana." Genes & genetic systems 77.2 (2002): 89-95.
Solyc07g017600.3	2.04	pectinesterase/pectinesterase inhibitor PPE8B	N/A	Plays roles in the deposition of pectin in developing tissues and in the wall loosening and cell separation that occurs in cell expansion, fruit ripening and abscission.	*Increased pectin deposition and plant cell wall loosening*	Glover, H., et al. "Multiple pectin esterase genes are expressed in ripening peach fruit: nucleotide sequence of a cDNA encoding peach pectin esterase." Plant Physiology 112 (1996): 864-864.
Solyc02g093230.3	2.03	caffeoyl-CoA O-methyltransferase 1	CCOAOMT1	Involved in the reinforcement of the plant cell wall; Involved in response to wounding or pathogen challenge by promoting the formation of cell wall-bound ferulic acid polymers	*Increased production of feruloylated polysaccharides; Promoted plant cell wall development and integrity*	Meyermans, Hugo, et al. "Modifications in lignin and accumulation of phenolic glucosides in poplar xylem upon down-regulation of caffeoyl-coenzyme A O-methyltransferase, an enzyme involved in lignin biosynthesis." Journal of Biological Chemistry 275.47 (2000): 36899-36909.
Solyc10g050160.2	2.02	caffeoyl-CoA O-methyltransferase 5	CCOAOMT5	Plays a role in the synthesis of feruloylated polysaccharides; Involved in the reinforcement of the plant cell wall; Involved in response to wounding or pathogen challenge	*Increased production of feruloylated polysaccharides; Promoted plant cell wall development and integrity*	Meyermans, Hugo, et al. "Modifications in lignin and accumulation of phenolic glucosides in poplar xylem upon down-regulation of caffeoyl-coenzyme A O-methyltransferase, an enzyme involved in lignin biosynthesis." Journal of Biological Chemistry 275.47 (2000): 36899-36909.
Solyc07g007590.1	2.01	arogenate dehydrogenase 2, chloroplastic-like	TYRAAT2	Involved in the biosynthesis of tyrosine	*Increased production of tyrosine biosynthesis*	Rippert, Pascal, and Michel Matringe. "Purification and kinetic analysis of the two recombinant arogenate dehydrogenase isoforms of Arabidopsis thaliana." European Journal of Biochemistry 269.19 (2002): 4753-4761.
Solyc02g082260.3	2.00	3-hydroxy-3-methylglutaryl-coenzyme A reductase 1	HMG1	Catalyzes the synthesis of mevalonate, the specific precursor of all isoprenoid compounds present in plants	*Increased synthesis of isoprenoids*	Dale, Susan, et al. "Bacterial expression of the catalytic domain of 3–hydroxy-3–methylglutaryl-coa reductase (isoform HMGR1) from Arabidopsis thaliana, and Its inactivation by phosphorylation at Ser577 by Brassica oleracea 3-hydroxy-3-methylglutaryl-CoA reductase kinase." European Journal of Biochemistry 233.2 (1995): 506-513.
Solyc12g096490.1	-2.05	protein GLUTAMINE DUMPER 5-like	GDU5	Involved in the regulation of the amino acid metabolism	Impaired amino acid tranport; Impaired regulation of amino acid metabolism	Pratelli, Réjane, et al. "Stimulation of nonselective amino acid export by glutamine dumper proteins." Plant Physiology 152.2 (2010): 762-773.

TomatoID	DE in Infected	NCBI Protein Name	Gene ID	Uniprot Description	Putative Consequences for Infection	Citation
Solyc00g203660.2	-2.09	ribulose-1,5-bisphosphate carboxylase/oxygenase large subunit	rbcL	RuBisCO catalyzes two reactions: Participates in the the carboxylation of D-ribulose 1,5-bisphosphate of RuBisCO	Decreased RuBisCo catalysis	Chun, Louis, Alana Kawakami, and David A. Christopher. "Phytochrome A mediates blue light and UV-A-dependent chloroplast gene transcription in green leaves." Plant Physiology 125.4 (2001): 1957-1966.
Solyc04g080040.3	-2.22	J domain-containing protein required for chloroplast accumulation response 1	JAC1	Required for chloroplast photorelocation movement	Impaired photoperiodism	Suetsugu, Noriyuki, Takatoshi Kagawa, and Masamitsu Wada. "An auxilin-like J-domain protein, JAC1, regulates phototropin-mediated chloroplast movement in Arabidopsis." Plant Physiology 139.1 (2005): 151-162.
Solyc10g005080.3	-2.27	protein LHY isoform X2	LHY	Transcription factor involved in the circadian clock; Represses CCA1	Impaired circadian rhythm; Impaired regulation of CCA1	Mizoguchi, Tsuyoshi, et al. "LHY and CCA1 are partially redundant genes required to maintain circadian rhythms in Arabidopsis." Developmental cell 2.5 (2002): 629-641.
Solyc11g066510.2	-2.29	RING-H2 finger protein ATL57	ATL57	Involved in the pathway protein ubiquitination, which is part of protein modification	Impaired protein ubiquitination	Thieme, Christoph J., et al. "Endogenous Arabidopsis messenger RNAs transported to distant tissues." Nature Plants 1.4 (2015): 1-9.
Solyc12g005750.1	-2.29	B-box domain protein 30	MIP1A	Developmental regulator; Involved in the CO-mediated long-day flowering-promotion pathway	Decreased sequestration of CO and CO-like proteins; Impaired flowering	Graeff, Moritz, et al. "MicroProtein-mediated recruitment of CONSTANS into a TOPLESS trimeric complex represses flowering in Arabidopsis." PLoS genetics 12.3 (2016): e1005959.
Solyc01g110360.3	-2.30	fructose-bisphosphate aldolase 1	FBA1	Plays a key role in glycolysis and gluconeogenesis	Impaired glycolysis and glucogenesis	Searle, Iain R., et al. "Long-distance signaling in nodulation directed by a CLAVATA1-like receptor kinase." Science 299.5603 (2003): 109-112.
Solyc07g005210.3	-2.33	temperature-induced lipocalin-1	TIL	Involved in basal and acquired thermotolerance; Lipocalin that confers protection against oxidative stress caused by heat, hypersalinity, freezing, paraquat, and light	Impaired thermotolerance; Impaired tolerance to salt stress, freezing, paraquat, and light	Abo-Ogiala, Atef, et al. "Temperature-induced lipocalin (TIL) is translocated under salt stress and protects chloroplasts from ion toxicity." Journal of plant physiology 171.3-4 (2014): 250-259.
Solyc11g005290.1	-2.40	RING-H2 finger protein ATL78-like	ATL78	Involved in the pathway protein ubiquitination, which is part of protein modification	Impaired protein ubiquitination	Thieme, Christoph J., et al. "Endogenous Arabidopsis messenger RNAs transported to distant tissues." Nature Plants 1.4 (2015): 1-9.
Solyc07g006600.1	-2.42	protein GLUTAMINE DUMPER 2	GDU2	Subunit of an amino acid transporter involved in the regulation of the amino acid metabolism	Impaired regulation of amino acid metabolism	Pratelli, Réjane, et al. "Stimulation of nonselective amino acid export by glutamine dumper proteins." Plant Physiology 152.2 (2010): 762-773.
Solyc12g099200.2	-2.47	cell wall invertase inhibitor precursor	LOC107808322	Enzyme inhibitor activity	Decreased enzyme inhibition	Hothorn, Michael, and Klaus Scheffzek. "Multiple crystal forms of the cell-wall invertase inhibitor from tobacco support high conformational rigidity over a broad pH range." Acta Crystallographica Section D: Biological Crystallography 62.6 (2006): 665-670.

TomatoID	DE in Infected	NCBI Protein Name	Gene ID	Uniprot Description	Putative Consequences for Infection	Citation
Solyc02g065220.3	-2.56	geraniol 8-hydroxylase-like	CYP76B6	Hydroxylase involved in the biosynthesis of hydroxygeraniol, a precursor of the	Decreased biosynthesis of hydroxygeraniol	Collu, Graziella, et al. "Activity of the cytochrome P450 enzyme geraniol 10-hydroxylase and alkaloid production in plant cell cultures." Plant Science 162.1 (2002): 165-
Solyc01g080460.3	-2.61	pyruvate, phosphate dikinase, chloroplastic	PPDK	Formation of phosphoenolpyruvate, which is the primary acceptor of CO2 in C4 and Crassulacean plants	Impaired primary metabolism	Rosche, Elke, and Peter Westhoff. "Primary structure of pyruvate, orthophosphate dikinase in the dicotyledonous C4 plant Flaveria trinervia." FEBS letters 273.1-2 (1990): 116-121.
Solyc12g087870.2	-2.62	purine permease 3-like	PUP3	Involved in transport of purine derivatives during pollen germination and tube elongation	Impaired transport of purine derivatives during pollen germination and tube elongation	Bürkle, Lukas, et al. "Transport of cytokinins mediated by purine transporters of the PUP family expressed in phloem, hydathodes, and pollen of Arabidopsis." The Plant Journal 34.1 (2003): 13-26.
Solyc03g115920.3	-2.77	probable E3 ubiquitin-protein ligase RNF217	RNF217	E3 ubiquitin-protein ligase which accepts ubiquitin from E2 ubiquitin-conjugating enzymes in the form of a thioester	Impaired primary metabolism	Ohmura-Hoshino, Mari, et al. "A novel family of membrane-bound E3 ubiquitin ligases." Journal of biochemistry 140.2 (2006): 147-154.
Solyc09g010530.3	-3.57	cation/H(+) antiporter 20	CHX20	Operates as a K+/H+ antiporter that maintains K+ homeostasis in guard cells; Helps regulate pH; Plays a critical role in osmoregulation through via stomatal opening	Impaired K+ homeostasis and pH homeostasis; Impaired osmoregulation	Padmanaban, Senthilkumar, et al. "Participation of endomembrane cation/H+ exchanger AtCHX20 in osmoregulation of guard cells." Plant Physiology 144.1 (2007): 82-93.
Solyc09g065790.1	-5.14	clp protease proteolytic subunit	CLPR2	Required for chloroplast development and integrity; Involved in the regulation of plastoglobules formation	Impaired chloroplast development and integrity; Impaired regulation of plastoglobule formation	Rudella, Andrea, et al. "Downregulation of ClpR2 leads to reduced accumulation of the ClpPRS protease complex and defects in chloroplast biogenesis in Arabidopsis." The Plant Cell 18.7 (2006): 1704-1721.
Solyc09g064580.2	-5.99	photosystem II protein M	psbM	Component of the core complex of photosystem II; Light-driven water:plastoquinone oxidoreductase that uses light energy to abstract electrons from H2O	Impaired light capture and photosynthesis	Kawakami, Keisuke, et al. "Location of chloride and its possible functions in oxygen-evolving photosystem II revealed by X-ray crystallography." Proceedings of the National Academy of Sciences 106.21 (2009): 8567-8572.
Solyc01g017220.1	-6.05	ATP synthase CF1 epsilon subunit	atpE	Produces ATP from ADP in the presence of a proton gradient across the membrane	Impaired ATP production	Goulas, Estelle, et al. "The chloroplast lumen and stromal proteomes of Arabidopsis thaliana show differential sensitivity to short-and long-term exposure to low temperature." The Plant Journal 47.5 (2006): 720-734.
Solyc04g039850.1	-6.40	ATP synthase beta subunit	atpB	Produces ATP from ADP in the presence of a proton gradient across the membrane	Impaired ATP production	Goulas, Estelle, et al. "The chloroplast lumen and stromal proteomes of Arabidopsis thaliana show differential sensitivity to short-and long-term exposure to low temperature." The Plant Journal 47.5 (2006): 720-734.
