# Supplementary material for: Effects of ‘Candidatus Liberibacter solanacearum’ haplotypes A and B on tomato gene expression and geotropism
Source: BMC Plant Biol. 2022 Mar 30;22:156. doi: 10.1186/s12870-022-03505-z (PMC8966271; doi:10.1186/s12870-022-03505-z)
Supplement: Supplementary file 8 — Additional file 8: Table S8. The 84 tomato plant DEGs associated with plant signaling and transport under Lso infection. DEGs were sorted by differential expression (DE) values comparing uninfected to infected samples (p-value < 0.05). NCBI Blast searches were used to identify Gene IDs and protein products in tomatoes as well as their homologs in other species. Specifically, the expression changes in 45 of these genes (54%, in italics) would have resulted in impaired signaling transduction or transport in infected plants. These DEGs were related to chloroplastic import, metal ion signaling and transport, abscisic acid signaling and transport, auxin-responsive signaling, and PAMP-induced signaling. [file 12870_2022_3505_MOESM8_ESM.pdf]

TomatoID	DE in Infected	NCBI Protein Name	Gene ID	Uniprot Description	Putative Consequences for Infection	Citation
Solyc07g008570.3	6.14	probable inactive purple acid phosphatase 27	PAP27	Acid phosphatase activity; Metal ion binding	N/A	Cheng, Chia-Yi, et al. "Araport11: a complete reannotation of the Arabidopsis thaliana reference genome." The Plant Journal 89.4 (2017): 789-804.
Solyc12g009220.2	4.88	jasmonate ZIM-domain protein 2	NtJAZ2	Involved in regulation of defense response; Involved in regulation of JA-mediated signaling pathway; Involved in response to wounding	Increased regulation of defense response; Increased regulation of JA-mediated signaling; Increased response to wounding	Shoji, Tsubasa, Takayuki Ogawa, and Takashi Hashimoto. "Jasmonate-induced nicotine formation in tobacco is mediated by tobacco COI1 and JAZ genes." Plant and cell physiology 49.7 (2008): 1003-1012.
Solyc06g076020.3	4.79	heat shock cognate 70 kDa protein 1	HSP70-1	Facilitates folding of de novo proteins, assists translocation of precursor proteins into organelles; Degrades damaged protein under stress conditions; Involved in defense response; Involved in protein transport to chloroplasts; Modulates stomatal aperture in response to environmental conditions	Increased protein folding; Increased translocation of presursor proteins into organelles; Increased degradation of damaged proteins; Promoted defense response; Increased response to abiotic stress and ABA signaling	Sung, Dong Yul, and Charles L. Guy. "Physiological and molecular assessment of altered expression of Hsc70-1 in Arabidopsis. Evidence for pleiotropic consequences." Plant Physiology 132.2 (2003): 979-987.
Solyc11g071740.2	4.64	putative calcium-binding protein CML19	CML19	Calcium sensor	Promoted calcium sensing	Boonburapong, Bongkoj, and Teerapong Buaboocha. "Genome-wide identification and analyses of the rice calmodulin and related potential calcium sensor proteins." BMC plant biology 7.1 (2007): 4.
Solyc06g051940.3	4.04	protein phosphatase 2C	PP2CA	Major negative regulator of ABA responses during seed germination and cold acclimation; Prevents stomata closure	*Impaired ABA responses; Promoted stomatal opening*	Chérel, Isabelle, et al. "Physical and functional interaction of the Arabidopsis K+ channel AKT2 and phosphatase AtPP2CA." The Plant Cell 14.5 (2002): 1133-1146.
Solyc01g007030.3	3.99	E3 ubiquitin-protein ligase PUB22-like	PUB22	Negatively regulates water stress response; Mediates drought signaling pathway; Negative regulator of immune response triggered by PAMPs	*Impaired response to drought; Impaired response to PAMPs; Impaired attenuation to PAMP-induced signaling*	Trujillo, Marco, et al. "Negative regulation of PAMP-triggered immunity by an E3 ubiquitin ligase triplet in Arabidopsis." Current Biology 18.18 (2008): 1396-1401.
Solyc03g013160.3	3.99	amino acid transporter AVT1I	AVT1I	Amino acid transporter	Increased amino acid transport	Fujiki, Yuki, et al. "Functional identification of AtAVT3, a family of vacuolar amino acid transporters, in Arabidopsis." FEBS letters 591.1 (2017): 5-15.
Solyc06g073830.1	3.69	putative calcium-binding protein CML19	CML19	Calcium sensor	Promoted calcium sensing	Boonburapong, Bongkoj, and Teerapong Buaboocha. "Genome-wide identification and analyses of the rice calmodulin and related potential calcium sensor proteins." BMC plant biology 7.1 (2007): 4.
Solyc01g096670.3	3.64	cytochrome P450 98A2	CYP98A2	Heme binding, iron ion binding, oxidoreductase activity, acting on paired donors	N/A	N/A
Solyc01g007040.3	3.63	E3 ubiquitin-protein ligase PUB22-like	PUB22	Negatively regulates water stress response; Mediates drought signaling pathway; Negative regulator of immune response triggered by PAMPs	*Impaired response to drought; Impaired response to PAMPs; Impaired attenuation to PAMP-induced signaling*	Trujillo, Marco, et al. "Negative regulation of PAMP-triggered immunity by an E3 ubiquitin ligase triplet in Arabidopsis." Current Biology 18.18 (2008): 1396-1401.

TomatoID	DE in Infected	NCBI Protein Name	Gene ID	Uniprot Description	Putative Consequences for Infection	Citation
Solyc11g010170.2	3.58	lanC-like protein GCL1	GCL1	Plays a role in signaling	N/A	Gao, Yajun, et al. "Genetic characterization reveals no role for the reported ABA receptor, GCR2, in ABA control of seed germination and early seedling development in Arabidopsis." The Plant Journal 52.6 (2007): 1001-1013.
Solyc04g082140.3	3.52	multicopper oxidase-like protein precursor	LPR1	Multicopper oxidase involved in Cu homeostasis and oxidative stress response; Necessary for root growth inhibition	Promoted copper homeostasis; Increased response to oxidative stress; Root growth inhibition	Svistoonoff, Sergio, et al. "Root tip contact with low-phosphate media reprograms plant root architecture." Nature genetics 39.6 (2007): 792-796.
Solyc00g095860.1	3.51	1-aminocyclopropane-1-carboxylate synthase-like	ACS1	Involved in ethylene biosynthesis	Increased ethylene biosynthesis; Increased ethylene-related signaling	Tsuchisaka, Atsunari, et al. "A combinatorial interplay among the 1-aminocyclopropane-1-carboxylate isoforms regulates ethylene biosynthesis in Arabidopsis thaliana." Genetics 183.3 (2009): 979-1003.
Solyc08g081550.3	3.14	1-aminocyclopropane-1-carboxylate synthase	ACS1	Involved in ethylene biosynthesis	Increased ethylene biosynthesis	Tsuchisaka, Atsunari, et al. "A combinatorial interplay among the 1-aminocyclopropane-1-carboxylate isoforms regulates ethylene biosynthesis in Arabidopsis thaliana." Genetics 183.3 (2009): 979-1003.
Solyc08g077020.1	3.04	auxin-responsive protein SAUR76	SAUR76	Involved in the regulation of ethylene receptor signaling; Promotes cell expansion and plant growth; Involved in the regulation of cell elongation	Increased ethylene receptor signaling; Promoted cell expansion and plant growth/development; Leaf curl	Markakis, Marios Nektarios, et al. "Characterization of a small auxin-up RNA (SAUR)-like gene involved in Arabidopsis thaliana development." PloS one 8.11 (2013): e82596.
Solyc01g097520.3	3.03	annexin D4-like	ANN4	Involved in osmotic stress response and ABA signaling	Increased osmostic stress and ABA signaling	Lee, Sumin, et al. "Proteomic identification of annexins, calcium-dependent membrane binding proteins that mediate osmotic stress and abscisic acid signal transduction in Arabidopsis." The Plant Cell 16.6 (2004): 1378-1391.
Solyc03g096290.3	2.99	aquaporin PIP1-7	PIP1-1	Water channel required to facilitate the transport of water across cell membrane	Increased water transport	Marmagne, Anne, et al. "Identification of new intrinsic proteins in Arabidopsis plasma membrane proteome." Molecular & Cellular Proteomics 3.7 (2004): 675-691.
Solyc01g007020.3	2.92	E3 ubiquitin-protein ligase PUB22-like	PUB22	Negatively regulates water stress response; Mediates drought signaling pathway; Negative regulator of immune response triggered by PAMPs	*Impaired response to drought; Impaired response to PAMPs; Impaired attenuation to PAMP-induced signaling*	Trujillo, Marco, et al. "Negative regulation of PAMP-triggered immunity by an E3 ubiquitin ligase triplet in Arabidopsis." Current Biology 18.18 (2008): 1396-1401.
Solyc04g071770.3	2.79	ethylene-responsive transcription factor ABR1-like	ABR1	Negative regulator of the ABA signaling pathway involved in seed germination and in response to stressors	Increased ABA signaling; Increased response to stress and pathogenesis	Pandey, Girdhar K., et al. "ABR1, an APETALA2-domain transcription factor that functions as a repressor of ABA response in Arabidopsis." Plant Physiology 139.3 (2005): 1185-1193.
Solyc03g093610.1	2.77	ethylene response factor A.2	ERF2	Involved in defense response; Involved in intracellular signal transduction; Involved in lipid metabolism	Increased defense response; Increased intracellular signal transduction; Increased lipid metabolism	Zhang, Zhijin, et al. "Transcriptional regulation of the ethylene response factor LeERF2 in the expression of ethylene biosynthesis genes controls ethylene production in tomato and tobacco." Plant Physiology 150.1 (2009): 365-377.

TomatoID	DE in Infected	NCBI Protein Name	Gene ID	Uniprot Description	Putative Consequences for Infection	Citation
Solyc04g079360.1	2.77	transcription factor MYB44-like	MYB44	Represses the expression of protein phosphatases 2C in response to ABA; Auxin-responsive; Promotes SA-mediated defense, but represses JA-mediated defense	Increases response to ABA; Increased response to auxin signaling; Increases SA-mediated defense; Decreased JA-mediated defense	Riechmann, José Luis, et al. "Arabidopsis transcription factors: genome-wide comparative analysis among eukaryotes." Science 290.5499 (2000): 2105-2110.
Solyc07g008560.3	2.72	probable inactive purple acid phosphatase 27	PAP27	Acid phosphatase activity; Metal ion binding	N/A	Cheng, Chia-Yi, et al. "Araport11: a complete reannotation of the Arabidopsis thaliana reference genome." The Plant Journal 89.4 (2017): 789-804.
Solyc04g009440.3	2.68	NAC domain protein	NAC002	Involved in cellular response to hypoxia; Involved in regulation of ABA-activated signaling pathway; Involved in response to wounding	Increased cellular response to hypoxia; Increased in regulation of ABA-activated signaling pathway; Increased in response to wounding	"Molecular characterization of Brassica napus NAC domain transcriptional activators induced in response to biotic and abiotic stress."
Solyc02g064680.3	2.67	calcium-transporting ATPase 2, plasma membrane-type-like	ACA2	Catalyzes the hydrolysis of ATP coupled with the translocation of calcium from the cytosol into the endoplasmic reticulum	Increased ATP-dependent translocation of calcium into the endoplasmic reticulum	Harper, Jeffrey F., et al. "A novel calmodulin-regulated Ca2+-ATPase (ACA2) from Arabidopsis with an N-terminal autoinhibitory domain." Journal of Biological Chemistry 273.2 (1998): 1099-1106.
Solyc03g122190.3	2.66	salt responsive protein 1	SlSRG1	Involved in regulation of defense; Involved in regulation of JA-mediated signaling pathway	Promoted regulation of defense; Promoted regulation of JA-mediated signaling pathway	Ouyang, Bo, et al. "Identification of early salt stress response genes in tomato root by suppression subtractive hybridization and microarray analysis." Journal of experimental botany 58.3 (2007): 507-520.
Solyc02g080190.3	2.63	nuclear transport factor 2B	NTF2B	Part of a multicomponent system of cytosolic factors that assemble at the pore complex during nuclear import	Increased protein transport into the nucleus	Zhao, Qiao, et al. "Identification and characterization of the Arabidopsis orthologs of nuclear transport factor 2, the nuclear import factor of ran." Plant physiology 140.3 (2006): 869-878.
Solyc03g007230.3	2.56	protein phosphatase 2C 51-like	AT3G63340	Metal ion binding; Protein serine/threonine phosphatase activity	N/A	Cheng, Chia-Yi, et al. "Araport11: a complete reannotation of the Arabidopsis thaliana reference genome." The Plant Journal 89.4 (2017): 789-804.
Solyc02g083850.3	2.54	calcium-dependent protein kinase 18-like	CPK18	Plays a role in signal transduction pathways that involve calcium as a second messenger	Promoted calcium-dependent signaling	Cheng, Shu-Hua, et al. "Calcium signaling through protein kinases. The Arabidopsis calcium-dependent protein kinase gene family." Plant physiology 129.2 (2002): 469-485.
Solyc07g008550.3	2.51	probable inactive purple acid phosphatase 27	PAP27	Acid phosphatase activity; Metal ion binding	N/A	Cheng, Chia-Yi, et al. "Araport11: a complete reannotation of the Arabidopsis thaliana reference genome." The Plant Journal 89.4 (2017): 789-804.
Solyc06g082440.1	2.45	CBL-interacting protein kinase 11	CIPK11	Acts as a negative regulator of the plasma membrane proton pump AHA2 by preventing its interaction with 14-3-3 protein	Increased activity of plasma membrane proton pump	Fuglsang, Anja T., et al. "Arabidopsis protein kinase PKS5 inhibits the plasma membrane H+-ATPase by preventing interaction with 14-3-3 protein." The Plant Cell 19.5 (2007): 1617-1634.
Solyc10g006700.1	2.39	calcium-binding protein PBP1	PBP1	Potential calcium sensor	Promoted calcium sensing; Promoted regulation of trichome morphogenesis	Reddy, Vaka S., et al. "KIC, a novel Ca2+ binding protein with one EF-hand motif, interacts with a microtubule motor protein and regulates trichome morphogenesis." The Plant Cell 16.1 (2004): 185-200.

TomatoID	DE in Infected	NCBI Protein Name	Gene ID	Uniprot Description	Putative Consequences for Infection	Citation
Solyc12g088190.2	2.39	amino acid permease 6	AAP6	Involved in the uptake of amino acids diffusing out of the xylem tracheids into the xylem parenchyma	Increased amino acid uptake and mobilization	Okumoto, Sakiko, et al. "High affinity amino acid transporters specifically expressed in xylem parenchyma and developing seeds of Arabidopsis." Journal of Biological Chemistry 277.47 (2002): 45338-45346.
Solyc03g115380.2	2.28	UDP-glucose 6-dehydrogenase 1-like	UGD1	Involved in the biosynthesis of UDP-glucuronic acid, providing nucleotide sugars for cell-wall polymers	Increased nucleotide transport for cell-wall polymers	Oka, Takuji, and Yoshifumi Jigami. "Reconstruction of de novo pathway for synthesis of UDP-glucuronic acid and UDP-xylose from intrinsic UDP-glucose in Saccharomyces cerevisiae." The FEBS journal 273.12 (2006): 2645-2657.
Solyc09g064820.1	2.26	EID1-like F-box protein 3	EDL3	Involved in the following processes: ABA-activated signaling pathway, regulation of seed germination, response to osmotic stress, response to salt stress, response to water deprivation, and meristem phase transition	Increased ABA-activated signaling; Increased regulation of seed germination; Promoted response to osmotic, salt, and water stress; Promoted transition from vegetative to reproductive phase in the meristem	Friso, Giulia, et al. "In-depth analysis of the thylakoid membrane proteome of Arabidopsis thaliana chloroplasts: new proteins, new functions, and a plastid proteome database." The Plant Cell 16.2 (2004): 478-499.
Solyc07g053230.3	2.22	transcription factor MYB15-like	MYB15	Transcription factor involved in cold-regulation of CBF genes and in freezing tolerance; Involved in drought and salt tolerance; Enhances ABA biosynthesis and signaling	Promoted freezing, drought, and salt tolerance; Enchanced ABA biosynthesis and signlaing	Agarwal, Manu, et al. "A R2R3 type MYB transcription factor is involved in the cold regulation of CBF genes and in acquired freezing tolerance." Journal of Biological Chemistry 281.49 (2006): 37636-37645.
Solyc02g088090.1	2.16	probable calcium-binding protein CML30	CML30	Calcium sensor	Promoted calcium sensing	Boonburapong, Bongkoj, and Teerapong Buaboocha. "Genome-wide identification and analyses of the rice calmodulin and related potential calcium sensor proteins." BMC plant biology 7.1 (2007): 4.
Solyc03g082660.3	2.14	molybdate-anion transporter	MFSD5	Mediates high-affinity intracellular uptake of the rare oligo-element molybdenum	Increased intracellular uptake of molybdenum	Tejada-Jiménez, Manuel, Aurora Galván, and Emilio Fernández. "Algae and humans share a molybdate transporter." Proceedings of the National Academy of Sciences 108.16 (2011): 6420-6425.
Solyc04g081530.1	2.14	DNAJ-like protein	T5J17.130	ATP binding, heat shock protein binding, metal ion binding, unfolding protein binding	N/A	Mayer, Klauss, et al. "Sequence and analysis of chromosome 4 of the plant Arabidopsis thaliana." Nature 402.6763 (1999): 769-777.
Solyc03g116070.1	2.12	mini zinc finger protein 3	MIF3	Involved in integrating signals from multiple hormones by regulating the expression of specific genes; Promotes the formation of ectopic shoot meristems	Increased hormonal signaling; Promoted formation of ectopic shoot meristems	Hu, Wei, Baomin Feng, and Hong Ma. "Ectopic expression of the Arabidopsis MINI ZINC FINGER1 and MIF3 genes induces shoot meristems on leaf margins." Plant molecular biology 76.1-2 (2011): 57-68.
Solyc04g071400.3	2.12	WAT1-related protein At1g43650-like isoform X2	AT1G43650	Transmembrane transporter activity	Increased transmembrane transporter	Busov, Victor B., et al. "An auxin-inducible gene from loblolly pine (Pinus taeda L.) is differentially expressed in mature and juvenile-phase shoots and encodes a putative transmembrane protein." Planta 218.6 (2004): 916-927.

TomatoID	DE in Infected	NCBI Protein Name	Gene ID	Uniprot Description	Putative Consequences for Infection	Citation
Solyc04g051360.3	2.09	ethylene-responsive transcription factor ABR1-like	ABR1	Negative regulator of the ABA signaling pathway involved in seed germination and in responses to stress	Increased ABA signaling; Increased response to stress and pathogenesis	Pandey, Girdhar K., et al. "ABR1, an APETALA2-domain transcription factor that functions as a repressor of ABA response in Arabidopsis." Plant Physiology 139.3 (2005): 1185-1193.
Solyc04g076570.3	2.08	protein CLT2, chloroplastic isoform X3	CLT2	Involved in thiol transport from the plastid to the cytosol; Transports glutathione and its precursor	Increased thiol transport; Increased transport of glutathione and its presursor	Maughan, Spencer C., et al. "Plant homologs of the Plasmodium falciparum chloroquine-resistance transporter, PfCRT, are required for glutathione homeostasis and stress responses." Proceedings of the National Academy of Sciences 107.5 (2010): 2331-2336.
Solyc07g007870.3	2.06	12-oxophytodienoate reductase 3	OPR3	Involved in the biosynthesis of JA and other oxylipin signaling moleclules; Required for the spatial and temporal regulation of JA levels during dehiscence of anthers	Increased biosynthesis of JA and other oxylipin signaling molecules	Sanders, Paul M., et al. "The Arabidopsis DELAYED DEHISCENCE1 gene encodes an enzyme in the jasmonic acid synthesis pathway." The Plant Cell 12.7 (2000): 1041-1061.
Solyc10g006660.3	2.06	calcium-binding protein KRP1	KRP1	Potential calcium sensor	Promoted calcium sensing; Promoted regulation of trichome morphogenesis	Reddy, Vaka S., et al. "KIC, a novel Ca2+ binding protein with one EF-hand motif, interacts with a microtubule motor protein and regulates trichome morphogenesis." The Plant Cell 16.1 (2004): 185-200.
Solyc09g089930.2	2.04	ethylene responsive element binding protein	EREBP1	Involved in defense response; Involved in ethylene-activated signaling pathway	Increased defense response; Increased ethylene-responsive signaling	Horvath, Diana M., Dorothy J. Huang, and Nam-Hai Chua. "Four classes of salicylate-induced tobacco genes." Molecular plant-microbe interactions 11.9 (1998): 895-905.
Solyc12g036470.2	2.01	transcription factor BEE 1-like	BEE1	Positive regulator of brassinosteroid signaling	Promoted brassinosteroid signaling	Friedrichsen, Danielle M., et al. "Three redundant brassinosteroid early response genes encode putative bHLH transcription factors required for normal growth." Genetics 162.3 (2002): 1445-1456.
Solyc01g102640.2	-2.00	nuclear pore complex protein NUP98A-like	NUP98A	Structural constituent of nuclear pore; Necessary for mRNA transport from the nucleus and protein import into the nucleus; Required for photoperiodism; Required for constitutive defense	*Impaired mRNA transport; Impaired photoperiodism; Impaired constitutive defense*	"A putative nucleoporin 96 is required for both basal defense and constitutive resistance responses mediated by suppressor of npr1-1,constitutive 1."
Solyc03g097930.3	-2.03	potassium channel SKOR-like	SKOR	Involved in potassium release into the xylem sap toward the shoots; Interacts with the cytoskeleton or with regulatory proteins	*Impaired potassium transport*	Gaymard, Frédéric, et al. "Identification and disruption of a plant shaker-like outward channel involved in K+ release into the xylem sap." Cell 94.5 (1998): 647-655.
Solyc10g051020.2	-2.04	cytochrome P450 CYP72A219	LOC104242999	Heme binding, iron ion binding, monooxygenase activity, acting on paired donors	N/A	Sierro, Nicolas, et al. "Reference genomes and transcriptomes of Nicotiana sylvestris and Nicotiana tomentosiformis." Genome biology 14.6 (2013): R60.
Solyc11g010270.2	-2.05	homeobox-leucine zipper protein ATHB-6	ATHB-6	Acts as a growth regulator in response to water deficit; Involved in the negative regulation of the ABA signaling pathway	*Impaired response to drought; Impaired regulation of ABA signaling*	Himmelbach, Axel, et al. "Homeodomain protein ATHB6 is a target of the protein phosphatase ABI1 and regulates hormone responses in Arabidopsis." The EMBO journal 21.12 (2002): 3029-3038.

TomatoID	DE in Infected	NCBI Protein Name	Gene ID	Uniprot Description	Putative Consequences for Infection	Citation
Solyc12g096490.1	-2.05	protein GLUTAMINE DUMPER 5-like	GDU5	Involved in the regulation of the amino acid metabolism	*Impaired amino acid tranport; Impaired regulation of amino acid metabolism*	Pratelli, Réjane, et al. "Stimulation of nonselective amino acid export by glutamine dumper proteins." Plant Physiology 152.2 (2010): 762-773.
Solyc07g014620.1	-2.11	auxin-responsive protein SAUR50	SAUR50	Effector of hormonal and environmental signals in plant growth	*Impaired response to hormonal and environmental signaling; Impaired growth/development*	Ren, Hong, and William M. Gray. "SAUR proteins as effectors of hormonal and environmental signals in plant growth." Molecular plant 8.8 (2015): 1153-1164.
Solyc07g056280.3	-2.11	WRKY transcription factor 30	WRKY30	Transcription factor involved in leaf senescence, response to hydrogen peroxide, response to ozone, and SA signaling pathway	*Impaired leaf senescence; Impaired response to hydrogen peroxide; Impaired reponse to ozone; Impaired resonse to SA signaling*	El-Esawi, Mohamed A., et al. "Overexpression of AtWRKY30 transcription factor enhances heat and drought stress tolerance in wheat (Triticum aestivum L.)." Genes 10.2 (2019): 163.
Solyc02g084930.3	-2.12	abscisic acid 8'-hydroxylase 3-like	CYP707A3	Involved in the oxidative degradation of ABA; Involved in the control of postgermination growth	*Impaired degradation of ABA; Impaired postgermination growth*	Umezawa, Taishi, et al. "CYP707A3, a major ABA 8′-hydroxylase involved in dehydration and rehydration response in Arabidopsis thaliana." The Plant Journal 46.2 (2006): 171-182.
Solyc12g036550.2	-2.12	Ycf1	Ycf1	Part of an intermediate translocation complex acting as a protein-conducting channel at the inner chloroplastic envelope	*Decreased protein precursor import into chloroplasts*	Kikuchi, Shingo, et al. "Uncovering the protein translocon at the chloroplast inner envelope membrane." Science 339.6119 (2013): 571-574.
Solyc01g100490.3	-2.15	nicotianamine synthase	NAS1	Synthesizes nicotianamine, a sensor for the physiological iron status within the plant; Involved in the transport of iron	*Impaired iron transport*	Weber, Michael, et al. "Comparative microarray analysis of Arabidopsis thaliana and Arabidopsis halleri roots identifies nicotianamine synthase, a ZIP transporter and other genes as potential metal hyperaccumulation factors." The Plant Journal 37.2 (2004): 269-281.
Solyc10g054820.2	-2.17	aquaporin-5 isoform X2	PIP2-5	Water channel required to facilitate the transport of water across cell membrane	*Decreased water transport*	Marmagne, Anne, et al. "Identification of new intrinsic proteins in Arabidopsis plasma membrane proteome." Molecular & Cellular Proteomics 3.7 (2004): 675-691.
Solyc09g090360.3	-2.22	phosphate transporter PHO1	PHO1	Regulates Pi starvation responses; Involved in the transfer of Pi from roots to shoots; Involved in ABA induction of stomatal closure	*Impaired Pi starvation response; Impaired transfer of Pi from roots to shoots; Impaired ABA induction of stomatal closure*	Stefanovic, Aleksandra, et al. "Members of the PHO1 gene family show limited functional redundancy in phosphate transfer to the shoot, and are regulated by phosphate deficiency via distinct pathways." The Plant Journal 50.6 (2007): 982-994.
Solyc02g011830.1	-2.24	Ycf1	Ycf1	Part of an intermediate translocation complex acting as a protein-conducting channel at the inner chloroplastic envelope	*Decreased protein precursor import into chloroplasts*	Kikuchi, Shingo, et al. "Uncovering the protein translocon at the chloroplast inner envelope membrane." Science 339.6119 (2013): 571-574.
Solyc11g021310.1	-2.27	Ycf1	Ycf1	Part of an intermediate translocation complex acting as a protein-conducting channel at the inner chloroplastic envelope	*Decreased protein precursor import into chloroplasts*	Kikuchi, Shingo, et al. "Uncovering the protein translocon at the chloroplast inner envelope membrane." Science 339.6119 (2013): 571-574.
Solyc05g049990.3	-2.29	heavy metal-associated isoprenylated plant protein 39-like	HIPP39	Heavy metal-binding protein involved in metal ion transport	*Impaired metal ion transport*	Hanada, Kousuke, et al. "Functional compensation of primary and secondary metabolites by duplicate genes in Arabidopsis thaliana." Molecular biology and evolution 28.1 (2011): 377-382.

TomatoID	DE in Infected	NCBI Protein Name	Gene ID	Uniprot Description	Putative Consequences for Infection	Citation
Solyc04g058150.3	-2.31	metallothionein-like protein type 2 A	MT2A	Functions as metal chelator of copper and Zn; Plays a role in Cu homeostasis	*Impaired copper homeostasis*	Guo, Woei-Jiun, Metha Meetam, and Peter B. Goldsbrough. "Examining the specific contributions of individual Arabidopsis metallothioneins to copper distribution and metal tolerance." Plant physiology 146.4 (2008): 1697-1706.
Solyc08g075880.3	-2.32	heavy metal-associated isoprenylated plant protein 30-like	HIPP30	Involved in metal ion transport; Involved in response to drought stress	*Impaired metal ion transport; Impaired response to drought stress*	"Stress induced and nuclear localized HIPP26 from Arabidopsis thaliana interacts via its heavy metal associated domain with the drought stress related zinc finger transcription factor ATHB29."
Solyc11g006290.2	-2.33	3-oxo-5-alpha-steroid 4-dehydrogenase 1	SRD5A1	Involved in phytohormone signaling	*Impaired phytohormone signaling*	Thieme, Christoph J., et al. "Endogenous Arabidopsis messenger RNAs transported to distant tissues." Nature Plants 1.4 (2015): 1-9.
Solyc03g045070.1	-2.44	ammonium transporter 1 member 3	AMT1-3	Transporter involved in ammonium uptake from the soil; Contributes with AMT1-1 to the overall ammonium uptake capacity in roots under nitrogen-deficiency conditions	*Impaired ammonium uptake and transport; Impaired response to nitrogen-deficiency*	Loqué, Dominique, et al. "Additive contribution of AMT1; 1 and AMT1; 3 to high-affinity ammonium uptake across the plasma membrane of nitrogen-deficient Arabidopsis roots." The Plant Journal 48.4 (2006): 522-534.
Solyc01g080870.3	-2.51	protein NRT1/ PTR FAMILY 7.3	NPF7.3	Low-affinity proton-dependent bidirectional nitrate transporter; Involved in nitrate loading into xylem	*Impaired nitrate transport*	Lin, Shan-Hua, et al. "Mutation of the Arabidopsis NRT1. 5 nitrate transporter causes defective root-to-shoot nitrate transport." The Plant Cell 20.9 (2008): 2514-2528.
Solyc01g110580.2	-2.55	auxin-responsive protein SAUR50	SAUR50	Effector of hormonal and environmental signals in plant growth	*Impaired response to hormonal and environmental signaling; Impaired growth/development*	Ren, Hong, and William M. Gray. "SAUR proteins as effectors of hormonal and environmental signals in plant growth." Molecular plant 8.8 (2015): 1153-1164.
Solyc11g012690.2	-2.56	heavy metal-associated isoprenylated plant protein 7-like isoform X2	HIPP07	Heavy metal-binding protein involved in metal ion transport	*Impaired metal ion transport*	Dykema, Philip E., et al. "A new class of proteins capable of binding transition metals." Plant molecular biology 41.1 (1999): 139-150.
Solyc12g087870.2	-2.62	purine permease 3-like	PUP3	Involved in transport of purine derivatives during pollen germination and tube elongation	*Impaired transport of purine derivatives during pollen germination and tube elongation*	Bürkle, Lukas, et al. "Transport of cytokinins mediated by purine transporters of the PUP family expressed in phloem, hydathodes, and pollen of Arabidopsis." The Plant Journal 34.1 (2003): 13-26.
Solyc05g012030.1	-2.63	protein BIG GRAIN 1-like E	AT1G69160	Involved in auxin transport; Regulator of the auxin signaling pathway	*Impaired auxin transport; Impaired regulation of auxin signaling*	Culligan, Kevin M., et al. "ATR and ATM play both distinct and additive roles in response to ionizing radiation." The Plant Journal 48.6 (2006): 947-961.
Solyc08g075570.3	-2.72	urea-proton symporter DUR3	DUR3	High-affinity urea-proton symporter involved in the active transport of urea across the plasma membrane into root cells	*Impaired urea uptake and transport*	Liu, Lai-Hua, et al. "AtDUR3 encodes a new type of high-affinity urea/H+ symporter in Arabidopsis." The Plant Cell 15.3 (2003): 790-800.

TomatoID	DE in Infected	NCBI Protein Name	Gene ID	Uniprot Description	Putative Consequences for Infection	Citation
Solyc05g047440.1	-2.75	Ycf1	Ycf1	Part of an intermediate translocation complex acting as a protein-conducting channel at the inner chloroplastic envelope	*Decreased protein precursor import into chloroplasts*	Kikuchi, Shingo, et al. "Uncovering the protein translocon at the chloroplast inner envelope membrane." Science 339.6119 (2013): 571-574.
Solyc10g047390.1	-2.92	Ycf1	Ycf1	Part of an intermediate translocation complex acting as a protein-conducting channel at the inner chloroplastic envelope	*Decreased protein precursor import into chloroplasts*	Kikuchi, Shingo, et al. "Uncovering the protein translocon at the chloroplast inner envelope membrane." Science 339.6119 (2013): 571-574.
Solyc09g008200.3	-2.95	heavy metal-associated isoprenylated plant protein 5	HIPP05	Heavy-metal-binding protein involved in metal ion tranpsport; Involved in disease resistance	*Impaired metal ion transport; Impaired disease resistance*	Cooper, Bret, et al. "A network of rice genes associated with stress response and seed development." Proceedings of the National Academy of Sciences 100.8 (2003): 4945-4950.
Solyc12g035550.1	-3.21	Ycf1	Ycf1	Part of an intermediate translocation complex acting as a protein-conducting channel at the inner chloroplastic envelope	*Decreased protein precursor import into chloroplasts*	Kikuchi, Shingo, et al. "Uncovering the protein translocon at the chloroplast inner envelope membrane." Science 339.6119 (2013): 571-574.
Solyc08g079230.1	-3.32	14 kDa proline-rich protein DC2.15-like	N/A	Involved with the initiation of embryogenesis or with the metabolic changes produced by the removal of auxins	*Impaired embryogenesis; Impaired response to auxin signaling*	Aleith, F., and G. Richter. "Gene expression during induction of somatic embryogenesis in carrot cell suspensions." Planta 183.1 (1991): 17-24.
Solyc08g016000.1	-3.35	Ycf1	Ycf1	Part of an intermediate translocation complex acting as a protein-conducting channel at the inner chloroplastic envelope	*Decreased protein precursor import into chloroplasts*	Kikuchi, Shingo, et al. "Uncovering the protein translocon at the chloroplast inner envelope membrane." Science 339.6119 (2013): 571-574.
Solyc07g006310.1	-3.43	transcription factor IBH1-like 1	IBL1	Acts as transcriptional repressor that negatively regulates cell and organ elongation in response to gibberellin and brassinosteroid signaling	*Impaired regulation of cell and organ elongation in response to gibberellin and brassinosteroid signaling*	Zhiponova, Miroslava K., et al. "Helix–loop–helix/basic helix–loop–helix transcription factor network represses cell elongation in Arabidopsis through an apparent incoherent feed-forward loop." Proceedings of the National Academy of Sciences 111.7 (2014): 2824-2829.
Solyc11g021300.1	-3.62	Ycf1	Ycf1	Part of an intermediate translocation complex acting as a protein-conducting channel at the inner chloroplastic envelope	*Decreased protein precursor import into chloroplasts*	Kikuchi, Shingo, et al. "Uncovering the protein translocon at the chloroplast inner envelope membrane." Science 339.6119 (2013): 571-574.
Solyc11g021260.1	-3.66	Ycf1	Ycf1	Part of an intermediate translocation complex acting as a protein-conducting channel at the inner chloroplastic envelope	*Decreased protein precursor import into chloroplasts*	Kikuchi, Shingo, et al. "Uncovering the protein translocon at the chloroplast inner envelope membrane." Science 339.6119 (2013): 571-574.
Solyc11g021290.2	-3.91	Ycf1	Ycf1	Part of an intermediate translocation complex acting as a protein-conducting channel at the inner chloroplastic envelope	*Decreased protein precursor import into chloroplasts*	Kikuchi, Shingo, et al. "Uncovering the protein translocon at the chloroplast inner envelope membrane." Science 339.6119 (2013): 571-574.

TomatoID	DE in Infected	NCBI Protein Name	Gene ID	Uniprot Description	Putative Consequences for Infection	Citation
Solyc10g049470.1	-4.08	Ycf1	Ycf1	Part of an intermediate translocation complex acting as a protein-conducting channel at the inner chloroplastic envelope	*Decreased protein precursor import into chloroplasts*	Kikuchi, Shingo, et al. "Uncovering the protein translocon at the chloroplast inner envelope membrane." Science 339.6119 (2013): 571-574.
Solyc11g021280.1	-4.23	Ycf1	Ycf1	Part of an intermediate translocation complex acting as a protein-conducting channel at the inner chloroplastic envelope	*Decreased protein precursor import into chloroplasts*	Kikuchi, Shingo, et al. "Uncovering the protein translocon at the chloroplast inner envelope membrane." Science 339.6119 (2013): 571-574.
Solyc11g021270.1	-4.71	Ycf1	Ycf1	Part of an intermediate translocation complex acting as a protein-conducting channel at the inner chloroplastic envelope	*Decreased protein precursor import into chloroplasts*	Kikuchi, Shingo, et al. "Uncovering the protein translocon at the chloroplast inner envelope membrane." Science 339.6119 (2013): 571-574.
