# Supplementary material for: Effects of ‘Candidatus Liberibacter solanacearum’ haplotypes A and B on tomato gene expression and geotropism
Source: BMC Plant Biol. 2022 Mar 30;22:156. doi: 10.1186/s12870-022-03505-z (PMC8966271; doi:10.1186/s12870-022-03505-z)
Supplement: Supplementary file 9 — Additional file 9: Table S9. The 44 tomato plant DEGs associated with transcription or translation regulation under Lso infection. DEGs were sorted by differential expression (DE) values comparing uninfected to infected samples (p-value< 0.05). NCBI Blast searches were used to identify Gene IDs and protein products in tomatoes as well as their homologs in other species. Specifically, the expression changes in 17 of these genes (39%, in italics) would have modified regulation of transcription or translation in infected plants. These DEGs were related to transcription regulation, response to auxin, and the homeostasis of certain compounds and ion. [file 12870_2022_3505_MOESM9_ESM.pdf]

TomatoID	DE in Infected	NCBI Protein Name	Gene ID	Uniprot Description	Putative Consequences for Infection	Citation
Solyc09g084450.3	6.67	transcription factor bHLH92 isoform X1	BHLH92	Involved in regulation of transcription	N/A	Jiang, Yuanqing, Bo Yang, and Michael K. Deyholos. "Functional characterization of the Arabidopsis bHLH92 transcription factor in abiotic stress." Molecular Genetics and Genomics 282.5 (2009): 503-516.
Solyc01g006400.3	4.94	Hop-interacting protein THI101 precursor	N/A	Transcriptional activator	N/A	N/A
Solyc06g076020.3	4.79	heat shock cognate 70 kDa protein 1	HSP70-1	Facilitates folding of de novo proteins, assists translocation of precursor proteins into organelles; Degrades damaged protein under stress conditions; Involved in defense response; Involved in protein transport to chloroplasts; Modulates stomatal aperture in response to environmental conditions	*Increased protein folding; Increased translocation of presursor proteins into organelles; Increased degradation of damaged proteins; Promoted defense response; Increased response to abiotic stress and ABA signaling*	Sung, Dong Yul, and Charles L. Guy. "Physiological and molecular assessment of altered expression of Hsc70-1 in Arabidopsis. Evidence for pleiotropic consequences." Plant Physiology 132.2 (2003): 979-987.
Solyc04g077980.1	4.66	C2H2-type zinc finger protein	At4g35610	Transcription factor activity	*Promoted transcription regulation*	Cheng, Chia-Yi, et al. "Araport11: a complete reannotation of the Arabidopsis thaliana reference genome." The Plant Journal 89.4 (2017): 789-804.
Solyc03g116890.3	4.56	probable WRKY transcription factor 40 isoform X1	WRKY40	Transcription factor	N/A	Eulgem, Thomas, et al. "The WRKY superfamily of plant transcription factors." Trends in plant science 5.5 (2000): 199-206.
Solyc03g026280.3	3.80	C-repeat-binding factor-1	CBF1	Transcription factor	N/A	Zhen, Ying, and Mark C. Ungerer. "Relaxed selection on the CBF/DREB1 regulatory genes and reduced freezing tolerance in the southern range of Arabidopsis thaliana." Molecular Biology and Evolution 25.12 (2008): 2547-2555.
Solyc02g087540.2	3.72	AAA-ATPase At3g28510-like	AT3G28510	Involved in inter-tissue RNA transport	*Increased inter-tissue RNA transport*	Thieme, Christoph J., et al. "Endogenous Arabidopsis messenger RNAs transported to distant tissues." Nature Plants 1.4 (2015): 1-9.
Solyc02g079590.3	3.62	receptor-like serine/threonine-protein kinase SD1-8	SD18	Involved in the regulation of cellular expansion and differentiation	*Increased regulation of cellular expansion and differentiation*	Ascencio-Ibáñez, José Trinidad, et al. "Global analysis of Arabidopsis gene expression uncovers a complex array of changes impacting pathogen response and cell cycle during geminivirus infection." Plant physiology 148.1 (2008): 436-454.
Solyc04g082140.3	3.52	multicopper oxidase-like protein precursor	LPR1	Multicopper oxidase involved in Cu homeostasis and oxidative stress response; Necessary for root growth inhibition	*Promoted Cu homeostasis; Increased response to oxidative stress; Root growth inhibition*	Svistoonoff, Sergio, et al. "Root tip contact with low-phosphate media reprograms plant root architecture." Nature genetics 39.6 (2007): 792-796.

TomatoID	DE in Infected	NCBI Protein Name	Gene ID	Uniprot Description	Putative Consequences for Infection	Citation
Solyc10g009270.3	3.50	transcription factor MYC2-like	MYC2	Involved in the regulation of ABA-inducible genes under drought stress; Negative regulator of light-regulated gene expression and growth; Positive regulator of lateral root formation; Regulates sesquiterpene biosynthesis	*Promoted response to drought; Impaired response to light-mediated expression; Promoted lateral root formation; Increased regulation of sesquiterpene biosynthesis*	Abe, Hiroshi, et al. "Role of Arabidopsis MYC and MYB homologs in drought-and abscisic acid-regulated gene expression." The Plant Cell 9.10 (1997): 1859-1868.
Solyc06g074030.1	3.26	probable CCR4-associated factor 1 homolog 9	CAF1-9	Ubiquitous transcription factor required for a diverse set of processes; Component of the CCR4 complex involved in the control of gene expression	*Promoted gene expression*	Chang, Shuang, and Craig S. Pikaard. "Transcript profiling in Arabidopsis reveals complex responses to global inhibition of DNA methylation and histone deacetylation." Journal of Biological Chemistry 280.1 (2005): 796-804.
Solyc12g005450.1	3.07	probable receptor-like protein kinase At4g10390	AT4G10390	Involved in protein phosphorylation; Involved in response to wounding	*Increased protein phosphorylation; Promoted response to wounding*	Peng, Mingsheng, et al. "Genome-wide analysis of Arabidopsis responsive transcriptome to nitrogen limitation and its regulation by the ubiquitin ligase gene NLA." Plant molecular biology 65.6 (2007): 775-797.
Solyc07g006570.3	2.99	ribonuclease 3-like	RTL1	Involved in production of siRNA involved in RNA interference	*Increased gene silencing*	Charbonnel, Cyril, et al. "The siRNA suppressor RTL1 is redox-regulated through glutathionylation of a conserved cysteine in the double-stranded-RNA-binding domain." Nucleic acids research 45.20 (2017): 11891-11907.
Solyc01g057770.3	2.80	boron transporter 1 isoform X1	BOR1	Efflux-type boron transporter for xylem loading Boron is essential for maintaining the integrity of plants cell walls	*Promoted boron homeostasis; Promoted plant cell wall development and integrity*	Noguchi, Kyotaro, et al. "bor1-1, an Arabidopsis thaliana mutant that requires a high level of boron." Plant Physiology 115.3 (1997): 901-906.
Solyc08g008280.3	2.73	probable WRKY transcription factor 53	WRKY53	Transcription factor regulating the early events of leaf senescence; Negatively regulates the expression of ESR/ESP; Promotes resistance to certain pathogens by enhancing SA- dependent genes; Contributes to the suppression of PDF1.2	*Down regulation of ESR/ESP; Promoted resistance to certain pathogens by enhancing SA- dependent genes; Suppression of PDF1.2*	Miao, Ying, and Ulrike Zentgraf. "The antagonist function of Arabidopsis WRKY53 and ESR/ESP in leaf senescence is modulated by the jasmonic and salicylic acid equilibrium." The Plant Cell 19.3 (2007): 819-830.
Solyc09g018250.2	2.63	GBF-interacting protein 1-like isoform X1	GIP1	Contributes to bZIP-mediated gene regulation; Acts as negative co-regulator in red and blue light-mediated hypocotyl elongation	*Promoted gene regulation; Increased seed germination*	Sehnke, Paul C., et al. "Identification and characterization of GIP1, an Arabidopsis thaliana protein that enhances the DNA binding affinity and reduces the oligomeric state of G-box binding factors." Cell research 15.8 (2005): 567-575.
Solyc06g075010.3	2.53	ruBisCO large subunit-binding protein subunit alpha, chloroplastic	N/A	Involved in protein refolding	N/A	N/A
Solyc07g018010.3	2.43	transcription factor bHLH35	BHLH35	Involved in regulation of transcription	N/A	Heim, Marc A., et al. "The basic helix–loop–helix transcription factor family in plants: a genome-wide study of protein structure and functional diversity." Molecular biology and evolution 20.5 (2003): 735-747.

TomatoID	DE in Infected	NCBI Protein Name	Gene ID	Uniprot Description	Putative Consequences for Infection	Citation
Solyc07g062700.3	2.43	sodium/calcium exchanger NCL	NCL	Participates in the maintenance of calcium homeostasis; Plays a role in auxin response, diurnal rhythm, and flowering time; Involved in salt stress response	*Promoted calcium homeostasis; Increased response to auxin; Promoted circadian rhythm; Promoted salt stress response*	Wang, Peng, et al. "A Na+/Ca2+ exchanger-like protein (AtNCL) involved in salt stress in Arabidopsis." Journal of Biological Chemistry 287.53 (2012): 44062-44070.
Solyc01g086800.3	2.36	histone H3.2	HTR2	Core component of the nucleosome; Plays central roles in transcription regulation, DNA repair, DNA replication and chromosomal stability	*Increased transcription regulation and DNA repair*	Seki, Motoaki, et al. "Functional annotation of a full-length Arabidopsis cDNA collection." Science 296.5565 (2002): 141-145.
Solyc05g007490.3	2.11	probable methyltransferase PMT2	AT1G26850	Involved in methylation	N/A	Ascencio-Ibáñez, José Trinidad, et al. "Global analysis of Arabidopsis gene expression uncovers a complex array of changes impacting pathogen response and cell cycle during geminivirus infection." Plant physiology 148.1 (2008): 436-454.
Solyc01g079600.3	2.09	phospholipase A1 PLIP2, chloroplastic isoform X1	PLIP2	Catalyzes the initial step of oxylipins and jasmonate biosynthesis; Links ABA-mediated abiotic stress responses and oxylipin and jasmonate signaling pathways	*Increased jasmonate biosynthesis; Increased jasmonate signaling crosstalk*	Wang, Kun, et al. "Two abscisic acid-responsive plastid lipase genes involved in jasmonic acid biosynthesis in Arabidopsis thaliana." The Plant Cell 30.5 (2018): 1006-1022.
Solyc01g080600.3	2.06	histone H3.2	HTR2	Core component of the nucleosome; Plays central roles in transcription regulation, DNA repair/replication, and chromosomal stability	*Increased transcription regulation and DNA repair*	Seki, Motoaki, et al. "Functional annotation of a full-length Arabidopsis cDNA collection." Science 296.5565 (2002): 141-145.
Solyc08g075870.3	2.05	probable methyltransferase PMT21	ERD3	Involved in methylation	N/A	Ascencio-Ibáñez, José Trinidad, et al. "Global analysis of Arabidopsis gene expression uncovers a complex array of changes impacting pathogen response and cell cycle during geminivirus infection." Plant physiology 148.1 (2008): 436-454.
Solyc05g050560.1	2.04	transcription factor bHLH13-like	BHLH13	Involved in negative regulation of gene expression	N/A	Borsics, Tamás, et al. "The cyclic nucleotide-gated calmodulin-binding channel AtCNGC10 localizes to the plasma membrane and influences numerous growth responses and starch accumulation in Arabidopsis thaliana." Planta 225.3 (2007): 563-573.
Solyc01g107390.3	2.02	indole-3-acetic acid-amido synthetase GH3.2	GH3.2	Catalyzes the synthesis of indole-3-acetic acid-amino acid conjugates, providing a mechanism for the plant to cope with the presence of excess auxin	*Promoted auxin homeostasis*	Hilson, Pierre, et al. "Versatile gene-specific sequence tags for Arabidopsis functional genomics: transcript profiling and reverse genetics applications." Genome research 14.10b (2004): 2176-2189.
Solyc01g102640.2	-2.00	nuclear pore complex protein NUP98A-like	NUP98A	Structural constituent of nuclear pore; Necessary for mRNA transport from the nucleus and protein import into the nucleus; Required for photoperiodism; Required for constitutive defense	Impaired mRNA transport; Impaired photoperiodism; Impaired constitutive defense	"A putative nucleoporin 96 is required for both basal defense and constitutive resistance responses mediated by suppressor of npr1-1,constitutive 1."

TomatoID	DE in Infected	NCBI Protein Name	Gene ID	Uniprot Description	Putative Consequences for Infection	Citation
Solyc08g068490.3	-2.09	indole-3-acetic acid-amido synthetase GH3.10-like	GH3.10	Catalyzes the synthesis of indole-3-acetic acid-amino acid conjugates, providing a mechanism for the plant to cope with the presence of excess auxin	Impaired auxin homeostasis	Takase, Tomoyuki, et al. "DFL2, a new member of the Arabidopsis GH3 gene family, is involved in red light-specific hypocotyl elongation." Plant and Cell Physiology 44.10 (2003): 1071-1080.
Solyc04g007210.3	-2.13	zinc finger protein CONSTANS-LIKE 16	COL16	Involved in regulation of transcription	N/A	Khanna, Rajnish, et al. "The Arabidopsis B-box zinc finger family." The Plant Cell 21.11 (2009): 3416-3420.
Solyc11g069700.2	-2.14	elongation factor 1-alpha	A1	Promotes the GTP-dependent binding of aminoacyl-tRNA to the A-site of ribosomes during protein biosynthesis	Impaired binding of aminoacyl-tRNA to the A-site of ribosomes during protein biosynthesis	Lahmy, Sylvie, et al. "DOMINO1, a member of a small plant-specific gene family, encodes a protein essential for nuclear and nucleolar functions." The Plant Journal 39.6 (2004): 809-820.
Solyc06g060830.3	-2.17	homeobox-leucine zipper protein HAT4	HAT4	Involved in the negative regulation of cell elongation and specific cell proliferation processes; Mediator of the red/far-red light effects on leaf cell expansion in the shading response	Impaired regulation of cell elongation; Impaired regulation of lateral root formation and vascular system; Impaired shading response	Schena, Mark, Alan M. Lloyd, and Ronald W. Davis. "The HAT4 gene of Arabidopsis encodes a developmental regulator." Genes & development 7.3 (1993): 367-379.
Solyc10g080920.2	-2.19	protein RADIALIS-like 1	RL1	Transcription factor	N/A	Cheng, Chia-Yi, et al. "Araport11: a complete reannotation of the Arabidopsis thaliana reference genome." The Plant Journal 89.4 (2017): 789-804.
Solyc07g066330.3	-2.20	NAC domain-containing protein 21/22 isoform X2	LOC107784013	Involved in regulation of transcription	Impaired regulation of transcription	Sierro, Nicolas, et al. "The tobacco genome sequence and its comparison with those of tomato and potato." Nature communications 5.1 (2014): 1-9.
Solyc01g007810.1	-2.37	transcription repressor OFP15	OFP15	Transcriptional repressor that regulates multiple aspects of plant growth and development	Promoted plant growth and development; Impaired regulation of plant growth and development	Wang, Shucai, et al. "Arabidopsis ovate family proteins, a novel transcriptional repressor family, control multiple aspects of plant growth and development." PLoS One 6.8 (2011): e23896.
Solyc01g112190.3	-2.41	protein NLP2	NLP2	Transcription factor	N/A	Konishi, Mineko, and Shuichi Yanagisawa. "Arabidopsis NIN-like transcription factors have a central role in nitrate signalling." Nature communications 4.1 (2013): 1-9.
Solyc05g007880.3	-2.46	cyclic dof factor 1 isoform X1	CDF1	Regulates a photoperiodic flowering response; Transcriptional repressor of 'CONSTANS' expression	Impaired photoperiodic flowering response; Decreased repression of CONSTANS expression	Imaizumi, Takato, et al. "FKF1 F-box protein mediates cyclic degradation of a repressor of CONSTANS in Arabidopsis." Science 309.5732 (2005): 293-297.
Solyc11g008530.2	-2.48	endoribonuclease Dicer 2d isoform X1	AT3G03300	Plays an essential role in transitive silencing of transgenes by processing secondary siRNAs; Plays a role in antiviral RNA silencing	Impaired post-transcriptional gene silencing; Impaired defense against viral pathogens	"DICER-LIKE2 plays a primary role in transitive silencing of transgenes in Arabidopsis."
Solyc01g049690.3	-2.51	transcription factor Pur-alpha 1 isoform X2	PURA1	Transcription factor that specifically binds the purine-rich double-stranded telomeric repeated sequence	Impaired gene expression	Tremousaygue, Dominique, et al. "Plant interstitial telomere motifs participate in the control of gene expression in root meristems." The Plant Journal 20.5 (1999): 553-561.
Solyc05g041360.2	-2.65	maturase K	matK	Assists in splicing its own and other chloroplast group II introns	Impaired chloroplastic gene splicing	Sato, Shusei, et al. "Complete structure of the chloroplast genome of Arabidopsis thaliana." DNA research 6.5 (1999): 283-290.

TomatoID	DE in Infected	NCBI Protein Name	Gene ID	Uniprot Description	Putative Consequences for Infection	Citation
Solyc11g073120.2	-2.66	transcription factor [illegible]	MYB48	Transcriptional regulator	N/A	Riechmann, José Luis, et al. "Arabidopsis transcription factors: genome-wide comparative analysis among
Solyc09g061390.1	-2.66	maturase K	matK	Assists in splicing its own and other chloroplast group II introns	Impaired chloroplastic gene splicing	Sato, Shusei, et al. "Complete structure of the chloroplast genome of Arabidopsis thaliana." DNA research 6.5 (1999): 283-290.
Solyc06g005330.3	-2.71	transcription factor MYB48-like	MYB48	Transcriptional regulator	N/A	Riechmann, José Luis, et al. "Arabidopsis transcription factors: genome-wide comparative analysis among eukaryotes." Science 290.5499 (2000): 2105-2110.
Solyc06g005320.1	-3.02	transcription factor MYB48-like	MYB48	Transcriptional regulator	N/A	Riechmann, José Luis, et al. "Arabidopsis transcription factors: genome-wide comparative analysis among eukaryotes." Science 290.5499 (2000): 2105-2110.
Solyc06g005310.3	-3.70	transcription factor MYB48-like	MYB48	Transcriptional regulator	N/A	Riechmann, José Luis, et al. "Arabidopsis transcription factors: genome-wide comparative analysis among eukaryotes." Science 290.5499 (2000): 2105-2110.
