# Supplementary material for: Effects of ‘Candidatus Liberibacter solanacearum’ haplotypes A and B on tomato gene expression and geotropism
Source: BMC Plant Biol. 2022 Mar 30;22:156. doi: 10.1186/s12870-022-03505-z (PMC8966271; doi:10.1186/s12870-022-03505-z)
Supplement: Supplementary file 10 — Additional file 10: Table S10. Complete table of all 451 DEGs between LsoA-infected and LsoB-infected tomato plants. Columns depict (in order) the tomato transcript ID, p- and q-values comparing the fragments per kilobase of transcript per million reads (fpkm) between uninfected to infected samples, the differential expression (DE) comparing uninfected to infected samples, the fpkm for each sample, the NCBI protein name for the identified transcript, its gene ID, the A. thaliana homolog used in the g:Profiler analysis, the UniProt description, the predicted consequences for differential expression after infection (based on homologs of published genes), and supporting literature. [file 12870_2022_3505_MOESM10_ESM.pdf]

TomatoID	DE	NCBI Protein Name	Gene ID	Uniprot Description	Putative Consequences for Infection	Citation
Solyc11g027710.1	-6.40	N/A	N/A	N/A	N/A	N/A
Solyc09g089530.3	-5.65	proteinase inhibitor 1-like	N/A	N/A	N/A	N/A
Solyc12g096780.2	-4.69	enoyl-[acyl-carrier-protein] reductase, mitochondrial-like	AT3G45770	Catalyzes the NADPH-dependent reduction of trans-2-enoyl thioesters in mitochondrial fatty acid synthesis	Decreased fatty acid synthesis and metabolism	Li-Beisson, Yonghua, et al. "Acyl-lipid metabolism." The Arabidopsis book/American Society of Plant Biologists 11 (2013).
Solyc09g089520.3	-4.23	proteinase inhibitor I-B-like	LOC107794480	Involved in response to wounding	*Impaired response to wounding*	Sierro, Nicolas, et al. "The tobacco genome sequence and its comparison with those of tomato and potato." Nature communications 5.1 (2014): 1-9.
Solyc11g071480.1	-3.83	agmatine hydroxycinnamoyltransferase 1-like	AHT1	Promotes the synthesis of hydroxycinnamic acid amides, which play a role in defense against pathogens	*Impaired defense against pathogens*	Chen, Wei, et al. "Genome-wide association analyses provide genetic and biochemical insights into natural variation in rice metabolism." Nature genetics 46.7 (2014): 714-721.
Solyc09g008670.3	-3.65	threonine deaminase	N/A	Involved in cellular amino acid biosynthetic process	N/A	Tieman, Denise M., and Avtar K. Handa. "Molecular cloning and characterization of genes expressed during early tomato (Lycopersicon esculentum Mill.) fruit development by mRNA differential display." Journal of the American Society for Horticultural Science 121.1 (1996): 52-56.
Solyc09g084450.3	-3.42	transcription factor bHLH92 isoform X1	BHLH92	Involved in regulation of transcription	N/A	Jiang, Yuanqing, Bo Yang, and Michael K. Deyholos. "Functional characterization of the Arabidopsis bHLH92 transcription factor in abiotic stress." Molecular Genetics and Genomics 282.5 (2009): 503-516.
Solyc08g074620.3	-3.40	phenol oxidase E, chloroplastic	N/A	Involved in pigment biosynthesis process	*Impaired pigment biosynthesis*	Shahar, Tamar, Nava Hennig, Tamar Gutfinger, Dana Hareven, and Eliezer Lifschitz. "The tomato 66.3-kD polyphenoloxidase gene: molecular identification and developmental expression." The Plant Cell 4, no. 2 (1992): 135-147.
Solyc01g006400.3	-3.08	Hop-interacting protein THI101 precursor	N/A	Transcriptional activator	N/A	N/A
Solyc03g083770.1	-2.89	21 kDa protein	HSP21	N/A	N/A	N/A
Solyc09g089540.3	-2.82	wound-induced proteinase inhibitor 1-like	N/A	Inhibits both chymotrypsin and trypsin	Impaired serine protease activity	Richardson, M., and L. Cossins. "Chymotryptic inhibitor I from potatoes: the amino acid sequences of subunits B, C and D." FEBS letters 45, no. 1-2 (1974): 11-13.
Solyc01g073695.1	-2.82	N/A	N/A	N/A	N/A	N/A
Solyc12g010030.2	-2.80	leucine aminopeptidase 2, chloroplastic isoform X1	LAP2	Involved in the processing and regular turnover of intracellular proteins; Heat shock protein	*Impaired turnover of intracellular proteins; Impaired response to heat-induced damaged*	Waditee-Sirisattha, Rungaroon, et al. "The Arabidopsis aminopeptidase LAP2 regulates plant growth, leaf longevity and stress response." New Phytologist 191.4 (2011): 958-969.

TomatoID	DE	NCBI Protein Name	Gene ID	Uniprot Description	Putative Consequences for Infection	Citation
Solyc07g008570.3	-2.75	probable inactive purple acid phosphatase 27	PAP27	Acid phosphatase activity; Metal ion binding	N/A	Cheng, Chia-Yi, et al. "Araport11: a complete reannotation of the Arabidopsis thaliana reference genome." The Plant Journal 89.4 (2017): 789-804.
Solyc12g010020.2	-2.71	leucine aminopeptidase 1, chloroplastic	LAPA1	Involved in the processing and regular turnover of intracellular proteins	Impaired intracellular protein turnover	Pautot, Veronique, et al. "Leucine aminopeptidase: an inducible component of the defense response in Lycopersicon esculentum (tomato)." Proceedings of the National Academy of Sciences 90.21 (1993): 9906-9910.
Solyc12g010025.1	-2.70	N/A	N/A	N/A	N/A	N/A
Solyc07g055720.3	-2.69	uncharacterized protein LOC101259555 isoform X2	N/A	N/A	N/A	N/A
Solyc00g187050.3	-2.68	leucine aminopeptidase 1, chloroplastic	LAPA1	Involved in the processing and regular turnover of intracellular proteins	Impaired intracellular protein turnover	Pautot, Veronique, et al. "Leucine aminopeptidase: an inducible component of the defense response in Lycopersicon esculentum (tomato)." Proceedings of the National Academy of Sciences 90.21 (1993): 9906-9910.
Solyc01g090180.3	-2.64	4,5-DOPA dioxygenase extradiol	DODA	Opens the cyclic ring of dihydroxy-phenylalanine, producing betalamic acid	*Impaired betalamic acid production; Impaired pigment production*	Christinet, Laurent, et al. "Characterization and functional identification of a novel plant 4, 5-extradiol dioxygenase involved in betalain pigment biosynthesis in Portulaca grandiflora." Plant Physiology 134.1 (2004): 265-274.
Solyc06g009190.3	-2.60	pectinesterase	PME1	Acts in the modification of cell walls via demethylesterification of cell wall pectin; Acts as negative regulators of genes involved in salt stress response	*Impaired cell wall modification; Impaired response to salt stress*	Creighton, Maria T., Anna Kolton, Amr RA Kataya, Jodi Maple-Grødem, Irina O. Averkina, Behzad Heidari, and Cathrine Lillo. "Methylation of protein phosphatase 2A—Influence of regulators and environmental stress factors." Plant, cell & environment 40, no. 10 (2017): 2347-2358.
Solyc06g083130.3	-2.55	dCTP pyrophosphatase 1	DCTPP1	Hydrolyzes dNTPs to the corresponding nucleoside monophosphates; Protects DNA/RNA against the incorporation of these genotoxic nucleotide analogs through their catabolism	*Impaired DNA/RNA protection against genotoxic nucleotide analogs*	Cheng, Chia-Yi, et al. "Araport11: a complete reannotation of the Arabidopsis thaliana reference genome." The Plant Journal 89.4 (2017): 789-804.
Solyc01g091170.3	-2.54	Arginase 2, chloroplastic/mitochondrial	ARGAH2	Utilized in the urea cycle; Precursor for the synthesis of both polyamines and proline; Catalyzes the formation of putrescine from agmatine	*Impaired jasmonate-related functions; Impaired urea waste management; Impaired freezing tolerance*	Dombrecht, Bruno, et al. "MYC2 differentially modulates diverse jasmonate-dependent functions in Arabidopsis." The Plant Cell 19.7 (2007): 2225-2245.
Solyc05g005535.1	-2.53	N/A	N/A	N/A	N/A	N/A
Solyc10g079350.2	-2.52	zeatin O-glucosyltransferase-like	ZOG1	Regulates cytokinin activity and storage; Impacts seed growth	*Impaired cellular division and plant growth in roots and shoots; Impaired reproductive investment*	Hou, Bingkai, et al. "N-glucosylation of cytokinins by glycosyltransferases of Arabidopsis thaliana." Journal of Biological Chemistry 279.46 (2004): 47822-47832.

TomatoID	DE	NCBI Protein Name	Gene ID	Uniprot Description	Putative Consequences for Infection	Citation
Solyc00g145170.2	-2.49	proteinase inhibitor type-2 precursor	N/A	N/A	N/A	Balandin, Teresa, et al. "Structure and induction pattern of a novel proteinase inhibitor class II gene of tobacco." Plant molecular biology 27.6 (1995): 1197-1204.
Solyc07g008380.2	-2.48	salutaridinol 7-O-acetyltransferase-like	SALAT	Involved in biosynthesis of morphinan-type benzylisoquinoline alkaloids	Impaired biosynthesis of morphinan-type benzylisoquinoline alkaloids	Lenz, Rainer, and Meinhart H. Zenk. "Acetyl coenzyme A: salutaridinol-7-O-acetyltransferase from Papaver somniferum plant cell cultures: The enzyme catalyzing the formation of thebaine in morphine biosynthesis." Journal of Biological Chemistry 270.52 (1995): 31091-31096.
Solyc07g064600.3	-2.36	inducible plastid-lipid associated protein	CHRD	Involved in organonitrogen compound catabolic process	N/A	N/A
Solyc06g083470.3	-2.32	tropinone reductase homolog At5g06060	At5g06060	Has oxidoreductase activity	*Decreased oxidoreductase activity*	Ciftci-Yilmaz, Sultan, Mustafa R. Morsy, Luhua Song, Alicia Coutu, Beth A. Krizek, Michael W. Lewis, Daniel Warren, John Cushman, Erin L. Connolly, and Ron Mittler. "The EAR-motif of the Cys2/His2-type zinc finger protein Zat7 plays a key role in the defense response of Arabidopsis to salinity stress." Journal of Biological Chemistry 282, no. 12 (2007): 9260-9268.
Solyc06g083480.3	-2.32	tropinone reductase homolog At5g06060	At5g06060	Has oxidoreductase activity	*Decreased oxidoreductase activity*	Ciftci-Yilmaz, Sultan, Mustafa R. Morsy, Luhua Song, Alicia Coutu, Beth A. Krizek, Michael W. Lewis, Daniel Warren, John Cushman, Erin L. Connolly, and Ron Mittler. "The EAR-motif of the Cys2/His2-type zinc finger protein Zat7 plays a key role in the defense response of Arabidopsis to salinity stress." Journal of Biological Chemistry 282, no. 12 (2007): 9260-9268.
Solyc06g006000.3	-2.28	uncharacterized protein LOC101264428	N/A	N/A	N/A	N/A
Solyc02g089350.3	-2.26	protein GAST1 precursor	GAST1	Involved in root-specific abscisic acid-signaling regulation	*Impaired abscisic acid-signaling regulation*	Shi, Lifang, Robert T. Gast, Manjula Gopalraj, and Neil E. Olszewski. "Characterization of a shoot-specific, GA3-and ABA-regulated gene from tomato." The Plant Journal 2, no. 2 (1992): 153-159.
Solyc01g005300.3	-2.25	adagio protein 3	ADO3	Component of an E3 ubiquitin ligase complex that plays a central role in blue light-dependent circadian cycles	*Impaired E3 ubiquitination; Accumulation of misfolded proteins*	Fornara, Fabio, Kishore CS Panigrahi, Lionel Gissot, Nicolas Sauerbrunn, Mark Rühl, José A. Jarillo, and George Coupland. "Arabidopsis DOF transcription factors act redundantly to reduce CONSTANS expression and are essential for a photoperiodic flowering response." Developmental cell 17, no. 1 (2009): 75-86.
Solyc03g123410.1	-2.24	auxin-binding protein ABP19a	ABP19A	Receptor for the plant growth-promoting hormone auxin	*Impaired auxin signaling*	Ohmiya, Akemi, Yoshiyuki Tanaka, Koh-ichi Kadowaki, and Tateki Hayashi. "Cloning of genes encoding auxin-binding proteins (ABP19/20) from peach: significant peptide sequence similarity with germin-like proteins." Plant and cell physiology 39, no. 5 (1998): 492-499.

TomatoID	DE	NCBI Protein Name	Gene ID	Uniprot Description	Putative Consequences for Infection	Citation
Solyc11g066390.2	-2.23	superoxide dismutase [Cu-Zn], chloroplastic	CSD2	Processes reactive oxygen species which are normally produced within the cells; Mediates tolerance to stress, including photo-oxidative stress	*Impaired photo-oxidative stress response*	Abarca, Dolores, Marta Roldán, Mercedes Martín, and Bartolomé Sabater. "Arabidopsis thaliana ecotype Cvi shows an increased tolerance to photo-oxidative stress and contains a new chloroplastic copper/zinc superoxide dismutase isoenzyme." Journal of experimental botany 52, no. 360 (2001): 1417-1425.
Solyc10g009150.3	-2.22	organ-specific protein S2	N/A	N/A	N/A	Williams, Mary E., John Mundy, Steven A. Kay, and Nam-Hai Chua. "Differential expression of two related organ-specific genes in pea." Plant molecular biology 14, no. 5 (1990): 765-774.
Solyc03g098300.1	-2.22	ornithine decarboxylase-like	ODC	Catalyzes the first and rate-limiting step of polyamine biosynthesis; Polyamines are essential for cell proliferation	*Impaired polyamine biosynthesis; Impaired cell proliferation*	Alabadı, David, and Juan Carbonell. "Expression of ornithine decarboxylase is transiently increased by pollination, 2, 4-dichlorophenoxyacetic acid, and gibberellic acid in tomato ovaries." Plant physiology 118, no. 1 (1998): 323-328.
Solyc05g007830.3	-2.19	expansin-A1	EXPA1	Causes loosening and extension of plant cell walls by disrupting non-covalent bonding between cellulose microfibrils and matrix glucans	*Impaired plant cell wall extension*	Zhang, Xiu-Qing, Peng-Cheng Wei, Yan-Mei Xiong, Yi Yang, Jia Chen, and Xue-Chen Wang. "Overexpression of the Arabidopsis α-expansin gene AtEXPA1 accelerates stomatal opening by decreasing the volumetric elastic modulus." Plant cell reports 30, no. 1 (2011): 27-36.
Solyc07g042390.2	-2.17	plant invertase/pectin methylesterase inhibitor superfamily protein precursor	At3g17140	Has enzyme inhibitor activity	Impaired enzyme inhibition	Johnston, Amal J., Patrick Meier, Jacqueline Gheyselinck, Samuel EJ Wuest, Michael Federer, Edith Schlagenhauf, Jörg D. Becker, and Ueli Grossniklaus. "Genetic subtraction profiling identifies genes essential for Arabidopsis reproduction and reveals interaction between the female gametophyte and the maternal sporophyte." Genome biology 8, no. 10 (2007): 1-21.
Solyc08g067530.1	-2.16	non-specific lipid-transfer protein 1-like	LTP1	Plays a role in wax or cutin deposition in the cell walls of expanding epidermal cells and certain secretory tissues	*Impaired wax/cutin deposition; Impaired plant cell wall expansion*	Skriver, Karen, Robert Leah, Frieder Müller-Uri, Finn-Lok Olsen, and John Mundy. "Structure and expression of the barley lipid transfer protein gene Ltp1." Plant molecular biology 18, no. 3 (1992): 585-589.
Solyc12g015690.2	-2.14	fasciclin-like arabinogalactan protein 1	FLA1	Cell surface adhesion protein; Involved in root and shoot development	*Impaired root/shoot development*	Sultana, Nighat, Hannah V. Florance, Alex Johns, and Nicholas Smirnoff. "Ascorbate deficiency influences the leaf cell wall glycoproteome in A rabidopsis thaliana." Plant, cell & environment 38, no. 2 (2015): 375-384.
Solyc01g005290.3	-2.13	fasciclin-like arabinogalactan protein 1	FLA1	Cell surface adhesion protein; Involved in root and shoot development	*Impaired root/shoot development*	Sultana, Nighat, Hannah V. Florance, Alex Johns, and Nicholas Smirnoff. "Ascorbate deficiency influences the leaf cell wall glycoproteome in A rabidopsis thaliana." Plant, cell & environment 38, no. 2 (2015): 375-384.
Solyc01g017490.1	-2.12	N/A	N/A	N/A	N/A	N/A

TomatoID	DE	NCBI Protein Name	Gene ID	Uniprot Description	Putative Consequences for Infection	Citation
Solyc03g120990.3	-2.09	NADP-dependent malic enzyme, chloroplastic	ME6	Cecarboxylates malate shuttled from neighboring mesophyll cells; CO2 released is refixed by RuBisCo; Eliminates the photorespiratory loss of CO2 that occurs in most plants	*Impaired photorespiratory efficiency of CO2 loss*	Cheng, Yuxiang, et al. "Expression, purification, and characterization of two NADP-malic enzymes of rice (Oryza sativa L.) in Escherichia coli." Protein expression and purification 45.1 (2006): 200-205.
Solyc11g027645.1	-2.09	N/A	N/A	N/A	N/A	N/A
Solyc03g118780.3	-2.04	thaumatin-like protein	TLP1	Involved in local responses of roots to colonization by non-pathogenic plant growth-promoting rhizobacteria fluorescent Pseudomonas spp.	*Impaired rhizobacterial integration*	Leon-Kloosterziel, Karen M., Bas WM Verhagen, Joost JB Keurentjes, Johan A. Van Pelt, Martijn Rep, L. C. Van Loon, and Corne MJ Pieterse. "Colonization of the Arabidopsis rhizosphere by fluorescent Pseudomonas spp. activates a root-specific, ethylene-responsive PR-5 gene in the vascular bundle." Plant molecular biology 57, no. 5 (2005): 731-748.
Solyc00g068980.2	-2.04	N/A	N/A	N/A	N/A	N/A
Solyc07g008410.3	-2.01	protein DETOXIFICATION 29-like	DTX29	Xenobiotic transmembrane transporter activity	*Impaired detoxification*	Hanada, Kousuke, et al. "Functional compensation of primary and secondary metabolites by duplicate genes in Arabidopsis thaliana." Molecular biology and evolution 28.1 (2011): 377-382.
Solyc02g076730.2	-1.98	low-temperature-induced cysteine proteinase-like	N/A	Proteolysis involved in cellular protein catabolic process	Impaired protein catabolism	Bar-Ziv, Amalia, Yael Levy, Hagit Hak, Anahit Mett, Eduard Belausov, Vitaly Citovsky, and Yedidya Gafni. "The Tomato yellow leaf curl virus (TYLCV) V2 protein interacts with the host papain-like cysteine protease CYP1." Plant signaling & behavior 7, no. 8 (2012): 983-989.
Solyc01g096220.3	-1.98	ras-related protein RABA3	RABA3	Involved in intracellular vesicle trafficking and protein transport	*Impaired intracellular vesicle trafficking*	Lunn, Daniel, Sanyasi R. Gaddipati, Gregory A. Tucker, and Grantley W. Lycett. "Null mutants of individual RABA genes impact the proportion of different cell wall components in stem tissue of Arabidopsis thaliana." PLoS One 8, no. 10 (2013): e75724.
Solyc12g039030.1	-1.97	photosystem II protein D1	psbA	Photosynthetic electron transporter in photosystem II	*Impaired photosynthesis*	Tsunoyama, Yuichi, Yoko Ishizaki, Kazuya Morikawa, Maki Kobori, Yoichi Nakahira, Go Takeba, Yoshinori Toyoshima, and Takashi Shiina. "Blue light-induced transcription of plastid-encoded psbD gene is mediated by a nuclear-encoded transcription initiation factor, AtSig5." Proceedings of the National Academy of Sciences 101, no. 9 (2004): 3304-3309.
Solyc01g060085.1	-1.96	N/A	N/A	N/A	N/A	N/A
Solyc01g017050.1	-1.95	N/A	N/A	N/A	N/A	N/A

TomatoID	DE	NCBI Protein Name	Gene ID	Uniprot Description	Putative Consequences for Infection	Citation
**Solyc05g016120.2**	-1.95	photosystem II protein D1	psbA	Photosynthetic electron transporter in photosystem II	**Impaired photosynthesis**	Tsunoyama, Yuichi, Yoko Ishizaki, Kazuya Morikawa, Maki Kobori, Yoichi Nakahira, Go Takeba, Yoshinori Toyoshima, and Takashi Shiina. "Blue light-induced transcription of plastid-encoded psbD gene is mediated by a nuclear-encoded transcription initiation factor, AtSig5." Proceedings of the National Academy of Sciences 101, no. 9 (2004): 3304-3309.
Solyc11g039860.2	-1.95	N/A	N/A	N/A	N/A	N/A
Solyc00g011150.1	-1.94	N/A	N/A	N/A	N/A	N/A
Solyc01g048590.2	-1.93	photosystem II protein D1	psbA	Photosynthetic electron transporter in photosystem II	*Impaired photosynthesis*	Tsunoyama, Yuichi, Yoko Ishizaki, Kazuya Morikawa, Maki Kobori, Yoichi Nakahira, Go Takeba, Yoshinori Toyoshima, and Takashi Shiina. "Blue light-induced transcription of plastid-encoded psbD gene is mediated by a nuclear-encoded transcription initiation factor, AtSig5." Proceedings of the National Academy of Sciences 101, no. 9 (2004): 3304-3309.
Solyc09g008320.3	-1.92	probable xyloglucan endotransglucosylase/hydrolase protein 32	XTH32	Cleaves and religates xyloglucan polymers, an essential constituent of the primary cell wall, and thereby participates in cell wall construction of growing tissues	*Impaired plant cell wall construction*	Bischoff, Volker, Sarah Jane Cookson, Shuang Wu, and Wolf-Rüdiger Scheible. "Thaxtomin A affects CESA-complex density, expression of cell wall genes, cell wall composition, and causes ectopic lignification in Arabidopsis thaliana seedlings." Journal of experimental botany 60, no. 3 (2009): 955-965.
Solyc03g081240.3	-1.92	two-component response regulator-like APRR5	APRR5	Transcriptional repressor of CCA1 and LHY, thereby controlling photoperiodic flowering response; Involved in the positive and negative feedback loops of the circadian clock	Impaired photoperiodic flowering response; Disrupted circadian clock	Matsushika, Akinori, Seiya Makino, Masaya Kojima, and Takeshi Mizuno. "Circadian waves of expression of the APRR1/TOC1 family of pseudo-response regulators in Arabidopsis thaliana: insight into the plant circadian clock." Plant and Cell Physiology 41, no. 9 (2000): 1002-1012.
Solyc05g005550.3	-1.92	polygalacturonase non-catalytic subunit AroGP2 precursor	GP2	Non-catalytic subunit of polygalacturonase involved in cell wall organization	*Impaired plant cell wall organization*	N/A
Solyc07g048090.2	-1.90	fasciclin-like arabinogalactan protein 2	FLA2	Cell surface adhesion protein	N/A	Sibout, Richard, Aymerick Eudes, Gregory Mouille, Brigitte Pollet, Catherine Lapierre, Lise Jouanin, and Armand Séguin. "CINNAMYL ALCOHOL DEHYDROGENASE-C and-D are the primary genes involved in lignin biosynthesis in the floral stem of Arabidopsis." The Plant Cell 17, no. 7 (2005): 2059-2076.
Solyc07g048085.1	-1.90	N/A	N/A	N/A	N/A	N/A
Solyc01g097000.3	-1.89	uncharacterized protein LOC101254239	N/A	N/A	N/A	N/A

TomatoID	DE	NCBI Protein Name	Gene ID	Uniprot Description	Putative Consequences for Infection	Citation
Solyc08g063090.2	-1.89	acyl-lipid (9-3)-desaturase	DES6	Required for the biosynthesis of arachidonic acid	Impaired arachidonic acid biosynthesis	Girke, Thomas, Hermann Schmidt, Ulrich Zähringer, Ralf Reski, and Ernst Heinz. "Identification of a novel D6-acyl-group desaturase by targeted gene disruption in Physcomitrella patens." The Plant Journal 15, no. 1 (1998): 39-48.
Solyc04g054990.3	-1.88	PLAT domain-containing protein 2	PLAT2	Involved in response to abiotic stress	*Impaired response to abiotic stress*	Giacomelli, Lisa, Andrea Rudella, and Klaas Jan van Wijk. "High light response of the thylakoid proteome in Arabidopsis wild type and the ascorbate-deficient mutant vtc2-2. A comparative proteomics study." Plant Physiology 141.2 (2006): 685-701.
Solyc02g076740.2	-1.88	N/A	N/A	N/A	N/A	N/A
Solyc04g063210.3	-1.88	probable caffeoyl-CoA O-methyltransferase At4g26220 isoform X2	AT4G26220	Plays a role in the synthesis of feruloylated polysaccharides; Involved in the reinforcement of the plant cell wall; Involved in response to wounding and/or pathogen challenge	*Impaired reinforcement of plant cell walls; Impaired response to wounding and/or pathogen challenge*	Meyermans, Hugo, et al. "Modifications in lignin and accumulation of phenolic glucosides in poplar xylem upon down-regulation of caffeoyl-coenzyme A O-methyltransferase, an enzyme involved in lignin biosynthesis." Journal of Biological Chemistry 275.47 (2000): 36899-36909.
Solyc01g058500.3	-1.87	uncharacterized protein LOC101254183	N/A	N/A	N/A	N/A
Solyc00g009760.2	-1.87	uncharacterized protein LOC101246232	N/A	N/A	N/A	N/A
Solyc02g076750.2	-1.86	low-temperature-induced cysteine proteinase-like	N/A	Proteolysis involved in cellular protein catabolic process	Impaired protein catabolism	Bar-Ziv, Amalia, Yael Levy, Hagit Hak, Anahit Mett, Eduard Belausov, Vitaly Citovsky, and Yedidya Gafni. "The Tomato yellow leaf curl virus (TYLCV) V2 protein interacts with the host papain-like cysteine protease CYP1." Plant signaling & behavior 7, no. 8 (2012): 983-989.
Solyc00g012430.1	-1.86	N/A	N/A	N/A	N/A	N/A
Solyc00g012440.1	-1.86	uncharacterized protein LOC101246232	N/A	N/A	N/A	N/A
Solyc06g024210.2	-1.86	uncharacterized protein LOC101254183	N/A	N/A	N/A	N/A
Solyc00g068970.2	-1.85	uncharacterized protein LOC101246232	N/A	N/A	N/A	N/A
Solyc11g027760.1	-1.85	uncharacterized protein LOC104645327	N/A	N/A	N/A	N/A
Solyc00g011160.2	-1.85	N/A	N/A	N/A	N/A	N/A

TomatoID	DE	NCBI Protein Name	Gene ID	Uniprot Description	Putative Consequences for Infection	Citation
Solyc10g052470.1	-1.85	SANT/MYB domain protein	At4g02210	Has DNA binding activity	N/A	Jen, Chih-Hung, Ioannis Michalopoulos, David R. Westhead, and Peter Meyer. "Natural antisense transcripts with coding capacity in Arabidopsis may have a regulatory role that is not linked to double-stranded RNA degradation." Genome biology 6, no. 6 (2005): 1-10.
Solyc06g051940.3	-1.85	protein phosphatase 2C	PP2CA	Major negative regulator of abscisic acid responses during seed germination and cold acclimation; Prevents stomata closure	*Impaired abscisic acid signaling responses; Promoted stomatal opening*	Chérel, Isabelle, et al. "Physical and functional interaction of the Arabidopsis K+ channel AKT2 and phosphatase AtPP2CA." The Plant Cell 14.5 (2002): 1133-1146.
Solyc11g019910.1	-1.84	pectinesterase inhibitor 4	PMEI4	Pectin methylesterase inhibitor that can target the root-expressed PME17; Regulates de-methylesterification of pectins in roots and affects root growth	*Impaired root growth*	Sénéchal, Fabien, Alain Mareck, Paulo Marcelo, Patrice Lerouge, and Jérôme Pelloux. "Arabidopsis PME17 activity can be controlled by pectin methylesterase inhibitor4." Plant signaling & behavior 10, no. 2 (2015): e983351.
Solyc02g070540.3	-1.84	carbohydrate binding domain-containing protein precursor	N/A	N/A	N/A	N/A
Solyc00g008580.1	-1.84	N/A	N/A	N/A	N/A	N/A
Solyc11g051200.1	-1.82	N/A	N/A	N/A	N/A	N/A
Solyc03g118120.3	-1.81	uncharacterized protein At4g14100	N/A	N/A	N/A	N/A
Solyc11g027770.1	-1.81	N/A	N/A	N/A	N/A	N/A
Solyc06g050315.1	-1.81	N/A	N/A	N/A	N/A	N/A
Solyc06g050320.3	-1.81	high mobility group B protein 7 isoform X1	HMGB7	Required for karyogamy during female gametophyte development, when the two polar nuclei fuse to form the diploid central cell nucleus	*Impaired female gametophyte development*	Grasser, Klaus D., Simon Grill, Meg Duroux, Dorte Launholt, Malene S. Thomsen, Birthe V. Nielsen, Hanne K. Nielsen, and Thomas Merkle. "HMGB6 from Arabidopsis thaliana specifies a novel type of plant chromosomal HMGB protein." Biochemistry 43, no. 5 (2004): 1309-1314.
Solyc10g048060.1	-1.80	N/A	N/A	N/A	N/A	N/A
Solyc11g044620.1	-1.80	N/A	N/A	N/A	N/A	N/A
Solyc11g056340.1	-1.80	photosystem II protein D1	psbA	Photosynthetic electron transporter in photosystem II	*Impaired photosynthesis*	Tsunoyama, Yuichi, Yoko Ishizaki, Kazuya Morikawa, Maki Kobori, Yoichi Nakahira, Go Takeba, Yoshinori Toyoshima, and Takashi Shiina. "Blue light-induced transcription of plastid-encoded psbD gene is mediated by a nuclear-encoded transcription initiation factor, AtSig5." Proceedings of the National Academy of Sciences 101, no. 9 (2004): 3304-3309.
Solyc07g063733.1	-1.79	N/A	N/A	N/A	N/A	N/A

TomatoID	DE	NCBI Protein Name	Gene ID	Uniprot Description	Putative Consequences for Infection	Citation
Solyc12g005430.1	-1.79	acetyl-CoA-benzylalcohol acetyltransferase	At3g30280	Has transferase activity	N/A	Cheng, Chia-Yi, Vivek Krishnakumar, Agnes P. Chan, Françoise Thibaud-Nissen, Seth Schobel, and Christopher D. Town. "Araport11: a complete reannotation of the Arabidopsis thaliana reference genome." The Plant Journal 89, no. 4 (2017): 789-804.
Solyc10g078920.2	-1.77	thioredoxin-like 3-1, chloroplastic	WCRKC1	Thiol-disulfide oxidoreductase that participates in various redox reactions	N/A	Cain, Peter, Michael Hall, Wolfgang P. Schröder, Thomas Kieselbach, and Colin Robinson. "A novel extended family of stromal thioredoxins." Plant molecular biology 70, no. 3 (2009): 273-281.
Solyc07g045440.1	-1.76	fasciclin-like arabinogalactan protein 2	FLA2	Cell surface adhesion protein	N/A	Sibout, Richard, Aymerick Eudes, Gregory Mouille, Brigitte Pollet, Catherine Lapierre, Lise Jouanin, and Armand Séguin. "CINNAMYL ALCOHOL DEHYDROGENASE-C and-D are the primary genes involved in lignin biosynthesis in the floral stem of Arabidopsis." The Plant Cell 17, no. 7 (2005): 2059-2076.
Solyc00g010525.1	-1.75	N/A	N/A	N/A	N/A	N/A
Solyc02g082260.3	-1.75	3-hydroxy-3-methylglutaryl-coenzyme A reductase 1	HMG1	Catalyzes the synthesis of mevalonate, the specific precursor of all isoprenoid compounds present in plants	*Impaired synthesis of isoprenoids*	Dale, Susan, et al. "Bacterial expression of the catalytic domain of 3–hydroxy-3–methylglutaryl-coa reductase (isoform HMGR1) from Arabidopsis thaliana, and Its inactivation by phosphorylation at Ser577 by Brassica oleracea 3-hydroxy-3-methylglutaryl-CoA reductase kinase." European Journal of Biochemistry 233.2 (1995): 506-513.
Solyc04g081890.1	-1.75	E3 ubiquitin-protein ligase ATL23	ATL23	E3 ubiquitin-protein ligase able to catalyze polyubiquitination with ubiquitin-conjugating enzymes	*Impaired E3 ubiquitination; Accumulation of misfolded proteins*	Kraft, E., Stone, S.L., Ma, L., Su, N., Gao, Y., Lau, O.S., Deng, X.W. and Callis, J., 2005. Genome analysis and functional characterization of the E2 and RING-type E3 ligase ubiquitination enzymes of Arabidopsis. Plant physiology, 139(4), pp.1597-1611.
Solyc01g111570.3	-1.75	probable receptor-like serine/threonine-protein kinase At5g57670	At5g57670	Has protein serine and threonine kinase activity	Impaired serine and threonine kinase activities	Dal Bosco, Cristina, Lina Lezhneva, Alexander Biehl, Dario Leister, Heinrich Strotmann, Gerd Wanner, and Jörg Meurer. "Inactivation of the chloroplast ATP synthase γ subunit results in high non-photochemical fluorescence quenching and altered nuclear gene expression in Arabidopsis thaliana." Journal of Biological Chemistry 279, no. 2 (2004): 1060-1069.
Solyc12g057060.2	-1.75	7-deoxyloganetin glucosyltransferase-like	N/A	Iridoid glucosyltransferase acting exclusively on 7-deoxyloganetin; Involved in the synthesis of secologanin	Increassed synthesis of secologanin	Asada, Keisuke, et al. "A 7-deoxyloganetic acid glucosyltransferase contributes a key step in secologanin biosynthesis in Madagascar periwinkle." The Plant Cell 25.10 (2013): 4123-4134.
Solyc05g014000.3	-1.73	probable pectate lyase 5	At1g67750	Part of the pathway pectin degradation, which is itself part of Glycan metabolism	*Impaired pectin degradation*	Pischke, Melissa S., Edward L. Huttlin, Adrian D. Hegeman, and Michael R. Sussman. "A transcriptome-based characterization of habituation in plant tissue culture." Plant Physiology 140, no. 4 (2006): 1255-1278.

TomatoID	DE	NCBI Protein Name	Gene ID	Uniprot Description	Putative Consequences for Infection	Citation
Solyc02g066970.1	-1.72	transcription factor PAR1	PAR1	Negative regulator of a variety of shade avoidance syndrome responses, including seedling elongation and photosynthetic pigment accumulation; Transcriptional repressor of two auxin-responsive genes	*Impaired shade avoidance; Impaired repression of auxin signaling*	Roig-Villanova, Irma, Jordi Bou-Torrent, Anahit Galstyan, Lorenzo Carretero-Paulet, Sergi Portolés, Manuel Rodríguez-Concepción, and Jaime F. Martínez-García. "Interaction of shade avoidance and auxin responses: a role for two novel atypical bHLH proteins." The EMBO journal 26, no. 22 (2007): 4756-4767.
Solyc10g074540.1	-1.72	protein EXORDIUM-like 5	EXL5	Plays a role in a brassinosteroid-dependent regulation of growth and development	*Impaired growth and development*	Krinke, Ondrej, Eric Ruelland, Olga Valentová, Chantal Vergnolle, Jean-Pierre Renou, Ludivine Taconnat, Matyás Flemr, Lenka Burketová, and Alain Zachowski. "Phosphatidylinositol 4-kinase activation is an early response to salicylic acid in Arabidopsis suspension cells." Plant Physiology 144, no. 3 (2007): 1347-1359.
Solyc01g098740.3	-1.71	probable serine/threonine-protein kinase PBL7	PBL7	Serine/threonine-protein kinase involved in the positive regulation of brassinosteroid signaling and plant growth	*Impaired brassinosteroid signaling; Impaired growth*	Kim, Tae-Wuk, Shenheng Guan, Alma L. Burlingame, and Zhi-Yong Wang. "The CDG1 kinase mediates brassinosteroid signal transduction from BRI1 receptor kinase to BSU1 phosphatase and GSK3-like kinase BIN2." Molecular cell 43, no. 4 (2011): 561-571.
Solyc01g096450.3	-1.71	aspartyl protease AED3	AED3	Involved in regulation of programmed cell death; Involved in systemic acquired resistance	*Impaired programmed cell death; Impaired systemic acquired resistance*	Dinneny, Jose R., Terri A. Long, Jean Y. Wang, Jee W. Jung, Daniel Mace, Solomon Pointer, Christa Barron, Siobhan M. Brady, John Schiefelbein, and Philip N. Benfey. "Cell identity mediates the response of Arabidopsis roots to abiotic stress." Science 320, no. 5878 (2008): 942-945.
Solyc05g009470.3	-1.70	alpha-xylosidase 1	XYL1	Glycoside hydrolase releasing xylosyl residues from xyloglucan oligosaccharides; Essential for growth/development	*Impaired growth/development*	Sampedro, Javier, et al. "Cloning and expression pattern of a gene encoding an α-xylosidase active against xyloglucan oligosaccharides from Arabidopsis." Plant Physiology 126.2 (2001): 910-920.
Solyc09g007940.3	-1.70	adenosine kinase 2	ADK2	Essential to sustain methyl recycling	*Impaired methyl recycling*	Pereira, L. A. R., M. Todorova, X. Cai, C. A. Makaroff, R. J. N. Emery, and B. A. Moffatt. "Methyl recycling activities are co-ordinately regulated during plant development." Journal of experimental botany 58, no. 5 (2007): 1083-1098.
Solyc04g081300.3	-1.70	endoglucanase 2	At1g19940	Involved in cellulose catabolism; Involved in cell wall organization	*Impaired cellulose catabolism; Impaired cell wall organization*	Brown, David M., Leo AH Zeef, Joanne Ellis, Royston Goodacre, and Simon R. Turner. "Identification of novel genes in Arabidopsis involved in secondary cell wall formation using expression profiling and reverse genetics." The Plant Cell 17, no. 8 (2005): 2281-2295.
Solyc07g045080.3	-1.69	2-alkenal reductase (NADP(+)-dependent)-like	DBR	Reduces the C=C double bonds of alpha, beta unsaturated enones	N/A	Hirata, Toshifumi, Akihito Matsushima, Yuya Sato, Toshihiko Iwasaki, Hidetaka Nomura, Takayoshi Watanabe, Saki Toyoda, and Shunsuke Izumi. "Stereospecific hydrogenation of the CC double bond of enones by Escherichia coli overexpressing an enone reductase of Nicotiana tabacum." Journal of Molecular Catalysis B: Enzymatic 59, no. 1-3 (2009): 158-162.

TomatoID	DE	NCBI Protein Name	Gene ID	Uniprot Description	Putative Consequences for Infection	Citation
Solyc04g055250.3	-1.68	uncharacterized protein LOC101258942	N/A	N/A	N/A	N/A
Solyc01g005295.1	-1.67	N/A	N/A	N/A	N/A	N/A
Solyc08g079680.3	-1.67	uncharacterized protein LOC101259562	N/A	N/A	N/A	N/A
Solyc04g082030.1	-1.66	ornithine decarboxylase	ODC	Catalyzes the first and rate-limiting step of polyamine biosynthesis that converts ornithine into putrescine	Impaired production of polyamines, spermidine, and spermine	Alabadı, David, and Juan Carbonell. "Expression of ornithine decarboxylase is transiently increased by pollination, 2, 4-dichlorophenoxyacetic acid, and gibberellic acid in tomato ovaries." Plant physiology 118.1 (1998): 323-328.
Solyc01g096040.3	-1.65	aspartyl protease family protein 2	APF2	Involved in response to karrikin, a positive plant growth regulator	*Impaired plant growth*	Li, Yurong, Mehdi Kabbage, Wende Liu, and Martin B. Dickman. "Aspartyl protease-mediated cleavage of BAG6 is necessary for autophagy and fungal resistance in plants." The Plant Cell 28, no. 1 (2016): 233-247.
Solyc04g078520.3	-1.64	protein RICE SALT SENSITIVE 3-like	RSS3	Represses jasmonate-induced genes; Involved in transcriptional regulation in the root tip; Regulates root cell elongation during salt stress	*Impaired repression of jasmonate-induced genes; Inceased jasmonate-related defense; Impaired root cell elongation*	Toda, Yosuke, Maiko Tanaka, Daisuke Ogawa, Kyo Kurata, Ken-ichi Kurotani, Yoshiki Habu, Tsuyu Ando et al. "RICE SALT SENSITIVE3 forms a ternary complex with JAZ and class-C bHLH factors and regulates jasmonate-induced gene expression and root cell elongation." The Plant Cell 25, no. 5 (2013): 1709-1725.
Solyc03g096050.3	-1.63	probable 2-oxoglutarate-dependent dioxygenase At5g05600	AT5G05600	Involved in anthocyanin and protoanthocyanidin biosynthesis	*Impaired anthocyanin and protoanthocyanidin biosynthesis*	Wang, Yi, et al. "Transcriptome analyses show changes in gene expression to accompany pollen germination and tube growth in Arabidopsis." Plant physiology 148.3 (2008): 1201-1211.
Solyc03g113060.3	-1.61	ABC transporter A family member 7 isoform X1	ABCA7	Involved in lipid transport	*Impaired lipid transport*	Badri, D.V., Loyola-Vargas, V.M., Broeckling, C.D., De-la-Peña, C., Jasinski, M., Santelia, D., Martinoia, E., Sumner, L.W., Banta, L.M., Stermitz, F. and Vivanco, J.M., 2008. Altered profile of secondary metabolites in the root exudates of Arabidopsis ATP-binding cassette transporter mutants. Plant Physiology, 146(2), pp.323-324.
Solyc03g005900.3	-1.61	GDSL esterase/lipase At5g45670-like	At5g45670	Involved in lipid catabolic process	Impaired lipid catabolism	Rouhier, Nicolas, Arsenio Villarejo, Manoj Srivastava, Eric Gelhaye, Olivier Keech, Michel Droux, Iris Finkemeier et al. "Identification of plant glutaredoxin targets." Antioxidants & redox signaling 7, no. 7-8 (2005): 919-929.
Solyc01g104400.3	-1.61	basic blue protein	ARPN	Forms a concentration gradient along the pollen tube growth path	*Impaired pollen tube growth*	Dong, Juan, Sun Tae Kim, and Elizabeth M. Lord. "Plantacyanin plays a role in reproduction in Arabidopsis." Plant Physiology 138, no. 2 (2005): 778-789.
Solyc10g047430.1	-1.61	ribulose 1,5-bisphosphate carboxylase, partial	RBCMT	Methylates 'Lys-14' of the large subunit of RuBisCO	*Impaired RuBisCO activity*	N/A

TomatoID	DE	NCBI Protein Name	Gene ID	Uniprot Description	Putative Consequences for Infection	Citation
Solyc12g005020.2	-1.60	NEP1-interacting protein-like 1 isoform X1	ATL27	Involved in the early steps of the plant defense signaling pathway	*Impaired plant defense*	Libault, Marc, Jinrong Wan, Tomasz Czechowski, Michael Udvardi, and Gary Stacey. "Identification of 118 Arabidopsis transcription factor and 30 ubiquitin-ligase genes responding to chitin, a plant-defense elicitor." Molecular plant-microbe interactions 20, no. 8 (2007): 900-911.
Solyc03g117560.3	-1.59	lamin-like protein	At5g15350	Has electron transfer activity; Involved in early response to salicylic acid	*Impaired salicylic acid-induced defense*	Krinke, Ondrej, Eric Ruelland, Olga Valentová, Chantal Vergnolle, Jean-Pierre Renou, Ludivine Taconnat, Matyás Flemr, Lenka Burketová, and Alain Zachowski. "Phosphatidylinositol 4-kinase activation is an early response to salicylic acid in Arabidopsis suspension cells." Plant Physiology 144, no. 3 (2007): 1347-1359.
Solyc07g008390.2	-1.58	salutaridinol 7-O-acetyltransferase-like	LOC103494518	Has transferase activity	N/A	Garcia-Mas, Jordi, Andrej Benjak, Walter Sanseverino, Michael Bourgeois, Gisela Mir, Víctor M. González, Elizabeth Hénaff et al. "The genome of melon (Cucumis melo L.)." Proceedings of the National Academy of Sciences 109, no. 29 (2012): 11872-11877.
Solyc05g005760.3	-1.58	uncharacterized protein LOC101255990 isoform X1	N/A	N/A	N/A	N/A
Solyc12g044310.2	-1.57	protein NRT1/ PTR FAMILY 1.2	NPF1.2	Low-affinity nitrate transporter involved in xylem-to-phloem transfer for redistributing nitrate into developing leaves	*Impaired nitrate transport; Impaired growth/development*	Hsu, Po-Kai, and Yi-Fang Tsay. "Two phloem nitrate transporters, NRT1. 11 and NRT1. 12, are important for redistributing xylem-borne nitrate to enhance plant growth." Plant Physiology 163, no. 2 (2013): 844-856.
Solyc09g008860.3	-1.56	leucine-rich repeat receptor-like protein kinase PXC1	PXC1	Involved in secondary cell wall formation in xylem fibers; Regulates interfascicular fiber cell maturation; Promotes secondary wall formation during cell expansion	*Impaired xylem growth; Impaired fiber cell maturation; Impaired cell expansion*	Wang, Jiehua, Melis Kucukoglu, Linbin Zhang, Peng Chen, Daniel Decker, Ove Nilsson, Brian Jones, Göran Sandberg, and Bo Zheng. "The Arabidopsis LRR-RLK, PXC1, is a regulator of secondary wall formation correlated with the TDIF-PXY/TDR-WOX4 signaling pathway." BMC plant biology 13, no. 1 (2013): 1-11.
Solyc06g083050.3	-1.56	serine carboxypeptidase-like 20	SCPL20	Carboxypeptidase	N/A	N/A
Solyc03g025730.3	-1.54	tubulin beta-1 chain	TUBB1	Tubulin is the major constituent of microtubules	*Impaired microtubule development*	Cao, Dongni, Hui Cheng, Wei Wu, Hui Meng Soo, and Jinrong Peng. "Gibberellin mobilizes distinct DELLA-dependent transcriptomes to regulate seed germination and floral development in Arabidopsis." Plant physiology 142, no. 2 (2006): 509-525.
Solyc02g090990.1	-1.54	mitogen-activated protein kinase kinase kinase 17-like	MAPKKK17	Component of the abscisic aci signaling pathway that acts as a signal transducer under abiotic stress	*Impaired response to abioitic stress*	Danquah, Agyemang, Axel de Zélicourt, Marie Boudsocq, Jorinde Neubauer, Nicolas Frei dit Frey, Nathalie Leonhardt, Stephanie Pateyron et al. "Identification and characterization of an ABA-activated MAP kinase cascade in Arabidopsis thaliana." The Plant Journal 82, no. 2 (2015): 232-244.

TomatoID	DE	NCBI Protein Name	Gene ID	Uniprot Description	Putative Consequences for Infection	Citation
Solyc08g007240.3	-1.53	nudix hydrolase 8 isoform X2	NUDT8	Mediates the hydrolysis of some nucleoside diphosphate derivatives; Positive regulator of defense response through salicylic acid signaling	*Impaired salicylic acid-induced defense*	Fonseca, Jose Pedro, and Xinnian Dong. "Functional characterization of a Nudix hydrolase AtNUDX8 upon pathogen attack indicates a positive role in plant immune responses." PloS one 9, no. 12 (2014): e114119.
Solyc11g063520.1	-1.52	dof zinc finger protein DOF2.1-like	DOF2.1	Transcription regulator	N/A	Riechmann, Jose Luis, J. Heard, G. Martin, L. Reuber, C-Z. Jiang, J. Keddie, L. Adam et al. "Arabidopsis transcription factors: genome-wide comparative analysis among eukaryotes." science 290, no. 5499 (2000): 2105-2110.
Solyc08g067890.3	-1.52	eukaryotic translation initiation factor 5B	At1g76720	Has translation initiation factor activity	N/A	Cheng, Chia-Yi, Vivek Krishnakumar, Agnes P. Chan, Françoise Thibaud-Nissen, Seth Schobel, and Christopher D. Town. "Araport11: a complete reannotation of the Arabidopsis thaliana reference genome." The Plant Journal 89, no. 4 (2017): 789-804.
Solyc02g080160.3	-1.51	probable xyloglucan endotransglucosylase/hydrolase protein 8 isoform X2	XTH8	Cleaves and religates xyloglucan polymers, an essential constituent of the primary cell wall, and thereby participates in cell wall construction of growing tissues	*Impaired plant cell wall growth/development*	Bischoff, Volker, Sarah Jane Cookson, Shuang Wu, and Wolf-Rüdiger Scheible. "Thaxtomin A affects CESA-complex density, expression of cell wall genes, cell wall composition, and causes ectopic lignification in Arabidopsis thaliana seedlings." Journal of experimental botany 60, no. 3 (2009): 955-965.
Solyc06g065820.3	-1.51	ethylene response factor H.1	ERF1A	Acts as a transcriptional activator; Involved in the regulation of gene expression by stress factors and by components of stress signal transduction pathways	*Impaired stress response*	Fujimoto, Susan Y., Masaru Ohta, Akemi Usui, Hideaki Shinshi, and Masaru Ohme-Takagi. "Arabidopsis ethylene-responsive element binding factors act as transcriptional activators or repressors of GCC box–mediated gene expression." The Plant Cell 12, no. 3 (2000): 393-404.
Solyc06g061200.1	-1.51	glycine-rich protein TomR2	TomR2	N/A	N/A	Lin, Wan-Chi, et al. "A glycine-rich protein gene family predominantly expressed in tomato roots, but not in leaves and ripe fruit." Plant science 168.2 (2005): 283-295.
Solyc05g054090.3	-1.50	induced stolen tip protein TUB8-like	N/A	N/A	N/A	N/A
Solyc08g042100.3	-1.50	U-box domain-containing protein 3-like	PUB3	Functions as an E3 ubiquitin ligase	*Impaired E3 ubiquitination; Accumulation of misfolded proteins*	Benschop, Joris J., Shabaz Mohammed, Martina O'Flaherty, Albert JR Heck, Monique Slijper, and Frank LH Menke. "Quantitative phosphoproteomics of early elicitor signaling in Arabidopsis." Molecular & Cellular Proteomics 6, no. 7 (2007): 1198-1214.
Solyc04g055207.1	-1.49	N/A	N/A	N/A	N/A	N/A
Solyc06g084460.3	-1.49	bifunctional 3-dehydroquinate dehydratase/shikimate dehydrogenase, chloroplastic	EMB3004	Involved in aromatic amino acid and chorismate biosynthesis; Involved in embryo development; Necessary for shikimate matabolism	*Impaired aromatic aminod acid and chorismate biosynthesis; Impaired embryo development; Impaired shikimate metabolism*	Singh, Sasha Anna, and Dinesh Christendat. "Structure of Arabidopsis dehydroquinate dehydratase-shikimate dehydrogenase and implications for metabolic channeling in the shikimate pathway." Biochemistry 45, no. 25 (2006): 7787-7796.
Solyc06g008990.1	-1.49	protein FANTASTIC FOUR 1-like	FAF1	Represses WUS, a gene related to plant organ development, when constitutively overexpressed	*Impaired growth and development*	Wahl, Vanessa, Luise H. Brand, Ya-Long Guo, and Markus Schmid. "The FANTASTIC FOUR proteins influence shoot meristem size in Arabidopsis thaliana." BMC Plant Biology 10, no. 1 (2010): 1-12.

TomatoID	DE	NCBI Protein Name	Gene ID	Uniprot Description	Putative Consequences for Infection	Citation
Solyc11g027690.1	-1.48	N/A	N/A	N/A	N/A	N/A
Solyc10g085550.2	-1.48	enolase	At1g56230	Has enolase activity	N/A	Ortega-Amaro, María A., Aída A. Rodríguez-Hernández, Margarita Rodríguez-Kessler, Eloísa Hernández-Lucero, Sergio Rosales-Mendoza, Alejandro Ibáñez-Salazar, Pablo Delgado-Sánchez, and Juan F. Jiménez-Bremont. "Overexpression of AtGRDP2, a novel glycine-rich domain protein, accelerates plant growth and improves stress tolerance." Frontiers in plant science 5 (2015): 782.
Solyc01g067510.3	-1.47	proline-rich receptor-like protein kinase PERK15 isoform X1	PERK15	Has protein serine/threonine kinase activity	Impaired serine and threonine kinase activities	Nakhamchik, Alina, Zhiying Zhao, Nicholas J. Provart, Shin-Han Shiu, Sarah K. Keatley, Robin K. Cameron, and Daphne R. Goring. "A comprehensive expression analysis of the Arabidopsis proline-rich extensin-like receptor kinase gene family using bioinformatic and experimental approaches." Plant and Cell Physiology 45, no. 12 (2004): 1875-1881.
Solyc03g080100.3	-1.47	heavy metal-associated isoprenylated plant protein 9-like	HIPP09	Heavy-metal-binding protein	N/A	Zhang, Ping, Ruling Wang, Qiong Ju, Weiqiang Li, Lam-Son Phan Tran, and Jin Xu. "The R2R3-MYB transcription factor MYB49 regulates cadmium accumulation." Plant physiology 180, no. 1 (2019): 529-542.
Solyc07g062260.3	-1.47	BES1/BZR1 homolog protein 4	BEH4	Involved in brassinosteroid mediated signaling pathway	*Impaired plant development and physiological regulation*	Wang, Zhi-Yong, Takeshi Nakano, Joshua Gendron, Junxian He, Meng Chen, Dionne Vafeados, Yanli Yang et al. "Nuclear-localized BZR1 mediates brassinosteroid-induced growth and feedback suppression of brassinosteroid biosynthesis." Developmental cell 2, no. 4 (2002): 505-513.
Solyc02g079790.3	-1.46	uncharacterized protein LOC101268247 isoform X2	N/A	N/A	N/A	N/A
Solyc04g016200.1	-1.46	zeatin O-glucosyltransferase	ZOG1	Regulates active vs storage forms of cytokinins	*Impaired seed development*	Hou, Bingkai, Eng-Kiat Lim, Gillian S. Higgins, and Dianna J. Bowles. "N-glucosylation of cytokinins by glycosyltransferases of Arabidopsis thaliana." Journal of Biological Chemistry 279, no. 46 (2004): 47822-47832.
Solyc10g080940.2	-1.46	tubulin beta-5 chain	TUBB5	Tubulin is the major constituent of microtubules	*Impaired microtubule development*	Cao, Dongni, Hui Cheng, Wei Wu, Hui Meng Soo, and Jinrong Peng. "Gibberellin mobilizes distinct DELLA-dependent transcriptomes to regulate seed germination and floral development in Arabidopsis." Plant physiology 142, no. 2 (2006): 509-525.
Solyc12g027550.1	-1.45	photosystem II CP43 chlorophyll apoprotein	psbC	Photosynthetic electron transporter in photosystem II	*Impaired photosynthesis*	Xu, Hong, Dmitrii Vavilin, and Wim Vermaas. "Chlorophyll b can serve as the major pigment in functional photosystem II complexes of cyanobacteria." Proceedings of the National Academy of Sciences 98, no. 24 (2001): 14168-14173.
Solyc11g045150.1	-1.45	N/A	N/A	N/A	N/A	N/A

**TomatoID**	**DE**	**NCBI Protein Name**	**Gene ID**	**Uniprot Description**	**Putative Consequences for Infection**	**Citation**
Solyc06g051680.1	-1.45	protein EARLY FLOWERING 4	ELF4	Component of the central CCA1/LHY-TOC1 feedback loop in the circadian clock; Controls flowering time	*Impaired circadian rhythm; Impaired flowering time*	Doyle, Mark R., Seth J. Davis, Ruth M. Bastow, Harriet G. McWatters, László Kozma-Bognár, Ferenc Nagy, Andrew J. Millar, and Richard M. Amasino. "The ELF4 gene controls circadian rhythms and flowering time in Arabidopsis thaliana." Nature 419, no. 6902 (2002): 74-77.
Solyc11g056250.1	-1.44	N/A	N/A	N/A	N/A	N/A
Solyc11g008080.2	-1.44	uncharacterized protein LOC101263962	N/A	N/A	N/A	N/A
Solyc08g066610.3	-1.43	uncharacterized protein LOC101250825	N/A	N/A	N/A	N/A
Solyc06g074463.1	-1.42	N/A	N/A	N/A	N/A	N/A
Solyc04g008850.1	-1.42	protein ASPARTIC PROTEASE IN GUARD CELL 2-like	ASPG2	Aspartic protease involved in drought avoidance through abscisic acid signaling	*Impaired drought response; Impaired abscisic acid signaling*	Yao, Xuan, Wei Xiong, Tiantian Ye, and Yan Wu. "Overexpression of the aspartic protease ASPG1 gene confers drought avoidance in Arabidopsis." Journal of experimental botany 63, no. 7 (2012): 2579-2593.
Solyc06g076570.3	-1.41	class I small heat shock protein	HSP17.8	Cytosolic mediator for sorting and targeting of nascent chloroplast outer envelope membrane proteins to the chloroplast	*Impaired chloroplast transport*	Kim, Dae Heon, Zheng-Yi Xu, Yun Jeong Na, Yun-Joo Yoo, Junho Lee, Eun-Ju Sohn, and Inhwan Hwang. "Small heat shock protein Hsp17. 8 functions as an AKR2A cofactor in the targeting of chloroplast outer membrane proteins in Arabidopsis." Plant physiology 157, no. 1 (2011): 132-146.
Solyc04g050730.3	-1.41	GDSL-motif esterase/acyltransferase/lipase precursor	At1g29670	Involved in lipid catabolism	Impaired lipid catabolism	Thieme, Christoph J., Monica Rojas-Triana, Ewelina Stecyk, Christian Schudoma, Wenna Zhang, Lei Yang, Miguel Miñambres et al. "Endogenous Arabidopsis messenger RNAs transported to distant tissues." Nature Plants 1, no. 4 (2015): 1-9.
Solyc05g005560.4	-1.41	polygalacturonase-1 non-catalytic subunit beta precursor	GP2	Non-catalytic subunit of polygalacturonase involved in cell wall organization	*Impaired plant cell wall organization*	N/A
Solyc03g123630.3	-1.41	pectinesterase/pectinesterase inhibitor U1 precursor	PMEU1	Acts in the modification of cell walls via demethylesterification of cell wall pectin	*Impaired plant cell wall modification/development*	Phan, Thanh D., Wen Bo, Gill West, Grantley W. Lycett, and Gregory A. Tucker. "Silencing of the major salt-dependent isoform of pectinesterase in tomato alters fruit softening." Plant physiology 144, no. 4 (2007): 1960-1967.
Solyc01g110340.3	-1.41	endoglucanase 24-like	At4g39010	Involved in fruit dehiscence and fruit valve development; Involved in plant cell wall loosening	*Impaired fruit development*	Urbanowicz, Breeanna R., Alan B. Bennett, Elena Del Campillo, Carmen Catalá, Takahisa Hayashi, Bernard Henrissat, Herman Höfte et al. "Structural organization and a standardized nomenclature for plant endo-1, 4-β-glucanases (cellulases) of glycosyl hydrolase family 9." Plant Physiology 144, no. 4 (2007): 1693-1696.

TomatoID	DE	NCBI Protein Name	Gene ID	Uniprot Description	Putative Consequences for Infection	Citation
Solyc06g034390.1	-1.40	protein EXORDIUM-like 3	EXL3	Plays a role in a brassinosteroid-dependent regulation of growth and development	*Impaired growth and development*	Krinke, Ondrej, Eric Ruelland, Olga Valentová, Chantal Vergnolle, Jean-Pierre Renou, Ludivine Taconnat, Matyás Flemr, Lenka Burketová, and Alain Zachowski. "Phosphatidylinositol 4-kinase activation is an early response to salicylic acid in Arabidopsis suspension cells." Plant Physiology 144, no. 3 (2007): 1347-1359.
Solyc07g052980.3	-1.39	xyloglucan endotransglycosylase/hydrolase 16 precursor	XTH16	Cleaves and religates xyloglucan polymers, an essential constituent of the primary cell wall, and thereby participates in cell wall construction of growing tissues	*Impaired plant cell wall expansion*	Sasidharan, Rashmi, C. C. Chinnappa, Marten Staal, J. Theo M. Elzenga, Ryusuke Yokoyama, Kazuhiko Nishitani, Laurentius ACJ Voesenek, and Ronald Pierik. "Light quality-mediated petiole elongation in Arabidopsis during shade avoidance involves cell wall modification by xyloglucan endotransglucosylase/hydrolases." Plant physiology 154, no. 2 (2010): 978-990.
Solyc04g015620.3	-1.39	uncharacterized protein LOC101245049	N/A	N/A	N/A	N/A
Solyc12g006120.2	-1.38	nuclear transcription factor Y subunit B-3-like	NFYB3	Component of the NF-Y/HAP transcription factor complex, which stimulates transcription	Impaired transcription	Zhang, Zhong-Wei, Ling-Yang Feng, Jian Cheng, He Tang, Fei Xu, Feng Zhu, Zhong-Yi Zhao et al. "The roles of two transcription factors, ABI4 and CBFA, in ABA and plastid signalling and stress responses." Plant molecular biology 83, no. 4-5 (2013): 445-458.
Solyc09g097890.2	-1.37	cytochrome b561 and DOMON domain-containing protein At3g25290	At3g25290	Catecholamine-responsive trans-membrane electron transporter	*Impaired transmembrane transport*	Verelst, Wim, and Han Asard. "Analysis of an Arabidopsis thaliana protein family, structurally related to cytochromes b 561 and potentially involved in catecholamine biochemistry in plants." Journal of plant physiology 161, no. 2 (2004): 175-181.
Solyc05g010630.3	-1.36	uncharacterized protein LOC101255791	N/A	N/A	N/A	N/A
Solyc02g090960.1	-1.36	protein RALF-like 34	RALFL34	Cell signaling peptide that regulates plant stress, growth, and development	*Impaired response to plant stress, growth, and development*	Murphy, Evan, Lam Dai Vu, Lisa Van den Broeck, Zhefeng Lin, Priya Ramakrishna, Brigitte Van De Cotte, Allison Gaudinier et al. "RALFL34 regulates formative cell divisions in Arabidopsis pericycle during lateral root initiation." Journal of Experimental Botany 67, no. 16 (2016): 4863-4875.
Solyc06g062960.2	-1.35	protein RALF-like 34	RALFL34	Cell signaling peptide that may regulate plant stress, growth, and development	*Impaired response to plant stress, growth, and development*	Murphy, Evan, Lam Dai Vu, Lisa Van den Broeck, Zhefeng Lin, Priya Ramakrishna, Brigitte Van De Cotte, Allison Gaudinier et al. "RALFL34 regulates formative cell divisions in Arabidopsis pericycle during lateral root initiation." Journal of Experimental Botany 67, no. 16 (2016): 4863-4875.
Solyc09g009010.3	-1.35	glucomannan 4-beta-mannosyltransferase 9-like isoform X2	CSLA9	Required for lateral root development; Involved in cell wall organization; Involved in response to bacteria	*Impaired lateral root development; Impaired plant cell wall organization; Impaired defense response to bacteria*	Liepman, Aaron H., C. Joseph Nairn, William GT Willats, Iben Sørensen, Alison W. Roberts, and Kenneth Keegstra. "Functional genomic analysis supports conservation of function among cellulose synthase-like a gene family members and suggests diverse roles of mannans in plants." Plant Physiology 143, no. 4 (2007): 1881-1893.

TomatoID	DE	NCBI Protein Name	Gene ID	Uniprot Description	Putative Consequences for Infection	Citation
Solyc09g008990.3	-1.35	glucomannan 4-beta-mannosyltransferase 9-like isoform X2	CSLA9	Required for lateral root development; Involved in cell wall organization; Involved in response to bacteria	*Impaired lateral root development; Impaired plant cell wall organization; Impaired defense response to bacteria*	Liepman, Aaron H., C. Joseph Nairn, William GT Willats, Iben Sørensen, Alison W. Roberts, and Kenneth Keegstra. "Functional genomic analysis supports conservation of function among cellulose synthase-like a gene family members and suggests diverse roles of mannans in plants." Plant Physiology 143, no. 4 (2007): 1881-1893.
Solyc03g093410.3	-1.35	hexose carrier protein HEX6	HEX6	Involved in uptake of hexoses; Symporter activity	*Impaired hexose uptake*	Weig, Alfons, Juliane Franz, Norbert Sauer, and Ewald Komor. "Isolation of a family of cDNA clones from Ricinus communis L. with close homology to the hexose carriers." Journal of Plant Physiology 143, no. 2 (1994): 178-183.
Solyc02g078850.1	-1.34	glycine-rich cell wall structural protein 1.8	At3g17050	Responsible for plasticity of plant cell wall	*Impaired plant cell wall modification/development*	Quigley, Françoise, Marie-Louise Villiot, and Régis Mache. "Nucleotide sequence and expression of a novel glycine-rich protein gene from Arabidopsis thaliana." Plant molecular biology 17, no. 4 (1991): 949-952.
Solyc12g099260.2	-1.34	ATP-citrate synthase beta chain protein 2-like	ACLA-2	Used for the elongation of fatty acids and biosynthesis of isoprenoids, flavonoids, and malonated derivatives; Required for normal growth and development in seeds	*Impaired seed development; Impaired production of primary metabolites*	Fatland, Beth L., Basil J. Nikolau, and Eve Syrkin Wurtele. "Reverse genetic characterization of cytosolic acetyl-CoA generation by ATP-citrate lyase in Arabidopsis." The Plant Cell 17.1 (2005): 182-203.
Solyc09g014610.3	-1.34	S-type anion channel SLAH2-like	LOC107824438	Involved in transmembrane transport	*Impaired transmembrane transport*	Sierro, Nicolas, James ND Battey, Sonia Ouadi, Nicolas Bakaher, Lucien Bovet, Adrian Willig, Simon Goepfert, Manuel C. Peitsch, and Nikolai V. Ivanov. "The tobacco genome sequence and its comparison with those of tomato and potato." Nature communications 5, no. 1 (2014): 1-9.
Solyc06g075010.3	-1.34	ruBisCO large subunit-binding protein subunit alpha, chloroplastic	N/A	Involved in protein refolding	N/A	N/A
Solyc08g014130.3	-1.33	isopropylmalate synthase	N/A	N/A	N/A	N/A
Solyc11g066720.2	-1.33	UDP-D-apiose/UDP-D-xylose synthase 2	AXS2	Catalyzes the conversion of UDP-D-glucuronate to a mixture of UDP-D-apiose and UDP-D-xylose, a plant cell wall monosaccharide with a unique role	*Impaired plant cell wall modification/development*	Mølhøj, Michael, Rajeev Verma, and Wolf-Dieter Reiter. "The biosynthesis of the branched-chain sugar d-apiose in plants: functional cloning and characterization of a UDP-d-apiose/UDP-d-xylose synthase from Arabidopsis." The Plant Journal 35, no. 6 (2003): 693-703.
Solyc02g081060.3	-1.32	chaperonin-like RBCX protein 1, chloroplastic	RBCX1	Chaperone involved in RuBisCO assembly process	*Impaired RuBisCO activity*	Kolesiński, Piotr, Janusz Piechota, and Andrzej Szczepaniak. "Initial characteristics of RbcX proteins from Arabidopsis thaliana." Plant molecular biology 77, no. 4 (2011): 447-459.

TomatoID	DE	NCBI Protein Name	Gene ID	Uniprot Description	Putative Consequences for Infection	Citation
Solyc10g061830.2	-1.32	photosystem II 44 kDa protein	psbC	Photosynthetic electron transporter in photosystem II	*Impaired photosynthesis*	Ahmed, Ibrar, Peter J. Matthews, Patrick J. Biggs, Muhammad Naeem, Patricia A. McLenachan, and Peter J. Lockhart. "Identification of chloroplast genome loci suitable for high-resolution phylogeographic studies of C olocasia esculenta (L.) S chott (A raceae) and closely related taxa." Molecular Ecology Resources 13, no. 5 (2013): 929-937.
Solyc02g080635.1	-1.32	N/A	N/A	N/A	N/A	N/A
Solyc03g113720.3	-1.32	two-component response regulator ARR15	ARR15	Functions as response regulator involved in His-to-Asp phosphorelay signal transduction system; Negative regulator of the cytokinin signaling	*Impaired regulation of cytokinin signaling*	Kiba, Takatoshi, Hisami Yamada, and Takeshi Mizuno. "Characterization of the ARR15 and ARR16 response regulators with special reference to the cytokinin signaling pathway mediated by the AHK4 histidine kinase in roots of Arabidopsis thaliana." Plant and Cell Physiology 43, no. 9 (2002): 1059-1066.
Solyc02g083040.1	-1.31	uncharacterized protein LOC104645768	N/A	N/A	N/A	N/A
Solyc12g089330.2	-1.30	synaptotagmin-1	SYT1	Maintains plasma membrane integrity during freezing and osmotic stresses; Functions in membrane resealing during calcium-dependent freezing tolerance; Regulates endocytosis and endosome recycling at the plasma membrane and cell-to-cell trafficking	*Impaired freezing response; Impaired cell-to-cell trafficking*	Yamazaki, Tomokazu, Naoki Takata, Matsuo Uemura, and Yukio Kawamura. "Arabidopsis synaptotagmin SYT1, a type I signal-anchor protein, requires tandem C2 domains for delivery to the plasma membrane." Journal of Biological Chemistry 285, no. 30 (2010): 23165-23176.
Solyc11g063500.1	-1.30	N/A	N/A	N/A	N/A	N/A
Solyc08g082250.3	-1.29	endo-beta-1,4-D-glucanase precursor	At3g13560	Involved in carbohydrate metabolism; Involved in defense response	*Impaired carbohydrate metabolism; Impaired defense response*	Jakoby, Marc J., Doris Falkenhan, Michael T. Mader, Ginger Brininstool, Elisabeth Wischnitzki, Nicole Platz, Andrew Hudson, Martin Hulskamp, John Larkin, and Arp Schnittger. "Transcriptional profiling of mature Arabidopsis trichomes reveals that NOECK encodes the MIXTA-like transcriptional regulator MYB106." Plant Physiology 148, no. 3 (2008): 1583-1602.
Solyc11g071640.2	-1.28	uncharacterized protein LOC101256554 isoform X2	N/A	N/A	N/A	N/A
Solyc04g077020.3	-1.28	tubulin alpha chain	TUBA1	Tubulin is the major constituent of microtubules	*Impaired microtubule development*	Saito, Yuka, Kouichi Soga, Kazuyuki Wakabayashi, and Takayuki Hoson. "Increase in expression level of alpha-tubulin gene in Arabidopsis seedlings under hypergravity conditions." Uchu Seibutsu Kagaku 17, no. 3 (2003): 177-178.

TomatoID	DE	NCBI Protein Name	Gene ID	Uniprot Description	Putative Consequences for Infection	Citation
Solyc02g030480.3	-1.28	probable cinnamyl alcohol dehydrogenase 6	CAD6	Involved in lignin biosynthesis	*Impaired lignin biosynthesis; Impaired plant cell wall growth/development*	Costa, Michael A., R. Eric Collins, Aldwin M. Anterola, Fiona C. Cochrane, Laurence B. Davin, and Norman G. Lewis. "An in silico assessment of gene function and organization of the phenylpropanoid pathway metabolic networks in Arabidopsis thaliana and limitations thereof." Phytochemistry 64, no. 6 (2003): 1097-1112.
Solyc01g111350.3	-1.28	protein NUCLEAR FUSION DEFECTIVE 4	NFD4	Required for karyogamy during female gametophyte development	*Impaired female gametophyte development*	Portereiko, Michael F., Linda Sandaklie-Nikolova, Alan Lloyd, Chad A. Dever, Denichiro Otsuga, and Gary N. Drews. "NUCLEAR FUSION DEFECTIVE1 encodes the Arabidopsis RPL21M protein and is required for karyogamy during female gametophyte development and fertilization." Plant physiology 141, no. 3 (2006): 957-965.
Solyc02g065170.3	-1.27	L-ascorbate oxidase homolog	AAO	Represses responses to high salinity and oxidative stress conditions such as vegetative growth and seed production reductions	*Impaired regulation of stress responses; Impaired growth/development under stress*	Yamamoto, Atsuko, Md Nazmul H. Bhuiyan, Rungaroon Waditee, Yoshito Tanaka, Muneharu Esaka, Kazuko Oba, André T. Jagendorf, and Teruhiro Takabe. "Suppressed expression of the apoplastic ascorbate oxidase gene increases salt tolerance in tobacco and Arabidopsis plants." Journal of Experimental Botany 56, no. 417 (2005): 1785-1796.
Solyc11g071460.2	-1.27	short-chain dehydrogenase/reductase 2b isoform X3	SDR2b	Aldehyde reductase that catalyzes the reduction of the aldehyde carbonyl groups on saturated and alpha,beta-unsaturated aldehydes with more than 5 carbons	N/A	Yamauchi, Yasuo, Ayaka Hasegawa, Ai Taninaka, Masaharu Mizutani, and Yukihiro Sugimoto. "NADPH-dependent reductases involved in the detoxification of reactive carbonyls in plants." Journal of biological chemistry 286, no. 9 (2011): 6999-7009.
Solyc04g053000.1	-1.27	auxin-responsive protein SAUR21-like	SAUR21	Positive effectors of cell expansion through modulation of auxin transport	*Impaired plant cell wall expansion; Impaired auxin transport*	Osakabe, Yuriko, Kyonoshin Maruyama, Motoaki Seki, Masakazu Satou, Kazuo Shinozaki, and Kazuko Yamaguchi-Shinozaki. "Leucine-rich repeat receptor-like kinase1 is a key membrane-bound regulator of abscisic acid early signaling in Arabidopsis." The Plant Cell 17, no. 4 (2005): 1105-1119.
Solyc03g112880.1	-1.27	fasciclin-like arabinogalactan protein 4	FLA4	Cell surface adhesion protein required for normal cell expansion	*Impaired cell expansion*	Xu, Shou-Ling, et al. "Two leucine-rich repeat receptor kinases mediate signaling, linking cell wall biosynthesis and ACC synthase in Arabidopsis." The Plant Cell 20.11 (2008): 3065-3079.
Solyc05g005710.3	-1.26	spermidine synthase	SPDS1	Involved in polyamine biosynthetic process	*Impaired polyamine biosynthesis; Impaired cell proliferation*	Cheng, Chia-Yi, Vivek Krishnakumar, Agnes P. Chan, Françoise Thibaud-Nissen, Seth Schobel, and Christopher D. Town. "Araport11: a complete reannotation of the Arabidopsis thaliana reference genome." The Plant Journal 89, no. 4 (2017): 789-804.
Solyc04g078810.3	-1.25	uncharacterized protein LOC101252176 isoform X1	N/A	N/A	N/A	N/A

TomatoID	DE	NCBI Protein Name	Gene ID	Uniprot Description	Putative Consequences for Infection	Citation
Solyc01g087785.1	-1.25	N/A	N/A	N/A	N/A	N/A
Solyc01g102330.3	-1.24	probable carbohydrate esterase At4g34215	At4g34215	Involved in secondary plant cell wall biosynthesis	*Impaired secondary plant cell wall biosynthesis*	Andersson-Gunnerås, Sara, Ewa J. Mellerowicz, Jonathan Love, Bo Segerman, Yasunori Ohmiya, Pedro M. Coutinho, Peter Nilsson, Bernard Henrissat, Thomas Moritz, and Björn Sundberg. "Biosynthesis of cellulose-enriched tension wood in Populus: global analysis of transcripts and metabolites identifies biochemical and developmental regulators in secondary wall biosynthesis." The Plant Journal 45, no. 2 (2006): 144-165.
Solyc02g063030.3	-1.24	uncharacterized protein LOC101260301	N/A	N/A	N/A	N/A
Solyc00g028960.1	-1.23	monothiol glutaredoxin-S1-like	GRXS1	Reduces GSH-thiol disulfides	Impaired reduction of GSH-thiol disulfides	Patterson, Kurt, et al. "Nitrate-regulated glutaredoxins control Arabidopsis primary root growth." Plant Physiology 170.2 (2016): 989-999.
Solyc11g021360.2	-1.22	uncharacterized protein LOC101250021	N/A	N/A	N/A	N/A
Solyc02g087190.1	-1.22	peroxidase 63	PER63	Involved in oxidation of toxic reductants, lignin metabolism, suberization, auxin catabolism; Involved in response to environmental stresses	*Impaired plant defense; Impaired lignin metabolism; Impaired auxin catabolism*	Valério, Luisa, Mireille De Meyer, Claude Penel, and Christophe Dunand. "Expression analysis of the Arabidopsis peroxidase multigenic family." Phytochemistry 65, no. 10 (2004): 1331-1342.
Solyc09g014490.3	-1.22	endochitinase A	At2g43590	Involved in plant cell wall macromolecule catabolism and chitin catabolism; Involved in defense response	*Impaired plant cell wall development; Impaired defense response*	Krysan, Patrick J., Peter J. Jester, Jennifer R. Gottwald, and Michael R. Sussman. "An Arabidopsis mitogen-activated protein kinase kinase kinase gene family encodes essential positive regulators of cytokinesis." The Plant Cell 14, no. 5 (2002): 1109-1120.
Solyc03g063480.2	-1.20	N/A	N/A	N/A	N/A	N/A
Solyc03g044150.3	-1.20	subtilisin-like protease SBT1.7	SBT1.7	Serine protease essential for mucilage release from seed coats	*Impaired seed coat development*	Rautengarten, Carsten, Björn Usadel, Lutz Neumetzler, Jürgen Hartmann, Dirk Büssis, and Thomas Altmann. "A subtilisin-like serine protease essential for mucilage release from Arabidopsis seed coats." The Plant Journal 54, no. 3 (2008): 466-480.
Solyc12g009110.2	-1.20	acetylserotonin O-methyltransferase	ASMT	Methyltransferase which catalyzes the production of melatonin; Involved in response to light stress	*Impaired development under light stress*	Shi, Haitao, Yunxie Wei, and Chaozu He. "Melatonin-induced CBF/DREB1s are essential for diurnal change of disease resistance and CCA1 expression in Arabidopsis." Plant Physiology and Biochemistry 100 (2016): 150-155.
Solyc07g008900.3	-1.20	subtilisin-like protease SBT2.5	SBT2.5	Has serine-type endopeptidase activity	N/A	Ascencio-Ibánez, José Trinidad, Rosangela Sozzani, Tae-Jin Lee, Tzu-Ming Chu, Russell D. Wolfinger, Rino Cella, and Linda Hanley-Bowdoin. "Global analysis of Arabidopsis gene expression uncovers a complex array of changes impacting pathogen response and cell cycle during geminivirus infection." Plant physiology 148, no. 1 (2008): 436-454.

TomatoID	DE	NCBI Protein Name	Gene ID	Uniprot Description	Putative Consequences for Infection	Citation
Solyc08g067840.3	-1.19	uncharacterized protein LOC101245936	N/A	N/A	N/A	N/A
Solyc05g054320.3	-1.19	uncharacterized protein LOC101268576	N/A	N/A	N/A	N/A
Solyc10g011960.2	-1.19	heavy metal-associated isoprenylated plant protein 39-like	HIPP39	Heavy-metal-binding protein	N/A	Hanada, Kousuke, Yuji Sawada, Takashi Kuromori, Romy Klausnitzer, Kazuki Saito, Tetsuro Toyoda, Kazuo Shinozaki, Wen-Hsiung Li, and Masami Yokota Hirai. "Functional compensation of primary and secondary metabolites by duplicate genes in Arabidopsis thaliana." Molecular biology and evolution 28, no. 1 (2011): 377-382.
Solyc04g017720.3	-1.18	protein GAST1-like	N/A	Involved in the gibberellic acid mediated signaling pathway	*Impaired gibberellic acid mediated signaling*	Haas, Brian J., Natalia Volfovsky, Christopher D. Town, Maxim Troukhan, Nickolai Alexandrov, Kenneth A. Feldmann, Richard B. Flavell, Owen White, and Steven L. Salzberg. "Full-length messenger RNA sequences greatly improve genome annotation." Genome biology 3, no. 6 (2002): 1-12.
Solyc10g017940.1	-1.18	photosystem II 44 kDa protein	psbC	Photosynthetic electron transporter in photosystem II	*Impaired photosynthesis*	Ahmed, Ibrar, Peter J. Matthews, Patrick J. Biggs, Muhammad Naeem, Patricia A. McLenachan, and Peter J. Lockhart. "Identification of chloroplast genome loci suitable for high-resolution phylogeographic studies of C olocasia esculenta (L.) S chott (A raceae) and closely related taxa." Molecular Ecology Resources 13, no. 5 (2013): 929-937.
Solyc08g065420.3	-1.18	homeobox protein ATH1	ATH1	Transcription factor; Controls floral competency as a specific activator of FLC expression; Responds to import of SHOOT MERISTEMLESS	Impaired floral competency	Gómez-Mena, Concepción, and Robert Sablowski. "ARABIDOPSIS THALIANA HOMEOBOX GENE1 establishes the basal boundaries of shoot organs and controls stem growth." The Plant Cell 20, no. 8 (2008): 2059-2072.
Solyc06g075000.3	-1.17	uncharacterized protein LOC101265549	N/A	N/A	N/A	N/A
Solyc10g055770.2	-1.15	N/A	N/A	N/A	N/A	N/A
Solyc11g066820.2	-1.14	glucomannan 4-beta-mannosyltransferase 2	CSLA2	Possesses glucomannan synthase and mannan synthase activities; Galactomannan is a noncellulosic polysaccharides of plant cell wall	*Impaired plant cell wall growth/development*	Liepman, Aaron H., Curtis G. Wilkerson, and Kenneth Keegstra. "Expression of cellulose synthase-like (Csl) genes in insect cells reveals that CslA family members encode mannan synthases." Proceedings of the National Academy of Sciences 102, no. 6 (2005): 2221-2226.
Solyc03g043620.1	-1.14	N/A	N/A	N/A	N/A	N/A
Solyc12g038080.1	-1.14	photosystem II CP43 chlorophyll apoprotein	psbC	Photosynthetic electron transporter in photosystem II	*Impaired photosynthesis*	Xu, Hong, Dmitrii Vavilin, and Wim Vermaas. "Chlorophyll b can serve as the major pigment in functional photosystem II complexes of cyanobacteria." Proceedings of the National Academy of Sciences 98, no. 24 (2001): 14168-14173.

TomatoID	DE	NCBI Protein Name	Gene ID	Uniprot Description	Putative Consequences for Infection	Citation
Solyc10g081810.2	-1.14	putative phosphatidylglycerol/phosphatidylinositol transfer protein DDB_G0278295	DDB_G0278295	Involved in sterol transport	*Impaired sterol transport*	Sillo, Alessio, Gareth Bloomfield, Alessandra Balest, Alessandra Balbo, Barbara Pergolizzi, Barbara Peracino, Jason Skelton, Alasdair Ivens, and Salvatore Bozzaro. "Genome-wide transcriptional changes induced by phagocytosis or growth on bacteria in Dictyostelium." BMC genomics 9, no. 1 (2008): 1-22.
Solyc01g006370.3	-1.14	callose synthase 3-like	CALS3	Involved in callose synthesis at the forming cell plate during cytokinesis	*Impaired callose synthesis; Impaired cell proliferation*	Chen, Xiong-Yan, Lin Liu, EunKyoung Lee, Xiao Han, Yeonggil Rim, Hyosub Chu, Seon-Won Kim, Fred Sack, and Jae-Yean Kim. "The Arabidopsis callose synthase gene GSL8 is required for cytokinesis and cell patterning." Plant Physiology 150, no. 1 (2009): 105-113.
Solyc11g011020.2	-1.14	probable inactive receptor kinase At1g48480	RKL1	Has protein kinase activity	N/A	Colette, A., Zoltán Bochdanovits, Vera MA Jansweijer, Fenne G. Koning, Lidija Berke, Gabino F. Sanchez-Perez, Ben Scheres, and Renze Heidstra. "Probing the roles of LRR RLK genes in Arabidopsis thaliana roots using a custom T-DNA insertion set." Plant molecular biology 76, no. 1-2 (2011): 69-83.
Solyc12g010590.2	-1.12	O-acyltransferase WSD1-like	At2g38995	Diacylglycerol O-acyltransferase activity	N/A	Li-Beisson, Yonghua, Basil Shorrosh, Fred Beisson, Mats X. Andersson, Vincent Arondel, Philip D. Bates, Sébastien Baud et al. "Acyl-lipid metabolism." The Arabidopsis book/American Society of Plant Biologists 11 (2013).
Solyc10g086650.1	-1.12	aldehyde oxidase GLOX	GLOX1	Regulated by the transcription factor MYB80 during anther development and plays a role in tapetum and pollen development	*Impaired pollen development*	Phan, Huy Anh, Sylvana Iacuone, Song F. Li, and Roger W. Parish. "The MYB80 transcription factor is required for pollen development and the regulation of tapetal programmed cell death in Arabidopsis thaliana." The Plant Cell 23, no. 6 (2011): 2209-2224.
Solyc07g055950.3	-1.12	protodermal factor 1	PDF1	Involved in the regulation of meristem growth	*Impaired growth and development*	Pholo, Motlalepula, Beatrix Coetzee, Hans J. Maree, Philip R. Young, James R. Lloyd, Jens Kossmann, and Paul N. Hills. "Cell division and turgor mediate enhanced plant growth in Arabidopsis plants treated with the bacterial signalling molecule lumichrome." Planta 248, no. 2 (2018): 477-488.
Solyc09g055940.3	-1.12	probable sodium/metabolite cotransporter BASS3, chloroplastic	BASS3	Functions as sodium-coupled metabolite transporter across the chloroplast envelope	*Impaired chloroplast transport*	Hanada, Kousuke, Yuji Sawada, Takashi Kuromori, Romy Klausnitzer, Kazuki Saito, Tetsuro Toyoda, Kazuo Shinozaki, Wen-Hsiung Li, and Masami Yokota Hirai. "Functional compensation of primary and secondary metabolites by duplicate genes in Arabidopsis thaliana." Molecular biology and evolution 28, no. 1 (2011): 377-382.
Solyc03g115380.2	-1.12	UDP-glucose 6-dehydrogenase 1-like	UGD1	Involved in the biosynthesis of UDP-glucuronic acid, providing nucleotide sugars for cell-wall polymers	*Impaired nucleotide transport for cell-wall polymers*	Oka, Takuji, and Yoshifumi Jigami. "Reconstruction of de novo pathway for synthesis of UDP-glucuronic acid and UDP-xylose from intrinsic UDP-glucose in Saccharomyces cerevisiae." The FEBS journal 273.12 (2006): 2645-2657.

TomatoID	DE	NCBI Protein Name	Gene ID	Uniprot Description	Putative Consequences for Infection	Citation
Solyc11g013270.2	-1.12	O-fucosyltransferase 10-like	FUT10	Involved in cell wall biosynthesis	*Impaired plant cell wall growth/development*	Sarria, Rodrigo, Tanya A. Wagner, Malcolm A. O'Neill, Ahmed Faik, Curtis G. Wilkerson, Kenneth Keegstra, and Natasha V. Raikhel. "Characterization of a family of Arabidopsis genes related to xyloglucan fucosyltransferase1." Plant Physiology 127, no. 4 (2001): 1595-1606.
Solyc09g091050.3	-1.11	uncharacterized protein LOC101247860	N/A	N/A	N/A	N/A
Solyc04g009790.3	-1.11	uncharacterized protein LOC101251972	N/A	N/A	N/A	N/A
Solyc11g045260.1	-1.11	photosystem II 44 kDa protein	psbC	Photosynthetic electron transporter in photosystem II	*Impaired photosynthesis*	Ahmed, Ibrar, Peter J. Matthews, Patrick J. Biggs, Muhammad Naeem, Patricia A. McLenachan, and Peter J. Lockhart. "Identification of chloroplast genome loci suitable for high-resolution phylogeographic studies of C olocasia esculenta (L.) S chott (A raceae) and closely related taxa." Molecular Ecology Resources 13, no. 5 (2013): 929-937.
Solyc06g009940.1	-1.11	photosystem I P700 apoprotein A1	psaA	saA and PsaB bind P700, the primary electron donor of photosystem I; Necessary for photosynthesis	*Impaired photosynthesis*	Cournac, Laurent, Kevin Redding, Jacques Ravenel, Dominique Rumeau, Eve-Marie Josse, Marcel Kuntz, and Gilles Peltier. "Electron flow between photosystem II and oxygen in chloroplasts of photosystem I-deficient algae is mediated by a quinol oxidase involved in chlororespiration." Journal of Biological Chemistry 275, no. 23 (2000): 17256-17262.
Solyc04g014510.3	-1.10	glutamine synthetase cytosolic isozyme 1-1	GLN1-1	High-affinity glutamine synthetase; Contributes to the homeostatic control of glutamine synthesis in roots	Impaired glutamine homeostasis in the roots	Ishiyama, Keiki, Eri Inoue, Akiko Watanabe-Takahashi, Mitsuhiro Obara, Tomoyuki Yamaya, and Hideki Takahashi. "Kinetic properties and ammonium-dependent regulation of cytosolic isoenzymes of glutamine synthetase in Arabidopsis." Journal of Biological Chemistry 279, no. 16 (2004): 16598-16605.
Solyc04g016480.3	-1.10	protein IQ-DOMAIN 14	IQD14	Involved in cooperative interactions with calmodulins or calmodulin-like proteins; Scaffold in cellular signaling and trafficking; Regulates cell and organ shapes	*Impaired cell-to-cell signaling and trafficking; Impaired regulation of plant organ shape*	Bürstenbinder, Katharina, Birgit Möller, Romina Plötner, Gina Stamm, Gerd Hause, Dipannita Mitra, and Steffen Abel. "The IQD family of calmodulin-binding proteins links calcium signaling to microtubules, membrane subdomains, and the nucleus." Plant physiology 173, no. 3 (2017): 1692-1708.
Solyc06g062580.3	-1.10	beta-galactosidase-like	BGAL1	Involved in carbohydrate metabolic process	*Impaired carbohydrate metabolism*	Cartieaux, Fabienne, Céline Contesto, Adrien Gallou, Guilhem Desbrosses, Joachim Kopka, Ludivine Taconnat, Jean-Pierre Renou, and Bruno Touraine. "Simultaneous interaction of Arabidopsis thaliana with Bradyrhizobium sp. strain ORS278 and Pseudomonas syringae pv. tomato DC3000 leads to complex transcriptome changes." Molecular plant-microbe interactions 21, no. 2 (2008): 244-259.

TomatoID	DE	NCBI Protein Name	Gene ID	Uniprot Description	Putative Consequences for Infection	Citation
Solyc03g120790.1	-1.10	uncharacterized protein LOC104646538	N/A	N/A	N/A	N/A
Solyc12g036650.2	-1.10	uncharacterized protein LOC101258832	N/A	N/A	N/A	N/A
Solyc12g009300.3	-1.10	sucrose synthase	SUS1	Sucrose-cleaving enzyme that provides UDP-glucose and fructose for various metabolic pathways	*Impaired sugar metabolism*	Bieniawska, Zuzanna, D. H. Paul Barratt, Andrew P. Garlick, Vera Thole, Nicholas J. Kruger, Cathie Martin, Rita Zrenner, and Alison M. Smith. "Analysis of the sucrose synthase gene family in Arabidopsis." The Plant Journal 49, no. 5 (2007): 810-828.
Solyc08g075080.3	-1.10	uncharacterized protein LOC101258477 isoform X2	N/A	N/A	N/A	N/A
Solyc10g076790.2	-1.09	auxin transporter-like protein 4	LAX4	Carrier protein involved in proton-driven auxin influx	*Impaired auxin signaling*	de Billy, Françoise, Cathy Grosjean, Sean May, Malcolm Bennett, and Julie V. Cullimore. "Expression studies on AUX1-like genes in Medicago truncatula suggest that auxin is required at two steps in early nodule development." Molecular Plant-Microbe Interactions 14, no. 3 (2001): 267-277.
Solyc01g020285.1	-1.09	N/A	N/A	N/A	N/A	N/A
Solyc06g071500.3	-1.09	probable boron transporter 2	BOR2	Boron transporter essential for maintaining the integrity of plants cell walls	*Impaired plant cell wall growth/development*	Takano, Junpei, Kyotaro Noguchi, Miho Yasumori, Masaharu Kobayashi, Zofia Gajdos, Kyoko Miwa, Hiroaki Hayashi, Tadakatsu Yoneyama, and Toru Fujiwara. "Arabidopsis boron transporter for xylem loading." Nature 420, no. 6913 (2002): 337-340.
Solyc01g106580.2	-1.08	probable pectin methylesterase CGR3	CGR3	Together with CGR2, required for homogalacturonan pectins (HG) methylesterification in the Golgi apparatus prior to integration into cell walls, essential for general growth and development	*Impaired growth and development; Impaired plant cell wall growth/development*	M. Weraduwage, Sarathi, Sang-Jin Kim, Luciana Renna, Fransisca C. Anozie, Thomas D. Sharkey, and Federica Brandizzi. "Pectin methylesterification impacts the relationship between photosynthesis and plant growth." Plant Physiology 171, no. 2 (2016): 833-848.
Solyc06g083650.3	-1.08	GDSL esterase/lipase At5g33370	AT5G33370	Involved in cuticle development; Involved in lipid catabolism	Impaired cuticle development; Impaired lipid catabolism	Li-Beisson, Yonghua, et al. "Acyl-lipid metabolism." The Arabidopsis book/American Society of Plant Biologists 11 (2013).
Solyc08g061930.3	-1.07	cytokinin oxidase/dehydrogenase-like isoform X2	CKX2	Involved in cytokinin catabolism	*Impaired cytokinin catabolism; Impaired plant root/shoot development*	Werner, Tomáš, Václav Motyka, Valérie Laucou, Rafaël Smets, Harry Van Onckelen, and Thomas Schmülling. "Cytokinin-deficient transgenic Arabidopsis plants show multiple developmental alterations indicating opposite functions of cytokinins in the regulation of shoot and root meristem activity." The Plant Cell 15, no. 11 (2003): 2532-2550.

TomatoID	DE	NCBI Protein Name	Gene ID	Uniprot Description	Putative Consequences for Infection	Citation
Solyc01g017740.1	-1.07	cytochrome b6	petB	Component of the cytochrome b6-f complex, which mediates electron transfer between photosystem II and photosystem I	*Impaired photosynthesis*	Felder, Susanne, Karin Meierhoff, Aniruddha P. Sane, Jörg Meurer, Christiane Driemel, Henning Plücken, Petra Klaff, Bernhard Stein, Nicole Bechtold, and Peter Westhoff. "The nucleus-encoded HCF107 gene of Arabidopsis provides a link between intercistronic RNA processing and the accumulation of translation-competent psbH transcripts in chloroplasts." The Plant Cell 13, no. 9 (2001): 2127-2141.
Solyc04g081490.3	-1.07	beta-tubulin	TUBB1	Tubulin is the major constituent of microtubules	*Impaired microtubule development*	Cao, Dongni, Hui Cheng, Wei Wu, Hui Meng Soo, and Jinrong Peng. "Gibberellin mobilizes distinct DELLA-dependent transcriptomes to regulate seed germination and floral development in Arabidopsis." Plant physiology 142, no. 2 (2006): 509-525.
Solyc01g080070.3	-1.06	heavy metal-associated isoprenylated plant protein 33	HIPP33	Heavy-metal-binding protein	N/A	Ascencio-Ibánez, José Trinidad, Rosangela Sozzani, Tae-Jin Lee, Tzu-Ming Chu, Russell D. Wolfinger, Rino Cella, and Linda Hanley-Bowdoin. "Global analysis of Arabidopsis gene expression uncovers a complex array of changes impacting pathogen response and cell cycle during geminivirus infection." Plant physiology 148, no. 1 (2008): 436-454.
Solyc06g068770.3	-1.06	probable beta-1,4-xylosyltransferase	IRX9H	Involved in the synthesis of the hemicellulose glucuronoxylan, a major component of secondary cell walls	*Impaired secondary plant cell wall biosynthesis*	Lee, Chanhui, Quincy Teng, Wenlin Huang, Ruiqin Zhong, and Zheng-Hua Ye. "The Arabidopsis family GT43 glycosyltransferases form two functionally nonredundant groups essential for the elongation of glucuronoxylan backbone." Plant Physiology 153, no. 2 (2010): 526-541.
Solyc04g071990.3	-1.06	protein GIGANTEA	GI	Involved in regulation of circadian rhythm and photoperiodic flowering	*Impaired circadian clock*	Huq, Enamul, James M. Tepperman, and Peter H. Quail. "GIGANTEA is a nuclear protein involved in phytochrome signaling in Arabidopsis." Proceedings of the National Academy of Sciences 97, no. 17 (2000): 9789-9794.
Solyc04g011850.1	-1.05	monothiol glutaredoxin-S1-like	N/A	Has protein disulfide oxidoreductase activity	N/A	Sierro, Nicolas, James ND Battey, Sonia Ouadi, Nicolas Bakaher, Lucien Bovet, Adrian Willig, Simon Goepfert, Manuel C. Peitsch, and Nikolai V. Ivanov. "The tobacco genome sequence and its comparison with those of tomato and potato." Nature communications 5, no. 1 (2014): 1-9.
Solyc03g032040.3	-1.05	monosaccharide-sensing protein 2-like	MSSP2	Involved in carbohydrate transport	*Impaired carbohydrate transport*	Wormit, Alexandra, Oliver Trentmann, Ingmar Feifer, Christian Lohr, Joachim Tjaden, Stefan Meyer, Ulrike Schmidt, Enrico Martinoia, and H. Ekkehard Neuhaus. "Molecular identification and physiological characterization of a novel monosaccharide transporter from Arabidopsis involved in vacuolar sugar transport." The Plant Cell 18, no. 12 (2006): 3476-3490.

TomatoID	DE	NCBI Protein Name	Gene ID	Uniprot Description	Putative Consequences for Infection	Citation
Solyc09g015080.3	-1.05	patellin-6	PATL6	Carrier protein involved in membrane-trafficking associated with cell-plate formation during cytokinesis; Involved in cellular division; Involved in auxin polar transport	*Impaired cellular division; Impaired auxin transport*	Zhou, Huapeng, Hongqin Duan, Yunhong Liu, Xia Sun, Jinfeng Zhao, and Honghui Lin. "Patellin protein family functions in plant development and stress response." Journal of plant physiology 234 (2019): 94-97.
Solyc04g053010.1	-1.04	auxin-responsive protein SAUR68-like	SAUR68	Promote auxin-stimulated organ elongation, such as hypocotyls, stamen filaments and petals	*Impaired organ elongation; Impaired growth and development; Impaired auxin signaling; Impaired flower development*	Zhao, Yunde, Xinhua Dai, Helen E. Blackwell, Stuart L. Schreiber, and Joanne Chory. "SIR1, an upstream component in auxin signaling identified by chemical genetics." Science 301, no. 5636 (2003): 1107-1110.
Solyc03g095500.3	-1.04	thioredoxin H4-1	TRX4	Thiol-disulfide oxidoreductase involved in the redox regulation of a number of cytosolic enzymes	N/A	Yamazaki, Daisuke, Ken Motohashi, Takeshi Kasama, Yukichi Hara, and Toru Hisabori. "Target proteins of the cytosolic thioredoxins in Arabidopsis thaliana." Plant and Cell Physiology 45, no. 1 (2004): 18-27.
Solyc05g012790.3	-1.03	probable protein S-acyltransferase 22	PAT22	Palmitoyl acyltransferase	*Impaired signaling and transport*	Hemsley, Piers, and Claire Grierson. "S-acylation: dynamic control of plant development and sigalling by lipid modification of proteins." In 18th International Conference on Arabidopsis Research (第十八届国际拟南芥大会), pp. 38-38. 中国科学院, 2007.
Solyc06g071330.3	-1.03	xanthine/uracil permease family protein	EF_2430	Has transmembrane transporter activity	N/A	Paulsen, Ian T., L. Banerjei, G. S. A. Myers, K. E. Nelson, Rekha Seshadri, Timothy D. Read, Derrick E. Fouts et al. "Role of mobile DNA in the evolution of vancomycin-resistant Enterococcus faecalis." Science 299, no. 5615 (2003): 2071-2074.
Solyc11g051170.2	-1.02	ATP synthase CF1 alpha subunit	atpA	Produces ATP from ADP in the presence of a proton gradient across the membrane; Necessary for defense response to bacteria and cold	*Impaired defense against pathogens; Impaired cold tolerance*	Kubis, Sybille, Ramesh Patel, Jonathan Combe, Jocelyn Beédard, Sabina Kovacheva, Kathryn Lilley, Alexander Biehl et al. "Functional specialization amongst the Arabidopsis Toc159 family of chloroplast protein import receptors." The Plant Cell 16, no. 8 (2004): 2059-2077.
Solyc11g069270.2	-1.02	beta-galactosidase 5	BGAL5	Involved in carbohydrate metabolic process	*Impaired carbohydrate metabolism*	Gantulga, Dashzeveg, Yusuf Turan, David R. Bevan, and Asim Esen. "The Arabidopsis At1g45130 and At3g52840 genes encode β-galactosidases with activity toward cell wall polysaccharides." Phytochemistry 69, no. 8 (2008): 1661-1670.
Solyc02g067360.3	-1.02	protease Do-like 8, chloroplastic	DEGP8	Serine protease involved in photosystem II repair	*Impaired photosynthesis*	Giacomelli, Lisa, Andrea Rudella, and Klaas Jan van Wijk. "High light response of the thylakoid proteome in Arabidopsis wild type and the ascorbate-deficient mutant vtc2-2. A comparative proteomics study." Plant Physiology 141, no. 2 (2006): 685-701.
Solyc05g012510.3	-1.01	alpha-1,4 glucan phosphorylase L-2 isozyme, chloroplastic/amyloplastic	N/A	Important allosteric enzyme in carbohydrate metabolism	*Impaired carbohydrate metabolism*	Lin, Chi-Tsai, Kai-Wun Yeh, Ping-Du Lee, and Jong-Ching Su. "Primary structure of sweet potato starch phosphorylase deduced from its cDNA sequence." Plant physiology 95, no. 4 (1991): 1250-1253.

TomatoID	DE	NCBI Protein Name	Gene ID	Uniprot Description	Putative Consequences for Infection	Citation
Solyc04g012030.3	-1.01	DHHC-type zinc finger family protein	dhhc-4	Involved in peptidyl-L-cysteine S-palmitoylation	*Impaired signaling and transport*	McKay, S. J., R. Johnsen, J. Khattra, J. Asano, D. L. Baillie, S. Chan, N. Dube et al. "Gene expression profiling of cells, tissues, and developmental stages of the nematode C. elegans." In Cold Spring Harbor symposia on quantitative biology, vol. 68, pp. 159-170. Cold Spring Harbor Laboratory Press, 2003.
Solyc12g037930.2	-1.01	N/A	N/A	N/A	N/A	N/A
Solyc12g008490.2	-1.01	uncharacterized protein LOC101259712 isoform X2	N/A	N/A	N/A	N/A
Solyc09g015170.3	-1.00	probably inactive leucine-rich repeat receptor-like protein kinase IMK2 isoform X2	IMK2	Protein kinase activity	N/A	Shahollari, Bationa, Ajit Varma, and Ralf Oelmüller. "Expression of a receptor kinase in Arabidopsis roots is stimulated by the basidiomycete Piriformospora indica and the protein accumulates in Triton X-100 insoluble plasma membrane microdomains." Journal of plant physiology 162, no. 8 (2005): 945-958.
Solyc02g085870.3	1.00	3-ketoacyl-CoA synthase 6	CUT1	Major condensing enzyme for stem wax and pollen coat lipid biosynthesis	*Increased wax and pollen coat biosynthesis; Cell wall expansion*	Kunst, Ljerka, and A. Lacey Samuels. "Biosynthesis and secretion of plant cuticular wax." Progress in lipid research 42, no. 1 (2003): 51-80.
Solyc05g007900.2	1.01	E3 ubiquitin-protein ligase MPSR1	MPSR1	E3 ubiquitin-protein ligase involved in protein quality control under proteotoxic stress	*Increased E3 ubiquitination during proteotoxic stress*	Kim, Jong Hum, Seok Keun Cho, Tae Rin Oh, Moon Young Ryu, Seong Wook Yang, and Woo Taek Kim. "MPSR1 is a cytoplasmic PQC E3 ligase for eliminating emergent misfolded proteins in Arabidopsis thaliana." Proceedings of the National Academy of Sciences 114, no. 46 (2017): E10009-E10017.
Solyc06g075310.3	1.02	adenylate kinase isoenzyme 6 homolog isoform X1	AAK6	Kinase that catalyzes the reversible transfer of the terminal phosphate group between nucleoside triphosphates and monophosphates	*Promoted stem growth*	Feng, Xue, Ruonan Yang, Xiaofeng Zheng, and FeiYun Zhang. "Identification of a novel nuclear-localized adenylate kinase 6 from Arabidopsis thaliana as an essential stem growth factor." Plant physiology and biochemistry 61 (2012): 180-186.
Solyc06g061240.3	1.04	uncharacterized protein LOC101268004	N/A	N/A	N/A	N/A
Solyc01g007190.3	1.04	uncharacterized protein LOC101262645	N/A	N/A	N/A	N/A
Solyc03g082550.3	1.04	N/A	N/A	N/A	N/A	N/A
Solyc12g099900.1	1.05	scarecrow-like protein 3	SCL3	Transcription factor involved in plant development	*Promoted plant growth and development*	Zhang, Zhong-Lin, Mikihiro Ogawa, Christine M. Fleet, Rodolfo Zentella, Jianhong Hu, Jung-Ok Heo, Jun Lim, Yuji Kamiya, Shinjiro Yamaguchi, and Tai-ping Sun. "Scarecrow-like 3 promotes gibberellin signaling by antagonizing master growth repressor DELLA in Arabidopsis." Proceedings of the National Academy of Sciences 108, no. 5 (2011): 2160-2165.

TomatoID	DE	NCBI Protein Name	Gene ID	Uniprot Description	Putative Consequences for Infection	Citation
Solyc07g043550.3	1.05	UDP-arabinose 4-epimerase 1	MUR4	Acts as a UDP-D-xylose 4-epimerase	N/A	Mølhøj, Michael, Rajeev Verma, and Wolf-Dieter Reiter. "The biosynthesis of D-Galacturonate in plants. functional cloning and characterization of a membrane-anchored UDP-D-Glucuronate 4-epimerase from Arabidopsis." Plant Physiology 135, no. 3 (2004): 1221-1230.
Solyc07g032480.3	1.06	cyclin-T1-3	CYCT1-3	Involved in cellular division; Positive regulator of DNA-templated transcription; Regulates transcription of RNA polymerase II	*Promoted cellular division; Increased DNA-templated transcription*	Cui, Xiaofeng, Baofang Fan, James Scholz, and Zhixiang Chen. "Roles of Arabidopsis cyclin-dependent kinase C complexes in cauliflower mosaic virus infection, plant growth, and development." The Plant Cell 19, no. 4 (2007): 1388-1402.
Solyc02g078380.3	1.07	stem-specific protein TSJT1	TSJT1	N/A	N/A	N/A
Solyc07g053130.3	1.07	G-type lectin S-receptor-like serine/threonine-protein kinase At4g03230	At4g03230	Involved in protein phosphorylation; Involved in pollen recognition	Promotoed pollen recognition	Cheng, Chia-Yi, Vivek Krishnakumar, Agnes P. Chan, Françoise Thibaud-Nissen, Seth Schobel, and Christopher D. Town. "Araport11: a complete reannotation of the Arabidopsis thaliana reference genome." The Plant Journal 89, no. 4 (2017): 789-804.
Solyc02g077080.3	1.07	protein trichome birefringence-like 38	TBL38	Bridging protein that binds pectin and other cell wall polysaccharides	*Promoted pectin-binding; Promoted plant cell wall growth and development*	Bischoff, Volker, Joachim Selbig, and Wolf-Rüdiger Scheible. "Involvement of TBL/DUF231 proteins into cell wall biology." Plant signaling & behavior 5, no. 8 (2010): 1057-1059.
Solyc07g044980.3	1.07	NIM1-like protein 2 isoform X2	N/A	N/A	N/A	N/A
Solyc12g011230.1	1.08	N/A	N/A	N/A	N/A	N/A
Solyc07g006130.2	1.08	eukaryotic peptide chain release factor subunit 1-3-like	ERF1-3	Directs the termination of nascent peptide synthesis; Modulates plant growth and development	*Promoted plant growth and development*	Chapman, Bernice, and Chris Brown. "Translation termination in Arabidopsis thaliana: characterisation of three versions of release factor 1." Gene 341 (2004): 219-225.
Solyc04g074850.3	1.09	putative polyprotein	T8L23.26	Involved in DNA integration	Increased DNA integration	Theologis, Athanasios, Joseph R. Ecker, Curtis J. Palm, Nancy A. Federspiel, Samir Kaul, Owen White, Jose Alonso et al. "Sequence and analysis of chromosome 1 of the plant Arabidopsis thaliana." Nature 408, no. 6814 (2000): 816-820.
Solyc01g086870.3	1.09	transcription factor bHLH130	BHLH130	Involved in photoperiodism, flowering; Transcription regulator	*Promoted photoperiodism*	Takahashi, Yohei, Yuta Ebisu, Toshinori Kinoshita, Michio Doi, Eiji Okuma, Yoshiyuki Murata, and Ken-ichiro Shimazaki. "bHLH transcription factors that facilitate K+ uptake during stomatal opening are repressed by abscisic acid through phosphorylation." Science Signaling 6, no. 280 (2013): ra48-ra48.
Solyc07g017610.3	1.10	alpha-aminoadipic semialdehyde synthase	LKR/SDH	Bifunctional enzyme that catalyzes the first two steps in lysine degradation	Promoted lysine degradation	Tang, Guiliang, Daphna Miron, Judith X. Zhu-Shimoni, and Gad Galili. "Regulation of lysine catabolism through lysine-ketoglutarate reductase and saccharopine dehydrogenase in Arabidopsis." The Plant Cell 9, no. 8 (1997): 1305-1316.

TomatoID	DE	NCBI Protein Name	Gene ID	Uniprot Description	Putative Consequences for Infection	Citation
Solyc05g052050.1	1.10	DNA-binding protein Pti4	Pti4	Transcription factor involved in defense	*Promoted defense*	Gu, Yong-Qiang, Mary C. Wildermuth, Suma Chakravarthy, Ying-Tsu Loh, Caimei Yang, Xiaohua He, Yu Han, and Gregory B. Martin. "Tomato transcription factors Pti4, Pti5, and Pti6 activate defense responses when expressed in Arabidopsis." The Plant Cell 14, no. 4 (2002): 817-831.
Solyc02g084950.3	1.11	jasmonate O-methyltransferase isoform X2	JMT	Catalyzes the methylation of jasmonate into methyljasmonate, a plant volatile that acts as an important cellular regulator mediating diverse developmental processes and defense responses	*Promoted methyljasmonate-regulated signaling; Promoted defenese*	Seo, Hak Soo, Jong Tae Song, Jong-Joo Cheong, Yong-Hwan Lee, Yin-Won Lee, Ingyu Hwang, Jong Seob Lee, and Yang Do Choi. "Jasmonic acid carboxyl methyltransferase: a key enzyme for jasmonate-regulated plant responses." Proceedings of the National Academy of Sciences 98, no. 8 (2001): 4788-4793.
Solyc04g071600.3	1.11	abscisic stress-ripening protein 3-like	ASR3	N/A	N/A	Gilad, A., Amitai-Zeigerson, H., Bar-Zvi, D. and Scolnik, P.A., 1996, June. ASR1, a tomato water-stress regulated gene: genomic organization, developmental regulation and DNA-binding activity. In III International Symposium on In Vitro Culture and Horticultural Breeding 447 (pp. 447-454).
Solyc05g007880.3	1.12	cyclic dof factor 1 isoform X1	CDF1	Regulates a photoperiodic flowering response; Transcriptional repressor of 'CONSTANS' expression	*Promoted photoperiodic flowering response; Increased repression of CONSTANS expression*	Imaizumi, Takato, et al. "FKF1 F-box protein mediates cyclic degradation of a repressor of CONSTANS in Arabidopsis." Science 309.5732 (2005): 293-297.
Solyc01g099910.3	1.12	uncharacterized protein LOC101259675	N/A	N/A	N/A	N/A
Solyc02g084430.3	1.12	B-box zinc finger protein 19	BBX19	Negative regulator of seedling photomorphogenesis	*Promoted seedling photomorphogenesis*	Kumagai, Takeshi, Shogo Ito, Norihito Nakamichi, Yusuke Niwa, Masaya Murakami, Takafumi Yamashino, and Takeshi Mizuno. "The common function of a novel subfamily of B-Box zinc finger proteins with reference to circadian-associated events in Arabidopsis thaliana." Bioscience, biotechnology, and biochemistry 72, no. 6 (2008): 1539-1549.
Solyc10g080670.2	1.13	uncharacterized protein LOC101267365	N/A	N/A	N/A	N/A
Solyc11g066060.2	1.14	heat shock cognate 70 kDa protein 2-like	HSP70-2	Facilitate folding of de novo synthesized proteins, assist translocation of precursor proteins into organelles, and are responsible for degradation of damaged protein under stress conditions	*Promoted folding of de novo synthesized proteins; Promoted protein transport; Promoted degradation of damanged proteins under stress*	Hilson, Pierre, Joke Allemeersch, Thomas Altmann, Sébastien Aubourg, Alexandra Avon, Jim Beynon, Rishikesh P. Bhalerao et al. "Versatile gene-specific sequence tags for Arabidopsis functional genomics: transcript profiling and reverse genetics applications." Genome research 14, no. 10b (2004): 2176-2189.
Solyc05g008110.3	1.14	wiskott-Aldrich syndrome protein family member 2	N/A	N/A	N/A	N/A

TomatoID	DE	NCBI Protein Name	Gene ID	Uniprot Description	Putative Consequences for Infection	Citation
Solyc05g046290.3	1.14	probable xyloglucan endotransglucosylase/hydrolase protein 23	XTH23	Cleaves and religates xyloglucan polymers, an essential constituent of the primary cell wall, and thereby participates in cell wall construction of growing tissues	*Promoted plant growth and development*	Bischoff, Volker, Sarah Jane Cookson, Shuang Wu, and Wolf-Rüdiger Scheible. "Thaxtomin A affects CESA-complex density, expression of cell wall genes, cell wall composition, and causes ectopic lignification in Arabidopsis thaliana seedlings." Journal of experimental botany 60, no. 3 (2009): 955-965.
Solyc01g111640.3	1.14	SKP1-like protein 1A	SKP1A	Involved in proteinubiquitination; Required for vegetative and floral organ development as well as for male gametogenesis; Involved in auxin signaling pathway; Regulates responses to jasmonates; Involved in light-signaling and the circadian clock; Plays a role during embryogenesis	*Promoted protein ubiquitination; Promoted floral development and male gametogenesis; Promoted auxin signaling; Promoted circadian clock; Promoted embryogenesis*	Zhao, Dazhong, Weimin Ni, Baomin Feng, Tianfu Han, Megan G. Petrasek, and Hong Ma. "Members of the Arabidopsis-SKP1-like gene family exhibit a variety of expression patterns and may play diverse roles in Arabidopsis." Plant physiology 133, no. 1 (2003): 203-217.
Solyc11g045465.1	1.14	N/A	N/A	N/A	N/A	N/A
Solyc11g007510.2	1.16	uncharacterized protein LOC101251943	N/A	N/A	N/A	N/A
Solyc02g063360.3	1.16	protein C2-DOMAIN ABA-RELATED 1	CAR1	Mediates the transient calcium-dependent interaction of PYR/PYL/RCAR abscisic acid receptors with the plasma membrane	*Promoted abscisic acid signaling*	Rodriguez, Lesia, Miguel Gonzalez-Guzman, Maira Diaz, Americo Rodrigues, Ana C. Izquierdo-Garcia, Marta Peirats-Llobet, Maria A. Fernandez et al. "C2-domain abscisic acid-related proteins mediate the interaction of PYR/PYL/RCAR abscisic acid receptors with the plasma membrane and regulate abscisic acid sensitivity in Arabidopsis." The Plant Cell 26, no. 12 (2014): 4802-4820.
Solyc12g009560.2	1.18	EIN3-binding F-box protein 1	EBF1	Component of ubiquitin ligase complexes, which mediate the ubiquitination and subsequent proteasomal degradation of target proteins; Regulates ethylene signaling cascade	*Promoted protein ubiquitination*	Gagne, Jennifer M., Jan Smalle, Derek J. Gingerich, Joseph M. Walker, Sang-Dong Yoo, Shuichi Yanagisawa, and Richard D. Vierstra. "Arabidopsis EIN3-binding F-box 1 and 2 form ubiquitin-protein ligases that repress ethylene action and promote growth by directing EIN3 degradation." Proceedings of the National Academy of Sciences 101, no. 17 (2004): 6803-6808.
Solyc12g088370.2	1.18	N/A	N/A	N/A	N/A	N/A
Solyc07g042550.3	1.19	sucrose synthase	SUS1	Sucrose-cleaving enzyme that provides UDP-glucose and fructose for various metabolic pathways	*Promoted sugar metabolism*	Bieniawska, Zuzanna, D. H. Paul Barratt, Andrew P. Garlick, Vera Thole, Nicholas J. Kruger, Cathie Martin, Rita Zrenner, and Alison M. Smith. "Analysis of the sucrose synthase gene family in Arabidopsis." The Plant Journal 49, no. 5 (2007): 810-828.
Solyc08g082740.3	1.19	signal recognition particle 19 kDa protein	SRP19	Part of the signal-recognition-particle assembly, binds directly to 7S RNA and mediates binding of the 54 kDa subunit of the SRP	*Promoted cell-to-cell signaling*	Wang, Yi, Wen-Zheng Zhang, Lian-Fen Song, Jun-Jie Zou, Zhen Su, and Wei-Hua Wu. "Transcriptome analyses show changes in gene expression to accompany pollen germination and tube growth in Arabidopsis." Plant physiology 148, no. 3 (2008): 1201-1211.

TomatoID	DE	NCBI Protein Name	Gene ID	Uniprot Description	Putative Consequences for Infection	Citation
Solyc10g051230.1	1.19	receptor-like protein Cf-9	CF-9	Involved in plant defense against fungal pathogens	*Promoted defense against fungi*	Jones, David A., Colwyn M. Thomas, Kim E. Hammond-Kosack, Peter J. Balint-Kurti, and J. D. Jones. "Isolation of the tomato Cf-9 gene for resistance to Cladosporium fulvum by transposon tagging." Science 266, no. 5186 (1994): 789-793.
Solyc12g015880.2	1.19	molecular chaperone Hsp90-1	N/A	N/A	N/A	N/A
Solyc07g054470.1	1.20	uncharacterized protein LOC101259953	N/A	N/A	N/A	N/A
Solyc07g006590.1	1.20	uncharacterized protein LOC101265142	N/A	N/A	N/A	N/A
Solyc09g092480.1	1.20	crocetin glucosyltransferase, chloroplastic	UGT75L6	UDP-glycosyltransferase activity	N/A	Nagatoshi, Mai, Kazuyoshi Terasaka, Miki Owaki, Makiko Sota, Tatsunori Inukai, Akito Nagatsu, and Hajime Mizukami. "UGT75L6 and UGT94E5 mediate sequential glucosylation of crocetin to crocin in Gardenia jasminoides." FEBS letters 586, no. 7 (2012): 1055-1061.
Solyc04g005610.3	1.21	NAC domain-containing protein 2	N/A	N/A	N/A	N/A
Solyc02g075625.1	1.21	N/A	N/A	N/A	N/A	N/A
Solyc03g114960.3	1.22	tip elongation aberrant protein 1 isoform X2	tea1	Acts as an end marker, directing the growth machinery to the cell poles; Involved in the regulation of microtubular organization, affecting the maintenance of a single central axis	*Promoted cell elongation; Promoted microtubule development*	Mata, Juan, and Paul Nurse. "tea1 and the microtubular cytoskeleton are important for generating global spatial order within the fission yeast cell." Cell 89, no. 6 (1997): 939-949.
Solyc03g116900.3	1.23	metal transporter Nramp6	NRAMP6	Intracellular cadmium transporter that participates in the distribution or availability of Cd within the cell	Promoted Cd homeostasis	Cailliatte, Rémy, Bruno Lapeyre, Jean-François Briat, Stéphane Mari, and Catherine Curie. "The NRAMP6 metal transporter contributes to cadmium toxicity." Biochemical Journal 422, no. 2 (2009): 217-228.
Solyc06g008870.2	1.26	gibberellin receptor GID1b-2	GID1B	Soluble gibberellin GA receptor; Regulates growth and development	*Promoted growth/development*	Griffiths, Jayne, Kohji Murase, Ivo Rieu, Rodolfo Zentella, Zhong-Lin Zhang, Stephen J. Powers, Fan Gong et al. "Genetic characterization and functional analysis of the GID1 gibberellin receptors in Arabidopsis." The Plant Cell 18, no. 12 (2006): 3399-3414.
Solyc07g043310.3	1.27	gamma aminobutyrate transaminase 1, mitochondrial	GABA-TP1	Transaminase that degrades GABA and uses pyruvate or glyoxylate as amino-group acceptor	*Promoted signaling turnover*	Clark, Shawn M., Rosa Di Leo, Owen R. Van Cauwenberghe, Robert T. Mullen, and Barry J. Shelp. "Subcellular localization and expression of multiple tomato γ-aminobutyrate transaminases that utilize both pyruvate and glyoxylate." Journal of experimental botany 60, no. 11 (2009): 3255-3267.
Solyc04g076010.3	1.28	uncharacterized protein LOC101247909	N/A	N/A	N/A	N/A

TomatoID	DE	NCBI Protein Name	Gene ID	Uniprot Description	Putative Consequences for Infection	Citation
Solyc09g075020.3	1.29	ABC transporter C family member 4	ABCC4	Involved in the regulation of stomatal aperture; High-capacity pump for folates	Promoted stomatal aperture regulation	Klein, Markus, Markus Geisler, Su Jeoung Suh, H. Üner Kolukisaoglu, Louis Azevedo, Sonia Plaza, Mark D. Curtis et al. "Disruption of AtMRP4, a guard cell plasma membrane ABCC-type ABC transporter, leads to deregulation of stomatal opening and increased drought susceptibility." The Plant Journal 39, no. 2 (2004): 219-236.
Solyc01g105620.3	1.30	RING-H2 finger protein ATL54-like	LOC109001200	This protein is involved in the pathway protein ubiquitination, which is part of Protein modification	*Promoted protein ubiquitination*	Martínez-García, Pedro J., Marc W. Crepeau, Daniela Puiu, Daniel Gonzalez-Ibeas, Jeanne Whalen, Kristian A. Stevens, Robin Paul et al. "The walnut (Juglans regia) genome sequence reveals diversity in genes coding for the biosynthesis of non-structural polyphenols." The Plant Journal 87, no. 5 (2016): 507-532.
Solyc03g116630.3	1.30	uncharacterized protein LOC101253674	N/A	N/A	N/A	N/A
Solyc12g096130.2	1.31	N/A	N/A	N/A	N/A	N/A
Solyc12g096610.2	1.32	poly(ADP-ribose) glycohydrolase 1-like isoform X3	PARG1	Involved in establishing period length of the circadian oscillator; Involved in defense against fungus; Involved in response to osmotic and water stress	*Promoted circadian clock; Promoted defense against fungi; Promoted drought tolerance; Increased defense against osmotic stress*	Panda, Satchidananda, Guy G. Poirier, and Steve A. Kay. "tej defines a role for poly (ADP-ribosyl) ation in establishing period length of the Arabidopsis circadian oscillator." Developmental cell 3, no. 1 (2002): 51-61.
Solyc11g008530.2	1.33	endoribonuclease Dicer 2d isoform X1	AT3G03300	Plays an essential role in transitive silencing of transgenes by processing secondary siRNAs; Plays a role in antiviral RNA silencing	*Promoted post-transcriptional gene silencing; Promoted defense against viral pathogens*	"DICER-LIKE2 plays a primary role in transitive silencing of transgenes in Arabidopsis."
Solyc12g096600.2	1.34	poly(ADP-ribose) glycohydrolase 1-like isoform X4	PARG1	Involved in establishing period length of the circadian oscillator; Involved in deefense against fungus; Involved in response to osmotic and water stress	*Promoted circadian clock; Promoted defense against fungi; Promoted drought tolerance; Increased defense against osmotic stress*	Panda, Satchidananda, Guy G. Poirier, and Steve A. Kay. "tej defines a role for poly (ADP-ribosyl) ation in establishing period length of the Arabidopsis circadian oscillator." Developmental cell 3, no. 1 (2002): 51-61.
Solyc05g013660.3	1.36	uncharacterized protein LOC101244185 isoform X1	N/A	N/A	N/A	N/A
Solyc09g059040.3	1.37	fructose-bisphosphate aldolase	FBA1	Plays a key role in glycolysis and gluconeogenesis	*Promoted glycolysis and glucogenesis*	Searle, Iain R., Artem E. Men, Titeki S. Laniya, Diana M. Buzas, Inaki Iturbe-Ormaetxe, Bernard J. Carroll, and Peter M. Gresshoff. "Long-distance signaling in nodulation directed by a CLAVATA1-like receptor kinase." Science 299, no. 5603 (2003): 109-112.
Solyc06g073990.2	1.37	uncharacterized protein LOC101253518	N/A	N/A	N/A	N/A

TomatoID	DE	NCBI Protein Name	Gene ID	Uniprot Description	Putative Consequences for Infection	Citation
Solyc03g113430.3	1.37	protein NRT1/ PTR FAMILY 2.11	NPF2.11	High-affinity, proton-dependent glucosinolate-specific transporter; Involved in removal of glucosinolates from the xylem in roots	*Promoted glucosinolate homeostasis*	Nour-Eldin, Hussam Hassan, Tonni Grube Andersen, Meike Burow, Svend Roesen Madsen, Morten Egevang Jørgensen, Carl Erik Olsen, Ingo Dreyer, Rainer Hedrich, Dietmar Geiger, and Barbara Ann Halkier. "NRT/PTR transporters are essential for translocation of glucosinolate defence compounds to seeds." Nature 488, no. 7412 (2012): 531-534.
Solyc07g065320.3	1.41	ABC transporter C family member 3	ABCC3	Pump for glutathione S-conjugates and chlorophyll catabolites; Heavy metal transporter	Increased glutathione S-conjugate and chlorophyll catabolite transport; Increased heavy metal transport	Tommasini, Roberto, Esther Vogt, Myriam Fromenteau, Stefan Hörtensteiner, Philippe Matile, Nikolaus Amrhein, and Enrico Martinoia. "An ABC-transporter of Arabidopsis thaliana has both glutathione-conjugate and chlorophyll catabolite transport activity." The Plant Journal 13, no. 6 (1998): 773-780.
Solyc11g010380.2	1.41	protein DETOXIFICATION 27	DTX27	Has xenobiotic transmembrane transporter activity	*Promoted xenobiotic transport*	Ascencio-Ibánez, José Trinidad, Rosangela Sozzani, Tae-Jin Lee, Tzu-Ming Chu, Russell D. Wolfinger, Rino Cella, and Linda Hanley-Bowdoin. "Global analysis of Arabidopsis gene expression uncovers a complex array of changes impacting pathogen response and cell cycle during geminivirus infection." Plant physiology 148, no. 1 (2008): 436-454.
Solyc02g077590.1	1.42	homeobox-leucine zipper protein ATHB-52	ATHB-52	Transcription factor	N/A	Fleury, Delphine, Kristiina Himanen, Gerda Cnops, Hilde Nelissen, Tommaso Matteo Boccardi, Steven Maere, Gerrit TS Beemster et al. "The Arabidopsis thaliana homolog of yeast BRE1 has a function in cell cycle regulation during early leaf and root growth." The Plant Cell 19, no. 2 (2007): 417-432.
Solyc08g067610.3	1.45	pleiotropic drug resistance protein 1-like	PDR1	Excretes secondary metabolites such as terpenes; Involved in both constitutive and jasmonic acid-dependent induced defense	*Increased secondary metabolite excretion; Promoted jasmonic-acid depended induced defense; Promoted constitutive defense*	Jasiński, Michal, Yvan Stukkens, Hervé Degand, Bénédicte Purnelle, Jacqueline Marchand-Brynaert, and Marc Boutry. "A plant plasma membrane ATP binding cassette–type transporter is involved in antifungal terpenoid secretion." The Plant Cell 13, no. 5 (2001): 1095-1107.
Solyc04g007980.3	1.47	1-aminocyclopropane-1-carboxylate oxidase homolog 4	ACO4	Involved in ethylene biosynthesis; Promotes stem elongation by maximizing the extensibility cells	*Promoted ethylene biosynthesis; Promotes stem elongation and cell extensibility*	Qin, Yong-Mei, Chun-Yang Hu, Yu Pang, Alexander J. Kastaniotis, J. Kalervo Hiltunen, and Yu-Xian Zhu. "Saturated very-long-chain fatty acids promote cotton fiber and Arabidopsis cell elongation by activating ethylene biosynthesis." The Plant Cell 19, no. 11 (2007): 3692-3704.
Solyc04g071890.3	1.47	peroxidase 12 precursor	PER12	Involved in removal of H2O2, oxidation of toxic reductants, biosynthesis and degradation of lignin, suberization, auxin catabolism, and response to environmental stressors	*Increased removal of H2O2, oxidation of toxic reductants, biosynthesis and degradation of lignin, suberization, auxin catabolism, and response to environmental stressors*	Paynel, Florence, et al. "Temporal regulation of cell-wall pectin methylesterase and peroxidase isoforms in cadmium-treated flax hypocotyl." Annals of botany 104.7 (2009): 1363-1372.
Solyc04g064870.3	1.48	protein early flowering 2-like	ELF5	Involved in the regulation of flowering time in both long and short days	*Promoted flowering time*	Noh, Yoo-Sun, Colleen M. Bizzell, Bosl Noh, Fritz M. Schomburg, and Richard M. Amasino. "EARLY FLOWERING 5 acts as a floral repressor in Arabidopsis." The Plant Journal 38, no. 4 (2004): 664-672.

TomatoID	DE	NCBI Protein Name	Gene ID	Uniprot Description	Putative Consequences for Infection	Citation
Solyc08g006470.3	1.49	cytoplasmic 60S subunit biogenesis factor REI1 homolog 1	REIL1	Involved in the dissociation and recycling of other late pre-60S factors before newly synthesized large ribosomal subunits enter translation; Required for leaf growth under cold temperature conditions	*Impaired growth under cold stress*	Schmidt, Stefanie, Frederik Dethloff, Olga Beine-Golovchuk, and Joachim Kopka. "The REIL1 and REIL2 proteins of Arabidopsis thaliana are required for leaf growth in the cold." Plant physiology 163, no. 4 (2013): 1623-1639.
Solyc08g078870.2	1.49	14 kDa proline-rich protein DC2.15-like	N/A	Involved in the initiation of embryogenesis and the metabolic changes related to the removal of auxins	*Promoted embryogenesis; Promoted auxin removal*	Aleith, F., and G. Richter. "Gene expression during induction of somatic embryogenesis in carrot cell suspensions." Planta 183, no. 1 (1991): 17-24.
Solyc09g097960.3	1.49	aldo-keto reductases superfamily protein isoform X1	N/A	N/A	N/A	N/A
Solyc09g082810.3	1.50	N/A	N/A	N/A	N/A	N/A
Solyc01g006560.3	1.52	lipoxygenase, partial	N/A	N/A	N/A	N/A
Solyc08g062220.3	1.53	UDP-glycosyltransferase 74F2	UGT74F2	Glycosyltransferase that glucosylates benzoic acid and derivatives	N/A	Quiel, Juan A., and Judith Bender. "Glucose Conjugation of Anthranilate by theArabidopsis UGT74F2 Glucosyltransferase Is Required for Tryptophan Mutant Blue Fluorescence." Journal of Biological Chemistry 278, no. 8 (2003): 6275-6281.
Solyc03g025450.3	1.55	U-box domain-containing protein 32 isoform X2	PUB32	Functions as an E3 ubiquitin ligase	*Promoted E3 ubiquitination*	Benschop, Joris J., Shabaz Mohammed, Martina O'Flaherty, Albert JR Heck, Monique Slijper, and Frank LH Menke. "Quantitative phosphoproteomics of early elicitor signaling in Arabidopsis." Molecular & Cellular Proteomics 6, no. 7 (2007): 1198-1214.
Solyc10g008620.3	1.56	N/A	N/A	N/A	N/A	N/A
Solyc11g066100.2	1.56	heat shock cognate 70 kDa protein	HSP70-1	Facilitate folding of de novo synthesized proteins, assist translocation of precursor proteins into organelles, and are responsible for degradation of damaged protein under stress conditions	*Promoted folding of de novo synthesized proteins; Promoted protein transport; Promoted degradation of damanged proteins under stress*	Sung, Dong Yul, and Charles L. Guy. "Physiological and molecular assessment of altered expression of Hsc70-1 in Arabidopsis. Evidence for pleiotropic consequences." Plant Physiology 132, no. 2 (2003): 979-987.
Solyc09g089930.2	1.57	ethylene responsive element binding protein	EREBP1	Involved in defense response; Involved in ethylene-activated signaling pathway	*Increased defense response; Increased ethylene-responsive signaling*	Horvath, Diana M., Dorothy J. Huang, and Nam-Hai Chua. "Four classes of salicylate-induced tobacco genes." Molecular plant-microbe interactions 11.9 (1998): 895-905.
Solyc04g080540.2	1.57	uncharacterized protein LOC101245159	N/A	N/A	N/A	N/A

TomatoID	DE	NCBI Protein Name	Gene ID	Uniprot Description	Putative Consequences for Infection	Citation
Solyc12g045020.2	1.57	cytochrome P450 CYP736A12	CYP734A1	Cytochrome P450 involved in brassinosteroids inactivation and regulation of BRs homeostasis	*Promoted brassinosteroid inactivation; Promoted brassinosteroid homeostasis; Impaired growth/development*	Neff, Michael M., Serena M. Nguyen, Elizabeth J. Malancharuvil, Shozo Fujioka, Takahiro Noguchi, Hideharu Seto, Masayoshi Tsubuki et al. "BAS1: A gene regulating brassinosteroid levels and light responsiveness in Arabidopsis." Proceedings of the National Academy of Sciences 96, no. 26 (1999): 15316-15323.
Solyc08g067630.3	1.58	uncharacterized protein LOC101248124	N/A	N/A	N/A	N/A
Solyc01g080800.3	1.62	uncharacterized protein LOC101249488	N/A	N/A	N/A	N/A
Solyc12g005720.1	1.63	cysteine-rich repeat secretory protein 38	CRRSP38	Involved in response to abscisic acid	*Promoted response to abscisic acid signaling*	Huang, Kai-Chau, Wei-Chih Lin, and Wan-Hsing Cheng. "Salt hypersensitive mutant 9, a nucleolar APUM23 protein, is essential for salt sensitivity in association with the ABA signaling pathway in Arabidopsis." BMC plant biology 18, no. 1 (2018): 1-21.
Solyc07g054760.1	1.64	uncharacterized protein LOC101254813	N/A	N/A	N/A	N/A
Solyc10g005080.3	1.65	protein LHY isoform X2	LHY	Transcription factor involved in the circadian clock; Represses CCA1	*Promoted circadian rhythm; Promoted regulation of CCA1*	Mizoguchi, Tsuyoshi, et al. "LHY and CCA1 are partially redundant genes required to maintain circadian rhythms in Arabidopsis." Developmental cell 2.5 (2002): 629-641.
Solyc12g006380.2	1.68	2-oxoglutarate-dependent dioxygenase	AOP3	Involved in glucosinolates biosynthesis	Promoted glucosinolate biosynthesis	Kliebenstein, Daniel J., Virginia M. Lambrix, Michael Reichelt, Jonathan Gershenzon, and Thomas Mitchell-Olds. "Gene duplication in the diversification of secondary metabolism: tandem 2-oxoglutarate–dependent dioxygenases control glucosinolate biosynthesis in Arabidopsis." The Plant Cell 13, no. 3 (2001): 681-693.
Solyc11g072470.2	1.68	LOB domain-containing protein 1	LBD1	N/A	N/A	Hanada, Kousuke, Yuji Sawada, Takashi Kuromori, Romy Klausnitzer, Kazuki Saito, Tetsuro Toyoda, Kazuo Shinozaki, Wen-Hsiung Li, and Masami Yokota Hirai. "Functional compensation of primary and secondary metabolites by duplicate genes in Arabidopsis thaliana." Molecular biology and evolution 28, no. 1 (2011): 377-382.
Solyc04g007000.1	1.69	AP2/ERF and B3 domain-containing transcription factor RAV1	RAV1	Negative regulator of plant growth and development	*Impaired growth and development*	Feng, C.Z., Chen, Y., Wang, C., Kong, Y.H., Wu, W.H. and Chen, Y.F., 2014. Arabidopsis RAV 1 transcription factor, phosphorylated by S n RK 2 kinases, regulates the expression of ABI 3, ABI 4, and ABI 5 during seed germination and early seedling development. The Plant Journal, 80(4), pp.654-668.

TomatoID	DE	NCBI Protein Name	Gene ID	Uniprot Description	Putative Consequences for Infection	Citation
Solyc09g011550.2	1.70	probable glutathione S-transferase	DHAR4	Involved in ascorbate glutathione cycle	Promoted ascorbate glutathione cycle	Cheng, Chia-Yi, Vivek Krishnakumar, Agnes P. Chan, Françoise Thibaud-Nissen, Seth Schobel, and Christopher D. Town. "Araport11: a complete reannotation of the Arabidopsis thaliana reference genome." The Plant Journal 89, no. 4 (2017): 789-804.
Solyc09g097760.3	1.70	glycine-rich protein DC9.1	N/A	N/A	N/A	N/A
Solyc11g007770.2	1.70	glycosyltransferase family protein 64 protein C5	At1g80290	Has glycosyltransferase activity	N/A	Rawat, Arun, Georg J. Seifert, and Youping Deng. "Novel implementation of conditional co-regulation by graph theory to derive co-expressed genes from microarray data." In BMC bioinformatics, vol. 9, no. 9, pp. 1-9. BioMed Central, 2008.
Solyc02g081180.2	1.73	uncharacterized protein LOC104645789	N/A	N/A	N/A	N/A
Solyc02g068470.1	1.75	VQ motif-containing protein 22	VQ22	Functions as a positive regulator of plant growth	*Promoted plant growth*	Cheng, Yuan, Yuan Zhou, Yan Yang, Ying-Jun Chi, Jie Zhou, Jian-Ye Chen, Fei Wang et al. "Structural and functional analysis of VQ motif-containing proteins in Arabidopsis as interacting proteins of WRKY transcription factors." Plant physiology 159, no. 2 (2012): 810-825.
Solyc07g006370.1	1.75	cation/calcium exchanger 1-like	CCX1	Involved in potassium and sodium ion transport	*Promoted potassium and sodium ion transport*	Shigaki, T., I. Rees, L. Nakhleh, and K. D. Hirschi. "Identification of three distinct phylogenetic groups of CAX cation/proton antiporters." Journal of Molecular Evolution 63, no. 6 (2006): 815-825.
Solyc12g099780.2	1.75	N/A	N/A	N/A	N/A	N/A
Solyc04g011480.3	1.77	CASP-like protein PIMP1	At2g38480	Metal-binding	N/A	Vergnolle, Chantal, Marie-Noëlle Vaultier, Ludivine Taconnat, Jean-Pierre Renou, Jean-Claude Kader, Alain Zachowski, and Eric Ruelland. "The cold-induced early activation of phospholipase C and D pathways determines the response of two distinct clusters of genes in Arabidopsis cell suspensions." Plant physiology 139, no. 3 (2005): 1217-1233.
Solyc09g008750.1	1.78	uncharacterized protein LOC104649135	N/A	N/A	N/A	N/A
Solyc07g052790.2	1.79	TMV resistance protein N	N/A	N/A	N/A	N/A
Solyc10g051020.2	1.81	cytochrome P450 CYP72A219	LOC104242999	Heme binding; Iron ion binding; Monooxygenase activity	N/A	Sierro, Nicolas, et al. "Reference genomes and transcriptomes of Nicotiana sylvestris and Nicotiana tomentosiformis." Genome biology 14.6 (2013): R60.
Solyc10g051030.2	1.81	uncharacterized protein LOC101245596 isoform X3	N/A	N/A	N/A	N/A

TomatoID	DE	NCBI Protein Name	Gene ID	Uniprot Description	Putative Consequences for Infection	Citation
Solyc07g065380.4	1.82	zinc transporter-like precursor	ZIP1	Mediates copper, cadmium, and zinc uptake from the rhizosphere	*Promoted copper, cadmium, and zinc uptake from the rhizosphere*	Grotz, Natasha, Tama Fox, Erin Connolly, Walter Park, Mary Lou Guerinot, and David Eide. "Identification of a family of zinc transporter genes from Arabidopsis that respond to zinc deficiency." Proceedings of the National Academy of Sciences 95, no. 12 (1998): 7220-7224.
Solyc03g114890.3	1.84	COBRA-like protein 4 isoform X2	COBL4	Involved in cellulose microfibril organization; Involved in plant cell wall cellulose biosynthesis; Invovled in secondary cell wall biogenesis	*Promoted plant cell wall cellulose biosynthesis; Promoted scondary cell wall biogenesis*	Brown, David M., Leo AH Zeef, Joanne Ellis, Royston Goodacre, and Simon R. Turner. "Identification of novel genes in Arabidopsis involved in secondary cell wall formation using expression profiling and reverse genetics." The Plant Cell 17, no. 8 (2005): 2281-2295.
Solyc04g040130.1	1.86	delta(12)-fatty-acid desaturase FAD2-like	FAD2	Required for desaturation of fatty acids present in extraplastidial membranes, including mitochondria; Required for salt tolerance during seed germination and early seedling growth	*Promoted salt tolerance during seed germination and early growth*	Okuley, John, Jonathan Lightner, Kenneth Feldmann, Narendra Yadav, Ellen Lark, and John Browse. "Arabidopsis FAD2 gene encodes the enzyme that is essential for polyunsaturated lipid synthesis." The Plant Cell 6, no. 1 (1994): 147-158.
Solyc07g054780.1	1.87	uncharacterized protein LOC544070	N/A	N/A	N/A	N/A
Solyc05g010040.2	1.89	N/A	N/A	N/A	N/A	N/A
Solyc10g076550.1	1.90	wall-associated receptor kinase 2-like	WAK2	Serine/threonine-protein kinase that functions as a signaling receptor of extracellular matrix component; Involved in the control of cell expansion, morphogenesis, and development	*Promoted signaling turnover; Promoted cell expansion, morphogenesis, and development*	Wagner, Tanya A., and Bruce D. Kohorn. "Wall-associated kinases are expressed throughout plant development and are required for cell expansion." The Plant Cell 13, no. 2 (2001): 303-318.
Solyc11g005840.2	1.91	uncharacterized protein LOC101247180	N/A	N/A	N/A	N/A
Solyc06g084370.1	1.93	uncharacterized protein LOC101254903	N/A	N/A	N/A	N/A
Solyc10g076537.1	2.11	N/A	N/A	N/A	N/A	N/A
Solyc04g014400.3	2.11	receptor-like protein 13 isoform X1	RLP13	Receptor protein	N/A	Theologis, Athanasios, Joseph R. Ecker, Curtis J. Palm, Nancy A. Federspiel, Samir Kaul, Owen White, Jose Alonso et al. "Sequence and analysis of chromosome 1 of the plant Arabidopsis thaliana." Nature 408, no. 6814 (2000): 816-820.
Solyc05g053610.2	2.22	pleiotropic drug resistance protein 1-like	PDR1	Excretes secondary metabolites such as terpenes; Involved in both constitutive and jasmonic acid-dependent induced defense	*Increased secondary metabolite excretion; Promoted jasmonic-acid depended induced defense; Promoted constitutive defense*	Jasiński, Michal, Yvan Stukkens, Hervé Degand, Bénédicte Purnelle, Jacqueline Marchand-Brynaert, and Marc Boutry. "A plant plasma membrane ATP binding cassette–type transporter is involved in antifungal terpenoid secretion." The Plant Cell 13, no. 5 (2001): 1095-1107.

TomatoID	DE	NCBI Protein Name	Gene ID	Uniprot Description	Putative Consequences for Infection	Citation
Solyc09g007520.3	2.27	peroxidase 21	PER21	Involved in removal of H2O2, oxidation of toxic reductants, biosynthesis and degradation of lignin, suberization, auxin catabolism, and response to environmental stressors	*Increased removal of H2O2, oxidation of toxic reductants, biosynthesis and degradation of lignin, suberization, auxin catabolism, and response to environmental stressors*	Mosher, Rebecca A., Wendy E. Durrant, Dong Wang, Junqi Song, and Xinnian Dong. "A comprehensive structure–function analysis of Arabidopsis SNI1 defines essential regions and transcriptional repressor activity." The Plant Cell 18, no. 7 (2006): 1750-1765.
Solyc12g100270.2	2.31	protein ECERIFERUM 1	N/A	Involved in the lipid biosynthetic process	Promoted lipid biosynthesis	Garcia-Mas, Jordi, Andrej Benjak, Walter Sanseverino, Michael Bourgeois, Gisela Mir, Víctor M. González, Elizabeth Hénaff et al. "The genome of melon (Cucumis melo L.)." Proceedings of the National Academy of Sciences 109, no. 29 (2012): 11872-11877.
Solyc06g005813.1	2.34	N/A	N/A	N/A	N/A	N/A
Solyc09g008760.1	2.39	probable cytochrome P450 556A1	cyp516A1	Involved in xenobiotic metabolic process	*Promoted xenobiotic metabolism*	Eichinger, L., J. A. Pachebat, G. Glöckner, M-A. Rajandream, R. Sucgang, M. Berriman, J. Song et al. "The genome of the social amoeba Dictyostelium discoideum." Nature 435, no. 7038 (2005): 43-57.
Solyc08g080585.1	2.41	N/A	N/A	N/A	N/A	N/A
Solyc03g095650.3	2.50	MLO-like protein 5	MLO5	Involved in modulation of pathogen defense and leaf cell death	*Promoted defense against pathogens; Promoted leaf programmed cell death*	Moriyama, Etsuko N., Pooja K. Strope, Stephen O. Opiyo, Zhongying Chen, and Alan M. Jones. "Mining the Arabidopsis thaliana genome for highly-divergent seven transmembrane receptors." Genome biology 7, no. 10 (2006): 1-9.
Solyc12g096570.1	2.52	protein AUXIN-REGULATED GENE INVOLVED IN ORGAN SIZE	ARGOS	Promotes cell proliferation-dependent organ growth; Takes part in the AXR1-dependent auxin signaling pathway that requires ANT during organogenesis	*Promoted organ growth; Promoted auxin signaling*	Hu, Yuxin, Qi Xie, and Nam-Hai Chua. "The Arabidopsis auxin-inducible gene ARGOS controls lateral organ size." The Plant Cell 15, no. 9 (2003): 1951-1961.
Solyc03g033840.3	2.57	AAA-ATPase At3g50940-like	At3g50940	Involved in lignin biosynthesis and response to salt stress	*Promoted lignin biosynthesis; Promoted resposne to salt stress*	van de Mortel, Judith E., Laia Almar Villanueva, Henk Schat, Jeroen Kwekkeboom, Sean Coughlan, Perry D. Moerland, Emiel Ver Loren van Themaat, Maarten Koornneef, and Mark GM Aarts. "Large expression differences in genes for iron and zinc homeostasis, stress response, and lignin biosynthesis distinguish roots of Arabidopsis thaliana and the related metal hyperaccumulator Thlaspi caerulescens." Plant physiology 142, no. 3 (2006): 1127-1147.
Solyc08g068870.3	2.63	aspartate protease family protein precursor	APF1	Aspartyl protease; Necessary for fungal resistance	*Increased fungal resistance*	Li, Yurong, Mehdi Kabbage, Wende Liu, and Martin B. Dickman. "Aspartyl protease-mediated cleavage of BAG6 is necessary for autophagy and fungal resistance in plants." The Plant Cell 28, no. 1 (2016): 233-247.

TomatoID	DE	NCBI Protein Name	Gene ID	Uniprot Description	Putative Consequences for Infection	Citation
Solyc12g100250.2	2.78	delta(12)-fatty-acid desaturase FAD2 isoform X1	FAD2	Required for desaturation of fatty acids present in extraplastidial membranes, including mitochondria; Required for salt tolerance during seed germination and early seedling growth	*Promoted salt tolerance during seed germination and early growth*	Okuley, John, Jonathan Lightner, Kenneth Feldmann, Narendra Yadav, Ellen Lark, and John Browse. "Arabidopsis FAD2 gene encodes the enzyme that is essential for polyunsaturated lipid synthesis." The Plant Cell 6, no. 1 (1994): 147-158.
Solyc12g100240.1	2.78	delta(12)-fatty-acid desaturase FAD2-like	FAD2	Required for desaturation of fatty acids present in extraplastidial membranes, including mitochondria; Required for salt tolerance during seed germination and early seedling growth	*Promoted salt tolerance during seed germination and early growth*	Okuley, John, Jonathan Lightner, Kenneth Feldmann, Narendra Yadav, Ellen Lark, and John Browse. "Arabidopsis FAD2 gene encodes the enzyme that is essential for polyunsaturated lipid synthesis." The Plant Cell 6, no. 1 (1994): 147-158.
Solyc04g014770.1	3.12	N/A	N/A	N/A	N/A	N/A
Solyc07g056510.3	3.12	probable glutathione S-transferase parA	DHAR2	As a soluble protein, exhibits glutathione-dependent thiol transferase and dehydroascorbate reductase activities; Key component of the ascorbate recycling system	Promoted ascorbate recycling	Dixon, David P., Benjamin G. Davis, and Robert Edwards. "Functional divergence in the glutathione transferase superfamily in plants: identification of two classes with putative functions in redox homeostasis in Arabidopsis thaliana." Journal of Biological Chemistry 277, no. 34 (2002): 30859-30869.
Solyc12g100260.1	3.19	delta(12)-fatty-acid desaturase FAD2-like	FAD2	Required for desaturation of fatty acids present in extraplastidial membranes, including mitochondria; Required for salt tolerance during seed germination and early seedling growth	*Promoted salt tolerance during seed germination and early growth*	Okuley, John, Jonathan Lightner, Kenneth Feldmann, Narendra Yadav, Ellen Lark, and John Browse. "Arabidopsis FAD2 gene encodes the enzyme that is essential for polyunsaturated lipid synthesis." The Plant Cell 6, no. 1 (1994): 147-158.
Solyc05g015850.3	3.81	WRKY DNA binding protein 1	WRKY1	Transcription factor	N/A	de Pater, Sylvia, Valentina Greco, Khanh Pham, Johan Memelink, and Jan Kijne. "Characterization of a zinc-dependent transcriptional activator from Arabidopsis." Nucleic Acids Research 24, no. 23 (1996): 4624-4631.
Solyc09g091000.3	4.16	pathogenesis-related protein STH-2	STH-2	Regulates protein serein/threonine phosphate activity; Involved in abscisic acid-activated signaling pathway; Involved in defense response to biotic stimulus	*Promoted absisic acid signaling; Promoted defense against biotic stimuli*	Matton, Daniel P., Gary Prescott, Charles Bertrand, Anne Camirand, and Normand Brisson. "Identification of cis-acting elements involved in the regulation of the pathogenesis-related gene STH-2 in potato." Plant molecular biology 22, no. 2 (1993): 279-291.
