# Supplementary material for: Effects of ‘Candidatus Liberibacter solanacearum’ haplotypes A and B on tomato gene expression and geotropism
Source: BMC Plant Biol. 2022 Mar 30;22:156. doi: 10.1186/s12870-022-03505-z (PMC8966271; doi:10.1186/s12870-022-03505-z)
Supplement: Supplementary file 11 — Additional file 11: Table S11. KEGG Pathway search analysis of tomato plant DEGs associated with infection by different Lso haplotypes. Among the 166-haplotype associated DEGs also involved in a KEGG Pathway, 39 were involved in metabolic pathways, 22 were involved in the biosynthesis of secondary metabolites, 5 were involved in plant hormone signal transduction, 5 were involved in photosynthesis, 5 were involved in circadian rhythm, and 5 were involved in phenylpropanoid biosynthesis. Several more DEGs were involved in other pathways (e.g., photosynthesis), but with less representation. [file 12870_2022_3505_MOESM11_ESM.pdf]

| KEGG Pathway | Pathway Name | KEGG Gene ID | Gene Name |
|---|---|---|---|
| ath01100 | Metabolic pathways | ath:AT1G05010 | EFE; ethylene-forming enzyme |
| ath01100 | Metabolic pathways | ath:AT1G08200 | AXS2; UDP-D-apiose/UDP-D-xylose synthase 2 |
| ath01100 | Metabolic pathways | ath:AT1G23820 | SPDS1; spermidine synthase |
| ath01100 | Metabolic pathways | ath:AT1G26570 | UGD1; UDP-glucose dehydrogenase 1 |
| ath01100 | Metabolic pathways | ath:AT1G30620 | MUR4; NAD(P)-binding Rossmann-fold superfamily protein |
| ath01100 | Metabolic pathways | ath:AT1G60810 | ACLA-2; ATP-citrate lyase A-2 |
| ath01100 | Metabolic pathways | ath:AT1G67750 | Pectate lyase family protein |
| ath01100 | Metabolic pathways | ath:AT1G68530 | KCS6; 3-ketoacyl-CoA synthase 6 |
| ath01100 | Metabolic pathways | ath:AT1G71695 | Peroxidase superfamily protein |
| ath01100 | Metabolic pathways | ath:AT1G75270 | DHAR2; dehydroascorbate reductase 2 |
| ath01100 | Metabolic pathways | ath:AT1G76490 | HMG1; hydroxy methylglutaryl CoA reductase 1 |
| ath01100 | Metabolic pathways | ath:AT2G21330 | FBA1; fructose-bisphosphate aldolase 1 |
| ath01100 | Metabolic pathways | ath:AT2G24200 | LAP1; Cytosol aminopeptidase family protein |
| ath01100 | Metabolic pathways | ath:AT2G37130 | Peroxidase superfamily protein |
| ath01100 | Metabolic pathways | ath:AT2G43590 | Chitinase family protein |
| ath01100 | Metabolic pathways | ath:AT3G06350 | MEE32; dehydroquinate dehydratase, putative / shikimate dehydrogenase |
| ath01100 | Metabolic pathways | ath:AT3G12120 | FAD2; fatty acid desaturase 2 |
| ath01100 | Metabolic pathways | ath:AT3G13560 | O-Glycosyl hydrolases family 17 protein |
| ath01100 | Metabolic pathways | ath:AT3G25400 | dCTP pyrophosphatase-like protein |
| ath01100 | Metabolic pathways | ath:AT3G45770 | Polyketide synthase, enoylreductase family protein |
| ath01100 | Metabolic pathways | ath:AT4G08870 | ARGAH2; Arginase/deacetylase superfamily protein |
| ath01100 | Metabolic pathways | ath:AT4G26220 | S-adenosyl-L-methionine-dependent methyltransferases superfamily protein |
| ath01100 | Metabolic pathways | ath:AT4G30920 | LAP2; Cytosol aminopeptidase family protein |
| ath01100 | Metabolic pathways | ath:AT4G33150 | lysine-ketoglutarate reductase/saccharopine dehydrogenase bifunctional |
| ath01100 | Metabolic pathways | ath:AT4G35160 | O-methyltransferase family protein |
| ath01100 | Metabolic pathways | ath:AT4G37970 | CAD6; cinnamyl alcohol dehydrogenase 6 |
| ath01100 | Metabolic pathways | ath:AT4G39010 | GH9B18; glycosyl hydrolase 9B18 |
| ath01100 | Metabolic pathways | ath:AT5G03300 | ADK2; adenosine kinase 2 |
| ath01100 | Metabolic pathways | ath:AT5G06060 | NAD(P)-binding Rossmann-fold superfamily protein |
| ath01100 | Metabolic pathways | ath:AT5G20830 | SUS1; sucrose synthase 1 |
| ath01100 | Metabolic pathways | ath:AT5G21100 | Plant L-ascorbate oxidase |
| ath01100 | Metabolic pathways | ath:AT5G37600 | GSR_1; hypothetical protein |
| ath01100 | Metabolic pathways | ath:AT5G40150 | Peroxidase superfamily protein |
| ath01100 | Metabolic pathways | ath:AT5G60340 | AAK6; P-loop containing nucleoside triphosphate hydrolases superfamily protein |
| ath01100 | Metabolic pathways | ath:ArthCp002 | psbA; photosystem II protein D1 |
| ath01100 | Metabolic pathways | ath:ArthCp007 | atpA; ATP synthase CF1 alpha subunit |
| ath01100 | Metabolic pathways | ath:ArthCp018 | psbC; photosystem II 44 kDa protein |
| ath01100 | Metabolic pathways | ath:ArthCp022 | psaA; photosystem I P700 chlorophyll a apoprotein A1 |
| ath01100 | Metabolic pathways | ath:ArthCp053 | petB; cytochrome b6 |
| ath01110 | Biosynthesis of secondary metabolites | ath:AT1G05010 | EFE; ethylene-forming enzyme |
| ath01110 | Biosynthesis of secondary metabolites | ath:AT1G19640 | JMT; jasmonic acid carboxyl methyltransferase |
| ath01110 | Biosynthesis of secondary metabolites | ath:AT1G60810 | ACLA-2; ATP-citrate lyase A-2 |
| ath01110 | Biosynthesis of secondary metabolites | ath:AT1G68530 | KCS6; 3-ketoacyl-CoA synthase 6 |
| ath01110 | Biosynthesis of secondary metabolites | ath:AT1G71695 | Peroxidase superfamily protein |
| ath01110 | Biosynthesis of secondary metabolites | ath:AT1G76490 | HMG1; hydroxy methylglutaryl CoA reductase 1 |
| ath01110 | Biosynthesis of secondary metabolites | ath:AT2G19500 | CKX2; cytokinin oxidase 2 |
| ath01110 | Biosynthesis of secondary metabolites | ath:AT2G21330 | FBA1; fructose-bisphosphate aldolase 1 |
| ath01110 | Biosynthesis of secondary metabolites | ath:AT2G24190 | SDR2; NAD(P)-binding Rossmann-fold superfamily protein |
| ath01110 | Biosynthesis of secondary metabolites | ath:AT2G26710 | BAS1; Cytochrome P450 superfamily protein |
| ath01110 | Biosynthesis of secondary metabolites | ath:AT2G36750 | UGT73C1; UDP-glucosyl transferase 73C1 |
| ath01110 | Biosynthesis of secondary metabolites | ath:AT2G37130 | Peroxidase superfamily protein |
| ath01110 | Biosynthesis of secondary metabolites | ath:AT3G06350 | MEE32; dehydroquinate dehydratase, putative / shikimate dehydrogenase |
| ath01110 | Biosynthesis of secondary metabolites | ath:AT4G08870 | ARGAH2; Arginase/deacetylase superfamily protein |
| ath01110 | Biosynthesis of secondary metabolites | ath:AT4G26220 | S-adenosyl-L-methionine-dependent methyltransferases superfamily protein |
| ath01110 | Biosynthesis of secondary metabolites | ath:AT4G33150 | lysine-ketoglutarate reductase/saccharopine dehydrogenase bifunctional enzyme |
| ath01110 | Biosynthesis of secondary metabolites | ath:AT4G35160 | O-methyltransferase family protein |

| KEGG Pathway | Pathway Name | KEGG Gene ID | Gene Name |
|---|---|---|---|
| ath01110 | Biosynthesis of secondary metabolites | ath:AT4G37970 | CAD6; cinnamyl alcohol dehydrogenase 6 |
| ath01110 | Biosynthesis of secondary metabolites | ath:AT5G06060 | NAD(P)-binding Rossmann-fold superfamily protein |
| ath01110 | Biosynthesis of secondary metabolites | ath:AT5G20830 | SUS1; sucrose synthase 1 |
| ath01110 | Biosynthesis of secondary metabolites | ath:AT5G40150 | Peroxidase superfamily protein |
| ath01110 | Biosynthesis of secondary metabolites | ath:AT5G60340 | AAK6; P-loop containing nucleoside triphosphate hydrolases superfamily protein |
| ath04075 | Plant hormone signal transduction | ath:AT1G74890 | ARR15; response regulator 15 |
| ath04075 | Plant hormone signal transduction | ath:AT2G25490 | EBF1; EIN3-binding F box protein 1 |
| ath04075 | Plant hormone signal transduction | ath:AT3G11410 | PP2CA; protein phosphatase 2CA |
| ath04075 | Plant hormone signal transduction | ath:AT3G63010 | GID1B; alpha/beta-Hydrolases superfamily protein |
| ath04075 | Plant hormone signal transduction | ath:AT5G18030 | SAUR-like auxin-responsive protein family |
| ath00195 | Photosynthesis | ath:ArthCp002 | psbA; photosystem II protein D1 |
| ath00195 | Photosynthesis | ath:ArthCp007 | atpA; ATP synthase CF1 alpha subunit |
| ath00195 | Photosynthesis | ath:ArthCp018 | psbC; photosystem II 44 kDa protein |
| ath00195 | Photosynthesis | ath:ArthCp022 | psaA; photosystem I P700 chlorophyll a apoprotein A1 |
| ath00195 | Photosynthesis | ath:ArthCp053 | petB; cytochrome b6 |
| ath04712 | Circadian rhythm | ath:AT1G01060 | LHY; Homeodomain-like superfamily protein |
| ath04712 | Circadian rhythm | ath:AT1G22770 | GI; gigantea protein (GI) |
| ath04712 | Circadian rhythm | ath:AT1G68050 | FKF1; flavin-binding, kelch repeat, f box 1 |
| ath04712 | Circadian rhythm | ath:AT5G24470 | PRR5; two-component response regulator-like protein |
| ath04712 | Circadian rhythm | ath:AT5G62430 | CDF1; cycling DOF factor 1 |
| ath00940 | Phenylpropanoid biosynthesis | ath:AT1G71695 | Peroxidase superfamily protein |
| ath00940 | Phenylpropanoid biosynthesis | ath:AT2G37130 | Peroxidase superfamily protein |
| ath00940 | Phenylpropanoid biosynthesis | ath:AT4G26220 | S-adenosyl-L-methionine-dependent methyltransferases superfamily protein |
| ath00940 | Phenylpropanoid biosynthesis | ath:AT4G37970 | CAD6; cinnamyl alcohol dehydrogenase 6 |
| ath00940 | Phenylpropanoid biosynthesis | ath:AT5G40150 | Peroxidase superfamily protein |
| ath00520 | Amino sugar and nucleotide sugar metabolism | ath:AT1G08200 | AXS2; UDP-D-apiose/UDP-D-xylose synthase 2 |
| ath00520 | Amino sugar and nucleotide sugar metabolism | ath:AT1G26570 | UGD1; UDP-glucose dehydrogenase 1 |
| ath00520 | Amino sugar and nucleotide sugar metabolism | ath:AT1G30620 | MUR4; NAD(P)-binding Rossmann-fold superfamily protein |
| ath00520 | Amino sugar and nucleotide sugar metabolism | ath:AT2G43590 | Chitinase family protein |
| ath00480 | Glutathione metabolism | ath:AT1G23820 | SPDS1; spermidine synthase |
| ath00480 | Glutathione metabolism | ath:AT1G75270 | DHAR2; dehydroascorbate reductase 2 |
| ath00480 | Glutathione metabolism | ath:AT2G24200 | LAP1; Cytosol aminopeptidase family protein |
| ath00480 | Glutathione metabolism | ath:AT4G30920 | LAP2; Cytosol aminopeptidase family protein |
| ath01230 | Biosynthesis of amino acids | ath:AT2G21330 | FBA1; fructose-bisphosphate aldolase 1 |
| ath01230 | Biosynthesis of amino acids | ath:AT3G06350 | MEE32; dehydroquinate dehydratase, putative / shikimate dehydrogenase |
| ath01230 | Biosynthesis of amino acids | ath:AT4G08870 | ARGAH2; Arginase/deacetylase superfamily protein |
| ath01230 | Biosynthesis of amino acids | ath:AT5G37600 | GSR_1; hypothetical protein |
| ath04141 | Protein processing in endoplasmic reticulum | ath:AT1G07400 | HSP20-like chaperones superfamily protein |
| ath04141 | Protein processing in endoplasmic reticulum | ath:AT1G75950 | SKP1; S phase kinase-associated protein 1 |
| ath04141 | Protein processing in endoplasmic reticulum | ath:AT5G02490 | Hsp70-2; Heat shock protein 70 (Hsp 70) family protein |
| ath04141 | Protein processing in endoplasmic reticulum | ath:AT5G02500 | HSC70-1; heat shock cognate protein 70-1 |
| ath04145 | Phagosome | ath:AT1G20010 | TUB5; tubulin beta-5 chain |
| ath04145 | Phagosome | ath:AT1G64740 | TUA1; alpha-1 tubulin |
| ath04145 | Phagosome | ath:AT1G75780 | TUB1; tubulin beta-1 chain |
| ath04144 | Endocytosis | ath:AT1G01200 | RABA3; RAB GTPase homolog A3 |
| ath04144 | Endocytosis | ath:AT5G02490 | Hsp70-2; Heat shock protein 70 (Hsp 70) family protein |
| ath04144 | Endocytosis | ath:AT5G02500 | HSC70-1; heat shock cognate protein 70-1 |
| ath00500 | Starch and sucrose metabolism | ath:AT3G13560 | O-Glycosyl hydrolases family 17 protein |
| ath00500 | Starch and sucrose metabolism | ath:AT4G39010 | GH9B18; glycosyl hydrolase 9B18 |
| ath00500 | Starch and sucrose metabolism | ath:AT5G20830 | SUS1; sucrose synthase 1 |
| ath00053 | Ascorbate and aldarate metabolism | ath:AT1G26570 | UGD1; UDP-glucose dehydrogenase 1 |

| KEGG Pathway | Pathway Name | KEGG Gene ID | Gene Name |
|---|---|---|---|
| ath00053 | Ascorbate and aldarate metabolism | ath:AT1G75270 | DHAR2; dehydroascorbate reductase 2 |
| ath00053 | Ascorbate and aldarate metabolism | ath:AT5G21100 | Plant L-ascorbate oxidase |
| ath04016 | MAPK signaling pathway | ath:AT2G25490 | EBF1; EIN3-binding F box protein 1 |
| ath04016 | MAPK signaling pathway | ath:AT2G32510 | MAPKKK17; mitogen-activated protein kinase kinase kinase 17 |
| ath04016 | MAPK signaling pathway | ath:AT3G11410 | PP2CA; protein phosphatase 2CA |
| ath01250 | Biosynthesis of nucleotide sugars | ath:AT1G08200 | AXS2; UDP-D-apiose/UDP-D-xylose synthase 2 |
| ath01250 | Biosynthesis of nucleotide sugars | ath:AT1G26570 | UGD1; UDP-glucose dehydrogenase 1 |
| ath01250 | Biosynthesis of nucleotide sugars | ath:AT1G30620 | MUR4; NAD(P)-binding Rossmann-fold superfamily protein |
| ath04626 | Plant-pathogen interaction | ath:AT1G68530 | KCS6; 3-ketoacyl-CoA synthase 6 |
| ath04626 | Plant-pathogen interaction | ath:AT2G04880 | ZAP1; zinc-dependent activator protein-1 |
| ath00330 | Arginine and proline metabolism | ath:AT1G23820 | SPDS1; spermidine synthase |
| ath00330 | Arginine and proline metabolism | ath:AT4G08870 | ARGAH2; Arginase/deacetylase superfamily protein |
| ath00062 | Fatty acid elongation | ath:AT1G68530 | KCS6; 3-ketoacyl-CoA synthase 6 |
| ath00062 | Fatty acid elongation | ath:AT3G45770 | Polyketide synthase, enoylreductase family protein |
| ath01240 | Biosynthesis of cofactors | ath:AT1G26570 | UGD1; UDP-glucose dehydrogenase 1 |
| ath01240 | Biosynthesis of cofactors | ath:AT5G60340 | AAK6; P-loop containing nucleoside triphosphate hydrolases superfamily protein |
| ath01212 | Fatty acid metabolism | ath:AT3G12120 | FAD2; fatty acid desaturase 2 |
| ath01212 | Fatty acid metabolism | ath:AT3G45770 | Polyketide synthase, enoylreductase family protein |
| ath00908 | Zeatin biosynthesis | ath:AT2G19500 | CKX2; cytokinin oxidase 2 |
| ath00908 | Zeatin biosynthesis | ath:AT2G36750 | UGT73C1; UDP-glucosyl transferase 73C1 |
| ath00270 | Cysteine and methionine metabolism | ath:AT1G05010 | EFE; ethylene-forming enzyme |
| ath00270 | Cysteine and methionine metabolism | ath:AT1G23820 | SPDS1; spermidine synthase |
| ath03040 | Spliceosome | ath:AT5G02490o | Hsp70-2; Heat shock protein 70 (Hsp 70) family pr |
| ath03040 | Spliceosome | ath:AT5G02500 | HSC70-1; heat shock cognate protein 70-1 |
| ath00220 | Arginine biosynthesis | ath:AT4G08870 | ARGAH2; Arginase/deacetylase superfamily protein |
| ath00220 | Arginine biosynthesis | ath:AT5G37600 | GSR_1; hypothetical protein |
| ath00040 | Pentose and glucuronate interconversions | ath:AT1G26570 | UGD1; UDP-glucose dehydrogenase 1 |
| ath00040 | Pentose and glucuronate interconversions | ath:AT1G67750 | Pectate lyase family protein |
| ath00230 | Purine metabolism | ath:AT5G03300 | ADK2; adenosine kinase 2 |
| ath00230 | Purine metabolism | ath:AT5G60340 | AAK6; P-loop containing nucleoside triphosphate hydrolases super family protein |
| ath00945 | Stilbenoid, diarylheptanoid and gingerol biosynthesis | ath:AT4G26220 | S-adenosyl-L-methionine-dependent methyltransferases superfamily protein |
| ath00592 | alpha-Linolenic acid metabolism | ath:AT1G19640 | JMT; jasmonic acid carboxyl methyltransferase |
| ath00941 | Flavonoid biosynthesis | ath:AT4G26220 | S-adenosyl-L-methionine-dependent methyltransferases superfamily protein |
| ath01200 | Carbon metabolism | ath:AT2G21330 | FBA1; fructose-bisphosphate aldolase 1 |
| ath00902 | Monoterpenoid biosynthesis | ath:AT2G24190 | SDR2; NAD(P)-binding Rossmann-fold superfamily protein |
| ath00960 | Tropane, piperidine and pyridine alkaloid biosynthesis | ath:AT5G06060 | NAD(P)-binding Rossmann-fold superfamily protein |
| ath00905 | Brassinosteroid biosynthesis | ath:AT2G26710 | BAS1; Cytochrome P450 superfamily protein |
| ath04146 | Peroxisome | ath:AT2G28190 | CSD2; copper/zinc superoxide dismutase 2 |
| ath03060 | Protein export | ath:AT1G48160 | signal recognition particle 19 kDa protein, putative / SRP19 |
| ath00310 | Lysine degradation | ath:AT4G33150 | lysine-ketoglutarate reductase/saccharopine dehydrogenase bifunctional enzyme |
| ath00190 | Oxidative phosphorylation | ath:ArthCp007 | atpA; ATP synthase CF1 alpha subunit |
| ath00051 | Fructose and mannose metabolism | ath:AT2G21330 | FBA1; fructose-bisphosphate aldolase 1 |
| ath00400 | Phenylalanine, tyrosine and tryptophan biosynthesis | ath:AT3G06350 | MEE32; dehydroquinate dehydratase, putative / shikimate dehydrogenase |
| ath00020 | Citrate cycle (TCA cycle) | ath:AT1G60810 | ACLA-2; ATP-citrate lyase A-2 |
| ath00010 | Glycolysis / Gluconeogenesis | ath:AT2G21330 | FBA1; fructose-bisphosphate aldolase 1 |
| ath00380 | Tryptophan metabolism | ath:AT4G35160 | O-methyltransferase family protein |
| ath00030 | Pentose phosphate pathway | ath:AT2G21330 | FBA1; fructose-bisphosphate aldolase 1 |
| ath01040 | Biosynthesis of unsaturated fatty acids | ath:AT3G12120 | FAD2; fatty acid desaturase 2 |
| ath00630 | Glyoxylate and dicarboxylate metabolism | ath:AT5G37600 | GSR_1; hypothetical protein |
| ath03015 | mRNA surveillance pathway | ath:AT3G26618 | ERF1-3; eukaryotic release factor 1-3 |
| ath00900 | Terpenoid backbone biosynthesis | ath:AT1G76490 | HMG1; hydroxy methylglutaryl CoA reductase 1 |

| KEGG Pathway | Pathway Name | KEGG Gene ID | Gene Name |
|---|---|---|---|
| ath00250 | Alanine, aspartate and glutamate metabolism | ath:AT5G37600 | GSR_1; hypothetical protein |
| ath03008 | Ribosome biogenesis in eukaryotes | ath:AT5G60340 | AAK6; P-loop containing nucleoside triphosphate hydrolases superfamily protein |
| ath00910 | Nitrogen metabolism | ath:AT5G37600 | GSR_1; hypothetical protein |
| ath00240 | Pyrimidine metabolism | ath:AT3G25400 | dCTP pyrophosphatase-like protein |
| ath00999 | Biosynthesis of various plant secondary metabolites | ath:AT3G06350 | MEE32; dehydroquinate dehydratase, putative / shikimate dehydrogenase |
| ath04120 | Ubiquitin mediated proteolysis | ath:AT1G75950 | SKP1; S phase kinase-associated protein 1 |
| ath00061 | Fatty acid biosynthesis | ath:AT3G45770 | Polyketide synthase, enoylreductase family protein |
| ath00710 | Carbon fixation in photosynthetic organisms | ath:AT2G21330 | FBA1; fructose-bisphosphate aldolase 1 |
