# Supplementary material for: Effects of ‘Candidatus Liberibacter solanacearum’ haplotypes A and B on tomato gene expression and geotropism
Source: BMC Plant Biol. 2022 Mar 30;22:156. doi: 10.1186/s12870-022-03505-z (PMC8966271; doi:10.1186/s12870-022-03505-z)
Supplement: Supplementary file 12 — Additional file 12: Table S12. The 89 tomato plant DEGs associated with plant growth and development under infection by different Lso haplotypes. DEGs were sorted by differential expression (DE) values comparing uninfected to infected samples (p-value < 0.05). NCBI Blast searches were used to identify Gene IDs and protein products in tomatoes as well as their homologs in other species. Specifically, the expression changes in 68 of these genes (76%, in italics) would have resulted in impaired cell growth and development in LsoB-infected plants relative to LsoA-infected plants. These DEGs were predominantly related to cell expansion and elongation and increased cell wall modification. [file 12870_2022_3505_MOESM12_ESM.pdf]

TomatoID	DE in LsoB	NCBI Protein Name	Gene ID	Uniprot Description	Putative Consequences for Infection	Citation
Solyc06g009190.3	2.60	pectinesterase	PME1	Acts in the modification of cell walls via demethylesterification of cell wall pectin; Acts as negative regulators of genes involved in salt stress response	*Impaired cell wall modification; Impaired response to salt stress*	Creighton, Maria T., Anna Kolton, Amr RA Kataya, Jodi Maple-Grødem, Irina O. Averkina, Behzad Heidari, and Cathrine Lillo. "Methylation of protein phosphatase 2A—Influence of regulators and environmental stress factors." Plant, cell & environment 40, no. 10 (2017): 2347-2358.
Solyc10g079350.2	2.52	zeatin O-glucosyltransferase-like	ZOG1	Regulates cytokinin activity and storage; Impacts seed growth	*Impaired cellular division and plant growth in roots and shoots; Impaired reproductive investment*	Hou, Bingkai, et al. "N-glucosylation of cytokinins by glycosyltransferases of Arabidopsis thaliana." Journal of Biological Chemistry 279.46 (2004): 47822-47832.
Solyc03g098300.1	2.22	ornithine decarboxylase-like	ODC	Catalyzes the first and rate-limiting step of polyamine biosynthesis; Polyamines are essential for cell proliferation	*Impaired polyamine biosynthesis; Impaired cell proliferation*	Alabadı, David, and Juan Carbonell. "Expression of ornithine decarboxylase is transiently increased by pollination, 2, 4-dichlorophenoxyacetic acid, and gibberellic acid in tomato ovaries." Plant physiology 118, no. 1 (1998): 323-328.
Solyc05g007830.3	2.19	expansin-A1	EXPA1	Causes loosening and extension of plant cell walls by disrupting non-covalent bonding between cellulose microfibrils and matrix glucans	*Impaired plant cell wall extension*	Zhang, Xiu-Qing, Peng-Cheng Wei, Yan-Mei Xiong, Yi Yang, Jia Chen, and Xue-Chen Wang. "Overexpression of the Arabidopsis α-expansin gene AtEXPA1 accelerates stomatal opening by decreasing the volumetric elastic modulus." Plant cell reports 30, no. 1 (2011): 27-36.
Solyc08g067530.1	2.16	non-specific lipid-transfer protein 1-like	LTP1	Plays a role in wax or cutin deposition in the cell walls of expanding epidermal cells and certain secretory tissues	*Impaired wax/cutin deposition; Impaired plant cell wall expansion*	Skriver, Karen, Robert Leah, Frieder Müller-Uri, Finn-Lok Olsen, and John Mundy. "Structure and expression of the barley lipid transfer protein gene Ltp1." Plant molecular biology 18, no. 3 (1992): 585-589.
Solyc12g015690.2	2.14	fasciclin-like arabinogalactan protein 1	FLA1	Cell surface adhesion protein; Involved in root and shoot development	*Impaired root/shoot development*	Sultana, Nighat, Hannah V. Florance, Alex Johns, and Nicholas Smirnoff. "Ascorbate deficiency influences the leaf cell wall glycoproteome in A rabidopsis thaliana." Plant, cell & environment 38, no. 2 (2015): 375-384.
Solyc01g005290.3	2.13	fasciclin-like arabinogalactan protein 1	FLA1	Cell surface adhesion protein; Involved in root and shoot development	*Impaired root/shoot development*	Sultana, Nighat, Hannah V. Florance, Alex Johns, and Nicholas Smirnoff. "Ascorbate deficiency influences the leaf cell wall glycoproteome in A rabidopsis thaliana." Plant, cell & environment 38, no. 2 (2015): 375-384.
Solyc03g118780.3	2.04	thaumatin-like protein	TLP1	Involved in local responses of roots to colonization by non-pathogenic plant growth-promoting rhizobacteria fluorescent Pseudomonas spp.	*Impaired rhizobacterial integration; Impaired growth/development*	Leon-Kloosterziel, Karen M., Bas WM Verhagen, Joost JB Keurentjes, Johan A. Van Pelt, Martijn Rep, L. C. Van Loon, and Corne MJ Pieterse. "Colonization of the Arabidopsis rhizosphere by fluorescent Pseudomonas spp. activates a root-specific, ethylene-responsive PR-5 gene in the vascular bundle." Plant molecular biology 57, no. 5 (2005): 731-748.
Solyc09g008320.3	0.92	probable xyloglucan endotransglucosylase/hydrolase protein 32	XTH32	Cleaves and religates xyloglucan polymers, an essential constituent of the primary cell wall, and thereby participates in cell wall construction of growing tissues	*Impaired plant cell wall construction; Impaired growth*	Bischoff, Volker, Sarah Jane Cookson, Shuang Wu, and Wolf-Rüdiger Scheible. "Thaxtomin A affects CESA-complex density, expression of cell wall genes, cell wall composition, and causes ectopic lignification in Arabidopsis thaliana seedlings." Journal of experimental botany 60, no. 3 (2009): 955-965.

TomatoID	DE in LsoB	NCBI Protein Name	Gene ID	Uniprot Description	Putative Consequences for Infection	Citation
Solyc05g005550.3	0.92	polygalacturonase non-catalytic subunit AroGP2 precursor	GP2	Non-catalytic subunit of polygalacturonase involved in cell wall organization	*Impaired plant cell wall organization; Impaired growth/development*	N/A
Solyc04g063210.3	0.88	probable caffeoyl-CoA O-methyltransferase At4g26220 isoform X2	AT4G26220	Plays a role in the synthesis of feruloylated polysaccharides; Involved in the reinforcement of the plant cell wall; Involved in response to wounding and/or pathogen challenge	*Impaired reinforcement of plant cell walls; Impaired growth; Impaired response to wounding and/or pathogen challenge*	Meyermans, Hugo, et al. "Modifications in lignin and accumulation of phenolic glucosides in poplar xylem upon down-regulation of caffeoyl-coenzyme A O-methyltransferase, an enzyme involved in lignin biosynthesis." Journal of Biological Chemistry 275.47 (2000): 36899-36909.
Solyc11g019910.1	0.84	pectinesterase inhibitor 4	PMEI4	Pectin methylesterase inhibitor that can target the root-expressed PME17; Regulates de-methylesterification of pectins in roots and affects root growth	*Impaired root growth/development*	Sénéchal, Fabien, Alain Mareck, Paulo Marcelo, Patrice Lerouge, and Jérôme Pelloux. "Arabidopsis PME17 activity can be controlled by pectin methylesterase inhibitor4." Plant signaling & behavior 10, no. 2 (2015): e983351.
Solyc05g014000.3	0.73	probable pectate lyase 5	At1g67750	Part of the pathway pectin degradation, which is itself part of Glycan metabolism	*Impaired pectin degradation; Impaired cell wall growth/development*	Pischke, Melissa S., Edward L. Huttlin, Adrian D. Hegeman, and Michael R. Sussman. "A transcriptome-based characterization of habituation in plant tissue culture." Plant Physiology 140, no. 4 (2006): 1255-1278.
Solyc10g074540.1	0.72	protein EXORDIUM-like 5	EXL5	Plays a role in a brassinosteroid-dependent regulation of growth and development	*Impaired growth and development*	Krinke, Ondrej, Eric Ruelland, Olga Valentová, Chantal Vergnolle, Jean-Pierre Renou, Ludivine Taconnat, Matyás Flemr, Lenka Burketová, and Alain Zachowski. "Phosphatidylinositol 4-kinase activation is an early response to salicylic acid in Arabidopsis suspension cells." Plant Physiology 144, no. 3 (2007): 1347-1359.
Solyc01g098740.3	0.71	probable serine/threonine-protein kinase PBL7	PBL7	Serine/threonine-protein kinase involved in the positive regulation of brassinosteroid signaling and plant growth	*Impaired brassinosteroid signaling; Impaired growth*	Kim, Tae-Wuk, Shenheng Guan, Alma L. Burlingame, and Zhi-Yong Wang. "The CDG1 kinase mediates brassinosteroid signal transduction from BRI1 receptor kinase to BSU1 phosphatase and GSK3-like kinase BIN2." Molecular cell 43, no. 4 (2011): 561-571.
Solyc05g009470.3	0.70	alpha-xylosidase 1	XYL1	Glycoside hydrolase releasing xylosyl residues from xyloglucan oligosaccharides; Essential for growth/development	*Impaired growth/development*	Sampedro, Javier, et al. "Cloning and expression pattern of a gene encoding an α-xylosidase active against xyloglucan oligosaccharides from Arabidopsis." Plant Physiology 126.2 (2001): 910-920.
Solyc04g081300.3	0.70	endoglucanase 2	At1g19940	Involved in cellulose catabolism; Involved in cell wall organization	*Impaired cellulose catabolism; Impaired cell wall organization*	Brown, David M., Leo AH Zeef, Joanne Ellis, Royston Goodacre, and Simon R. Turner. "Identification of novel genes in Arabidopsis involved in secondary cell wall formation using expression profiling and reverse genetics." The Plant Cell 17, no. 8 (2005): 2281-2295.
Solyc01g096040.3	0.65	aspartyl protease family protein 2	APF2	Involved in response to karrikin, a positive plant growth regulator	*Impaired plant growth*	Li, Yurong, Mehdi Kabbage, Wende Liu, and Martin B. Dickman. "Aspartyl protease-mediated cleavage of BAG6 is necessary for autophagy and fungal resistance in plants." The Plant Cell 28, no. 1 (2016): 233-247.

TomatoID	DE in LsoB	NCBI Protein Name	Gene ID	Uniprot Description	Putative Consequences for Infection	Citation
Solyc04g078520.3	0.64	protein RICE SALT SENSITIVE 3-like	RSS3	Represses jasmonate-induced genes; Involved in transcriptional regulation in the root tip; Regulates root cell elongation during salt stress	*Impaired repression of jasmonate-induced genes; Inceased jasmonate-related defense; Impaired root cell elongation*	Toda, Yosuke, Maiko Tanaka, Daisuke Ogawa, Kyo Kurata, Ken-ichi Kurotani, Yoshiki Habu, Tsuyu Ando et al. "RICE SALT SENSITIVE3 forms a ternary complex with JAZ and class-C bHLH factors and regulates jasmonate-induced gene expression and root cell elongation." The Plant Cell 25, no. 5 (2013): 1709-1725.
Solyc01g104400.3	0.61	basic blue protein	ARPN	Forms a concentration gradient along the pollen tube growth path	*Impaired pollen tube growth*	Dong, Juan, Sun Tae Kim, and Elizabeth M. Lord. "Plantacyanin plays a role in reproduction in Arabidopsis." Plant Physiology 138, no. 2 (2005): 778-789.
Solyc12g005020.2	0.60	NEP1-interacting protein-like 1 isoform X1	ATL27	Involved in the early steps of the plant defense signaling pathway	*Impaired plant defense*	Libault, Marc, Jinrong Wan, Tomasz Czechowski, Michael Udvardi, and Gary Stacey. "Identification of 118 Arabidopsis transcription factor and 30 ubiquitin-ligase genes responding to chitin, a plant-defense elicitor." Molecular plant-microbe interactions 20, no. 8 (2007): 900-911.
Solyc12g044310.2	0.57	protein NRT1/ PTR FAMILY 1.2	NPF1.2	Low-affinity nitrate transporter involved in xylem-to-phloem transfer for redistributing nitrate into developing leaves	*Impaired nitrate transport; Impaired growth/development*	Hsu, Po-Kai, and Yi-Fang Tsay. "Two phloem nitrate transporters, NRT1. 11 and NRT1. 12, are important for redistributing xylem-borne nitrate to enhance plant growth." Plant Physiology 163, no. 2 (2013): 844-856.
Solyc09g008860.3	0.56	leucine-rich repeat receptor-like protein kinase PXC1	PXC1	Involved in secondary cell wall formation in xylem fibers; Regulates interfascicular fiber cell maturation; Promotes secondary wall formation during cell expansion	*Impaired xylem growth; Impaired fiber cell maturation; Impaired cell expansion*	Wang, Jiehua, Melis Kucukoglu, Linbin Zhang, Peng Chen, Daniel Decker, Ove Nilsson, Brian Jones, Göran Sandberg, and Bo Zheng. "The Arabidopsis LRR-RLK, PXC1, is a regulator of secondary wall formation correlated with the TDIF-PXY/TDR-WOX4 signaling pathway." BMC plant biology 13, no. 1 (2013): 1-11.
Solyc02g080160.3	0.51	probable xyloglucan endotransglucosylase/hydrolase protein 8 isoform X2	XTH8	Cleaves and religates xyloglucan polymers, an essential constituent of the primary cell wall, and thereby participates in cell wall construction of growing tissues	*Impaired plant cell wall growth/development*	Bischoff, Volker, Sarah Jane Cookson, Shuang Wu, and Wolf-Rüdiger Scheible. "Thaxtomin A affects CESA-complex density, expression of cell wall genes, cell wall composition, and causes ectopic lignification in Arabidopsis thaliana seedlings." Journal of experimental botany 60, no. 3 (2009): 955-965.
Solyc06g008990.1	0.49	protein FANTASTIC FOUR 1-like	FAF1	Represses WUS, a gene related to plant organ development, when constitutively overexpressed	*Impaired growth and development*	Wahl, Vanessa, Luise H. Brand, Ya-Long Guo, and Markus Schmid. "The FANTASTIC FOUR proteins influence shoot meristem size in Arabidopsis thaliana." BMC Plant Biology 10, no. 1 (2010): 1-12.
Solyc07g062260.3	0.47	BES1/BZR1 homolog protein 4	BEH4	Involved in brassinosteroid mediated signaling pathway	*Impaired plant development and physiological regulation*	Wang, Zhi-Yong, Takeshi Nakano, Joshua Gendron, Junxian He, Meng Chen, Dionne Vafeados, Yanli Yang et al. "Nuclear-localized BZR1 mediates brassinosteroid-induced growth and feedback suppression of brassinosteroid biosynthesis." Developmental cell 2, no. 4 (2002): 505-513.
Solyc04g016200.1	0.46	zeatin O-glucosyltransferase	ZOG1	Regulates active vs storage forms of cytokinins	*Impaired seed development*	Hou, Bingkai, Eng-Kiat Lim, Gillian S. Higgins, and Dianna J. Bowles. "N-glucosylation of cytokinins by glycosyltransferases of Arabidopsis thaliana." Journal of Biological Chemistry 279, no. 46 (2004): 47822-47832.

TomatoID	DE in LsoB	NCBI Protein Name	Gene ID	Uniprot Description	Putative Consequences for Infection	Citation
Solyc10g080940.2	0.46	tubulin beta-5 chain	TUBB5	Tubulin is the major constituent of microtubules	*Impaired microtubule development*	Cao, Dongni, Hui Cheng, Wei Wu, Hui Meng Soo, and Jinrong Peng. "Gibberellin mobilizes distinct DELLA-dependent transcriptomes to regulate seed germination and floral development in Arabidopsis." Plant physiology 142, no. 2 (2006): 509-525.
Solyc12g027550.1	0.45	photosystem II CP43 chlorophyll apoprotein	psbC	Photosynthetic electron transporter in photosystem II	*Impaired photosynthesis*	Xu, Hong, Dmitrii Vavilin, and Wim Vermaas. "Chlorophyll b can serve as the major pigment in functional photosystem II complexes of cyanobacteria." Proceedings of the National Academy of Sciences 98, no. 24 (2001): 14168-14173.
Solyc05g005560.4	0.41	polygalacturonase-1 non-catalytic subunit beta precursor	GP2	Non-catalytic subunit of polygalacturonase involved in cell wall organization	*Impaired plant cell wall organization; Impaired growth/development*	N/A
Solyc03g123630.3	0.41	pectinesterase/pectinesterase inhibitor U1 precursor	PMEU1	Acts in the modification of cell walls via demethylesterification of cell wall pectin	*Impaired plant cell wall modification/development; Impaired growth/development*	Phan, Thanh D., Wen Bo, Gill West, Grantley W. Lycett, and Gregory A. Tucker. "Silencing of the major salt-dependent isoform of pectinesterase in tomato alters fruit softening." Plant physiology 144, no. 4 (2007): 1960-1967.
Solyc01g110340.3	0.41	endoglucanase 24-like	At4g39010	Involved in fruit dehiscence and fruit valve development; Involved in plant cell wall loosening	*Impaired fruit development*	Urbanowicz, Breeanna R., Alan B. Bennett, Elena Del Campillo, Carmen Catalá, Takahisa Hayashi, Bernard Henrissat, Herman Höfte et al. "Structural organization and a standardized nomenclature for plant endo-1, 4-β-glucanases (cellulases) of glycosyl hydrolase family 9." Plant Physiology 144, no. 4 (2007): 1693-1696.
Solyc06g034390.1	0.40	protein EXORDIUM-like 3	EXL3	Plays a role in a brassinosteroid-dependent regulation of growth and development	*Impaired growth and development*	Krinke, Ondrej, Eric Ruelland, Olga Valentová, Chantal Vergnolle, Jean-Pierre Renou, Ludivine Taconnat, Matyás Flemr, Lenka Burketová, and Alain Zachowski. "Phosphatidylinositol 4-kinase activation is an early response to salicylic acid in Arabidopsis suspension cells." Plant Physiology 144, no. 3 (2007): 1347-1359.
Solyc07g052980.3	0.39	xyloglucan endotransglycosylase/hydrolase 16 precursor	XTH16	Cleaves and religates xyloglucan polymers, an essential constituent of the primary cell wall, and thereby participates in cell wall construction of growing tissues	*Impaired plant cell wall expansion; Impaired growth/development*	Sasidharan, Rashmi, C. C. Chinnappa, Marten Staal, J. Theo M. Elzenga, Ryusuke Yokoyama, Kazuhiko Nishitani, Laurentius ACJ Voesenek, and Ronald Pierik. "Light quality-mediated petiole elongation in Arabidopsis during shade avoidance involves cell wall modification by xyloglucan endotransglucosylase/hydrolases." Plant physiology 154, no. 2 (2010): 978-990.
Solyc02g090960.1	0.36	protein RALF-like 34	RALFL34	Cell signaling peptide that regulates plant stress, growth, and development	*Impaired response to plant stress, growth, and development*	Murphy, Evan, Lam Dai Vu, Lisa Van den Broeck, Zhefeng Lin, Priya Ramakrishna, Brigitte Van De Cotte, Allison Gaudinier et al. "RALFL34 regulates formative cell divisions in Arabidopsis pericycle during lateral root initiation." Journal of Experimental Botany 67, no. 16 (2016): 4863-4875.

TomatoID	DE in LsoB	NCBI Protein Name	Gene ID	Uniprot Description	Putative Consequences for Infection	Citation
Solyc06g062960.2	0.35	protein RALF-like 34	RALFL34	Cell signaling peptide that may regulate plant stress, growth, and development	*Impaired response to plant stress, growth, and development*	Murphy, Evan, Lam Dai Vu, Lisa Van den Broeck, Zhefeng Lin, Priya Ramakrishna, Brigitte Van De Cotte, Allison Gaudinier et al. "RALFL34 regulates formative cell divisions in Arabidopsis pericycle during lateral root initiation." Journal of Experimental Botany 67, no. 16 (2016): 4863-4875.
Solyc09g009010.3	0.35	glucomannan 4-beta-mannosyltransferase 9-like isoform X2	CSLA9	Required for lateral root development; Involved in cell wall organization; Involved in response to bacteria	*Impaired lateral root development; Impaired plant cell wall organization; Impaired defense response to bacteria*	Liepman, Aaron H., C. Joseph Nairn, William GT Willats, Iben Sørensen, Alison W. Roberts, and Kenneth Keegstra. "Functional genomic analysis supports conservation of function among cellulose synthase-like a gene family members and suggests diverse roles of mannans in plants." Plant Physiology 143, no. 4 (2007): 1881-1893.
Solyc09g008990.3	0.35	glucomannan 4-beta-mannosyltransferase 9-like isoform X2	CSLA9	Required for lateral root development; Involved in cell wall organization; Involved in response to bacteria	*Impaired lateral root development; Impaired plant cell wall organization; Impaired defense response to bacteria*	Liepman, Aaron H., C. Joseph Nairn, William GT Willats, Iben Sørensen, Alison W. Roberts, and Kenneth Keegstra. "Functional genomic analysis supports conservation of function among cellulose synthase-like a gene family members and suggests diverse roles of mannans in plants." Plant Physiology 143, no. 4 (2007): 1881-1893.
Solyc02g078850.1	0.34	glycine-rich cell wall structural protein 1.8	At3g17050	Responsible for plasticity of plant cell wall	*Impaired plant cell wall modification/development*	Quigley, Françoise, Marie-Louise Villiot, and Régis Mache. "Nucleotide sequence and expression of a novel glycine-rich protein gene from Arabidopsis thaliana." Plant molecular biology 17, no. 4 (1991): 949-952.
Solyc12g099260.2	0.34	ATP-citrate synthase beta chain protein 2-like	ACLA-2	Used for the elongation of fatty acids and biosynthesis of isoprenoids, flavonoids, and malonated derivatives; Required for normal growth and development in seeds	*Impaired seed development; Impaired production of primary metabolites*	Fatland, Beth L., Basil J. Nikolau, and Eve Syrkin Wurtele. "Reverse genetic characterization of cytosolic acetyl-CoA generation by ATP-citrate lyase in Arabidopsis." The Plant Cell 17.1 (2005): 182-203.
Solyc11g066720.2	0.33	UDP-D-apiose/UDP-D-xylose synthase 2	AXS2	Catalyzes the conversion of UDP-D-glucuronate to a mixture of UDP-D-apiose and UDP-D-xylose, a plant cell wall monosaccharide with a unique role	*Impaired plant cell wall modification/development*	Mølhøj, Michael, Rajeev Verma, and Wolf-Dieter Reiter. "The biosynthesis of the branched-chain sugar d-apiose in plants: functional cloning and characterization of a UDP-d-apiose/UDP-d-xylose synthase from Arabidopsis." The Plant Journal 35, no. 6 (2003): 693-703.
Solyc10g061830.2	0.32	photosystem II 44 kDa protein	psbC	Photosynthetic electron transporter in photosystem II	*Impaired photosynthesis*	Ahmed, Ibrar, Peter J. Matthews, Patrick J. Biggs, Muhammad Naeem, Patricia A. McLenachan, and Peter J. Lockhart. "Identification of chloroplast genome loci suitable for high-resolution phylogeographic studies of C olocasia esculenta (L.) S chott (A raceae) and closely related taxa." Molecular Ecology Resources 13, no. 5 (2013): 929-937.
Solyc04g077020.3	0.28	tubulin alpha chain	TUBA1	Tubulin is the major constituent of microtubules	*Impaired microtubule development*	Saito, Yuka, Kouichi Soga, Kazuyuki Wakabayashi, and Takayuki Hoson. "Increase in expression level of alpha-tubulin gene in Arabidopsis seedlings under hypergravity conditions." Uchu Seibutsu Kagaku 17, no. 3 (2003): 177-178.

TomatoID	DE in LsoB	NCBI Protein Name	Gene ID	Uniprot Description	Putative Consequences for Infection	Citation
Solyc02g030480.3	0.28	probable cinnamyl alcohol dehydrogenase 6	CAD6	Involved in lignin biosynthesis	*Impaired lignin biosynthesis; Impaired plant cell wall growth/development*	Costa, Michael A., R. Eric Collins, Aldwin M. Anterola, Fiona C. Cochrane, Laurence B. Davin, and Norman G. Lewis. "An in silico assessment of gene function and organization of the phenylpropanoid pathway metabolic networks in Arabidopsis thaliana and limitations thereof." Phytochemistry 64, no. 6 (2003): 1097-1112.
Solyc01g111350.3	0.28	protein NUCLEAR FUSION DEFECTIVE 4	NFD4	Required for karyogamy during female gametophyte development	*Impaired female gametophyte development*	Portereiko, Michael F., Linda Sandaklie-Nikolova, Alan Lloyd, Chad A. Dever, Denichiro Otsuga, and Gary N. Drews. "NUCLEAR FUSION DEFECTIVE1 encodes the Arabidopsis RPL21M protein and is required for karyogamy during female gametophyte development and fertilization." Plant physiology 141, no. 3 (2006): 957-965.
Solyc02g065170.3	0.27	L-ascorbate oxidase homolog	AAO	Represses responses to high salinity and oxidative stress conditions such as vegetative growth and seed production reductions	*Impaired regulation of stress responses; Impaired growth/development under stress*	Yamamoto, Atsuko, Md Nazmul H. Bhuiyan, Rungaroon Waditee, Yoshito Tanaka, Muneharu Esaka, Kazuko Oba, André T. Jagendorf, and Teruhiro Takabe. "Suppressed expression of the apoplastic ascorbate oxidase gene increases salt tolerance in tobacco and Arabidopsis plants." Journal of Experimental Botany 56, no. 417 (2005): 1785-1796.
Solyc04g053000.1	0.27	auxin-responsive protein SAUR21-like	SAUR21	Positive effectors of cell expansion through modulation of auxin transport	*Impaired plant cell wall expansion; Impaired growth/development; Impaired auxin transport*	Osakabe, Yuriko, Kyonoshin Maruyama, Motoaki Seki, Masakazu Satou, Kazuo Shinozaki, and Kazuko Yamaguchi-Shinozaki. "Leucine-rich repeat receptor-like kinase1 is a key membrane-bound regulator of abscisic acid early signaling in Arabidopsis." The Plant Cell 17, no. 4 (2005): 1105-1119.
Solyc03g112880.1	0.27	fasciclin-like arabinogalactan protein 4	FLA4	Cell surface adhesion protein required for normal cell expansion	*Impaired cell expansion; Impaired growth/development*	Xu, Shou-Ling, et al. "Two leucine-rich repeat receptor kinases mediate signaling, linking cell wall biosynthesis and ACC synthase in Arabidopsis." The Plant Cell 20.11 (2008): 3065-3079.
Solyc05g005710.3	0.26	spermidine synthase	SPDS1	Involved in polyamine biosynthetic process	*Impaired polyamine biosynthesis; Impaired cell proliferation*	Cheng, Chia-Yi, Vivek Krishnakumar, Agnes P. Chan, Françoise Thibaud-Nissen, Seth Schobel, and Christopher D. Town. "Araport11: a complete reannotation of the Arabidopsis thaliana reference genome." The Plant Journal 89, no. 4 (2017): 789-804.
Solyc01g102330.3	0.24	probable carbohydrate esterase At4g34215	At4g34215	Involved in secondary plant cell wall biosynthesis	*Impaired secondary plant cell wall biosynthesis; Impaired growth*	Andersson-Gunnerås, Sara, Ewa J. Mellerowicz, Jonathan Love, Bo Segerman, Yasunori Ohmiya, Pedro M. Coutinho, Peter Nilsson, Bernard Henrissat, Thomas Moritz, and Björn Sundberg. "Biosynthesis of cellulose-enriched tension wood in Populus: global analysis of transcripts and metabolites identifies biochemical and developmental regulators in secondary wall biosynthesis." The Plant Journal 45, no. 2 (2006): 144-165.

TomatoID	DE in LsoB	NCBI Protein Name	Gene ID	Uniprot Description	Putative Consequences for Infection	Citation
Solyc02g087190.1	0.22	peroxidase 63	PER63	Involved in oxidation of toxic reductants, lignin metabolism, suberization, auxin catabolism; Involved in response to environmental stresses	*Impaired plant defense; Impaired lignin metabolism; Impaired cell wall expansion; Impaired growth/development; Impaired auxin catabolism*	Valério, Luisa, Mireille De Meyer, Claude Penel, and Christophe Dunand. "Expression analysis of the Arabidopsis peroxidase multigenic family." Phytochemistry 65, no. 10 (2004): 1331-1342.
Solyc09g014490.3	0.22	endochitinase A	At2g43590	Involved in plant cell wall macromolecule catabolism and chitin catabolism; Involved in defense response	*Impaired plant cell wall development; Impaired defense response*	Krysan, Patrick J., Peter J. Jester, Jennifer R. Gottwald, and Michael R. Sussman. "An Arabidopsis mitogen-activated protein kinase kinase kinase gene family encodes essential positive regulators of cytokinesis." The Plant Cell 14, no. 5 (2002): 1109-1120.
Solyc03g044150.3	0.20	subtilisin-like protease SBT1.7	SBT1.7	Serine protease essential for mucilage release from seed coats	*Impaired seed coat development*	Rautengarten, Carsten, Björn Usadel, Lutz Neumetzler, Jürgen Hartmann, Dirk Büssis, and Thomas Altmann. "A subtilisin-like serine protease essential for mucilage release from Arabidopsis seed coats." The Plant Journal 54, no. 3 (2008): 466-480.
Solyc12g009110.2	0.20	acetylserotonin O-methyltransferase	ASMT	Methyltransferase which catalyzes the production of melatonin; Involved in response to light stress	*Impaired development under light stress*	Shi, Haitao, Yunxie Wei, and Chaozu He. "Melatonin-induced CBF/DREB1s are essential for diurnal change of disease resistance and CCA1 expression in Arabidopsis." Plant Physiology and Biochemistry 100 (2016): 150-155.
Solyc11g066820.2	0.14	glucomannan 4-beta-mannosyltransferase 2	CSLA2	Possesses glucomannan synthase and mannan synthase activities; Galactomannan is a noncellulosic polysaccharides of plant cell wall	*Impaired plant cell wall growth/development*	Liepman, Aaron H., Curtis G. Wilkerson, and Kenneth Keegstra. "Expression of cellulose synthase-like (Csl) genes in insect cells reveals that CslA family members encode mannan synthases." Proceedings of the National Academy of Sciences 102, no. 6 (2005): 2221-2226.
Solyc01g006370.3	0.14	callose synthase 3-like	CALS3	Involved in callose synthesis at the forming cell plate during cytokinesis	*Impaired callose synthesis; Impaired cell proliferation; Impaired growth/development*	Chen, Xiong-Yan, Lin Liu, EunKyoung Lee, Xiao Han, Yeonggil Rim, Hyosub Chu, Seon-Won Kim, Fred Sack, and Jae-Yean Kim. "The Arabidopsis callose synthase gene GSL8 is required for cytokinesis and cell patterning." Plant Physiology 150, no. 1 (2009): 105-113.
Solyc10g086650.1	0.12	aldehyde oxidase GLOX	GLOX1	Regulated by the transcription factor MYB80 during anther development and plays a role in tapetum and pollen development	*Impaired pollen development*	Phan, Huy Anh, Sylvana Iacuone, Song F. Li, and Roger W. Parish. "The MYB80 transcription factor is required for pollen development and the regulation of tapetal programmed cell death in Arabidopsis thaliana." The Plant Cell 23, no. 6 (2011): 2209-2224.
Solyc07g055950.3	0.12	protodermal factor 1	PDF1	Involved in the regulation of meristem growth	*Impaired growth and development*	Pholo, Motlalepula, Beatrix Coetzee, Hans J. Maree, Philip R. Young, James R. Lloyd, Jens Kossmann, and Paul N. Hills. "Cell division and turgor mediate enhanced plant growth in Arabidopsis plants treated with the bacterial signalling molecule lumichrome." Planta 248, no. 2 (2018): 477-488.
Solyc11g013270.2	0.12	O-fucosyltransferase 10-like	FUT10	Involved in cell wall biosynthesis	*Impaired plant cell wall growth/development*	Sarria, Rodrigo, Tanya A. Wagner, Malcolm A. O'Neill, Ahmed Faik, Curtis G. Wilkerson, Kenneth Keegstra, and Natasha V. Raikhel. "Characterization of a family of Arabidopsis genes related to xyloglucan fucosyltransferase1." Plant Physiology 127, no. 4 (2001): 1595-1606.

TomatoID	DE in LsoB	NCBI Protein Name	Gene ID	Uniprot Description	Putative Consequences for Infection	Citation
Solyc04g016480.3	0.10	protein IQ-DOMAIN 14	IQD14	Involved in cooperative interactions with calmodulins or calmodulin-like proteins; Scaffold in cellular signaling and trafficking; Regulates cell and organ shapes	*Impaired cell-to-cell signaling and trafficking; Impaired regulation of plant organ shape*	Bürstenbinder, Katharina, Birgit Möller, Romina Plötner, Gina Stamm, Gerd Hause, Dipannita Mitra, and Steffen Abel. "The IQD family of calmodulin-binding proteins links calcium signaling to microtubules, membrane subdomains, and the nucleus." Plant physiology 173, no. 3 (2017): 1692-1708.
Solyc06g071500.3	0.09	probable boron transporter 2	BOR2	Boron transporter essential for maintaining the integrity of plants cell walls	*Impaired plant cell wall growth/development*	Takano, Junpei, Kyotaro Noguchi, Miho Yasumori, Masaharu Kobayashi, Zofia Gajdos, Kyoko Miwa, Hiroaki Hayashi, Tadakatsu Yoneyama, and Toru Fujiwara. "Arabidopsis boron transporter for xylem loading." Nature 420, no. 6913 (2002): 337-340.
Solyc01g106580.2	0.08	probable pectin methylesterase CGR3	CGR3	Together with CGR2, required for homogalacturonan pectins (HG) methylesterification in the Golgi apparatus prior to integration into cell walls, essential for general growth and development	*Impaired growth and development; Impaired plant cell wall growth/development*	M. Weraduwage, Sarathi, Sang-Jin Kim, Luciana Renna, Fransisca C. Anozie, Thomas D. Sharkey, and Federica Brandizzi. "Pectin methylesterification impacts the relationship between photosynthesis and plant growth." Plant Physiology 171, no. 2 (2016): 833-848.
Solyc08g061930.3	0.07	cytokinin oxidase/dehydrogenase-like isoform X2	CKX2	Involved in cytokinin catabolism	*Impaired cytokinin catabolism; Impaired plant root/shoot development*	Werner, Tomáš, Václav Motyka, Valérie Laucou, Rafaël Smets, Harry Van Onckelen, and Thomas Schmülling. "Cytokinin-deficient transgenic Arabidopsis plants show multiple developmental alterations indicating opposite functions of cytokinins in the regulation of shoot and root meristem activity." The Plant Cell 15, no. 11 (2003): 2532-2550.
Solyc04g081490.3	0.07	beta-tubulin	TUBB1	Tubulin is the major constituent of microtubules	*Impaired microtubule development*	Cao, Dongni, Hui Cheng, Wei Wu, Hui Meng Soo, and Jinrong Peng. "Gibberellin mobilizes distinct DELLA-dependent transcriptomes to regulate seed germination and floral development in Arabidopsis." Plant physiology 142, no. 2 (2006): 509-525.
Solyc09g015080.3	0.05	patellin-6	PATL6	Carrier protein involved in membrane-trafficking associated with cell-plate formation during cytokinesis; Involved in cellular division; Involved in auxin polar transport	*Impaired cellular division; Impaired growth/development; Impaired auxin transport*	Zhou, Huapeng, Hongqin Duan, Yunhong Liu, Xia Sun, Jinfeng Zhao, and Honghui Lin. "Patellin protein family functions in plant development and stress response." Journal of plant physiology 234 (2019): 94-97.
Solyc04g053010.1	0.04	auxin-responsive protein SAUR68-like	SAUR68	Promote auxin-stimulated organ elongation, such as hypocotyls, stamen filaments and petals	*Impaired organ elongation; Impaired growth and development; Impaired auxin signaling; Impaired flower development*	Zhao, Yunde, Xinhua Dai, Helen E. Blackwell, Stuart L. Schreiber, and Joanne Chory. "SIR1, an upstream component in auxin signaling identified by chemical genetics." Science 301, no. 5636 (2003): 1107-1110.
Solyc02g085870.3	-1.00	3-ketoacyl-CoA synthase 6	CUT1	Major condensing enzyme for stem wax and pollen coat lipid biosynthesis	*Increased wax and pollen coat biosynthesis; Cell wall expansion; Impaired growth/development*	Kunst, Ljerka, and A. Lacey Samuels. "Biosynthesis and secretion of plant cuticular wax." Progress in lipid research 42, no. 1 (2003): 51-80.

TomatoID	DE in LsoB	NCBI Protein Name	Gene ID	Uniprot Description	Putative Consequences for Infection	Citation
Solyc06g075310.3	-1.02	adenylate kinase isoenzyme 6 homolog isoform X1	AAK6	Kinase that catalyzes the reversible transfer of the terminal phosphate group between nucleoside triphosphates and monophosphates	Promoted stem growth	Feng, Xue, Ruonan Yang, Xiaofeng Zheng, and FeiYun Zhang. "Identification of a novel nuclear-localized adenylate kinase 6 from Arabidopsis thaliana as an essential stem growth factor." Plant physiology and biochemistry 61 (2012): 180-186.
Solyc12g099900.1	-1.05	scarecrow-like protein 3	SCL3	Transcription factor involved in plant development	Promoted plant growth and development	Zhang, Zhong-Lin, Mikihiro Ogawa, Christine M. Fleet, Rodolfo Zentella, Jianhong Hu, Jung-Ok Heo, Jun Lim, Yuji Kamiya, Shinjiro Yamaguchi, and Tai-ping Sun. "Scarecrow-like 3 promotes gibberellin signaling by antagonizing master growth repressor DELLA in Arabidopsis." Proceedings of the National Academy of Sciences 108, no. 5 (2011): 2160-2165.
Solyc07g032480.3	-1.06	cyclin-T1-3	CYCT1-3	Involved in cellular division; Positive regulator of DNA-templated transcription; Regulates transcription of RNA polymerase II	Promoted cellular division; Increased DNA-templated transcription	Cui, Xiaofeng, Baofang Fan, James Scholz, and Zhixiang Chen. "Roles of Arabidopsis cyclin-dependent kinase C complexes in cauliflower mosaic virus infection, plant growth, and development." The Plant Cell 19, no. 4 (2007): 1388-1402.
Solyc02g077080.3	-1.07	protein trichome birefringence-like 38	TBL38	Bridging protein that binds pectin and other cell wall polysaccharides	Promoted pectin-binding; Promoted plant cell wall growth and development	Bischoff, Volker, Joachim Selbig, and Wolf-Rüdiger Scheible. "Involvement of TBL/DUF231 proteins into cell wall biology." Plant signaling & behavior 5, no. 8 (2010): 1057-1059.
Solyc07g006130.2	-1.08	eukaryotic peptide chain release factor subunit 1-3-like	ERF1-3	Directs the termination of nascent peptide synthesis; Modulates plant growth and development	Promoted plant growth and development	Chapman, Bernice, and Chris Brown. "Translation termination in Arabidopsis thaliana: characterisation of three versions of release factor 1." Gene 341 (2004): 219-225.
Solyc05g046290.3	-1.14	probable xyloglucan endotransglucosylase/hydrolase protein 23	XTH23	Cleaves and religates xyloglucan polymers, an essential constituent of the primary cell wall, and thereby participates in cell wall construction of growing tissues	Promoted plant growth and development	Bischoff, Volker, Sarah Jane Cookson, Shuang Wu, and Wolf-Rüdiger Scheible. "Thaxtomin A affects CESA-complex density, expression of cell wall genes, cell wall composition, and causes ectopic lignification in Arabidopsis thaliana seedlings." Journal of experimental botany 60, no. 3 (2009): 955-965.
Solyc01g111640.3	-1.14	SKP1-like protein 1A	SKP1A	Involved in ubiquitination and subsequent proteasomal degradation of target proteins; Required for vegetative and floral organ development as well as for male gametogenesis; Involved in auxin signaling pathway; Regulates responses to jasmonates; Involved in light-signaling and the circadian clock; Plays a role during embryogenesis and early postembryonic development, especially during cell elongation and division	Promoted protein ubiquitination; Promoted floral development and male gametogenesis; Promoted auxin signaling; Promoted circadian clock; Promoted embryogenesis	Zhao, Dazhong, Weimin Ni, Baomin Feng, Tianfu Han, Megan G. Petrasek, and Hong Ma. "Members of the Arabidopsis-SKP1-like gene family exhibit a variety of expression patterns and may play diverse roles in Arabidopsis." Plant physiology 133, no. 1 (2003): 203-217.

TomatoID	DE in LsoB	NCBI Protein Name	Gene ID	Uniprot Description	Putative Consequences for Infection	Citation
Solyc03g114960.3	-1.22	tip elongation aberrant protein 1 isoform X2	tea1	Acts as an end marker, directing the growth machinery to the cell poles; Involved in the regulation of microtubular organization, affecting the maintenance of a single central axis	Promoted cell elongation; Promoted microtubule development	Mata, Juan, and Paul Nurse. "tea1 and the microtubular cytoskeleton are important for generating global spatial order within the fission yeast cell." Cell 89, no. 6 (1997): 939-949.
Solyc06g008870.2	-1.26	gibberellin receptor GID1b-2	GID1B	Soluble gibberellin GA receptor; Regulates growth and development	Promoted growth/development	Griffiths, Jayne, Kohji Murase, Ivo Rieu, Rodolfo Zentella, Zhong-Lin Zhang, Stephen J. Powers, Fan Gong et al. "Genetic characterization and functional analysis of the GID1 gibberellin receptors in Arabidopsis." The Plant Cell 18, no. 12 (2006): 3399-3414.
Solyc04g007980.3	-1.47	1-aminocyclopropane-1-carboxylate oxidase homolog 4	ACO4	Involved in ethylene biosynthesis; Promotes stem elongation by maximizing the extensibility cells	Promoted ethylene biosynthesis; Promotes stem elongation and cell extensibility	Qin, Yong-Mei, Chun-Yang Hu, Yu Pang, Alexander J. Kastaniotis, J. Kalervo Hiltunen, and Yu-Xian Zhu. "Saturated very-long-chain fatty acids promote cotton fiber and Arabidopsis cell elongation by activating ethylene biosynthesis." The Plant Cell 19, no. 11 (2007): 3692-3704.
Solyc12g045020.2	-1.57	cytochrome P450 CYP736A12	CYP734A1	Cytochrome P450 involved in brassinosteroids inactivation and regulation of BRs homeostasis	*Promoted brassinosteroid inactivation; Promoted brassinosteroid homeostasis; Impaired growth/development*	Neff, Michael M., Serena M. Nguyen, Elizabeth J. Malancharuvil, Shozo Fujioka, Takahiro Noguchi, Hideharu Seto, Masayoshi Tsubuki et al. "BAS1: A gene regulating brassinosteroid levels and light responsiveness in Arabidopsis." Proceedings of the National Academy of Sciences 96, no. 26 (1999): 15316-15323.
Solyc08g078870.2	-1.49	14 kDa proline-rich protein DC2.15-like	N/A	Involved in the initiation of embryogenesis and the metabolic changes related to the removal of auxins	Promoted embryogenesis; Promoted auxin removal	Aleith, F., and G. Richter. "Gene expression during induction of somatic embryogenesis in carrot cell suspensions." Planta 183, no. 1 (1991): 17-24.
Solyc04g007000.1	-1.69	AP2/ERF and B3 domain-containing transcription factor RAV1	RAV1	Negative regulator of plant growth and development	*Impaired growth and development*	Feng, C.Z., Chen, Y., Wang, C., Kong, Y.H., Wu, W.H. and Chen, Y.F., 2014. Arabidopsis RAV 1 transcription factor, phosphorylated by S n RK 2 kinases, regulates the expression of ABI 3, ABI 4, and ABI 5 during seed germination and early seedling development. The Plant Journal, 80(4), pp.654-668.
Solyc02g068470.1	-1.75	VQ motif-containing protein 22	VQ22	Functions as a positive regulator of plant growth	Promoted plant growth	Cheng, Yuan, Yuan Zhou, Yan Yang, Ying-Jun Chi, Jie Zhou, Jian-Ye Chen, Fei Wang et al. "Structural and functional analysis of VQ motif-containing proteins in Arabidopsis as interacting proteins of WRKY transcription factors." Plant physiology 159, no. 2 (2012): 810-825.
Solyc03g114890.3	-1.84	COBRA-like protein 4 isoform X2	COBL4	Involved in cellulose microfibril organization; Involved in plant cell wall cellulose biosynthesis; Invovled in secondary cell wall biogenesis	Promoted plant cell wall cellulose biosynthesis; Promoted scondary cell wall biogenesis; Promoted growth/development	Brown, David M., Leo AH Zeef, Joanne Ellis, Royston Goodacre, and Simon R. Turner. "Identification of novel genes in Arabidopsis involved in secondary cell wall formation using expression profiling and reverse genetics." The Plant Cell 17, no. 8 (2005): 2281-2295.

TomatoID	DE in LsoB	NCBI Protein Name	Gene ID	Uniprot Description	Putative Consequences for Infection	Citation
Solyc04g040130.1	-1.86	delta(12)-fatty-acid desaturase FAD2-like	FAD2	Required for desaturation of fatty acids present in extraplastidial membranes, including mitochondria; Required for salt tolerance during seed germination and early seedling growth	Promoted salt tolerance during seed germination and early growth	Okuley, John, Jonathan Lightner, Kenneth Feldmann, Narendra Yadav, Ellen Lark, and John Browse. "Arabidopsis FAD2 gene encodes the enzyme that is essential for polyunsaturated lipid synthesis." The Plant Cell 6, no. 1 (1994): 147-158.
Solyc10g076550.1	-1.90	wall-associated receptor kinase 2-like	WAK2	Serine/threonine-protein kinase that functions as a signaling receptor of extracellular matrix component; Involved in the control of cell expansion, morphogenesis, and development	Promoted signaling turnover; Promoted cell expansion, morphogenesis, and development	Wagner, Tanya A., and Bruce D. Kohorn. "Wall-associated kinases are expressed throughout plant development and are required for cell expansion." The Plant Cell 13, no. 2 (2001): 303-318.
Solyc12g096570.1	-2.52	protein AUXIN-REGULATED GENE INVOLVED IN ORGAN SIZE	ARGOS	Promotes cell proliferation-dependent organ growth; Takes part in the AXR1-dependent auxin signaling pathway that requires ANT during organogenesis	Promoted organ growth; Promoted auxin signaling	Hu, Yuxin, Qi Xie, and Nam-Hai Chua. "The Arabidopsis auxin-inducible gene ARGOS controls lateral organ size." The Plant Cell 15, no. 9 (2003): 1951-1961.
Solyc03g033840.3	-2.57	AAA-ATPase At3g50940-like	At3g50940	Involved in lignin biosynthesis and response to salt stress	Promoted lignin biosynthesis; Promoted cell wall expansion; Promoted growth/development Promoted response to salt stress	van de Mortel, Judith E., Laia Almar Villanueva, Henk Schat, Jeroen Kwekkeboom, Sean Coughlan, Perry D. Moerland, Emiel Ver Loren van Themaat, Maarten Koornneef, and Mark GM Aarts. "Large expression differences in genes for iron and zinc homeostasis, stress response, and lignin biosynthesis distinguish roots of Arabidopsis thaliana and the related metal hyperaccumulator Thlaspi caerulescens." Plant physiology 142, no. 3 (2006): 1127-1147.
Solyc12g100250.2	-2.78	delta(12)-fatty-acid desaturase FAD2 isoform X1	FAD2	Required for desaturation of fatty acids present in extraplastidial membranes, including mitochondria; Required for salt tolerance during seed germination and early seedling growth	Promoted salt tolerance during seed germination and early growth	Okuley, John, Jonathan Lightner, Kenneth Feldmann, Narendra Yadav, Ellen Lark, and John Browse. "Arabidopsis FAD2 gene encodes the enzyme that is essential for polyunsaturated lipid synthesis." The Plant Cell 6, no. 1 (1994): 147-158.
Solyc12g100240.1	-2.78	delta(12)-fatty-acid desaturase FAD2-like	FAD2	Required for desaturation of fatty acids present in extraplastidial membranes, including mitochondria; Required for salt tolerance during seed germination and early seedling growth	Promoted salt tolerance during seed germination and early growth	Okuley, John, Jonathan Lightner, Kenneth Feldmann, Narendra Yadav, Ellen Lark, and John Browse. "Arabidopsis FAD2 gene encodes the enzyme that is essential for polyunsaturated lipid synthesis." The Plant Cell 6, no. 1 (1994): 147-158.
Solyc12g100260.1	-3.19	delta(12)-fatty-acid desaturase FAD2-like	FAD2	Required for desaturation of fatty acids present in extraplastidial membranes, including mitochondria; Required for salt tolerance during seed germination and early seedling growth	Promoted salt tolerance during seed germination and early growth	Okuley, John, Jonathan Lightner, Kenneth Feldmann, Narendra Yadav, Ellen Lark, and John Browse. "Arabidopsis FAD2 gene encodes the enzyme that is essential for polyunsaturated lipid synthesis." The Plant Cell 6, no. 1 (1994): 147-158.
