# Supplementary material for: Effects of ‘Candidatus Liberibacter solanacearum’ haplotypes A and B on tomato gene expression and geotropism
Source: BMC Plant Biol. 2022 Mar 30;22:156. doi: 10.1186/s12870-022-03505-z (PMC8966271; doi:10.1186/s12870-022-03505-z)
Supplement: Supplementary file 13 — Additional file 13: Table S13. The 61 tomato plant DEGs associated with plant defense response to abiotic and biotic stress under infection by different Lso haplotypes. DEGs were sorted by differential expression (DE) values comparing uninfected to infected samples (p-value < 0.05). NCBI Blast searches were used to identify Gene IDs and protein products in tomatoes as well as their homologs in other species. Specifically, the expression changes in 32 of these genes (52%, in italics) would have resulted in impaired responsiveness to or promoted defense against stressors in LsoB-infected plants relative to LsoA-infected plants. These DEGs were predominantly related to defense against pathogens and response to drought, temperature, and salt stress. [file 12870_2022_3505_MOESM13_ESM.pdf]

TomatoID	DE in LsoB	NCBI Protein Name	Gene ID	Uniprot Description	Putative Consequences for Infection	Citation
Solyc09g089520.3	4.23	proteinase inhibitor I-B-like	LOC107794480	Involved in response to wounding	*Impaired response to wounding*	Sierro, Nicolas, et al. "The tobacco genome sequence and its comparison with those of tomato and potato." Nature communications 5.1 (2014): 1-9.
Solyc11g071480.1	3.83	agmatine hydroxycinnamoyltransferase 1-like	AHT1	Promotes the synthesis of hydroxycinnamic acid amides, which play a role in defense against pathogens	*Impaired defense against pathogens*	Chen, Wei, et al. "Genome-wide association analyses provide genetic and biochemical insights into natural variation in rice metabolism." Nature genetics 46.7 (2014): 714-721.
Solyc12g010030.2	2.80	leucine aminopeptidase 2, chloroplastic isoform X1	LAP2	Involved in the processing and regular turnover of intracellular proteins; Heat shock protein	*Impaired turnover of intracellular proteins; Impaired response to heat-induced damaged*	Waditee-Sirisattha, Rungaroon, et al. "The Arabidopsis aminopeptidase LAP2 regulates plant growth, leaf longevity and stress response." New Phytologist 191.4 (2011): 958-969.
Solyc06g009190.3	2.60	pectinesterase	PME1	Acts in the modification of cell walls via demethylesterification of cell wall pectin; Acts as negative regulators of genes involved in salt stress response	*Impaired cell wall modification; Impaired response to salt stress*	Creighton, Maria T., Anna Kolton, Amr RA Kataya, Jodi Maple-Grødem, Irina O. Averkina, Behzad Heidari, and Cathrine Lillo. "Methylation of protein phosphatase 2A—Influence of regulators and environmental stress factors." Plant, cell & environment 40, no. 10 (2017): 2347-2358.
Solyc06g083130.3	2.55	dCTP pyrophosphatase 1	DCTPP1	Hydrolyzes dNTPs to the corresponding nucleoside monophosphates; Protects DNA/RNA against the incorporation of these genotoxic nucleotide analogs through their catabolism	*Impaired DNA/RNA protection against genotoxic nucleotide analogs*	Cheng, Chia-Yi, et al. "Araport11: a complete reannotation of the Arabidopsis thaliana reference genome." The Plant Journal 89.4 (2017): 789-804.
Solyc01g091170.3	2.54	Arginase 2, chloroplastic/mitochondrial	ARGAH2	Utilized in the urea cycle; Precursor for the synthesis of both polyamines and proline; Catalyzes the formation of putrescine from agmatine	*Impaired jasmonate-related functions; Impaired urea waste management; Impaired freezing tolerance*	Dombrecht, Bruno, et al. "MYC2 differentially modulates diverse jasmonate-dependent functions in Arabidopsis." The Plant Cell 19.7 (2007): 2225-2245.
Solyc06g083470.3	2.32	tropinone reductase homolog At5g06060	At5g06060	Has oxidoreductase activity	*Decreased oxidoreductase activity*	Ciftci-Yilmaz, Sultan, Mustafa R. Morsy, Luhua Song, Alicia Coutu, Beth A. Krizek, Michael W. Lewis, Daniel Warren, John Cushman, Erin L. Connolly, and Ron Mittler. "The EAR-motif of the Cys2/His2-type zinc finger protein Zat7 plays a key role in the defense response of Arabidopsis to salinity stress." Journal of Biological Chemistry 282, no. 12 (2007): 9260-9268.
Solyc06g083480.3	2.32	tropinone reductase homolog At5g06060	At5g06060	Has oxidoreductase activity	*Decreased oxidoreductase activity*	Ciftci-Yilmaz, Sultan, Mustafa R. Morsy, Luhua Song, Alicia Coutu, Beth A. Krizek, Michael W. Lewis, Daniel Warren, John Cushman, Erin L. Connolly, and Ron Mittler. "The EAR-motif of the Cys2/His2-type zinc finger protein Zat7 plays a key role in the defense response of Arabidopsis to salinity stress." Journal of Biological Chemistry 282, no. 12 (2007): 9260-9268.

TomatoID	DE in LsoB	NCBI Protein Name	Gene ID	Uniprot Description	Putative Consequences for Infection	Citation
Solyc01g005300.3	2.25	adagio protein 3	ADO3	Component of an E3 ubiquitin ligase complex that plays a central role in blue light-dependent circadian cycles	*Impaired E3 ubiquitination; Accumulation of misfolded proteins*	Fornara, Fabio, Kishore CS Panigrahi, Lionel Gissot, Nicolas Sauerbrunn, Mark Rühl, José A. Jarillo, and George Coupland. "Arabidopsis DOF transcription factors act redundantly to reduce CONSTANS expression and are essential for a photoperiodic flowering response." Developmental cell 17, no. 1 (2009): 75-86.
Solyc11g066390.2	2.23	superoxide dismutase [Cu-Zn], chloroplastic	CSD2	Processes reactive oxygen species which are normally produced within the cells; Mediates tolerance to stress, including photo-oxidative stress	*Impaired photo-oxidative stress response*	Abarca, Dolores, Marta Roldán, Mercedes Martín, and Bartolomé Sabater. "Arabidopsis thaliana ecotype Cvi shows an increased tolerance to photo-oxidative stress and contains a new chloroplastic copper/zinc superoxide dismutase isoenzyme." Journal of experimental botany 52, no. 360 (2001): 1417-1425.
Solyc07g008410.3	2.01	protein DETOXIFICATION 29-like	DTX29	Xenobiotic transmembrane transporter activity	*Impaired detoxification*	Hanada, Kousuke, et al. "Functional compensation of primary and secondary metabolites by duplicate genes in Arabidopsis thaliana." Molecular biology and evolution 28.1 (2011): 377-382.
Solyc04g063210.3	1.88	probable caffeoyl-CoA O-methyltransferase At4g26220 isoform X2	AT4G26220	Plays a role in the synthesis of feruloylated polysaccharides; Involved in the reinforcement of the plant cell wall; Involved in response to wounding and/or pathogen challenge	*Impaired reinforcement of plant cell walls; Impaired response to wounding and/or pathogen challenge*	Meyermans, Hugo, et al. "Modifications in lignin and accumulation of phenolic glucosides in poplar xylem upon down-regulation of caffeoyl-coenzyme A O-methyltransferase, an enzyme involved in lignin biosynthesis." Journal of Biological Chemistry 275.47 (2000): 36899-36909.
Solyc04g081890.1	1.75	E3 ubiquitin-protein ligase ATL23	ATL23	E3 ubiquitin-protein ligase able to catalyze polyubiquitination with ubiquitin-conjugating enzymes	*Impaired E3 ubiquitination; Accumulation of misfolded proteins*	Kraft, E., Stone, S.L., Ma, L., Su, N., Gao, Y., Lau, O.S., Deng, X.W. and Callis, J., 2005. Genome analysis and functional characterization of the E2 and RING-type E3 ligase ubiquitination enzymes of Arabidopsis. Plant physiology, 139(4), pp.1597-1611.
Solyc01g096450.3	1.71	aspartyl protease AED3	AED3	Involved in regulation of programmed cell death; Involved in systemic acquired resistance	*Impaired programmed cell death; Impaired systemic acquired resistance*	Dinneny, Jose R., Terri A. Long, Jean Y. Wang, Jee W. Jung, Daniel Mace, Solomon Pointer, Christa Barron, Siobhan M. Brady, John Schiefelbein, and Philip N. Benfey. "Cell identity mediates the response of Arabidopsis roots to abiotic stress." Science 320, no. 5878 (2008): 942-945.
Solyc04g078520.3	1.64	protein RICE SALT SENSITIVE 3-like	RSS3	Represses jasmonate-induced genes; Involved in transcriptional regulation in the root tip; Regulates root cell elongation during salt stress	*Impaired repression of jasmonate-induced genes; Inceased jasmonate-related defense; Impaired root cell elongation*	Toda, Yosuke, Maiko Tanaka, Daisuke Ogawa, Kyo Kurata, Ken-ichi Kurotani, Yoshiki Habu, Tsuyu Ando et al. "RICE SALT SENSITIVE3 forms a ternary complex with JAZ and class-C bHLH factors and regulates jasmonate-induced gene expression and root cell elongation." The Plant Cell 25, no. 5 (2013): 1709-1725.
Solyc12g005020.2	1.60	NEP1-interacting protein-like 1 isoform X1	ATL27	Involved in the early steps of the plant defense signaling pathway	*Impaired plant defense*	Libault, Marc, Jinrong Wan, Tomasz Czechowski, Michael Udvardi, and Gary Stacey. "Identification of 118 Arabidopsis transcription factor and 30 ubiquitin-ligase genes responding to chitin, a plant-defense elicitor." Molecular plant-microbe interactions 20, no. 8 (2007): 900-911.

TomatoID	DE in LsoB	NCBI Protein Name	Gene ID	Uniprot Description	Putative Consequences for Infection	Citation
Solyc03g117560.3	1.59	lamin-like protein	At5g15350	Has electron transfer activity; Involved in early response to salicylic acid	*Impaired salicylic acid-induced defense*	Krinke, Ondrej, Eric Ruelland, Olga Valentová, Chantal Vergnolle, Jean-Pierre Renou, Ludivine Taconnat, Matyás Flemr, Lenka Burketová, and Alain Zachowski. "Phosphatidylinositol 4-kinase activation is an early response to salicylic acid in Arabidopsis suspension cells." Plant Physiology 144, no. 3 (2007): 1347-1359.
Solyc08g007240.3	1.53	nudix hydrolase 8 isoform X2	NUDT8	Mediates the hydrolysis of some nucleoside diphosphate derivatives; Positive regulator of defense response through salicylic acid signaling	*Impaired salicylic acid-induced defense*	Fonseca, Jose Pedro, and Xinnian Dong. "Functional characterization of a Nudix hydrolase AtNUDX8 upon pathogen attack indicates a positive role in plant immune responses." PloS one 9, no. 12 (2014): e114119.
Solyc06g065820.3	1.51	ethylene response factor H.1	ERF1A	Acts as a transcriptional activator; Involved in the regulation of gene expression by stress factors and by components of stress signal transduction pathways	*Impaired stress response*	Fujimoto, Susan Y., Masaru Ohta, Akemi Usui, Hideaki Shinshi, and Masaru Ohme-Takagi. "Arabidopsis ethylene-responsive element binding factors act as transcriptional activators or repressors of GCC box–mediated gene expression." The Plant Cell 12, no. 3 (2000): 393-404.
Solyc08g042100.3	1.50	U-box domain-containing protein 3-like	PUB3	Functions as an E3 ubiquitin ligase	*Impaired E3 ubiquitination; Accumulation of misfolded proteins*	Benschop, Joris J., Shabaz Mohammed, Martina O'Flaherty, Albert JR Heck, Monique Slijper, and Frank LH Menke. "Quantitative phosphoproteomics of early elicitor signaling in Arabidopsis." Molecular & Cellular Proteomics 6, no. 7 (2007): 1198-1214.
Solyc04g008850.1	1.42	protein ASPARTIC PROTEASE IN GUARD CELL 2-like	ASPG2	Aspartic protease involved in drought avoidance through abscisic acid signaling	*Impaired drought response; Impaired abscisic acid signaling*	Yao, Xuan, Wei Xiong, Tiantian Ye, and Yan Wu. "Overexpression of the aspartic protease ASPG1 gene confers drought avoidance in Arabidopsis." Journal of experimental botany 63, no. 7 (2012): 2579-2593.
Solyc02g090960.1	1.36	protein RALF-like 34	RALFL34	Cell signaling peptide that regulates plant stress, growth, and development	*Impaired response to plant stress, growth, and development*	Murphy, Evan, Lam Dai Vu, Lisa Van den Broeck, Zhefeng Lin, Priya Ramakrishna, Brigitte Van De Cotte, Allison Gaudinier et al. "RALFL34 regulates formative cell divisions in Arabidopsis pericycle during lateral root initiation." Journal of Experimental Botany 67, no. 16 (2016): 4863-4875.
Solyc06g062960.2	1.35	protein RALF-like 34	RALFL34	Cell signaling peptide that may regulate plant stress, growth, and development	*Impaired response to plant stress, growth, and development*	Murphy, Evan, Lam Dai Vu, Lisa Van den Broeck, Zhefeng Lin, Priya Ramakrishna, Brigitte Van De Cotte, Allison Gaudinier et al. "RALFL34 regulates formative cell divisions in Arabidopsis pericycle during lateral root initiation." Journal of Experimental Botany 67, no. 16 (2016): 4863-4875.
Solyc09g009010.3	1.35	glucomannan 4-beta-mannosyltransferase 9-like isoform X2	CSLA9	Required for lateral root development; Involved in cell wall organization; Involved in response to bacteria	*Impaired lateral root development; Impaired plant cell wall organization; Impaired defense response to bacteria*	Liepman, Aaron H., C. Joseph Nairn, William GT Willats, Iben Sørensen, Alison W. Roberts, and Kenneth Keegstra. "Functional genomic analysis supports conservation of function among cellulose synthase-like a gene family members and suggests diverse roles of mannans in plants." Plant Physiology 143, no. 4 (2007): 1881-1893.

TomatoID	DE in LsoB	NCBI Protein Name	Gene ID	Uniprot Description	Putative Consequences for Infection	Citation
Solyc09g008990.3	1.35	glucomannan 4-beta-mannosyltransferase 9-like isoform X2	CSLA9	Required for lateral root development; Involved in cell wall organization; Involved in response to bacteria	*Impaired lateral root development; Impaired plant cell wall organization; Impaired defense response to bacteria*	Liepman, Aaron H., C. Joseph Nairn, William GT Willats, Iben Sørensen, Alison W. Roberts, and Kenneth Keegstra. "Functional genomic analysis supports conservation of function among cellulose synthase-like a gene family members and suggests diverse roles of mannans in plants." Plant Physiology 143, no. 4 (2007): 1881-1893.
Solyc12g089330.2	1.30	synaptotagmin-1	SYT1	Maintains plasma membrane integrity during freezing and osmotic stresses; Functions in membrane resealing during calcium-dependent freezing tolerance; Regulates endocytosis and endosome recycling at the plasma membrane and cell-to-cell trafficking	*Impaired freezing response; Impaired cell-to-cell trafficking*	Yamazaki, Tomokazu, Naoki Takata, Matsuo Uemura, and Yukio Kawamura. "Arabidopsis synaptotagmin SYT1, a type I signal-anchor protein, requires tandem C2 domains for delivery to the plasma membrane." Journal of Biological Chemistry 285, no. 30 (2010): 23165-23176.
Solyc08g082250.3	1.29	endo-beta-1,4-D-glucanase precursor	At3g13560	Involved in carbohydrate metabolism; Involved in defense response	*Impaired carbohydrate metabolism; Impaired defense response*	Jakoby, Marc J., Doris Falkenhan, Michael T. Mader, Ginger Brininstool, Elisabeth Wischnitzki, Nicole Platz, Andrew Hudson, Martin Hulskamp, John Larkin, and Arp Schnittger. "Transcriptional profiling of mature Arabidopsis trichomes reveals that NOECK encodes the MIXTA-like transcriptional regulator MYB106." Plant Physiology 148, no. 3 (2008): 1583-1602.
Solyc02g065170.3	1.27	L-ascorbate oxidase homolog	AAO	Represses responses to high salinity and oxidative stress conditions such as vegetative growth and seed production reductions	*Impaired regulation of stress responses; Impaired growth/development under stress*	Yamamoto, Atsuko, Md Nazmul H. Bhuiyan, Rungaroon Waditee, Yoshito Tanaka, Muneharu Esaka, Kazuko Oba, André T. Jagendorf, and Teruhiro Takabe. "Suppressed expression of the apoplastic ascorbate oxidase gene increases salt tolerance in tobacco and Arabidopsis plants." Journal of Experimental Botany 56, no. 417 (2005): 1785-1796.
Solyc02g087190.1	1.22	peroxidase 63	PER63	Involved in oxidation of toxic reductants, lignin metabolism, suberization, auxin catabolism; Involved in response to environmental stresses	*Impaired plant defense; Impaired lignin metabolism; Impaired auxin catabolism*	Valério, Luisa, Mireille De Meyer, Claude Penel, and Christophe Dunand. "Expression analysis of the Arabidopsis peroxidase multigenic family." Phytochemistry 65, no. 10 (2004): 1331-1342.
Solyc09g014490.3	1.22	endochitinase A	At2g43590	Involved in plant cell wall macromolecule catabolism and chitin catabolism; Involved in defense response	*Impaired plant cell wall development; Impaired defense response*	Krysan, Patrick J., Peter J. Jester, Jennifer R. Gottwald, and Michael R. Sussman. "An Arabidopsis mitogen-activated protein kinase kinase kinase gene family encodes essential positive regulators of cytokinesis." The Plant Cell 14, no. 5 (2002): 1109-1120.
Solyc12g009110.2	1.20	acetylserotonin O-methyltransferase	ASMT	Methyltransferase which catalyzes the production of melatonin; Involved in response to light stress	*Impaired development under light stress*	Shi, Haitao, Yunxie Wei, and Chaozu He. "Melatonin-induced CBF/DREB1s are essential for diurnal change of disease resistance and CCA1 expression in Arabidopsis." Plant Physiology and Biochemistry 100 (2016): 150-155.

TomatoID	DE in LsoB	NCBI Protein Name	Gene ID	Uniprot Description	Putative Consequences for Infection	Citation
Solyc11g051170.2	1.02	ATP synthase CF1 alpha subunit	atpA	Produces ATP from ADP in the presence of a proton gradient across the membrane; Necessary for defense response to bacteria and cold	*Impaired defense against pathogens; Impaired cold tolerance*	Kubis, Sybille, Ramesh Patel, Jonathan Combe, Jocelyn Beédard, Sabina Kovacheva, Kathryn Lilley, Alexander Biehl et al. "Functional specialization amongst the Arabidopsis Toc159 family of chloroplast protein import receptors." The Plant Cell 16, no. 8 (2004): 2059-2077.
Solyc05g007900.2	-1.01	E3 ubiquitin-protein ligase MPSR1	MPSR1	E3 ubiquitin-protein ligase involved in protein quality control under proteotoxic stress	Increased E3 ubiquitination during proteotoxic stress	Kim, Jong Hum, Seok Keun Cho, Tae Rin Oh, Moon Young Ryu, Seong Wook Yang, and Woo Taek Kim. "MPSR1 is a cytoplasmic PQC E3 ligase for eliminating emergent misfolded proteins in Arabidopsis thaliana." Proceedings of the National Academy of Sciences 114, no. 46 (2017): E10009-E10017.
Solyc05g052050.1	-1.10	DNA-binding protein Pti4	Pti4	Transcription factor involved in defense	Promoted defense	Gu, Yong-Qiang, Mary C. Wildermuth, Suma Chakravarthy, Ying-Tsu Loh, Caimei Yang, Xiaohua He, Yu Han, and Gregory B. Martin. "Tomato transcription factors Pti4, Pti5, and Pti6 activate defense responses when expressed in Arabidopsis." The Plant Cell 14, no. 4 (2002): 817-831.
Solyc02g084950.3	-1.11	jasmonate O-methyltransferase isoform X2	JMT	Catalyzes the methylation of jasmonate into methyljasmonate, a plant volatile that acts as an important cellular regulator mediating diverse developmental processes and defense responses	Promoted methyljasmonate-regulated signaling; Promoted defenese	Seo, Hak Soo, Jong Tae Song, Jong-Joo Cheong, Yong-Hwan Lee, Yin-Won Lee, Ingyu Hwang, Jong Seob Lee, and Yang Do Choi. "Jasmonic acid carboxyl methyltransferase: a key enzyme for jasmonate-regulated plant responses." Proceedings of the National Academy of Sciences 98, no. 8 (2001): 4788-4793.
Solyc11g066060.2	-1.14	heat shock cognate 70 kDa protein 2-like	HSP70-2	Facilitate folding of de novo synthesized proteins, assist translocation of precursor proteins into organelles, and are responsible for degradation of damaged protein under stress conditions	Promoted folding of de novo synthesized proteins; Promoted protein transport; Promoted degradation of damanged proteins under stress	Hilson, Pierre, Joke Allemeersch, Thomas Altmann, Sébastien Aubourg, Alexandra Avon, Jim Beynon, Rishikesh P. Bhalerao et al. "Versatile gene-specific sequence tags for Arabidopsis functional genomics: transcript profiling and reverse genetics applications." Genome research 14, no. 10b (2004): 2176-2189.
Solyc01g111640.3	-1.14	SKP1-like protein 1A	SKP1A	Involved in ubiquitination; Required for vegetative and floral organ development and for male gametogenesis; Involved in auxin signaling pathway; Regulates responses to jasmonates; Involved in light-signaling and the circadian clock	Promoted protein ubiquitination; Promoted floral development and male gametogenesis; Promoted auxin signaling; Promoted circadian clock; Promoted embryogenesis	Zhao, Dazhong, Weimin Ni, Baomin Feng, Tianfu Han, Megan G. Petrasek, and Hong Ma. "Members of the Arabidopsis-SKP1-like gene family exhibit a variety of expression patterns and may play diverse roles in Arabidopsis." Plant physiology 133, no. 1 (2003): 203-217.
Solyc12g009560.2	-1.18	EIN3-binding F-box protein 1	EBF1	Component of ubiquitin ligase complexes, which mediate the ubiquitination and subsequent proteasomal degradation of target proteins; Regulates ethylene signaling cascade	Promoted protein ubiquitination	Gagne, Jennifer M., Jan Smalle, Derek J. Gingerich, Joseph M. Walker, Sang-Dong Yoo, Shuichi Yanagisawa, and Richard D. Vierstra. "Arabidopsis EIN3-binding F-box 1 and 2 form ubiquitin-protein ligases that repress ethylene action and promote growth by directing EIN3 degradation." Proceedings of the National Academy of Sciences 101, no. 17 (2004): 6803-6808.

TomatoID	DE in LsoB	NCBI Protein Name	Gene ID	Uniprot Description	Putative Consequences for Infection	Citation
Solyc10g051230.1	-1.19	receptor-like protein Cf-9	CF-9	Involved in plant defense against fungal pathogens	Promoted defense against fungi	Jones, David A., Colwyn M. Thomas, Kim E. Hammond-Kosack, Peter J. Balint-Kurti, and J. D. Jones. "Isolation of the tomato Cf-9 gene for resistance to Cladosporium fulvum by transposon tagging." Science 266, no. 5186 (1994): 789-793.
Solyc01g105620.3	-1.30	RING-H2 finger protein ATL54-like	LOC109001200	This protein is involved in the pathway protein ubiquitination, which is part of Protein modification	Promoted protein ubiquitination	Martínez-García, Pedro J., Marc W. Crepeau, Daniela Puiu, Daniel Gonzalez-Ibeas, Jeanne Whalen, Kristian A. Stevens, Robin Paul et al. "The walnut (Juglans regia) genome sequence reveals diversity in genes coding for the biosynthesis of non-structural polyphenols." The Plant Journal 87, no. 5 (2016): 507-532.
Solyc12g096610.2	-1.32	poly(ADP-ribose) glycohydrolase 1-like isoform X3	PARG1	Involved in establishing period length of the circadian oscillator; Involved in defense against fungus; Involved in response to osmotic and water stress	Promoted circadian clock; Promoted defense against fungi; Promoted drought tolerance; Increased defense against osmotic stress	Panda, Satchidananda, Guy G. Poirier, and Steve A. Kay. "tej defines a role for poly (ADP-ribosyl) ation in establishing period length of the Arabidopsis circadian oscillator." Developmental cell 3, no. 1 (2002): 51-61.
Solyc11g008530.2	-1.33	endoribonuclease Dicer 2d isoform X1	AT3G03300	Plays an essential role in transitive silencing of transgenes by processing secondary siRNAs; Plays a role in antiviral RNA silencing	Promoted post-transcriptional gene silencing; Promoted defense against viral pathogens	"DICER-LIKE2 plays a primary role in transitive silencing of transgenes in Arabidopsis."
Solyc12g096600.2	-1.34	poly(ADP-ribose) glycohydrolase 1-like isoform X4	PARG1	Involved in establishing period length of the circadian oscillator; Involved in deefense against fungus; Involved in response to osmotic and water stress	Promoted circadian clock; Promoted defense against fungi; Promoted drought tolerance; Increased defense against osmotic stress	Panda, Satchidananda, Guy G. Poirier, and Steve A. Kay. "tej defines a role for poly (ADP-ribosyl) ation in establishing period length of the Arabidopsis circadian oscillator." Developmental cell 3, no. 1 (2002): 51-61.
Solyc11g010380.2	-1.41	protein DETOXIFICATION 27	DTX27	Has xenobiotic transmembrane transporter activity	Promoted xenobiotic transport	Ascencio-Ibánez, José Trinidad, Rosangela Sozzani, Tae-Jin Lee, Tzu-Ming Chu, Russell D. Wolfinger, Rino Cella, and Linda Hanley-Bowdoin. "Global analysis of Arabidopsis gene expression uncovers a complex array of changes impacting pathogen response and cell cycle during geminivirus infection." Plant physiology 148, no. 1 (2008): 436-454.
Solyc08g067610.3	-1.45	pleiotropic drug resistance protein 1-like	PDR1	Excretes secondary metabolites such as terpenes; Involved in both constitutive and jasmonic acid-dependent induced defense	Increased secondary metabolite excretion; Promoted jasmonic-acid depended induced defense; Promoted constitutive defense	Jasiński, Michal, Yvan Stukkens, Hervé Degand, Bénédicte Purnelle, Jacqueline Marchand-Brynaert, and Marc Boutry. "A plant plasma membrane ATP binding cassette–type transporter is involved in antifungal terpenoid secretion." The Plant Cell 13, no. 5 (2001): 1095-1107.
Solyc04g071890.3	-1.47	peroxidase 12 precursor	PER12	Involved in removal of H2O2, oxidation of toxic reductants, biosynthesis and degradation of lignin, suberization, auxin catabolism, and response to environmental stressors	Increased removal of H2O2, oxidation of toxic reductants, biosynthesis and degradation of lignin, suberization, auxin catabolism, and response to environmental stressors	Paynel, Florence, et al. "Temporal regulation of cell-wall pectin methylesterase and peroxidase isoforms in cadmium-treated flax hypocotyl." Annals of botany 104.7 (2009): 1363-1372.

TomatoID	DE in LsoB	NCBI Protein Name	Gene ID	Uniprot Description	Putative Consequences for Infection	Citation
Solyc08g006470.3	-1.49	cytoplasmic 60S subunit biogenesis factor REI1 homolog 1	REIL1	Involved in the dissociation and recycling of other late pre-60S factors before newly synthesized large ribosomal subunits enter translation; Required for leaf growth under cold temperature conditions	Promoted growth under cold stress	Schmidt, Stefanie, Frederik Dethloff, Olga Beine-Golovchuk, and Joachim Kopka. "The REIL1 and REIL2 proteins of Arabidopsis thaliana are required for leaf growth in the cold." Plant physiology 163, no. 4 (2013): 1623-1639.
Solyc03g025450.3	-1.55	U-box domain-containing protein 32 isoform X2	PUB32	Functions as an E3 ubiquitin ligase	Promoted E3 ubiquitination	Benschop, Joris J., Shabaz Mohammed, Martina O'Flaherty, Albert JR Heck, Monique Slijper, and Frank LH Menke. "Quantitative phosphoproteomics of early elicitor signaling in Arabidopsis." Molecular & Cellular Proteomics 6, no. 7 (2007): 1198-1214.
Solyc11g066100.2	-1.56	heat shock cognate 70 kDa protein	HSP70-1	Facilitate folding of de novo synthesized proteins, assist translocation of precursor proteins into organelles, and are responsible for degradation of damaged protein under stress conditions	Promoted folding of de novo synthesized proteins; Promoted protein transport; Promoted degradation of damanged proteins under stress	Sung, Dong Yul, and Charles L. Guy. "Physiological and molecular assessment of altered expression of Hsc70-1 in Arabidopsis. Evidence for pleiotropic consequences." Plant Physiology 132, no. 2 (2003): 979-987.
Solyc09g089930.2	-1.57	ethylene responsive element binding protein	EREBP1	Involved in defense response; Involved in ethylene-activated signaling pathway	Increased defense response; Increased ethylene-responsive signaling	Horvath, Diana M., Dorothy J. Huang, and Nam-Hai Chua. "Four classes of salicylate-induced tobacco genes." Molecular plant-microbe interactions 11.9 (1998): 895-905.
Solyc04g040130.1	-1.86	delta(12)-fatty-acid desaturase FAD2-like	FAD2	Required for desaturation of fatty acids present in extraplastidial membranes, including mitochondria; Required for salt tolerance during seed germination and early seedling growth	Promoted salt tolerance during seed germination and early growth	Okuley, John, Jonathan Lightner, Kenneth Feldmann, Narendra Yadav, Ellen Lark, and John Browse. "Arabidopsis FAD2 gene encodes the enzyme that is essential for polyunsaturated lipid synthesis." The Plant Cell 6, no. 1 (1994): 147-158.
Solyc05g053610.2	-2.22	pleiotropic drug resistance protein 1-like	PDR1	Excretes secondary metabolites such as terpenes; Involved in both constitutive and jasmonic acid-dependent induced defense	Increased secondary metabolite excretion; Promoted jasmonic-acid depended induced defense; Promoted constitutive defense	Jasiński, Michal, Yvan Stukkens, Hervé Degand, Bénédicte Purnelle, Jacqueline Marchand-Brynaert, and Marc Boutry. "A plant plasma membrane ATP binding cassette–type transporter is involved in antifungal terpenoid secretion." The Plant Cell 13, no. 5 (2001): 1095-1107.
Solyc09g007520.3	-2.27	peroxidase 21	PER21	Involved in removal of H2O2, oxidation of toxic reductants, biosynthesis and degradation of lignin, suberization, auxin catabolism, and response to environmental stressors	Increased removal of H2O2, oxidation of toxic reductants, biosynthesis and degradation of lignin, suberization, auxin catabolism, and response to environmental stressors	Mosher, Rebecca A., Wendy E. Durrant, Dong Wang, Junqi Song, and Xinnian Dong. "A comprehensive structure–function analysis of Arabidopsis SNI1 defines essential regions and transcriptional repressor activity." The Plant Cell 18, no. 7 (2006): 1750-1765.
Solyc09g008760.1	-2.39	probable cytochrome P450 556A1	cyp516A1	Involved in xenobiotic metabolic process	Promoted xenobiotic metabolism	Eichinger, L., J. A. Pachebat, G. Glöckner, M-A. Rajandream, R. Sucgang, M. Berriman, J. Song et al. "The genome of the social amoeba Dictyostelium discoideum." Nature 435, no. 7038 (2005): 43-57.

TomatoID	DE in LsoB	NCBI Protein Name	Gene ID	Uniprot Description	Putative Consequences for Infection	Citation
Solyc03g095650.3	-2.50	MLO-like protein 5	MLO5	Involved in modulation of pathogen defense and leaf cell death	Promoted defense against pathogens; Promoted leaf programmed cell death	Moriyama, Etsuko N., Pooja K. Strope, Stephen O. Opiyo, Zhongying Chen, and Alan M. Jones. "Mining the Arabidopsis thaliana genome for highly-divergent seven transmembrane receptors." Genome biology 7, no. 10 (2006): 1-9.
Solyc03g033840.3	-2.57	AAA-ATPase At3g50940-like	At3g50940	Involved in lignin biosynthesis and response to salt stress	Promoted lignin biosynthesis; Promoted resposne to salt stress	van de Mortel, Judith E., Laia Almar Villanueva, Henk Schat, Jeroen Kwekkeboom, Sean Coughlan, Perry D. Moerland, Emiel Ver Loren van Themaat, Maarten Koornneef, and Mark GM Aarts. "Large expression differences in genes for iron and zinc homeostasis, stress response, and lignin biosynthesis distinguish roots of Arabidopsis thaliana and the related metal hyperaccumulator Thlaspi caerulescens." Plant physiology 142, no. 3 (2006): 1127-1147.
Solyc08g068870.3	-2.63	aspartate protease family protein precursor	APF1	Aspartyl protease; Necessary for fungal resistance	Increased fungal resistance	Li, Yurong, Mehdi Kabbage, Wende Liu, and Martin B. Dickman. "Aspartyl protease-mediated cleavage of BAG6 is necessary for autophagy and fungal resistance in plants." The Plant Cell 28, no. 1 (2016): 233-247.
Solyc12g100250.2	-2.78	delta(12)-fatty-acid desaturase FAD2 isoform X1	FAD2	Required for desaturation of fatty acids present in extraplastidial membranes, including mitochondria; Required for salt tolerance during seed germination and early seedling growth	Promoted salt tolerance during seed germination and early growth	Okuley, John, Jonathan Lightner, Kenneth Feldmann, Narendra Yadav, Ellen Lark, and John Browse. "Arabidopsis FAD2 gene encodes the enzyme that is essential for polyunsaturated lipid synthesis." The Plant Cell 6, no. 1 (1994): 147-158.
Solyc12g100240.1	-2.78	delta(12)-fatty-acid desaturase FAD2-like	FAD2	Required for desaturation of fatty acids present in extraplastidial membranes, including mitochondria; Required for salt tolerance during seed germination and early seedling growth	Promoted salt tolerance during seed germination and early growth	Okuley, John, Jonathan Lightner, Kenneth Feldmann, Narendra Yadav, Ellen Lark, and John Browse. "Arabidopsis FAD2 gene encodes the enzyme that is essential for polyunsaturated lipid synthesis." The Plant Cell 6, no. 1 (1994): 147-158.
Solyc12g100260.1	-3.19	delta(12)-fatty-acid desaturase FAD2-like	FAD2	Required for desaturation of fatty acids present in extraplastidial membranes, including mitochondria; Required for salt tolerance during seed germination and early seedling growth	Promoted salt tolerance during seed germination and early growth	Okuley, John, Jonathan Lightner, Kenneth Feldmann, Narendra Yadav, Ellen Lark, and John Browse. "Arabidopsis FAD2 gene encodes the enzyme that is essential for polyunsaturated lipid synthesis." The Plant Cell 6, no. 1 (1994): 147-158.
Solyc09g091000.3	-4.16	pathogenesis-related protein STH-2	STH-2	Regulates protein serein/threonine phosphate activity; Involved in abscisic acid-activated signaling pathway; Involved in defense response to biotic stimulus	Promoted absisic acid signaling; Promoted defense against biotic stimuli	Matton, Daniel P., Gary Prescott, Charles Bertrand, Anne Camirand, and Normand Brisson. "Identification of cis-acting elements involved in the regulation of the pathogenesis-related gene STH-2 in potato." Plant molecular biology 22, no. 2 (1993): 279-291.
