# Supplementary material for: Effects of ‘Candidatus Liberibacter solanacearum’ haplotypes A and B on tomato gene expression and geotropism
Source: BMC Plant Biol. 2022 Mar 30;22:156. doi: 10.1186/s12870-022-03505-z (PMC8966271; doi:10.1186/s12870-022-03505-z)
Supplement: Supplementary file 14 — Additional file 14: Table S14. The 48 tomato plant DEGs associated with plant signaling and transport under infection by different Lso haplotypes. DEGs were sorted by differential expression (DE) values comparing uninfected to infected samples (p-value < 0.05). NCBI Blast searches were used to identify Gene IDs and protein products in tomatoes as well as their homologs in other species. Specifically, the expression changes in 29 of these genes (60%, in italics) would have resulted in impaired signaling transduction or transport in LsoB-infected plants relative to LsoA-infected plants. These DEGs were predominately related to ion transport, abscisic signaling and transport, and auxin-responsive signaling. [file 12870_2022_3505_MOESM14_ESM.pdf]

TomatoID	DE in LsoB	NCBI Protein Name	Gene ID	Uniprot Description	Putative Consequences for Infection	Citation
Solyc02g089350.3	2.26	protein GAST1 precursor	GAST1	Involved in root-specific abscisic acid-signaling regulation	*Impaired abscisic acid-signaling regulation*	Shi, Lifang, Robert T. Gast, Manjula Gopalraj, and Neil E. Olszewski. "Characterization of a shoot-specific, GA3-and ABA-regulated gene from tomato." The Plant Journal 2, no. 2 (1992): 153-159.
Solyc03g123410.1	2.24	auxin-binding protein ABP19a	ABP19A	Receptor for the plant growth-promoting hormone auxin	*Impaired auxin signaling*	Ohmiya, Akemi, Yoshiyuki Tanaka, Koh-ichi Kadowaki, and Tateki Hayashi. "Cloning of genes encoding auxin-binding proteins (ABP19/20) from peach: significant peptide sequence similarity with germin-like proteins." Plant and cell physiology 39, no. 5 (1998): 492-499.
Solyc01g096220.3	1.98	ras-related protein RABA3	RABA3	Involved in intracellular vesicle trafficking and protein transport	*Impaired intracellular vesicle trafficking*	Lunn, Daniel, Sanyasi R. Gaddipati, Gregory A. Tucker, and Grantley W. Lycett. "Null mutants of individual RABA genes impact the proportion of different cell wall components in stem tissue of Arabidopsis thaliana." PLoS One 8, no. 10 (2013): e75724.
Solyc06g051940.3	1.85	protein phosphatase 2C	PP2CA	Major negative regulator of abscisic acid responses during seed germination and cold acclimation; Prevents stomata closure	*Impaired abscisic acid signaling responses; Promoted stomatal opening*	Chérel, Isabelle, et al. "Physical and functional interaction of the Arabidopsis K+ channel AKT2 and phosphatase AtPP2CA." The Plant Cell 14.5 (2002): 1133-1146.
Solyc02g066970.1	1.72	transcription factor PAR1	PAR1	Negative regulator of a variety of shade avoidance syndrome responses, including seedling elongation and photosynthetic pigment accumulation; Transcriptional repressor of two auxin-responsive genes	*Impaired shade avoidance; Impaired repression of auxin signaling*	Roig-Villanova, Irma, Jordi Bou-Torrent, Anahit Galstyan, Lorenzo Carretero-Paulet, Sergi Portolés, Manuel Rodríguez-Concepción, and Jaime F. Martínez-García. "Interaction of shade avoidance and auxin responses: a role for two novel atypical bHLH proteins." The EMBO journal 26, no. 22 (2007): 4756-4767.
Solyc01g098740.3	1.71	probable serine/threonine-protein kinase PBL7	PBL7	Serine/threonine-protein kinase involved in the positive regulation of brassinosteroid signaling and plant growth	*Impaired brassinosteroid signaling; Impaired growth*	Kim, Tae-Wuk, Shenheng Guan, Alma L. Burlingame, and Zhi-Yong Wang. "The CDG1 kinase mediates brassinosteroid signal transduction from BRI1 receptor kinase to BSU1 phosphatase and GSK3-like kinase BIN2." Molecular cell 43, no. 4 (2011): 561-571.
Solyc03g113060.3	1.61	ABC transporter A family member 7 isoform X1	ABCA7	Involved in lipid transport	*Impaired lipid transport*	Badri, D.V., Loyola-Vargas, V.M., Broeckling, C.D., De-la-Peña, C., Jasinski, M., Santelia, D., Martinoia, E., Sumner, L.W., Banta, L.M., Stermitz, F. and Vivanco, J.M., 2008. Altered profile of secondary metabolites in the root exudates of Arabidopsis ATP-binding cassette transporter mutants. Plant Physiology, 146(2), pp.323-324.
Solyc12g044310.2	1.57	protein NRT1/ PTR FAMILY 1.2	NPF1.2	Low-affinity nitrate transporter involved in xylem-to-phloem transfer for redistributing nitrate into developing leaves	*Impaired nitrate transport; Impaired growth/development*	Hsu, Po-Kai, and Yi-Fang Tsay. "Two phloem nitrate transporters, NRT1. 11 and NRT1. 12, are important for redistributing xylem-borne nitrate to enhance plant growth." Plant Physiology 163, no. 2 (2013): 844-856.
Solyc04g008850.1	1.42	protein ASPARTIC PROTEASE IN GUARD CELL 2-like	ASPG2	Aspartic protease involved in drought avoidance through abscisic acid signaling	*Impaired drought response; Impaired abscisic acid signaling*	Yao, Xuan, Wei Xiong, Tiantian Ye, and Yan Wu. "Overexpression of the aspartic protease ASPG1 gene confers drought avoidance in Arabidopsis." Journal of experimental botany 63, no. 7 (2012): 2579-2593.

TomatoID	DE in LsoB	NCBI Protein Name	Gene ID	Uniprot Description	Putative Consequences for Infection	Citation
Solyc06g076570.3	1.41	class I small heat shock protein	HSP17.8	Cytosolic mediator for sorting and targeting of nascent chloroplast outer envelope membrane proteins to the chloroplast	*Impaired chloroplast transport*	Kim, Dae Heon, Zheng-Yi Xu, Yun Jeong Na, Yun-Joo Yoo, Junho Lee, Eun-Ju Sohn, and Inhwan Hwang. "Small heat shock protein Hsp17. 8 functions as an AKR2A cofactor in the targeting of chloroplast outer membrane proteins in Arabidopsis." Plant physiology 157, no. 1 (2011): 132-146.
Solyc09g097890.2	1.37	cytochrome b561 and DOMON domain-containing protein At3g25290	At3g25290	Catecholamine-responsive trans-membrane electron transporter	*Impaired transmembrane transport*	Verelst, Wim, and Han Asard. "Analysis of an Arabidopsis thaliana protein family, structurally related to cytochromes b 561 and potentially involved in catecholamine biochemistry in plants." Journal of plant physiology 161, no. 2 (2004): 175-181.
Solyc03g093410.3	1.35	hexose carrier protein HEX6	HEX6	Involved in uptake of hexoses; Symporter activity	*Impaired hexose uptake*	Weig, Alfons, Juliane Franz, Norbert Sauer, and Ewald Komor. "Isolation of a family of cDNA clones from Ricinus communis L. with close homology to the hexose carriers." Journal of Plant Physiology 143, no. 2 (1994): 178-183.
Solyc09g014610.3	1.34	S-type anion channel SLAH2-like	LOC107824438	Involved in transmembrane transport	*Impaired transmembrane transport*	Sierro, Nicolas, James ND Battey, Sonia Ouadi, Nicolas Bakaher, Lucien Bovet, Adrian Willig, Simon Goepfert, Manuel C. Peitsch, and Nikolai V. Ivanov. "The tobacco genome sequence and its comparison with those of tomato and potato." Nature communications 5, no. 1 (2014): 1-9.
Solyc03g113720.3	1.32	two-component response regulator ARR15	ARR15	Functions as response regulator involved in His-to-Asp phosphorelay signal transduction system; Negative regulator of the cytokinin signaling	*Impaired regulation of cytokinin signaling*	Kiba, Takatoshi, Hisami Yamada, and Takeshi Mizuno. "Characterization of the ARR15 and ARR16 response regulators with special reference to the cytokinin signaling pathway mediated by the AHK4 histidine kinase in roots of Arabidopsis thaliana." Plant and Cell Physiology 43, no. 9 (2002): 1059-1066.
Solyc12g089330.2	1.30	synaptotagmin-1	SYT1	Maintains plasma membrane integrity during freezing and osmotic stresses; Functions in membrane resealing during calcium-dependent freezing tolerance; Regulates endocytosis and endosome recycling at the plasma membrane and cell-to-cell trafficking	*Impaired freezing response; Impaired cell-to-cell trafficking*	Yamazaki, Tomokazu, Naoki Takata, Matsuo Uemura, and Yukio Kawamura. "Arabidopsis synaptotagmin SYT1, a type I signal-anchor protein, requires tandem C2 domains for delivery to the plasma membrane." Journal of Biological Chemistry 285, no. 30 (2010): 23165-23176.
Solyc04g053000.1	1.27	auxin-responsive protein SAUR21-like	SAUR21	Positive effectors of cell expansion through modulation of auxin transport	*Impaired plant cell wall expansion; Impaired auxin transport*	Osakabe, Yuriko, Kyonoshin Maruyama, Motoaki Seki, Masakazu Satou, Kazuo Shinozaki, and Kazuko Yamaguchi-Shinozaki. "Leucine-rich repeat receptor-like kinase1 is a key membrane-bound regulator of abscisic acid early signaling in Arabidopsis." The Plant Cell 17, no. 4 (2005): 1105-1119.
Solyc02g087190.1	1.22	peroxidase 63	PER63	Involved in oxidation of toxic reductants, lignin metabolism, suberization, auxin catabolism; Involved in response to environmental stresses	*Impaired plant defense; Impaired lignin metabolism; Impaired auxin catabolism*	Valério, Luisa, Mireille De Meyer, Claude Penel, and Christophe Dunand. "Expression analysis of the Arabidopsis peroxidase multigenic family." Phytochemistry 65, no. 10 (2004): 1331-1342.

TomatoID	DE in LsoB	NCBI Protein Name	Gene ID	Uniprot Description	Putative Consequences for Infection	Citation
Solyc04g017720.3	1.18	protein GAST1-like	N/A	Involved in the gibberellic acid mediated signaling pathway	*Impaired gibberellic acid mediated signaling*	Haas, Brian J., Natalia Volfovsky, Christopher D. Town, Maxim Troukhan, Nickolai Alexandrov, Kenneth A. Feldmann, Richard B. Flavell, Owen White, and Steven L. Salzberg. "Full-length messenger RNA sequences greatly improve genome annotation." Genome biology 3, no. 6 (2002): 1-12.
Solyc10g081810.2	1.14	putative phosphatidylglycerol/phosphatidylinositol transfer protein DDB_G0278295	DDB_G0278295	Involved in sterol transport	*Impaired sterol transport*	Sillo, Alessio, Gareth Bloomfield, Alessandra Balest, Alessandra Balbo, Barbara Pergolizzi, Barbara Peracino, Jason Skelton, Alasdair Ivens, and Salvatore Bozzaro. "Genome-wide transcriptional changes induced by phagocytosis or growth on bacteria in Dictyostelium." BMC genomics 9, no. 1 (2008): 1-22.
Solyc09g055940.3	1.12	probable sodium/metabolite cotransporter BASS3, chloroplastic	BASS3	Functions as sodium-coupled metabolite transporter across the chloroplast envelope	*Impaired chloroplast transport*	Hanada, Kousuke, Yuji Sawada, Takashi Kuromori, Romy Klausnitzer, Kazuki Saito, Tetsuro Toyoda, Kazuo Shinozaki, Wen-Hsiung Li, and Masami Yokota Hirai. "Functional compensation of primary and secondary metabolites by duplicate genes in Arabidopsis thaliana." Molecular biology and evolution 28, no. 1 (2011): 377-382.
Solyc03g115380.2	1.12	UDP-glucose 6-dehydrogenase 1-like	UGD1	Involved in the biosynthesis of UDP-glucuronic acid, providing nucleotide sugars for cell-wall polymers	*Impaired nucleotide transport for cell-wall polymers*	Oka, Takuji, and Yoshifumi Jigami. "Reconstruction of de novo pathway for synthesis of UDP-glucuronic acid and UDP-xylose from intrinsic UDP-glucose in Saccharomyces cerevisiae." The FEBS journal 273.12 (2006): 2645-2657.
Solyc04g016480.3	1.10	protein IQ-DOMAIN 14	IQD14	Involved in cooperative interactions with calmodulins or calmodulin-like proteins; Scaffold in cellular signaling and trafficking; Regulates cell and organ shapes	*Impaired cell-to-cell signaling and trafficking; Impaired regulation of plant organ shape*	Bürstenbinder, Katharina, Birgit Möller, Romina Plötner, Gina Stamm, Gerd Hause, Dipannita Mitra, and Steffen Abel. "The IQD family of calmodulin-binding proteins links calcium signaling to microtubules, membrane subdomains, and the nucleus." Plant physiology 173, no. 3 (2017): 1692-1708.
Solyc10g076790.2	1.09	auxin transporter-like protein 4	LAX4	Carrier protein involved in proton-driven auxin influx	*Impaired auxin signaling*	de Billy, Françoise, Cathy Grosjean, Sean May, Malcolm Bennett, and Julie V. Cullimore. "Expression studies on AUX1-like genes in Medicago truncatula suggest that auxin is required at two steps in early nodule development." Molecular Plant-Microbe Interactions 14, no. 3 (2001): 267-277.
Solyc08g061930.3	1.07	cytokinin oxidase/dehydrogenase-like isoform X2	CKX2	Involved in cytokinin catabolism	*Impaired cytokinin catabolism; Impaired plant root/shoot development*	Werner, Tomáš, Václav Motyka, Valérie Laucou, Rafaël Smets, Harry Van Onckelen, and Thomas Schmülling. "Cytokinin-deficient transgenic Arabidopsis plants show multiple developmental alterations indicating opposite functions of cytokinins in the regulation of shoot and root meristem activity." The Plant Cell 15, no. 11 (2003): 2532-2550.
Solyc03g032040.3	1.05	monosaccharide-sensing protein 2-like	MSSP2	Involved in carbohydrate transport	*Impaired carbohydrate transport*	Wormit, Alexandra, Oliver Trentmann, Ingmar Feifer, Christian Lohr, Joachim Tjaden, Stefan Meyer, Ulrike Schmidt, Enrico Martinoia, and H. Ekkehard Neuhaus. "Molecular identification and physiological characterization of a novel monosaccharide transporter from Arabidopsis involved in vacuolar sugar transport." The Plant Cell 18, no. 12 (2006): 3476-3490.

TomatoID	DE in LsoB	NCBI Protein Name	Gene ID	Uniprot Description	Putative Consequences for Infection	Citation
Solyc09g015080.3	1.05	patellin-6	PATL6	Carrier protein involved in membrane-trafficking associated with cell-plate formation during cytokinesis; Involved in cellular division; Involved in auxin polar transport	*Impaired cellular division; Impaired auxin transport*	Zhou, Huapeng, Hongqin Duan, Yunhong Liu, Xia Sun, Jinfeng Zhao, and Honghui Lin. "Patellin protein family functions in plant development and stress response." Journal of plant physiology 234 (2019): 94-97.
Solyc04g053010.1	1.04	auxin-responsive protein SAUR68-like	SAUR68	Promote auxin-stimulated organ elongation, such as hypocotyls, stamen filaments and petals	*Impaired organ elongation; Impaired growth and development; Impaired auxin signaling; Impaired flower development*	Zhao, Yunde, Xinhua Dai, Helen E. Blackwell, Stuart L. Schreiber, and Joanne Chory. "SIR1, an upstream component in auxin signaling identified by chemical genetics." Science 301, no. 5636 (2003): 1107-1110.
Solyc05g012790.3	1.03	probable protein S-acyltransferase 22	PAT22	Palmitoyl acyltransferase	*Impaired signaling and transport*	Hemsley, Piers, and Claire Grierson. "S-acylation: dynamic control of plant development and sigalling by lipid modification of proteins." In 18th International Conference on Arabidopsis Research (第十八届国际拟南芥大会), pp. 38-38. 中国科学院, 2007.
Solyc04g012030.3	1.01	DHHC-type zinc finger family protein	dhhc-4	Involved in peptidyl-L-cysteine S-palmitoylation	*Impaired signaling and transport*	McKay, S. J., R. Johnsen, J. Khattra, J. Asano, D. L. Baillie, S. Chan, N. Dube et al. "Gene expression profiling of cells, tissues, and developmental stages of the nematode C. elegans." In Cold Spring Harbor symposia on quantitative biology, vol. 68, pp. 159-170. Cold Spring Harbor Laboratory Press, 2003.
Solyc02g084950.3	-1.11	jasmonate O-methyltransferase isoform X2	JMT	Catalyzes the methylation of jasmonate into methyljasmonate, a plant volatile that acts as an important cellular regulator mediating diverse developmental processes and defense responses	Promoted methyljasmonate-regulated signaling; Promoted defenese	Seo, Hak Soo, Jong Tae Song, Jong-Joo Cheong, Yong-Hwan Lee, Yin-Won Lee, Ingyu Hwang, Jong Seob Lee, and Yang Do Choi. "Jasmonic acid carboxyl methyltransferase: a key enzyme for jasmonate-regulated plant responses." Proceedings of the National Academy of Sciences 98, no. 8 (2001): 4788-4793.
Solyc11g066060.2	-1.14	heat shock cognate 70 kDa protein 2-like	HSP70-2	Facilitate folding of de novo synthesized proteins, assist translocation of precursor proteins into organelles, and are responsible for degradation of damaged protein under stress conditions	Promoted folding of de novo synthesized proteins; Promoted protein transport; Promoted degradation of damanged proteins under stress	Hilson, Pierre, Joke Allemeersch, Thomas Altmann, Sébastien Aubourg, Alexandra Avon, Jim Beynon, Rishikesh P. Bhalerao et al. "Versatile gene-specific sequence tags for Arabidopsis functional genomics: transcript profiling and reverse genetics applications." Genome research 14, no. 10b (2004): 2176-2189.

TomatoID	DE in LsoB	NCBI Protein Name	Gene ID	Uniprot Description	Putative Consequences for Infection	Citation
Solyc01g111640.3	-1.14	SKP1-like protein 1A	SKP1A	Involved in ubiquitination; Required for vegetative and floral organ development and male gametogenesis; Involved in auxin signaling pathway; Regulates responses to jasmonates; Involved in light-signaling and the circadian clock	Promoted protein ubiquitination; Promoted floral development and male gametogenesis; Promoted auxin signaling; Promoted circadian clock; Promoted embryogenesis	Zhao, Dazhong, Weimin Ni, Baomin Feng, Tianfu Han, Megan G. Petrasek, and Hong Ma. "Members of the Arabidopsis-SKP1-like gene family exhibit a variety of expression patterns and may play diverse roles in Arabidopsis." Plant physiology 133, no. 1 (2003): 203-217.
Solyc02g063360.3	-1.16	protein C2-DOMAIN ABA-RELATED 1	CAR1	Mediates the transient calcium-dependent interaction of PYR/PYL/RCAR abscisic acid receptors with the plasma membrane	Promoted abscisic acid signaling	Rodriguez, Lesia, Miguel Gonzalez-Guzman, Maira Diaz, Americo Rodrigues, Ana C. Izquierdo-Garcia, Marta Peirats-Llobet, Maria A. Fernandez et al. "C2-domain abscisic acid-related proteins mediate the interaction of PYR/PYL/RCAR abscisic acid receptors with the plasma membrane and regulate abscisic acid sensitivity in Arabidopsis." The Plant Cell 26, no. 12 (2014): 4802-4820.
Solyc08g082740.3	-1.19	signal recognition particle 19 kDa protein	SRP19	Part of the signal-recognition-particle assembly, binds directly to 7S RNA and mediates binding of the 54 kDa subunit of the SRP	Promoted cell-to-cell signaling	Wang, Yi, Wen-Zheng Zhang, Lian-Fen Song, Jun-Jie Zou, Zhen Su, and Wei-Hua Wu. "Transcriptome analyses show changes in gene expression to accompany pollen germination and tube growth in Arabidopsis." Plant physiology 148, no. 3 (2008): 1201-1211.
Solyc07g043310.3	-1.27	gamma aminobutyrate transaminase 1, mitochondrial	GABA-TP1	Transaminase that degrades GABA and uses pyruvate or glyoxylate as amino-group acceptor	Promoted signaling turnover	Clark, Shawn M., Rosa Di Leo, Owen R. Van Cauwenberghe, Robert T. Mullen, and Barry J. Shelp. "Subcellular localization and expression of multiple tomato γ-aminobutyrate transaminases that utilize both pyruvate and glyoxylate." Journal of experimental botany 60, no. 11 (2009): 3255-3267.
Solyc07g065320.3	-1.41	ABC transporter C family member 3	ABCC3	Pump for glutathione S-conjugates and chlorophyll catabolites; Heavy metal transporter	Increased glutathione S-conjugate and chlorophyll catabolite transport; Increased heavy metal transport	Tommasini, Roberto, Esther Vogt, Myriam Fromenteau, Stefan Hörtensteiner, Philippe Matile, Nikolaus Amrhein, and Enrico Martinoia. "An ABC-transporter of Arabidopsis thaliana has both glutathione-conjugate and chlorophyll catabolite transport activity." The Plant Journal 13, no. 6 (1998): 773-780.
Solyc11g010380.2	-1.41	protein DETOXIFICATION 27	DTX27	Has xenobiotic transmembrane transporter activity	Promoted xenobiotic transport	Ascencio-Ibánez, José Trinidad, Rosangela Sozzani, Tae-Jin Lee, Tzu-Ming Chu, Russell D. Wolfinger, Rino Cella, and Linda Hanley-Bowdoin. "Global analysis of Arabidopsis gene expression uncovers a complex array of changes impacting pathogen response and cell cycle during geminivirus infection." Plant physiology 148, no. 1 (2008): 436-454.
Solyc04g071890.3	-1.47	peroxidase 12 precursor	PER12	Involved in removal of H2O2, oxidation of toxic reductants, biosynthesis and degradation of lignin, suberization, auxin catabolism, and response to environmental stressors	Increased removal of H2O2, oxidation of toxic reductants, biosynthesis and degradation of lignin; Increased suberization, auxin catabolism, and response to environmental stressors	Paynel, Florence, et al. "Temporal regulation of cell-wall pectin methylesterase and peroxidase isoforms in cadmium-treated flax hypocotyl." Annals of botany 104.7 (2009): 1363-1372.

TomatoID	DE in LsoB	NCBI Protein Name	Gene ID	Uniprot Description	Putative Consequences for Infection	Citation
Solyc08g078870.2	-1.49	14 kDa proline-rich protein DC2.15-like	N/A	Involved in the initiation of embryogenesis and the metabolic changes related to the removal of auxins	Promoted embryogenesis; Promoted auxin removal	Aleith, F., and G. Richter. "Gene expression during induction of somatic embryogenesis in carrot cell suspensions." Planta 183, no. 1 (1991): 17-24.
Solyc11g066100.2	-1.56	heat shock cognate 70 kDa protein	HSP70-1	Facilitate folding of de novo synthesized proteins, assist translocation of precursor proteins into organelles, and are responsible for degradation of damaged protein under stress conditions	Promoted folding of de novo synthesized proteins; Promoted protein transport; Promoted degradation of damanged proteins under stress	Sung, Dong Yul, and Charles L. Guy. "Physiological and molecular assessment of altered expression of Hsc70-1 in Arabidopsis. Evidence for pleiotropic consequences." Plant Physiology 132, no. 2 (2003): 979-987.
Solyc09g089930.2	-1.57	ethylene responsive element binding protein	EREBP1	Involved in defense response; Involved in ethylene-activated signaling pathway	Increased defense response; Increased ethylene-responsive signaling	Horvath, Diana M., Dorothy J. Huang, and Nam-Hai Chua. "Four classes of salicylate-induced tobacco genes." Molecular plant-microbe interactions 11.9 (1998): 895-905.
Solyc12g005720.1	-1.63	cysteine-rich repeat secretory protein 38	CRRSP38	Involved in response to abscisic acid	Promoted response to abscisic acid signaling	Huang, Kai-Chau, Wei-Chih Lin, and Wan-Hsing Cheng. "Salt hypersensitive mutant 9, a nucleolar APUM23 protein, is essential for salt sensitivity in association with the ABA signaling pathway in Arabidopsis." BMC plant biology 18, no. 1 (2018): 1-21.
Solyc07g006370.1	-1.75	cation/calcium exchanger 1-like	CCX1	Involved in potassium and sodium ion transport	Promoted potassium and sodium ion transport	Shigaki, T., I. Rees, L. Nakhleh, and K. D. Hirschi. "Identification of three distinct phylogenetic groups of CAX cation/proton antiporters." Journal of Molecular Evolution 63, no. 6 (2006): 815-825.
Solyc07g065380.4	-1.82	zinc transporter-like precursor	ZIP1	Mediates copper, cadmium, and zinc uptake from the rhizosphere	Promoted copper, cadmium, and zinc uptake from the rhizosphere	Grotz, Natasha, Tama Fox, Erin Connolly, Walter Park, Mary Lou Guerinot, and David Eide. "Identification of a family of zinc transporter genes from Arabidopsis that respond to zinc deficiency." Proceedings of the National Academy of Sciences 95, no. 12 (1998): 7220-7224.
Solyc10g076550.1	-1.90	wall-associated receptor kinase 2-like	WAK2	Serine/threonine-protein kinase that functions as a signaling receptor of extracellular matrix component; Involved in the control of cell expansion, morphogenesis, and development	Promoted signaling turnover; Promoted cell expansion, morphogenesis, and development	Wagner, Tanya A., and Bruce D. Kohorn. "Wall-associated kinases are expressed throughout plant development and are required for cell expansion." The Plant Cell 13, no. 2 (2001): 303-318.
Solyc09g007520.3	-2.27	peroxidase 21	PER21	Involved in removal of H2O2, oxidation of toxic reductants, biosynthesis and degradation of lignin, suberization, auxin catabolism, and response to environmental stressors	Increased removal of H2O2, oxidation of toxic reductants, biosynthesis and degradation of lignin, suberization, auxin catabolism, and response to environmental stressors	Mosher, Rebecca A., Wendy E. Durrant, Dong Wang, Junqi Song, and Xinnian Dong. "A comprehensive structure–function analysis of Arabidopsis SNI1 defines essential regions and transcriptional repressor activity." The Plant Cell 18, no. 7 (2006): 1750-1765.

TomatoID	DE in LsoB	NCBI Protein Name	Gene ID	Uniprot Description	Putative Consequences for Infection	Citation
Solyc12g096570.1	-2.52	protein AUXIN-REGULATED GENE	ARGOS	Promotes cell proliferation-dependent organ growth; Takes part in the AXR1-	Promoted organ growth; Promoted auxin signaling	Hu, Yuxin, Qi Xie, and Nam-Hai Chua. "The Arabidopsis auxin-inducible gene ARGOS controls lateral organ size." The Plant Cell 15, no. 9 (2003): 1951-1961.
Solyc09g091000.3	-4.16	pathogenesis-related protein STH-2	STH-2	Regulates protein serein/threonine phosphate activity; Involved in abscisic acid-activated signaling pathway; Involved in defense response to biotic stimulus	Promoted absisic acid signaling; Promoted defense against biotic stimuli	Matton, Daniel P., Gary Prescott, Charles Bertrand, Anne Camirand, and Normand Brisson. "Identification of cis-acting elements involved in the regulation of the pathogenesis-related gene STH-2 in potato." Plant molecular biology 22, no. 2 (1993): 279-291.
