# Supplementary material for: Effects of ‘Candidatus Liberibacter solanacearum’ haplotypes A and B on tomato gene expression and geotropism
Source: BMC Plant Biol. 2022 Mar 30;22:156. doi: 10.1186/s12870-022-03505-z (PMC8966271; doi:10.1186/s12870-022-03505-z)
Supplement: Supplementary file 15 — Additional file 15: Table S15. The 50 tomato plant DEGs associated with either carbohydrate metabolism or photosynthesis under infection by different Lso haplotypes. DEGs were sorted by differential expression (DE) values comparing uninfected to infected samples (p-value < 0.05). NCBI Blast searches were used to identify Gene IDs and protein products in tomatoes as well as their homologs in other species. Specifically, the expression changes in 26 of the 34-carbohydrate related genes (76%, in italics) would have resulted in impaired carbohydrate metabolism in LsoB-infected plants relative to LsoA-infected plants, while every change among the photosynthesis-related genes (100%, in italics) would have resulted in impaired photosynthesis in LsoB-infected plants. These DEGs were predominantly related to cell wall-related carbohydrates, sugar biosynthesis, and essential components of photosynthesis. [file 12870_2022_3505_MOESM15_ESM.pdf]

TomatoID	DE in LsoB	NCBI Protein Name	Gene ID	Uniprot Description	Putative Consequences for Infection	Citation
Solyc03g120990.3	2.09	NADP-dependent malic enzyme, chloroplastic	ME6	Cecarboxylates malate shuttled from neighboring mesophyll cells; CO2 released is refixed by RuBisCo; Eliminates the photorespiratory loss of CO2 that occurs in most plants	*Impaired photorespiratory efficiency of CO2 loss*	Cheng, Yuxiang, et al. "Expression, purification, and characterization of two NADP-malic enzymes of rice (Oryza sativa L.) in Escherichia coli." Protein expression and purification 45.1 (2006): 200-205.
Solyc12g039030.1	1.97	photosystem II protein D1	psbA	Photosynthetic electron transporter in photosystem II	*Impaired photosynthesis*	Tsunoyama, Yuichi, Yoko Ishizaki, Kazuya Morikawa, Maki Kobori, Yoichi Nakahira, Go Takeba, Yoshinori Toyoshima, and Takashi Shiina. "Blue light-induced transcription of plastid-encoded psbD gene is mediated by a nuclear-encoded transcription initiation factor, AtSig5." Proceedings of the National Academy of Sciences 101, no. 9 (2004): 3304-3309.
Solyc05g016120.2	1.95	photosystem II protein D1	psbA	Photosynthetic electron transporter in photosystem II	*Impaired photosynthesis*	Tsunoyama, Yuichi, Yoko Ishizaki, Kazuya Morikawa, Maki Kobori, Yoichi Nakahira, Go Takeba, Yoshinori Toyoshima, and Takashi Shiina. "Blue light-induced transcription of plastid-encoded psbD gene is mediated by a nuclear-encoded transcription initiation factor, AtSig5." Proceedings of the National Academy of Sciences 101, no. 9 (2004): 3304-3309.
Solyc01g048590.2	1.93	photosystem II protein D1	psbA	Photosynthetic electron transporter in photosystem II	*Impaired photosynthesis*	Tsunoyama, Yuichi, Yoko Ishizaki, Kazuya Morikawa, Maki Kobori, Yoichi Nakahira, Go Takeba, Yoshinori Toyoshima, and Takashi Shiina. "Blue light-induced transcription of plastid-encoded psbD gene is mediated by a nuclear-encoded transcription initiation factor, AtSig5." Proceedings of the National Academy of Sciences 101, no. 9 (2004): 3304-3309.
Solyc09g008320.3	1.92	probable xyloglucan endotransglucosylase/hydrolase protein 32	XTH32	Cleaves and religates xyloglucan polymers, an essential constituent of the primary cell wall, and thereby participates in cell wall construction of growing tissues	*Impaired plant cell wall construction*	Bischoff, Volker, Sarah Jane Cookson, Shuang Wu, and Wolf-Rüdiger Scheible. "Thaxtomin A affects CESA-complex density, expression of cell wall genes, cell wall composition, and causes ectopic lignification in Arabidopsis thaliana seedlings." Journal of experimental botany 60, no. 3 (2009): 955-965.
Solyc05g005550.3	1.92	polygalacturonase non-catalytic subunit AroGP2 precursor	GP2	Non-catalytic subunit of polygalacturonase involved in cell wall organization	*Impaired plant cell wall organization*	N/A
Solyc04g063210.3	1.88	probable caffeoyl-CoA O-methyltransferase At4g26220 isoform X2	AT4G26220	Plays a role in the synthesis of feruloylated polysaccharides; Involved in the reinforcement of the plant cell wall; Involved in response to wounding and/or pathogen challenge	*Impaired reinforcement of plant cell walls; Impaired response to wounding and/or pathogen challenge*	Meyermans, Hugo, et al. "Modifications in lignin and accumulation of phenolic glucosides in poplar xylem upon down-regulation of caffeoyl-coenzyme A O-methyltransferase, an enzyme involved in lignin biosynthesis." Journal of Biological Chemistry 275.47 (2000): 36899-36909.

TomatoID	DE in LsoB	NCBI Protein Name	Gene ID	Uniprot Description	Putative Consequences for Infection	Citation
Solyc11g056340.1	1.80	photosystem II protein D1	psbA	Photosynthetic electron transporter in photosystem II	*Impaired photosynthesis*	Tsunoyama, Yuichi, Yoko Ishizaki, Kazuya Morikawa, Maki Kobori, Yoichi Nakahira, Go Takeba, Yoshinori Toyoshima, and Takashi Shiina. "Blue light-induced transcription of plastid-encoded psbD gene is mediated by a nuclear-encoded transcription initiation factor, AtSig5." Proceedings of the National Academy of Sciences 101, no. 9 (2004): 3304-3309.
Solyc05g014000.3	1.73	probable pectate lyase 5	At1g67750	Part of the pathway pectin degradation, which is itself part of Glycan metabolism	*Impaired pectin degradation*	Pischke, Melissa S., Edward L. Huttlin, Adrian D. Hegeman, and Michael R. Sussman. "A transcriptome-based characterization of habituation in plant tissue culture." Plant Physiology 140, no. 4 (2006): 1255-1278.
Solyc02g066970.1	1.72	transcription factor PAR1	PAR1	Negative regulator of a variety of shade avoidance syndrome responses, including seedling elongation and photosynthetic pigment accumulation; Transcriptional repressor of two auxin-responsive genes	*Impaired shade avoidance; Impaired repression of auxin signaling*	Roig-Villanova, Irma, Jordi Bou-Torrent, Anahit Galstyan, Lorenzo Carretero-Paulet, Sergi Portolés, Manuel Rodríguez-Concepción, and Jaime F. Martínez-García. "Interaction of shade avoidance and auxin responses: a role for two novel atypical bHLH proteins." The EMBO journal 26, no. 22 (2007): 4756-4767.
Solyc05g009470.3	1.70	alpha-xylosidase 1	XYL1	Glycoside hydrolase releasing xylosyl residues from xyloglucan oligosaccharides; Essential for growth/development	*Impaired growth/development*	Sampedro, Javier, et al. "Cloning and expression pattern of a gene encoding an α-xylosidase active against xyloglucan oligosaccharides from Arabidopsis." Plant Physiology 126.2 (2001): 910-920.
Solyc09g007940.3	1.70	adenosine kinase 2	ADK2	Essential to sustain methyl recycling	*Impaired methyl recycling*	Pereira, L. A. R., M. Todorova, X. Cai, C. A. Makaroff, R. J. N. Emery, and B. A. Moffatt. "Methyl recycling activities are co-ordinately regulated during plant development." Journal of experimental botany 58, no. 5 (2007): 1083-1098.
Solyc04g081300.3	1.70	endoglucanase 2	At1g19940	Involved in cellulose catabolism; Involved in cell wall organization	*Impaired cellulose catabolism; Impaired cell wall organization*	Brown, David M., Leo AH Zeef, Joanne Ellis, Royston Goodacre, and Simon R. Turner. "Identification of novel genes in Arabidopsis involved in secondary cell wall formation using expression profiling and reverse genetics." The Plant Cell 17, no. 8 (2005): 2281-2295.
Solyc03g096050.3	1.63	probable 2-oxoglutarate-dependent dioxygenase At5g05600	AT5G05600	Involved in anthocyanin and protoanthocyanidin biosynthesis	*Impaired anthocyanin and protoanthocyanidin biosynthesis*	Wang, Yi, et al. "Transcriptome analyses show changes in gene expression to accompany pollen germination and tube growth in Arabidopsis." Plant physiology 148.3 (2008): 1201-1211.
Solyc10g047430.1	1.61	ribulose 1,5-bisphosphate carboxylase, partial	RBCMT	Methylates 'Lys-14' of the large subunit of RuBisCO	*Impaired RuBisCO activity*	N/A

TomatoID	DE in LsoB	NCBI Protein Name	Gene ID	Uniprot Description	Putative Consequences for Infection	Citation
Solyc02g080160.3	1.51	probable xyloglucan endotransglucosylase/hydrolase protein 8 isoform X2	XTH8	Cleaves and religates xyloglucan polymers, an essential constituent of the primary cell wall, and thereby participates in cell wall construction of growing tissues	*Impaired plant cell wall growth/development*	Bischoff, Volker, Sarah Jane Cookson, Shuang Wu, and Wolf-Rüdiger Scheible. "Thaxtomin A affects CESA-complex density, expression of cell wall genes, cell wall composition, and causes ectopic lignification in Arabidopsis thaliana seedlings." Journal of experimental botany 60, no. 3 (2009): 955-965.
Solyc05g005560.4	1.41	polygalacturonase-1 non-catalytic subunit beta precursor	GP2	Non-catalytic subunit of polygalacturonase involved in cell wall organization	*Impaired plant cell wall organization*	N/A
Solyc07g052980.3	1.39	xyloglucan endotransglycosylase/hydrolase 16 precursor	XTH16	Cleaves and religates xyloglucan polymers, an essential constituent of the primary cell wall, and thereby participates in cell wall construction of growing tissues	*Impaired plant cell wall expansion*	Sasidharan, Rashmi, C. C. Chinnappa, Marten Staal, J. Theo M. Elzenga, Ryusuke Yokoyama, Kazuhiko Nishitani, Laurentius ACJ Voesenek, and Ronald Pierik. "Light quality-mediated petiole elongation in Arabidopsis during shade avoidance involves cell wall modification by xyloglucan endotransglucosylase/hydrolases." Plant physiology 154, no. 2 (2010): 978-990.
Solyc02g081060.3	1.32	chaperonin-like RBCX protein 1, chloroplastic	RBCX1	Chaperone involved in RuBisCO assembly process	*Impaired RuBisCO activity*	Kolesiński, Piotr, Janusz Piechota, and Andrzej Szczepaniak. "Initial characteristics of RbcX proteins from Arabidopsis thaliana." Plant molecular biology 77, no. 4 (2011): 447-459.
Solyc10g061830.2	1.32	photosystem II 44 kDa protein	psbC	Photosynthetic electron transporter in photosystem II	*Impaired photosynthesis*	Ahmed, Ibrar, Peter J. Matthews, Patrick J. Biggs, Muhammad Naeem, Patricia A. McLenachan, and Peter J. Lockhart. "Identification of chloroplast genome loci suitable for high-resolution phylogeographic studies of C olocasia esculenta (L.) S chott (A raceae) and closely related taxa." Molecular Ecology Resources 13, no. 5 (2013): 929-937.
Solyc08g082250.3	1.29	endo-beta-1,4-D-glucanase precursor	At3g13560	Involved in carbohydrate metabolism; Involved in defense response	*Impaired carbohydrate metabolism; Impaired defense response*	Jakoby, Marc J., Doris Falkenhan, Michael T. Mader, Ginger Brininstool, Elisabeth Wischnitzki, Nicole Platz, Andrew Hudson, Martin Hulskamp, John Larkin, and Arp Schnittger. "Transcriptional profiling of mature Arabidopsis trichomes reveals that NOECK encodes the MIXTA-like transcriptional regulator MYB106." Plant Physiology 148, no. 3 (2008): 1583-1602.
Solyc02g030480.3	1.28	probable cinnamyl alcohol dehydrogenase 6	CAD6	Involved in lignin biosynthesis	*Impaired lignin biosynthesis; Impaired plant cell wall growth/development*	Costa, Michael A., R. Eric Collins, Aldwin M. Anterola, Fiona C. Cochrane, Laurence B. Davin, and Norman G. Lewis. "An in silico assessment of gene function and organization of the phenylpropanoid pathway metabolic networks in Arabidopsis thaliana and limitations thereof." Phytochemistry 64, no. 6 (2003): 1097-1112.
Solyc02g087190.1	1.22	peroxidase 63	PER63	Involved in oxidation of toxic reductants, lignin metabolism, suberization, auxin catabolism; Involved in response to environmental stresses	*Impaired plant defense; Impaired lignin metabolism; Impaired auxin catabolism*	Valério, Luisa, Mireille De Meyer, Claude Penel, and Christophe Dunand. "Expression analysis of the Arabidopsis peroxidase multigenic family." Phytochemistry 65, no. 10 (2004): 1331-1342.

TomatoID	DE in LsoB	NCBI Protein Name	Gene ID	Uniprot Description	Putative Consequences for Infection	Citation
Solyc12g009110.2	1.20	acetylserotonin O-methyltransferase	ASMT	Methyltransferase which catalyzes the production of melatonin; Involved in response to light stress	*Impaired development under light stress*	Shi, Haitao, Yunxie Wei, and Chaozu He. "Melatonin-induced CBF/DREB1s are essential for diurnal change of disease resistance and CCA1 expression in Arabidopsis." Plant Physiology and Biochemistry 100 (2016): 150-155.
Solyc10g017940.1	1.18	photosystem II 44 kDa protein	psbC	Photosynthetic electron transporter in photosystem II	*Impaired photosynthesis*	Ahmed, Ibrar, Peter J. Matthews, Patrick J. Biggs, Muhammad Naeem, Patricia A. McLenachan, and Peter J. Lockhart. "Identification of chloroplast genome loci suitable for high-resolution phylogeographic studies of C olocasia esculenta (L.) S chott (A raceae) and closely related taxa." Molecular Ecology Resources 13, no. 5 (2013): 929-937.
Solyc11g066820.2	1.14	glucomannan 4-beta-mannosyltransferase 2	CSLA2	Possesses glucomannan synthase and mannan synthase activities; Galactomannan is a noncellulosic polysaccharides of plant cell wall	*Impaired plant cell wall growth/development*	Liepman, Aaron H., Curtis G. Wilkerson, and Kenneth Keegstra. "Expression of cellulose synthase-like (Csl) genes in insect cells reveals that CslA family members encode mannan synthases." Proceedings of the National Academy of Sciences 102, no. 6 (2005): 2221-2226.
Solyc01g006370.3	1.14	callose synthase 3-like	CALS3	Involved in callose synthesis at the forming cell plate during cytokinesis	*Impaired callose synthesis; Impaired cell proliferation*	Chen, Xiong-Yan, Lin Liu, EunKyoung Lee, Xiao Han, Yeonggil Rim, Hyosub Chu, Seon-Won Kim, Fred Sack, and Jae-Yean Kim. "The Arabidopsis callose synthase gene GSL8 is required for cytokinesis and cell patterning." Plant Physiology 150, no. 1 (2009): 105-113.
Solyc03g115380.2	1.12	UDP-glucose 6-dehydrogenase 1-like	UGD1	Involved in the biosynthesis of UDP-glucuronic acid, providing nucleotide sugars for cell-wall polymers	*Impaired nucleotide transport for cell-wall polymers*	Oka, Takuji, and Yoshifumi Jigami. "Reconstruction of de novo pathway for synthesis of UDP-glucuronic acid and UDP-xylose from intrinsic UDP-glucose in Saccharomyces cerevisiae." The FEBS journal 273.12 (2006): 2645-2657.
Solyc11g045260.1	1.11	photosystem II 44 kDa protein	psbC	Photosynthetic electron transporter in photosystem II	*Impaired photosynthesis*	Ahmed, Ibrar, Peter J. Matthews, Patrick J. Biggs, Muhammad Naeem, Patricia A. McLenachan, and Peter J. Lockhart. "Identification of chloroplast genome loci suitable for high-resolution phylogeographic studies of C olocasia esculenta (L.) S chott (A raceae) and closely related taxa." Molecular Ecology Resources 13, no. 5 (2013): 929-937.
Solyc06g009940.1	1.11	photosystem I P700 apoprotein A1	psaA	saA and PsaB bind P700, the primary electron donor of photosystem I; Necessary for photosynthesis	*Impaired photosynthesis*	Cournac, Laurent, Kevin Redding, Jacques Ravenel, Dominique Rumeau, Eve-Marie Josse, Marcel Kuntz, and Gilles Peltier. "Electron flow between photosystem II and oxygen in chloroplasts of photosystem I-deficient algae is mediated by a quinol oxidase involved in chlororespiration." Journal of Biological Chemistry 275, no. 23 (2000): 17256-17262.
Solyc04g014510.3	1.10	glutamine synthetase cytosolic isozyme 1-1	GLN1-1	High-affinity glutamine synthetase; Contributes to the homeostatic control of glutamine synthesis in roots	*Impaired glutamine homeostasis in the roots*	Ishiyama, Keiki, Eri Inoue, Akiko Watanabe-Takahashi, Mitsuhiro Obara, Tomoyuki Yamaya, and Hideki Takahashi. "Kinetic properties and ammonium-dependent regulation of cytosolic isoenzymes of glutamine synthetase in Arabidopsis." Journal of Biological Chemistry 279, no. 16 (2004): 16598-16605.

TomatoID	DE in LsoB	NCBI Protein Name	Gene ID	Uniprot Description	Putative Consequences for Infection	Citation
Solyc06g062580.3	1.10	beta-galactosidase-like	BGAL1	Involved in carbohydrate metabolic process	*Impaired carbohydrate metabolism*	Cartieaux, Fabienne, Céline Contesto, Adrien Gallou, Guilhem Desbrosses, Joachim Kopka, Ludivine Taconnat, Jean-Pierre Renou, and Bruno Touraine. "Simultaneous interaction of Arabidopsis thaliana with Bradyrhizobium sp. strain ORS278 and Pseudomonas syringae pv. tomato DC3000 leads to complex transcriptome changes." Molecular plant-microbe interactions 21, no. 2 (2008): 244-259.
Solyc12g009300.3	1.10	sucrose synthase	SUS1	Sucrose-cleaving enzyme that provides UDP-glucose and fructose for various metabolic pathways	*Impaired sugar metabolism*	Bieniawska, Zuzanna, D. H. Paul Barratt, Andrew P. Garlick, Vera Thole, Nicholas J. Kruger, Cathie Martin, Rita Zrenner, and Alison M. Smith. "Analysis of the sucrose synthase gene family in Arabidopsis." The Plant Journal 49, no. 5 (2007): 810-828.
Solyc01g017740.1	1.07	cytochrome b6	petB	Component of the cytochrome b6-f complex, which mediates electron transfer between photosystem II and photosystem I	*Impaired photosynthesis*	Felder, Susanne, Karin Meierhoff, Aniruddha P. Sane, Jörg Meurer, Christiane Driemel, Henning Plücken, Petra Klaff, Bernhard Stein, Nicole Bechtold, and Peter Westhoff. "The nucleus-encoded HCF107 gene of Arabidopsis provides a link between intercistronic RNA processing and the accumulation of translation-competent psbH transcripts in chloroplasts." The Plant Cell 13, no. 9 (2001): 2127-2141.
Solyc06g068770.3	1.06	probable beta-1,4-xylosyltransferase	IRX9H	Involved in the synthesis of the hemicellulose glucuronoxylan, a major component of secondary cell walls	*Impaired secondary plant cell wall biosynthesis*	Lee, Chanhui, Quincy Teng, Wenlin Huang, Ruiqin Zhong, and Zheng-Hua Ye. "The Arabidopsis family GT43 glycosyltransferases form two functionally nonredundant groups essential for the elongation of glucuronoxylan backbone." Plant Physiology 153, no. 2 (2010): 526-541.
Solyc03g032040.3	1.05	monosaccharide-sensing protein 2-like	MSSP2	Involved in carbohydrate transport	*Impaired carbohydrate transport*	Wormit, Alexandra, Oliver Trentmann, Ingmar Feifer, Christian Lohr, Joachim Tjaden, Stefan Meyer, Ulrike Schmidt, Enrico Martinoia, and H. Ekkehard Neuhaus. "Molecular identification and physiological characterization of a novel monosaccharide transporter from Arabidopsis involved in vacuolar sugar transport." The Plant Cell 18, no. 12 (2006): 3476-3490.
Solyc11g069270.2	1.02	beta-galactosidase 5	BGAL5	Involved in carbohydrate metabolic process	*Impaired carbohydrate metabolism*	Gantulga, Dashzeveg, Yusuf Turan, David R. Bevan, and Asim Esen. "The Arabidopsis At1g45130 and At3g52840 genes encode β-galactosidases with activity toward cell wall polysaccharides." Phytochemistry 69, no. 8 (2008): 1661-1670.
Solyc02g067360.3	1.02	protease Do-like 8, chloroplastic	DEGP8	Serine protease involved in photosystem II repair	*Impaired photosynthesis*	Giacomelli, Lisa, Andrea Rudella, and Klaas Jan van Wijk. "High light response of the thylakoid proteome in Arabidopsis wild type and the ascorbate-deficient mutant vtc2-2. A comparative proteomics study." Plant Physiology 141, no. 2 (2006): 685-701.

TomatoID	DE in LsoB	NCBI Protein Name	Gene ID	Uniprot Description	Putative Consequences for Infection	Citation
Solyc05g012510.3	1.01	alpha-1,4 glucan phosphorylase L-2 isozyme, chloroplastic/amyloplastic	N/A	Important allosteric enzyme in carbohydrate metabolism	*Impaired carbohydrate metabolism*	Lin, Chi-Tsai, Kai-Wun Yeh, Ping-Du Lee, and Jong-Ching Su. "Primary structure of sweet potato starch phosphorylase deduced from its cDNA sequence." Plant physiology 95, no. 4 (1991): 1250-1253.
Solyc02g077080.3	-1.07	protein trichome birefringence-like 38	TBL38	Bridging protein that binds pectin and other cell wall polysaccharides	Promoted pectin-binding; Promoted plant cell wall growth and development	Bischoff, Volker, Joachim Selbig, and Wolf-Rüdiger Scheible. "Involvement of TBL/DUF231 proteins into cell wall biology." Plant signaling & behavior 5, no. 8 (2010): 1057-1059.
Solyc05g046290.3	-1.14	probable xyloglucan endotransglucosylase/hydrolase protein 23	XTH23	Cleaves and religates xyloglucan polymers, an essential constituent of the primary cell wall, and thereby participates in cell wall construction of growing tissues	Promoted plant growth and development	Bischoff, Volker, Sarah Jane Cookson, Shuang Wu, and Wolf-Rüdiger Scheible. "Thaxtomin A affects CESA-complex density, expression of cell wall genes, cell wall composition, and causes ectopic lignification in Arabidopsis thaliana seedlings." Journal of experimental botany 60, no. 3 (2009): 955-965.
Solyc07g042550.3	-1.19	sucrose synthase	SUS1	Sucrose-cleaving enzyme that provides UDP-glucose and fructose for various metabolic pathways	Promoted sugar metabolism	Bieniawska, Zuzanna, D. H. Paul Barratt, Andrew P. Garlick, Vera Thole, Nicholas J. Kruger, Cathie Martin, Rita Zrenner, and Alison M. Smith. "Analysis of the sucrose synthase gene family in Arabidopsis." The Plant Journal 49, no. 5 (2007): 810-828.
Solyc09g059040.3	-1.37	fructose-bisphosphate aldolase	FBA1	Plays a key role in glycolysis and gluconeogenesis	Promoted glycolysis and glucogenesis	Searle, Iain R., Artem E. Men, Titeki S. Laniya, Diana M. Buzas, Inaki Iturbe-Ormaetxe, Bernard J. Carroll, and Peter M. Gresshoff. "Long-distance signaling in nodulation directed by a CLAVATA1-like receptor kinase." Science 299, no. 5603 (2003): 109-112.
Solyc03g113430.3	-1.37	protein NRT1/ PTR FAMILY 2.11	NPF2.11	High-affinity, proton-dependent glucosinolate-specific transporter; Involved in removal of glucosinolates from the xylem in roots	Promoted glucosinolate homeostasis	Nour-Eldin, Hussam Hassan, Tonni Grube Andersen, Meike Burow, Svend Roesen Madsen, Morten Egevang Jørgensen, Carl Erik Olsen, Ingo Dreyer, Rainer Hedrich, Dietmar Geiger, and Barbara Ann Halkier. "NRT/PTR transporters are essential for translocation of glucosinolate defence compounds to seeds." Nature 488, no. 7412 (2012): 531-534.
Solyc12g006380.2	-1.68	2-oxoglutarate-dependent dioxygenase	AOP3	Involved in glucosinolates biosynthesis	Promoted glucosinolate biosynthesis	Kliebenstein, Daniel J., Virginia M. Lambrix, Michael Reichelt, Jonathan Gershenzon, and Thomas Mitchell-Olds. "Gene duplication in the diversification of secondary metabolism: tandem 2-oxoglutarate–dependent dioxygenases control glucosinolate biosynthesis in Arabidopsis." The Plant Cell 13, no. 3 (2001): 681-693.
Solyc03g114890.3	-1.84	COBRA-like protein 4 isoform X2	COBL4	Involved in cellulose microfibril organization; Involved in plant cell wall cellulose biosynthesis; Invovled in secondary cell wall biogenesis	Promoted plant cell wall cellulose biosynthesis; Promoted scondary cell wall biogenesis	Brown, David M., Leo AH Zeef, Joanne Ellis, Royston Goodacre, and Simon R. Turner. "Identification of novel genes in Arabidopsis involved in secondary cell wall formation using expression profiling and reverse genetics." The Plant Cell 17, no. 8 (2005): 2281-2295.

TomatoID	DE in LsoB	NCBI Protein Name	Gene ID	Uniprot Description	Putative Consequences for Infection	Citation
Solyc09g007520.3	-2.27	peroxidase 21	PER21	Involved in removal of H2O2, oxidation of toxic reductants, biosynthesis and degradation of lignin, suberization, auxin catabolism, and response to environmental stressors	Increased removal of H2O2, oxidation of toxic reductants, biosynthesis and degradation of lignin, suberization, auxin catabolism, and response to environmental stressors	Mosher, Rebecca A., Wendy E. Durrant, Dong Wang, Junqi Song, and Xinnian Dong. "A comprehensive structure–function analysis of Arabidopsis SNI1 defines essential regions and transcriptional repressor activity." The Plant Cell 18, no. 7 (2006): 1750-1765.
Solyc03g033840.3	-2.57	AAA-ATPase At3g50940-like	At3g50940	Involved in lignin biosynthesis and response to salt stress	Promoted lignin biosynthesis; Promoted response to salt stress	van de Mortel, Judith E., Laia Almar Villanueva, Henk Schat, Jeroen Kwekkeboom, Sean Coughlan, Perry D. Moerland, Emiel Ver Loren van Themaat, Maarten Koornneef, and Mark GM Aarts. "Large expression differences in genes for iron and zinc homeostasis, stress response, and lignin biosynthesis distinguish roots of Arabidopsis thaliana and the related metal hyperaccumulator Thlaspi caerulescens." Plant physiology 142, no. 3 (2006): 1127-1147.
