# Supplementary figures and images for: Effects of ‘Candidatus Liberibacter solanacearum’ haplotypes A and B on tomato gene expression and geotropism
Source: BMC Plant Biol. 2022 Mar 30;22:156. doi: 10.1186/s12870-022-03505-z (PMC8966271; doi:10.1186/s12870-022-03505-z)

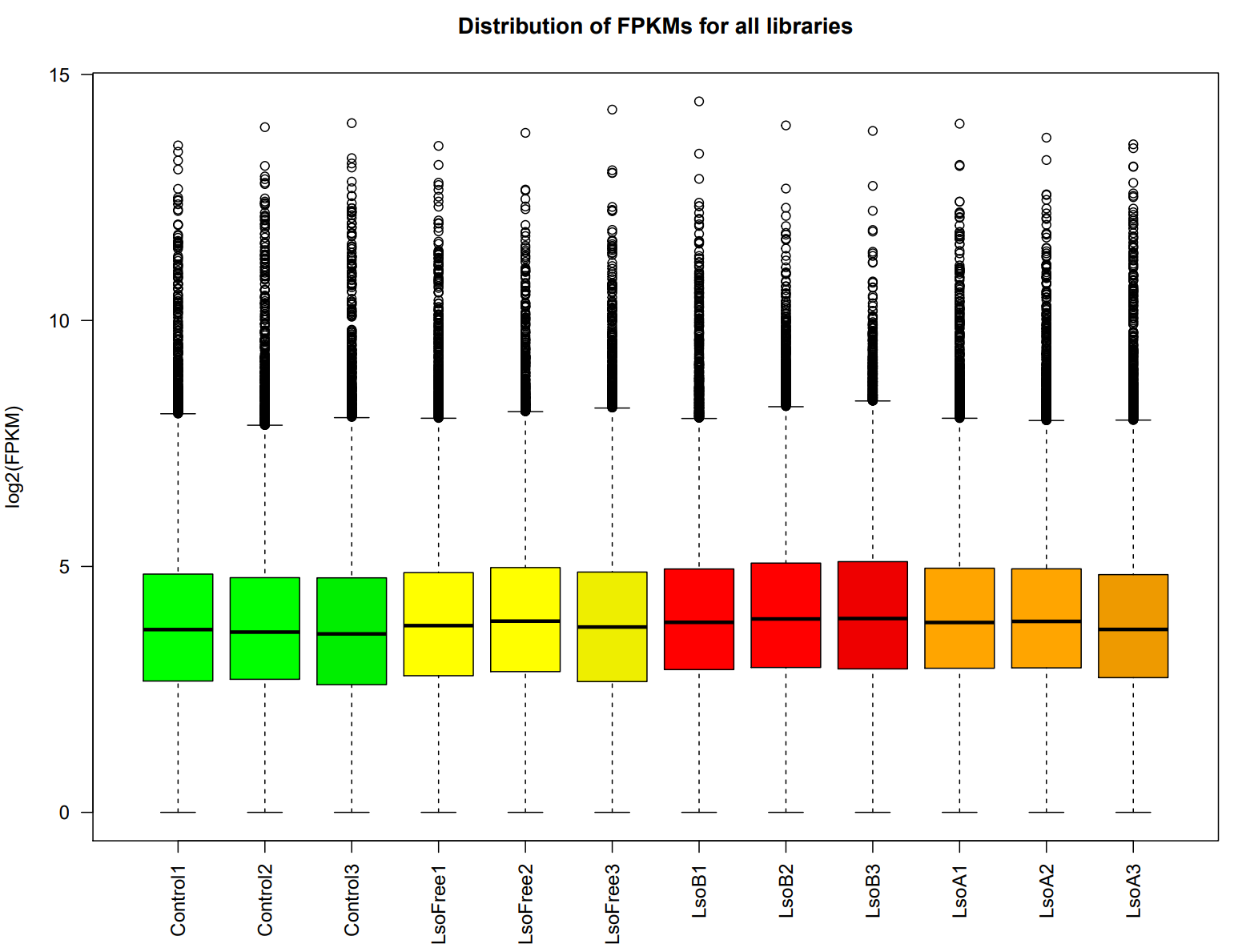

Supplement: Supplementary file 16 — Additional file 16: Figure S1. Distribution of log2 fragments per kilobase of transcript per million reads (fpkm) among among uninfested (Control#), Lso-free psyllid infested (LsoFree#), LsoB-infected (LsoB#), and LsoA-infected (LsoA#) tomato plant sample libraries. These values were not signficantly different between treatments. [file 12870_2022_3505_MOESM16_ESM.tiff]

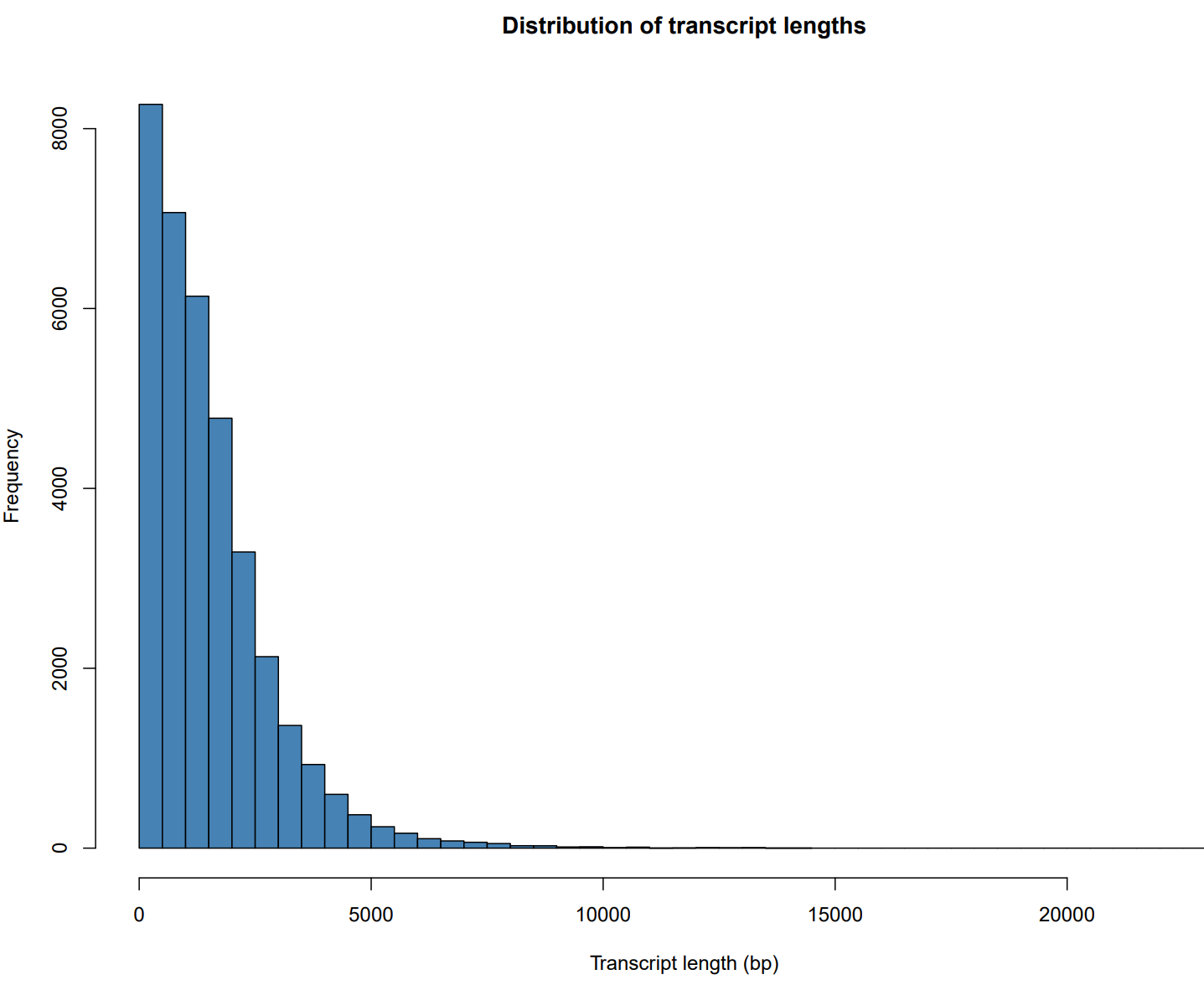

Supplement: Supplementary file 17 — Additional file 17: Figure S2. Distribution of transcript lengths across all samples and treatments. The X-axis depicts transcript lengths (bp), while the Y-axis depicts the relative frequency of those transcripts. [file 12870_2022_3505_MOESM17_ESM.tiff]

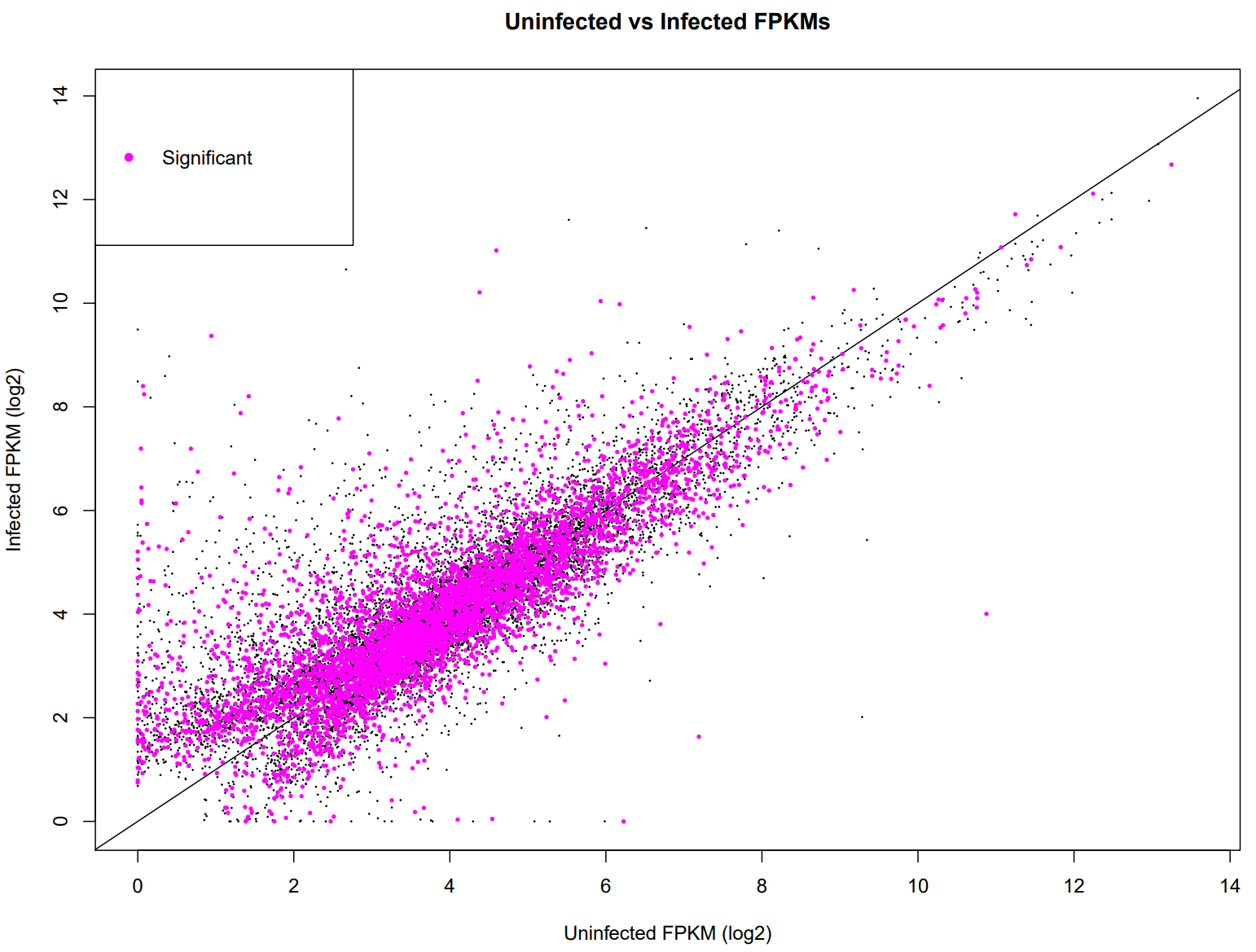

Supplement: Supplementary file 18 — Additional file 18: Figure S3. Per-gene comparison of log-transformed fragments per kilobase of transcript per million read (fpkm) values between uninfected (X-axis) and Lso-infected (Y-axis) tomato plants. Pink dots depict genes with significantly different fpkm values between uninfected and infected treatments. Black dots depict genes that do not have significantly different fpkm values between uninfected and infected plants. [file 12870_2022_3505_MOESM18_ESM.tiff]

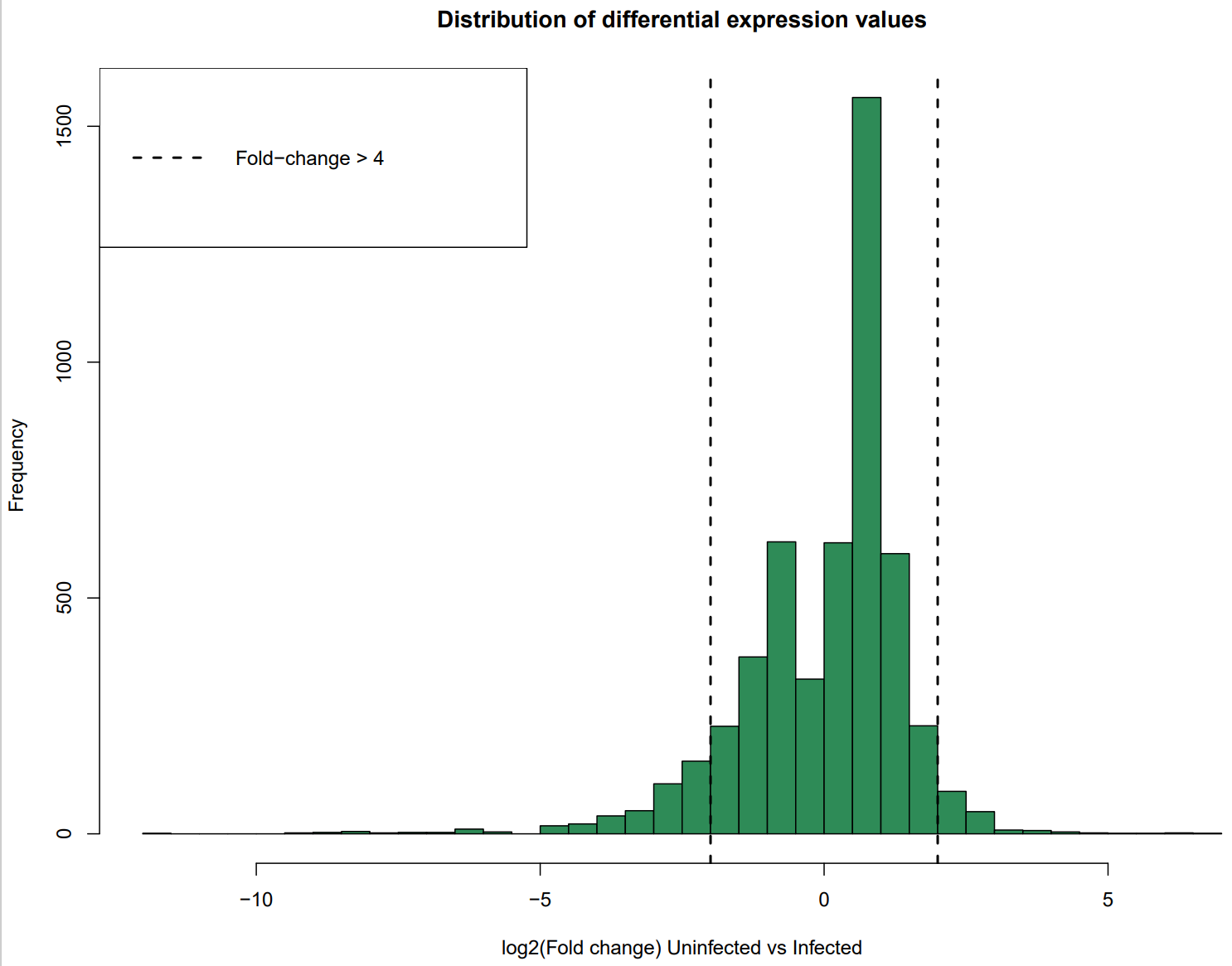

Supplement: Supplementary file 19 — Additional file 19: Figure S4. Distribution (in frequency) of differentially expressed genes (DEGs) across all libraries. The X-axis depicts the log-transformed fold change value of sequenced DEGs (relative to controls), while the Y-axis depicts the relative frequency of those DEGs. [file 12870_2022_3505_MOESM19_ESM.tiff]

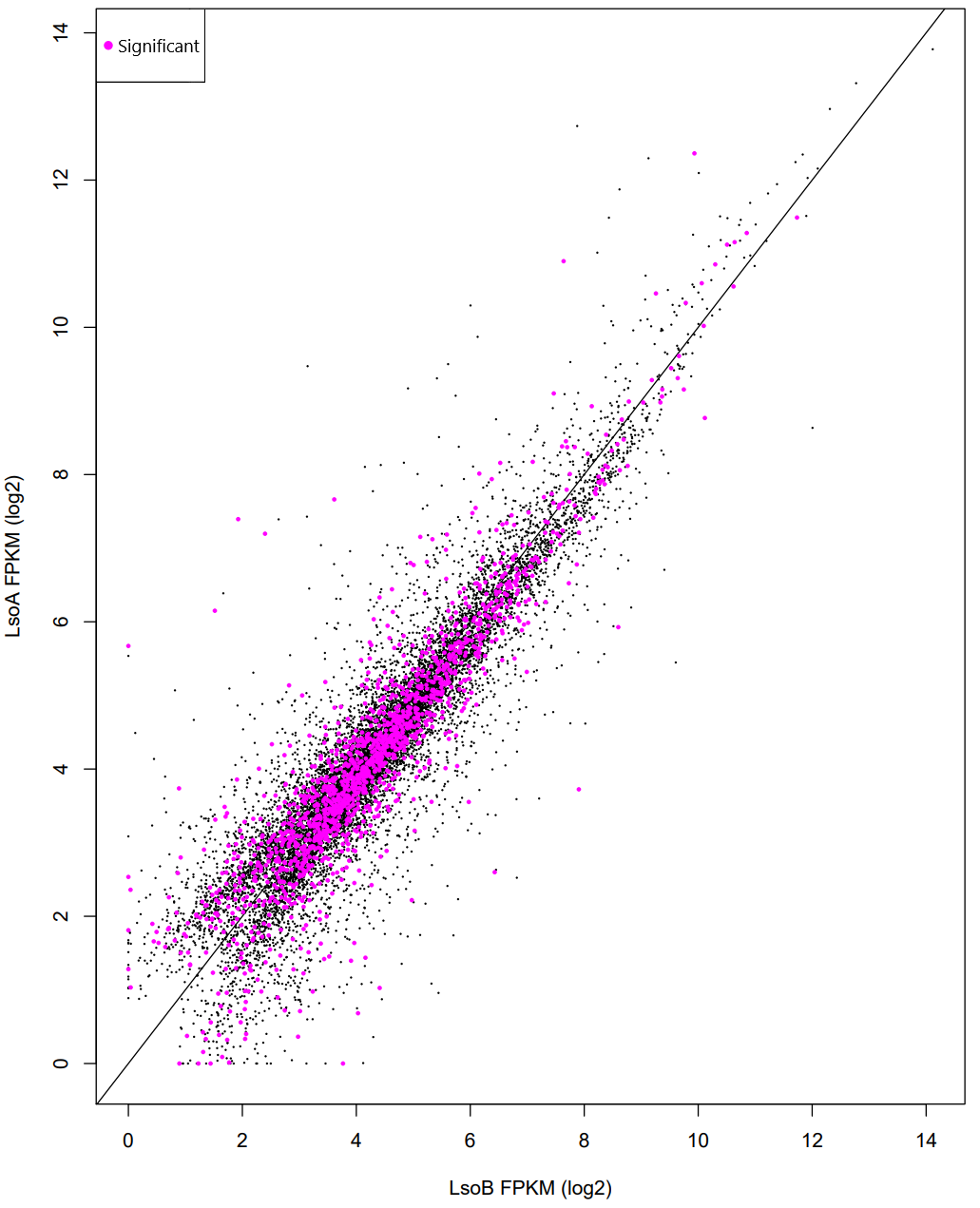

Supplement: Supplementary file 20 — Additional file 20: Figure S5. Per-gene comparison of log-transformed fragments per kilobase of transcript per million read (fpkm) values between LsoB- and LsoA-infected tomato plants. Pink dots depict genes with significantly different fpkm values between treatments infected with Lso haplotype B (X-axis) and treatments infected with Lso haplotype A (Y-axis). Black dots depict genes that do not have significantly different fpkm values. [file 12870_2022_3505_MOESM20_ESM.tiff]
